# Frequent loss of lineages and deficient duplications accounted for low copy number of disease resistance genes in Cucurbitaceae

**DOI:** 10.1186/1471-2164-14-335

**Published:** 2013-05-17

**Authors:** Xiao Lin, Yu Zhang, Hanhui Kuang, Jiongjiong Chen

**Affiliations:** 1 Key Laboratory of Horticulture Biology, Ministry of Education, and Department of Vegetable Crops, College of Horticulture and Forestry, Huazhong Agricultural University, Wuhan 430070, P.R. China

**Keywords:** *R*-genes, Cucurbitaceae, Copy number, Evolution, Sequence exchange

## Abstract

**Background:**

The sequenced genomes of cucumber, melon and watermelon have relatively few *R*-genes, with 70, 75 and 55 copies only, respectively. The mechanism for low copy number of *R*-genes in Cucurbitaceae genomes remains unknown.

**Results:**

Manual annotation of *R*-genes in the sequenced genomes of Cucurbitaceae species showed that approximately half of them are pseudogenes. Comparative analysis of *R*-genes showed frequent loss of *R*-gene loci in different Cucurbitaceae species. Phylogenetic analysis, data mining and PCR cloning using degenerate primers indicated that Cucurbitaceae has limited number of *R*-gene lineages (subfamilies). Comparison between *R*-genes from Cucurbitaceae and those from poplar and soybean suggested frequent loss of *R*-gene lineages in Cucurbitaceae. Furthermore, the average number of *R*-genes per lineage in Cucurbitaceae species is approximately 1/3 that in soybean or poplar. Therefore, both loss of lineages and deficient duplications in extant lineages accounted for the low copy number of *R*-genes in Cucurbitaceae. No extensive chimeras of *R*-genes were found in any of the sequenced Cucurbitaceae genomes. Nevertheless, one lineage of *R*-genes from *Trichosanthes kirilowii*, a wild Cucurbitaceae species, exhibits chimeric structures caused by gene conversions, and may contain a large number of distinct *R*-genes in natural populations.

**Conclusions:**

Cucurbitaceae species have limited number of *R*-gene lineages and each genome harbors relatively few *R*-genes. The scarcity of *R*-genes in Cucurbitaceae species was due to frequent loss of *R*-gene lineages and infrequent duplications in extant lineages. The evolutionary mechanisms for large variation of copy number of *R*-genes in different plant species were discussed.

## Background

The vast majority of the cloned disease resistance genes from plants encode nucleotide-binding site (NBS) and leucine-rich repeat (LRR) domains. The NBS-LRR proteins are often referred to as R proteins and their encoding genes as *R*-genes. R proteins can be further divided into two subclasses, the TIR (toll, interleukin receptor-like) subclass and the non-TIR subclass [1]. The TIR subclass proteins have the TIR domain in their N terminals, while most R proteins from the non-TIR subclass have a coiled-coil (CC) domain instead.

The *R*-genes in plants belong to a large gene family, and *R*-genes tend to be clustered in genomes. For instance, approximately 66% of the 149 *R*-genes in *Arabidopsis thaliana* (Col-0) and 76% of the 623 *R*-genes in rice (*Oryza sativa* cultivar Nipponbare) are located in clusters [2,3]. Many *R*-genes within a cluster belong to the same subfamily and may have had frequent sequence exchanges (either by gene conversion or recombination) resulting in chimeric structures [4-16]. Those chimeras, termed Type I *R*-genes, are highly diverse in different genotypes of a species, and consequently, a large number of *R*-genes with distinct sequences are predicted in a population/species [12,13,17]. Those chimeras were generated either by unequal crossovers or gene conversions. The frequent sequence exchanges among some Type I *R*-genes did not homogenize their coding sequences (i.e. no concerted evolution), though their intron sequences may be homogenized [12]. The lack of concerted evolution for the coding sequences of *R*-genes was likely due to diversifying selection after sequence exchanges [12].

In contrast to the extensively chimeric *R*-genes, other *R*-genes (termed Type II) evolved independently and did not have sequence exchanges with homologues. The sequences of Type II *R*-genes, when present, are highly conserved in different genotypes of the same or closely related species. Surprisingly, these highly “conserved” *R*-genes are frequently absent in some genotypes, showing presence/absence (P/A) polymorphism [3,12,17-20]. For example, 124 *R*-genes in two rice cultivars 93–11 and Nipponbare exhibit P/A polymorphism [3]. In the absence haplotypes, the entire Type II *R*-gene sequence is missing. Balancing selection may have played an important role in maintaining such P/A polymorphism [20,21]. The mechanism for such balancing selection remains poorly understood, but it is likely that the presence of some *R*-genes may have fitness cost such as low viability, low seed productions*, etc.*[22].

The number of *R*-genes in different plant genomes varies dramatically. Some genomes, such as the genomes of apple and wheat, contain approximately 1,000 *R*-genes [23,24]. In contrast, less than 100 *R*-genes are present in the sequenced genomes of papaya, cucumber, watermelon and melon, respectively [25-28]. It remains unclear why the number of *R*-genes varies considerably in different genomes while the total number of coding genes in a genome is relatively stable. Interestingly, the number of *R*-genes in a genome is significantly correlated with the number of LRR-LRK encoding genes, which may also be involved in disease resistance [29]. The identification and annotation of *R*-genes in a genome are challenging, simply because they are highly diverse and a considerable proportion of them are pseudogenes [2,30,31]. Large deletions (i.e. partial genes), frameshift indels or nonsense point mutations of *R*-genes make annotations using computer programs problematic. Consequently, many (the vast majority, in some cases) *R*-genes may be mis-annotated by gene prediction programs, and manual annotation is recommended to correct the errors [2].

The Cucurbitaceae family includes several agriculturally important crops such as melon (*Cucumis melo*), cucumber (*Cucumis sativus*), pumpkin (*Cucurbita moschata*) and watermelon (*Citrullus lanatus*). Disease is one of the main factors affecting their yields and forcing massive use of chemical sprays. Only one *R*-gene, *Fom-2* in melon, has been cloned from the Cucurbitaceae speies, while a candidate gene *Ccu* encoding resistance against cucumber scab was identified [32,33]. Recently, genomes of cucumber, melon and watermelon have been sequenced [26-28]. Only 61, 81 (*R*-genes plus genes encoding TIR only) and 44 *R*-genes were reported in the genomes of cucumber (9930), melon and watermelon, respectively. Low copy number of *R*-genes was also found in cucumber cultivar Gy14 [34]. The genetic mechanisms for such low copy number of *R*-genes in Cucurbitaceae species remain unclear. The *R*-genes from Cucurbitaceae genomes (except watermelon) were annotated using computer programs and were not verified manually. Thought the distribution of *R*-genes on cucumber chromosomes, *R*-gene sequences from other Cucurbitaceae species and phylogenetic comparison of *R*-genes from Cucurbitaceae and *Arabidopsis thaliana* were investigated in a previous study [34], the evolution of *R*-genes and the genetic mechanisms underlying low copy number of *R*-genes in Cucurbitaceae remain poorly understood.

In this study, *R*-genes in the sequenced genomes of cucumber, melon and watermelon were *de novo* identified and annotated. The structure (exon and intron) of each *R*-gene lineage in Cucurbitaceae was determined. The *R*-gene loci and *R*-gene sequences in different Cucurbitaceae species were compared. Degenerate primers were used to amplify *R*-genes from 9 species of Cucurbitaceae. The diversity of *R*-genes in cucumber and a wild Cucurbitaceae species, *Trichosanthes kirilowii*, was studied in detail. The genetic mechanisms for low copy number of *R*-genes were investigated through phylogenetic comparison of *R*-genes in Cucurbitaceae and those from poplar (*Populus trichocarpa*) and soybean (*Glycine max*). The evolutionary mechanisms for large variation of copy number of *R*-genes in different species were discussed.

## Results

### Low copy number of *R*-genes in Cucurbitaceae

Using HMMER, BLASTN search, and R protein database search (see MM section), 70, 71, 48, 75 and 55 *R*-genes were identified from the sequenced genomes of three cucumber cultivars (9930, Gy14 and B10), melon and watermelon, respectively. The number (71) of *R*-genes identified from cucumber inbred line Gy14 is considerably more than that (57) found in a previous study [34]. Nevertheless, the number of *R*-genes in different Cucurbitaceae genomes is quite similar but consistently low when compared with most sequenced plant genomes (Additional file 1: Table S1) [3,30,35-37].

### Eighteen gene models for all *R*-genes in Cucurbitaceae

**Figure 1:**
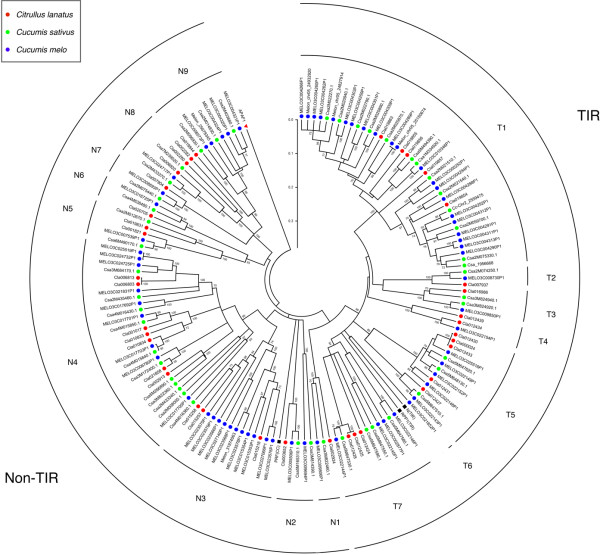
**Phylogenetic tree of NBS-LRR proteins from cucumber, melon and watermelon.** Most clades have R proteins from all three species, showing obvious orthologous relationship. Clade names starting with “T” are from TIR subclass, while clade names starting with “N” are from non-TIR subclass.

The amino acid sequences between the P-loop and GLPL motifs encoded by all identified *R*-genes from Cucurbitaceae were used to construct a Neighbor Joining (NJ) tree (Figure 1). As expected, the R proteins in the tree are divided into two major clades, one for TIR subclass and the other for non-TIR subclass. The TIR and non-TIR subclasses can be further divided into 7 and 9 clades (also called lineages or subfamilies hereafter), respectively. Each clade is supported by bootstrap value of greater than 80 and members between different clades, using distance of 0.3 as cutoff. Most (15 out of 16) clades contains R proteins from multiple species. For example, the N1 clade contains R proteins from all three species included in this study. However, clade T6 has only one protein from melon, suggesting deletions or sequencing gap.

**Figure 2:**
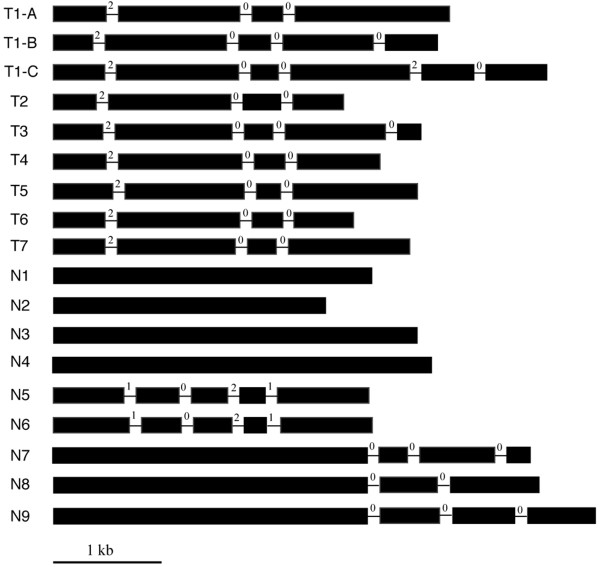
**Gene models for *R*-genes in Cucurbitaceae.** Filled boxes represent exons, while lines represent introns. The number right after an exon shows its intron phase. The bar represents the scale of exon length, while introns are not drawn to scale. Gene model name starting with T refers to TIR-type *R*-genes, while “N” refers to non-TIR *R*-genes.

All identified *R*-genes were annotated manually (Additional files 2 and 3). First, each *R*-gene sequence was used as query in BLASTX search of GenBank. If significant hits were found in non-Cucurbitaceae species, the corresponding sequences in the *R*-gene were considered to be exon sequences. The homologous *R*-genes/proteins in non-Cucurbitaceae species were then used as references for the annotation of the *R*-genes in Cucurbitaceae. Similar gene structure was expected for orthologous *R*-genes in Cucurbitaceae and closely related plant families such as Rosaseae, until proven otherwise. Then, homologous *R*-genes (within a clade in Figure 1) from Cucurbitaceae were aligned using MUSCLE, and full-length homologues with open reading frame (ORF) in the same clade are expected to have identical gene structures, including the number of exons/introns, intron position and intron phase [38]. After integrating above results, the final annotation was given for each gene. Eighteen gene models were provided for *R*-genes with full-length and ORF, one for each clade (Figure 2). The sequences of 18 genes, one representing each gene model are listed in Additional file 4. Of the 18 gene models, 14 are shared by *R*-genes in other plant families (see Additional file 4).

Three gene models (T1-A, T1-B and T1-C) represent 3 groups of genes that are closely related. The three groups share similar structures in the first 4 exons (with identical intron phase and similar exon size), but some homologues contain one or two additional exons (Figure 2). Interestingly, these three types of gene models are present in all sequenced genomes of Cucurbitaceae. Homology search showed that gene model T1-B (five exons) is conserved in other plant families (such as an *R*-gene from soybean, GenBank No. 100796191). Therefore, gene model T1-B is most likely ancient, while gene model T1-A (four exons) was a deletion derivative (exon 5 missing) of gene model T1-B. The exon 6 of gene model T1-C (six exons) has only weak homology (25% a.a. similarity) with an R protein from *Vitis vinifera* (GenBank No. XP_002269054). Surprisingly, the homologous part is located in the N terminal of this grape R protein. Therefore, gene model T1-C was most likely generated in Cucurbitaceae progenitor, through combining of T1-B with some coding sequences from another *R*-gene. The genes with gene model T1-A are mingled with genes with model T1-B and T1-C in the NJ tree, suggesting that the loss of exon 5 might have occurred independently in the genes with model T1-A.

Interestingly, all gene models (except T1-B and T1-C see above) representing TIR type *R*-genes in Cucurbitaceae are identical, while gene models for non-TIR type *R*-genes vary considerably (Figure 2). The 9 genes representing the non-TIR group are highly diverse, with only seven pairwise amino acid similarities of greater than 37%, while all 36 pairwise amino acid similarities for the TIR group are > 37%.

### A large proportion of *R*-genes in Cucurbitaceae genomes are pseudogenes

Using above gene models as references, 32 of the 70 *R*-genes in cucumber inbred line 9930, 38 of the 71 *R*-genes in cucumber Gy14, 38 of the 75 *R*-genes in melon and 24 of the 55 *R*-genes in watermelon were annotated as pseudogenes (Additional file 5). The pseudogenes were caused by large deletions (i.e. partial genes), frameshift insertions/deletions, or nonsense point mutations. Therefore, not only have the genomes of Cucurbitaceae species relatively few *R*-genes, a large proportion of them are pseudogenes.

Above annotations of *R*-genes in Cucurbitaceae were compared with those from previous studies [27,34]. As expected, no pseudogenes were annotated in cucumber inbred line Gy14 and melon, where annotation was done using computer programs. The annotations for 39 of 57 genes in cucumber cultivar Gy14, and 38 of 81 genes in melon in previous studies were likely wrong. Most of the errors included adding extra introns to remove premature stop codons or small frameshift indels, imprecise exon/intron boundaries and failure to recognize partial genes.

### An integrated *R*-gene map for Cucurbitaceae species

**Figure 3:**
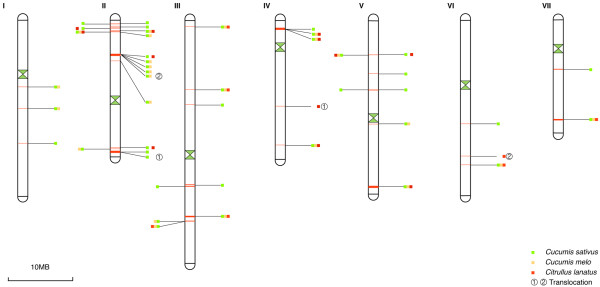
**An integrated map of *R *loci in Cucurbitaceae.***R*-genes from cucumber, melon and watermelon were mapped onto the 7 chromosomes of Cucumber cultivar 9930. *R* loci from cucumber, melon and watermelon were in green, yellow and red, respectively. Each *R* locus may contain several *R*-genes. The two translocation events are marked as numbers ① and ②.

Two *R*-genes that are interrupted by 8 or fewer non-*R*-genes, they are considered to be in the same cluster (also referred to as a multiple copy *R*-gene locus). The 70 *R*-genes from cucumber line 9930 are distributed at 32 loci, including 25 single-copy loci and 7 multiple-copy loci (i.e. clusters). Similarly, the 75 *R*-genes from melon are distributed at 18 loci, with the majority (61) in *R*-gene clusters; the 55 *R*-genes from watermelon are from 28 loci, with 38 in *R*-gene clusters. The position of each *R*-gene locus is shown in Figure 3. *R*-gene loci from melon and watermelon were mapped onto cucumber line 9930 chromosomes based on the synteny of their flanking regions, resulting in an integrated *R*-locus map for Cucurbitaceae (Figure 3). Note that 8 *R*-genes from melon could not be anchored to cucumber chromosomes and were not listed on the map. This integrated *R*-gene map will be a useful reference for future map-based cloning of resistance genes in Cucurbitaceae.

### Frequent loss of *R*-gene loci in Cucurbitaceae species

The integrated *R*-gene map shows that only 13 *R*-gene loci are present in all three Cucurbitaceae species. The other 32 loci are present in one or two genomes only, showing P/A polymorphism between different species. Cucumber and melon are closely related, which are distantly related with watermelon [39]. Cucumber and melon are identical (either absence or presence) at about half (24 of 43) *R*-gene loci, but have P/A polymorphisms at the other loci. The nucleotide identities between orthologous *R*-genes in cucumber and melon are usually higher than 90%, while nucleotide identities between *R*-genes at any two loci are lower than 90%. We conclude the P/A polymorphisms between cucumber and melon were caused by deletions (or translocations, see below) in one species rather than duplications in the other. At 4 loci, *R*-genes were present in cucumber and watermelon but not in melon, obviously due to deletions in melon, consistent with above conclusion. Among the 8 melon *R*-genes that could not be anchored onto cucumber chromosomes, two showed orthologous relationship with *R*-genes in cucumber according to bi-direction BLAST results. However, their flanking regions suggest that these two genes are not located in the syntenic regions of their “orthologues”, suggesting translocations.

A total of 28 loci exhibit P/A polymorphisms between cucumber/melon and watermelon. In all but two cases, *R*-genes are present in cucumber or melon but absent in watermelon. Orthologous relationship analysis suggests that these two *R*-gene loci in watermelon were translocated (alternatively, the corresponding two loci in cucumber/melon were translocated) (Figure 3). All other P/A polymorphisms were most likely caused by deletions or sequencing gap in watermelon.

### Low diversity of *R*-genes in different genotypes of the same species

At least 50 groups of *R*-genes from cucumber cultivars 9930, Gy14 and B10 showed obvious allelic relationships. The pairwise nucleotide identities between alleles range from 97.1%-100%, with an average of 99.1%. No recent sequence exchanges were detected between any *R*-genes in the three sequenced cucumber genotypes, i.e. no chimeric *R*-genes are found in cucumber. The high nucleotide identities of *R*-genes in cucumber are in marked contrast to the moderate nucleotide identities of some *R*-genes in other species, such as the *Rp1* genes in maize [3,10,12-14,17].

Similarly, none of the *R*-genes from the sequenced genomes of melon and watermelon showed extensive chimeric structures. To investigate if there is any extensive chimeras of *R*-genes in other Cucurbitaceae species, *R*-gene fragments were amplified from a panel of seven *T. kirilowii* genotypes from different natural populations. PCR primers were designed based on known *R*-genes from Cucurbitaceae. Fourteen primer combinations were used to amplify PCR products from the seven genotypes, and a total of 121 *R*-gene fragments were obtained [GenBank accession No: KC106898-KC107018]. All of them are closely related with *R*-genes from the sequenced genomes of Cucurbitaceae. They can be classified into 14 groups, and all except one group (see below) are obvious alleles, exhibiting higher than 95.5% nucleotide identity. No sequence exchanges were detected between different genes.

**Figure 4:**
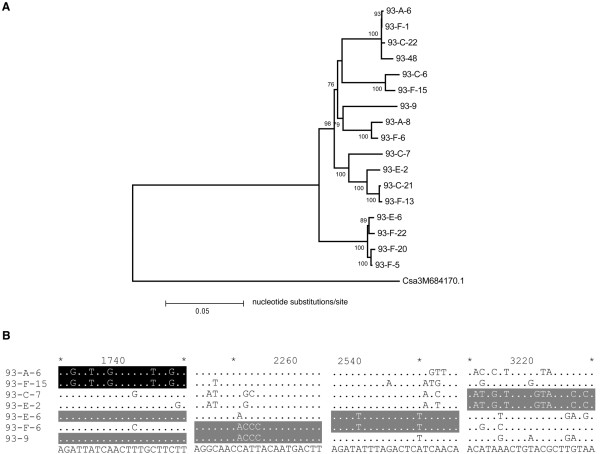
**Type I *R*-genes in *T. Kirilowii*.** (**A**) phylogenetic tree for the17 *R*-gene fragments amplified using primer combination GL-93, with Csa3M684170 from cucumber as an outgroup; (**B**) chimeric structure of the *R*-genes from *T. kirilowii*. Sequence exchange tracts are marked in the same color. Number represents the position in the sequences. Dots represent nucleotides identical to the consensus sequence.

The only exceptions are fragments amplified using primer combination GL-93, which was designed based on cucumber gene Csa3M684170. Seventeen fragments (2 from JS, 2 from DZ-1, 4 from NX-1, 2 from HD and 7 from XY) of 3,200 bp were obtained from five of the seven genotypes of *T. kirilowii* included in this study. Only weak bands were amplified from the remaining two genotypes (NX-2 and SD), probably due to poor DNA quality. The 17 fragments exhibited nucleotide identities of 88-98%, with an average of 93.1%. A NJ distance tree was constructed for these 17 fragments using their orthologues in cucumber as an outgroup (Figure 4A). No allelic relationship could be detected for most of the 17 genes. Further analysis showed that these genes have chimeric structure, a typical feature of Type I *R*-genes (Figure 4B). Seventeen sequence exchanges were detected among the 17 homologues using software Geneconv (*p* < 0.05). The exchange tracts varied from 174 to 1,755 bp, with an average of 515 bp. Since the chimeric sequences vary considerably in different genotypes, and a large number of distinct genes of this lineage are expected in natural populations of *T. kirilowii.*

### No new lineages obtained from Cucurbitaceae species using degenerate primer strategy

To investigate if there are additional *R*-gene lineages in Cucurbitaceae, 171 *R*-gene sequences from Cucurbitaceae species were retrieved from GenBank using Geneious [40]. These sequences were obtained mainly using degenerate primers for *R*-genes [34]. Of them, 160 sequences are highly similar (>70% nucleotide identity) to some *R*-genes from the three sequenced Cucurbitaceae genomes. The other 11 sequences [GenBank accession No. JN230607-JN230609, JN230645-JN230652] generated by Wan et al. [34] were most likely mislabeled because they are highly similar to *R*-genes from Solanaceae species (>70% nucleotide identity). For example, accession No. JN230646 was labeled as from *Luffa aegyptiaca*,but it has greater than 99% nucleotide identity with a gene from *Solanum lycopersicum* [such as Genbank accession No. AC238924]. To confirm that these sequences were mislabed, four pairs of PCR primers specific to some of the 11 potentially mislabeled sequences were designed. These primers could not amplify PCR products from any of the nine Cucurbitaceae species included in this study but could amplify PCR products from tomato cultivar Hongxiaoli (data not shown), confirming that the 11 sequences were mislabeled. Therefore, no new lineage of *R*-genes from Cucurbitaceae was found from GenBank or literature.

**Figure 5:**
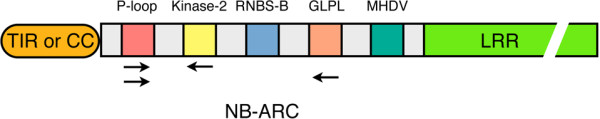
**The relative positions of domains of R proteins and degenerate PCR primers.** Five motifs from the NB-ARC domain are shown. Two pairs of degenerate primers are shown in arrows.

To search for new lineages of *R*-genes in Cucurbitaceae, 11 pairs of degenerate primers at the conserved motifs of *R*-genes were used to amplify *R*-gene fragments (240–700 bp) from the nine Cucurbitaceae species (Figure 5)*.* The PCR products were cloned into TA vector and 511 colonies were sequenced. A total of 196 distinct *R*-gene fragments were obtained from the nine species [GenBank accession No. KC107019 - KC107214]: 31 from *Cucumis melo*, 23 from *Cucurbita pepo*, 29 from *Cucurbita moschata*, 26 from *Lagenaria siceraria*, 26 from *Momordica charantia*, 10 from *Citrullus lanatus*, 15 from *Benincasa savi*, 20 from *Luffa cylindrica* and 16 from *Trichosanthes kirilowii*. The 196 newly amplified *R*-gene sequences were compared with all *R*-genes identified above. They are highly similar (>70% nucleotide identity and with e-value < 10^-40^) to at least one of the Cucurbitaceae *R*-genes identified previously. We conclude that there are few additional *R*-gene lineages, if any, present in Cucurbitaceae species besides those found in the sequenced genomes of cucumber, melon and watermelon.

### *R*-gene lineages lost in Cucurbitaceae

**Figure 6:**
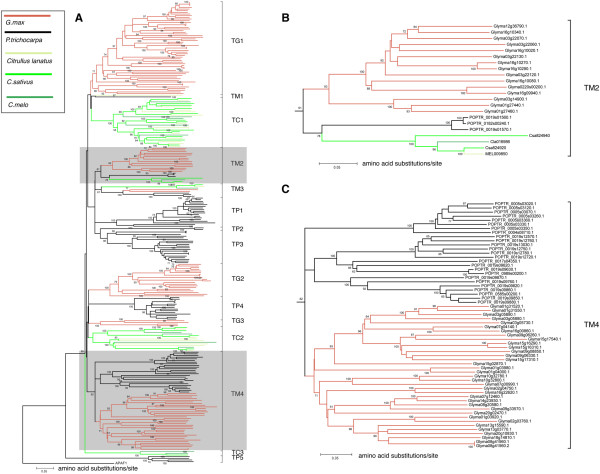
**Phylogenetic trees of NBS-LRR proteins from cucumber, melon, watermelon, soybean and poplar.** (**A**) trees for all TIR proteins. TG, clade specific to TIR proteins from *G.max*; TP, clades specific to TIR proteins from *P.trichocarpa*; TC, clades specific to TIR proteins from Cucurbitaceae; TM, clade with members from more than one plant family. The two shaded clades are enlarged in (**B**) and (**C**), respectively. (**B**) a clade with members from all three plant families; (**C**) a clade with members from soybean and polar but not Cucurbitaceae species.

Comparative genomics was used to investigate the genetic mechanisms underlying the scarcity of *R*-genes in Cucurbitaceae. The sequenced genomes of soybean and poplar have 450 and 552 *R*-genes, respectively. They were compared with the *R*-genes identified from the genomes of cucumber, melon and watermelon. According to the phylogenetic tree constructed using genome sequence data [Phytozome v8.0, http://www.phytozome.net/], Cucurbitaceae is more closely related with soybean (*G. max*, Fabaceae) than with poplar (*P. trichocarpa*, Salicaceae). To simplify the analysis, TIR and non-TIR subclasses were analyzed separately. Animo acid sequences between the P-loop and GLPL motifs were used to construct distance trees (Figure 6, Additional file 1: Figure S1). Consequently, a total of 39 clades (15 for TIR and 24 for non-TIR subclasses) were obtained for all R proteins from the three plant families. R proteins from Cucurbitaceae are present in 13 clades, which is very similar to the clades in Figure 1. The only changes are that clades T5 and T7 in Figure 5 are in one clade (TC2), and N7, N8 and N9 in Figure 5 are also in one clade (NC1). The clade numbers in soybean (22) and poplar (26) are considerably more than that in Cucurbitaceae (13). The remaining 16 clades (TM1-TM4 and NM1-NM12) contain sequences from two or all three plant families (Figure 6B). The high proportion (58.9%) of family-specific clades may suggest frequent loss of *R*-gene lineages in different plant species. Clades TM4, NM1, NM3, NM5, NM6, NM7, NM8 and NM9 are comprised of R proteins from soybean and poplar but none from Cucurbitaceae species (Figure 6C, Additional file 1: Figure S1). Since Fabaceae is more closely related with Cucurbitaceae than with Salicaceae, we can conclude that above eight lineages (i.e. clades) were lost in Cucurbitaceae after the divergence of Fabaceae and Cucurbitaceae. In summary, compared with soybean and poplar, Cucurbitaceae species lost more lineages of *R*-genes.

As a comparison, the same method was used to analyze the LRR-LRK proteins in the five genomes. A total of 189, 169, 184, 501 and 447 LRR-LRK proteins were identified from cucumber line 9930, melon, watermelon, soybean and poplar, respectively. The copy number of LRR-LRK encoding genes is significantly correlated with the number of NBS-LRR encoding genes in different species (*r* = 0.96). Such correlation was also discovered in other species [29]. The distance tree constructed using amino acid sequences in the conserved region of RLK domain showed 55 clades (data not shown). In striking contrast to R proteins, the LRR-RLK proteins have no clades specific to a single plant family. Furthermore, 54 of the 55 clades contained LRR-RLK proteins from all three plant families included in this study.

### Infrequent *R*-gene expansion in Cucurbitaceae

The number of *R*-gene subfamilies in cucumber line 9930, melon, watermelon, soybean and poplar is 11, 12, 12, 22 and 26, respectively. Their average of *R*-gene number per subfamily is 5.0, 4.2, 3.1, 15.6 and 15.4, respectively (Table 1, Additional file 1: Table S2). The number of *R*-genes per subfamily in cucumber line 9930, melon and watermelon is significantly lower than those in soybean and poplar (*p* <0.05, *t*-test). Three or fewer *R*-gene subfamilies in cucumber line 9930, melon and watermelon have more than 10 copies, while several *R*-gene subfamilies in soybean and poplar have more than 30 copies. The prevalence of small *R*-gene subfamilies in Cucurbitaceae species is consistent with previous observations that there have been very few duplications of *R*-genes after speciation of cucumber, melon and watermelon.

**Table 1:** Gene and gene family numbers of NBS-encoding genes and LRR-RLK genes in five species

	***Cucumis sativus***	***Cucumis melo***	***Citrullus lanatus***	***Glycine max***	***Populus trichocarpa***
*R-genes*					
Total numbers	70	75	55	450	552
TIR type	20	29	29	148	116
Non-TIR type	50	46	27	302	436
Subfamily number	11	12	12	22	26
Average per subfamily	5.0	4.2	3.1	15.6	15.4
*LRR-RLK* encoding genes					
Total numbers	189	169	184	501	447
Subfamily number	54	54	54	55	55
Average per subfamily	3.5	3.1	3.4	9.1	8.1

## Discussion

### Scarcity of *R*-genes in Cucurbitaceae

Compared with most sequenced plant genomes, the genomes of cucumber, melon and watermelon harbor relatively few *R*-genes. The scarcity of *R*-genes in Cucurbitaceae species is supported using degenerate primer approach. The degenerate primers were designed based on *R*-gene sequence from non-Cucurbitaceae species [41,42]. They were applied to eight cultivated and one wild species of Cucurbitaceae. The results from this work, and previous studies suggest that there are relatively few *R*-gene lineages in Cucurbitaceae [34], and the vast majority (if not all) of *R*-gene lineages in Cucurbitaceae are present in the three sequenced genomes.

The scarcity of *R*-genes in Cucurbitaceae was partially accounted for by the loss of *R*-gene lineages. In the distance tree for *R*-genes from Cucurbitaceae species, soybean and poplar, most clades are specific to a plant family. Many species-specific clades of *R*-genes were also observed in previous studies. To minimize the effects of rapid evolution of *R*-genes on our analysis, only the highly conserved motifs of the NBS region were used to construct the phylogenetic tree. Furthermore, clades were defined such that some clades contain *R*-genes from different plant families. The clades that contain *R*-genes from two or three plant families include the *ADR1* and the *NRG1* families [43,44]. The same threshold was used to classify RLK proteins, and all but one clade contain members from all three plant families, suggesting that the empirical definition of clade in this study is plausible. The prevalence of clades lacking members from all species indicates frequent loss of *R*-genes in plant species. Compared with soybean and poplar, Cucurbitaceae species lost more *R*-gene lineages. The loss of *R*-genes might be caused by various mechanisms such as unequal crossover, breakage followed by homologous repair, *etc.*[3]. Besides the loss of *R*-gene lineages, deficient duplications in extant *R*-gene lineages also accounted for the low copy number of *R*-genes in Cucurbitaceae. The average of gene number in a *R*-gene lineage in Cucurbitaceae is approximately 1/3 of that in soybean and poplar.

### Costs of *R*-genes in plants

High copy number of *R*-genes in plants should be advantageous because better resistance against pathogens is expected. However, the *R*-genes in a plant genome have not expanded infinitely. The limited number of *R*-genes in plant species suggests that *R*-genes must have biological cost to balance their expansion in a genome. The costs include not only energy for transcription and translation, but also their toxic effects. It is well known that high expression of *R*-genes may be lethal to plant cells. *R*-genes usually have low expression, controlled by complicated mechanisms [45,46]. Though each *R*-gene was kept at low expression level and its biological cost is limited [22], the cumulative effects of all *R*-genes in a genome might be decisive. Consequently, the number of *R*-genes in a genome, though maybe large, is kept at a certain range.

The total number of *R*-genes in different genomes (such as rice and cucumber) may vary more than 10 times. The number of *R*-genes in maize is also considerably lower than that in rice, though they are from the same family. Interestingly, the five sequenced genomes of Cucurbitaceae have consistently low copy number of *R*-genes. It seems unlikely that the low copy number is due to less challenge from pathogens since Cucurbitaceae species also face many devastating pathogens [47]. The mechanisms for large variations of R-gene number in different species will be an interesting topic for future studies. Unlike *R*-genes, the LRR-RLK encoding genes maintain most lineages in different species, though their copy number may vary considerably. Interestingly, the number of *R*-genes in a genome is significantly correlated with the number of LRR-RLK encoding genes [29]. It is most likely that similar mechanism may be involved in constraining the expansion of the LRR-RLK encoding family and the *R*-gene family.

### The P/A polymorphism for Type II *R*-genes and chimeric structure for Type I *R*-genes

A species needs a large number of *R*-genes to fight against various pathogens, which are usually diverse and evolve rapidly. As discussed above, a species can not increase the copy number of distinct *R*-genes through expanding the *R*-gene family in a genome. Alternatively, a plant species increases its *R*-gene number through unique population structures: different individuals in a species contain a different set of *R*-genes. First, many *R*-gene loci (Type II *R*-genes) show presence/absence polymorphism in different genotypes. Such population structure can contain a large number of distinct *R*-genes in a population/species, and at the same time maintain a low copy number of *R*-genes in each genome.

Compared with Type II *R*-genes, Type I *R*-genes have completely different patterns in evolutionary and population genetics. Type I *R*-genes are extensive chimeras and are highly diverse in different genotypes of a species [12,13,17]. Due to such population structure, a large number of *R*-genes with distinct sequences are predicted in a population/species, though their copy number in each genotype maintains at a low level. In rice, each cultivar or wild genotype contains a different set of Type II *R*-genes (due to P/A polymorphism) and Type I *R*-genes (different chimeric sequences), and consequently a tremendous number of distinct *R*-genes are present in the species [3].

The Cucurbitaceae species have not only low copy number but also low diversity of *R*-genes. There is limited number of *R*-gene lineages in Cucurbitaceae species, and therefore, P/A polymorphism of Type II *R*-genes contributed little to the *R*-gene diversity in Cucurbitaceae species. Furthermore, no sequence exchanges were detected between any *R*-genes in the eight cultivated Cucurbitaceae species included in this study, and it is very likely there is no Type I *R*-genes in the cultivated species of Cucurbitaceae. Interestingly, our primary study discovered a Type I *R*-gene lineage in the wild Cucurbitaceae species (*T. Kirilowii*) included in this study. It remains unknown if domestication in Cucurbitaceae played critical role in the low diversity of *R*-genes in cultivated species.

## Conclusions

Genome-wide analysis, data mining and PCR amplification all suggested that the Cucurbitaceae species harbor relatively few *R*-genes in a genome. Our analysis showed that the scarcity of *R*-genes in Cucurbitaceae species was due to frequent loss of *R*-gene lineages and deficient duplications in extant lineages. Most *R*-genes are highly conserved in different genotypes of cucumber, but in a wild Cucurbitaceae species, *T. kirilowii*, one lineage of *R*-genes exhibits chimeric structures.

## Methods

### Plant materials

Eight cultivated species of Cucurbitaceae: melon (*Cucumis melo*), watermelon (*Citrullus lanatus*), *Cucurbita pepo L*., pumpkin (*Cucurbita moschata*), bottlegourd (*Lagenaria siceraria*), balsam pear (*Momordica charantia*), fatmelon (*Benincasa savi*) and luffa (*Luffa cylindrica*) and seven genotypes of *Trichosanthes kirilowii*, a wild species of Cucurbitaceae, were included in this study for *R*-gene analysis. The seven wild genotypes were collected from different natural populations in central China (Table 2). DNA was extracted using a modified CTAB procedure as described previously [48].

**Table 2:** Plant materials used in this study

**Species name**	**Genotype name**	**Origin**
*Cucumis melo*		Hubei, China
*Citrullus lanatus*		Hubei, China
*Cucurbita pepo*		Hubei, China
*Cucurbita moschata*		Hubei, China
*Lagenaria siceraria*		Hubei, China
*Momordica charantia*		Hubei, China
*Benincasa savi*		Hubei, China
*Luffa cylindrica*		Hubei, China
*Trichosanthes kirilowii*	DZ-1	Henan, China
*T. kirilowii*	NX-1	Henan, China
*T. kirilowii*	NX-2	Henan, China
*T. kirilowii*	HD	Hebei, China
*T. kirilowii*	XY	Henan, China
*T. kirilowii*	SD	ShanDong, China
*T. kirilowii*	JS	JiangSu, China

### Amplification of *R*-gene fragments

Eleven pairs of degenerate primers were designed from the conserved regions of *R*-genes [41,42] (Table 3). These primers were used to amplify *R*-gene fragments from different Cucurbitaceae species in a 50 μl PCR reaction, containing 2 μM of degenerate primers, 2× PCR mix buffer (NovoGene, Wuhan, China), and 50 ng genomic DNA. Genomic DNA was denatured for 4 min at 95°C followed by 30 amplification cycles of 95°C for 45 s, 38-45°C for 45 s, and 72°C for 1 min (Additional file 1: Table S3). PCR products were gel purified and cloned into the p-EASY-T5 vector (TransGen Biotech, Beijing). Individual colonies were sequenced.

**Table 3:** **Degenerate primers used to amplify *R*-gene fragments**

**Primer name**^**a**^	**Primer position (motif, domain)**	**Sequence 5’→3’**^**b**^
15912	GGVGKTT, Kinase-1a	GGT GGG GTT GGG AAG ACA ACG
15914	GGVGKTT, Kinase-1a	GGI GGI GTI GGI AAI ACI AC
16410	GGLGKTT, Kinase-1a	GGI GGI YTI GGI AAR ACI AC
16403	GGMGKTT, Kinase-1a	GGI GGI ATI GGI AAA ACI AC
16409	GGSGKTT, Kinase-1a	GGI GGI WSI GGI AAR ACI AC
PLP	GG(G/O/M)GKTT, Kinase-1a	GGI GGI RTI GGI AAR ACI AC
310	VLDDVW, Kinase-2	CCA IAC RTC RTC NAR NAC
antiK2	VLDDVW, Kinase-2	CCA NAC RTC RTC IAR IAC
antiHD1	CKGLPL, HD	ARN GGI ARI CCY TTR CA
17609	FLDIACF, NBS-VI	RAA RCA IGC SAT RTC IAR RAA
28107	FLHIACF, NBS-IX	RAA RCA IGC DAT RTG IAR RAA

^a^ All degenerate primers used in this study were from references [41,42].

^b^Keys for degenerate positions: I, inosine; R,A/R; Y, C/T; K, G/T; N,A/G/C/T; A/G/T; W, A/C; S, G/C.

If an obtained sequence has significant similarity with an *R*-gene (e-value < e^-10^) or encodes expected R protein motifs, it is considered as *R*-gene fragments. *R*-gene sequences that were derived from the same PCR reaction and had 99.5% nucleotide identity were considered to be from the same gene.

All *R*-genes identified from the sequenced genomes of cucumber, melon and watermelon were classified into subfamilies based on threshold of nucleotide identity of 70%. PCR primers specific to each subfamily were designed and used to amplify corresponding genes from seven genotypes of *T. Kirilowii* (Additional file 1: Table S4). PCR products were sequenced directly. If sequencing results suggest more than one sequence in the PCR products, they were cloned into TA vector (TransGen Biotech, Beijing, p-EASY-T5 vector) and individual colonies were sequenced. *R*-gene sequences that were derived from the same PCR reaction and had 99.5% nucleotide identity were considered to be from the same gene.

### Sequence analysis

Genome sequences of cucumber line 9930 (v2.0) and watermelon (v1.0) were obtained from Cucurbit Genomics Database [http://www.icugi.org/]; melon genome sequences (v3.5) were from Melonomics [http://melonomics.net/]; poplar (v2.2) and soybean (v1.0) genome sequences were retrieved from Phytozome [http://www.phytozome.net/]; genome sequences of the North American pickling cucumber inbred line ‘Gy14’ and the North-European Borszczagowski cultivar (line B10) were downloaded from [http://www.phytozome.net/] and [http://csgenome.sggw.pl/], respectively. *R*-genes and LRR-RLK encoding genes were identified using hidden Markov models (HMM) and BLASTN. First, NB-ARC (Pfam: PF00931) and Pkinase (Pfam: PF00069) were used to search for NBS and RLK proteins in the five genomes using HMMER [49], and the results were parsed using a Perl script. Then, LRR (Pfam: PF00560, PF12799, PF13516, PF13855) were used in the parsed RLK homologues to identify the LRR-RLK proteins. A database containing 5,158 protein sequences with NB-ARC domain, 3,110 protein sequences with ATP binding domain and 6,979 protein sequences with LRR domain from NCBI was used to verify the potential NBS-LRR or LRR-LRK encoding genes [3]. All identified sequences were annotated using FGENESH [http://www.softberry.com] and redundant sequences were removed. TIR (Pfam: PF13676) and non-TIR protein were distinguished using HMMER. The verified *R*-genes were used to identify partial or divergent homologues using BLASTN.

Sequences were aligned using MUSCLE [50] and manually edited in Geneious [40]. Nucleotide identity was calculated using Geneious. Neighbor-Joining (NJ) trees using Kimura’s two-parameter model (for DNA sequences) and p-distance (for amino-acid sequences) were constructed and bootstrap values (100 replications) were caculated using MEGA 5.0 [51]. For amino acid sequences, only the highly conserved regions sequences between the P-loop and GLPL motifs were used for phylogenetic analysis [35,52]. Sequence exchanges were identified using Geneconv with no mismatch allowed [53].

### Colinearity and presence/absence (P/A) polymorphism of *R*-genes in cucumber, melon and watermelon

To compare *R*-genes in cucumber, melon and watermelon, orthologous pairs of *R*-genes were determined first. For an *R*-gene in cucumber line 9930 and an *R*-gene in melon/watermelon to be orthologues, the two genes must be mutually best hits in bi-directional BLASTN search [35]. To exclude false-positive results, only two genes with high scoring pair (HSP) of more than 500 bp and average nucleotide identity of greater than 80% were considered orthologous. If a pair of orthologues are not located in syntenic regions in two genomes, one of the them is considered to have been translocated.

To investigate the presence/absence (P/A) polymorphism of *R*-locus, the syntenic region of *R*-genes was used to compare. The definition of syntenic region follows [54]. First, 20 genes, 10 from each side of an *R*-gene in cucumber inbred line 9930, were used in BLASTN search of the melon (watermelon) genomes. If the best hits of at least 14 genes in melon (watermelon) are present in a 20-gene window, this region in melon (watermelon) is considered syntenic to the *R*-gene region in cucumber line 9930. If an *R*-gene is present in cucumber line 9930 but absent in its syntenic region in melon (watermelon), the *R*-gene locus is considered to have P/A polymorphism. Two *R*-genes separated by no more than 8 non-*R*-genes were considered to be clustered [55]. Each *R*-gene cluster is considered as a multiple-copy *R*-locus, and non-clustering *R*-gene is referred to as single-copy *R*-locus [3].

## Competing interests

The authors declare that they have no competing interests.

## Authors’ contributions

XL carried out the molecular genetic studies, participated in analysis and interpretation of the data and drafted the manuscript. JC was involved in sequence analysis. YZ carried out whole genome bioinformation analyses and wrote a series of Perl scripts to identify the NBS-LRR-encoding genes. HK and JC designed the study and revised the manuscript. All authors read and approved the final manuscript.

## Supplementary Material

Additional file 1 **Table S1.***R*-gene numbers in different sequenced plant genomes. **Table S2.** Average *R*-gene numbers per clade. **Table S3.** RGAs amplified using degenerate primers. **Table S4.** PCR primers used to amplify *R*-gene fragments from *Trichosanthes kirilowii. * Click here for file

Additional file 2 **Re-annotation of *R*-genes in cucumber Gy14.** Click here for file

Additional file 3 **Re-annotation of *R*-genes in melon.** Click here for file

Additional file 4 Gene structure model in Cucurbitaceae. Click here for file

Additional file 5 Annotation summary for R-genes from melon cucumber Gy14. Click here for file
