# Supplementary material for: Frequent loss of lineages and deficient duplications accounted for low copy number of disease resistance genes in Cucurbitaceae
Source: BMC Genomics. 2013 May 17;14:335. doi: 10.1186/1471-2164-14-335 (PMC3679737; doi:10.1186/1471-2164-14-335)

Table. S1 *R*-gene numbers in different sequenced plant genomes

|  | **NBS-encoding gene numbers** | **Genome size (MB)** | **predicted coding gene numbers** |
| --- | --- | --- | --- |
| *Populus trichocarpa* | 552 | 550 | 45,555 |
| *Oryza sativa* | 508 | 390 | 37,544 |
| *Glycine max* | 450 | 1,115 | 46,430 |
| *Sorghum bicolor* | 245 | 730 | 35,899 |
| *Brachypodium distachyon* | 239 | 350 | 32,255 |
| *Arabidopsis* | 149 | 157 | 25,498 |
| *Zea mays* | 129 | 2,300 | 32,504 |
| *Cucumis melo* | 75 | 375 | 27,427 |
| *Cucumis sativus* | 70 | 367 | 26,682 |
| *Citrullus lanatus* | 56 | 425 | 23,440 |
| *Carica papaya* | 55 | 372 | 28,629 |
| TTEST |  | 0.0486 | 0.0000000706 |

Table. S2 Average *R*-gene numbers per clade

|  | **Cucumber**  **(11)** | **Melon**  **(12)** | **Watermelon**  **(12)** | **Soybean**  **(22)** | **Poplar**  **(26)** |
| --- | --- | --- | --- | --- | --- |
| *R*-genes  per clade | 12 | 5 | 1 | 55 | 1 |
|  | 2 | 1 | 13 | 15 | 3 |
|  | 1 | 1 | 1 | 2 | 3 |
|  | 4 | 4 | 1 | 20 | 18 |
|  | 1 | 2 | 7 | 4 | 2 |
|  | 12 | 5 | 6 | 35 | 20 |
|  | 5 | 8 | 5 | 57 | 14 |
|  | 4 | 4 | 2 | 12 | 25 |
|  | 8 | 3 | 5 | 2 | 4 |
|  | 1 | 3 | 2 | 9 | 20 |
|  | 2 | 1 | 1 | 15 | 22 |
|  |  | 1 | 2 | 3 | 32 |
|  |  |  |  | 2 | 3 |
|  |  |  |  | 47 | 23 |
|  |  |  |  | 4 | 9 |
|  |  |  |  | 1 | 10 |
|  |  |  |  | 4 | 3 |
|  |  |  |  | 14 | 3 |
|  |  |  |  | 9 | 24 |
|  |  |  |  | 14 | 77 |
|  |  |  |  | 4 | 3 |
|  |  |  |  | 16 | 11 |
|  |  |  |  |  | 40 |
|  |  |  |  |  | 25 |
|  |  |  |  |  | 2 |
|  |  |  |  |  | 4 |
|  |  |  |  |  |  |
|  |  |  |  |  |  |
| TTEST |  | Melon | Watermelon | Soybean | Poplar |
| Cucumber |  | 0.445 | 0.954 | 0.055 | 0.032 |
| Melon |  |  | 0.407 | 0.027 | 0.015 |
| Watermelon |  |  |  | 0.047 | 0.026 |
| Soybean |  |  |  |  | 0.639 |

Table. S3 RGAs amplified using degenerate primers.

| **Species** | **Primer combinations** | **Length** | **Number of new RGAs** | **sequenced TA clones** | **annealing temperature** |
| --- | --- | --- | --- | --- | --- |
| *Cucumis melo* | 2：16410＋310 | 250bp | 13 | 20 | 43℃ |
|  | 7：16410＋17696 | 500bp | 7 | 20 | 43℃ |
|  | 13：16403＋310 | 245bp | 3 | 10 | 38℃ |
|  | 14：PLP+antiK2 | 245bp | 8 | 15 | 42℃ |
| *Cucurbita pepo L.* | 2：16410＋310 | 245bp | 5 | 20 | 43℃ |
|  | 4：16409＋310 | 245bp | 5 | 10 | 40℃ |
|  | 7：16410＋17696 | 245bp | 6 | 10 | 43℃ |
|  | 14：PLP+antiK2 | 245bp | 5 | 15 | 42℃ |
|  | 15：15914＋310 | 245bp | 2 | 10 | 42℃ |
| *Lagenaria siceraria* | 2：16410＋310 | 245bp | 7 | 10 | 43℃ |
|  | 4：16409＋310 | 245bp | 5 | 10 | 40℃ |
|  | 5: 16403 + 28107 | 700bp | 5 | 10 | 40℃ |
|  | 7：16410＋17696 | 500bp | 2 | 10 | 43℃ |
|  | 14：PLP+antiK2 | 245bp | 7 | 24 | 42℃ |
| *Cucurbita moschata* | 2：16410＋310 | 280bp | 11 | 20 | 43℃ |
|  | 4：16409＋310 | 269bp | 5 | 20 | 40℃ |
|  | 13：16403＋310 | 245bp | 4 | 14 | 38℃ |
|  | 14：PLP+antiK2 | 245bp | 4 | 15 | 42℃ |
|  | 15：15914＋310 | 245bp | 2 | 10 | 42℃ |
|  | 17：15912＋310 | 245bp | 3 | 10 | 48℃ |
| *Momordica charantia* | 2：16410＋310 | 245bp | 4 | 18 | 43℃ |
|  | 4：16409＋310 | 245bp | 9 | 10 | 40℃ |
|  | 14：PLP+antiK2 | 245bp | 9 | 15 | 42℃ |
|  | 17：15912＋310 | 245bp | 4 | 10 | 48℃ |
| *Citrullus lanatus* | 2：16410＋310 | 245bp | 2 | 10 | 43℃ |
|  | 14：PLP+antiK2 | 245bp | 5 | 12 | 42℃ |
|  | 15：15914＋310 | 245bp | 3 | 12 | 42℃ |
| *Benincasa Savi* | 2：16410＋310 | 245bp | 6 | 10 | 43℃ |
|  | 4：16409＋310 | 245bp | 1 | 10 | 40℃ |
|  | 14：PLP+antiK2 | 245bp | 5 | 12 | 42℃ |
|  | 15：15914＋310 | 245bp | 3 | 12 | 42℃ |
| *Luffa cylindrica* | 2：16410＋310 | 245bp | 4 | 12 | 43℃ |
|  | 4：16409＋310 | 245bp | 4 | 10 | 40℃ |
|  | 14：PLP+antiK2 | 245bp | 6 | 10 | 42℃ |
|  | 15：15914＋310 | 245bp | 6 | 10 | 42℃ |
| *Trichosanthes* *kirilowii* | 2：16410＋310 | 245bp | 8 | 15 | 43℃ |
|  | 4：16409＋310 | 245bp | 1 | 10 | 40℃ |
|  | 5: 16403 + 28107 | 700bp | 1 | 10 | 40℃ |
|  | 6： 16403 + 17696 | 700bp | 3 | 10 | 40℃ |
|  | 7：16410＋17696 | 700bp | 3 | 10 | 43℃ |
| **Total** |  |  | 196 | 511 |  |

Table. S4 PCR primers used to amplify *R*-gene fragments from *Trichosanthes kirilowii*

| Primer combination | Primer | Sequence(5’ → 3’) |
| --- | --- | --- |
| GL-164 | 2698-F1 | TGGCCGGAAATCTGCTCGGAA |
|  | 2698-R1 | TCCAAGAGTGTGCTCCTCAACA |
| GL-179 | 10121-F1 | TGGCGCTGGAATTGGTGGGT |
|  | 10121-R1 | CGAGAGGATAGTTCGTCAAGGGT |
| GL-202 | 9608-F1 | AGGCTCTTCAGCTCTTTTGCC |
|  | 9608-R1 | AGCAGCCACCCAATGCGAGA |
| GL-43 | CG1-2-F | GTGGAAGCCATTCTTCGTGATGT |
|  | CG1-3-R2 | TGTGTGGTTGAAGCCCTTCTAAC |
| GL-47 | CG1-2-F | GTGGAAGCCATTCTTCGTGATGT |
|  | CG1-2-R2 | TTTTTGTGTGGCTGAAGTCCTTC |
| GL-49 | CG2-F2 | GCGGGTATAGGTAAGAGCACA |
|  | CG2-R2 | TTGGAAGCTCTCTGATTGTGAA |
| GL-6 | CG1-6-F | GTGCTTGACGATGTTTGGAATGAAA |
|  | CG1-6-R | CGAAACAATAGAGTCTGGAAGC |
| GL-101 | 8307-F | CTATATGGGTATGTGTGTCAG |
|  | 8307-R2 | TGAAGTTGTTGAGGAAGTTGTGTT |
| GL-221 | 51-F1 | GCAATGTGGGGGTCTTGCTC |
|  | 51-R1 | TGGCTTTAACTGCTCTTGCTTCCT |
| GL-240 | 101-A3-F3 | AAAGTTTAACGGATAAAGTCCCA |
|  | 101-A3-R3 | TCTGGCAACTGTTTCAACCC |
| GL-251 | 9413-F2 | TTTCTTTGTTGGAGAGATAGCCAC |
|  | 9413-R6 | TACATCTACCGCCCACTCGTC |
| GL-61 | 8307-F | CTATATGGGTATGTGTGTCAG |
|  | CG1-5-R | TTGTTGTGTGGTTGAAGTCCTTC |
| GL-77 | CG3-F | AGGTATGGGCAAGACAACTTTGGC |
|  | 6768-R1 | GCTGAACCATTCTGGAATCTCA |
| GL-93 | 6803-F1 | AATGGGTGATTTCCTATGGACTT |
|  | 6803-R2 | TTGGAAGGTGGGAAAGTTTTG |

Figure S1


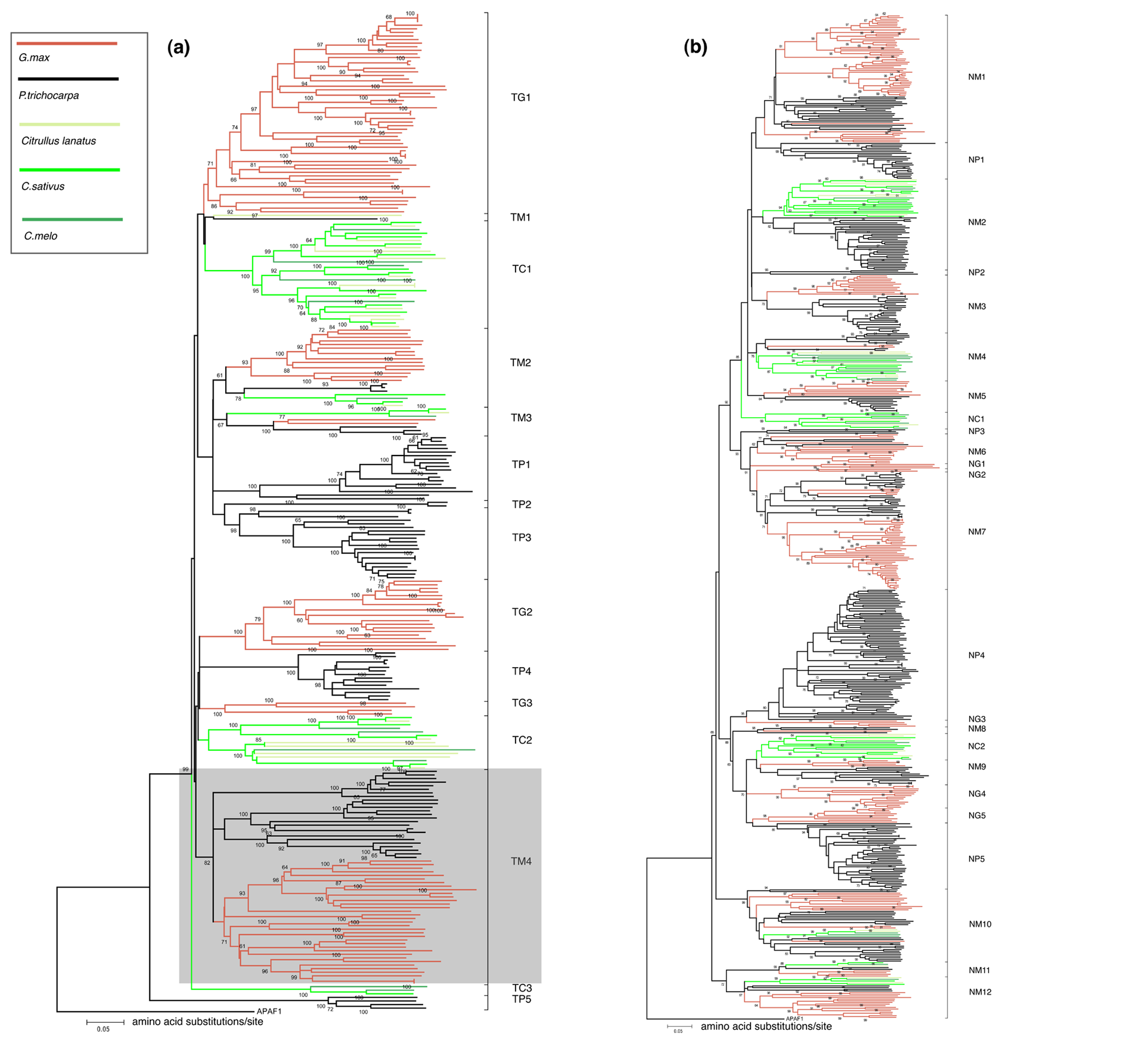

Supplement: Additional file 1 — Table S1.R-gene numbers in different sequenced plant genomes. Table S2. Average R-gene numbers per clade. Table S3. RGAs amplified using degenerate primers. Table S4. PCR primers used to amplify R-gene fragments from Trichosanthes kirilowii. [file 1471-2164-14-335-S1.docx]
