# Supplementary material for: Frequent loss of lineages and deficient duplications accounted for low copy number of disease resistance genes in Cucurbitaceae
Source: BMC Genomics. 2013 May 17;14:335. doi: 10.1186/1471-2164-14-335 (PMC3679737; doi:10.1186/1471-2164-14-335)
Supplement: Additional file 2 — Re-annotation of R-genes in cucumber Gy14. [file 1471-2164-14-335-S2.docx]

**Additional file 2:**

**Re-annotation of *R*-genes in cucumber Gy14 (Introns were marked in red)**

>Cucsa.089350

ATGTCTGATAAAAAAATTGTTATGCTTGGATTATATGGAATTGGAGGTATCGGCAAGACAACTTTGGCCAAAGCATTGTACAATAGAATTGCTCATGACTTTGAAGGTTGTTGCTTTTTGGAAAAAATTAGAGAAGCTTCAAATCAATATGACGGCCTTGTTCAACTCCAAAAGAAAATACTTTGTGATATTCTAATGGATAATTCGATCAATGTTAGCAATCTTGATATAGGGGTTAACATCATAAGGAATCGACTATGCTCAAAAAAAATTCTTTTAATTCTTGATGATGTTGATACGAGAGAACAACTAGAAGCATTAGCGGGAGGGCATGATTGGTTTGGACATGGAAGTAAGATCATTGCGACAACAAGAAACATGCAATTACTTGCTAGTCATGGATTTAATAAATTGGAAAAAGTTAACGGATTGAATGCCATTGAAGGTCTTGAGCTTTTTAGTTGGCATGCATTCAACAATTGTCATCCCTCAAGTGATTATTTAGACCTTTCAAAACGTGCTGTACATTATTGTAAAGATCTTCCATTAGCTCTTGAAGTGTTAGGTTCCTTCCTTAATTCTATTCACGATCAATCCAAATTTGAACGTATATTGGACGAGTATAAGAACTTCTATCTAGACAAAGACATCCAAGATATTCTTCGAATAAGTTATGATGAACTTGAACAAGATGTAAAAGATATTTTCCTTTACATTTCTTGTTGCTTTGTAGGAGAAGATATCAACGAAGTTAAAATGAAGTTAGAAGCATGTGGTTGTTTATGTTTGGAAAAGGGAACAACAAAACTAATGAATTTATCACTTCTAACCATTGAATCCAATCGAATTAAAATGCATGACTTAATACAACAAATGGGTCGCTCAATTCATCTCTCGAAAACTTTTACATCTCATAAAAGAAAAAGATTGTTGATTAAAGATGATGCTATGGAT

GTCTTAAATGGGAATAAGGTAAGAACTATCCAGCAAAACTAATATTTTTTATATGCTTATATTTATTATGAGAACTTTTATTATTTTGAAGGGTTTGTTTTGATCATTTGTAG

GAAGCAAGAGCAGTTAAAGTTATAAAATTGGATTTTCCTCGACCTACCCAACTTGACATTGATTCAAGAGCTTTTGAAAAAGTGAAAAATTTGGTAGTACTCGATGTTCGCAATGTCACATCTTCAAAAGGTACTGATCTTGAGTATCTACCTAGTAGCATAAGGTGGATGAATTGGCCTCAATTTCCTTTTTCATATTTGCATACAAGCTTCACAATAGAGAACCTTGTCAAGTTCAACTTGCCATATAGCTCCATAAAAAAATTTGGGAAAGCATTAATGGTATTAATCTTTGTCAATAATATTAATTTAATTTCAATGTTGGCTTATTTATTTGAAATTTAATTATATATTTTTTATATTAATTGCAGTGTGGTGAATGGTTGAAGGAAATTAATCTTAGTTACTCTAA

GTTTTTGGTGGAAATTCCAGATTTAACTACTGCAATAAACCTCGAAAAGTTGAATCTTGAAGGGTGTGAAAAATTAGTAAAGGTTCATGAATCAGTTGGATCTCTCAGTAAGCTTGTTGAGTTTTATCTTTCCAGCAGTGTTGAGGGTTTTGAGAAGTTTCCATCCTGCCTTAAGTTGAATTCTCTTGAAGCTTTGGTAGTGAGATATTGTAGAATAGAAGAATGTTGTCCTCAATTTAGTGAAGAAATGAATAGCCTAGAAATATTGGAGATCGATGATAGTATAATTAATCAATTATCTCCAACAATTGAATATCTTACTGGCCTAAAAGAACTCTGGATCACAGAGTGCACGAAGCTCGAAACTCTTCCAAGTACAATTTATCGTTTAAGTAATCTTACTTCTTTAGAAGTCAAAAAATCTGATCTTTCAATCTTTCCTTCCTTAAATGATCCTTCTTCATCTTCCTTATTAATTCCCTACCTAACATCAATAAAGCTTTTCAATTGTCAG

ATAACAAATTTGGATTTCTTAGAAACAATGGTTCACGTTACCCCTTCATTGGAAATGTTGGACTTATCTCAAAACAACTTTTGTGGACTACCCTCCTGTATTATTAATTTTAAATCCTTGAAATATCTTTATATAATCGAATGTAAGTCGCTTGAAGAAATTCTAAAGGTTCCAAAAGGAGTAGTTCGTATGGATACTAGAGGGTGTGTATCATTGGCCAAATTTCCTAACAACATTCCTGATTTCATATCTTGTGATGATAATGTG

GTACGTATCATTTCTTTTTCATGACTTCATCTCTTATGTATTTCATTCATATAAGAATTAATTAATTTTTCGTGCATTGTTAATTAATATAACTTTTAGCTATTTACTTTCATTGTGCAG

GAATATGATACAAAAGACGGAGTAATCAAACAACTCATATTAATGAATTGTGATATTCCAGATTGGTGCAAGTACAAGAGTATGAACAATTCAGTAACGTTTGATTTTCTAGCTGATTATTTAAGTTGGAAAAGGAAGGCTTTTATTGCTCTTTGTGTCAAATTTCATGTTACCAATGATCACGAGTTGGTTAAGCTTAATTGTGGAGTACTGTTTATCAACGATATTGAAGTATGGAGTCGAATGTCAATCTCCAATTTTAACTTTTGGCTATCACGAGGTGAATGTCTATGGATGGCAGTACTTCATCCTTGCATGCATCGTCTAATCAACCCATATGGTGATGATATTATGGACATCTCACCGAATTTCTCAATAGGCATTTTGGACAATAAAATTACATTGTTATTTGAGGTTAATCCAGAGTGTAAAGACACA

>Cucsa.091460

ATGGGTTCTTCTGTTGTTGGAGATGAATCATTTTCTTCTTCTCCCAATTTCAATTACGATTATGATGTGTTTTTTAGTTTCAGAGGAGAAGATACTCGCTCCAATTTTATCAGTCATCTTCATATGGCCTTGCGTCTAAAGGAAGTCAACGTTTTCATAGACGACAAACTCAAAAGGGGTGAACAAATTTATGAGTCTCTTCTCAAATTTATAGAGCGATCTAGACTTTCCCTCGTTATTTTCTCTAAAGATTATGCATCTTCAACTTGGTGTTTGGATGAACTGGTGAAAATAATTGAGTGTAAGAAATCCAAAGGACAAGCAGTTTGGCCAGTGTTCTACAAGGTGGATCCATCCGAGGTTCGAAAACAAACCGGTGGGTTTGGGGAAGCATTGGCCAAACATGAAGCTAATAAGTTATTGGCCAACAAGATTCAACCATGGAGGGAAGCTTTGACTTTTGCTGCTGGTTTGTCTGGTTGGGATCTAGCAAATAG

GTATTTCTTTTTTCTTTTTTTTGTTTTAATCTTTGAAGACTCATTGTCCAAGTGAAGTTTAAATTTATCTACTATTTCCAAATTCTGATTCTTTTTGTATGATGTTACTTTACAACAG

CAAGGATGAGGCTGAACTTATCCAAAAAATTGTTAAACGAGTGTTGTCTGCAGTAAATCCAATGCAATTACTACATGTAGCCAAGCACCAAGTAGGAGTTGATTCTCGACTAAGGAAAATTGAGGAGTTGGTCTCTCATATTGGGTCCGAGGGTGTTAATCTGGTGGGGTTGTATGGCATTGGAGGCATTGGTAAGACCACTTTGGCTAAGGCTTTGTACAACAAAATTGCTACCCAATTTGAAGGATGCTGCTTTCTACAAGATGTTAGACGAGAAGCTTCGAAGCATGGGCTCGTTCAACTACAGGAAACCTTACTCAATGAGATCTTAAAAGAGGATTTGAAGGTTATTGTCAGTCGTGATAGAGGAATTAACATCATAAGAAGTAGACTGTGTTCAAAGAAAGTTCTTATAGTTCTTGATGATGTGAATGATCTTGAGCAATTAGAAGCACTGGTTGGTGGGCGTGATTGGTTTGGTCAAGGTAGTAAAATCATTGTGACGACAAGGAATGAACATTTACTTTCTAGCCATGGATTTGATGAAAAGCATAAAATTCAAGAATTGAATCAAGACCATGCTCTTGAGCTTTTCAGTTGGCATGCTTTTAAGAAAAGTCATCCATCAAGTAATTATTTAGATTTTTCAAAGCGTGCTACAAGTTATTGTAAAGGTCTCTCTTTGGCCCTCGTTGTTTTGGGTTCTTTCCTTTGCGGCAGAGCTAAAGAAGAATGGAATGGTATACTAGATGAATTTGAAAACTCTTTGAGAAAAGATATTAAAGATGTACTTCAATTAAGTTTTGATGGACTTGAAGACAAAATAAAGGATATTTTCCTCGACATTTCTTGTTTATTCGTGGGAGAAGAATACAAGTGTGCTAAAAAAATGTTGAGTGCATGTCATTTGAACATAGATTTTGGAATTATGATACTCATGGATCTTTCACTTATTACGGTTGAAATGGATAGAGTGCAAATGCATGAGTTAATACAACAAATGGGTCGTAGCATAGTTCATAATGAATCATCTGAGCCTGGAAAGAGGAGTAGGCTGTGGTTGGAACACGACATTTGGGAG

GTGTTTGTTAATAATTCTGTGAGTAACTCTCTTACCTAAAGTATTGATAATTTACTTATTTTCAAGGCTTCACATGATAAGAAATGTTTGTCAAAAGTAAGTTGTAAGCAGTGTTTACTAAATACATGACTTTATGCAG

GGAACAGATGCAGTTAAAGCCATAAAGTTGGACTTGCCTAAATCCACAAGGCTAAATGTAGATCCACGGGCATTTGGAAGCATGAAAAATTTGAGATTGCTTATCATTCGAAATGCACGATTTTGTACAAAGATTAGGTACTTACCTAATAGCTTAAAGTGGATTGAATGGCATGGATTTGCTCATCGAACTTTGCCGTCCTGCTTCATTACCAAAAATCTTGTTGGACTTGATTTGCAACATAGCCTCATCAAAAGATTTGGGAAAAGACTTAAG

GTATGAAAATGAGGTTTAGTTTCTTCATTGTTTTTCTTAAAAACTTTTAATAGGCAGCTTAATTACATTTTTAAAAAAATCACAAATACATGGATTAGCAAAAAATTATCATCGATTAACTCTTGCTAGTCATATATTTTTTAAGAATTACAAAATATATCTATTAACGATAGATTCATCTATCGCACAACAATAGCTCCTACTAGTCATATAATCTATCATTAATAAACTCATATAAGTGATATGATCTACCATGGATAAACTCTAAGAACATCCGCTAATTTTTTTCTAAATTTGTAATTTTTTGTGTTATCCTTTTGAGTATGATAACCATCATGATGCAAAATAATGTATTGGGTAGATTCTTTTTATGTTATGTAACTTACTTGAACTAGATTTGTTGCATACAAGATTGTCCAATTATAATGTGTCCTTAAGTTCCAAAATTTTGATTTTGTTACGTTTTTATCTTTTTTTTAAAAAAAATTAAATTTGATTTAAGATCAAATCAAACTAAAATTTGAAACCAAATTTCCACTATAAATTGACTAAATTACAATTTTGTTGGTAATTAGTTTACAATCTGCTAAATTCAAAATAATATATTTGATGTAAAATTACCTTTTTAACATCTGAGTTTTCATGAATACGTGCCATTGGTCCATAAGTTTTAAAAATAGACATTTTTAATTTCAGATTTATAATAATAGAATAATTTGATCCTAAGTTTTTAAAAATAGGTTTAAAAGACCTTCAAATAATTTTATGCTTTTATTTTAAATAGTTATATGACATTGTAAATTAGAATAATATATGGCTTCTAAAATTCATATCCTCTCTAAAACTTTTTTTTTCTAGTTTTAAATATCATATAATTATTTTTAAAAAACCATAAAGTACTTTTAGGACGTTATAAACCTATTATTTCTTAAAACTCAAGTGAGTAAGAAGATACATTTTTAAAATTTAAGGACCAAATATGATTATTTTTATAAACTTGGATACTAAAATTCATATCATCTCTAAAACTAACAGTTGTAAAAGTGTGTCGTGGCTAACATGAACGTATAGCTCAACTGACATAGTTTTTGTACTATCAATTTTGATATTGGAGGTTTGATTCTCTCATGCTTTAATTACAATACCTTTTAAAAAGAAAGAAAAGTGTTGTTATTTTATGGGGCATTTGTAAAAATAGCAAAAACTTTTATGATAATGGGGTTTATGTCACTATATTTTCTAAATTACAAAAGTAACAAATTTAAAAGCAAATAGTCCTCTGATAGCTGTATGATATATTTAATTAATTGTCATATTTGTCAAAAAGAGGTGCTATGAAATGCTTTTTTTCTAATTTTTTTTGTCATCTAATGCAATTTTCCTTATTATATATGCAATATAATAGTTGTGGATTAATGCAAATAGATGATTAATCTTGATATGCAAATCGATTTACTTTTGTATATGGAGTTTTATTAGTTTGCCTAAATATTAATTAATGGTTATTATAGATTTTTTTTAAGGAAAATTTACATAAGCAGAAGAAAGTGGAAAGTATTTACATCCTATAGACAAAAAATGGATATTTTAGACTTGATTAAAATTTTGGTTATTCATTTTGAGCTTGGATTAAAATTTTGGACTGTAAATAGTTTGGTAATTCTTCTATTTTAAAAATTTTCCTTTTTTTAACATATATTCTATATATTTTTCTATATTTACAATTATTGTTTTAGCCAATATTTTATCTAAATTTTGTTTGGTATGCCAACTACATACCCATTTTCCTTTTGGTTTATTTTCCAATGTGTTTTAG

GATTGTGAAAGGTTGAAGTATGTTGATCTTAGCTACTCTACTTTATTAGAGCAAATTCCTGATTTCACTGCAGCATCAAACCTTGAAGAGTTGTATCTCATCAATTGCACAAATTTAGGAATGATAGATAAGTCTATTTTCTCTCTCAATAAGCTTGTTGTCCTAAACTTTGATGGTTGTTCTAACCTTAAAAAGCTTCCAAGTGGCTACTTCATGTTAAGTTCTCTTAAAGAATTGAATCTCTCTTACTGCAAAAAACTTGAGAAAATTCCAGACTTATCTTCAGCATCAAACCTTACGAGCTTGCATCTCTACGAATGCACAAATTTAAGAGTGATTCATGAATCTGTTGGATCTTTGGATAAGCTTGACCATTTGAACCTTAGACAATGCACGAACCTGGTAAAGCTTCCAAGCTATCTTAGGTTAAAGTCTCTTCGATATTTATCACTTTCTGGGTGTTGTAAGCTTGAAAGCTTCCCAACAATTGCTGAAAGCATGAAATCTTTAAGGTCCTTGGATTTGGATTTTACTGCCATAAAGGAGTTACCTTCATCAATGGGATATCTTACCAATCTATCTCGATTAAACCTTGACAGTTGCACAGGCCTCATCTCCCTTCCCAATACAATTTCTTTGTTAATGTCCCTATTGAAACTTGACTTAAGAAATTGCAGGTCTCTTCAAGAAATTCCGAACCTTCCTCAAAACATACAAATTTTGAATGCGAATGGCTGTAAATTGGTGGGTAAAAGTCCAGATAACATTGGGGATATAATATCAAAAAAACAG

GTTCTGTTTCTTTCCATTCAATTTGTTATGATATTTTGTAAAAAGTTTTATGCATATCAAAATTCTTCAATTATTTTGTAG

GACCTCACATTGGGTGAGATTTCAAGAGAGTTCTTACTAACAGGCATAAGACTACATCAAATTTAGTGAGTGCTAGCTTTCGTCACTATCCAGACATGGAAAGAACTTTGGCTGCCTGTGTTAGTTTCACAGTGAATGGAGATTCATCTGAAAGAATTTCATGCAATATATTCATCTGCAATAGACTGCATTGTTCTTTTTCAAGATCATTTCTTCCATCAAAATCAGAATATGTGTGGTTAGTGTCAACTTCTCTAGCTTGGGGCTCCATGGAGGTGAATGATTGGAAAGAAGTTTTGGTTTGGTTTGAGGTTCATGATGAGGTAAATACAAGTATAAGAAGATGTGGTGTCCATGTCACTGAAGAGCTCCATGGGATAATACAAATGGATGTCAAGTGGCCGGTGGTAAATTATGCTGATTTTTATCAACTGGAGAAATTGCAAAATCT

GTAAGTTGATTGTTTACTCTTTTTTTATTTTGAGTGAAAGTAGGATTAGGTTAATATATGGACGGATATGATAATATGTTGTTTTAACTTAATGGCACACAG

GGATATTGAGGATCTTCTTGTCAAACGCTTTTTTGAAGAAATGTCCTCCTTGTCAAATTGCAAAGCAATGTTGCATGCAGGAAGTTATGCTCCAGAAGCAATAATTGGTTCCAACGTACAACCTATGGTTTTCCCGTTGCACGTATCATATAATGGTGATACAGTGATATGTGGAATGGAAGGCATGGCAAAAACTGCACTAGCCAACTCTGTATGCAACAAATTTAATTGGTCATATGATAATGATTGGAGACAACCTTTAGATAATCCTACAAGCTTTTACTTGGTCGAAGAATCACAGTACCGTTTCATGAGATATTCAGGCCTCGACAAGCGTGGAGGTTGTAAAAAAGGGACCAACATTATCGCAAACCATAGTACAATTACATCATCCAAAATGTATTACATATACTTTGAAAATTTGGATGATAGAGTATACAGATTTGTAAGTGCATGGGCTATAGCAAAGCCTCGTTGGATTGAGATTTACAGATTTGAAGATCATGATGTTGCAAGAAATCATCATTTCATTATTAGAAGGGTTGATCCATCCTTATGGCAACCCTGGGCCCGTTGA

>Cucsa.091470

ATGGATTCTTCCACTGTTGCAACAGAATCACCGACTTTCAAATGGACTTATGATGTGTTTTTGAGTTTCAGAGGAGAGGATACTCGCACAAATTTCACCAGTCATCTTGATATGGCCTTGCGTCAAAAGGGTGTCAATGTCTTCATAGACAACAAGCTCGAAAGGGGTGAGCAAATTTCTGAATCCCTTTTCAAATCTATACAGGAAGCTTCCATTTCTATTGTTATATTCTCTCAAAATTATGCATCTTCTTCCTGGTGTCTGGATGAATTGGTGAACATAATTGAGTGTAAGAAATCCAAGGGCCAGAATGTTTTCCCAGTTTTCTATAAGGTGGATCCGTCCGATATACGAAAACAAACTGGTAGCTTCGGAGAAGCACTGGCCAAACATCAGCCTAAGTTCCAAACAAAGACCCAAATTTGGAGGGAAGCTTTAACTACTGCTGCTAACTTGTCTGGTTGGAATCTAGGAACTAG

GTATATATTTTTACAGACATTTTTTTTTTCCATTCCAAATTTCATTTTTATGCCCATGATTTGTATGTATGTACTTATGTATGTATTTACGTATGTATGTAAATAAGCACACAGACCAAATTTTAATTTTAGAAGATTCATTCTCTCTCAAGATGCAATTTAATAATTAAATGTGTTCATCCCTTTTAATTTTGGTATGTTTATGCACTTAACAACAG

GAAGGAGGCTGATCTTATTGGAGATCTTGTTAAAAAAGTGTTGTCTGTATTAAACCGCACTTGCACGCCCTTATATGTAGCTAAGTATCCGGTTGGAATTGATTCTAAACTAGAATATATGAAGCTTCGTTCACATAATCTTTTTGAGAAGAGCAACAAATTCCATTATCGAAAACAACATGAGTATGAGTCTGATACTGGTGTTTACATGGTGGGCTTATATGGCATTGGAGGCATTGGTAAGACAACTTTGGCTAAAGCTTTATACAACAAAATTGCTAGCCAATTTGAAGCTTGCTGCTTTCTATCAAATGTTAGAGAAGCTTCAAAGCAATTCAATGGCCTTGCTCAACTACAGGAAACCCTACTCTATGAGATCCTAACGGTTGATTTGAAGGTTATCAACCTTGATAGAGGAATTAACATCATAAGGAATAGATTGTGTTTGAAGAAAGTCCTTATAGTTCTTGATGATGTAGATAAGCTTGAGCAGTTAGAAGCATTGGTTGGCGGGCGTGATTGGTTTGGCCAAGGCAGTAGAATCATTGTGACGACAAGGAACAAACATTTACTTTCTAGCCATGGCTTTGATGAAATGGAAAATATTCTAGGATTGGATGAAGACGAAGCTATTGAGCTTTTTAGTTGGCATGCTTTCAAGAAAAATCATCCATCAAGTAATTATTTAGACCTTTCAAAACGTGCTACAAGTTATTGTAAAGGCCATTCTTTGGCTCTCGTTGTTTTGGGTTCTTTCCTCTGTACCAGAGATCAAGTAGAATGGTGTAGTATATTAGATGAATTTGAAAACTCTTTGAACAAAGATATCAAAGATATCCTTCAATTAAGTTTTGATGGTCTGGAAGACAAAGTAAAGGATATCTTTCTTGATATTTCTTGTTTACTTGTGGGAGAGAAAGTTAAGTACGTTAAGAATATGTTGAGTGCATGTCATGTAAATCTAGATTTTGGAATTATAGTACTCACGGATCTTTCATTTATTACGATTGAAAATGACATAATGCAAATGCATGATTTAATAAAACAGATGGGTCATAAAATAGTTTGTGGTGAATCTCTTGAGCTTGGAAAGAGGAGTAGGTTGTGGTTGGTACAGGATGTTTGGGAG

GTGCTTGTTAATAATTCAGTGAGTAACTCTCTTTAACATACTTATTTATAATATTTCAAATAACGAAGAAGGTTTGTGAAATGTTGTTAAGTTACTAAATATATAACTTTTTCAG

GGAACAGACGCAGTTAAAGGCATAAAGTTGGACTTTCCTAATTCCACGAGGCTGGATGTGGATCCACAAGCTTTTAGAAAAATGAAAAATTTGAGATTGCTTATTGTTCAAAATGCAAGATTTTCTACAAAGATTGAGTACCTACCTGATAGCTTAAAGTGGATTAAGTGGCATGGATTTCGTCAACCAACTTTTCCTTCGTTCTTCACTATGAAAAATCTTGTTGGACTAGATTTGCAACATAGCTTCATCAAAACATTTGGGAAAAGACTTGAG

GTAAAGTTTATTTCTATATATGCAATAAATGGGTAACTTACATTTTAAAATAATTCACAAATAACACAGACCTATAATAATAGTTTTCGAATGTAAGGAGTTTTTTGTGGCGTTAAGTTGGGATATGATGATGTGGAGTTTATATTGTCTATGTGTGTGGTGGAGAGTTGCTTTTTCTAGGTTCATATGTTTGTACTTAGGATGCAGAGTTGAGTTAAGGTATCCATATGCAATTTTTTGAATTATAAATAATTTGTTTTTTATAACTATTTCTTTTTTAAATTTCTTTATTAAATACTTATATATATATATATATATAAACATTTTTTAAAATAGCAAAATAAATTAAAATATTTACGAGCTATAGCAAAATTTTGGATTCTATTAATGATAGTCATTGATAAACTTCTATCACCGTAGTATATTAGTGATAGAATTTAAAAATTTTGCTATGAGTTGTAAATATTTTGTAAAATTTGCTATTTTTTAAATTCCTCCCTTATTATTAGTTTGCCTAAATACTGATTAATGCTTATTTATAGAATTTTTTTACCTATATTATTCTATAATTTTTTATATTTACAATTATTGTTTTAGCCACTGATTTATCTAAATTTTGTTTGGTATGCTAATTACATACCTCTTTTCCTTTTGGTTTATTTTCCAATGTGTTTTAG

GATTGTGAAAGGTTGAAGTATGTTGATCTTAGCTACTCTACTTTCTTAGAGAAAATTCCTAATTTCTCTGCAGCATCAAACCTTGAAGAGTTGTATCTCACCAATTGCACAAATTTAGGAATGATAGATAAGTCTGTTTTCTCTCTCGATAAGCTTACTGTCCTAAACCTTGATGGTTGTTCTAACCTTAAAAAGCTTCCAAGAGGCTACTTCATGTTAAGTTCTCTTAAAAAATTGAATCTCTCTTACTGCAAAAAACTTGAGAAAATTCCAGACTTATCTTCAGCATCAAACCTTACGAGCTTGCATATCTACGAATGCACAAATTTAAGAGTAATTCATGAATCTGTTGGATCTTTGGATAAGCTTGAAGGTTTGTACCTTAAACAATGCACTAACCTGGTAAAGCTTCCAAGCTATCTCAGCTTAAAGTCTCTTCTATGTTTATCACTTTCTGGGTGTTGTAAGCTTGAAAGCTTCCCAACAATTGCTAAAAACATGAAATCTTTAAGGACCTTGGATTTGGATTTTACTGCCATAAAGGAGTTACCTTCATCAATTAGATATCTCACTGAGCTTTGGACATTAAAACTTAATGGTTGCACAAACCTCATCTCCCTTCCCAATACAATTTATTTGTTAAGGAGTCTTGAGAATCTTCTTCTTAGTGGCTGTTCTATATTTGGAATGTTTCCCGATAAATGGAACCCAACCATCCAACCAGTATGCTCTCCTTCAAAAATGATGGAAACTGCTTTGTGGAGCTTAAAAGTTCCCCATTTCCTAGTACCAAATGAAAGTTTTTCCCATTTCACTTTGTTGGATCTTCAGTCTTGCAACATATCAAATGCAAATTTTTTGGATATTTTATGTGACGTTGCTCCTTTCTTATCTGATCTACGCTTGTCCGAAAACAAATTCTCTAGTTTACCCTCATGTCTCCACAAGTTCATGTCGTTGTGGAATCTTGAATTAAGGAATTGTAAGTTTCTTCAAGAAATTCCAAGCCTTCCTGAGAGTATACAAAAAATGGATGCCTGTGGTTGTGAATCATTGTCTCGAATTCCAGATAACATTGTGGATATAATATCAAAAAAACAG

GTTTGCCTCTAATTTCCACTCGATTTATATTCTTACCTTGTAAACAAATTAATGCATTGAGAATTTTTGTTCTCTATAG

GACCTCACAATGGGTGAGATTTCAAGAGAGTTTTTATTAACGGGGATTGAGATTCCAGAATGGTTCAGCTATAAGACTACATCCAATTTGGTGAGTGCTAGCTTTCGTCACTATCCAGACATGGAAAGAACTTTGGCTGCCTGTGTTAGTTTCAAAGTAAATGGAAATTCATCTGAAAGAGGTGCCCGGATTTCATGCAATATATTCGTCTGCAATAGACTCTATTTTTCATTGTCAAGACCATTTCTTCCCTCAAAATCAGAATATATGTGGTTAGTAACAACTTCTCTAGCGTTGGGTTCCATGGAGGTGAATGACTGGAATAAAGTTTTGGTCTGGTTTGAGGTTCATGAAGCACATAGTGAGGTTAATGCAACTATAACAAGGTATGGTGTCCATGTCACTGAAGAGCTCCATGCGATACAAACGGATGTCAAGTGGCCGATGGTAAATTATGCTGATTTTTATCAACTGGAGAAATTGCAAAGTCT

GTAAGTTGATTCTTTACTTGTTAGTTATTTACTTTTTTTGTTTGGAGTTGAAGTATGATAGATCTCAAAGGGGAGATGTGTATGTTATGATTATGTTTGTTTAATGGCATAG

GGATATTGAGGAACTTCTTCTCAAACGCCTTTTTGAAGAAATGTCGTGCTGGTCCAATTCCCAAGCAATGTTATATGCGGCAAATTATGATCCAGAAGCAATAATCGATTCGAATATACAACCTATGATATTTCCATTGCACGTAACATATAATGGTGAGACATTTATATGTGGAATGGAAGGCATGGGAGACACTACACTCGCCAACTCTTTATGCAATAAATTTAATAGGTCAAATGACAACGGTTGGCCAAGAGAAGCTTTAGATGATTCTACAAGCTTTTTGCATTTTCGAGGAGGAAAGTTTTATGGAGGTTCCTGGTCATTGTCCCACCACCGTAAGCGTGGAGATGGTGAAAGAGGAACCAATATCACAACCCGCACAATATCCTCCAAACGCTATTTGATACTCTTTCATAAAGCGGGGAGCTATAATCATTTATTTAACTTTGCTGGTAGCCACCGTTTGATTGCAGGTTCTGGCAGTTATGACAGTCTTAACGGAAGAGGTGATGTTCGGCTTCTGATTGAAAGGGTTGATACATCCTTGCTCTGA

>Cucsa.091680

ATGCAGTTGGATGTAGCTAAATATCCAGTTGGAATTGACATACAAGTTAGAAATTTACTCCCACATGTTATGTCTAATGGAATTACCATGTTTGGATTATATGGAGTTGGAGGTATGGGCAAGACAACTATAGCGAAAGCTTTATACAATAAAATTGCTGATGAGTTTGAAGGTTGTTGCTTTCTGTCAAATATTAGAGAAGCTTCGAATCAATATGGAGGCCTTGTTCAATTTCAAAAGGAGCTACTTTGTGAGATTCTAATGGATGATTCGATTAAAGTTAGCAATCTTCCTAGAGGAATTACTATCATAAGGAATCGACTATACTCAAAAAAGATTCTTTTGATTCTTGATGATGTTGATACGCGTGAACAACTACAGGCATTGGCGGGAGGACATGATTGGTTTGGACATGGAAGTAAGGTGATTGCGACAACAAGAAACAAGCAATTACTTGTTACTCATGGATTTGATAAAATGCAAAATGTTGGGGGATTGGATTACGATGAAGCTCTTGAGCTCTTTAGTTGGCATTGTTTTAGGAATAGTCATCCCTTAAATGTTTATTTAGAACTTTCAAAACGTGCCGTAGATTATTGTAAAGGCCTTCCCTTAGCTCTTGAAGTTTTAGGTTCCTTCCTTCATTCTATTGGTGATCCCTCCAATTTTAAACGTATATTGGATGAATATGAAAAACATTACCTCGATAAGGACATCCAAGACTCTCTTCGAATAAGTTACGATGGACTCGAAGATGAAGTAAAAGAAATATTTTGCTATATTTCTTGTTGCTTTGTACGAGAAGATATATGCAAAGTTAAAATGATGTTAGAAGCATGTGGTTGTTTATGTTTGGAAAAGGGAATAACTAAACTTATGAATCTATCACTTCTTACCATTGGTAGATTCAACAGAGTTGAAATGCATAACATAATACAACAAATGGGTCGGACAATTCATCTTTCAGAAACTTCTAAATCTCACAAAAGAAAAAGATTGTTGATTAAAGATGATGCTATGGATGTTTTAAATGGGAATAAGGTAAAAATTATTG

GTCAAACTTTAGCATGAGAACAAGTTTTATTATTTTGAAAATTAATAACTTGAAATGTTTATTTTGTAACCTTTTGTAGGAAG

CAAGAGCAGTGAAAGTCATAAAACTAAATTTTCCTAAACCTACGAAGTTGGACATCGATTCAAGAGCTTTTGATAAAGTGAAAAATTTGGTAGTACTGGAAGTTGGCAATGCCACATCTTCAGAAAGTTCTACTCTTGAGTATCTACCCAGTAGCTTAAGGTGGATGAATTGGCCTCAATTTCCTTTTTCATCTTTGCCTACAACCTACACAATGGAGAACCTTATTGAATTGAAATTGCCATATAGCTCCATCAAACATTTTGGTCAAGGATATATG

GTATTTATATAATATAAATATAATTGCATTTGTTTTTAATTTCTATGTTGAAATTTGATTGTTACTTTCTTCTTTATATTCATTGCAG

AGTTGTGAAAGGTTGAAGGAAATTAATCTTAGTGACTCCAATCTTTTGGTGGAAATCCCAGATTTATCTACTGCAATAAACCTCAAATACTTGAATCTTGTAGGATGTGAAAATTTAGTAAAAGTTCATGAATCAATTGGATCTCTCAGTAAGCTTGTCGCCCTTCATTTTTCTAGTAGTGTTAAGGGCTTTGAGCAGTTTCCATCCTGCCTCAAGTTGAAATCTCTTAAGTTTTTGTCAATGAAAAATTGTAGAATAGATGAATGGTGTCCCCAATTCAGTGAAGAAATGAAGTCTATAGAATATTTATCGATTGGGTACAGTACTGTAACATATCAGCTATCTCCAACAATTGGATATCTTACTAGCCTAAAACATTTGAGCCTCTATTATTGCAAAGAGCTCACAACTCTTCCAAGTACAATTTATCGTTTAACCAATCTTACTTCTTTAACTGTGTTGGATTCTAATCTTTCAACATTTCCTTTCTTAAATCATCCGTCCTTACCTTCCTCACTTTTTTACCTAACCAAGTTACGTATTGTTGGTTGCAAGATAACAAATTTGGATTTCTTAGAAACAATTGTTTATGTCGCCCCTTCATTGAAAGAGTTGGACTTGTCCGAAAACAACTTTTGTAGATTACCGTCATGTATTATTAATTTTAAATCTCTAAAATATCTTTATACGATGGATTGTGAGTTGCTTGAAGAAATTTCAAAGGTTCCCGAAGGCGTAATTTGTATGAGTGCTGCTGGGAGCATATCATTGGCTAGATTTCCTAACAACTTGGCTGATTTCATGTCTTGTGATGATTCT

GTGGTGCGTACCATCTCTTTCCCATGACTTCATCATTATCTCTAGCTCATGTATTTAATTTCATTCATATAATATATATTACTTATAACTATTTACTGATCTCATGGTGCAG

GAATATTGTAAGGGTGGAGAATTGAAACAACTGGTATTAATGAATTGTCATATTCCAGATTGGTATAGGTACAAGAGCATGAGCGATTCATTAACATTTTTTTTGCCAGCTGATTATCTAAGTTGGAAATGGAAGCCTTTGTTTGCTCCTTGTGTCAAATTTGAAGTTACGAATGATGATTGGTTCCAGAAGCTTGAATGTAAAGTTTTTATCAACGATATTCAAGTATGGAGTTCTGAAGAGGTGTATGCCAATCAGAAGGAACGGAGTGGGATGTTTGGAAAAGTATCACCAGGTGAGTATATGTGGCTGATAGTACTTGATCCTCATACACGTTTCCAATCATATTCGGATGATATCATGGACAGGAGGTCACTGAAGATTATTGATCTAAATCAACTAAGTTCTGAGATTAATTCCTCACAAAGTATTTTGGGTAAAATTACGGTGTCATTTGAGGTTACTCCATGGTATAAAGACGTAGTTATAAAAATGTGTGGTGTTCATGTCATCATGGGGGAATGA

>Cucsa.091690

ATGGATAGAAGTAGGGTTGGATTTAATTAGATTCAGTTTATTTGGTCGTTTTCCTTTTCTCTTCTGCTTCTCATATTAAGAAATGTTGCTGAAAAAAAAAATAATTTCATTCATCTTTGTCATGTATGTTTTTTTTCTTTAAAATGTCAGTGAAAATGAAGCAAGTTTGATTCAAATAATTGTTCAAGAAGTCAGAAAGAAATTAAAGAATAGTGCAACAACGGAGTTAGATGTAGCTAAATATCCAGTTGGAATTGACATACAAGTTAGTAATTTACTGCCACATGTTATGTCTAATGAAATTACTATGGTTGGATTGTATGGAATTGGAGGTATGGGCAAGACAACTTTGGCCAAAGCTTTATACAATAAAATTTCTGATGACTTTGAAGGTTGTTGCTTTTTGGCAAATGTTAGAGAAGCTTCAAATCAATACTGGGGTCTTGTTGAACTCCAAAAGACGCTAATTCGTGAGATTCTGATGGATGATTCAATCAAAGTTAGCAATGTTGGTATAGGAATTAGCATCATAAGGGATCGATTATGCTCAAAAAAGATAATTTTGATTCTTGATGACATTGATACACATGAACAACTACAGGCATTGGCTGGAGGACATGATTGGTTTGGACATGGAAGTAAGGTCATTGCAACAACAAGAAACAAGCAATTACTTGCTAGTCATGGGTTTAATATATTGAAAAGAGTTAACGGATTAAATGCGATTGAAGGTCTTGAGCTTTTTAGTTGGCATGCATTTAAAAATAGTCATCCCTCAAGTGATTATTTAGATGTTTCAAAACGTGCTGTGCATTATTGTAAAGGTCTTCCCTTAGCACTTGAAGTGTTAGGTTCCTTCCTTAATTCTATCGATGATCAATCCAAGTTTGAACGTATATTGGATGAATATGAGAACTCATACCTGGACAAAGGCATCCAAGATATTCTTCGAATAAGTTATGATGAGCTTGAACAAGATGTAAAAGAAATTTTCCTTTACATTTCTTGTTGCTTTGTACATGAAGACAAAAACGAGGTTCAAATGATGTTAAAAGAATGTGACTCTCGTTTCAGATTGGAAATGGGTATTAAAAAACTCACTGATCTATCACTTCTTACAATTGATAAATTCAACCGGGTCGAAATGCATGACTTGATACAACAAATGGGTCACACAATTCATCTCTTGGAGACTTCTAATTCTCATAAAAGAAAAAGATTGTTGTTTGAAAAAGACGTCATGGATGTCTTAAATGGAGATATGGTGAGAAG

GTTTACACAAAGTTTATTCTGTATATATTGATCATTTTATTATTTCATAGCTGACTTTAATTCAAAGTTTTATTTTGTGTGTCACTGCAGGAAGCAAG

GGCTGTGAAAGTCATAAAGCTAAATTTTCATCAGCCCACTGAGCTAGACATTGATTCAAGAGGTTTTGAAAAAGTGAAAAACTTGGTAGTGCTCAAAGTTCACAACGTCACATCTTCAAAAAGTCTCGAGTATCTACCGAGTAGCTTAAGGTGGATGATTTGGCCTAAATTTCCGTTTTCATCTTTGCCTTCAACCTACTCACTGGAGAAACTTACTGAACTCAGCATGCCAAGTAGCTTCATCAAACATTTTGGAAATGGATATCTG

GTACTAATTGATCGTGTCTATATATACTTAACAATGATGCATTAATTTAATTAATTAACTTTAAATGTCAGCCAAATTATTTGAAATTTGATTCTATTTTCTTTTGTAATTCATTGCAG

AATTGCAAATGGTTGAAGCGTATAAATCTTAACTACTCAAAGTTTTTAGAAGAAATTTCTGATCTATCCAGTGCGATAAATCTTGAAGAGTTGAATCTTTCCGAGTGTAAAAAGCTGGTAAGAGTTCATGAATCAGTTGGATCACTAGGTAAACTTGCTAAATTGGAACTTTCTAGTCATCCTAATGGCTTTACGCAATTTCCATCCAACCTCAAGTTGAAGTCCCTACAAAAATTG

GTAATGTACGAGTGCAGGATTGTTGAAAGTTATCCTCATTTCAGTGAAGAAATGAAGTCTAGTTTAAAAGAATTACGGATTCAGTCTTGTAG

TGTGACAAAACTATCCCCAACGATTGGAAATCTTACTGGTCTTCAACATTTGTGGATCGATGTATGCAAAGAGCTCACTACTCTTCCAA

GTACCATTTGTCATTTAAGCAATCTTATTTCTTTAAGTGTTTTCAGATCTGAAGTTTCAACTTTTTCGTTCTTATATTCTCGTTCCCTTTCCTTATTTCCCTACCTAACACTTTTAAAACTTTGTTACTGCAAGATAACAAATTTGAGTTTCTTAGAAACAATCACCCATGTCGCCCCTTCATTGACACAGTTGTACTTGACTGGAAACGACTTTTGTAGCCTACCCTCATGTATTGTTAATTTTAAATCTTTGAGATATTTTGATATAAGTTATTGTGGGTTCCTTGAAGAAATTTTAAAGGTTCCAGAAGGCGTAATTTATATGAATGCTCAAGGGTGCAGATCATTGGCTAGATTTCCAGACAATATAGCTGAATTCATATCTTGTGATTCGGTACATATCATCTCCTTTCTCCTGGCCTTCATCTCTCATGTACTTTTCTCATATTATATATATTTTAACACATTCACTCTTATCTATAAATGGTGCAGGAATATGTAG

ATGGAAAATACAAACAACTCATATTAATGAATAATTGTGATATTCCAGAATGGTTTCATTTCAAGAGTACGAACAATTCAATAACGTTTCCTACGACATTTAATTATCCGGGTTGGAAATTGAAAGTTCTTGCTGCTTGTGTTAAAGTTCAAGTTCATGATCCTGTTAATGGGTATCATAGAGGGGGGGATCTTGAATGTGAAGTGTTCTTTAAGGACATTCTAGTATGGAGTTCTGGAGACTGGACAAATTATCTTGGATACGATTCAAGATGGTTGCCCCTAGGAGCATCACCAAGTGAGTATACATGGTTTATTGTACTCAATCCTCATAGAGATTTCTCCCTAGATGATTGGGATGATATGATGGAGAGATCACCAGAGACTGATCTAAGTCAGCTATGTTTTGGAATTAATTCCATGGAAATGGACCGTAATAGATCAAATGATAAATGGAATTCTATTGGGGGAAGTATTTGGAAGAACTTTACGGTGTTGTTTGAGCCTCGTCCCCTGTCTCCAGACACTACAATAAGTATAAAAGGTTGTGGTGTTCATGTCATCATGGAGTAA

>Cucsa.091710

ATGGTTGGATTGCATGGAATTGGAGGTATGGGCAAGACAACTTTGGCCAAAACATTATACAATCGGATTGCTGATGACTTTGAAGGCTGTTGTTTTTTAGCAAATATTAGAGAAGCTTCAAAGCAACACGAGGGACTTGTTCGACTCCAAGAGAAACTACTTTATGAGATTTTAATGGATGACTTTATTAGAGTTAGTGATCTTTACAAAGGAATCAACATCATAAGGAATCGACTATGCTCCAAAAAGATTCTCTTAATTCTTGATGATATAGATACCAGTGAACAACTACAGGTATTAGCTGGAGGATACGATTGGTTTGGATATGGAAGTAAGGTCATTGTGACAACAAGAAACGAACACTTACTTGATATCCATGGATTTAATAAATTGCGAAGTGTTCCTGAATTGAATTATGGTGAAGCTCTTGAGCTTTTTAGCTGGCATGCTTTTCAGTGTAGTAGTCCACCAACCGAGTATTTACAACTTTCAAAAGATGCTGTAAATTATTGTAAAAATCTTCCCTTGGCGCTTGAAGTTTTAGGTTCATTCCTTTATTCAACTGATCAATCCAAATTTAAAGGTATATTGGAGGAATTTGCAATCTCCAACCTTGACAAAGACATCCAAAATCTTCTTCAAGTAAGTTACGATGAACTTGAAGGTGATGTACAAGAAATGTTCTTGTTTATTTCTTGTTTCTTTGTGGGAGAAGATAAAACCATGGTTGAAACGATGTTGAAGAGTTGTGGTTGTTTATGTTGGGAAAAGGGAATTCAAAAACTCATGAATCTATCACTTCTTACTATTAACCAATGGAACAAAGTTGAAATGCATGACTTAATACAACAATTGGGTCACACAATCGCACGTTCAAAGACTTCTATATCTCCTTCAGAAAAAAAATTATTGGTTGGAGATGATGCTATGCAT

GTGCTAGATGGCATTAAGGTAAGAAATTTTTGCTCAATCTTTTGATTATGCTTATTAATTATTATGAGAGCAACTTTTATCATTTTGAAAACAAGTTGAAATGTATGTTTTTGTATAATTTGTAG

GATGCAAGAGCAGTTAAAGCCATAAAGTTAGAATTTCCTAAACCGACAAAGTTGGACATTATTGATTCAACGGCTTTTAGAAAAGTAAAGAACCTTGTAGTACTCAAAGTGAAGAATGTCATATCTCCAAAAATTAGTACTCTTGATTTTCTACCTAATAGCTTAAGGTGGATGAGTTGGTCTGAATTTCCTTTTTCATCATTTCCTTCAAGCTACTCAATGGAGAACCTTATTCAACTCAAATTGCCACATAGCGCCATCCAACATTTTGGAAGAGCATTTATG

GTATTTCTATGGATACTTAGACTTCTATTAATTTCATTGTTTGGTTTATTTGAAATTTGATTATATTTTTTCTGTATATTCATAACAG

CATTGTGAAAGGTTGAAGCAACTTGATCTTAGCAACTCCTTCTTTTTGGAGGAAATTCCTGATTTATCTGCGGCAATAAACCTCGAAAATTTGTCTCTTTCTGGATGTATAAGTTTAGTAAAGGTTCATAAATCAGTTGGATCTCTTCCTAAACTTATTGATTTGAGTCTTTCAAGCCATGTTTATGGCTTTAAGCAGTTTCCTTCACCACTCAGGTTGAAATCCCTTAAAAGATTTTCAACTGATCATTGTACAATACTTCAAGGCTATCCTCAATTCAGCCAAGAAATGAAGTCTAGTCTAGAAGATTTGTGGTTTCAAAGTAGTTCTATAACAAAGCTATCTTCAACAATTAGATATCTTACCAGCCTCAAAGATTTGACCATCGTGGATTGCAAAAAGCTCACTACTCTTCCAAGTACAATTTATGACTTGAGCAAACTTACATCCATAGAAGTCTCACAATCCGATCTTTCAACATTTCCTTCCTCATATTCCTGCCCTTCCTCACTTCCCCTCCTAACAAGATTACACCTTTATGAGAACAAGATAACAAATTTAGATTTTTTGGAAACTATCGCTCATGCTGCTCCATCACTGAGAGAGTTGAACTTGTCTAACAACAACTTTTCTATACTACCTTCATGTATTGTTAATTTTAAATCCTTGAGATTTCTTGAAACATTTGATTGTAAGTTTCTGGAAGAAATTCCAAAGATTCCAGAAGGCTTAATTTCTTTGGGTGCATATCATTGGCCAAATCTCCCGACAACTTAGCTGATTTCATATCTTGTGATTCG

GTGCATATCTCATCTCTTTATCTTGTCTTGATCTCTCCTATAATTTTTATTTTCATATAGTATAGCTAAGTTATTCATGTGTGGTTGTAAATTAATAGGATTATTACTCTCACATTCACGTGGTGCAG

GAACATGTAGATGGACACTTCAAACAACTCATATTAATGAATTGTGATATTCCAGATTGGTTCAGTTACAAGAGTAGAAACAATCACATAACGATTTTTATGCCATCCAATGATCCAAGTTGGGAAATGAAGGTTTTTGCTCCTTGTGTCAAATTCCAAGTTAATCATGTTGACCGGAATCGTTATATGGATCTTGAATGTAAAATGTTCATAAACGACATTCAAGTATGGAGTTCTGAAGAAGTACCCTGTCTTGAAGAATCGAGAAGGATATTGATAGAAGAATCACCATATGAGTATATGTGGTTATTAGTACTATATCCTCATATAGATTTCCCACTAAATTCGGAAGATATTGTCAAGAGATCACAGGAGTACTTTAATCTACATCAGCCAAGTTTTGGGATCAATTCCATGGGAAGGGACAATAATAATTGTAATGTAGATGATGATCGGAGGAATCATTTTGGGGAGA

GTATTTGGAGGAAATTTACGGTGTCATTTGGTGTCAATTCCAAATTTAAAGACTCCCAAGTAAGTATAAAAAGGTGTGGTGTTCATGTCATCATGGAG

GAATGGCGAGACTGATGTAGCCTGAATGTAAAAGAACAATCAAATGAAATTTATACTAAAAGTACTAAGTATTACAAGTGAAAAAATCGAGTGGATCCACCGGAAGCACGAAGATTAATTTGTCAAAGTTATTTTTTTAATTACAGTGATAAAATATGAGGTTTGGTTTGGTTTGAGATTCAAGTGAATAAAGTGGAAGAAAAATAAAGAGATTTGCAATATAGTTAAAACATTGTAGTATAGAGGAATGAAATCACCCAATGCAGTTTTGAACATCGAATATTATCAACTTATAAATTTTTTAGCTTCTTACCTATATCAAGCCCAAAA

>Cucsa.091780

ATGGCCAAACATCAGCCTAAGTTCCAAACAAAGACCCAAATTTGGAGGAAAGCTTTAACTACTGCTGCTAACTTGTCTGGTTGGGATCTAGGAGCTTATAG

GTATATATTTTTACGTAAATCTTGCTTGTCCTATCTTTTCCAGATCTCATTTTCAATGCCATGTCTTTTTTTTTTTACTCATTTATCAACGAGCACACTGCACAACTTTTGTCTTAGAAGATCCATTCTCTCGCAAGATGCCATTTAATTAACTGTGAACATCATTCTTTAGTTTTGTTATCATTACGTATTTAACAACAG

GAGGGAGGCTGATCTTATTCGGGATCTTGTTAAGGAAGTGTTATCTACAATAAATCGCACTCGCACACCCTTATATGTCGCCAAGTATCCAGTTGGAATTGATTCTCAACTAGAATACATGAAGTTTCACTCACATCATCTCAACAAGGGAAACAAATTCCAATATTGGACACAAAATGAGTATGAGTCTGATATTGGTGTTTACATGGTGGGGATATATGGCATTGGAGGCCTTGGTAAGACAACTTTGGCTAAAGCTCTATACAATAAAATAGCTAGCCAATTTGAAGGGTGCTGTTTTCTATCAAATGTTCGACAAGCTTCAAACCAATTCAATGGCCTTGTTCAACTACAGCAAAACCTACTCTATGAAATCTTAGAGGATGATTTGAAGTTTGTCAATCTTGATAAAGGAATTACCATCATAAGGAATAGACTGCGTTCAAAGAAAGTTTTGATAGTTCTTGATGATGTGGATAAGCTCGAACAACTAGAAGCATTGGTTGGTGGACGTGATTGGTTTGGTCAAGGTAGTAAAATCATAGTGACGACGAGGAATAGTCATTTACTTTCTAGCCATGGATTTGATGAAATGCACAATATTCAAGGATTGAATCAAGACAGAGCTATTGAGCTTTTTAGTTGGCATGCTTTTAAGGAAAGTCATCCATCAAGTAATTATTTAGACCTTGCCGAACGTGCTACAAGTTATTGTAAAGGTCATCCTTTGGCTCTTGTTGTTCTGGGTTCTTTCCTTTGTAATAGAGGTCAAACAGAATGGAGAAGTATATTGGATAAATTTGAAAACTCTTTGAACAATGATATTAAAGATATTCTTCAATTAAGTTTTGATGGGCTGGAAGGTGGAGTAAAGGATATTTTTCTTGATATTTCTTGTTTATTTGTAGGGGAAAAATACAATAATTGTGCTAAAAAAATGTTGAGTGCATGCCATTTGAACGTAGATTTTGGAATTATGATACTCATGGATCTTTCACTTGTTACGATTGAAAAGGATAGAGTGCAAATGCACGGATTAATACAACAGATGGGTCATAGCATAGTTCATAATGAATCATTTGAGTCAGGAAAGAGGAGTAGATTGTGGTCGGAGCGGGACATTTGGAAC

GTGTTTGTTAATAATTCGGTGAGTAACTCTTTCCTAAAATATCGAATATTTTAATTATTTTCAGGACTTCACCTGATAATAAATGTTTGTGAAAGTAAGTTGTCAATAATGTTAAATTACTAAATCTATGACTTCGTAG

GGAACAGATGCAATTAAAGCCATAAAGTTAGACTTGCCTAATCCCATAAACGTAAATGTAGATCCAAAAGCATTCTTTAGAAGCATGAAAAATTTGAGATTGCTTATCATTCGAAATGCACAAGTTTGTACAAAGATTAAGTACCTACCTAATAGCTTAAAGTGGATTGAGTGGCAAGGATTTGCTCATCGAACTTTCCCGTCGTGCTTCATTACCAAAAATCTTGTTGGACTTGATTTGCGACATAGCTTCATCAAAAGATTTGGGAAGAGACTTGAG

GTAAAATATATTTCTTTCCTTATGTGTATTTGATTGGAGGTTTCTTCATTGTTTTTCTTGAAAACTTTTAATGGATAGCTTAACTACATTTTACAAAAAAAAATCACAAATGCGATTTTAGCAAAATTTATCATGGATTAACTTCTAGTAGTCATATATTTTTAACAAATTTACAAAATATAACAAAATTTATCAATGATAAACTGATCTATCGTACAACAATAGGCTCCTACTAGTCCTATGGTCTATCATTAATAGACTCCTATTAATGATATGATCTACCGTGGCTAAAATTTAAAAGTTGAAGTTTTACAATTTTTTGGATTGTAATATATATGTTATCATTTCGAGGGTTGATTTTTTTTAATGCAATTATCATAATGCAAAATAATATATTGGGTAGATTCTTTTTATGTTATGACTTTACTTGAATAAGATTTGTTATATAGAAGCTAAGATTGTCCAATTATATTGTGTGTCGTTTCAGTTACAAGATTTCGTATTAGGTTAAGTTTTTAATTTTTTTAATTTTAAATTTGATTTAAGATCAAATCAAACTAAAAATTGAAATCAAATTTCGAGTGTAATTAAATTGACTAAACTACATTCTCAATCTGTTTAAGATATCAAATTAAATGGTTAGTTTACAATCTACAAAATTCAAGATAATATATTTTAGTCATAATGGTTACTTTAAAAAATAATAATTAATTAATAAATTACTTTTAAACTAAAAACTTAATTCAAAACTATGTTTAATTTAAACTAATTATTCTTTAGGTTTTTTAAGCTTTTTGGGGTGGATTACGTTTCAAACTTATAAAACTGAACATCAAAATAATTGTTGTCAAGAGTCCGTTATTTATCAACATGAGTGTAGTACAACTGACTTAATGTTTGTACTATCAATTGGATTGATTACTCATTTATTTTTTGTATATAAAATTTTATAATTAGTTTGCCTAAATACGATTAAAGGTTATTTATAGATTTTTTTTACCTATATTTTATATTTTCTATATTTACAATTATTTTTTTAGCCACTATTTTATCTAAATTTTGTTTGGTATGCCAATTATATTACATTACATACCCCTTTTCCTTTTGGTTTCTTTTTCAATATGTTTTAG

GATTGTGAAAGGTTGAAGCATGTTGATCTTAGCTACTCTACTTTATTAGAGAAAATTCCTGATTTATCCGCTGCATCAAACCTTGAAGAATTGTATCTCATCAATTGCACAAATTTAGGAATGATAGATAAGTCTGTTTTCTCTCTCAATAAGCTTACTGTCCTAAACTTTAAAGGTTGTTCTAACCTTAAAAAGCTTCCAAAAGGCTACTTCATGTTCAGTTCTCTTAAAATATTGAATCTCTCTTACTGCCAAGAACTTGAGAAAATTCCAGACTTATCTTCAGCATCAAACCTTCAGAGCTTGCTACTCAACGGATGCACAAATTTAAGAGTGATTCATGAATCTGTTGGATCTTTGAATGAGCTTGTATTGTTGGACCTTGGACAATGCACTAACCTTTCAAAGCTTCCGAGCTATCTCAGGTTAAAGTCTCTTGTCTATTTAGTACTTTTTGGGTGTGGTAAGCTTGAAAGCTTTCCAACAATTGCTGAAAACATGAAATCTTTAAGGTGCTTGGATTTGCATTCCACCGCCATAAAGGAGTTACCTTCATCACTTGGATATCTTACTCAACTCGATAAATTACACCTTACCGGTTGCACAAACCTCATCTCCCTTCCCAATACAATCTATTTGTTAAGGAATCTTAACGAACTTCATCTTGGTGGGTGTTCTAGATTTGAAATGTTTCCCCATAAATGGGTCCCAACCATCCAACCAGTATGCTCTCCTTCAAAAATGATGGAAGCAGCTTCGTGGAGCTTAGAGTTTCCCCATTTAGTAGTACCAAATGAAAGCATATGTTCCCATTTCACTTTGTTGGATCTTAAATCTTGCAACATATCAAATGCAAAATTTTTGGAAATTTTATGTGATGTTGCCCCTTTCTTATCTGATCTACGTTTGTCCGAAAACAAATTCTCTAGTTTACCCTCATGTCTCCACAAGTTCATGTCCTTATCGAATCTTGAATTAAGGAATTGTAAGTTTCTTCAAGAAATCCCAAACCTTCCCCAAAACATACGAAACTTGGATGCCAGTGGTTGCAAATCGTTGGCTCGAAGTCCAGATAACATTGTGGATATAATATCAATAAAACAG

GTTTGATTCTTTCCATTCATTTTGTTCCTATCTTTGCACATAGACAATTTAATGCATATGAATTTTTATTCTCTATAG

GACCTTGAATTGGGTGAGATTTTAAGAGAGTTCTTATTAACGGACATTGAGATTCCAGAATGGTTCAGCTATAAGACTGCATCCAATTTGGTGACTGCTAGCCTTCGTCACTATCCAGACATGGAAAGAACTTTGGCTGTCGCTGTTAGTTTTAAAGTGAATGGAGATTCATCTGAAAGTGAGGCCCAAATTTCATGCAATATATTCATCTACAATAAACTCCGTTGTTTGTTTTCAAGATCATTTCTTCCATCAAAATCAGAATATATGTGGTTAGTAACAATTTCTCTAGCGTGTTCCCTGGAGGTGAATGATTGGAATAAAGTTTTCGTCTGGTTTGAGGTTCATGAAGCACATGGTGTAACTGTAACAAGGTATGGGGTCCATGTCACTGAACAACTCCATGGGATACAAACGGATGTCAAGTGGCCGATGGTTAATTATGCTGATTTTTATCAACTGGAGAAATTGCGAAGGGATCTGTAA

>Cucsa.091820

ATGGGTTCTTCCATTGTTGGAGCTGAATCATCAACTTCTTCTTCTTCTAGTTTCAAGTGGAGTTTTGATGTGTTTTTGAGTTTTAGGGGAGATGATACTCGTTCTAATTTCACCGGTCATCTTGACATGGCCTTGCGTCAAAAGGGTGTCAATGTCTTCATAGACGACATGCTCAAAAGGGGTGAGCAAATTTCTGAAACCCTTTCCAAAGCTATACAGGAAGCTTTGATTTCTATTGTTATTTTCTCTCAAAATTATGCATCTTCTTCATGGTGTCTGGATGAATTGGTGAAAATAGTTGAGTGTAAGAAATCCAAGGGCCAGCTTGTTTTGCCAATTTTCTACAAGGTGGATCCTTCCGATGTACGAAAACAAACTGGTTGCTTTGGAGAAGCATTGGCCAAACATCAGGCTAATTTCATGGAGAAGACTCAAATATGGAGGGATGCTTTAACTACTGTTGCCAACTTCTCTGGTTGGGATCTAGGAACTAG

GTATATTTTTACTGACATTTTGTCTTTTCTTTTCCATATTTCATTTCCAATTCTCTAATTTTGTTGACAAAGCACACTTAACAAATCCCTACTTGAAGTAATTCCACAATACAAATGACTGATGTTAATCATGGAAGATTCACAATCAAGATGCAGTTTAATTTTATCTACTATTTTCCCATGTTCATCAATTTTTATTTGTTTTCATATGATGTACTTAATAACAG

GAAGGAGGCTGATTTTATTCAAGACCTTGTTAAAGAAGTATTGTCTAGATTAAATTGTGCCAACGGGCAGTTATATGTAGCTAAGTATCCAGTTGGAATTGATTCTCAACTAGAAGATATGAAGTTACTCTCGCATCAGATACGAGATGCGTTTGATGGCGTTTACATGATGGGGATATACGGCATTGGAGGCATTGGTAAGACTACTTTGGCTAAAGCTTTGTACAATAAAATTGCTAACCAATTTGAAGGTTTCTGCTTTCTATCAAATGTTAGAGAAACTTCAAAACAGTTCAATGGACTCGTTCAACTACAGGAAAAACTACTCTATGAAATCTTAAAGTTTGATTTGAAGATTGGCAATCTTGATGAAGGAATTAACATCATAAGAAGTAGATTGCGTTCAAAGAAAGTTCTTATAGTTCTTGATGATGTGGATAAGCTCAAGCAATTGGAAGCATTGGTTGGTGAACGTGATTGGTTTGGCCATGGTAGTAAAATCATTGTGACAACAAGAAATAGTCATTTACTTTCTAGCCATGAATTTGATGAAAAGTATGGTGTTCGGGAATTGAGTCATGGTCATTCCCTTGAACTTTTTAGTTGGCATGCTTTTAAGAAAAGTCATCCATCAAGTAATTACTTAGACCTTTCAAAACGTGCGACAAATTATTGTAAAGGTCATCCTTTGGCCCTTGTTGTTTTGGGTTCTTTCCTTTGTACCCGAGACCAAATAAAATGGAGAACTATATTAGATGAATTTGAGAACTCTTTGAGTGAAGACATTGAACATATTATTCAAATCAGTTTTGATGGGCTTGAAGAAAAAATAAAGGAGATCTTCCTTGATATTTCTTGTTTGTTTGTGGGAGAGAAAGTTAATTATGTTAAGAGTGTGTTAAATACGTGTCATTTCAGCCTAGATTTTGGAATCATAGTTCTCATGGATCTTTCACTTATTACGGTTGAAAATGAAGAGGTTCAAATGCATGATTTAATTCGACAAATGGGCCAGAAAATAGTTAATGGTGAATCTTTTGAGCCCGGGAAAAGGAGTAGGTTGTGGTTGGTACATGATGTTTTGAAG

GTGTTTGCTGATAATTCGGTAAGTAACCCTTAGAAGAAGTATATTTAGTTTTTACTTATTTCCTAGACTTCATGATGGAAGATGTTTGTCAAATTAAGTTGTCAATTATGTTAAATTACTATATCGATGTCTTTGTAG

GGAACGATTGCAGTTAAAGCCATAAAGTTAGACTTGTCTAATCCCACGAGGCTAGACGTGGATTCACGAGCTTTTAGGAACATGAAGAATCTGAGGTTGCTTATTGTTCGAAATGCAAGATTTTCGACAAATGTTGAGTATCTACCTGATAACTTGAAGTGGATTAAGTGGCATGGCTTTTCTCATCGATTTTTGCCACTGTCCTTCCTTAAGAAAAATCTTGTAGGACTAGATTTGCGTCATAGCTTGATCAGAAATTTGGGCAAAGGATTTAAG

GTAATTATATATCTACCTGTATTTAGTTGGAAGCTTCTTCATTGATTTTCTTAGAAATTTTCATGGGTAGCTATAGCTTATTTTCTAACGATTTGCAAATATTTATTTTAAAACAACAGCTCGCAAATATAAGAGTTATGTTTATGTGAAAAATATTTGTTAATGTGGTATATATGCTTTAATTACTAAACCATTTACTTATGTAAATAGCAGTGGTTTGTCCTAAACCATAACTTTGATACTTTTCTCTTATTTTGATTTAATGTGTTATCTAATCATGTCCGATCAATATTTACTCCTTGTTTTTGTTTCTTTTCCATATATTTTAG

GATTGTAAAAGGTTGAAGCATGTTGATCTTAGTTACTCTTCTTTATTAGAGAAGATTCCCGACTTCCCTGCAACATCAAATCTTGAAGAATTATATCTTAACAACTGCACAAATTTAAGAACAATTCCTAAGTCAGTTGTTTCTCTTGGTAAGCTTCTTACTTTAGACCTTGATCATTGTTCAAACCTTATAAAGCTTCCAAGCTACCTCATGCTGAAGTCTCTTAAAGTTTTGAAGCTTGCTTACTGCAAAAAACTTGAGAAACTTCCAGACTTCTCTACAGCTTCAAACCTTGAAAAGTTGTACCTCAAAGAATGCACAAATTTAAGAATGATTCATGATTCTATTGGATCTCTGAGTAAGCTTGTTACCTTGGACCTTGGAAAATGCTCTAACCTTGAAAAGCTTCCAAGCTACCTCACATTAAAGTCTCTTGAATATTTGAATCTTGCTCATTGCAAAAAGCTTGAGGAAATTCCCGACTTCTCTTCTGCATTAAACCTTAAAAGCTTATATCTTGAACAATGCACAAATTTAAGAGTAATTCATGAGTCTATTGGATCTTTGAATAGTCTTGTTACCTTGGACCTTAGACAATGCACTAACCTTGAAAAGCTTCCAAGCTACCTCAAGTTGAAGTCTCTTAGACATTTCGAACTCTCTGGCTGCCACAAGCTCGAAATGTTTCCAAAAATTGCTGAAAACATGAAATCTTTAATTTCATTGCATTTGGATTCTACTGCCATAAGGGAGCTACCTTCATCAATTGGATACCTTACTGCGCTTTTGGTATTAAACCTTCACGGTTGCACAAATCTCATCTCCCTTCCTAGTACAATTTATTTGTTAAAGAGCCTTAAGCATCTTTATCTTGGTGGGTGTTCTAGATTTCAATTGTTTTCCCGTAAATGGGACCCAACCGCCCATCCACTATGCTCTTTTTCAAAAATTATGGATACTTCATCGAGTTCAGAATTTCCCCATTTACTTGTCCCAAAAGAAAGCTTATGTTCCAAGTTCACCATGTTGGATCTTCAATGTTGCAATATATCAAATGTAGATTTTTTGGAAATTTTATGTAATGTCGCCCCTTTCTTATCTAGTATACTTTTGTCGGAAAACAAATTCTCTAGTCTACCACCATGTCTTCATAAGTTTATGTCCTTGTGGAATCTCCAATTAAGGAATTGCAAGTTCCTTCAAGAAATTCCTAACCTCCCTCATTGTATACAAAAAATGGATGCCACTGGTTGCACATTGTTGGGTAGAAGTCCAGACAACATCATGGACATAATATCGAGCAAGCAG

GTTCCTCACTTCCATTTCCATTTTCCTTGTTCATATCATTTTATTGTCTCTCATTAAGATTCTGTTCACATTAATTTGTATGCCATAG

GACGTTGCACTCGGTGACTTTACAAGAGAGTTTATTCTAATGAATACTGGGATTCCAGAATGGTTCAGCTATCAGTCAATATCAAATTCAATAAGGGTTAGCTTTCGACACGATCTCAATATGGAACGAATTTTGGCTACATATGCTACTTTGCAAGTGGTTGGAGATTCATATCAAGGAATGGCCTTAGTTTCATGTAAAATATTCATTGGCTACAGACTCCAAAGTTGTTTTATGAGAAAATTTCCATCATCAACATCAGAATATACATGGTTAGTAACAACTTCTTCTCCAACATTTAGCACTTCCTTGGAGATGAATGAGTGGAATCATGTCACAGTCTGGTTTGAGGTTGTGAAATGTTCTGAGGCCACCGTAACTATAAAATGCTGTGGTGTCCATCTCACTGAAGAGGTCCATGGAATACAAAATGATGTCAAGGGGCCAGGGGTAGTTTATACAGTTTTCGATCAACTGGACAAATTACCGAGCCG

GTGAGTGAGATTATTGATTGTTTTAAGAAGGCTTGATTAGTTGTCCTGTTTTAGAAAATTTTTGCAACAACATTATTTTGTCTATTTGAAGATATGATTCATCTAAAGTTTTCTTTAACAAAGTTTATTTAAAATCTATTTCTATTAGCATATCCATAATTGTTTCTTGAAAAGGTTTAAGGTTCTTACTCTTAACCAATTGAAACAGAGCAGTTGTTAGTTTACAAAATTACTTGATATTATTTGTTTTATTTTGGTTTTGATTTTGTTGGTAGTATATTTGTTCTAAAACTTATTGCAATTGTTTTGGTTGTAATCAGTTGAATATTGTTTTGAATAAATTGTGGTGTTTCGTGTGTATGATGTTCACTATGGCACAG

GGATGTTATAAAATCCTTTGGTCAAGAAGTATCTGCCAAATCAGATTGCAATGCAATGTTGCATGCAGAAAATTTTCCAGTTTGGAACGATTCGAAAATGCAACAACATATGAATTTTCCCTTACATGTAACTTCTCAAGGTGTTACAAGGATACGTGGTATGGAAGGCATGGCAGAGACAACACTTGCCAACTCTATATGTAACAAATATGAAAGAAGTCGGAATCTTTTCTCTGCAAAAAAAGCTTTGAATCATTCTACTGGCTTTCTTTGTGGAGATGGAAATGGGCTTTCTTGGGAAATGGTAGACAGACCGATATTAAGTGATAGATTGTCTTCCCAAAAATATCTTAGAATTTTCGACGATCGTGATCGATATGGAGACCTAAATGATGTGGCTCATGGGACTGGTAATAGGTTTCGTTCAAGATTTTTAAGGATGGATGATATAAAAGAAGATGATATCAGAGAAGAGCCTTATTGGAAGTACATGGAAAGGTTATTCCAAACAGATCCTATATCATGA

>Cucsa.091840

ATGGGTTCTAACGCTGCTGGAGCGGAATCGTCGTCTTCTTCTCCAATCAATTGGATTTATGATGTGTTTTTGAGTTTTAGAGGAGAGGATACTCGCTCCAATTTCACAAGTCATCTTCACATGTTCTTGCGTCACAAGGGTGTCAATGTTTTCATAGATGACAGGATCGAAAGGGGTGAGCAAATTTCTGAAGCCCTTTTAAAAACTATACAGTGTTCTTTGATTTCTATTGTTATATTCTCGGAAAATTATGCATCTTCTACATGGTGTCTGGATGAATTGGTGGAAATAATTGAGTGTAAGAAATCCAAGGGTCAGAAAGTATTGCCAATTTTCTACAAGGTGGATCCTTCGGATGTACGAAAACAAAATGGTTGCTATGGAGAAGGATTGGCCAAACATGAGGCTAATTTCATGGAGAAGATTCCAATATGGAGGAATGCTCTAACGACTGCTGCCAACTTGGCTGGTTGGGATCTCGGAACAATAAG

GTACATATGTTTTTACAAGATTGAGATTTGCAAGATTTTTTTACCTTTATTTTTAAAATGTATAGTTGATTAATTCTCTTTCTTTATTGTATATGTGTTCTTTTTAGCGTTTTATAAATCTAAACAATTTTTTTTTCTTTAAAAAAAAGAGAGATCTCCAAGACTTATTACTCATAGACCAAAGTATAAGTCGTGGACTAGGTCCATGAGTTGTTTTGTTGTTTTTATTGTTGAACACATTGATGCAAACACGGATAAGAGATTTGAATTATGGAATAATGGGACCCCTACAAGTACTTGTACTCATGTCATTAAAATTTTTAAATTGTAAAAGCAGCAAATTTAAAAATCTATAATTTTATGATTATCATCTAATAGACGTCTGATATCACTAATTTTGATATATATATATTCGCAATATAAAAAAAAGTTTGTGCATTATTTTTTCTAAATATTTTTGTCATTTGATATAATTTTCCTATAGCAATTGTGGACTTGGTTCACTATTTTTCACACCTATTCTGTTAATACTTTTTTGCTTTTTCTGTATTTTTATCAATTATTTATATTTTCACTTTATTTAAGGACCCTATTTCTTGTAATTTATTTGGCCAATTACATTATTTTTGGAATTAGCTCTGTTTTTTATTCATTAGAAATTCTCACACTATATAATTTAAACTCAGAAGATTATTCTCTCTCAAGATGCAATTTAATTAATTATGTCCTCTGACTCTCTCATCATCTATTAATTTTGCAATGATTATGTACTAATTAACAACAG

AAATGAGGCTGACCTTATTCAAGTTATTGTTAAAGAAGTGTCGTCTACATTAAATGTCACCACGCCCTCAGATAAGCCTCTACTAGTTGGAATTGATTCCAAAATTGAATCCCTTTATTGGCCTACAGAAGAAATGTACAAGTCTGAATGTGTTGACATGTTGGGGATATATGGCATTCGAGGCATTGGTAAAACAACTTTGGCTAAAGCTTTATACTACAAAATGGCTAGCCAATTTGAATGTTGCTGCTTTCTATCAAATGTTAGAGAAGCTTCAAAGCAACTCAATGGCCTCGCTCAACTACAGAAAAAACTACTTTTTCAGATCTTAAAGTATGATTTGGAGGATGTCGATGATCTTGACAGGAGAAATAATATCATAAAGCATAGACTCCATTCGAAGAAAGTTCTTATACTTCTTGATGATGTGGATGAGATGAAGCAATTAAAAGCATTGGCTGGTGGGCATGATTGGTTTGGTCAGGGTAGTAAAATCATTGTGACGACTAGAGATAAACATTTACTTGATAGCCATGGATTTGGTCAAACATATGAAGTTGAAGGATTGTGGGAACACAATGCATTTGAGCTTTTTTGTTGGCATGCCTTCAAAAAAAGTCATCCATCTAGTAATTATTTAGACCTTTCAGAACGTGCTACAAGGCATTGTAAAGGTCATCCTTTGGCTCTTGTTGTTTTGGCTTCTTTCCTTTGTGGCAGAGATCAAGCAGAATGGAGTGGTCTATTAGATGGATTTGAAAACTCTTTGAGAAAAGGTATTAAAGATGTTCTTCAATTAAGTTTTGATGGGCTGGAAGACGAAGTAAAGAAATTTTTTCTTGATATTTCTTGTTTACTCGTGGGTGAGACAGTTACCTATGTTAAGAAAATGTTGAGTGAATTCCATTCGATTCTGGATTTCAAAATTAGCAATCTGAGGCATCTTTCACTTATTAGGATGGAAGAATATGATGATGATAGGGTGCAAATGCATGATTTAATAAAACAAATGGGTCATAAAATAGTTTATGATGAATGTGGTGATGAGCCTGGAAAAAGGAGTAGATCAGGTTGGAGGAGGACATTCTGGAG

GTGTTTAGTAACAATTCAGTAAGTTGGTGTTACCTGAATTAATTGTCTTTAATTTAAGTAATTAATTTTCCAAGACTTTCAGATGATGAAAAATGTTTGTGAAAGTAATGTTAAATTATACAAAAATGTATGACTTCAATTTGTAG

GGAAGCGATGCAGTGAAAGGCATAAAGTTGGTGTTTATCTGATCCCACCAAGGGTCATAAACGTGAATCCAGAGGCATTTAGAAGCATGAAGAATTTGAGAATACTGATTGTTGATGGAAATGTGAGGTTTTGCAAAAAAAAAAAGTATGTACCGAATGGGTTAAAGTGGATAAAATGGCATAGATTTCCTCATCAAACTTTACCCTCATGCTTTATTACAAAAGATCTGGTTGGACTACATTTGCAACATAGCCTCATCACTAATTTCGGAAAAGAACTGTATCTTAACAACTGCCCAAATTTAAAAACCATTCCTAAGTCATTTCTTTCTCTTCCTAACCTTCTTACCTTCAACCTCCACCATTGTTTAAACCTTAAAAAGATTCCAAGTAGCTACATTTCATGGGAGCTAGTGTCTTGAAAATTTTGATCTTTCTCACTGCCAAAAGCTTGAAAAAATTCCTGACTTCTCTTCTGCATTAAACCTTAAAAGCTTGTCTCTAATACAATGCACAACCTTGAAAAGCTTCCAAGCTACCTCAAGTTAAAGTCTCTTACAGATTTAGATCTCTCTGGTTGTCGTAAGCTCGAAACGTTTCCAGAAATTGATGAAAACATGAAATCCTTAGAAAGGTTGAGGTTGTCTTATACGGCCATAAGGAAGCTACCTTCGTCAATTGAACACCTTACTAATCTTCAATATATTAAATCTTAAACGTTGCACAAACCTCATCTTCCTTCCTTGTACAGCTTATAATTTGTTTAAGAGTCTTTGCGGGCTTAGTCTTTCTGAGTGTTCTATTGAAATGCGTTCCAAAAGTTATAAAAACTATGAAAATTATGAAAACTTCTTCGACTTGGGAGTTTTTCCATTCACGAGTTCCAAAAGAAATCTTATTTTTCGAACATTTGGAGTTGCTGGATCTTAAAGGTTGCAATATATCAAATGTTGATTTTCTGGAAAATTTATGTAATGTAGCTCTATCCTTAACTAGTATAGTCTTGTCAGAAAACGAATTCTGTAGTCTACCTTCATGTCTCCATAAGTTTATGTCCTTGCGGAATCTCCAATTAAGGAATTGCATGTTTCTTCAAGAAATTCCAAACCTCCCTCAGAGTATACAAATAGTAGATGCCACTGGTTGCATATCGTTGAGAAGAAGTCCAAACATTATGTGGACATAATATCAGGCGGGCAG

GTTCATCTCTTACCAATTCATTTTCTTTGTTCATATCAAGTTCTTGTCTTTTATACTAACATTCCATGCATAATATATATATATAATTCTTATTCCATAG

GTCAATGTTAAATGGAGAAGGCGTTGTTATATCTTCAGGGAATTCGTTCTAATGAATAGTGGGATTCCGGAATGGTTGAGCTATGAGACTGCATCAAATTCAATAAGGGCCAGTTTTCAACACAATCGCAATAGAAAGATAACTTTGGCTACATCCGTTACTTTCCGAGTGGATATTGGAGATTCATATCAAGGAATGGCCTTAGTTTCATGTAACATATTCATCGGTTGTAGACTCGAAAGTTTTCATATGAGAAAATTTCCAACATCAACATTAGAATATACATGGTTAGTAGAAGCTATTTCTACAAGACAAAGTGAGTCCTTGGAAGAGAATGATTGGAATGATGTTATAGTCTTGTTTGAGGCTGTGAAATGGCTACTATTGCCTACTATAATTTTCACTTTATTTATAAACTTTATTGCCTACTATAATTTTCCAAAATTTATTTATATTAAAGTACAAACCTTTAATATATAGAATCAATGAGCAAATTATTGTAATATTGTTTCTTGAGTGAATTATGAGGTTAAGATACTTTATAGAATTGACGAAATTTTAATTAATTAATTAATTACATTAAACTAAATTAAATTTTTCAATGATCAATAAAAGAGTAATTTACGTTAACGTAACAACACCTAAAAATAATTACACCGTGTTGTAACAAAAAAAAATAAGAGCATATAAAGCCACCAATGTTTTTTCACAAATTTCAGATTTGTTCTTTAATATTGCGAACCATTCGTCTTCTCTTTTTTCTTTTCTGTTATTTATTCGTCTCCTCTACCTGATTTTCTTTCTTATATTGGTAAACAATTTATTCTTCCTTTAAATCTTCTATTTCATATTCATCATATTCGACAAGATTTTTTAAAGCTAACGATTGTTTCAATATT

>Cucsa.178450

ATGAATCAAGCAAGTGGGTCATCTTCTTCCTCACGTTTTAGATGGCATTTCGATGTATTTTTAAGCTTTCGAGGGGAAGACACTCGATCCAACTTCACTAGTCATCTTAATATGGCTTTGCGTCAAAGAGGAATCAACGTTTTTATAGATAACAAACTTTCAAGGGGTGAAGAAATTTCTGCATCTCTTTTGGAAGCTATTGAAGGATCCAAGATCTCCATTGTCATAATCTCTGAAAATTATGCTTCTTCCAGGTGGTGTTTGAATGAGCTGGTGAAAATCATTATGTGTAACAAATTGAGAGGACAAGTGGTTTTACCAATTTTCTACAAAGTGGATCCATCTGAAGTAAGAAAACAAAGTGGAAAATTTGGAGAAGAATTTGCCAAACTTGAAGTTAGATTCTCGTCGGAGAAGATGCAAGCATGGAGGGAGGCCATGATTTCTGTTTCTCATATGTCTGGATGGCCGGTTCCTAAGAAAGA

GTATTTTCCTTTTCCATATATATATTTTTACTCTTTTGCTCTTCATATTCTATTCTTACTGTATACTTCTAACTATCCCAAGGTGAGAGCATGCTGGACTGAGGGAAAATGCAAAGAAAATGTGACATTTAAGTTCGGTTCGATAACAAGTTTATTTTCGAGTTTTTCATTTTTGAAATTTATATAGTCGTGTTATAGAACTACCAATTTTTTAATTATTGTGAGTTTGAACTTTATTAAGGAAACATTTGAATTTTTAACTTGTACAAAAAAAAAACAAGATTTGAAAATTATATATGTGTGTGAATTGTTTTAATTAAATGATATAATTGTTGAAAATAATTACATTATAACAAAATTTGTAGTAATAGGAATTAAAGTGAATTCCAAATCAATTTTTAGGGTATGTTTTTTTCTTAAATGACAG

TGACGAGGCCAATTTGATTCAAAGAATTGTTCAAGAAGTCTGGAAGAAATTAAATCGTGGAACAAGAGAGATGCGTGTACCTAAATATCCAGTTGGAATAGATAGACAAGTTAATAATATACTCTCCCAAGTTATGTCTGATGAAATAATTACTATGGTTGGATTATATGGAATTGGAGGTATTGGCAAGACAACTTTGGCCAAAGCTTTATACAATAAAATTGCTGATGACTTTGAAGGTTGTTGCTTTTTGATAAATGTTAGAGAAGCTTCAAATCAATATCGGGGTCTTGTTGAACTCCAAAAGGAGCTACTTCGTGAGATTCTAATGGATGATTCAATCAAAGTTAGCAATCTCGATATAGGAATTAGCATCATAAGGGATCGACTATGCTCAAGAAAGATTCTTTTGATTCTTGATGATGTTGATACGAGTGAACAACTAGAAGCATTAGCAGGAGGACATGATTGGTTTGGACCGGGAAGTGTGGTCATTGCGACAACAAGAAACAAACACTTACTTGCTATTAATGAATTTGATATATTGCAAAGTGTTCAGGGATTGAATGATGATGAAGCCTTCGAGCTTTTTAGCTGGCATGCTTTTAAGATGAGTTGTCCATCAAGTCATTATTTATACCTAATTTCAAAACGTGCCGTAAGTTATTGTAAAGGTCTTCCCTTGGCTTTGGAAGTTGTAGGTTCATTCCTTTATTCTATTGAGCCATCCAAGCTTAAACTTATATTGGATGAATATGAAAACCAATACCTTGACAAGGGCATCCAAGATCCTCTTCGAATAAGTTATGATGGACTTGAAGATGAAGTAAAAGAAATTTTTCTTTATATTTCTTGTTGCTTTGTAGGAGAAGACATCAACAAAGTTAAAATGAAGTTAGAAGCATGTGGTTGTTTATGTTTGGAAAAAGGAACAACAAAACTCATGAATCTATCACTTCTGACCATTGATAAATCCAATCGGGTTGAAATGCATAATTTAATACAACATATGGGTCGCACAATTCATCTTTCGAAGACTTCTACATCTCATAAAAGAAAAAGATTGTTGATTAAAGATGATGCTATGGATGTCTTAAATGGGAATAAG

GTAAGAATTATCGGTCAAACTTTGATTATGAGAAGAAGTTTTATTATAATTTGAAAGTTAATGACTTGAAACGTTTGTTTTGTAACCTTTAAATTTGTAGGAAGCAAAAGGAGTTAAAGCCATAAAATTAAGTTTTCCTAAAGCTACGGAG

TTGGACATTGATTCAAGAGCTTTTGAAAAAGTGAAAAATGTGGTAGTACTCGAAGTTGGCAATGTCACATCTTCAAAAGGTACTGATCTTGAGTATCTACCTAGTAGCTTAAGGTGGATGAATTGGCCTCATTTTCCTTTTCCATCTTTGCCTACAACCTACACAATGGAGAACCTTATGGAATTGAAATTGCCATATAGCTCCATCAAACATTTTGGAAGAGGATTCATG

GTATTAATTTATGTCAATAATTGAGTTATATTAATTTAACTTCAATGTCAGCTTATTTATTTCAACTTTTATTATATATTCCTTTTATATTAATTGCAG

AGTGGTGAACGGTTGAAGGAAATTGATCTTAGTGGCTCTGAGTTTTTAGTGGAAATTGCTGATTTATCTACTGCAACAAACCTTGAAAAGTTGAATCTTTTAGGGTGTGTAAATTTAGTAAAAGTTCATGATTCTGTGGGATCTCTCACTAAGCTTGTTACATTTTCTCTTTCTAGCAATGTTAAGGGCTTTGAGCAGTTTCCACCCCACCTCAAGTTGAAATCCCTCAAACTTTTGTCAATGGAAAATTGTAGAATAGATGAATGGTGTCCTCAATTTAGCGAAGAAATGAAGTCTAGCCTAGAAGAATTGTTGATTCAATATAGTACTGTAATTAATCAGCTATCTCCAACAATTGGATATCTTACTAGCCTAAAACGTTTGTTTATCATAGAGTGCATGAAGCTCAAAACTCTTCCAAGTACAATTTATCGTTTAAGGAATCTTACTTTTTTAAGTGTCATTAAATCTGATCTATCAACCTTTCCTTCCTTAAATAATCCTTCTTCACCTTCCTTATTTCCCTACCTAACATCAATATACCTTTACAATTGTAAGATAACAAATTTGGATTTCTTAGAAACAATGGTTCATGTTGCCCCTGCATTGAAACTGTTGGACTTATCTGGAAACAATTTTTGTAGACTACCCTCATGTATTATTAATTTTAAATCCTTGAAATCTCTTGTTACAATGGAATGCAAGTTGCTTGAAGAAATTCCAAAGGTTCCAAAAGGAGTAGTTCGTATGAATGCTACAGGGTGTATATCATTAACCAGATTTCCTGACAACATACCTGACTTCATATGCTGCGATGATAATGTGGTGC

GTATCATTGTTCTTTCTCATGACCTCATGATCTCTCGTGTATTTCGTTCATATAAGAATTAAAGTTTATGTTCTTTGTTAATTAATATAACTTTTAGCTATTTACTCTCATCTTGCAGGAATATGATAAAGAACACCCAG

TAATCAAACAACTCATTTTAATGAATTGTGATATTCCAGATTGGTTCAGTTACAAGAGTATGAACAATTCAGTAACGTTTCTTTTTCCACTTATTGATTATCTAAGTTGGAAAAGGAAGGCTTTTATTACTCCCTGCGTCAAATTTCAAGTTTCTATCGATCAACCAGTTGAGTTTAAATGTAGAGTGTTTATCAACGATATTGAAGTAAAATATGATCGACGAAAGTGTTCAATTTTTAGTTTGGAGTTATTGCTTAAAA

>Cucsa.249360

CACCATAAGAAAGTTGGCCTATTTCGACCATCTTTAATCAATGGTTATAAAAATTCTTAAAAATGATATACATTCGTTGGAGGTTAAAAAAAACCTTCGTGAATTATATACTTTTTTTATCAAAGAATAATAAAAACCTTTAAAAAAGATATATATTTTATCGAGGGTGTTGGAATTGGCTTTTGCTTCTAGTTTGCCTGGTTGGGATATTGCAAATAGGTATTTCTTTCTTTAGTCTTCAAAGACTCATTGTCCAAGTGAAGTTTAAATTTATCTACTATTTCCTATTTTCACTTTTTTGTATGATGTTACTTTGTGACAACAAGGATGAGGCTAAACTCATCCTAAAAATTGTTAAATGAATGTTGTCTGTAGTAAATCTAAAGCGATTACTACGAGTAGCGAAACATCTAGTCGAAATTGATTCTCAACTAAGAGAAATTGAGGAGTCGGTCTCTCATATTGGGTCCGAGGGTGTTAACATGGTGGGGATATGTATGGCATTGGAGGAATTGGTAAGACCACTTTGGCTAAGGATTTGTACAACAAAATTGCTACCCAATTTGAAAGATGCTGCTTTCTACAAGATGTTAGACGAGAAGCTTCAAAGCAATATGGGCTCGTTCAACTACATGAAACCTTACTCTGTGAGATTTTAAAGGAGGATTTGAAGGTTGTCAATTGTGATAAAGGAATTAACATCATAAGAAGTAGACTGTGTTTAAAGAAAGTTCTTATAGTTTTTGATGATGTGGATCATCACAGGCAATTAGAAGCACTAGTTGGTGAGCTCGATTGGTTTGGTCGAGGTAGTAAAATCATTATGTTGACAAGGAATGGACATTTACTTTCTAGCCATGGATTCGATGAAAAGCATAAAATTCATGAATTGGATCAAGACCATGCTCTTGTTCTTTTTAGTTGGCATGCTTTAAAAAAAATCATCCATCAAGTAGTTATATAGGCCTTTCAGAAAGTGCTACAAATTATTGTAAAGGTCTCTCTGTGGCACTCGTTGTTTTGGGTTCTTTCCTTCGTGGTAGAGATCAAACAAGATGGAGTTGTATATTAGATGAATTTGAAAACTCTCTACCAAAAGATATTAAAGATGTTCTTCAATTAAGTTTTGATGGACTAGAAGACAAAGCAAATGATATTTTTCTTGATATTTCTTGTTTATTTGTGGGAGAAGATTACAATTGTGCTTTAAAAAAAAATGTTGAGTGCATGTCATTCGACCGTTAGATTTTGGAATTATGATTCTCATGGATCTTTCACTTATTATGATTGAAAGTGATAGAGTGCAAATGCATGGATTAATACAACAAATGGGTTGTAGCATAGTTCGTAATGAATCATCTCAACCTGAAAAGAGGAGTAGGTTGTGGTTGGTTCAGGATATTGGGGAGGTATTCGTTAATAAGTCTGTGAGAAACTCTTTACCTAAAGTATTGATAGTTTACTTATTTTTAGGACTTCACATTATAAGAAATATTTGTTACAAAGTTGGACTTGCCTAATCCCACATGACTAAATGTAGATCCACTAGTATTTAAAAGCATGAAAAATTTGAGATTGCTTATCATTCGAAATGCACAATTTAGTACAAAGATTAAGTACCTACCTGATAGCTTAAAGTGGATTGAGTGGCATTGATTTGCTCATCGAACTTTGCCGTCCTGCTTCATTACCAAAAATCTTGTTGGACTTGATTTGCGACATAGCTTCATCAAAAGATTTGGGAGAAGACTTCAAGGTAAAGTATATTTCTTTGGTATGTGTATTTGATTGGAGATTTCTTCATTGTTTTTCTTAAAAACTTTTATCATTTTTTTTAAATCACAAACATATGATTAAACAAAATTTATTAATGATAGACGACTGATCTATCGCACAACAATAGACTCCTACTAGTCATATGATCTATTATTAACAAATTCATATTAGTGATATGGCCTATCGTGGAACTAAATTTTTCTATATTTATAATTTTTTGCATTGTAATATCTCTATTATCACTTTTGAGTCTAGACTATAGATGATATATTGGAATTATATATGCAATTATCATAATGTAACATAATATATTGGATATATTTTTTTTTATGTTACTAGTATTAAACCCGTGCATGAAATACAATTTTATTAGTAGCATTTAAAGTTTGAAAGTGAATATATTCTAAAAGAGATTATTTGCTCACCAACTAATGTTTGATGGCAAACTTTTTGTTTTTAGTTATTGAGTAGATGGACAAATTAAGCTATGTTTGATCCCACCTTTTGTTTTTCTTTCCAACGCTTCCATATCTCTAAAATCGAACTACCAACAATATTGTTGAACATCAAACTTGCCAACCCTTTCATATCTCTAAAATCCAACTACCTTGTTCAACATTAAACTTATCATGACTTGTTGTGAGCATAAAAGAGAACTTGTGCATATATAGACAGAATTAGAAATGTAGTTAAGGAAGAAATAAATGATAGAGAAACTAATGTTACGAAAAGCTTTTCAACTTCCAAATCTTTAAATTTGTATGGTTAATTAGATTTAATAAGATATTTAATTTTTAATTAATTTTTAAAATGTAAATGAGCTTTTCACATTCATTCTTTAACTATAATTAATTGAAAAAGTAGTGTGAGACTTCTACTATAATTAATTATATTAAAAAAAACAAAACTAAAAATGAATTTTATCATAAAACCAAAGAGTGGTTTGGAAATAAAACTAATTGAAAAAGTTTTTATGTTTTAGTTTGAATTAGTACCTAAAAAGTTTCTCCACGTTAAACATTAAAGCTTTTATATATACTATAGATGATATATAGAAGATTATATTGTTCAATTATATCTTTTCCGTTACGTTTTTTTATTTTTTATTTTAAATTTAATTTAAGATCAAATAAAACTAATTAAAAATTGAAACCAAATTTCCACCCTAATTAAATTGGCTAAACTACATTCTCAATCTTTTTAAGGTATAAAATTAAATGGTTAGTTTACAATCTACTAAATTTAAGATAATATATTTTAGTTATAATGATTACTTTACAATAATAATAATTAATTAATAAATTACTTATAAACTAAAACTTAATTCAAAACAAAATTTAATTTAAACTAATTATTCTCTGCATAAATTACATTTTTTAAGCATTTCAAACTTATAAAATACATCCAATACACATAGTATCAAACTAATTGAAGTAAAAAGTGTGTTGTTTGTCAATATGAGCGTAGCACAACTGACTTGATGTTTGTACTATCAATTTCAAGATTAGAGGTTTGATTATTTAATTACAATATCTTTTAAGAATTAGTCTTATGGAATAGTGCCAAATAAATAGATGATTAATCTTGATATGGATTGAGTTGATCACTGATTTACTTTTGTATATGGAATTTTATAATTAGTTTGCCTAAATATATTGATTAATGGTTATTATTAAAAAATTTAACCTATATTTTATATTTTTCTATATTTACAATTATTCTTTTAGCCACTATTTTATCTAAATTTTGTTTGGTATGCCAATTATATTACATACCCTTTTTCCTTTTGGTTTCTTTTTCAATGTGTTTTAGGATTGTGAAAGTTGAAGCATGTTGATCTTAGCTACTCTACTTTATTAGAGAAAATTCTGGATTTCTCTGCTGCATCAAACCTTGAAGAATTGTATCTCACCAATTGCACAAATTTAGGAATGCTAGATAAGTCTATTTTATCTCTCAATAAGCTTACTGTCCTAAACTTTGAAGGTTGTTCTAACCTTAAAATGCTTTCAAGAGGCTACTTCATGTTAAGTTCTCTTAAAGAATTGAGGCCCTCTTACTATAAGAAGCTTGAGAAAATTCCAGACTTATCTGCAGCATCAAACCTTAAGAGATTGTATCTCCAAGAATGCACAAATTTAAGAGTGATTCATAAATCTGTTGGATCTTTGGATAAGCTTGGATTGTTGGACCTTAGTCAATGTACTAACCTAGTAAAGCTTCCAAGCTATCTCAGGTTAAAGTCTCTATACACTTTATATCTTTCTGGGTGTTGTAAGCTTGAAAGCTTCCCAACAATTGCTGAAAACATGAAATATTTAGAGGAATTGTATTTGAATTTTACTGCCATTTAGGAGTTACCTTCATCCATTGAATATCTTACTAACCTCTATAGATTAAACCTTACCGGTTGCACAAACCTCACCTCCCTTCCCAATACATTTATTTGTTAAGGAGTCTTGAGGATCTTCCTCTTAGTGGCTGTTCTATATTTGAAATGTTTCCCCATAAATGGGATCCAACCATCCACCCAGTATGCTCTCCTCCAAAAATGATGGAAGCATCTTCGTGGAGCTTGGAATTTCCCCATTTGCTAGTAGCAGTTGAAAGTTTACGTTCCCGTTTCACTTTGTTGGATCTTCAATCTTGCAACATATCAAATGCAAATTTTTTGGAAATTTTATGTGATGTTGCCCTCTCTTATCTGATATACGCTTGTCCAAAAACAA

>Cucsa.275630

ATGGGTTCTTCTTTGGTTGGTTTAGCACAATCATCATCGTCTTGTTCTTCAAATTTGAAATGGAGTTATGATGTGTTTTTGAGTTTCAGAGGTGAGGATACTCGAAACAACTTCACTAGTCATCTTGACAGGGCCTTGCGTGAAAAGGGTGTCAATTTCTTCATAGATGACAAGCTAGAGAGGGGTGGTCAAATTTCTGAATCCCTTCTCAAATCTATTGATGGTTCTAAAATTTCCATCATTATTTTCTCCAAAAATTATGCATCTTCCACCTGGTGTTTGGATGAACTGGTGAAAATAGTTCAGTGCATGAAATCCATGGGACATATAGTTTTTCCTGTCTTCTACAAG

GTTTAATTTGTTGGACTATCTGAATTCGTATTTTTTATATAGTTATGTTATCTATGATTTTGACCCTAACGTTTTAGAGAAGCCCACTAACACTCCAACCATAGAAGCACATTTGATGTGTAATATGAATTTTCTTTCTAATAAAATTGCTCCCTCCCTCTACCTGTGGAGTTAGCCACCACTGATAATAAATCATATAAATCTGTATCATAGTCTCCATTGCTTTACGGGTTTATCTTTATTAATTTGTTGATTAATTTCTTAACAAG

GTGGATCCATCTGAGGTTCGAAAACAAACTGGTGGGTTTGGTGAAGCATTGGCCAAACATGAAGCTAATGAGTTAATGACCAACAAGGTTCAACCATGGAAGGAGGCTTTGACCACTGCTGCTTCTTTGTCTGGTTGGGATTTAGCAACTAG

GTATTTTTTACATGCATTATTCTAAAAGGTAGTGAATTTATTTTAGGAGTGAAATTATATTTTACCCTTTAGATTACAAAAAAATATCAAATACATTAAGAAAAAAAACGACTTTTAAGAAAACAGATTGATTGGTATCATTGTTTTTAGTGTTTTTAGTTTTTAAGTTAGTTTTAACAGTTTTCAAGGTTAACGATGAGATGAATTAAAATTACCTTGTTCAAGGTAAACAATTCATTTTATGCAAGTTGATTCCTCAAATAGTTAAAAAAAAAAAAGTGGTTAGTTTTCTATCATAAATAAGCGTGTATAACAATTTTACCTCCAAACTTCTTGCTTTGTTATCAACTTTTTCCAGTCATTTAAAAATCAAGCCAAAATTTGAAAACTAAATAAAGTAGATTTTAAAAACTTATTTTTGTTTTTAAAACTTCGATAATAATATTCAACCATTGCGATAAGAGATGCAATAATAATAAAATATTGGAAGAAGATAGGCTTAATTTTCAAAAACCTAAAACAAAAAACACAGAATGAAAACCTCAAACAAAAAACACAAAATGGATATTGAAAGAGAAATCTGTGAGTGCCATGTTGACCAATCTAATTTTGTTATTGAAAAATTTAATGTTATAATGAATTTAACTTCATCTACTATGTTTCTTAATTCTTATTATTGTTCCCTACTATGTTTCTTAACTTCGTCTACTTGTATTTGTGTATGATGTGCTCAATCATTACAG

GAAGAATGAGGCTGATCTTATTCATGACCTTGTTAAGGAGGTGTTGTCTATATTAAATCAAACACAACTACTACATGTAGCCAAGCATCCAGTTGGAATTGATTCTCAACTTAGAGCTGTTGAGGAATTGGCCTCCCATGATGTGCCCGATGGTGTTAACATGGTGGGGATACATGGGATGGGAGGCATTGGTAAGACCACTCTGGCCAAAGCTTTATACAACAAAATCGCTTATCAATTTGAAGCTTGTTGCTTTCTTTCGAATGTTAGAGAAACCTTAGAGCAATTCAAAGACCTGGTTCAACTACAAGAAAAACTACTCAGTGAGATCTTAAAAGATAATGCTTGGAAGGTGGGCAACGTTCATAAAGGAAAGAATATCATTAGGGATCGGTTATGCTCAAAGAAAGTTCTTATCATTCTTGATGATGTGGATAAGGATGAACAATTAGACGCACTAGTTGGTGAACGTGATTGGTTCGGTCGAGGAAGTAAAATCATAGCAACAACAAGAGATCGACATTTACTAGAAAACCATTCATTTGATATAGTATATCCTATTCAGTTGTTGGATCCTAAGAAATCCCTTGAGCTTTTTAGCCTGCATGCTTTTAAGCAAAATCATCCCTCAAGTAATTATGTAGACCTTTCAAAATTTGCTGTAAGTTATTGCAAAGGTCTTCCATTGGCTCTTGTTATTTTGGGTTCTCTTCTCCATAAGAGAGAGCGAAAAATATGGAAAAGTAAATTACATGAACTTGAAAATTCCCTCGAACCAAGTGTTGAAGCTGTTTTTCAAATAGGTTTTAAGGAGCTTCACGAAAGAGTGAAGGAGATTTTTCTTGATATTTCTTGCTTTTTCGTGGGAGAGGATATTAACTACAGTAAGGATGTGTTAAAGGCATGTGATCTCAATCCAGACTATGGAATTATAATTCTTATGGATCTTTCCCTTGTTACTGTTGAAGATGGAAAGATACAAATGCATGATTTAATACAACAAATGGGTCAAACAATTGTTCGCCATGAATCTTTTGAGCCTGCAAAAAGGAGTAGGTTGTGGGAGGCAGAAGGAGCTATCAAGATATTGAAAGAGAAATCT

GTGAGTGACTTTAGACAATGTTTTTATTTACTTATTGCCAAAGATATATACTGTGATGAAATTAAAACATAATGCAAGTCAAATTGGTCGGTAATTATTTGTTAATAATGTAAAATTACTTGATTTATAACCTTGTAG

GGAACTAAAGCAGTTAAAGCCATAAAGTTAGACTTGCACTACAAACCTTGGCTGAAAATTGTTGAAGCAGAAGCATTTAGAAACATGAAAAATCTTAGATTGCTTATCCTTCAAAGAGTAGCATACTTCCCTAGAAATATATTTGAGTATTTACCTAATTCGTTGAAGTGGATTGAGTGGTCTACATTTTATGTTAACCAGTCTTCGTCCATAAGTTTTTCTGTGAAAGGTCGGCTTGTCGGACTAGTTATGAAAGGTGTAGTCAACAAACAGCCAAGGATTGCATTTGAG

GTAAATTTTAGTACTGCTTCTACTTGTGTGTGATTTTTTGGAAGTTTATTTAATGATTTTTTTTCCCATTTGTTTTAG

AATTGTAAAACAATGAAGCATGTTGATCTGAGTTATTGTGGCACGTTAAAGGAAACTCCCAACTTCTTTGCGACATTAAACCTTGAGAAATTATATCTTAGGGGATGCACGAGTTTGAAAGTGATTCACGAGTCTGTGGCTTCTCTTAGTAAGCTTGTTACATTGGACCTTGAAGGTTGTGACAACCTAGAAAGGTTTCCAAGCAGCTATCTCATGTTAAAATCTCTTGAAGTTTTGAATCTTAGTAGGTGCAGAAAAATTGAAGAAATTCCTGACTTGTCTGCATCTTCAAACCTTAAGGAACTATATCTCAGGGAATGCGACCGTTTGAGAATAATTCACGACTCTATTGGTCGTTCTCTTGATAAGCTTATTATCTTGGATCTGGAAGGCTGTAAAAACCTTGAAAGGCTACCAATTTACACCAACAAGTTAGAGTCTCTTGAACTTTTGAATCTCGCTTCATGTCTAAAGCTTGAAACTTTTTTTGACAGCTCTTTTAGAAAGTTTCCAAGCCACCTGAAGTTCAAATCTCTTAAAGTTCTGAATCTACGGGATTGTCTAAATCTTGAAGAAATTACTGACTTTTCAATGGCATCAAACCTTGAGATATTAGATCTCAATACTTGCTTCTCTTTAAGAATAATTCACGAGTCTATTGGGTCTCTTGATAAACTTATCACCTTACAACTCGATTTATGCCATAACCTAGAAAAGCTTCCTAGCAGCCTGAAGTTGAAGTCTCTTGATTCTTTGAGTTTCACTAATTGTTACAAGCTTGAACAACTTCCAGAATTTGATGAAAACATGAAATCTTTAAGGGTGATGAATTTGAACGGTACAGCCATAAGGGTGTTACCTTCATCAATTGGATATCTTATTGGGCTCGAGAATTTAAACCTTAATGATTGTGCAAACCTGACTGCCCTTCCAAATGAAATTCATTGGCTAAAAAGTCTCGAGGAACTTCATCTTCGCGGGTGTTCTAAACTCGACATGTTTCCCCCGAGATCAAGCTTAAATTTTTCCCAAGAAAGCTCATATTTCAAGCTGACGGTATTGGATCTCAAAAATTGTAATATATCAAATTCTGATTTCCTCGAAACATTATCTAATGTCTGCACTTCCTTGGAGAAGCTAAATTTGTCAGGAAACAAATTCTCTTGTCTACCCTCTCTCCAAAATTTTAAGTCATTAAGGTTTCTTGAATTAAGGAATTGCAAGTTTCTTCAAAATATAATAAAGCTTCCCCATCATTTAGCTCGGGTGAATGCCAGTGGTAGCGAATTGTTGGCTATACGTCCTGATTGCATTGCTGATATGATGTTCGGAAAACAG

GTTCCTCTCCTTTCATCTAATTTCTATTTGTTCATATCATAATATCTAATGTTATATATGTTCTCTTATTTAATGCCAATTAACTCTTAATCCCTTAG

GACGCTGAATTTAGTGACTCAACAAAAGTGCTCTTCATAACAAACAATGAGATTCCAAAATACTGCAACAAACAAACTACGAGAAGTTCAATGAGTGTTAGGTTTCGTCACAATTTAGATAAGAACATACCAGCTTTGGTTCTGTGTGTAATTTTCAAAGCAGATGGAGATTCATGTGACGAAGCGGAGGGTTTTATTCATTTTGAAGTGTCAATCGACGGTGAAATAATAATGGCTTCTACTGTGGGATGTTGTTGGTCCTCAAAATCAGAACATATGTTGTTACTAAGAACTTCTCCAACAAAATTAAGATACTTGCACGCCAATGATCGACACCACATCAAAGTCTTGTTTCCCAACACAACATCAAAATTTGTATCGAAAAGATTTAAAAGTGCAAATGTTATTATGAGAACCCAAGGAGTCTATATGGTTGATAGACGGTTTTCATGCTATACATAA

>Cucsa.292710

AAGAATAATGAAGCTCATCTTATTCAAGACCTTGTTAAGAAGGTGTCTATATTAAAACAAACACAATTACTAAATGTAGCCAAGCATCCTGTTGCAATCGATTCTCAACTTAAAGCTATTGAGGAATTAGCCTCCCATGGTGTGTCAGATAATGGTGTTAACATGGTGGGGATACATGGGATGGGAGGCATTGGTAAGACAACTTTGGCCAAAGCTTTATACAACAAAATCACTTATCAATTTGAAGCTTGTTGCTTTCTTTCAAATGTTAGAGAAACTTCAGAGCAATTCAACGGCCTAGTTCAACTGCAAGAAAAATTACTCAATGAGATCTTCAAGGATAATAACTTGAAGGTTGACAATGTGGACAAAGGAATGAATATCATAAAGGATCGATTGTGCTCAAGGAAAGTTCTTATGGTTTTGGATGACGTGGATAAGGACGATCAACTAGATGCATTGGTAGGTGGACGTGATTGGTTCGGTCGAGGTAGCAAAATCATTGTGACAACAAGAGATAGACATTTACTCGAAACATATTCATTTGATAAAATACATCCTATTCAATTGTTGGATTGTGATAAATCTCTTGAGCTTTTTTGTTGGCATGCTTTTAAGCAAAGCCATCCATCAAGGAATTATTCGGAACTTCCAGAACTAGTACGTTATTGCAACGGTCTTCCTCTAGCTCTTGTTATTTTGGGCTCTCTCCTTTGTAAGAGAGATCAAATAATATGGAAAAGCAAATTAGATGAACTTAAAAACTTCCCCGAACCAGGTATTGAAGCTGTTTTCCAGATAAGTTTTAAGAGGCTTCCAGAAAACCCCCCAGTAAAGGAAATTTTCCTTGATATTTGTTGTTTTTTTGTGGGAGAGGATGTTAGCTATAGTAAGAATGTGTTAAAGGCATGTGATCCTTATCTAGAATCAAGAATTATAATTCTCATGGATCTTTCTCTTGTTACGGTTGAAGACGGCAAGATACAAATGCATGATTTAATTCGACAAATGGGTCAGATGATTGTACGTCGTAAATCTTTTAAGCCAGAAAAAAGGAGTAGGCTGTGGGTGGCAAAAGAAGCTGTCAAGATGTTGATAGAAAAATCA

GTGAGTAACCTTATGTAAATTATTTTCGTTTACTTATTTCCAAAGACATTGTGATAAAAAAAATGGGTCAGCTTGGTTACTTGTTAATAATGTTAAAATGACTTCATATTTAACTTTGTAG

GGAACTCATAAAGTTAAAGCCATAAAGCTAGACTTGCGCAACAACGGTTCACTGATTGTTGAAGCAGAAGCATTTAGAAACATGGAAAATCTTAGATTGCTTATTCTTCAAAATGCAGCAAAATTGCCTACAAATATATTCAAGTATTTACCTAATATTAAGTGGATTGAGTACTCATCATCTAGTGTTCGATGGTATTTCCCTATAAGCTTTGTTGTGAATGGCGGGCTAGTTGGACTAGTCATAAATGGTGTATCCAACAAACATCCAGGGATTATATTTGAG

GTAATTAAATTATACAACTTCTACTTGTGTGCTTGATATCACTGCAAGTGTTTTTAATGATTTTTTCCATGTGTTTTAG

GATTGCAAAATGTTGAAGCATGTTGATCTGAGTTATTGGCGGTTATTAGAGGAAACCCCTGACTTCTCTGCAGCATTAAACCTTGAAAAATTATATCTTTTAAGTTGCAAACGTTTAAAAATGATTCATGGATCTGTTGCTTCTCTTAGTAAGCTTGTTACCTTGGACCTCGAAGGCTGTGAAAATCTAGAAAAGCTTCCAAGTAGCTTCCTCATGTTAAAGTCTCTTGAAGTTTTGAATCTAAGTGGATGCATAAAGCTAAAAGAAATTCCTGACTTATCGGCATCGTCAAACCTTAAAGAACTACATCTTAGAGAATGCTATCACTTGAGAATAATTCACGACTCTGCAGTTGGACGCTTTCTTGATAAACTTGTTATCCTGGACCTTGAAGGATGTAAAATTCTTGAAAGGCTTCCAAGATACATCAGCAACTCAAAGTCTATTGAAGTTATGAATCTTGATTCATGCCGAAAGATCGAACAACTTTTTGACAACTATTTTGAAAAGTTTCCAAGCCACCTCAAGTTCGAATCTCTTAAAGTTTTGAATCTTAGTTATTGTCAAAATCTTAAGGAAATTACTGACTTTTCAATTGCATCAAACCTTGAGATATTTGATCTTAGGGGCTGCTTCTCTTTAAGAACGATTCACAAGTCTGTTGGATCTCTCGATCAACTTATTGCCTTAAAACTTGATTTTTGCCATCAACTTGAAGAGCTTCCTAGTTGCCTCAGATTGAAGTCTCTTGATTCTTTGAGTCTCACTAACTGTTATAAGATTGAACAACTTCCAGAATTTGATGAAAATATGAAGTCTTTGAGGGAGATGAATTTGAAAGGTACAGCCATAAGGAAGTTACCCACATCAATTAGATATCTTATTGGGCTTGAGAATTTGATCCTTAGTTATTGCACAAACCTGATTTCTCTTCCAAGTGAAATTCATTTGTTAAAGAGTCTTAAGGAACTTGATCTTCGAGAGTGTTCTAGACTCGACATGCTTCCCTCGGGATCAAGCTTAAACTTTCCCCAACGAAGCTTATGTTCAAACTTGACTATATTGGATCTACAAAATTGCAACATATCAAATTCAGATTTTTTGGAAAATTTATCTAATTTCTGCACTACCTTGAAGGAGCTAAATTTGTCCGGAAACAAATTCTGTTGTCTACCTTCTCTCAAAAATTTTACATCATTGAGGCTTCTTGAACTAAGAAATTGTAAGTTTCTTCGAAACATTGTGAAGATTCCACATTGTTTAAAACGAATGGATGCTAGTGGTTGCGAATTGTTGGTAATAAGCCCCGACTACATTGCCGATATGATGTTCAGAAATCAG

GTTCCTCTATTTTAAGATTCATCTAATTTCTATTTTGTTCATATTATATGCTCCTCTCTTGTTTTGTTAACATTGATTTTGAGAATATTAAATCTTATTCTATAG

GACCTTAAGTTAAGGAACTTCAAAAGAGAGCTAATCGTAACGTACAGCGAGATCCCGAAATTCTGCAACAATCAAACCACAGAAAGTTCAATAAGTTTTAGCTTTCAACATAATTCAGATATGATTATACCTGCTTTGGTTGTGTGTGTTGTTTTCAAAGTGGATGCAGATTCATTTGTTGCTGAGGCTTTTATTCATTTTCAAGTATTGTTTGATGGTCAAAAGCTTATGATGCCTACTATGGAAAGTTGGTGTGGGTCAAAATCAGAGCATATGTTGTTACTAAGAACTCCTCCATCACAGTTGATATGCTTGAATGAGAATAATCGACACAAAATCGAAGTCTCGTTTCAGGTTCGGAATTATAATAAAAAGGCTAAAGTTATTATAAGAAGCCTG

>Cucsa.155730

AAAATAGTGGAAAGGGTTCAAAGCGAACTGCGAGTGACATATTTGGAAGTCGCCATCTACCCTGTTGGCATTGATCTTCGCCTCAAACACTTGATCTCATTAATGGCCATTTCTACAAACCACTCCACTCTCGTCCTCGGCATCTATGGCATGAGCGGCATTGGCAAAACCACTCTCTCTAAAGCACTCTTCAACCACTTCTTCCACTTCTTCAATTCTAGATCTTTTCTCCCCAACATCAACTCCCTCTCTACCTCCTCTCCCGACGGTCTCCTTCGACTCCAACAAACTCTCCTCTCCGATCTCCTCATCGCCACAAACCTCCGCTCTCGTTCCTCAACCACCACCGACTCCACCGTCGTTCGGATGCAGGAAAGACTCCAAAACAAAAAGGTCTTGGTAGTCCTCGACGACCTGGATCGTATCGAACAAGCAAATGCACTAGCAATACGGGACCGAAGATGGTTTGGAGACGGAAGCCGAATCATAATCACAACACGAAACAAACAAATTTTGGACACTCTAAAAGTCGACGAAGTTTACAACATGGAATCCAATCTACTGAACGACGAGGAATCGTTGGAGCTTTTTAGCTACCACGCATTCCGGGAGCAAAATCCACCAGAGGAGCTTTTGGAATGTTCGAAATCCATCGTTTCGTACTGCGGAAGCCTTCCTCTAGCTCTGGAAATCCTGGGTGGGTCATTCTTCGGAGGGAGACCGATGGAGGAATGGAGATCAGCGATGGAGAGATTGAAGAGGATTCCGGCGTGGGATTTGCAAGAGAAGCTTCGAATAGGGTTTGAAGGATTGAGAGATGAGATGGAGAGGGAGATATTTCTTGATGTGTGTTGCTATTTTGTGGGAATGAAAGAGGAATTGGTAGTGAAGATTATGGATGGATGTGGAATGTATGGAGAAAGTGGATTGAGAGGGTTGAAATGGAGGTGTTTGGTTGGTGTTGAGTTTTGGAGTGGGAGGTTGAAGATGCATGATTTGGTTAGGGACATGGGGAGGGAGATTGTGAGGCAAACATGTGTGAAGGAACCTGCTAGACGGTCCAGGGTTTGGCTTTATCATGAGGCTCTCAAAATCTTACTCCATCAGAAC

GTAACTTTAATTATTACTTCATGATCTATATTAGTTATATCTCCACCTACACTACTCATTTAATATCTAATATAGTTGTAGTGATCACTTAACAG

GGAAGTGAAAACATTGAAGGACTTGCAATAGACATGGGTAAAGGAAATAACAAGGAGAAATTCAGATTGGAAGCATTTGGGAAAATGAGAAATCTAAGGTTACTCAAACTCAACTATGTGCATCTCATTGGAAGTAATTTTGAGCATATAATAAGCAAAGAATTAAGGTGGATTTGTTGGCATGGATTCCCTTTGAAGTCTATTCCAAGCTCATTTTATCAAGGAAACCTTGTTGCCATTGACATGAGATATAGCAGCTTGATACACCCTTGGACTTGGAGGGATTCACAG

GTAAATTTATACAATTATTTAATAATTACCATTTTACTTTTATATATATATATATATATATAATGATTATTCATCCTATTTTTAGGTAAAAATTTAACTTTTCTTCGTTCCGTTTAGGATTTACTTTTATATATATATATATATATATAAATGATTATTCATCCATTTTTGGAAAAATTACTTTTCTCGTCCGTTTAGGGCTTAATTTTCCTCTATGTTTTTTCAATTTCAAAATGCTATACCTTTAGTTCTTATATTTTTTTTTCAATATTCCATAACATTTTTAACTACATCTCTCTCGCGTATGTTTTGGGCAG

ATTCTTGAGAATCTAAAAGTTCTAAACCTAAGCCACTCTGAAAAGCTAAAGAAGTCCCCAAACTTCACAAAGCTCCCAAACCTAGAGCAGCTAAAACTCAAGAATTGCACAGCCTTATCAAGCCTCCACCCCTCCATTGGTCAACTTTGTAAGCTTCATCTCATCAACCTCCAAAACTGTACAAATCTCTCGTCTTTACCAACCTCCATCTACAACCTCCACTCCCTCCAAACTTTCATCATCTCTGGCTGCTCCAAGATCCACTGCCTCCACGACGACCTCGGTCACCTTGAGTCCCTCACCACCCTTCTCGCTGACCGAACCGCCATATCCCACATTCCTTTCTCCATTGTCAAGTTGAAGAAACTCACTGACTTGTCTCTATGTGGTTGTAACTGCAGATCAGGGTATGTTGATCTTTTATTTTGTAACAATATATATGTGAGCTATATGATCAATATTAAATTAATGGAGCGCCTAAGCTTTGAAACATCTTGA

>Cucsa.237390

ATGGTCCCTTCTTCTCCTTCTTCTTCTGCTGCTTCTTCTTCTTCTCCTTCTTCTTCTCCTTCAATTGGTAAATGGAAATTTGATGTGTTCTTGAGCTTTCGAGGCGAAGATACACGTGGTGGCTTCACGGATCATCTCTACAAAGCCTTAACACGAAAGGGAATTTCAACATTTAGAGATGAAAATGAGATCGAAGAAGGTGAACACATTCCTTCAAATCTGCTGGCTTCCATTGATGCCTCGAGATTTGCCATTGTTGTGGTTTCGGAAGATTATGCATCTTCAAGATGGTGCCTCGAGGAATTGGCTAGGATGTTTGAATGTAAAAAAGAAGTTTTACCAATTTTTTATAAAGTGGATCCCTCTCATGTGAAAAACCAAAGTGGAACGTTTGAAGAAGCTTTTGTTAAACATGAAAAGAGATTTGGAAGAGGTGATGGGAAGGTTCAAAGTTGGAGGACGTTTCTCACCGAGCTTGCTAACACCAAAGCTTGGCTTTCTCAATCTTG

GTAATTAACTTAACTATTTATCATTTAAATTCTTAGTATATTGTAAATATAGATGGTGATATCCTAATATATTTTATGTAGCCACTTGGATTGAAATTTGGAAGAGAAATTTAGGCAAAAAATTTGCTCCTATTTTTTTTTCTTTTTCTTCTAATTTTCTTTTGTCTTCTAGATCTCTTTCACAATTAATATATGCATTTTAGTTTTAATCAAATATATAAAATTTTCCCTTTTGATCTCTTCCATATAGTTTAATTATTTGTTATTTAAATTGAAATAATGTATATATTGCCCCAATTTTGAGACCTAGAATAGAATCCAAGTTTTACTTTTTTTTTTTTCTAAAGCTTGAAATGTTTTGACAAACCTTTATCTTAGCTTGTATCTATGTATTTGTATACAATTATTGAGATGAATGATTGTGTATTCTTGTTCGGCTCTCAACTCTCAACTCTTGCTACTACACGCGTCACCGTTCTCGATTCTTACACTTAACACTCGCCACTACAAATCATTGCTTTCGATAATAATAAAAAGCAAGAGATTTCAAGGGGCGAGAAAACGGAAAAAAGGAACGAGAGCTAGTGAGGCAAAAAAAAAACATGAGCAAAAACGATTGGAAGAGTCCAAAAACCTAAACCTAAGATCCAAGGATAAGCAGGAGCAAGGATTGAAGGAAAGGCGAGTGTAGGATGCCCACTTGTATGCATATTATTATATGTTTCGATGATCACACCAAGCAGAAAGTCAAAAACTTCGAGATGGATTGCATCCGTCTTTTGTGCAATCCAACTAGCACCCAATAGGAACGTATCACACATTCTACTTTTGTTTTTTATTTAGTATGCGTTAAAATTATTTGTTTGCGTCTAAATGAGAGATTCACAATTAAACGCGTAATTTCTTCTCAGGTCACAATTGCACATCCATATACATCAGAATCATTTTAAATATTTAGGGACAAAAGTTGATAAATATTTTAAAAGCAATAACTATGTAAGACTTTTCTTGGTACCCAAAAAGCAATATATATATATATATATATGTTAGACTCAGTCAGCAAACCAATCATATAAAAGTAAACTGCCTTACATATTGAATTAATTAATTATAACTATCAAATTTTCTTGATTGTGATTGAAGTGGAAAAAAAAAATTGAAAGTTGTATTGTAATTGTAAATATAATATTTTAAGAAACCATTATTTATTAATAAATAATTAGCATTTGTGATAATAATAAAGTCAACATGTGTTTTATTTATGTGATAATGTTATATATGTTGAACATAAATCAATAGGTCATGTCACTATATAGTTTTTGTATATAGTTGTTATCAAATGAATGTGTGAATAAACTATCCAAGTGTATATGTAATCACTTATATTTACATGTGTTGTAATTTTTATTGTATTTTATACCTCGATTATAAATGGCAAATTTGAAATGATATTTGTGAAAGATTTATGTATCTTCATTAATTGTTTTCTTGCCATTAG

GTCACATGAATCAAATATCATTGAAGAAATCACCACAAAAATATGGAAAAGATTGAAACCCAATTTGACAGTCATTAAGGAAGACCAACTAGTTGGAATTAATTCTAAAATAAACAAACTTTCTTCACTTTTGATTCCAAACTCAGATGATGATGATGCTGATGATGATGTGATCTTTGTGGGAATACATGGAATGGGTGGCATTGGTAAGACCACAATAGCTAGGGTCTGTTATGAGCGAATTCGTGACGAATTTGAAGCTCATTGCTTCCTCTCCAACGTTCGAGAGAATTATATCAGAACCCTTGGGAACCTTTCATGTTTACAAACCAAACTCCTTTCAAGCATGTTTTCGCTTAAAAACAATCACATAATGGATGTTGAAGAAGGTACCGCTATGATCAATAAAGCCATTTTTCGAAAAAAGACACTTCTCGTCCTTGACGACGTGGATTCTTCGGATCAAATCAAAGGATTGATTCCAGACAACAACTCTTTTGGCAATGGAAGTAGAGTCATCATCACAACACGGAATGCGGATTTTCTTTCGAATGAATTTGGGGTGAAAAGAATTTTTGAAATGGATGAACTTAAATATGAGGAAGCTCTTCAACTTCTTAGTTTGAGTGCTTTTATGAAAACATGTCCAAAAGAAGGTTACTTGGAACACTCCAAGAAGATTGTAAAGGTTGTGGGAGGCCACCCTCTTGCACTCAAATTGTTAGGGTCGTCTCTAAGAAACAAAAATTTGAGTGTGTGGAATGAGGTGATAGAAGAGGTTGGAGGAGGTGGGAATATTCATGAAAAAATTTTCAAGTGTCTTAAAGTGAGTTATGATGGGTTGGATGAAAGGGAGAGAGAGATATTTCTTGACGTTGCTTGCTTCTTCAATGGGAAGAGAAGAGAAGTTGTAGAAGAGATATTAAATGGATGTGGTTTCTATGCCAAAACAAGGATTGAACTTCTTATTCAAAAGTCTCTCTTAACTCTTTCTTATGACAATAAGTTACATATGCATAATTTATTGCAAGAAATGGGTCGAAAGATTGTTCGGGATAAGCATGTTCGAGATCGATTAATGTGCCACAAAGATATAAAAAGTGTGGTAAGATATATATTAATG

GTGACCCTATTTGGTTGGAATGACTTTCTACATAATTCTTAAAAAAACTGCAAAAGAATAGTATCTTTAAATGTTTAGATGGTAACCATTTGATTTTATGTTAGTTTATTTTTTAAAAAAAACTAATGGTTTCAGGTGACAGAG

GCATTGATCCAAAGCATATTTTTCAAATCAAGTTCAAAGAATATGGTGGAATTTCCAATTTTGTTTTCAAGAATGCACCAACTTAGGCTGCTTAATTTTCGCAATGTGAGACTGAAAAACAAGTTGGAATATAGCATTCCAAGTGAGTTAAGGTATTTGAAGTGGAAAGGATATCCGTTGGAGTTTCTGCCAATCGATAGCTCTGAAGAATGTAAGCTTATTGAGCTTCACATGTGCCATAGCAATCTCAAACAATTTTGGCAACAAGAAAAG

GTAGCAATCTCATACCCATATATCTAAAGTTTTGGAAGAAGTTATTATGATATCTTTTAGTTTCAAACAATCTTTACTTATAACAGAATTTGGTGGAG

CTGAAGTATATCAAACTCAATAGTTCTCAAAAGTTGTCCAAAACTCCAAACTTTGCAAACATTCCAAATCTCAAAAGATTAGAGCTTGAAGATTGCACAAGTTTAGTCAACATTCATCCATCAATTTTCACTGCAGAAAAACTCATATTCTTGAGTTTGAAAGATTGCATCAATCTCACCAATCTTCCTTCTCACATTAACATCAAGGTTCTTGAAGTCTTGATTCTCTCTGGTTGTTCAAAAGTAAAAAAAGTCCCTGAATTTTCAGGTAACACTAATAGATTACTCCAACTCCATTTGGATGGTACCTCCATATCAAACCTACCTTCATCAATTGCAAGCTTGAGTCATCTAACAATATTGAGTTTAGCCAACTGCAAAATGTTAATCGACATTTCGAACGCGATTGAGATGACATCTCTCCAAAGCTTAGATGTTTCTGGATGTTCGAAGCTTGGAAGTAGAAAAGGAAAGGGGGACAATGTCGAATTGGGGGAGGTCAACGTGAGAGAAACCACACGAAGAAGAAGAAACGACGACTGTAACAATATTTTCAAAGAAATCTTCCTTTGGTTATGCAACACTCCAGCTACTGGCATTTTTGGGATCCCATCATTAGCTGGTTTGTACTCTCTTACAAAACTAAACTTGAAGGATTGCAACCTTGAAGTAATCCCACAAGGGATTGAGTGTATGGTGTCATTGGTAGAGCTCGACTTGAGTGGCAATAATTTCTCTCATCTTCCAACAAGCATATCAAGACTTCATAACTTGAAAAGATTGAGGATAAACCAATGCAAAAAGCTTGTACATTTCCCAAAGTTACCTCCAAGGATCTTGTTTTTGACGTCAAAGGATTGCATTTCATTGAAAGATTTTATAGATATTTCAAAAGTTGATAATTTATACATAATGAAAGAAGTGAACCTTTTGAACTGCTACCAGATGGCTAACAACAAAGACTTCCATAGATTGATCATTTCTTCGATGCAGAAGATGTTCTTTCGAAAAGGAACATTCAACATCATGATTCCGGGGAGTGAGATTCCCGATTGGTTTACAACAAGGAAAATGGGATCTTCGGTATGCATGGAGTGGGATCCAGATGCCCCAAACACCAACATGATTCGATTTGCGCTCTGCGTCGTTATTGGTCTGAGTGACAAAAGCGACGTTTGCAATGTTTCGTCCTTCACCATTATCGCATCAGTGACTGGAAAAGACCGTAACGACACGAATTTGAAGAATGGAGATGATCTTCTGGTTGATGGATTTCTTGTTTCAGGGATGAAGAAGTTAGACCATATATGGATGTTTGTTTTGCCACGAACTGGGACTCTGCTAAGAAAGATTAGCAACTATAAAGAGATTAAGTTTAGATTCTTACTTCAAGCTATT

>Cucsa.338650

ATGGCCGACGAGCTCCGACCTCAACACGGGAATTGGACTTACGATGTTTTCTTGAGTTTTAGAGGTGAAGATACTCGCAAGAACTTCACTGATCATCTCTACTACGCATTCAAAGATGCAGGCATCAATGTGTTTCGAGACGATCCAGAGCTCGAACGGGGTGAAGACATAAGTTCGGAGCTCGAGCGAGCGATCGAAGGGTCGAAGGTGGCAGTTGTCGTATTCTCGGAAAGGTATGCGGAGTCGGGATGGTGTTTGGAGGAGTTGGTAAAGATCATGGAGTGCAGGAGGACTTTGAGACAACTGGTTTTCCCAATATTTTATAATGTGGATCCTTCATGTGTGAGGAAGCAAAAGGGTGAATTTGAAGAGGCTTTTGTTAAACATGAAGTGCGTTATTTTAGGGATATTGATAGAGTTCTTAAGTGGAGAATGGCTCTCACTGAAGCTGCTAATTTATCTG

GTTGGGATTTGAGAAACATTGCAAATGGGTATTTATTGATTCCTTCCTTTCTTTCAAGTTTTGAATGTTAATTAGCATATAGAAGTTTCTAAATAGCCCTTTTGGTTTTTTGAAATTAAGCTTGGGAATACTTGGTTAAATTGCAAATTTGGTCCAAGAAAGTTATAGTTTAGTTTCTATGATAACTTATAAAAATCTGATAAAACACCAGTTTTAGAAAACATGTTCTTTGTTTTTGGAAATTGAACTCACAACTCATATGGACTGAAAAATTCACATTAACATATCATATACCCAACCATTATATTTGAGTCTTCTCCTAAGTTTCGATGAGTAACTTATCTAAATCTTGCTATATGGCAGACATGAAG

CGAAGTTCATAAGGTTGATTGTTGAAAAGGTATCAAAGGAGGTGAACAGTAAATACTTATTCATAGCTCTTTATCCAGTGGGAATTGAATCAAGACTCAAACTTCTTTTATCACATCTTCATATTGGTTCAAATGATGTTAGGTTTGTAGGAATTTTGGGGATGGGAGGACTGGGTAAAACCACCGTTGCAAAAGCACTTTACAACCAGCTTTATCACAACTTTGAAGCCAAATGTTTCCTTTCCAATATCAAAGCTGAAACCTCCAATCTAATTCACTTACAAAAACAACTCCTCTCTTCCATCACAAATTCTACCAACATCAATCTTGGAAACATCGACCAAGGAATCGCAGTGTTGCAAGAAAGACTTCGTTGCAAAAGGCTTCTTTTGATATTAGACGATGTAGACGACTTAAGCCAGTTAACTGCATTAGCAACAAGTCGTGATTTGTTTGCTTCAGGTAGTAGAATTATCATAACAACTCGAGATCGACATCTGCTAAATCAGCTTGAAGTAGACGAAATTTGTTCCATCGATGAAATGGATGACGATGAAGCACTTGAACTCTTTAGTTGGCATGCTTTTCGCAATAGTTATCCATCAGAAACCTTTCATCAACTTTCGAAACAAGTGGTCACTTATTGTGGAGGATTGCCATTAGCTCTCGAAGTGTTGGGTTCTTTCCTTTTTGGTAGAAGTAGAGAAGAATGGGAAGATACACTGAAGAAATTGAAGAAAATCCCAAACGATCAAATTCAAAAAAAGCTTAAAATAAGCTTTGATGGGCTAAACGATCATACTTACAAAGATATATTTCTCGACGTGTCATGTTTCTTTATTGGAATGGAAAGAAACTACGTTGAACAAATATTAGATGGGTGTGGATTTTTTCCAAGAATCGGAATTAGTGTTCTTCTTCAAAGATGTCTATTAACAATTGGAGACAAAAACAGATTAATGATGCATGATTTGTTAAGAGATATGGGGAGAGAAATTGTTCGTGAAAATTTTCCAAAATACCCTGAGAGACATTCAAGACTTTTTCTTCATGAGGAAGTGCTTTCTGTTCTTACAAGACAAAAG

GTAAGAAAAGAAAGTCCTATTTAAGTTGATCCAATTTTATTTTTAAGCTTAATATTTTTTATTTCATTGGTTTAG

GGAACTGATGCAACTGAAGGCCTAAGTTTGAAGTTGCCAAGATTTAGCAAGCAGAAGTTGAGCACAAAAGCATTTAATGAAATGCAAAAATTGAGGTTACTTCAACTTAATTTTGTTGATGTAAATGGAGATTTCAAGCATATTTCTGAAGAGATAAGATGGGTTTGTTGGCACGGATTTCCTTTGAAGTTTTTGCCTAAAGAATTTCATATGGACAAATTGGTTGCTATGGACTTGAGATATAGCCAAATCAGATTCTTTTGGAAGGAGTCTAAG

GTACACATTTTACTGTTGAATTAAAATGTTTTACTTTGGTTCTATACCTAGAATATAGCCAAATCAGATTCTTTCAATGAGTCTTTTTCACTGCTACTGTTAAAGTAGTGTTGATATTATTGAATTTACCGTAACTTATCAACTTTAACATTTTGGATTAATCACTGTTATTATAGCATCATAGCAATGCCATTTTCTTCTCAATTAACGATTTTTCCTGATCTCTTTGTTCTCTTTTGTGTGAACATGCAG

TTTCTCAAGAATTTGAAGTTTCTTAATCTAGGCCATTCTCATTACTTAACCCACACTCCAAATTTCTCCAAACTCCCCAATCTAGAGATACTCAGCCTCAAAGACTGCAAGAATTTGATTGAATTGCACCCTACAATTGGAGAATTAAAAGCCCTCATTTCCCTAAACTTAAAAGATTGCAAATCCCTCAATTCACTTCCAAATAGTTTCTCCAACTTAAAATCCTTACAAACTCTCATTATTTCAGGTTGTTCAAAGCTCAATAGTTTGCCAGAAGATTTAGGCGAAATTACATCATTAATAACTCTAATAGCTGATAACACACCAATCCAAAAAATCCCTAACACAATTATAAACTTAAAAAACCTCAAATATTTATCTTTATGTGGGTGCAAAGGGTCACCATCAAAATCATCATTCTCTTCAATGATTTGGTCTTGGATTTCACCAAAGAAATTATCTCAAAACTACACATCAATTCTTCTCCCTTCTTCATTACAAGGCTTAAACTCCTTAAGAAAATTATGCCTTAAAAATTGTAACTTGTCAAATAACACAATTCCAAAAGATATCGGGAGTTTGAGTTCTTTGAGAGAATTGGATTTGAGTGAGAATTTATTCCACAGTTTGCCATCAACTATCAGTGGCCTTTTGAAACTTGAGACACTTTTGTTGGATAATTGCCCTGAACTTCAACTTATACCAAATTTGCCACCACATTTGAGTTCATTGTATGCATCAAACTGTACTTCATTGGAAAGGACTTCAGATTTGTCTAATGTGAAGAAAATGGGATCTTTGTCTATGAGTAATTGTCCTAAACTTATGGAGATTCCTGGCTTGGACAAATTATTGGATTCTATTAGAGTTATTCACATGGAAGGATGTAGCAACATGTCCAATTCCTTCAAGGATACCATTCTACAG

GTTCTAATCTCTCTCTCTTTATTAATTGAAAACGAACACAAAATTAGAATAACTAAAATAAACATCGTTTTGTTTTGATTTGAAAGGGATGGACAGTTAGTGGATTTGGAGGAGTATGTCTTCCAGGCAAAGAAGTTCCAGATTGGTTTGCATACAAAGATGAAGGTCACTCAATATTTTTAGAATTGCCTCAGTATAATAATTCCAATTTAGAAGGCTTCATTGTTTGCATAGTTTACTGTTCTTGTTTTAACAACACAGTCTCAACTGACCTTCCAAGTTTATCAGTCATTAATTACACAAAATCTTCCATTACAACCAACAAACCTCTTACCAATGATGTAATAATGTCAACTCAAGATCACTTGTGGCAAGGCCATTTATCTAACAAAGCCTTCAAGATGGAACCTGGCGATGAAGTCGAGATCATCGTTGATTTCGGTGCTGAAATCACCGTGAAGAAAATTGGCATCTCGCTTGTGTTTGACAAG

TATGTCGATCAAACAATGTTAGAGTTTGCATCCACCTGTAATGATGATGATGTCGTCGTGGATAACCAAGATGAAACTGTAAGTGAAAAGGATGGAGAAGTTGGGAGCAAGAGAGGTTTTGACGAGAATGATGATGAAGGATTGAAAAATTCATACCAAATTCCCAAAAGGTTGAAGTGTGAGATTGATTCTAACATGAAAATTGATGAGGAGTAG

>Cucsa.338660

ATGGCCAACGAGTTCCAAGCTCAACATGGAGACTGGACGTACGATGTTTTCTTAAGTTTTAGAGGCGAAGATACTCGAAAAAACTTCACAGATCATCTCTACTACGCATTGAAAGATGCAGGCATCAATGTCTTTCGAGACGACCCAGAGCTCCAACGAGGCGAAGACATAAGTTCGGGGCTGGAGCGAGCAATCGAAGGATCGAAGGTGGCAGTTATCGTATTCTCGGAAAGGTATGCGGAGTCGGGATGGTGTTTGGAGGAGTTGGTAAAGATCATGGAGTGTAGAAGGACTTTGAGACAAATGGTTTTGCCAGTATTTTATAATGTGGATCCTTCATGTGTGAGGAAGCAAAAGGGTGAATTTGAAGAGGCTTTTGTTAAGCATGAAAAGGGTAAGGATATTGATAAAGTTCGTAGGTGGAGAATGGCTCTCACTGAAGCTGCTAATGTAGCTG

GTTTGGGTTTGACACAAAATGCAAATGGGTATTTATTTCCCTCCTCCTCCTCCTTCTTCTTCTTCTTCAACTTAGTCGACTATGCTTTAATGTTTCTAATCGTGAGTGAATAAAAAAAACAAAAAAGTAGTTTTGAAAGCGGGTTTTTGTCTTTGAAAGTTGAACTAAAAAAGGACTAATTTTACCCAAACATCATAACGAATCTTTATATTTGAGGAAATGAATCTGATGTTTTGATGAGTAGATGTGAATCTTGTATGGCAGGCATGAGGCAG

AATTCATAAGGTCAATTGTTAAAATGATATCAAAGGAGGTGAAGAGCAATTACTTATTCATAGCTCTCTATCCAGTGGGAATTGAATCAAGAATCAAACTTGTTTTACCACATCTTCATATTGGTTCAAATGATGATGTTAAGTTTGTAGGAATTTTGGGGATTGGAGGTTTGGGAAAAACCACCATTGCAAAAGCACTTTACAATCAACTTCATCACAACTTTGAAGCTGCATGCTTCCTTGCTAATATCAAACAAACCCCCAACCAACCCAATGGTCTAGTTCACTTACAAAAACAACTCCTCTCTTCGATTACAAATTCCAGTAACATCAATTTCGAAAACATGGATCGAGGAATCGTTGTGTTGCAAGAAAGCCTTCGTCGCAAAAAGCTTCTTTTGATATTAGACGATGTAGACAAAATAAGCCAATTAACTGCATTAGCAACAAGACGTGAATGTTTCGGTTCAGGTAGTAGAATTGTCATAACAACTCGACATCGACGTTTACTAAACCAGATTGAAGTAGATGGAATTTGTTCCATTGATGTAATGGATGACGCTGAAGCGCTCCAACTCTTTAGTTGGCATGCCTTTCACAATAGTTATCCTTCAGAAACTTTTCATCAACTTTCAAAACGTGTTGTTAATTATTGTGGAGGATTGCCATTAGCCCTTCAAGTGTTGGGCTGTTTCCTTTTTGGCAGAAGTAGAGAAGAATGGCAAGATACATTGAAGAATTTGAAGAAAATTCTAGATGATCAAATTCAAATAAAGCCTAAAATAACCTTTGATACCCACAATGATCACACTTGTAAAGATATATATCTTGTGAACCAAATGTTAGATGGGTGGGGATCTTTTCCAAGAATTGGTGACATAAACAGATTAGTGACAAGTGATTTGTTAAGAGATCATACCCAACTTTTTCTTCCGAAGGAAGTGCGCCTTTCTGTACTTGGACCAAAGGTAAGAAAATAA

>Cucsa.237410

ATGGCTTCTCCAGCAATAATGGAGAGAAGAGCTTCAATTAAATCCTTATCTCCTCCTCCCTATTCTATCTCTCTTCCTCTTCCTCCCTTACGAAACTATGACGTTTTCCTCAGCCACAGAGTTAAGGATACCGGGAGTAGTTTCGCAGCTGATCTTCATGAAGCTTTGACAAACCAAGGAATTGTAGTTTTCAGAGACGGCATAGACGACGAAGACGCAGAGCAACCATATGTAGAGGAGAAGATGAAGGCCGTGGAAGAATCGAGGTCTTCGATCGTGGTTTTTTCAGAGAACTACGGGAGTTTTGTTTGCATGAAGGAAGTAGGGAAGATTGTAACGTGTAAGGAGTTGATGGATCAACTGGTTCTTCCTATATTTTACAAAATAGATCCAGGCAATGTGAGGAAGCAAGAGGGGAACTTTAAGAAGTACTTTAATGACCATGAAGCCAATCCTAAGATTGATATTGAAGAAGTTGAGAACTGGAGATATTCTATGAATCAAGTTGGCCATCTCTCTGGATGGCATGTCCAAGATTCCCAGTTAA

GTAATAATTATATATTCACTGATCTCTTCAGCTTTTTCTTTCTAATTTACGTATGTTTTGTAGTTTATAATTACAAAGTTTACAAAAGGAAGCACAAATGTGTTTTTAAACGTCATCTCTAAAATTTAGATTTTAAACCAAATTCCAAAACAATAACAATATTTTCAAAATTTAGAGATGATTACTAAAATTGAAAAGTATATATATGTATATAGGTCTGAAG

AAGGGAGCATAATCAATGAAGTTGTGAAGCATATATTCAACAAATTGCGTCCTGATTTGTTTCGATATGATGATAAATTAGTTGGAATTTCCCCAAGATTACACCAAATAAATATGCTTTTGGGAATAGGTTTAGATGATGTACGCTTTGTTGGAATATGGGGAATGGGTGGAATTGGCAAAACTACAATTGCTAGAATCATTTACAAAAGTGTTTCTCATTTATTTGATGGATGTTATTTCTTGGACAATGTCAAAGAAGCTTTGAAGAAAGAAGACATAGCTTCATTACAACAAAAGCTTCTAACAGGAACTCTAATGAAAAGAAACATTGACATCCCTAATGCTGATGGAGCTACATTAATTAAGAGAAGAATAAGTAATATTAAAGCTCTTATAATTCTTGACGATGTCAACCATCTAAGCCAACTTCAAAAATTAGCCGGCGGTTTAGATTGGTTTGGCTCAGGAAGTCGAGTCATCGTTACAACGAGAGACGAACATCTCCTAATTTCACATGGAATCGAAAGACGATACAATGTTGAAGTGCTGAAAATTGAAGAAGGTCTTCAGCTTTTTTCACAAAAGGCATTTGGAGAAGAGCATACAAAGGAAGAGTATTTTGATGTTTGTAGCCAAGTTGTAGACTATGCTGGAGGACTTCCATTGGCAATTGAGGTTCTTGGATCTTCTTTACGTAATAAACCAATGGAGGATTGGATAAATGCAGTGGAAAAGTTGTGGGAAGTTCGTGATAAGGAAATTATAGAAAAGTTGAAAATTAGTTATTATATGTTGGAGAAATCTGAACAGAAAATTTTTCTAGATATTGCATGTTTTTTTAAGAGAAAGAGTAAGAAACAAGCAATAGAAATTCTTGAAAGTTTTGGATTTCCTGCTGTTCTTGGACTAGAAATATTGGAGGAGAAATGTCTTATTACTACACCACATGATAAGCTACATATGCATGATTTAATACAAGAAATGGGCCAAGAAATTGTTCGCCAAAACTTTCTGAATGAGCCCGAAAAGCGAACTAGGTTGTGGCTTCGTGAGGATGTCAATCTCGCACTAAGTCGAGATCAG

GTAACTATATATATATATATTGTTTTAACTCATAGGAAAACTTGGCATGGGAATGATATATATCTATATCTATATAGATGTATGATGAATTAGTAATTTGTCAG

GGAACAGAAGCAATTGAAGGGATAATGATGGATTTGGATGAGGAAGGAGAATCACATTTGAATGCCAAAGCCTTTTCAGAAATGACAAATCTAAGAGTATTGAAATTGAACAATGTTCATCTTAGTAAAGAAATTGAATATCTGTCTGATCAACTAAGGTTTCTCAATTGGCATGGTTACCCTTTAAAGACCTTACCATCAAATTTCAATCCCACAAATCTATTGGAGCTTGAGTTGCCAAATAGCTCTATTCACCATCTTTGGACTGCTTCAAAGGTACATCAAAACAACAGTAGTAATTAA

>Cucsa.237440

ATGGCTTCTCCAGCAATAATGGAGAGAAGAGATTCAATGACATCCTTATCTTTTCCTCCTCCTCCTCCTCCTCCTTATTCTATCTCTCTTCCTCTTCCTCCCTTACGAAGATATGACGTTTTCCTCAGCCACAGAGCTAAGGACACTGGATGTAGTTTCACTTCCAACCTCCACGAAGCTCTAACAAGTCAAGGAATTGTAGTTTTCATAGACAAGGAAGACGGAGGGAAACCGTTAACGGAGAAGATGAAAGCGGTGGATGAATCGAGGTCTTCGATCGTGGTTTTTACCAAGAATTATGGGAGTTTGGTTTGCATGAAGGAAATAAGGAAGATTAGAATGTGTCAGAAGTTAAGGGATCAATTGGTCCTTCCAGTATTTTACAAAATAGATCCAGGCGATGTGAGGAAGCAAGAGGGGAGCTTTGAGAAGTACTTTAATGAACATGAAGTCAATCCTAATATTAGTATTGAAGAAGTTAAAAAATGGAGAAAATCTATGAACAAAGTTGGCAATCTCTCCGGATGGCATGTCCAAGATTCCCAGTTAA

GTAATTATTCAACTTTCACTTTTTTCTTTAATTTTTTAAGCTTTTGTGAGATTAGTTGAGGTGTATGAAGATGGAATATTTCTAGAGAAATTAAACAGGTTTAGCCAGAAAAAGATTAGATAAAGGAAAGTTTTAGTTCTTTTAGTTATTATTTTTTTAGGCCAAGGGAGAAAAGCAATAGATTAAAGAAAAAGTTAAAGAGATGTTTTAGAGAGAGAAGCTAAAAAATGGAGCAAATTAAAAGATCAATTAGAGAGAGAACATAAGAGGAATCTAATTCTCAATTATAAATCCAAATGGAAATCAATAATAAAATAATAGAAAGCAAAATTATAATGATTTTGTTCCTAATCATTTTATTTTATAACTTATAAGATGTGAAGTCAATATAAATGTGATACGTTCTATGTCTATCAACAAAAGTTTCCATCTTTGCTAGCCAAACATTTAATTTATTAGCCAAATTAATTCTAAAAACGATAACAACTATGAATGAAATTTATATGTAGGTCTGAAG

AAGGGACCATCAATGAAGTTGTGAATCATATTTTCAACAAATTACGTCCAGATTTATTTCGATATGATGATAAATTAGTTGGAATTAGCCGAAGATTACATGAAATAAATAAGCTAATGGGAATAGGCTTAGATGACGTACGGTTGATTGGAATATGGGGAATGGGTGGAATTGGCAAAACAACCATCGCTAGAATCATTTACAAAAGTGTTTCCCATTTGTTTGATGGATGTTATTTTTTGGACAATGTCAAAGAAACTTTAAAGAAAGAAGGCATAGCTTCTTTACAACAAAAGCTTCTAACAGGAGCTCTAATGAAAAGAAACATTGACATCCCTAATGCTGAAGGAGCTACATTAATCAAGAGAAGAATGAGTAATATTAAAGCTCTTATAATTCTCGATGATGTCGACCATCTAAGCCAACTTCAGCAGTTAGCTGGCGGTTCGGATTGGTTCGGTTCAGGAAGTCGAGTCATCGTTACGACGAGAGAAGAACATCTCCTAATTTCACATGGAATCAAAAGACGATACAATGTTGAAGTGCTGAAAATTGAAGAAGGTATTCAGCTTTTCTCACAAAAGGCATTTGGAGAGGACCATCCAAAGAAAGGGTATTTTGATCTTTGTAGCCAAGTTGTAGATTATGCTGGAGGGCTTCCATTAGCAATTGAGGTTCTTGGATCTTCTCTACGTAATAAACCAATGGAGGATTGGATAGATGCTGTGAAAAAGTTGTGGGAAGTTCGTGATAAGGAAATTATTGAAAAGTTGAAAATTAGTTATTATATGTTAGAGAAAGATGATAGGGAAATTTTTCTAGATATTGCATGTTTTTTCAAGAGGAAGAGTAAGAGACAAGCAATAGAAATTCTTGAAAGTTTTGGATTTCCTGCTGTTTTTGGACTAGACATATTGAAGGAAAAGTCTCTTATTACTACACCACACGAGAAGATACAAATGCATGATTTGATACAAGAAATGGGTCAAAAAATCGTTAACGAAAAGTTTCCAGATGAACCCGAAAAACGAAGCAGGTTGTGGCTTCGTGAGGATATAACTCGTGCTCTAAGTCATGATCAG

GTAACGTTTCTGAATTGAAATCTTTTGAGCTATTATCTTTCTACAATAAATATATAATGTTTAGTAACGTTTGATCTTTAAACTTCTAAATTTGTCAG

GGAACAGAAGCAATTAAAGGGATAATGATGGATTTGGATGAGGAGGGAGAATCACATTTAAATGCCAAAGCCTTTTTTTCAATGACAAATCTAAGAATATTGAAATTGAACAATGTTCATCTTAGTGAAGAAATTGAATATCTGTCTGATCAACTAAGGTTTCTCAATTGGCATGGTTACCCTTTAAAGACCTTACCATCAAATTTTAATCCCACAAATCTATTGGAGCTTGAGTTGCCAAATAGCTCTATTCACCATCTTTGGACTGCTTCAAAGGTACATCAAAACAACAGTAGTAATTAA

>Cucsa.237560

ATGGCTTCCTCAACCCCCAAGGAATTATCTTCTTTTTCTTCTTCTCCTAGATTCATATTTGACGTCTTTCTCAGCTTCAGAGGCGTCGACACTCGCAAGAATGTCACAAATCGTCTTTACGAAGCTCTGAGGCGACAAGGCATCATTGTTTTCAGAGATGACGATGAGCTCGAGAGAGGGAAGACTATTGCTAACACTCTAACCAACTCGATTAACCAATCCAGGTGTACCATTGTTATTCTCTCTAAAAGATATGCAGATTCAAAATGGTGCTTGAGGGAGTTGGTTGAGATTGTCAAATGCAAGAATACCTTCAAGCAATTAGTTCTTGTGGTTTTCTACAAAATTAAGCCCTCCGATGTCAACAGCCCTACTGGGATTTTTGAGAAATTTTTTGTTGATTTCGAAAATGATGTTAAGGAGAATTTTGAAGAGGTTCAGGACTGGAGGAAGGCCATGGAAGTGGTTGGAGGTCTCCCTCCATGGCCTGTAAATGAACA

GTATGATTCTTTGCTCCCTTTCTCGTATTGTCGTCATTGTTCCCTTTAGCTATTGAATTTGGAATTTGAAATTCATGTTTGAGTGATGTTGGATGTTAAAGAAATGGACAATCAAAGTTGGATAGAAAACGTAAAGCAGCAGAGAATCGACACATATTTCTGAGGGAGAGCAGTTTATTTCTGGAGATAATAATTCATTCTATATAAACTCCCTAACGTTAATGGTGCATCCTTTATTATTCTCTCTATTAATAAACTACTCTCCTTGGGTGAACGTAGTTGACACATTGATAGTGAACCATGTGAATATGTGGTTCGATTATCTTCATATTTTCATTTCTTCTCTATCATTGATTGTCTGATTTCTAGTACCAGAATTGTGTCTGTTGATCACTGAAAGACTAAACTTATATTTTTACCTTCTAGACATGAAGGCATAGGATTAGTTAGGATAAACTTATCTACGTGAATGATAGTTGTTTGTATTATTTGCAG

GACCGAAACAGAGAAAGTCCAAAAGATTGTTAAGCATGCTTGCGATCTTCTGCGTCCTGATTTGCTTAGCCATGATGAGAATTTGGTTGGCATGAACTTGAGATTAAAAAAAATGAATATGCTTATGGGCATAGGACTGGATGATAAGCGCTTTATTGGGATATGGGGGATGGGTGGAATAGGCAAGACAACTATTGCTAAAGCTGTTTTCAAAAGTGTCGCTCGTGAATTCCATGGAAGTTGCATTCTGGAAAATGTTAAGAAAACTTTAAAGAATGTTGGAGGCTTGGTGTCCTTGCAGGAGAAACTTCTTTCCGATACTCTAATGAGAGGAAAAGTTCAAATTAAAGATGGCGATGGAGTTGAAATGATAAAGAAAAACTTAGGAAATCAAAAAGTTTTTGTTGTTCTTGATGGTGTTGATCATTTTAGCCAGGTGAAAGATCTGGCAGGAGGAGAAGAATGGTTTGGTTGTGGAAGTAGAATCATCATTACAACAAGAGATGAAGGTTTGCTTCTTTCTCTTGGAGTTGATATAAGATACAATGTTGAGAGTTTCGATGATGAAGAGGCTCTTCAGCTCTTTTGCCATGAAGCATTTGGAGTAAAGTTCCCTAAGAAAGGTTATTTGGATCTTTGTATGCCATTTATAGAATATGCTGAGGGCCTTCCATTAGCAATCAAGGCTCTTGGGCATTCTTTGCACAATAGATTGTTTAAGTCATGGGAAGGTGCTATTAGAAAGTTAAATAATTCTTTAAACAGGCAAGTATATGAAAACTTGAAAATTAGTTACGATGCACTTGGAAAGGAAGAGAGGAGAATTTTTTTGTATATTGCCTGTTTTCTTAAAGGACAGAACAAAGACCAAGTCATTGACACATTCGTGAGTTTTGAAATTGATGCTGCTGATGGGCTTCTTACCAGAAAAAATGCTGCCGATGTACTTTGTATAAAAGAAACTGCTGCTGATGCTCTAAAAAAATTGCAGGAGAAATCCCTTATAACTATGTTATATGACAAAATAGAGATGCATAATTTACACCAAAAACTAGGTCAAGAAATTTTTCATGAGGAGTCATCGAGGAAAGGTAGTAGGCTATGGCATCGAGAGGATATGAACCACGCTTTAAGGCATAAACAG

GTAGAACTTCAATAAGGAAATGTAAGCAATGTTACTCATAGTTCAAAGACAATCATTTGACTTTGATTTCTTCTCATTGCATATCCTCAG

GGAGTTGAAGCTATTGAAACCATTGTCTTGGACTCAAAAGAGCATGGAGAGTCACACTTAAATGCCAAGTTCTTTTCAGCAATGACCGGTCTAAAAGTGTTGCGTGTTCATAATGTATTCCTTTCTGGAGTTCTTGAATATCTCTCAAACAAGTTGAGACTTCTCAGTTGGCATGGATATCCCTTCAGAAATTTACCATCGGATTTCAAGCCGAGTGAACTATTGGAACTCAATTTACAGAATAGCTGCATTGAAAATATTTGGAGAGAAACAGAG

GTAGGCAGTGTAGCTTTTATCAATAATATTGTTTATTTTCTTTGGAGGTTGAATTATAATATCATAACATTCTTGTAACCATATCTTTTTTTAACAG

AAGTTGGATAAATTGAAGGTAATTAACCTTAGTAATTCCAAGTTCTTATTGAAGACCCCTGACCTGTCAACGGTGCCAAATCTTGAGAGGTTGGTCTTGAATGGTTGTACAAGACTACAAGAGCTTCACCAATCTGTCGGCACTCTAAAGCATCTAATCTTTTTGGATCTTAAGGACTGCAAATCTCTCAAAAGCATTTGTTCTAATATTTCTCTTGAATCACTCAAGATTCTCATTCTTTCTGGTTGTTCAAGACTTGAAAATTTTCCAGAGATTGTGGGAAACATGAAACTTGTGAAAGAGCTTCATTTAGATGGCACTGCTATTCGAAAATTGCATGTCTCGATTGGAAAACTTACAAGCCTTGTTTTGTTGGATCTTAGATACTGCAAAAATCTTCGTACACTTCCAAATGCAATCGGTTGCTTAACATCCATTGAACATCTCGCATTGGGTGGCTGCTCAAAGCTTGATAAAATTCCTGACAGCTTGGGGAACATTTCTTGTTTAAAGAAACTTGATGTGAGTGGTACTTCTATTAGTCATATCCCATTTACTCTAAGACTTTTGAAGAACCTTGAAGTATTGAATTGCGAAGGCCTATCCCGAAAATTATGTTATTCGTTGTTCCTATTATGGAGTACGCCGAGGAATAACAATTCACATTCATTTGGTTTGTGGTTGATAACTTGCTTAACGAATTTTAGTTCGGTAAAGGTTTTGAATTTTAGTGATTGCAAGCTGGTAGATGGAGACATACCCGACGACCTCAGCTGTTTGTCTTCATTGCACTTTCTGGATCTAAGCAGGAACCTCTTCACCAATCTGCCTCATAGTTTGAGTCAACTTATCAATCTCAGATGCCTTGTTTTGGACAACTGCAGTAGACTCAGGTCATTACCGAAGTTCCCAGTCAGTTTACTTTATGTACTCGCAAGGGATTGTGTGTCACTGAAAGAACACTATAACTATAACAAAGAAGATCGCGGGCCTATGAGCCAAGCAGAAGTAAGGGTCCTTAGTTACCCCTCATCAGCTAAAGACCAAAACTCTAAAATCTCTCAGTTAATGATATCAAGTATGTGCACAGCTTGCGAGAATGGGGGTTGA

>Cucsa.237520

TTACTGACAATTAACTTGACGGAGTTTGCATGCACATGCAGCTATACAAAGTTCAAAATCCAATCCATCGCAAATTCAATAGGTGATCACTTGCTTCGTCTTAAGCTCCAAGCCAAAGAAGAAAATTTATTTGAAATGCCACTTCGATTAAGAACAATGAAAATGCTCCTTGGCTTAGGCTCAAATGACGTACGTTTTATAGGGATAGTGGGGATGAGTGGTATTGGTAAAACAACCCTTGCGGAAATGACATATTTACGTATTTTTAAGCCTTTTGTATCTGCCTTACGAAAACCTTACTTTCTTCACTTTGTTGGACGCAGTATAGTCTCCTTGCAGCAACAACTACTTGATCAACTTGCTTTCCTAAAACCTATCGATATCCAAGTTTTGGATGAGAATCATGGAGTAGAATTGATTATGCAGCATTTGAGTTCACTTAAAAATGTGCTTATTGTTTTTGATGGAATAACCGAAAGAAGTCAATTAGAAATGTTAGCTGGCAGCCCCGATTGGTTTGGCGCAGGGAGTCGAATCATTATTACAACCACAAATAAAAATATTTTTCATCACCCTAATTTCAAAGACAAAGTGCAAGAATATAATGTAGAATTACTTTCTCATGAGGCTGCCTTCTCCCTCTTTTGCAAGCTTGCATTTGGAGATCACCCTCATACCCAGAATATGGATGATCTTTGTAATGAGATGATCGAAAAGGTTGGAAGACTCCCATTAGCTTTGGAAAAAATAGCTTTTTCATTGTATGGTCAGAACATAGATGTATGGGAACATACATTGAAGAATTTTCATCAAGTAGTTTATGATAATATTTTCTCTGATGTATTAAAGTCAAGTTATGAAGGATTAGAAGCAGAGAGCCAACAAATTTTCCTAGATTTGGCATGTTTCCTCAATGGAGAGAAGGTGGATAGAGTGATTCAAATACTTCAAGGCTTTGGTTATACCTCACCTCAAACTAATTTGCAATTGTTGGTTGATAGATGTCTTATTGATATTTTAGACGGACATATACAAATGCACATCTTGATTCTTTGTATGGGCCAAGAAATTGTGCACCGCGAGCTGGGAAATTGTCAACAAACAAGGATTTGGCTTCGAGATGATGCTCGTCGTCTATTTCATGAAAACAAT

GTAAGACATTGTATACTTTATAATGCATGTATATACTATTGTTTTTAGTCCTTGTAATCAATTATTGCTATGTTAATTATGTTTTGGTTTGCCAG

GAATTAAAATATATTCGTGGAATAGTGATGGACTTAGAGGAGGAAGAAGAATTGGTATTGAAGGCTAAGGCATTTGCAGATATGTCTGAGCTAAGAATTTTACGAATCAACAATGTGCAACTTTCGGAAGATATTGAATGTCTGTCAAATAAATTGACGTTGCTCAACTGGCCTGGCTATCCTTCAAAGTATTTGCCATCAACTTTTCAACCACCATCTCTGCTTGAGTTACACTTGCCTGGTAGTAATGTTGAACGACTCTGGAATGGAACACAG

GTTAGTATATGTATAAATGCTAATTGATGTATTATCTGTCTCTAAATTTTTCATCATATACTAAACGTATTAATTGTGTTGAGGACTCCTTAAATCTCTGCCCCACACTCGAAATGAATAAGAAAGTCACGAGTTCTGATGTCTGAAAGCAGTAGGAATTGAATGAAAATCCTACTTCTATGTTTCTTTTCATATTCTTATGTATTCCATATTTTTCTTCACCTAATAGACAATAAAACTGATTCTCCCTCAGTCGGTGCACGTAATTAACACACAGTCATCAACATAAATAATCTCTAAGTCGAATTCTTCTTTGCAAATCAGTTTGTTTCTGTCATAACAAAAATAAATCTTATTTGAATTCAAGTATACAATAAATTATTATATGTCTGCATTACAAAATTATTGTCTAACTAAGTAGATGCAATTGATTTGTTTTGCAACAG

AATTTTAAGAACTTAAAGGAGATTGATGCAAGTGATTCGAAGTTTTTGGTTGAAACTCCTAATTTTTCAGAGGCTCCAAAGCTTCGACGATTGATTTTACGAAATTGTGGAAGACTAAACAAGGTTCATTCTTCAATAAATAGTCTCCATCGTCTAATTTTATTGGACATGGAAGGTTGTGTCAGTTTCAGAAGCTTCTCATTTCCTGTCACTTGCAAAAGTCTCAAAACTTTAGTTCTTTCGAACTGTGGTCTAGAGTTTTTTCCAGAGTTTGGATGTGTGATGGGATATTTGACTGAACTACACATTGATGGGACTTCCATAAATAAACTTTCTCCCTCAATTACAAATCTACTTGGCTTGGTTTTATTGAACCTGAGGAATTGTATTAGACTTTCTAGTCTTCCAACTGAAATTTGTAGGTTGAGTTCACTTAAAACTCTCATTCTGAATGGTTGCAAAAACTTGGACAAAATTCCACCATGTTTGAGGTATGTAAAGCATCTTGAGGAGCTTGACATTGGCGGAACATCCATAAGCACAATTCCTTTCTTGGAAAATCTAAGAATTTTGAACTGCGAAAGGCTGAAAAGCAATATTTGGCATTCTTTGGCTGGTTTGGCAGCACAGTATTTAAGGTCACTCAACGATTTAAATTTAAGTGATTGTAATCTTGTGGATGAAGACATTCCAAATGATCTTGAACTCTTTTCCTCATTGGAAATTCTAGATCTGAGCAGCAATCATTTTGAAAGACTGTCAGAAAGTATTAAACAACTTATTAACCTTAAAGTATTGTACTTGAATGATTGCAACAAGCTAAAGCAAGTACCCAAGCTTCCAAAAAGTATAAAGTATGTGGGAGGAGAGAAGTCCTTGGGCATGTTAAGAACTTCACAAGGCAAAGTGCTTTGTTTCTTCTTCATCTTTTTATTTTTCTATTTTTATTTTTCTTGGGTGTTTCCAATCCCAT

>Cucsa.237530

ATCCTACTATCGATCGTAATTAACTTTGAAATACTTCGATCTTGCAGCACTGAAGAAGTGGATCTTATCAAGGATATGGGGAAGCAGACTGATAATAAGCTAGTACTTTCTCACAAAACTAGTTTAGTTGGAATGGAGAATCAAGTGAAGAAAGTTTGTAATCTCCTAGATTTAGAACGATCCAAGGACATACTTTTTGTGGGGATATTTGGATCAAGTGGCATTGGTAAAACAACCATTGCTGAAGTTGTTTACAACACAATTATAGATGAATTCCAAAGTGGTTGTTTTCTTTACCTTTCTTCAAAGCAAAACAGTTTGGTCCCACTTCAGCATCAAATTCTTTCTCATCTTCTATCAAAAGAAACTAAAATTTGGGACGAAGATCATGGAGCACAACTGATTAAGCATCACATGAGTAATAGAAAAGTTGTTATTGTTCTTGACGGAGTTGATGAAAGAAACCAAATTGAAAAGTTAGTTGGAAGTCCGAATTGGTTTGCACCCGGAAGCAGAGTTATTATTACGGCTACAAATAGAGATGTTCTGCATCAACTCAATTATAGAGATCAAGTGCAAGAATACAAGGTGGAGTTACTTTCCCGCGAGAGTGCTTACTCACTGTTTTGCAAGAATGCATTTGGAGATGGCCCATCTGATAAAAATGATCTCTGCAGTGAAATTGTGGAAAAGGTTGGAAGACTTCCATTAGCTTTGAGAACCATTGGTTCCTATTTGCATAATAAGGACTTGGATGTATGGAATGAAACACTGAAGAGACTAGATGAAGAGGAACAAAATTACTTTGATACAATATTGAAGAGAAATGTGGAGAAAATATAA

>Cucsa.237540

ATGCAGAGTTCATCATCGTCTTCTTTGGATCGTCCTAAGATGAACTATGATGTGTTCATAAGCTTTAGAGGTAGAGATGTTCGTCACACTTTTGCAGGATATTTGTACGATGCTTTGAATCGTTTGGGGATAAAAGCTTTCCTGGACAACAAGAGGTTTCTAATTGGAGATGATCTTCATGACTTATTCAAAATAATCGATGAATCAAGATCAGCAATTGTTGTTCTTTCAGAAGACTATGCTTCTGCTAAATGGTGTTTGAGAGAGTTGACTAAGATAATGGATTCCATGGGAACCTCAATGGAGCGTGTCCTTCCTGTGTTTTATCATATTGATCCATCAATTGTTAAAGATCAATCTGGAACTTTTAAGACAAGTTTTGATGAACATGAAGCCAATGTTTTAAAGGAAATTGATAATCAAGAGAAGGAGAAGCGCTTGAAGGAACTCCAGAATTGGAAAAGTGCACTGAAGAAAATTGGCAATCACACTGGAGTTGTCATCACTAAGAACAG

GTAAATTTCACACGACTATTTGTTTTTTCTTAAAAACTATTATCTCTTTTGATTGTTTTGATTTGGTCTCTTGTCAATTTTGAATCTGCTCCCTATTCTCCTATGTATGTGTGAAGATAAGTTGAATATTTGTATTCGGTTGGCTTATTCGGATATGAGTTAGATATTTGTTGGTTGTTCCAAGAGACTCATATTAGCACGATCAATTTAGGATTAGAGAGAAAGTTATAAAATTATGAGATGACTAAATGTTAAAGAAATTTCAGCTATCATTTGTATTAAAATCATAATATCATATATGCATTGATCAACTTCTGAAAATTGGATATACTATTTACAGCATTCACTTAACATTAAGTTTAATTATATTTTGCAG

TTCTGAGGTAGATATAGTAAATAAAATTGCAAGTCAAATATTCGATGCATGGCGTCCTAAGTTGGAAGCATTGAATAAGAATTTAGTTGGAATGACATCCCGATTGCTCCATATGAACATGCATCTTGGTTTAGGATTAGACGATGTACGCTTCGTTGCGATAGTAGGAATGGGTGGTATTGGTAAAACAACTATTGCTCAAGTCGTTTTTGATTGCATTCTTTCAAAGTTTGAAGATTGCTGCTTTCTAACATTACCTGGAGGTGATTCAAAGCAAAGTTTAGTGTCATTACAACGGGAAATGCTTTCTCAAATTTTTCATAAAGAAGATTTTAGAATATGGCATGAGAATCATGGAGTAGAGATGATTAAAAATCGACTGAGTGGTAGAAAGGTTCTTATTGTTCTTGATGGCATCGAAGAGAGAAGGCAGTTAGAAATGTTGGCTGGAAGCATCGAGTGGTTTGGTCCTGGAAGCAGAATCATCATTACAACTAGAAATAAAGGATTATTGTGCCATCCTAATTATGATGAAATGAAAGTATACAATGTTGAAGAACTAGATCATGATAGTGCCCTTCAACTCTTTTTGAAGCATGCATTTGGTAGTAATCATCAAAACAATGACAGTTTCATGGATCTTAGTAATGAGATAGTTGAGAAGGCTAAAAGACTTCCATTAGCTTTAAGAGTGATTGGATCTTCTTTGTATGGTAAAGATATTACAGTATGGAGAGAAACGTTGAAGAGGCTGATCAAAGTGGATGAAAGAAATTTTTTTGATGTATTGAAAATAAGTTATGATGGATTAGGAGTTGAAAGCCAACAAGTTTTTCTTGACATTACATGTTTCTTCAATGGAAAAAATGAAGATAGAGTAATTGAAATATTAGAGAGTTTTGGTTATAGTCCTAATAGTGAAGTACAATTACTGATGCAAAGATGTTTAATTGAAGTTTCACACAAGAAAATATTGGTGCATGATTTAATTCTTGAAATGGGTCGAGAAATTGTGCGTAAGGAGTCCCTCACTCAAGCAGAAAAACAGAGTAGGATTTGGCTTCATGAAGATCTTTACTGCAGGTTTGCTGAAAAACAT

GTAAGAAAATTGGTAATTTTTGTACCTAATATTCTTCTATGCCTTCAAATCTTCCCTTGGGCCAAGCCCACTAATTCTAACAATTATTAATGTTCTTTTTTAATTATTGTTTGTTAG

GACTTGATGCATATTCAAGGGATAGTTTTAAGTTTGGCAAAAGAAATGGAAGAATCAATAGAATTGGATGCTGAATCCTTTTCAGAGATGACCAAACTAAGAATACTGGAAATCAGTAATGTGGAGCTCGATGAAGACATTGAATATCTCTCTCCACTCTTACGGATAATTAATTGGCTTGGCTATCCTTCGAAGAGTTTGCCCCCAACGTTTCAATCCCGCTATTTGTTTGAACTACTCTTGCCTCATAGTCACCTTTTACGAATTTGGGATGGAAAAAAG

GTTTGCTTTTTAAAAACCGTTTTTGTTTTTAGTACTTTTTAGTTTGTGTTTGATTTCAATCTGGTCCCTATAGTTTTATTTAATATACATTTTTTTTTTCTTAACAG

AGATTTCCAAAGCTGAAATTAATTGATGTTAGTAACTCAGAACACTTGAGGGTGACACCTGATTTTTCTGGGGTTCCAAATCTTGAGAGATTGGTTCTATGTAACTGTGTTAGACTGTGTGAGATTCATCCCTCCATCAATTCCCTCAACAAACTCATTTTACTGGATTTAGAGGGTTGTGGTGATCTTAAACATTTTCCAGCAAATATAAGATGTAAAAATCTCCAAACACTCAAACTTTCTGGTACAGGTCTTGAAATTTTTCCAGAGATAGGCCATATGGAACATTTGACTCATCTTCATCTTGATGGATCCAATATAACCCATTTTCATCCTTCAATTGGGTATCTAACTGGCTTAGTTTTCTTGGACCTATCCTCCTGTTTAGGTCTTTCTAGTCTTCCTTGTGAAATTGGTAACTTGAAGTCTTTGAAAACCCTCCTTTTGAAATATTGTAAAAAACTTGATAAAATCCCTCCAAGCTTAGCAAATGCAGAATCCTTGGAGACACTTTCTATTAGTGAAACCTCAATAACCCATGTTCCACCAAGCATTATTCATTGTTTAAAGAACCTAAAAACGTTAGATTGTGAAGGACTATCACATGGAATTTGGAAGTCATTGCTCCCCCAATTCAACATTAATCAAACAATAACCACTGGTTTGGGGTGCCTCAAAGCTCTAAATTTAATGGGTTGCAAACTTATGGATGAGGACATTCCTGAAGATCTCCATTGCTTTTCTTCATTAGAAACACTAGATCTCAGCTATAATAACTTCACAACACTCCCTGATAGTCTTAGCCACCTCAAGAAGTTAAAGACATTGAACCTGAATTGTTGCACTGAGCTTAAAGACTTACCAAAGCTTCCAGAAAGTTTGCAATATGTAGGAGGAATAGACTGCAGATCGATGTCAGAACGATATTATAACAAAATTTTGCTTATCCCTTCTAGTTCTGGGCACCAACTTTACCTTACTTTTATCATTCCTTCCAAGGATGTGGATGTAGAATGTGACATGAATGAGTTCCAACATTCGATATTTACTCGAAGATCATTTGAGGTATGCATAAAAACATTGAAATATACTGCCTTTTGA

>Cucsa.338190

ATGGTTGGACTTCTCGACAGTGTGGCCGGAAATCTGCTCGGAAGGATAATCGAAGCCGCCGACCGACTAGAGTTTCGTGCTATCCAAAGCGAATTGAAAAACCTCGAAACAGATGTGTTGAATCTTAAGGCCAGACTCCGAGACGCCGAGGAGAAGCAGGCTAGTAATTGTGAACTCAATGAACTGCTTAAAAACCTCAAAAATGTGTTTTCAAGGGCAGACATTGCAATTGAGGAATTGGAATGCGATTATTTGAAGTGGAGAGTGCAGAATCGAAAGAACGACGTTGACGATAAGGGATGCCAGTTCTCTTCTTGTTTCTCCTCCAATTTCCTCATTTCTCCATTTAATACCGGCAGTAAATTCCAGGAAGATCTTAAAATAATTACCTCCGAATTACGTTCGATTGAGAAAGCCATGTCTAAATTCTCTCTGGTTGAAGATGAAGATGAATATATAAAAAAATTGAAGGGTGAAATGACTTTGCGGACCTCCATTACTGGTTCGCATGCTTTCGCTAGGCTTCTGCGCTTGAGGAGAGAGGCGATTCTCTCTAATGTAGATTCCATTTTTGGTAGAGATAAAATACAAGAGAGTATCATTAAGGAACTTGTGAATGATGAACAAAAATCTCCCCGTATTCTTTCAATCCAAGGAGATGGAGGGATGGGAAAGACGGCTCTGGCCAAGTTAGTCTACAATGCAGACGAAGTGTTTGATCATTTTGACAAGAGAATGTGGGTATGCGTTTCTGAAGATTTTGATATCCGGAGAATCTTAAGGGAGGTTCTGATGTCTGCAACTGGAGAAAATGTTACCACTGTTGCCTTAACCGAAAGTCGTTTACGAATCCGGCTCCAGCGGTACTTTTTTGGCAAAAAAATCTTGCTTGTTTTGGATGATTTTGGGAATTTGGATCCCGAAAGAGTATCAGAACTGAAAAAAATCGTGAAGATGGGTGTTGGTGGCAGCAAGATAATGATAACCACTCGCAGCGATGAAACTCTAAATGTTGCTACGACACACAAGATTGACAAACTCGACGAGACGATATCTATGCAAATATTCGAAGATACATATGGAAGCGAAGGGCTTAGCGAAGGGCTTAGAGACGATTTGTATCTCAAAAACCTTGTGGCAGAATGTGGAGGAGCTCCTTTGGCAATCAAATGTTTGGCTGGACTGCTCTCTTCAAAACCGAGCGATGGTGCTAAGAGTCCAAATGTCAAGGACTTGAGTGAGAAATGGAAACAGGAAGAGGCAAACAACGGTGGTGGCGTTTTATGTGCACTAAGACTGAGTTATGATCTAATGCCATCTTATTTGAAACCTTGTTTTCTTTGCTTTTCAGTGTTGCCGAAAGATAATGTGTTCTTCTCATTTGAGCTAATCCAGTTATGGATGGCACAAGGAATCCTTCCTTCGGGTACCAAAGATAATCCTGAAGAAGTTGGGGAGAAATATTTCAAGGAATTTCGGGATCGCCGTTTACTCGTTGATGTTGAGGAGCACACTCTTGGATATTGGTTCAAAATCCATAGCCTTGTACATGATCTTGCAGTCCAAAAGGCTACGGAACAAAAGAACCTCGGAAATTTTCATATGCTTTCATTTGTCGATTGCGACAGCATCCCTTCGTCGACAAACTATGATAACACTCGTTTTATTTCCATTCCCGTGGTAGGAGGTGCGGGACCAAATATCAATAGTGACCTTTTCAAATGCATCACCCAGTTCAGGCAGCTAAGGTTTTTGTACTTGTGCAACTCTTCTCTGGAAGAAATTCCAACCTCCATCGACACGCTGAAACATTTGAGGTGTTTAGATTTGCGAGGGAGTCAACGTCTGAAGAGGTTGCCAGAATCAATTTGCAAACTACAGAGCCTACAGACTTTGGTTCTTGCATTCTGCTCAGAGCTTGAAGAGCTTCCCAGAAACATAAAGAACTTGATCAGCCTCAGATTCTTATGGATACAAACAAAGCAAGCCCGCTTGGAAAAAGATGAAATAGGAAGCTTAACATCCCTTCGTTTTCTCGCCATTGGAAGGAGTGAAAACTTGACTCACTTGTTTGAAGATATCAACAAACTCAATTCCCTCAAAACACTGATCATTTATGAGTGCAAATCGCTGCTAACACTGCCAAAAGGCTTGGAAAACATGAAATCTATATGTAATATGGGAATATGGGAATGTGATCGGCTGAGATTTACATTCTCACTGGCTTCACTTCACCTCAAGAAACTGATACTCAGAGAACTTACAGCAGTGTCCACTTTGCCTAATTGGCTGTCCAATTTGGATGGTACTTTAGAAGTGCTAGAAATTGGAGAGTTCCCCACGCTAAGAAAATTGCCAATCTGGCTTTTAAACTTTTGGGAACTCCGAATTCTTGGGATCTCCAACTGTCCTAAGTTGAAGCATGATTCCTTCCCTCCTGAGCTAAATTATTTTTGTGATAAGATTGAGGAGTTGAGGATCACATTTTGTGGGTCTTTGAGCAAGTCTTTGTTGAAAAAAAGCATGAAGGAAATTGAACCTGAAAGCCGGGTAATCTTTTACATCCATACCATTTATGTGGACTCCAAAAGAATGACGCCACCAGTAGAATCAACAGACGAACCTAAGGAAGCAGAGACAAAACAGGATGATGCTTATAACAATGCAAGTCCTCCTGGGACTGAACAACCTTCAAAGACTAAACATGATGATGCGAATAACAATATGAGTCATCCTGGGATTGGACTACTTTCAGAGTCAAAACAGGAGCATACAAATAACAATATAAACGAGATTGAGACTGTTAAGGTTTGTTTGGGTGATAATGACCATGCTGAAGCTCACCAAGCTATG?

>Cucsa.337180

ATGTACCTGAGACGAATGCAGTATACCATGTTGAGCCTTAAAACAATTCTTAAGGATGCTGAAAAGGAAGAATATCGTCATTGTCTAAATGATTGGCTACAGAAGCTTCAAAGTGTATTTTTACAAATTGAGGAATTGCTATATGAATCCAATAGGGAAGTCAAAAAACAAGAGGCTACTGGAAAATGGGTATTTCTTCCTTCCTTTAACTTCAGTCAAATTGATCAAACTAAAAAAATGATGAAACTATGCGACGATTTGGATGAAATTGCATCCCATATGTATGGCTTCAATCTAACAAACATGGAGACAACACACTCCTTTCTTAGTGCTACTGAAGTTTCGACAAGACTCATGAAACCAAGTTGGCAATTGCTTTACTCGTTGACTAATGCTCCCAAGGTTTTCCAAGACAAACGATATCATAACTTTCTGGATCATTTCAAAAAATCTACTCACGGGCTCTTCCACATAGTTGGAGAACCAGGTATAGGTAAGACCACACTTGCCAAATTCTTTTACAACAATCTGGTGAACACGTTTCCATCAAGATTGTGGATTTGTGTGAAAGAGGAATTTGATCCACAGAGATTGATAAAAGAGATGCTCAGTTTTTCACATTGCCAAGTAACATGTGATAACTTGACTGAGAAACAATTGTGCTTTGCAGTTCAACAATTTCTGAGGGATAAAAAATTTCTGATTGTTTTTCAAGATATTTCAATCAAAAACCTTGGTAATTGCTCCATATTTAAAAGTTTATTGGGGATGGGAAACCGTGGCAGCAAAATCATAGTGACCACTCAGAATGAGAAAATAGCTGATGCTGTCGGACTAAAAAAACTCTACAAGAACGAGAGCCAGGTAGTTCCATCCCCAGAAGCCACAAAACCTTCAGATGTTAACAAGGATAATATGAAACATCAAACAATTTTCAAAGTTGAGAGGTTGTCAAAGGAAAATTCATTGTCTTTGTTCAAAGTTCATGCTTTCACAGAAACACAGGAAGCACAAATCCCAAATCTCACAAAAATACAAGAAGTAATTGAGCAGAAATGTCATGGGGTTCCTTTGGCAATAAAGTGCCTGGGGGGTCTGCTATCAAAAACTAGTATAGCTGAGTGGAACGGTGTCATCGATAAGTTATGGGAACATGAGGAAGAGGAGGATGGGAATAAGAGTATTTTACCTACACTTAGATTATGCTATGATCAAATGCCTTCACACCTACAACGTTGTTTTCTTTATTGTTCCCAATTAAAAAAAGATCGCATATTGTCTTCCAATGATGTGATTCAATTATGGATTGCAAGCGACCTCCTACCCAAAGAGAATTACTTATCTTTGGAAAAAATAGGTGAGAATTATTTCAAGGAACTATGCTCAAGATGTTTCCTACAAGAACTAGAGGAATATGGTTTTGGCTATTGGTTTAAATTGCACCCTCTTATTGAAAAACTTGCACGTCTACTCACACAAAAACAGGTATTCGAAGTCACAAAAACCCAATCTATAGCCTTCACAATAAGAGATAAGGTGCCCCCTAGTGCATTCCTAGCAAATGCATGCATCGACAAGTTCAAATACTTAAGACTATTGCATTTAGGCAATGCAAATCTACAGGGAATTCCAAGTGCTGTAGAAAATCTGGTACAGCTCAGATACCTAGACTTGCAAGGGAATAAGAAAATCAAGCGGCTACCAAATTCAATCTTCAAGCTAAAAAATTTACAAACCTTGATTCTTGCATCCTGTTCCGCACTTAAAGAACTGCCCAATGATATTAGGCAATTGACCAACCTGAGATACCTCTGGGTAACAGCAAACAACCTTCGTCTGCACAAAAATGGAGTTGGAACCATGACTTCTCTTCGATTTCTCGCAATTGGAGGGTGCCAAAACCTACAAGATCTATTCAAAAAGCCGTCATGCCTCGTACGCCTAGAAACCCTAATGATTTACGATTGTAAGACATTGAAATCGTTGCCAAACGAGATAGGATCGCTAATATCACTAAAGAATTTGGTGATTTGGAGTTGCAAAAAACTTACACTGACGTTGAAAGGAGTGGAGTTCAGGCTTCAGAGGTTCACAATCAGAGAGCTTCCAATAGTGAAAAAATTGCCGGAATGGACTCAAAGATTCACCGAAACCCTAAGAGTTTTGGAAATCATCGATTGTCCCATCGAATGGAATGATGATGTGTTAAAATCATACAAATCACTTGAACGGTTTTCAATTCATGGAGCTGTGAGGACCAAAAACCAGATCGGGGGGTACAACATCGATTATCGTAATTTCGTTAGGAGTAGGAAAGTCAAGAAGGAAGTGAAGACATGTGTCTACTACTAATTCATCAAATTTGAGCTTCAAAAAGGAAAACGAAAATATGTTCATATTAAGTGGAAGGTTTGA

>Cucsa.337190

ATGGCTTATTGCATCTATTACCGAGCTGAAAACATCTTGAGTGAACTGAAAAACCTCCCAAACTACCCAAGAAGAATTGAGTATACCATGTTGAGTCTTAAATCAATTCTTATGGATGCGGAAGAGAAGCAAGAACAGAGTCGTGGTCTACAGAATTGGCTAGAGGAGCTTCAAAATGTATTTTCCCAAATTGAAGGCTTCATAGATGAACACAAAGAGGAAGCCTACGAGGGTATTGGTAAACAGGTACTTGCTCCTTTCTCGTGCTCCAGTAATCAAATAGCACGTACTTGGAAAATGGAGAAACTATTCGACCATTTGAATGAAGTTGCGGCAAAAATGTATGAATTTAATCTTACAGAAAGGCACACTGGTGCCATAAAAACGGAGACAACAAACTCTTTCCTTACTGCTACTGAAGTTTCAACAAGACTCATGAAACCAAGCTGGAAAGTACTTTACCCCTTAACTAATGCTCCGAAGTTTTATCAGGATGAGCGGTACCGTAAGATTCTGAATGATTTCAAAAACCCTACTCTAGGGTTCTTCCACATAGTTGGAGAAGCAGGTATAGGTAAGAGCACACTTGCCAAATTCATTTACAATGATCCAGAAGTAGAAGGAATGTTTCCATCAAGATTGTGGGTTTGTGTGAAAGAGGAATTTGATACACAGAGATTGATGAAAGAGATACTCAACTTTTCATATTCTCCAGCAACTTGTGACAATTTGACTACAAAATTGTGCCCCACAGATCAATATCTGAGAGAGAGAACTTTTCTGCTTGTTTTTCAAGACCTTTCAATCAAGAACCTAGATAATTGTTCCCTGTTTACAAGTTTATTGATGATGGGAAAGCCTGGTAGCAAAATCATAGTGACCACTCAGAATGAGGAAATTGCAAATGCTATAGAACTAACAATGATTTACAAGGTTGGGCAACAATCAGAGCAAAATCGGAGCCAGACAGCCCTAGACACGGTTACTAAAGAGACTGCAAATGTTAACAACGCCGACCAGTTCGTTCAAGCTAACCCTTTGGGTAAGATAGATCAATCTATCCCTTCTCAAACAATATTCAAAGTTAAGAGGCTGTCAGAAAAAGATTCCCTTTCTTTATTCAAAGATTATGCTTCTACATATGAAGGTAATGAAAAAGATATAATGAAAACTCTGAAGAAATGTAATGGAATACCATTGGCAATAAAGTGTCTGGGGAGCATGTTATCTCTAGGTCCTCCAGCAACTAAATGGATGGAGGATAATGAGCGACAAAAGGGAGATAATGAGTCTTCTAGTACATTTAGTATACTTAAACTATGCTACAATGAGATGCCCTCACACCTGAAGCGTTGTTTTCTTTATTGTTCTCAATTACCAAACGATAGCATACTGTCCTCAAATGATGTCATTCAGTTATGGATGGCAAATGGACTTCTCCGTTCACGCCAAGAGAATTACTTATCCTTGGAAGACATAGGTGAGATTTATTTCAAAGAACTATGCTCAAGATGTTTCCTTCAAGATGTTGAGGAATATGGTCTTGGCTATTGGTTTAAAATGCACCCTCTCATTCGGGAACTTGCACGCCTCGTGCAAAAACGAACTAAGGACTTGATAAGCATTAAACCAGTCACCAATGTCACATCTATAGCCTTCCCAGTAAGAGATGAGGTGCCATCTAGTTCATTTCTAGCTGAAAAATGCATCTCAAAGTTCCAACACTTAAGATTATTGTATTTAGGCCACACAGATCTACAGGAAATTCCAAATACTATAGAAACACTGAATCACCTAACATACCTCGACTTGCAGGGGAATAAGAACATCAAGCGGTTACCAAATGCAATCTGTAATCTACAACATTTGCAGACCTTGATTCTTGCATCTTGTTCTGCACTTGAAGAATTGCCAAAAGATATATGCAAGTTGAGCAACCTCAGATACCTGTGGGTAACATCAAACAAGCTTCGTTTGCACAAAAATGGAGTAGGAACCATGACTTCTCTAAGATTCCTCGCAATTGGAGGATGTGACAAACTTCAAGATCTATTCGAACGGCCATCATGCCTTGTACGCCTTGAAACCCTAATGATTTACGATTGTAACTCTTTGCAATTGTTGCCAAACGAGATGGGGTCTCTAATATCGTTACAGAATTTGGTGATATGGAGTTGCAAACAACTTACACTGAAGGGCTTAGAGAAAGTCGATTTCAGCCTCCAAAGATTCACAATCAGAGAGCTTCCAGAAGTGAATAAATTGCCTGAATGGCTTCAAAGGTCGACAGAAACCCTAAGAGTCCTGGAAATCATCGATTGTCCCATCAAAGTGGAGGAAGAGGGAATCAAAATGTACAAAGCAGTTGAAAGTAAGATAATTCAAGGAGCTGTAGACATCACTGGGAATTTGGTACGGCGTAGTCCAATGGTAACGAAGAAAGTACAGATGACAGGTAACTTCTATTAA

>Cucsa.128030

ATGGCCGAAGCAATTCTCTTCCAAGTTGCTGGGGAGATCTTGATGAAGCTAAGCTCTCAAGCTTTCCAGCGTCTTGGGATGCTATTTGGGCTGAAGGGTGATCTTAACAAACTCACAACAACTGTTTCCACCATTAAGGATGTGCTTCTTGATGCGGAGGGACGTCAAACTAAAAGTCACTTGCTGCAAAATTGGCTCCATAAGCTGGAAGAAGCTCTTTATGATGCAGAGGATGTGCTTGATGAACTCTCTACGGAGGCTCTCCGTCGAGAACTGATGACTAGAGATCATAAAAATGCAAAACAAGTAAGGATCTTCTTCTCCAAATCTAATCAAATTGCATTTAATTATAGGATGGCTCGTCAAATAAAGAATATTTGGGAGAGGCTAGATGCTATTGATGCTGAAAAAACACAATTTCACTTGCGTGAAAACTGTGAATCACGGACTCAATACGGTTCATTTGATCGAATAATGATGGGAAGGGAAACTTGGTCTTCTTCAAATGACGAGGAAGTGATTGGAAGGGATGATGATATAAAAGAAGTAAAAGAGCGTTTATTGGATATGAATATGAATGTCACGCATAATGTTTCGTTCATTGCTATAGCTGGAATGGGTGGGATAGGCAAGACGACCTTGGCTAAATCTCTCTACAATGACGAAGAGGTATCAGGATTTTTCGACTTAAAAATATGGGTTTGGGTTTCTGATCAATTTGAGGTACAAGTGGTAGCGGAAAAAATGATAGAATCAGCAACCAAAAACAATCCTAGTGTAAAAGGAATGGAAGCTTTACAAGCAAAGCTTCAGAAAGTGATTGGAGAAAGGAAGTATCTGTTAGTTATGGATGATGTATGGAATGAAAGTGAAGAGAAATGGCATGGGTTGAAATCATTGTTGATGGGTGGTGCAAGAGGGAGTAAGGTTTTGATCACAAAGCGTGACAGAAAAGTAGCCACAGAAATCAAAAGCATGACATCTTTGTTCACTTTAGAAGGCTTATCAGAGAGTAATTCCTGGTTATTGTTTAGTAAAGTGGCATTTAAAGAAGGCAAAGAGTCCACAGATCCAAGCACGATACATTTAGGAAAAGAAATTTTAGTGAGATGTGGAGGTGTTCCTCTTGTTATAAGACATGTTGGACGCATGTTATACTCTAAAACTTCACAAGAAGAGTGGATGTCCTTCAAGGATAATGAACTTTTAGAAGTCATTCAACAAGACAATGATATGACATCAATATTAAAATTGAGTTATAACCATCTCCCACCAAATTTGAAACGATGTTTTGCATATTCATCCCTGTTTCCCAAAGGATATAAAATAGAAATAAAAGACCTAATAAGGCAATGGGTGGCTCAAGGTTTTATTGAAGTGTCAAATGGAAGAAAATCCTTGGAAGATACAGGGAAGGACTATTTTAACGAATTATGTTGGAGGTTTTTTTATGCAAATTCTAGTGATGAGTGTAACATCAATGATATTGTTTGTATGCATGATGTGATGTGTGAGTTTGTAAGGAAGGTGGCAGGAAATAAATTATATGTACGTGGAAATCCCAATAATGATTATGTTGTCAGCGAACAAACACTTCACATTTCATTTGACTACGGAATACAATCATGGCAAGATGTTCTATCTAAATTATGCAAGGCTAAGGGATTAAGAACAATCCTTTTATTATTTCGTCCCTACGAGAAAATGAATAAAATTGATAAAGCTATTTTGGATGAATTATTTTCCAGTTTTCCACGTTTGCGAGTATTAGATCTTCATTTCTCGCAGATTTCTGTAGTGCCGAAGTCTATAAAAAAACTTAGACACCTTCGATATTTGGATCTCTCTGAAAATGATATGGAATTAATTCCACATTCTATCATTGAATTGCAAAATTTGCAAACACTAAATCTAACAGAATGCTATGAGCTAAAAGAATTGCCAAGGGACATCGACAATCTTGTAAATCTCAGGCATCTTACCTTTGAACCTTGTATGGAAGTAACTCCTACATCGGAGGGGATGGAGAAGTTGACTTGTCTACAAACAATCAGTTTATTTGTGTTTGACTGCAAAAAGACCAATAAGCTATGGGAATTGAATGATCTCAGTTATTTGACAGGAGAGTTAAAAATCATAGGTTTAGAGAAGTTGAGGTCTTCTCCATCTGAAATCACCTTAATAAACCTGAAAGACAAAAAAGGTTGGCAAGGTTTAAATTTGGAATGGAAACTGGGCAAGGATGAATACGAAGGTGAGGCTGATGAAACAATAATGGAAGGCTTGGAACCACATCCAAATGTTGAATCGTTGAGCATTAACGGGTACACTGGAGGAGCATTGCCCAATTGGGTGTTCAACTCGCTTATGAAGTTAACTGAAATTGAAATTGAAAATTGCCCTAGAGTGCAACATCTACCTCAGTTCAACCAGCTTCAGGATCTCAGAGCTCTACATTTAGTGGGCTTAAGATCTCTCGAGTTCATAGATAAGAGTGATCCATACTCATCATCAGTGTTTTTTCCATCTCTCAAGTTTCTACGTTTAGAAGATATGCCTAATTTGGAAGGATGGTGGGAATTAGGGGAATCAAAAGTAGTAGCAAGGGAGACATCTGGGAAAGCTAAATGGTTGCCTCCAACTTTTCCTCAAGTTAATTTCCTACGTATCTACGGATGTCCGAAGTTAAGTTCCATGCCCAAGCTAGCTTCTATTGGAGCAGATGTTATTTTACATGATATTGGGGTTCAGATGGTGAGTACCATAGGTCCAGTATCGAGTTTTATGTTTCTATCAATGCATGGAATGACGAATCTCAAATATTTATGGGAGGAATTTCAGCAAGATCTAGTTTCTTCAAGTACCTCAACAATGTCCTCACCTATTTCCCTTCGTTATCTGACAATAAGTGGATGCCCCTATCTCATGAGTTTACCGGAATGGATTGGCGTTCTCACTTCCCTTGAAACATTGCATATTAAAGAATGTCCAAAATTAAAATCACTACCAGAAGGAATGCAGCAACTCAAATCTTTGAAAGAACTTCACATAGAAGACTGCCCTGAACTAGAGGACAGATGCAAGCAGGGAGGAGAGGATTGGCCAAACATTTCCCACGTTCCCAACTTTACTTACAAAAATGCCTCTGACATTGACACACCACAATCTTCTTCAGGTTTTTCACACCATCCCTTTTCAATCGTTCGTATCTCTGTTATATAG

>Cucsa.128100

ATGGCGGATTCAGTTCTGTTCAATGTTGCTGCAAGTGTTATTACTAAACTGGGATCTTCCGCACTTCGAGAACTTGGGTCTCTGTGGGGAGTCAACGATGAGCTCGACAAACTCCAAAACACTCTTTCGGCCATTAAAGCCGTCCTTCTCGATGCAGAGGAGCAACAGTCCAAGAGCCACACAGTCAAGGATTGGATTGCAAAGATCAAAGATGTTTTCTATGACATTGATGACTTGATTGACGAGTTCTCTTATGAAACCTTGAGAAGACAAGTTCTTACCAAGGATAGAACAATCACCAAACAAGTACGTATCTTCTTCTCCAAATCTAATCAGATTGCTTTTGGTTTCAAAATGGGTCAAACAATTAAAAAAGTTAGGGAGAAGCTAGATGCTATTGCGGCTATTAAAGCTCAACTTCACCTCTCTGTGTGTGCGAGGGAGGTACGAGATAATGAGCCAAGGAAGGTACGAGAGACGTCCTCATTCATACCCGAGGGGGAAATCATTGGTAGGGATGAGGATAGGAAATCTGTTATGGATTTTCTATTGAATACCAGCAACATCACAAAGGATAACGTTGAAGTTGTTTCCATTGTTGGAATGGGAGGATTAGGAAAGACAGCACTCGCTCAAACTGTCTATAATGATGAAAAAATAAACAATCGTTTTAAGTGGAAAATATGGGTGTGTATTTCTCAAGAATTTGATATCAAAGTAATTGTTGAAAAGATTTTAGAGTCTATTACGAAAACAAAACAAGAATCCCTTCAGTTGGATATATTACAAAGTATGCTTCAAGAGAAAATTTATGGAAAAAAATACTTGTTGGTCATGGATGATGTGTGGAATGTAGACCACGAGAAATGGATTGGTCTGAAAAGATTTCTGATGGGTGGTGCCAGCGGAAGTAAGATTTTGGTGACAACCCGTAATCTACAAACTGCCCAGGCTTCTGACACGGTTTGGTTCCATCACTTAAAAGAACTTGACAAGGATAACTCTTGGGCGTTGTTTAGGAAAATGGCATTCTTAAACAAAGAAGAAGAGCTTGAGAATTCAAATTTGGTTAGAATCGGTAAAGAGATTGTAGCAAAGTTGAAAGGTTATCCCCTTTCAATAAGAGTTGTTGGACGTCTGTTATATTTCAAAAACACAGAAATGGATTGGTCATCATTTAAGGACAACGAACTTGACTCAATTTTGCAAGAAGATGATCAAATTCAACCAATACTGAAGATAAGTTTTAACCACCTTCCACCTAAATTGAAGCAATGTTTTACGTACTGTGCTTTGTTTCCCAAGGATTATGAGTTTAAAAAGAATGGATTGGTAAAACAATGGATGGCACAAGGTTTCATTCAAGCACATAATAAAAAGGCAATTGAAGATGTGGGTGACGATTATTTTCAAGAGTTAGTGGGGAGGTCATTCTTTCAAGACATAAGAAAAAACAAATGGGGAGACTTAAAGTACTGTAAGATGCATGATTTGTTACATGATCTTGCGTGTTCGATAGGAGAAAATGAATGTGTGGTTGTAAGTGATGATGTCGGGTCCATTGACAAAAGGACTCGACATGCCTCATTTCTCTTGAGCAAGAGGCTAACAAGGGAAGTTGTATCAAAATCATCCATTGAGGTAACGAGTTTGAGAACATTGGATATTGATAGTCGTGCTTCTTTCCGTTCTTTCAAGAAAACTTGTCACATGAACCTTTTTCAATTACGAACATTGAATTTGGATAGATGCTGCTGTCATCCTCCTAAGTTTGTTGATAAGTTGAAACATTTGAGATATCTTAATCTTTCTGGTTTAAATGTAACTTTCCTTCCCAATTCTATTACCACATTGTATAATTTGGAAACACTTATCCTTCGTTACTGCCTTTGGCTAAGAAAATTGCCAAAAGATATTAACAATTTGATCAATCTCAGGCATCTTGATATTTATGATTGTTCCAGTTTGACTCACATGCCAAAAGGATTAGGTGGGATGACTAGCCTTCAGACAATGAGTATGTTTGTATTAGGAAAGAATAAAGGTGGTGATTTAAGTGCATTGAATGGACTTAAAAGCTTGAGAGGATTATTATGTATTAAAGGTTTACAATTTTGCACAACTGCTGATTTAAAAAATGTAAGCTACTTAAAAGAAATGTATGGAATTCAAAAGCTGGAATTACACTGGGATATAAAAATGGATCATGAAGATGCCTTAGATGATGGTGATAATGATGATGAGGGAGTTTTGGAGGGCTTAAAACCACATTCAAATATTCGCAAAATGATTATAAAAGGATACAGAGGAATGAAGTTATGTGATTGGTTTTCTTCTAATTTCCTGGGTGGTTTGGTTAGCATAGAGCTTTCACATTGTGAAAAATTGGAGCATCTCCCACAGTTTGATCAATTCCTATATCTCAAGCATCTTCTTCTTGGATACTTACCCAATATTGAATACATTGATAGCGGCAATTCTGTTTCTTCATCAACAACATTTTTTCCATCTCTCGAGAAGCTAAGGATTGAGAGCATGCCTAAGTTGAAAGGGTGGTGGAAGGGGGAAATTTCATTTCCAACAACAATATTACATCAACTCTCAGAATTATGTATTTTTTATTGTCCTCTGTTGGCTTCTATTCCACAACATCCATCTTTGGAATCATTGAGAATATGTGGTGTTAGTGTGCAACTTTTTCAAATGGTAATACGAATGGCTACAGACCTTTCTGAACATTCTTCTTCTTCTTCAACATTGTCTAAATTATCTTTCCTTGAGATTGGAACTATTGATCTTGAGTTCTTGCCAGTGGAGTTATTCTGCAATATGACACATCTTGAGTCTCTTATCATAGAACGCTGCAAAAGTTTACAAATGTCTTCTCCGCATCCTGTTGATGAGGATAATGATGTGGTATGGAAAAAACTCAGCAATCTCCGGACACTTCGGCTTGAGAGCATCCTCAAATTGGAGTATTTTCCCAAGAGTTTGAAATATATTACAAGTCTTGAAACTTTGAAGCTATCAAATTGTGAAAATTTAGTGAGTACGGAAGGGATTGGCGAACTCATTTCACTATCACATTTGGAAATTGATAGATGTCCTAATTTACCTATATTGTCGGAAGATGTCGGCGACCTCATTTCCCTATCACACTTGCTTATTTGGAATTGTCCCAAATTAACTTCCTTGTCAGAAGGAATCACTCGCCTCACTTCACTCTCAAGTTTGTGTCTTGAAGATTGTCCCAACTTAGTCTCCTTGCCCCAAGAATTTCTCCACCACCACAGCTCCTTACCAGGAGGACGGTTCTTGAGAATTTTGAACTGTCCCAAATTGCAGATTCAAGACAAGAAACAAAAGGAAGAAGAAGAAGAAGACCAGGAGGATTGGAATGAACTCATCCATGTATTGACCGGATGTAGGTAA

>Cucsa.128130

ATGGCACTAGAGGGAATTCTTTCGGGTGTTGGAGTGGAGATCTTGAAGAAGCTTAGCTCTCATGCTTTAGAATGCCTTGGAATGGTATGTGGTCTTAATGATGATCTTAACAAACTAAGGAGCAATGTTTCGTCCATTCAATCTGTACTTCGTGATGCAGAGCAACGTCAAATCAAAGGCAATGATCATTCTTTAACCGATTGGCTCGAAAAGTTGGGAGACGTTTTTTACGATGTTGAGGACGTTCTTGATGAAATCTCTACTGAGGCTCTCCGTCGAGAAGTGATGACAAGAGGAAAAAATGCAAAGCAGGTTAGAATCTTCTTCTCCAATTCTAACCAACTTGCATTTAACTATAGGATGGCATGTCAAGTCAAGAAAATTAATGAGAGGCTTGATGTTATTTCCCAAGAAAAAGATAAGTTTCAGCTCAATGGAATTGCTTATCTTGGGATACAAAATGTTTTATCTTATCCAATTGGGATGGAAAGGGATACTCACTCATCTTTAAGTGGGGATCAGAAAATAATTGGAAGGGATGATGAAATGAACAACCTTAAAAAAAATTTACTAGCAGAGGATGACAAGGTGAAAGCTAACGTTTCATTCATCGCTATTGTTGGAATGGGTGGAATTGGCAAGACAACTTTGGCCAAATCTCTCTACAATGACAAACAAGTCTCTGATGGTTTTAGTTCAAGAATTTGGATTTGGGTTTCTAATCAATTTGACACAAAAACAATATTGAAAAAGATAATTGAATCAGCAACCGAAAAGAAACCAAAGGTAGAAGAAATGGAACCTTTAAAGACAAAGCTTGAAGAAGTGATTGGAGGAAAGAAGTATTTGTTAGTTATGGATGATGTATGGAATGAAAATGAAAATGAATGGGAGAATTTGAAAAACCTGTTAATGCTTGGTGCAAGAGGGAGTAAGGTTTTGATCACAAAGCGTGACAGTAAAGCAGTTCCAGGAGTCGAAACAATTCCTCTAAAAGACTTAACTGAAGATTTTTCTTGGTTGTTGTTTAAAGAAGTGGCATTTAAAGAAAGTGACTTAGAGTCAATAAATCAAAACTTGATAAAAATGGGAAAAGAAATTTCAAAAAGATGTGGAGGTATTCCTCTTGTAATAAGACACATAGGACGTTTATTATATGGAAAAACTTCCGCAGAAGATTGGGAGTTCATCAAAGAAAATGAACTTTTAAATGTCACTCGTGAAAAAAATAATAATGATGGTCATGTGATATCAACATTAAAATTGAGCTATAACCATTTGTCACCAAATTTGAAGCAATGTTTTTCCTATTCATCCTTGTTTCCCAAAGGATACAAAATTAGAATGAATGAATTGATTAGACAATGGATAGCTCAAGGTTTTATCGAATCATCAAATGGAGGAAAATCTGTAGAGAATATTGGGAAGGAGTACTTGGATGAATTATGTTGGAGGTTTTTCTATGAAATTTCTATTGAGGATGTTCCTTTTGAAGAAGTTGGCATGCATGATTTGATGTGTGATCTTGCAAGAGAGGTAGCTGGACAGAAATTGTACATACGTGGATATCCAGAGAGTGGATATGTTGTGAGTGAACAAACTCGTCATATTTCATTTGAATATGAACCACGATCATGGATTGATGATGTGTCCAAATTGCAACAAGCTAAAGGATTAAGAACGTTCCTTTTGTTTACGAAAAATCCTTTCTTTACGAGAAATCCAATTGAAAAAGTTCTTTTGGACAGACTGTTTTCTCACTTTCCACGTTTGCGAGTATTACAAATCCCTAATGTGTCAAAGTCAATAAAAAAGCTTAGACATCTTCGATATCTAGAACTCGGTGAAGATGCGAAATCAGTTCCAAACTCCATCACGAAATTGCAAAATTTGCAAACACTAGATCTAACCAAGTGTTATGACCTAAAGGAGTTGCCAAGGGATATTAACAATTTTGTAAACCTCAGACATCTTCTTTGTGATTCAAGATTAATGAATATGCTGCAAGGGACGATGGAGAAGTTGACTAGTCTACAAACATTAAGTTCATTTTTGTTTGATTGTAAAAGGTTTGATAAGGTAAAGGAATTCAGTGAGCGGAGTTATTTTATAGAATTTGACTTAAAAATCAAAGGTTTGGAGCAGTTGAGGTTTTCTCCATCAGACGTCAAATCAGTAAATCTTAAAAACAAAAAAGTCCCACTTTTGAGACTGAAATGGAAATTTGAGAATGGTAATGAATATGAAGGTGATGCTGATGATATAGTATTGGAAGGCTTAGAACCACATCCATATGTTAATCTCTTGCAAATTGAAGGGTATTGTGGAGTAGGATTACCCAATTGGGTGTCCACCTCAATTTTGTTAAGGGGAATTCGAATTGGTAATTGTGATAGATTACATCTGAATCAACTCTCCCATCTTCATGCTCTTGAAATTCTAAATTTAGAGGGTTTAAAATCTGTCATGAGTATATCGGAATGGATTGGCACCCTTACTTCTCTTGTATCTTTGGAAATAGAAGAATGTCCAAAATTAAAATCACTTCCAAAGGAAATGCAACAGCTCAAATCTTTGGTGCAACTTAACATAATCAAGTGCCCACAACTTGGGGAGAGATGCAAGGAGGGAGGAGAGGATTGGCCTAACATTTCCCATATTCCTGACGTTCTTATTGATTGA

>Cucsa.128140

ATGGCTGAAGCTATTCTCTACAACGTTACTGCAGACATCATATTCAAATTGGGCTCTTCCGCACTACAGGAGCTTGGGTTGTTGTGGGGTGTCAATGATGAACTCGACAAACTCAAACACTCGCTTTCTGCCATTCAAGCTGTGCTTCTCGATGCGGAGGAGCAGCAGTCCAAGAGCCTTGCTGTCAAGGCTTGGGTTTCAAGGCTTAAGGATGCTTTGTACGAGATTGATGACCTGGTGGACGAGTCCTCCTACGAAACCTTAAGAAGGCAGGTTTTGGCCAAAGATCAGAGAAAAAGAAAACTAGTACGTATCCTCTTTTCCAAATTTAAATCTAATTGGAAAATAGATCACAAAATCAAGGATATTAGACAGAGGCTACAATCTATTAATGATGACAAAAATCAATTTAGCTTTTCTGAGCATGTGATCGAGAAAAGAGATGATGAAGAGTTGAGAAAGAGACGGGAGACTTACTCTTACATACTTGAAGAGGAAGTGATCGGTAGGAATGATGACAAGGAAGTAGTCATAGATCTTCTATTAAATTCCAACATCACAGAGGATATTGCAATTGTTTCCATTGTTGGAATGGGAGGACTGGGAAAGACTGCCCTTGCTCAGTCTATTTATACCCATCACAATATGACTAATAGTGGGTTTGAATTGAAGTTATGGGTGTGTGTTTCTGAAGAATTTGATCTAAAAGTTATTATCCAAAAGATGATAGAGTCTGCAACTGGGACGAAGCCTAAGCCGTACCTTCAAATAGATTCATTACAAAGTGAGCTTAGAAAGAAAATCGATGGAAAGAAATACTTATTCGTAATGGATGATGTGTGGAATGAGAAAAAAGAGGAATGGTTACGCCTTAAAAGATTATTGATGGGCGGTGCAAAGGGTAGTAGGATTTTGATCACAACACGGAGTGAACAAGTTGCTAAAACTTTTGACTCTACTTTCATCCATTTTTTACAAATTTTGGATGAGTACAATTCCTGGTTATTGTTTCAAAAAATTACTTGTTTGGAAGGACATCCAAGTAATCCAGAGAAGCTTGATCAAAGTTCAAGTTTGATACAAATTGGCAGGGAAATCGTTTCAAAGCTAAAAGGTGTTCCTCTCACGATAAGAACCATCGGAGGACTTTTAAAAGACAATAAATCAAAAAGAGTTTGGTTGTCTTTCAAAGATAATGAACTTCATCGAATTTTGGGGCAAGGACAAGATAATCTAAAAGAAGTGCGATTAATTCTTGAACTCAGCTATAAATACCTTCCAGCTAATTTGAAGCAATGTTTCCTATACTGTGCTTTGTTCCCGAAAGATTATGAAATTAAAACACATGAACTTATACTAATGTGGAGTGCGCAAGGTTTCATTCAACCAAATGGCAGCAAGGACAACAGCCTCATTGATATTGGCAATGATTATTTCATGGAGTTATTATCACGATCATTTTTTCAAGAGGTTACAAAAAATGAACGGGGAGACATAATAGCATGTAAGATGCATGATTTGATGCATGATCTTGCTTGTTGGATAGCAGATAATGAATGCAATGTCATCAACATAGGAACTCGTCACTTTGCATGGAAAGATCAATATTCTCATAAAGATCAACTTCTAAGATCATTATCAAAGGTGACAAATTTGAGAACATTTTTCATGCTAGATTCTGCAAATGATTTGAAATGGGAATTTACAAAAATACTTCATGATCATTTGCAATTACGAGCCTTGTATTTCAAAAATTTGAAGAATGCAATGATCGTTTTGGAGTTTACTGGTAAGTTGAAACATTTGAGATATTTGAGTATTATGGACTCATTTATTTTAAATCTTCCAGATTCCATTACAGAATTGTATAATTTAGAAACACTGATCCTTCGGAATTCCAGTTTTAAAATGTTGCCCGATAATATCGGCAATTTGATCAACCTCAAGCATTTAGATCTTTCTAATAATCGAAATTTAAAATTCCTGCCAGATTCTATTAGTGACTTGTGTAAATTGGAAGAACTCATCCTTCATGGTTGTTTGAGATTAGAAGAATTCCCAGAAGATACAAAAAAGTTGATCAACCTTAAGCATCTTAGCATATGCGGATGTTTGAGTCTCACCTATTTACCAAAAAGATTGGGTGAGCTGAGTGATCTTCAGATATTGAGATTTCAGATTAACAGAATTGATTAA

>Cucsa.163670

ATGGCAGAATCAATTCTGTTCAGCCTTGCAGCAAATATTGCAACCAAATTGGGTTCTTTAGCACTCCAAGACCTTGGATTGCTGTGGACCGGTATCCATGAGGAGATTGACAAACTCAGAGACACTCTTTCCGCCATCCAAGCAGTACTTCACGACGCAGAACAGAAGCAGTACAAGAGTTCTGCTGTGAAGGAATGGGTTTCAAGGCTAAAAGATGCTTTCTATGATATGGATGATTTGATGGATGAGTTCTCCTATGAATCCTTTCAAAGACAGGTTATGACCAAACATAGAACCAACAACTGTACCAAACAAGTATGTATTTTCTTCTCAAAATCTAATCAAATTAGATTTCGTTTGAAAATGGTTCATAAAATAAAAAAGATCAGGGAGAAACTCGATACTATTGATAAGGATAAAACTCAATTCAATCTTTTTGATAATACAAGGGAGATACGAAATGATGAAATGACAAAACGATCAGAGACTTGCTCTTTTATACTTGAAGGAGAAGTAATTGGTCGAGATGATGACAAGAAATGTATTGTACATTTTCTATTGGATACCAACATTATTGCAAAGGAAAATATTGTTGTGGTTGCCATTATTGGAATGGGAGGATTAGGAAAGACTGCCCTTGCTCAATCTATCTACGGCGATATGAAGGAAAATAAACATTTTGAATTGACAATGTGGGTGTGTATTTCTGAAGAATTTGATGTCAAAGTAATTGTTGAAAAGATCATAGAATCTCTCACAAAAAAGAGACCTAAGCCCAACCTTACACTCGATACCTTACAAAGTATGCTACGAGAGAAAATTGATGGAAAAAAATACTTGCTTGTCATGGATGATGTGTGGAACGATGAACGGACGAAATGGATTAATCTAAAAAAATTTCTTATGGGTGGAGCTAAGGGAAGTAGGATTTTGATCACAACTCGTACCCATCAAGTTGCACATATTTTTGACACAGATTTGTTCCATGATTTAAGTGAACTAGACAAGGACAACTCTTGGGAGTTGTTTAGAAAAATGGCATTTTCCAACGAATCAGAGATGCTTGAGAATTCAAAGTTGGTCGGGATCGGTAAGGAGATTGTGACAAAGTTGAAAGGTTCTCCTCTTGCAATAAGAGTAATTGGAAGCTATCTGTATTCTAAAAAGTCAGAAAAGGATTGGTTGTCATTCAAGGAGAACGAACTTGACACAATCATGCAACAGGAAAATGAGATTCAATCCATACTAAAGATTAGTTTTAACCACCTCTCATCCAGTTTGAAGCAATGTATCACATATTGTGCTTTGTTCCCTAAAGATTTTGAGATTGATAAAGATGATTTGATAAAACAATGGATGGGAGAAGGCTTCATTCAACCACATAATAAGAAGGCAATGGAAGATGTTGGTGATGAATATTTCAAAGAACTCTTGGGAAGATCATTTTTTCAAGACATAAGTAAAAACCAACTGGGAGAGATCATGAAGTTCAAGATGCACGACTTCATGCATGATCTTGCATGTTTTGTTGGAGAAAATGATTATGTGTTTGCTACTGATGACACTAAGTTCATTGACAAAAGGACTCGACATTTGTCAATTTCGCCCTTCATCTCAAAGACAAGATGGGAAGTCATTAAAGAATCATTAATAGCGGCAAAGAATTTGAGAACATTGAACTATGCTTGTCACAATTATGATGGTGATGAAATCGAAATCGACTTCTCTAATCATTTGCGGTTACGAACATTGAATTTAATATTTTCTACTCATGTTCCCAAGTGTATTGGTAAGATGAAACATTTGAGATATATTAATTTTACTCGGTGTTATTTTGATTTCCTTCCCAAGGTAGTTACAAAATTGTACCATTTGGAAACACTTATCTTTCGTGAATGTTTCAAGCTAAGAGAACTGCCAAGTGATATTACGAATTTGATCAATCTCAGGCATCTTGGTATTAACTCTTTAATTGAAGGTTTAAGTTATATGCCAAAAGGAATGGGTTCAATGACTACCCTTCAAACAATGAATTTGTTTATATTGGGAGAGAATGAAGGTGGTGAGTTAAGTGAACTTAATGGATTGATTAACTTGAGAGGATCATTAAGTATTCAACAATTGCAGTTCTGCAAACCCATTGGTATAGAAAATGCTAAACACCTTGAAGAAAAGTCTGGAATTCAAAAGTTAAAATTATATTGGTATCTCTTGGAAAGGAAATATGAAATTGATGATGAAGATGAGAAAGTTTTAGAGTGCTTGAAACCACATCCAAATCTTCAGAAAATAGTCATAAATGGATACGGTGGAGTGAAGCTATGTAATTGGTTCTCATTTGATTATATTGTCAATTTGGTCATTATAGACCTTTTCAACTGTAATAAATTGCAACAGCTCCCTCGATTTGATCAATTTCCTTTTCTCAAACATCTTAAGCTCCAATATTTACCAAATGTTGAGTTTATTGATAATAACGATTCTGTTTCTTCTTCGTTAACAACTTTCTTTCCCTCCCTTGAGAAACTGAGAATCTTTAGGTTACCTAAGTTGAAAGAATGGTGGAAGAGGAAACTCATCGATCAAACTATTCCACAACATAGACGTTTGGAATCATTGAACATAAGTGGTGTTAGTTTGCAAGTTTTTGAGTTGGTAATGGAAATGGCTACTACAAACATTATTGTTGGATCACAGGATTCTTCTTCTTCAACTACATCTATATCATTATCTTTTCTAAGTATTGAAGACATTGATTTTGAGTTTTTACAATTCCATGACTTATTCTCCAATATGACACATCTTAAGTCTCTTTGGATAATAAATTGCAAGAATATAAAAATGTCTTCTTCTCTTGATGCTGTGACATGGAAAGGACTTGGAAGTCTTCGTGAACTTATGTTGTCCAGCATCCCTGATTTGGAATATTTGCCGAAGAGTTTGCAATGTGTGACAACTCTTCAAAGTTTGCAAATATATAATTGTCCAAATTTGGTATCTATTGAAAGTATTAGGCATCTCACCACTTCACTATCAGTATTGGAAATTCATGGTTGTCCTAATATAACTTTCTACCCTCACGAAATGAGTCAACTCGCTTCACTAGCTATCACATTTCAGAATCGTGGTTGGTCGGATGTAAGAGGCAGATTTTCCACCACTGAAGACATTCTTATTTGCCTAAGGTATGCATTACATTTATTTTCTAACAATAATTATAATTTTTTGCATATCTTTGAAAAAACATATTATTTTCATATACCCTCCAACTTTACGTGTGTTTTGGCAAAGGTTTTGGTACTAATAAATAAAAAGTATTCTTCAACAACATTGGATAAGAATTATTTAAACAATATTTTCACAAAAGGATGA

>Cucsa.178620

ATGGCTGAAGCTATTCTCTTCAACCTTACTGCAGACATCATATTCAAACTGGGTTCTTCAGCACTCCGACAGTTTGGATCTCTACGGGGCGGTGTCAAGGATGATTTTGACAAACTCTGGCACTCTCTTTCTGCCATCCAAGCTGTTCTTCACGACGCGGAGGAGAAGCAGTTCAAGGACCATGCGGTCGAAGTTTGGGTTTCAAGGCTTAAGGATGTTTTGTACGAGATTGATGACTTGATCGACGAGTTCTCTTACCAAATCTTGAGAAGGCAAGTTCTGCGAAGTAACAGAAAACAAGTACGTACCCTCTTCTCCAAATTTATAACTAATTGGAAAATAGGCCACAAAATCAAGGAAATCAGTCAGAGGCTACAAAATATTAATGAAGATAAAATTCAATTTAGCTTTTGTAAGCATGTGATAGAGAGAAGAGATGATGATGATGAAGGGTTGAGAAAGAGACGGGAGACTCACTCTTTTATACTTGAAGATGAAGTGATTGGTAGGAATGATGACAAGGAAGCAGTCATAGATCTTCTGCTAAATTCCAACACCAAAGAGGATATTGCAATTGTTTCCATTGTTGGAATGCCAGGATTTGGAAAAACTGCCCTTGCCCAATCTATTTATAACCATAAGAGGATAATGACTCAATTTCAGTTGAAAATATGGGTGTGTGTTTCTGACGAATTTGATCTGAAAATTACTATCCAAAAGATAATAGAGTCTGCAACCGGGAAGAAGCCTAAATCATTCCTTCAAATGGATCCATTACAATGTGAGCTTAGAAAGCAAATTGATGGAAAGAAATATTTGATCGTCATGGATGATGTGTGGAATGAGAAAAAAGAGAAATGGTTACATCTGAAAAGATTGTTGATGGGCGGTGCAAAGGGTAGTAGGATTTTGATCACAACACGCAGTGAACAAGTTGCAAAAACTTTTGACTCTACTTTCGTTCATCTATTACAAATTTTGGATGCATCCAATTCTTGGTTATTGTTTCAAAAGATGATTGGTTTAGAAGAACATTCAGATAATCAAGAGGTCGAGCTTGATCAAAAGAATTCAAATTTGATCCAAATCGGCATGGAGATTGTGTCAACGTTAAGAGGTGTTCCGCTTTTAATAAGAACCATTGGAGGACTTTTAAAAGATAATAAATCAGAAAGATTTTGGTTGTCTTTTAAGGATAAGGAACTTTATCAAGTCTTGGGACGAGGACAAGATGCTCTGAAAGAAATACAATTGTTTCTTGAGCTTAGTTATAAATATCTCCCATCGTCTAACTTGAAACAATGTTTCCTATATTGTGCTTTGTTCCCCAAAGATTATCGAATTAAAAAGGATGAACTTATATTACTATGGAGAGCACAAGGTTTCATTCAACAAAATGGCAACAACGACGACAATAGTTCCCTCGTTGATATTGGTGAAGATTATTTCATGGAGTTATTATCAAGGTCGTTTTTTCAAGAGGTTGAAAAAAATGATTTTGGAGATATAATAACATGTAAGATGCATGATTTGATGCACGATCTTGCTTGTTCGATAACAAATAATGAATGTGTGCGTGGACTGAAGGGAAATGTCATCGACAAAAGAACTCATCACCTTTCTTTTGAAAAAGTTAGTCATGAAGATCAACTTATGGGATCATTATCTAAGGCAACTCATTTGAGGACACTTTTTAGTCAAGATGTTCATTCACGATGTAACTTGGAAGAAACCTTCCACAATATTTTCCAATTGCGAACATTGCACTTAAACTCGTATGGTCCACCCAAATGTGCAAAGACTTTGGAGTTTATTAGTAAGTTGAAACATTTGAGATATTTGCATCTTAGAAATTCGTTTCGTGTTACATATCTTCCAGATTTAAAGTTGTATAATTTAGAAACATTTATCTTTCAAAGCTCTTTGTTAAAAAAGTTGCCTAGTAATGTAGGAAACTTGATCAACCTTAAGCATTTGGATCTTTCTTCTCATTTAAATTTAGAATTCCTTCCCGATTCTATTACAAAATTGTATAAGTTGGAAGCACTTATACTTGACGGTTGTTCCAATTTGAAAGAATTGCCAAAATATACTAAAAGGTTGATCAACCTTAAGCGTCTTGTTTTGTACGGATGTTCGGCTCTCACTCATATGCCAAAAGGATTAAGTGAGATGACTAATCTTCAAACATTGACTACATTTGTATTGGGAAAGAATATTGGTGGTGAGTTAAAGGAGTTGGAAGGACTTACTAAATTAAGGGGAGGATTAAGCATTAAACATTTGGAATCTTGTACCAGCATTGTTGATCAACAAATGAAGAGTAAGAACAGTAAGTTCTTGCAACTAAAGTCTGGTCTTCAAAACTTGGAGTTACAATGGAAGAAACTGAAAATTGGTGATGATCAGTTGGAGGATGTGATGTACGAAAGTGTTTTAGATTGCTTACAACCACATTCAAATCTTAAAGAGATACGTATTGATGGATATGGTGGAGTAAATTTATGTAATTGGGTATCCTCTAATAAGTCCCTTGGTTGTCTTGTCACTACATATCTTTATCGTTGTAAAAGATTACGACATCTCTTCAGATTAGATCAATTTCCTAATCTCAAGTATCTTACGCTTCAAAACTTACCCAACATCGAGTACATGATTGTAGACAATGATGATTCAGTTTCTTCATCAACAATTTTTCCATACCTAAAGAAATTTACTATTTCAAAAATGCCTAAGTTGGTGAGCTGGTGCAAGGATTCAACCTCAACCAAATCTCCTACAGTAATATTTCCTCACCTTTCTAGTTTAATGATTCGCGGTCCTTGCCGACTACATATGTTGAAGTATTGGCATGCACCTAAGTTGAAACTCTTGCAAATTAGTGATTCAGAGGATGAGTTGAATGTTGTACCGTTGAAAATTTATGAAAACCTCACCTTTCTATTTCTTCACAATTTGAGTAGAGTGGAGTACTTGCCCGAGTGTTGGCAACATTATATGACATCTCTACAACTTCTTTGTTTAAGCAAATGCAACAATTTAAAGAGCTTGCCGGGTTGGATTCGCAACCTTACCTCTCTTACAAATTTGAATATTTCTTATTGCGAAAAGCTAGCTTTTCTACCAGAAGGGATTCAACACGTCCATAATTTACAATCGATAGCAGTTGTTGATTGTCCTATATTGAAAGAATGGTGCAAGAAAAACAGACGAGAAGATTGGCCTAAGATCAAGTACTATATTTCCGAACATAGTCATTTAATCAAGAATTGA

>Cucsa.328080

ATGGCAGAATCCATTCTGTGCAGCCTTGCAGGAAGCATTATTACCAAATTGGGTTCTTTCGCACTTCAAGACCTTGGATTGTTGTGGGGTTTCCATGATGAACTTGACAAACTGAAAGGCACTGTTTCCGCCCTCGAAGCCGTACTTCTCGACGCAGAAGAGAAGCAGTCCAAAAGTCGTGCAGTGAAGGACTGGATTTTAAAGCTTAAAGATACTTTCTACGACATCGACGATTTGTTGGACGTGTTCTCCTATGAATCTTTGAAAAGACAAGTTATGACCAAACATAGAACTAACAACACCAAAAAAGTACGCATTTTCTTCTCAAAATCTAATCAAATTGCATTTCGTTTGAAAATGAGTCAAAAAATCAAAAGGGTCCGAGAGAAATTAGATGCAATTGCTATGGATAAAACTCAATTCAATCTTTATGAGAATACTAGGGAAATACAAGATGATGAATCGACGAAACGACTGGAGACTACCTCTTTCATACGTGAAGGAGAAATAATTGGTCGGGATGATGACAAAAAAAGTATTATACATTATCTATTGGATACCAACATCCACGAAGATAGTGTTGCAGTGATTGCGATTATTGGAATGGGAGGATTAGGAAAGACTGCTCTTGTTCAATCTATTTATGGTGACGAGAAGGTAAAGAAACATTTTGAGTTGACAATGTGGGTGTGTATTTCTGAAGAATTTGATGTCAAAGTAATTATTGAAAAAATTATAGAGTCTCTCACAAAAAAGAAACGTGAGCCTGACCTTCAGCTTGATACGTTGCAAAGTATGGTCCGAGAGAAAATTGATGGAAAAAGATACTTGCTTGTCATGGATGATGTGTGGAATGTTAATCGTGCAAAATGGATAAGTCTAAAAAGGTATCTCATGGGTGGAGCTAAGGGAAGTAGGATTTTGATCACAACCCGTACTCATCAAGTTGCACAGACTTTTGAAACAATTTTATCCCATCATTTAAAAGAACTAGATGAAGAAAAATCTTGGAAATTGTTTAGAAAAATGGCATTTTCCAACGAATCAGAGGTGCTTGAGAATTCAAAGTTGGTTGTAATTGGTAAGGAGATTGTTACAAAGTTGAAAGGTTCTCCTCTTGCAATAAGAGTAATTGGGAGTTATCTCTATTCAAAAAAGTCAGAAAAGGATTGGTTGTCATTCAAGGACCATGAACTCGACACAATCATGCAACAAGAAAATGAGATACAATCGATACTAAAGATCAGTTTTAACCACCTCTCATCTAGTTTGAAGCATTGTTTCACATATTGTGCATTATTCTCCAAAGATTATCACTATGAAATTCGAAAAAATGATTTGATAAAACAATGGATGGCACAAGGCTTCATTCAACCACATAATAAGAAGGCAATGGAAGATGTTGGTGATGATTATTTCGAAGAACTACTGGGGAGATCATTTTTTCAAGACATAAGAAAAAACAAATGGGGAGAGATCAAGAAGTTCAAGATGCACGACATCATACACGATCTTGCATGTTCTGTTGTAGAAAATGACTGTGTACTTGCTAATGATGACACTAAGTCCATTGACAAAAGGACTCGACATGTGTCAATTTCGGCCTTCAACTCAATGACAAGATGGAAACTCATTACAAAATCATTAATAGAGGCAAAGAATTTGAGAACATTGAATTATGCTCGTCGTCATCACATCGATCTCTCTAATCATTTGCGGTTACGAACATTGAATTTGGAGTTTCATTTTGTTCCCAAGTGTATTGGTAAGATGAAACATTTGAGATATATTAATATTACTTACTGTTATATTGATTTCCTTCCCAAGGCAGTTACAAAACTGTACCATTTGGAAACACTCATCATTCGTGGATGTCTCGAGCTAAGAGAATTGTCAAGTGATATTAAGAATCTTATCAATCTTAGACATCTAGATATTAAGGATTTTAAACATGTTTGGAGTTATATGCCAAAAGGAATGGGTTCAATGACTACCCTTCAAACAATGAATTTGTTTATATTGGGAGAGAATAAAGGTGGTGAGTTAAGTGAACTCAATGGATTGGTCAACTTGAGAGGATCATTAAGTATTCAACAATTGCAGTTCTGCAAACCCATTGGCTTAGAAAATGTTAAATACCTTGAAGAAAAATCCAGAATTCAAAAGTTGGAATTACATTGGAAGACCTATCAAAGGGAATCTAAAATTGATGATGAAGATGAGAGAGTTTTGGAGAGCTTGAAACCACATTCAAATCTTCAAAAAATACGCATAGAAGGATATAGAGGATTGAAGTTATGTAACTGGTTCTCATTTGATTCTATTGTGAATTTGGTCTTTATAAAGCTTTTCAACTGTGAAAAATTGCAACAGCTTCCTCGATTTGATCGATTTCCTTTTCTCAAACATCTTCATCTGGAAGATTTACCGAGTATCGAGTATATTGCTATTAACAATTATGTTTCTTCATCAATGACTACTTTCTTTCCATCCCTTGAGAATCTAAGCATCATTAAGTTGCCTAACTTGAAAGAATGGTGGAAGGGGGAAAGCATTGATCAAAATACTTCATTTCCAACAATTTTACGTCATCTTTCTCAACTAAAGATTCATTATTGTAGACAATTGGCTTCTATTCCACAACATGGACCTTTGCAATCATTGGACATACGTGATATCAGTTTGCAACTTTTTGAGTTGGTAATCAAAATGACCGCTACGAACATTATTGTTGGGGAGGATTCTTCTTCTTCAGCGAATGATATGTTTATTAGATCCTCATCTTCTCTAAAGATCTGGAAGATTGATTGGGAGTTTCTACCAAATGACTTATTCTCCAATGTGACACATCTCCAGTCTCTTGTCATAGGACGTTGCTTCAATTTAAAAATGTCTTTTGATGATGATAATGTAAGATGGAAAGAACTCGGAAGTCTTCGCACACTTCGACTTTGTTTCATCCCCAAATTAGAGTATTTGCCTAAGGGTTTCCAATATTTGAAAGCACTCGAACATTTGGAACTACTTTGGTGTGAAAATTTGGCATGTATTTTAGGGATTGAGCATCTCACTTCACTATCACGATTGGAAATTTCAAATTGTCCTAATTTAACTTCTTTGCCGGAAGGGATGACTCAACTTATTTCATTAACATGTTTGATAATCGATGATTGTCCCAATTTAAGTACCTTGCCAGAAGGGCTTCATCACCTCCTTAATACCCCGAGGTACGCACCACTAATTTTTTCCCACTAA

>Cucsa.017460

ATGGCTGAGTTCTTATGGACTTTTGCTGTGCAAGAAATTTTGAAGAAGGTCCTAACTCTTGTGGCTGAGCAGATCATTCTAGCAAGGGAGGTCAAGGATGTGCTACAACAACTACAAAAAGAGCTAGTTGAGTCTCAAAAAATTGTTAGTGCTATCACTACCCAAAGACAAAATCATTATTCACCAGATAGCTTAGTGACTCAGTGGGTGAATGATCTTCAACTTATTGTTCATGAGGCTGACGACTTGCTGGATTTGTTTGTTTATGAACATCTTCAGCAAAGAGTGAACCCATCAGCTCATGGAAAAATAATAAAAAAGGTACTCTTTATTTCTTACTTCATTCATATATGTTTAGTGTGTAACGACTTATAAATTAGTATATTGAATAAAAATCAACCCATCGAGAGGTTATGGTCTGTGATCTCTTTATCTTGCCTGCTATATTCTTCAAACCCTGAGTTATTGCATATAATTGGGCCCTAGAATAATTTTGAGTTTGGCATATTTATTATTCTTTTGCCAACAGGTACCTCATTTCTTATGCGCTTCGGCAAGGACTAAAAAAATGAAGGAAATTATAGCGTTGTTAAACAAACATTGCACCAAATTACCTCACTTACTTCAACTTGAGCCTACACCCTCAAACATTGCAGAGACTGAAGTTGCGCAAATTCAAGAGACAGTCTCAAAGCCTGAAGATTATGTGGTGGGAAGGAACAGGGAAGTTGAAACCATAGTTGATCGAGTGATTGATGCCAGCAAACAGGAACTCAATTCTATTTTACCCGTTTTTGGAATGGGTGGATTAGGAAAAACCACTTTGGCAAAGTCGGTCTTCAACCATGATAGGATCAAAAATCATTTTGGTATCACTATTTGGATATATGTGTCACAACCTTTTGTCATCAACAACATTTTGCAAGCAATCTTACAAAAGGTGGAGGTTCATTCTAGTGATTGCTCCAACAATAGGGAGGCCTTACTTGAAAAGCTTACAGAAAACATGGGAGAGAAAACATATTTTCTTGTTCTTGACGATGTTTGGAATGAAAACAAAATGTTGTGGGAGAAGTTGAAGGAATGTTTGATGAGTATTACTCATATGTCAGGAAATAGTATTCTTGTCACTACAAGGAGCAGTGGAATTGCAAAAATGATGGAAGAAAATATTGGAAGTCATGAATTAAGAAAATTATCTGATGACCAATGTTGGTCAATATTTAGGAACTTTGCCAATGCCAAGGATGTACCAATGACTTCCAATTTGGAGTTTGTGCAAAAAGAGTTTGATAAAAGAATCGGTGGTCTTCCATTAATCGCTAAAGTTTTGGGAGCAGCAGTTCCATTTTCAGGAGACCATGACCAGTGGGTAGCAAATATAAAAAGCGTTCTAACAACTCCAATAAAAGAGGAAGAGTTTGTTAAATTCACATTGAAGTTAAGCGTTGATCGTCTACCAAATGCTTCAGTAAAGCAGTGTTTTGCTTATTGTTCAAATTTTTCCAAGGGTTGTGAGTTTGACAAAAAGCAAGTGATTCGAATGTGGATGGCACAAGGATTTACTCAACCAGATGAAAGAAACAATGAAACAATGGAAGATACAGGAGAAAGGTACTTTAACATCTTGTTGTCTTTCTGCTTATTTCAAGATGTTGTTAAGAATGAAAGAGGAATAATTGAGAAGGTTCGAATGCATGATCTTATACATGATATTGCTTGTCAAGTTTCAAATGATAAAAAGTTGCGAATAGATCACATCATTTCATCAAATTGGAAAGATTGGACGAAAGATGACAAAATACTTGTGAGCAAGTTACGAACAATAAATTTTTATGATCGTCATCATGTGGTGGTTCAGGATAAGATTGGGGACTTTACTGGTTTGCGTGTTTTGACAATTGAAAATTATATTGTTGAGGAGTTACCAAACTCAATATTCAAGTTGAAGCACTTGAGATATCTAGACATTTCGTATTGTTATTCAATAAAGAAGCTTCCTGAATCTATTGTTCTGCTTTATAATTTGCAAACACTGAGATTTCATCTCTTAAGCAAGGGATTTCTACCAAAAAACGTTGGACAAATGATTAGTTTGAGGCATTTGGAGTTCTCATCTATCGATAAACAAATGTCTCCCTATTTGAGTCAATTGATTCAACTTGAAACATTGCCTAAATTTGCAGTAGGTTTTGAGAAGGGTTGTAAGATTACAGAACTTGGGGTTCTAAGAAACCTGAAAGGGTTGTTGAAGCTTCAACGTTTAGAACATGTTGAAAGTAAAGAAGAAGCCGAAACTGCAAAATTAGTGGAAAAGGAGAATCTAGAAGAAGTACATTTTGTGTGGACAAAGGAAAGGAAGAGAAAAGTAGAGAATAAGAATGATTTGGAAGTGTTGGAAGGACTTCAACCACCCAAAAATGTAGAATATTTGAGAATCAAATACTTTTTAGGTGGGTGTTTACCAAACCAGACGTTTGTTGAGAATTTAGTGAAAATAGAGCTAAGAGATTGTGGAAATTGTGAGAAGCTTCCAAGGCTTGGGCAATTAGGAAATCTAGAGATACTTGATATTTCATGGTTTGAAAGAGTAAAGAGTATAGGGAATGAATTCTATGGAAACAGCTCCAACAACCAAAGGAGTTTATTCCCCAGGTTGAAGGAATTGTATGTTGATGAGATGAGGAGGATAGGAGAATGGGAAGAAGTGGGAAGTAATGTTAAAGCTTTCCCACGTCTTGAACGTTTGTATATTGGTTGTTGTAGAGATTTAGTGAAAATTCCAGATGTTTTTGGGTATTGTGATGAGTATGGTGAGAAGCATCTGGAAGTTGTGGAAATTATTGAACATTTGTG

>Cucsa.094560

ATGGCTGAGTTCCTATGGACTTTTGCTGTTCAAGAAGTGTTGAAGAAGGTATTGAAACTTGCAGCTGACCAAATTGGTTTGGCATGGGGCTTGGACAAGGAGCTTTCAAACCTCTCCCAATGGCTACTCAAAGCAGAAGCTATTTTAGGTGAGATTAACAGGAAAAAACTACACCCTAGTTCTGTGAGACTGTGGGTGGAAGATCTTCAACTTGTTGTTCATGAAGCAGACGATCTATTGGATGAGCTTGTTTATGAAGATCTTCGTACGAAGGTGGAAAAAGGACCGATTAACAAGGTACGTTCTTCTATATCAAGTCTCTCGAATATTTTCATTATCTTTCGCTTCAAAATGGCCAAGAAAATCAAGGCTATTATTCAAAAGTTGCGTAAATGTTACTCTGAGGCCACTCCTTTAGGACTTGTTGGTGAAGAATTCATACAAACAGAGAATGATCTTAGTCAGATTCGAGAGACGATCTCAAAACTTGACGATTTTGAAGTTGTTGGAAGGGAGTTTGAAGTTTCAAGCATAGTGAAACAAGTGGTTGATGCTAGTATTGACAATGTTACATCTATCTTGCCCATTGTGGGTATGGGTGGAATCGGAAAAACAACTTTGGCAAAGACAATCTTCAATCATGAGGAGATCAAAGGACATTTTGATGAAACAATTTGGATATGTGTGTCCGAACCATTTCTTATCAACAAAATTTTGGGAGCAATTTTACAAATGATAAAGGGTGTTTCCAGTGGCTTGGATAATAGAGAGGCTTTACTTCGAGAGCTTCAAAAGGTGATGCGAGGTAAAAGATATTTTCTTGTGCTTGATGATGTTTGGAATGAAAATCTTGCTTTATGGACTGAATTGAAACATTGTTTACTGAGTTTCACTGAAAAATCTGGAAACGCTATTATTGTGACTACAAGAAGTTTCGAAGTAGGAAAGATTATGGAGAGTACTCTTTCTAGCCATCATTTGGGAAAATTATCTGATGAACAATGTTGGTCTTTGTTTAAAAAAAGTGCAAATGCAGATGAACTGCCAAAGAATCTAGAGTTGAAGGATCTTCAAGAAGAATTGGTGACAAGGTTTGGTGGTGCACCATTGGTTGCAAGAGTTTTGGGAGGGGCACTGTGCCTGCGAGTTTTAGTAGTGGATTCATCTATTACAAAACTATCCGAGTCGATTGGTAAGATGAAACATTTGAGATATCTCGACATTTCAAATTCAAAGATAGAGGAACTTCCAAATTCTATCTCTTTGCTTTATAACTTACAAACACTGAAGCTTGGAAGCTCAATGAAAGACCTTCCACAGAATTTGAGCAAGTTGGTTAGTTTAAGACATCTAAAGTTCTCAATGCCACAAACGCCTCCACATTTGGGTCGATTGACTCAACTTCAAACATTGTCTGGTTTTGCAGTTGGATTCGAGAAGGGTTTCAAAATAGGAGAACTTGGATTTTTGAAAAACCTCAAAGGTAGATTAGAACTTTCAAATCTTGATCGAATTAAACATAAAGAGGAAGCCATGAGTTCCAAATTGGTAGAAAAGAACTTGTGTGAGCTATTCTTGGAATGGGATATGCATATTTTAAGAGAAGGTAACAACTACAATGACTTTGAAGTGTTAGAAGGGCTTCAACCACACAAAAATCTTCAATTCCTGAGTATCATAAACTTTGCTGGCCAACTTCTGCCTCCTGCCATTTTTGTTGAAAATTTAGCTGTGATACATCTAAGACATTGTGTAAGATGTGAAATACTTCCAATGCTTGGACAATTACCTAATTTAGAGGAACTAAATATTTCCTACTTACTTTGTCTAAGAAGTATTGGGTATGAATTCTATGGAAATTATTATCATCCCTACAGCCATAAGGTTTTATTTCCCAAGTTGAAGAAATTTGTACTCTCTCAAATGCCCAATCTAGAGCAATGGGAAGAAGTAGTATTCATATCAAAGAAAGATGCAATTTTTCCTCTTCTTGAGGACTTAAATATTAGTTTTTGTCCTATATTAACAAGTATTCCAAATATTTTTAGACGTCCTCTTAAAAAGCTACATGTTTATGGATGTCATGAAGTGACAGGATTGCCAAAAGATCTACAACTTTGCACTTCCATTGAGGATCTAAAGATTGTTGGGTGCCGTAAAATGACACTAAATGTGCAAAATATGGATAGCTTGTCTCGTTTCTCTATGAATGGGTTGCAGAAGTTTCCCCAAGGGCTGGCTAATCTAAAAAACTTGAAAGAAATGACAATCATTGAATGCTCACAAGATTGTGACTTTAGTCCTCTCATGCAACTTTCTTCACTTGTAAAGCTTCATTTGGTTATTTTCCCAGGGAGCGTGACTGAGCAACTTCCTCAACAACTTGAGCATCTCATTGCCTTAAGATCTTTGTACATTAATGATTTTGATGGAATTGAGGTTTTACCAGAATGGTTGGGAAACCTTACCTCTTTGGAAGTTTTGGGACTTTATTATTGTATAAATTTGAAACAGTTTCCTTCAAAGAAAGCCATGCAATGTCTCACCCAATTAGTCCACGTGGATGTCCACAACTGCCCGAGTTCGCAGATTTTGTCCCATGATCTAAAGGCCAAAGCTCATGCCAAAGCAAACTTAGTTCAATGGTAA

>Cucsa.094580

GGTTAGCTGTGTCAATTTCATCCCCGAACACATTAACAAAGGCCACTTATGTTTATATTTTCGAAGTCCAAAGCAATTGTGTTCTTTCCTTGCACCTTCCCCATCACAAACTAAAAGCGTGTGGATATAAATCTTGTCATGTGGAATTTAAATTTTCTTCCCCACAACAAACTGTTGTCCTATTTTGTTTGACCAAGTCATGTGGAATTTAAATCTTCTTCCTCACTTCATGATCATCCATAGTTCTTTGGGAAAGTAAAGATCATAACAGCAAAAAAGTAAAGTTAATAATATGGCTATCGCTGAGTTCCTATGGACTTATGCTGTCCAACAAGTGTTGAAGAAGGTATTGGAACTTGCGGCTGACCAAATTGGTTTGGCATGGGGCTTGGACAGGGAGCTTTCAAACCTCTCCCAATGGCTACTCAAAGCAGAAGCTATTTTAGGTGACGTTAACAGAAAAAAAACTACACCATAGTTCTGTGAGACTGTGGGTGGCAGATCTTCTACTTGTTGTTCATGAAGCCGACAATCTATTGGATGAGCTTGTTTATGAATATCTTCGTACAAAGGTGGAAAAAGGATCGATTAACAAGGTATGTTCTTCGGTGTCAAGTCTTTCTAATATTTTCATTATCTTTCGCTTCAAAATGGCCAAGAAAATCAAGAGTATTATTGAAAAGTTGCGTAAATGTTACTACGAGGCGACTCCTTTAGGACTTGTTGGTGAAGAATTCATAGAAACAGAGAATGATCTTAGTCAGATTCGAGAGACGATCTCAAAACTTGATGATTTTGAAGTTGTTGGAAGGGAGTTTGAAGTTTCAAGCATAGTGAAACAAGTAGTTGATGCTAGTAATCAATATGTTACATCTATCTTACCCATTATGGGTATGGGTGGAATCGGAAAAACAACTTTGGCAAAGACAATCTTCAATCATGAGGAGATCAAAAGACATTTTGATGAAACAATATGGATATGTGTGTCCGAACCATTTCTTATCAACAAGATTTTGGGAGCAATTTTACAAATGATAAAGGGTGTTTCTAGTGGCTTGGATAATAAAGAGGTTCTACTTCAAGAGCTTCAAAAAGTGATGCGAGGTAAAAGATATTTTCTTGTGCTTGATGATGTTTGGAATGAAAATATTGCTTTATGGACTGAATTGAAAAAATGTTTACTGTGTTTTACTGAAAAATCTGGAAACGGTATCATTGTAACTACGAGAAGTATTGAAGTTGGAAAGATTATGGAGAGTACTCTTCCTAGCCATCATTTGGGAAAATTATTTGATGAACAATGTCGGTCTTTGTTTAAAGAAAGTGCAAATGCAGATGAATTGCCAATGGATCCAGAGTTGAAGGATCTTCAAGAAGAATTGGTGACAAGGTTTGGTGGTGTACCATTTGTTGCAAGAGTTTTGGGAGGGGCACCGCGCTTGCGAGTTTTAGTAGTGGATTCATCTATTACAAAACTACCTGAGTCAATTGGTAAGATGAAACATTTGAGATATCTCGACATTTCAAGTTCAAACATAGAAGAACTTCCAAATTCTATCTCTTTGCTTTATAACTTACAAACATTGAAGCTTGGAAGCTCAATGAAACACCTTCCATATAATTTGAGCAAGTTGGTTAGTTTAAGACATTTAAAGTTCTCAATACCACAAACGCCTCCACATTTGAGCCGGTTGACTCAACTACAAACGTTGTCTGGTTTTGCAGTTGGATTTGAGAAGGGTTGCAAAATAGAAGAACTTGGATTTTTGAAAAACTTCAAAGGTAGATTAGAACTTTCAAATCTCAATGGAATTAAACACAAAGAGGAAGCCATGAGTTCCAAATTGGTAGAAAAGAACTTATGTGAGCTATTCTTGGAATGGGATTTGCATATTTTAAGAGAAGGTAGCAACTACAATGACTTGGAAGTGTTAAAAGGGCTTCAACCACACAAAAATCTTCAATTCTTGAGTATCATAAACTATGCTGGCCAAATTTTGCCTCCTGCCATTTTTGTTGAAAATTTAGTTGTGATACATCTAAGACATTGTGTAAGATGCGAAACACTTCCAATGCTTGGAGAATTACCTAATTTGGAGGAACTAAATATTTCCAACTTACATTGTCTAAGATGTATTGGGAATGAATTCTACGGAAGTTATGATCATCCCAACAACCATAAGGTTTTATTTCGCAAGTTGAAGAAATTTGTACTCTCTGAAATGCACAATCTAGAGCAATGGGAAGAATTAGTATTCACATCAAGGAAAGATGCAATTTTTCCTCTTCTTGAAGACTTGAATATTCGTGATTGTCCTATATTAACAAGTATTCCAAATATTTTTGGATGTCCTCTTAAAAAGCTACATGTTTGTGGATGTGATGAAGTGACAAGATTGCCCAAAGATCGATCTACAACTCTGCACTTCCATTGAGGATCTAAAGATTGTTGGGTGCCTTGAAATGATACTAAATGTGCAAAATATGCATACCTTGTCTCGTTTCTCTATGAATGGGTTGCAAAAGTTTCCCCAAGGACTATCTCATCTCAAAAACTTGAAAGAAATGATAATCACTGAATGCTCACAAGATTGTGACTTTACTCCTCTTATGCAACTTTCTTCACTCGTAAATCTTGATTTGGTTCTTTTCGCGGGGAACGGGGCCGTGCAACTTCCTCAGCAACTCCAGCATCTCACAGCCTTAAGATCTTTGATCATTAATGATTTTGATGGAATTGAAGTTTTACCAGAATGGTTGGGAAATCTTGCATCTTTGGAAGTTTTGGGACTTTATTATTGTAGAAGTCTGAAACAGTTTCCTTCAAAGAAAGCCATTGCAATGTCTCACCCAATTAGTCCATGTGGATGTCTTTGGTTGTCCACAACTACCCAAGTTCGGAGATTTTGTGCCATGATGTTCTAAAGACCAAAGCTCGTGCCAAAGCAAACGTAATTTAGTGGTAAAGTTAATATATTGTAACTTGTAGGTAGCTGCCTTAAATCTCTCGCTAATATTATTCGCATTTGTTTTTCTTGTATTGAACAATGCATGTTGTTTTTTCCTTTTTTGTTAGTAGAGTGTGAGAAGACTAACTTTTTTTCTCTTTGTGATTGATTTGTCTATAACATAATGTATACTCAAAACTCTATATACGAACAGCATGTTTGAGGTGCAGGTGAAATTAAATGAAAAAGAAAAACTAGCAATTTCTTAC

>Cucsa.094650

ATGGCTGAATTCCTTTGGACATTTGCTGTGGAAGAGACGTTGAAGAGAACGGTGAACGTTGCAGCTCAGAAAATTTCTCTCGTTTGGGGTTTGGAAGATGAACTTTCAAATTTAAGCAAATGGCTACTCGATGCTGGAGCCCTTTTGCGCGATATCGATAGGGAAATACTTCGCAAGGAATCGGTGAAGAGATGGGCAGATGGGCTTGAAGATATCGTTAGTGAAGCTGAGGATCTTTTGGACGAGCTTGCTTATGAAGATCTTCGAAGAAAAGTGGAAACAAGTTCAAGGGTGTGTAATAATTTCAAATTTTCTTCTGTTCTTAACCCTCTTGTTCGTCATGATATGGCCTGTAAAATGAAGAAAATTACTAAAATGTTAAAACAACATTATCGCAACTCTGCTCCTTTAGGGCTTGTTGGGAAGGAATCCATGGAGAAAGAAGATGGAGGTAATAATCTTAGGCAGATTAGGGAAACAACTTCGATTCTGAATTTTGATGTTGTGGGAAGGGAAACTGAAGTTTTAGACATATTGAGATTGGTGATTGATTCTAGTAGTAATGAGTATGAGCTTCCTTTGTTGATTGTACCGATTGTAGGGATGGGTGGAGTTGGAAAAACAACTTTGGCGAAATTGGTTTTTCGTCATGAGTTGATCAAGAAACATTTTCATGAAACAATATGGATATGTGTGTCGGAACACTTCAACATCGACGAGATTTTGGTAGCAATTTTGGAAAGTTTGACGGATAAAGTTCCAACCAAAAGGGAAGCTGTACTTCGCAGGCTTCAAAAAGAGTTGCTAGACAAAAGATGTTTCCTTGTTTTGGATGATGTTTGGAATGAAAGTTCTAAGTTGTGGGAAGAGTTAGAAGACTGTTTAAAAGAGATAGTTGGGAAATTTGGAATCACCATTATAGTAACTACAAGGTTGGATGAAGTTGCTAATATTATGGGAACAGTTTCGGGTTATCGTTTGGAAAAGTTACCTGAAGACCATTGTTGGTCCTTATTTAAGAGAAGTGCAAATGCAAATGGAGTAAAAATGACTCCAAAGTTGGAGGCTATTCGAATAAAGTTGCTTCAAAAAATTGATGGCATACCGCTTGTTGCAAAAGTTTTGGGAGGAGCCGTGGAATTTGAAGGAGATCTTGATAGGTGGGAGACCACACTTGAAAGCATAGTAAGAGAAATTCCAATGAAACAAAAAAGTTATGTGTTGTCCATATTACAATTAAGTGTGGACCGTCTACCCTTTGTGGAAAAACAATGTTTTGCCTATTGTTCAATTTTTCCTAAAGATTGTGAAGTTGTTAAAGAAAATTTGATTAGAATGTGGATAGCACAAGGGTTTATTCAACCAACAGAAGGAGAGAACACGATGGAGGATCTGGGAGAAGGGCACTTCAACTTCCTCTTATCTCGCTCCTTATTTCAAGATGTCGTCAAGGATAAGTATGGGAGAATTACTCACTTTAAGATGCATGATCTAATACATGATGTTGCCCTTGCCATTTTGTCAACTCGTCAAAAGTCGGTATTAGATCCTACTCATTGGAATGGAAAAACGTCAAGAAAGTTGCGCACCTTACTTTACAATAACCAAGAGATCCACCATAAAGTTGCAGACTGTGTTTTCTTGCGTGTTTTAGAAGTGAATTCCTTACATATGATGAATAACTTACCAGACTTCATTGCTAAGTTGAAACACTTGAGATACCTTGACATTTCATCATGTTCTATGTGGGTTATGCCCCACTCTGTTACTACGCTTTTCAATTTACAGACACTGAAGCTTGGAAGTATAGAAAATCTTCCAATGAATTTGAGAAATTTGGTTAGACTACGTCACTTAGAATTCCACGTCTATTACAACACAAGGAAAATGCCTTCTCATATGGGTGAGTTGATTCATCTTCAAATATTGTCTTGGTTTGTTGCAGGGTTTGAGGAAGGCTGTAAAATTGAAGAACTCGGAAATTTGAAAAATTTGAAAGGTCAATTGCAACTTTCAAATCTTGAGCAAGTGAGGAGTAAAGAAGAAGCTCTAGCTGCAAAATTGGTCAATAAGAAAAACTTACGTGAGCTAACTTTTGAATGGAGTATAGATATTTTACGAGAATGTAGCAGCTACAATGACTTTGAAGTGTTGGAAGGACTTCAACCACCCAAAAATCTCAGTTCTTTGAAAATTACCAACTTTGGAGGGAAATTTTTGCCTGCTGCTACTTTTGTTGAAAATTTGGTGTTCCTATGTTTGTATGGTTGTACAAAATGTGAAAGGCTTCCAATGCTTGGACAATTAGCCAACTTGCAAGAACTTAGTATTTGTTTCATGGATAGTGTGAGAAGTATAGGGAGTGAGTTTTATGGCATTGACTCCAACCGAAGGGGTTATTTTCCCAAGTTGAAGAAATTTGACTTCTGTTGGATGTGCAACCTAGAGCAATGGGAATTAGAAGTGGCAAATCATGAGTCAAATCATTTTGGTTCTCTTCAAACTCTAAAGTTGGATAGATGTGGCAAATTGACAAAACTGCCAAATGGGTTAGAATGTTGCAAATCTGTTCATGAGGTGATAATATCAAATTGTCCTAACCTTACCTTAAATGTAGAGGAAATGCATAACCTGTCTGTTTTATTAATAGATGGGTTGAAGTTTTTGCCAAAAGGATTAGCTCTCCACCCTAACTTGAAGACCATAATGATTAAAGGATGCATAGAGGATTATGATTATAGCCCTTTCCTAAACTTGCCTTCTCTTACAAAACTTTACTTGAACGATGGCCTTGGAAATGCCACCCAGCTTCCTAAACAACTTCAGCATCTCACTGCCTTAAAGATTTTAGCCATTGAAAATTTTTATGGCATTGAAGTTCTTCCTGAATGGTTGAGAAAGCTTACATGTTTGGAGACTTTGGATCTTGTTCGTTGCAAAAACTTGAAACGGTTGCCTTCAAGAGGAGCCATGCGATGCCTCACCAAATTAAAGGATTTCAAAGTTATAGCATGTCCATTGTTGCTACTTGGGGGCCAAGCTGACCAAGAAGGTGCCAAGTATCTTCATATTCCAGCCTATCTTTGTCATGTGTATCAATCTAGAGGAAGCCCTCTTTCCAAAACATCTTCCATCTAA

>Cucsa.094660

ATGGCGGATTTCCTATGGAGCTTTGCTGTAGATGAAGTGTTAAAGAAGACAGTGAAGCTTGTGGCAGAGCAAATTGGCATGTCATGGGGGTTTAAGAAGGATCTTTCAAAACTAAGGGACTCTTTACTAATGGTAGAAGCCATCCTACGTGATGTTAACAGAATCAAGGCAGAACATCAAGCCTTGAGGCTATGGGTGGAGAAGCTTGAACATATCGTTTTTGAAGCCGACGTTTTACTCGACGAGCTCTCTTACGAAGATCTTCGACGCAAGGTGGACGCCAGGCCGGTACGTAGTTTCGTTTCATCCTCAAAAAATCCCCTTGTTTTTCGCCTCAAAATGGCCAATAAAATTAAAGCTATTGCTAAAAGGTTAGACGAGCATTATTGTGCAGCGAGTATCATGGGGCTTGTTGCTATAACATCCAAAGAAGTCGAGTCCGAACCTAGCCAAATTCTAGAGACAGACTCGTTTCTTGATGAGATTGGAGTTATAGGGAGGGAAGCTGAAGTATTAGAGATAGTGAATAAACTACTTGAACTTAGCAAACAAGAAGCAGCTCTATCTGTTTTACCAATTGTTGGTATAGGTGGACTAGGAAAAACATCTTTGGCGAAGGCGATATTTCATCATGAAATGATAAGGGAGAATTTCGATAGAATGATATGGGTGTGTGTGTCTGAACCTTTTGTTATCAACAAGATTTTAAGAGCAATTTTGGAAACTCTTAATGCTAATTTTGGTGGATTAGACAATAAGGAAGCTTTACTTCAAGAGCTTCAAAAATTGTTGAGGAACAAAAAGTATTTTCTGGTGCTTGACGATGTCTGGAATGAAAATCCTGATCTGTGGAATGAGTTAAGGGCTTGTTTGCTAAAGGCCAATAAAAAATTTGGAAGTGTTATTGTTGTGACTACTAGGAGTGATGAAGTTGCAAATATTGTGGAGACAAATCATCAAAGACATCGTTTGAGAAAGTTATCAAATGATTATTGTTGGACTTTATTTGAAAAATGTGCATTTGGAAGTGATTTGCCAGTGACTCCAAGAGTTGATCATGTAATCAGAGAAGAGCTTGTTAAAAGATTTGGTGGCATACCTTTGGTTGTGAAAGTGTTTGGAGGAATGGTGAAATTAGACAAGAATAAATGTTGTCAAGGATTGCGATCAACTTTGGAAAATCTAATCATAAGTCCATTACAATATGAAAATAGTATTTTATCTACCATAAAATTAAGTGTGGACAGGCTGCCATCATCTTCATTGAAGCAATGTTTTGCCTATTGTTCAAACTTTCCACGAGGCTTCTTATTTATAAGAGAACCACTTGTTCAAATGTGGATAGCACAAGGGTTTATTCATCTACCTAGTGGGAGCAATGTAACGATGGAGGATATTGGAGCAAACTACTTTAATACTTTGTTGTCTCGCTCTTTGTTTCAAGATGTCGTCAAAGATGACAGAGAAAGAATTCTGTATTGCAAGATGCACGATGTTGTACATGATGTTGCATGTGCTATTTCAAATGCTCAAAAATTGAGACTGAGTGGCAAATCTAATGGAGACAAAGCTCTTTCGATCGGTCATGAAATTAGAACACTTCATTGCAGTGAAAATGTTGTTGAACGGTTTCACCTGCCAACCTTTGATAGTCATGTATTTCACAATGAGATCAGCAACTTCACCTACTTGTGCGTTTTAATTATTCATTCATGGTTTATACATCAACTGCCAGATTCAATTGCTAAGTTGAAGCATTTAAGGTACCTCGACATTTCACACTCTCTAATAAGAACGCTTCCAGACTCTATTGTTTCACTCTATAATCTGCAGACATTGAGGCTTGGAAGTAAAATTATGCATCTTCCTACAAAATTGAGAAAATTGGTCAATTTAAGGCATTTAGAATTCTCTCTCTCAACTCAAACTAAACAAATGCCTCAACATCTGAGTCGATTGCTTCAACTTCAAACGCTTTCGAGTTTTGTAGTCGGTTTCGACAAAGGATGTAAGATAGAGGAACTTGGACCACTGAATAACCTTAAAGGTGAACTAAGCCTTTTCCATCTTGAGCATGTCAAAAGTAAAACCGAGGCTATGGCTGCAAATTTGGCAATGAAGGAAAACATTTCTGATCTATATTTTCAATGGAGTTTGTTAAGTGAAAGAGAAGATTGTAGTAACAATGATTTGAATGTGTTGGAAGGGCTTCGACCACACAAAAACCTTCAAGCCTTGAAAATTGAAAACTTTGGAGGTGTTCTGCCTAATGGCCTCTTTGTTGAAAATTTGGTGGAGGTAATTCTATATGATTGCAAAAGATGTGAAACTTTGCCAATGTTGGGGCACTTATCTAAGCTTGAATTACTTCATATTCGTTGCTTAGATAGTGTAAAAAGTATTGGGGATGAATTTTATGGGAACAATAATAGTTACCACAATGAGTGGTCTTCATTGTTATTCCCTAAACTCAAGACCCTTCATATTTCCCAAATGAAAAGTTTAGAGCTTTGGCAAGAAATAGGGAGTTCATCAAACTATGGTGCGACCTTTCCTCATCTTGAAAGCTTGAGCATTGTTTGGTGTTCGAAATTGATGAATATTCCTAACCTTTTTCAAGTTCCTCCAAAGCTTCAATCTCTCAAGATTTTTTATTGTGAAAAATTGACAAAGTTACCACATTGGTTAAATCTCTGCAGCTCCATTGAAAATATGGTCATATGCAATTGTCCTAACGTTAACAATAATTCTCTTCCAAATTTGAAAAGTATGCCAAACTTGTCGTCCTTGAGCATCCAAGCTTTCGAGAAGTTGCCGGAGGGGCTTGCCACCATTCATAACTTGAAAAGATTGGATGTTTATGGGGAATTGCAAGGTTTGGATTGGAGTCCATTCATGTATCTCAATTCATCGATTGAAATTCTTCGGTTGGTTAACACAGGAGTAAGTAATTTACTTCTGCAACTTCCTCGACAACTTGAGTATCTCACCGCTTTAAGATCATTGGATATCGAACGTTTTAGTGACATTGATTCTTTGCCAGAATGGTTGGGAAACCTTACATCTTTAGAGACGTTAAATCTACGTTATTGCAAAAATTTGAAAAGTTTCCCTTCAATAGAAGCCATGTCTAATCTCACCAAATTAAGTCGTTTGGAAACTTATGAATGTTTCCAACTTAAACTCGACGAAGGTAGCTATGAGCGGGCGAAAATTGCGCATGTACATGATATTAGCTGCTAG

>Cucsa.094670

ATGGCGGAATTTCTTTGGACTTTCGCAGCTCAAGAGCTGTTGAAGAAGACAGTGAAGCTCGCAGCAGAACAGATCGGCCTGGCATGGGGTTTCAACAATGAGCTGTCAAACCTCAGAGACTCTCTACTTATGGTGGAAGCCATTCTTCGTGATGTCGACAGAATTAAGGCAGAGCATCAAGCTGTGAAGCTATGGGTAGAGAAGCTTGAAGCTATTATTTTCGAAGTCGATGTTCTACTGGATGAGCTCGCTTACGAAGATCTTCGCCGCAAGGTTGAACCCCAAAAAGAGATGATGGTAAGTAATTTCATTTCTTTCTCCAAAACCCCTCTTGTTTTTCGTCTCAAAATGGCCAATAAAATCAAGAACATTGCTAAGATGTTGGAAAGACATTATTCTGCTGCTAGTACTGTGGGGCTTGTTGCTATATTATCTAAACAGACTGAACCTGATTTTAGCCAAATTCAGGAGACAGATTCGTTTCTTGATGAGTATGGAGTTATTGGGAGAGAAAGTGAAGTTTTGGAGATTGTGAATGTATCTGTCGATCTTAGCTATAGGGAGAATTTGTCTGTTTTGCCAATTGTTGGCATGGGTGGATTAGGAAAGACAGCTTTGGCTAAGGTAATATTCAATCATGAATTGATAAAGGGGAATTTTGATAGAGCTGTATGGGTGTGTGTTTCGGAACCTTTTCTTATCAAGAAGATTTTAAGAGCAATTTTGGAAACTCTTAATTCTCATTTTGGTGGCTTAGATAGTAAAGAAGCCTTACTTCAAGAGCTACAAAAGTTGTTGAATGATAAAAAGTATTTTCTAGTTCTTGATGATGTTTGGAATGAGAATCCTATCCTCTGGAATGAGTTGAAAGGTTGTTTGTTAAAGATTAGCCAAAGATCTGGAAATGTTGTTGTTGTGACTACTAGGAGTGACAGAGTTGCTGAAATCATGGAGACACATTCTAGATATCATTTGACAAAACTATCCGATGACCATTGCTGGTCTTTATTCAAGAAATATGCATTTGGAAATGAATTGCTACGAATTCCTGAATTGGATATTGTTCAGAAAGAGCTCGTTAAAAGATTTGGAGGCATACCATTGGCTGTAAAAGTGATGGGAGGAATCGTTAAATTTGACGAGAATCACGAGGGATTGCAGAAATCTTTGGAGAATCTAATGAGACTTCAATTGCAAGATGAAAACCATGTTGTATCCACAATAAAGTTAACTGTAGATCGCCTACCATTGCCATCGTTAAAACAATGTTTTGCCTACTGTTCAAATTTTCCAAAAGACTTTAAGTTCAGAAAAGAAGCCCTTATTCAGATGTGGATAGCACAAGGCTTTATTCAACCGTCTTTGGGAAGTGATGAAATGATGGAGGATATTGGTGAGAAGTACTTCAATGTTTTGTTGTCTCGCTTCTTGTTTCAAGATATTGTCAAGGATAATAGAGGGAGAATTATATTCTGTAAGATGCATGATCTTATACATGATGTTGCATGTGCTATTTCAAATTCTCCAGGATTGAAATGGGATCCTTCAGATTTGTTTGATGGAGAACCTTGGAGACGTCAAGCTTGCTTTGCTAGCCTTGAACTAAAAACGCCAGATTGTAATGAAAATCCTTCTAGAAAGTTGCACATGTTGACATTTGATAGTCATGTGTTTCACAATAAGGTCACAAACTTTCTCTACTTGCGGGTTTTAATTACACATTCGTGGTTTATATGTAAATTACCAAATTCAATTGCTAAGCTGAAGCATTTGAGGTATCTTGACATTTCATATTCTACCATAAGGGAGCTACCAGATTCCGCTGTTTTGCTTTATAATTTGCAAACACTGAAGCTTTCAAGATTTTTAAACGGCCTTCCAAAAAATTTGAGGAAGTTGGTTAGTTTAAGACATTTAGAATTTTTCTCTGATCCTTGTAATCCTAAACAAATGCCTCAACATTTGGGTAAATTGATTCAACTTCAAACGTTGTCTAGCTTTGTAGTTGGGTTTGATGATGGATGTAAGATAGAAGAACTCAGATCTTTGAGAAATCTTAAAGGTAAGTTAAGCCTTTTATGTCTTGAGCGAGTGAAAAGTAAAAAGGAAGCCATGGCTGCAAATTTGGTGGAGAAGAGGAATATTTCATATCTGTCTTTTTATTGGGCCTTGAGATGTGAAAGATCAGAGGGAAGCAACTACAATGATCTGAACGTGTTAGAAGGACTTCAACCACATAAAAATCTTCAAGCTTTGAGAATTCAAAACTTTTTAGGCAAACTTCTGCCCAATGTTATTTTTGTCGAAAATTTGGTCGAGATATATCTACACGAATGCGAAATGTGTGAAACTTTACCAACACTTGGGCAGTTATCAAAGCTTGAAGTACTCGAACTTCGTTGTCTATATAGTGTAAGAAGTATTGGAGAAGAATTTTATGGGAATTACCTTGAGAAGATGATTTTATTCCCAACATTGAAAGCATTTCATATCTGTGAAATGATCAATCTAGAGAATTGGGAAGAAATAATGGTTGTATCAAATGGTACAATCTTTTCCAACCTTGAAAGCTTCAACATTGTTTGTTGTCCGAGATTGACGAGCATTCCAAACCTTTTTGCATCTCAGCATGAGAGTTCATTTCCAAGCTTACAACATTCGGCAAAGCTTCGATCTCTAAAGATTTTGGGATGTGAAAGTTTGCAAAAACAACCAAATGGTTTAGAATTCTGCAGCTCCCTTGAAAACATGTGGATAAGCAACTGTTCTAACTTGAACTACCCTCCAAGCTTGCAGAATATGCAGAATTTAACTTCTTTAAGCATAACCGAGTTTCGAAAGCTGCCAGACGGGTTAGCTCAGGTTTGTAAGTTGAAAAGCTTGAGTGTTCATGGTTACTTGCAAGGTTACGATTGGAGTCCTCTTGTACATCTTGGTTCACTCGAAAATCTTGTGTTGGTTGACTTGGATGGAAGTGGTGCAATACAACTTCCTCAACAACTTGAGCAACTCACTTCTTTGAGATCACTGCATATTTCGCATTTTAGTGGCATTGAAGCGCTACCAGAATGGTTCGGAAACTTTACATGTTTGGAAACGTTGAAGCTTTACAATTGTGTAAACTTGAAAGACATGGCGTCGAAGGAAGCTATGTCAAAACTTACAAGATTAACGAGTCTACGAGTTTATGGATGTCCACAACTTAAGCTTAATATAGGAGACTTTGAGCGGGTAAACATTTCCCTTGTACCTACCATCAGTTGA

>Cucsa.132370

TCTAGTCCTTGGATAGATTAGATAGAGAATCTTCCTGCTTCTCTTTCTTAAGAGACTCTCAAACTATTCCCTTGTGCTTGAGAAAGACTCTATAATAGGAGATGCTCTCAATCTTCTTGTTTCTCTAGTGAGCAACTCCTGGTTTTAGATTTAGACCCATCAAACAACCTCTTTCCTTATATAGACAGGCTAATGGATTAATATATTATTCTGAGTGATTTTGTGAAATCTTTTTAAACAAATCTTAGTAAAATTATAAAAAAACGTTATCCAAATTAAAGAGCAGAATATGCAAAACCATGAAAAACAGTAATACTGTTATATGTTATTCCAGTCTTGTTGATATGATCAACATATGTGCTCTATGACTAGCTTCGTCGTAAGCTCGCATTTTCTGCATTTGTTGCAGTTATCTAATTCTTTTGTTCATGAAGGACCCGTGTCTTCTTTCAAAAAAAATGATCGGACGTTGGAATAGCAAATGAGCCAAGGGGCCTCCTCTTGTAGTTGTAGTAACAAAAGACCACAAATTTGAACCTTTGTCTAAGACGAAATGTTGAATGGAATGGCATGATTGTATCCCATTTGTAAGTTCCTTTAGTTGGTCACAGAAACGAAACTCCACAAATTTAAGATGCTTCTCCCCATCATCCTCATCACAATAACCAAAAACATCTGGAATTTTCAATAATTTCTTGCATGAAATAATATGCAAACATTCAAGTCGTGGAAAAGCTTTAACATTATAATTTGACACAGCTTCTTCCCATTCAACTAGGCTGTCCATCTCATCAACATAAAATTCCTTCAACTTGGGGAATACACTACTACTACTACGCCTTTGGTTTCCATAGAACTCATTGCCTATACTCTCTACTTTTCGTAATCGAAAAATAACAAGTATTTCTAAGTACTTGGTTAATTGCCCAAGCATTGGAAGCTTTTCACAGTTTCCACATTCAATTAGAGTTATTTTTACTAAATTTTCAACAAAAATCTGGTTGGGAAACTGCCCACCTGAAAAGGATTCGATTTCCAAATATTTCAGATTATTGTGTTGTTCAAGTCCTTCCAACACTTCAAAATCATTCTCATTCTTCACTATATTCTATGGCTTTCCGTACTCCACACAAAGTGTAGTTCTTCTACCTTCTCTTTTTCCACCAATTTTGCACTCTTGGCTTCCTCTTTACTTTCAACATGTTCTAAACATAGCATCACCAACAAACCTTTCAGCTTTCTTAGACTCCCCAGTTCTGTAATCATACAACCCTTCTTCAATCCTACTACAAAAGCAGACAACGTTTGAAGTTGAGTTAATTCACTCAAATATAAAGGCATTTCTAATGCGGGGGATACATCACTATCTTCAAGTGAAAATTCCAAATGCCTTAAACTAACCATAATTGTCTAAAATTTATTGGTAGAAGACCATTCACTAAGTAAGGTAACCTCAGTGTTTGCAGATTATAAAGCACAACAATAGATTTTGGAAGTTCCCTTATGAAATCACAACTTGAAATGTCTAGATATCTTAGGTGCTTCAACTTGGAAATTCATATGAAAATGTCAAAACACGCAAACGCACAAAGTCTCCAATCTTATCAATATTCTTCTCCATATCTATTACCGTACGTAGCTTGCTCGCAACTATTTTATCATCGTTCGTCCAATGTTTCCTCATTGATAAAAGGCTATGATCCGATGGCAATTTTTCATCATTTGAAACATGACAAGCAATATCATGTATAAGATCATGCATAAGAAACTTCTGACCTATCTCACTTTCATCTTCAACATCTTGAAATAAGCAACAAGACAATAAGATTTTGAGTGAATTTGGAAGCAAATTTAAGTAACTAATAGAATGATCAAAATTAATTAAAGAAGAAAAGACAATTTTTAATGCTGATGTCTACTATATAAATCTTGTACCTGACTCGTGGAAAAGAAGTGCTGTACGGATCTTCTGAGCGTCAGTCCAAATCTCCGGCAGTAATCGCCCAAACTTCTTCACATCTGCAATGAACCCCTTTTCTAGTTCCCCCAAAATCTTCAAATGGGTATGCTTGTAACCCATTTGGAAGTTTCAACTGTTGGTTACAAAGTCTAATATCCACAGAGTTAAGATGTTTCTCGCCATCATTACCATAGTAACCAAAAATAAACATCTGGAATTTTCACTAATTTTGTACATTTAACAATATGCAGACATTCAAGACGAGGAAAAGCTTTAACATTATTTACCACTTCTTCCCATTCAACTAGGCTGTACATCGCAATAACATAAAATTCCTTCAACTTGGGGAATACTACACTACTCTTGCTTTGGCCGTCATTATAATTTCCATAGAATTCATTGCCTATACTCTTTACTTTTGGAAAGTTTGATATAATTATAAGTGCCTCTAGCTTGCTTAATTGCCCAAGCATTGGAAGCTTTTCACAATTTCCACATTTATGTAGAGTTATTGTTACTAAATTCTCAACAAAAGTCTCCTTCGGAAAACACCCACCTAAAAAGGATTGGATTTTCAAATCTTTAAGATTTTTGTGTGGTTCAAGTCCTTCAAACACTTCCAAATCATTCTGATTCTCCACTATTCTTCCACTTTCCACGCCACGACAAGTTTAGTCCTTCTACCTTCTCTTTTTCCACCAATTTTGCAGCCTTGGCTTCCTCTTTACTTTCAACATGTTCTAAACGATGAAGCTTCAACAAACCTTTCAAGTTTCTAAGACCCCTAAGTTCAGAAATCTTACGACCCGTCTCAAATCCTACTGCAAAAGCAAACAACGTTTGAAGTTGAACCAATTCGCTCAAATATGGAGGCATTTCAAATTTAGGGGGTAAATCAATACCCATTAATAGAATTTCCAAATGCCTTAAACTAACCATTCGTCTCAAATTTGTTGGTAGAAGATCATTCGTCAAGCAAAATAGCCCAACAATAGATTCTGGAAGCTTCTTTATTGAATAACAACGTGAAATGTCTAGATATCTAAGATGTTTCAACTTAGAAATTGAGTTTGGTAACTCACGAACATTTTCTGCAATTGTCAAAACACGCAAACACACAAAATCTTTAATCTTACCATTCCTCCCGAAATCTATTACCGTACGCAACTTGCTCTCAATTTTTTTTTTCTCTTCTGTCAAACCTTTCCACTTTGATGAACTGCTATGATCTAATTGCAACCTTTTATCGCTTGAAACATCACAAGCAATATCGTGTATAAGATCATGCATACGAAACTTCCTACCAATATGCCATTTGTCATCATCAGCATCTTGAAATAAGCAATAAGACAATAAGATATTGAAGTACCTCTCTCCTGTATCCTCCATTGTTTCATTATTTCCTTCATCTGGTCGAGTAAACCCGTGTGCCATCCACATTTTGATCACTTGTTTTTTGTCAAACCAATAACCTTTAGGAAAATTTGAACAGTAAGCAAAGCATTGCTTTATTGAAGCTTTTGGTAGACGATCCACGCTTAACTTCAACATGTATTTAACATATGACGACTCTTCATATTTTAATGGAGTTGTTGGAATAGATTTTAAAATTTCCACCAATTTTTTTAACGACCTCTTCTCGCACAAGCTCCAAATTGGAAGTTATTGGTACTGCATTCGCCTTGGCAAAGTAGCTAAATACTGACCAACATTGATCATCAGTTAATTTGTTTAAATGATAGGTGTCAAGTGTTTCTTTCATCATTTCTGCAATTTTACGACTCCTGGTCGTGACAAGGACACTGCTTCCTAATTCTTCAACAATACTCATTAAGCATTCCTTCAACTTCTCCCAGAATATTTTGTTTTCATTCCAAACATTGTCAAGCACAAGAAAATATCTTTTTCCACCCATCACTTCTTTAAGATTGCGAATTAAGGTGTCCTTATCATCCTTGCTACGATTATCATTGCTTTTGCTTACCTTTTTCAAGATTGACTGCAAGATCTCGTTGATGACAAAAGGTTGAGACACACATACCCAAACAGTATGATGAAATTGTTTTCCAATCCTCTCGTCATTAAACACCAACTGGGCCAAAGTGGTTTTTCCTGATCCACCCGTTCCATAAACGGGTAAAATAGAATTGAGTTGTTGTTGACTAGCCTCAGTCACATCTTGAACTATGCTTTCAACTTCCATCTCCCTTCCCACCACATAATTTTCAAGCAGTGAAGTTGTCTCTTCAACCTGCTCAACTTCACTCTCTATTTTTCGGGTTGTATTGTCAAGATGTACTTATTGAAGTAACTTGGTGCAGTGTTTGATAACACAACTATAATTTCCTTCATTTTTTTCATCCTAGTTGAACCAAAGACGACCAAAGAAAAACAATCAAGCATGGGTTTTATGGTGCATAAGGAATTAGGAAATACCTTGTTTGATCTTATTTTTCGTTCAACATCGTCATAAGCAAACAAGTCTAACATATCCTCAGCTTCATAAACAATAAGTTGAAGATCATTCACCCAATGAGTTATTTTGTCATTGAGTGAATGATCATGCTTTGTTTTTATGGCATGGAGGATCCATTCAGCATGAAGTAGATCATCTTTCAGCTCTTCTAGCACCACCTTAAATTCACTTGACGCAATAATTTTTTTGGCTACAAGAGTTACAGCCTGCTTCAAAGTTTCTTGCACACCAAAAGTCAGTAGGTCACCCATTATTTGATCTGATCACCTCTTTAATTTCTCTTTTTCCACACTTGGGATAATGGATACAAAGGATATATTGATTTGATGAAATAATAAACTCCACAACTACCCTATTTATGTACAGCAACCCCTCAAGTATGGTTGGTGGTAACGGTACGTTACACCCTCTTAAACTTCAAAAATTGAACTCGAATAAATTATGTCCTTACAGACCCTACATGCAACGGTAATAATATTCTGTTCATCGATATACTAGACTCTCGAGTCAAGTAAAGTTTTCTTTTTGAAAGAGATAAGCATGACTTGGGGTTTGGCTTATATATAATATTAAATTTCTTTTCACCTTTGGGTTAAGAATTCACGTAGAAAAAATCAAAACACTTCAAGATAGATAGGTTAGTCATCTCGTTGTCAATTGTTAATATTTTGGGTATG

>Cucsa.248810

ATGGCTGATTTCCTATGGACTTTTGCTGTCGAAGAAATGTTGAAGAATGTGTTGAAGGTTGCAGGGGAGCAAACTGGCCTTGCATGGGGCTTCCAGGAGCATCTCTCCAACCTCCAAAAATGGCTACTCAACGCTCAAGCTTTCTTACGCGATATCAACACCAGAAAACTACATCTTCATTCTGTGAGCATCTGGGTGGACCATCTTCAGTTTCTTGTTTATCAAGCCGAGGATCTATTAGACGAAATTGTTTATGAACATCTTCGACAAAAGGTCCAAACAACAGAAATGAAGGTGTGTGATTTCTTCTCTCTTTCTACCGATAATGTTTTGATCTTTCGTCTTGACATGGCAAAAAAAATGATGACCCTTGTACAACTGTTAGAAAAGCATTACAATGAGGCTGCTCCTTTAGGACTAGTTGGGATTGAAACTGTAAGACCCGAGATCGATGTTATTAGTCAATATCGAGAGACAATTTCAGAACTTGAAGATCATAAGATTGCGGGGAGGGATGTTGAAGTTGAAAGTATAGTGAAACAAGTGATTGATGCTAGCAATAATCAACGTACATCTATCCTGCCCATTGTTGGTATGGGTGGATTAGGAAAAACAACTTTGGCAAAGTTAGTTTTTAACCATGAGTTGGTTAGACAACGTTTTGATAAAACTGTATGGGTTTGTGTGTCTGAACCATTTATTGTCAACAAGATTTTGCTTGATATTTTAAAAAATGTAAAAGGTGCCTATATTTCTGATGGAAGGGATAGCAAGGAGGTTTTACTTCGTGAACTCCAAAAAGAGATGCTTGGGCAAAGCTATTTTCTTGTGCTTGACGATGTTTGGAACGAAACTTTTTTTCTATGGGATGACTTGAAATATTGTTTGCTCAAGATCACTGGAAACTCTAACAATAGTATCCTTGTGACTACAAGGAGTGCTGAAGTTGCAAAAATCATGGGAACATGTCCTAGTCATCTTTTAAGTAAATTATCTGATGATCAATGTTGGTCTTTGTTTAAAGAAAGTGCAAATGCATATGGACTATCAATGACTTCAAACTTGGGGATCATTCAAAAAGAGTTGGTCAAAAAAATTGGTGGCGTACCATTGGCTGCACGAGTTTTGGGTAGGGCAGTAAAATTTGAAGGAGATGTTGAGAGATGGGAGGAAATGTTGAAAAATGTGCTAACAACTCCACTGCAAGAGGAAAATTTTGTTTTATCTATATTAAAATTAAGTGTGGATCGTTTACCATCATCTTCAGTAAAGCAGTGTTTTGCATATTGTTCAATTTTTCCCAAAGACTTTGTGTTTGAAAAACAAGAATTGATTCAAATGTGGATGGCCCAAGGTTTTCTTCAACCACAACAAGGAAGATACAATAACACAGCAATGGAAAATGTAGGAGATATATACTTCAACATCTTGTTGTCACGTTGCTTATTTGAATTCGAAGATGCCAATAAAACAAGGATAAGAGATATGATAGGTGATTATGAAACAAGAGAAGAATATAAGATGCATGATCTTGTACATGATATTGCAATGGAAACTTCAAGGTCGTATAAAGATTTGCATCTAAATCCTAGCAATATATCGAAGAAGGAACTTCAAAAGGAGATGATAAATGTTGCAGGCAAGTTACGCACAATTGATTTCATTCAAAAGATTCCTCACAATATAGATCAAACACTTTTTGATGTTGAGATAAGAAACTTTGTTTGTTTGCGTGTTTTGAAGATATCGGGTGATAAATTACCAAAGTCAATTGGTCAATTGAAACACTTGAGATATCTAGAAATTTTAAGTTATTCAATAGAATTAAAATTACCAGAGTCTATTGTTTCACTTCATAATTTGCAAACGCTAAAGTTCGTATACTCAGTGATTGAAGAATTTCCAATGAACTTTACAAATTTGGTAAGTTTAAGGCACTTGGAATTAGGGGAAAATGCTGACAAAACACCTCCACATTTAAGTCAATTGACTCAACTTCAAACATTGTCTCATTTTGTAATCGGATTTGAAGAAGGTTTTAAGATTACTGAATTGGGTCCATTGAAAAACTTGAAAAGATGTCTGTGTGTTTTGTGTTTGGAGAAAGTTGAAAGTAAAGAGGAAGCAAAGGGAGCAGATTTGGCAGGAAAGGAGAATTTAATGGCGCTACACTTAGGGTGGTCCATGAATAGAAAAGATAATGATTTGGAAGTGTTGGAAGGACTTCAACCAAACATAAATCTCCAATCATTGAGAATCACCAACTTTGCTGGAAGACATTTGCCTAACAATATTTTTGTTGAGAATTTAAGAGAGATACATTTGTCTCATTGTAATAGTTGTGAAAAGCTTCCAATGCTTGGACAACTAAACAACCTAAAGGAACTTCAGATTTGCAGCTTTGAAGGCCTCCAAGTTATAGACAACGAGTTCTACGGCAATGATCCAAACCAAAGAAGGTTCTTCCCAAAGCTTGAGAAATTTGAAATCAGTTATATGATCAACTTAGAGCAATGGAAAGAAGTAATAACAAATGATGAATCATCAAATGTCACAATTTTCCCCAATCTCAAGTGCTTGAAAATATGGGGATGTCCCAAATTATTAAACATTCCAAAAGCTTTTGATGAGAATAATATGCAACACCTTGAATCATTGATCCTTTCATGTTGTAACAAATTGACAAAACTCCCAGATGGATTACAATTTTGTAGCTCTATTGAAGGGTTGACAATAGACAAATGTTCAAATTTGAGCATAAATATGAGAAATAAGCCGAAATTATGGTATTTAATCATTGGTTGGTTAGACAAGCTGCCGGAAGATTTATGTCATCTCATGAATTTGAGGGTAATGAGAATTATTGGAATTATGCAGAATTATGATTTTGGCATCCTTCAGCACCTTCCTTCCCTTAAACAACTTGTTTTGGAAGAGGATTTGTTGAGCAATAATAGTGTAACGCAAATTCCTGAACAACTTCAACACCTCACTGCCTTACAATTTCTGTCTATTCAACATTTTAGATGCATTGAAGCTTTGCCAGAATGGTTAGGAAACTATGTATGTTTGCAAACACTCAATCTTTGGAATTGCAAAAAATTGAAAAAACTGCCTTCTACAGAAGCAATGCTACGTCTCACCAAATTAAATAAATTGCATGTTTGTGATTGTCCGCAACTACTACTTGAGGAAGGCGACATGGAGCGGGCGAAACTTTCCCACCTTCCAGAAATTCAGATCAATCGTTGGTTTATACAATTATTATGA

>Cucsa.251930

ATGGCTGATTTTATATGGACATTTGCACTGCAAGAGATTCTCAAGAAGACATTGCACCTTGCAACCCAACAAATCCGTCTGGCCTCCGGTTTCAACCACGACCTCTCTAAACTCCTCCACTCATTGCTCTTCTTCGAAGCCATTCTTCGCGATGTCGATCGAACAAAATCCGACCTACAGTCGGTCAAGATTTGGGTCACTAAGCTTCAGGATTTAGTGCTCGATGCTGAAGTTGTGCTGGACGAGCTCTCCTACGAGGACCTTAGGCGAGAAGTGGACGTCAATGGAAATTCGAAGAAAAGAGTACGCGATTTCTTTTCGTTCTCGAATCCCTTGATGTTTAGGTTGAAAATGGCGCGTAAAATTAGAACCATCACCCAAGTTTTGAATGAGATTAAAGGGGAGGCTAGTGCTGTTGGGGCTATTCCTACAGGGGGCAGTGATGAAATAGTGGCTGATAATGGCCATATTCCGGAGACTGACTCATTTCTTGATGAATTCGAAGTTGTAGGAAGAAGGGCTGATATATCTAGAATAGTGAACGTTGTTGTTGATAATGCCACTCATGAAAGGATCACTGTGATTCCTATTGTGGGAATGGGTGGTCTTGGAAAGACAACTTTGGCAAAAGCAGTTTTCAACCATGAGCTTGTGATAGCACATTTTGATGAAACTATTTGGGTGTGTGTGACTGCAACTTTTGATGAAAAGAAGATTTTAAGAGCAATTTTGGAATCTCTAACGAATTTTCCAAGTGGTTTGGATAGTAAGGATGCTATACTTAGAAGGCTACAAAAGGAGCTGGAAGGGAAAAGGTATTTTCTTGTGCTGGATGACGTGTGGAATGAAAATGTTAAACTGTGGAACAATTTCAAGAGTCTTCTGCTAAAGATTACAAATAGTATTGGGAACAGAGTTCTTGTGACAACTAGAAGTGAGGAAGCTGGAAAAATCATGGAAACATTTCCCAGTCATCATGTAGAAAAGTTATCGGATGATGAATGCTGGTCAATATTCAAGGAAAGAGCATCGGCAAATGGATTACCACTGACTCCAGAATTGGAAGTTATTAAGAATGTGCTTGCAGAGCAGTTTGGAGGCATTCCATTGGTTGCAAAAGTTTTGGGAGGGGCTGTACAATTTAAGAAAAGAACAGAGACTTGGTTGATGTCAACATTGGAAACCCTTATAATGAATCCACTTCAAAATGAAAATGACGTTTCATCTATTTTGAGATTAAGCGTGGATCATCTGCCAAACTCATCATTGAAACAATGCTTTGCCTACTTTTCTAATTTTCCCAAGGGTTTTAACTTTGAAAAGGAACAACTAATCCAATTTTGGATGGCAGAAGGGTTCATTCAACCTTCTGATAAAGTAAACCCCGAAACCATGGAAGATATAGGAGATAAATACTTCAATATCTTGCTGGCTCGTTCCTTATTTCAAGATATTGTTAAAGATGAGAATGGTAAAATTACACACTGTAAGATGCATCATCTTCTACATGATCTTGCTTATTCTGTCTCAAAATGTGAAGCACTGGGTTCGAATCTTAATGGTCTGGTTGATGATGTTCCTCAAATTCGACGATTATCCCTGATTGGCTGCGAGCAAAATGTAACGTTGCCTCCTAGAAGGAGCATGGTGAAGTTGCGTTCTCTATTTTTGGATAGAGATGTGTTTGGCCACAAGATTTTAGATTTCAAGCGTTTGCGTGTTCTGAACATGTCCCTATGTGAAATCCAAAACTTACCAACTTCAATCGGAAGGTTAAAGCATCTAAGGTATCTTGATGTCTCAAATAATATGATAAAGAAACTTCCAAAATCTATTGTTAAGCTTTATAAATTGCAGACCCTGAGGCTGGGTTGTTTCCGTGGAGAAGCCCCCAAAAAATTCATAAAATTGATCAGCTTGAGACATTTCTATATGAATGTTAAAAGACCAACAACTAGGCACATGCCTTCGTATTTAGGCAGGTTGGTTGATCTTCAATCCTTGCCTTTTTTTGTTGTTGGGACAAAGAAGGGTTTCCATATAGAAGAGCTTGGATACTTGAGGAATCTCAGAGGTAAATTAAAGCTTTACAATCTTGAATTAGTAAGAAATAAGGAGGAAGCCATGAGGGCAGATTTGGTGAAAAAGGATAAGGTGTACAAATTGAAACTGGTATGGAGTGAAAAAAGAGAAAATAATAATAACCATGACATTTCTGTTTTAGAAGGACTTCAACCACACATCAATCTTCAGTACTTGACAGTTGAAGCCTTTATGGGAGAACTTTTTCCAAATCTTACTTTTGTTGAAAATTTGGTACAAATTTCTCTAAAAAATTGTAGCAGATGTCGAAGAATTCCAACATTTGGACATCTACCCAATCTTAAGGTTCTTGAGATTTCTGGATTACACAACCTAAAATGTATAGGAACAGAGTTCTATGGGAATGAATATGGAGAAGGAAGTTTGTTTCCAAAATTGAAAAGATTTCATCTTTCAGACATGAATAATCTTGGACGTTGGGAAGAAGCAGCAGTGCCAACAGAAGTTGCAGTTTTTCCTTGTCTTGAAGAGTTGAAAATTCTCGACTGTCCTAGACTAGAAATTGCACCTGATTACTTCTCAACTCTTAGGACATTAGAAATTGATGATGTCAACAACCCAATTTCACAGATCACTCTTCAGACATTCAAACTACTTGGTATTATACACTCTGGCAACCTGAGTGGTTTGCCTGAGGAGTTACGTGGTAATCTGTCATCTCTTGAGGAGTTTAAGGTTTGGTATTATCTTCACTTGAAATCCTTTCCAACTATTCAGTGGCTCACTGATATTTTGAAAGGCAAGACCGGATATGACACAAAGTGGACAAATATTCAATCTCATGGGCTAGAATCGTACACTTCTGTGAATGAATTGTCCATTGTTGGGCACTCTGATCTCACATCAACCCCAGATATAAAAGCTTTATATAATCTTTCGTCTTTAACAATTAGTGGCTTGAAGAAATTGCCAAAAGGATTTCACTGCCTCACTTGCTTGAAAAGTTTGTCAATTGGTGGATTCATGGAGGGGTTTGATTTTAGGCCTCTTTTGCATCTCAAGTCTCTTGAAAATCTTGCAATGATAGACTTTATCCTTGCAGAAAGCACACTTCCTGATGAGCTTCAACACCTAACTGGCTTAAAGCACTTGAAAATTGTTGGATTTCAGGGCATTGAATCTCTGCCAGAGTGGTTAGGAAATCTTAACTCATTGGAAAGTTTGCACATTGAGAGTTGCAGAAAATTGAGAGAGCTTCCAGAAGCCATGGGTTGCCTTGCCAAATTGGAGGAAGTGCGGAGTTTCAATTGCCCAGAGTTGAGGGTTTACCAAGACGAATCAGAATGGGCGAAGATTTCTTACATTCCAAGATTCATATCATTCAATTATTGGGTTGATGAGTAA

>Cucsa.277260

ATGGCTGAATTTTTATGGACTTTTGCTGTTCAGGAAGTTTTGAAGAAGATTGTGAACTTTGGAGCAGAGCAAATTAGTTTGGCATGGGGTTTGGAGAAGGAGTTGTCCCACTTGAAAAAGTGGTTACTCAAAGCGCAAACAATCTTAGCAGACATTAACACAAAGAAATCACACCATCATTCTGTTGGGTTATGGGTGGAAGAACTTCATGATATTATCTATGAAGCTGATGATTTGTTAGATGAGATTGTTTATGAACAAATTCGACAAACTGTGGAGCAAACTGGTAAACTTAGAAAGGTACGTGATTCTATCTCACCATCCAAAAATTCCTTTTTGTTTGGTCTCAAGATGGCCAAGAAAATGAAGAAGATTACCAAAACTTTATACGAACATTACTGTGAGGCAAGTCCTTTAGGACTAGTTGGTGATGAATCCCCCACAGAATCAGAGGCTGCACTTAATCAGATTCGGGAGACAACCTCAATTCTTGACTTTGAAGTTGAAGGAAGGGAAGCTGAAGTCTTGGAGATACTAAAATTGGTGATTGACTCTACCGATGAAGATCATATCTCTGTGATATCCATTGTTGGAATGGGTGGTCTTGGAAAAACAACTTTGGCCAAGATGGTTTTCAATCATGATGCCATTAAAGGACATTTTGATAAAACTGTATGGGTTTGTGTGTCTAAACCATTTATTGTGATGAAAATTTTGGAAGCAATCTTTCAAGGTTTAACGAATACTAGTAGTGGTTTGAACTCCAGGGAGGCCTTGCTTAATCGACTCCGAGAGGAGATGCAAGGAAAAAAGTATTTTCTTGTGCTTGACGATGTTTGGGATAAAGAGAATTGCTTGTGGGACGAGCTTATTGGCAATTTGAAATATATTGCTGGAAAATCTGGAAATAGTATTATGGTGACCACAAGGAGTGTAGAAGTAGCGACCATGGTGAAGACAGTTCCCATTTATCATCTAAAAAAATTATCGGATGATCATTGTTGGGCGTTGTTAAAAAAAAGTGCAAATGCAAATCAGCTGCAGATGAATTCAAAGTTGGAGAATACGAAAAATATTTTGGTTAGAAAAATTGGTGGTGTACCACTCATTGCAAAAGTTTTAGGTGGGGCAGTAAAGTTTGAAGAAGGTGGGTCTGAGAGTTGGATGGCAAAAATTGAAAGCTTTGCGAGAAATATTTCAATAGAGGACAAAGATTTTGTTTTGTCCATATTAAAATTAAGTGTAGAGTCTCTCCCTCATTCTGCATTGAAGCAATGTTTTGCTTACTGCTCAAATTTTCCTCAAGATTATGAATTTGATAAAGATGAAGCAATCCAAATGTGGATAGCCGAAGGATTTATTCAACCCGAACAAGAAAGAGAAAACTTGACAATGGAGAACATAGGAGAAGAGTATCTTAACTTTTTATTGTCTCGCTCCTTATTTGAAGATGCCATTAAATATGATGGAAGAATTGTCACCTTTAAGATTCATGATCTAATGCATGATATTGCTTGTGCAATTTCAAATCATCATAAGATGGACTCAAATCCTATTAGTTGGAATGGAAAAAGTACAAGAAAGTTGCGCACATTAATTTGCGAGAATGAAGAAGCTTTTCATAAAATTCAGACTGACATTATTTGTTTGCGTGTGTTAGTCTTAAAATGGTTTGACACTAATACCTTGTCGACTATTATGGCGAAATTGATACATTTGAGATATCTTGATATTTCAAACTGTAATATAAACAAGCTTCTTCGAGATTCTATTTGTGCACTTTATAATTTACAAACGCTAAAACTTGGATATATTGAATGTGATCTGCCGAAGAATTTGAGGAACTTGGTTAATTTGAGACATTTAGAATTTAAGAAATTTTTTGATATGGGACAAATGCCTTCACATATGGGCAACATGATTCATCTTCAAACACTATCTGAGTTTGTAGTTGGACTTGAGAAGGGTTGTAAAATTGATGAGCTTGGACCGTTAAAAGACCTCAAAGGTACACTAACTCTTAAAAATCTACAAAATGTGCAAAATAAAGACGAGGCTATGGCTGCAAAATTGGTGGAAAAGAAGTATTTACGTCATCTAATCTTTCAATGGTTTCTAAATCTTTATGATAGAGGAGAATATGATGAAGATGATAACAAACAAGTGTTGGAAGGACTTCAGCCACACAAAAACGTACAGTCATTGGACATTAGAGGCTTCCAAGGAAGAGTTTTGAATAATAATATTTTTGTTGAAAATTTAGTTGAGATACGTTTGGTTGATTGTGGAAGATGTGAAGTGCTTCCTATGCTTGGACAGTTGCCCAACTTGAAGAAACTTGAGATTATTTCAATGAACAGTGTGAGAAGTATAGGCAGTGAGTTCTATGGAGTTGACTGTAACGACAGAAATTCTTCTGCTTTTCCTCAGCTGAACAAATTTCATATTTGTGGGTTGAAGAAGCTACAACAATGGGATGAAGCAACGGTTTTTGCATCAAATCGCTTTGGATGTCTAAAAGAACTTATTCTTTCTGGATGTCATCAATTGGCAAAATTGCCAAGTGGGTTAGAAGGGTGCTACTCCATTGAATATTTGGCCATCGATGGGTGTCCTAATTTAATGCTAAATGTGCAAAATTTGTACAACTTGTATCATTTAGACATTCGTGGGTTGAAAAGATTGCCAGATGAATTTGGTAAGCTCACTAACTTGAAAAAATTGAGAATTGGTGGATGTATGCAAAACTATGAATTTAGTCCCTTCATACATTTATCTTCTCAGCTTGTTGAACTTGAGTTGACTGATGATGGGTCAAGTGGTAGTGAAACAACCCAACTTCCCCAACAACTTCAGCATCTGACCAACTTGAAGGTTTTGAAGATTGCAGATTTTGATGACATTGAAGTTCTACCAGAATGGTTGGGAAACCTTACATGTTTGGCAACATTGGTTTTCCTCGAATGCAAAAATTTGAAAGAGTTACCTTCGAGAGAGGCCATACAACGATTAACCAAATTAGATGATTTGGTGATCGATGGATGTCCCAAACTACTACTAGGGGAAGGCGATCAGGAGAGGGCTAAACTTTCTCATCTCCCATCAAAATGTGTTCGTTACAATAATTTTGGTTTTAGATGTTAG

>Cucsa.318890

AATCAACTTACATCTATCCTACCCATTGTTGGTATGGGTGGATTGGGAAAAACAACTTTGGCAAAGTTAGTTTTCAACCATGAGTTGGTTAGACAACATTTTGATAAAACTGTTTGGGTTTGTGTCTCTGAACCATTTATTGTCAACAAGATTTTGCTAGATATTTTACAAAATCTAAAAGGCACCATTTCTAATGGAGGGGATAGTAAGGAAGTTTTACTTCGTGAACTCCAAAAGAAGATGCATGGCCAAAGATATTTTCTTGTGCTTGACGATGTTTGGAACGAAAATTCTTTTCTATGGGATGAGTTGAAATACTGTTTGCTCAAGATCACTGGAAACTCTAAAAATAGTATTGTTGTGACTACAAGGAGTGCTGAAGTTGCAAAAATCATGGGAACATGTTCTGGTCATCTTTTAAGTAAATTATCTGATGATCATTGTTGGTCCTTGTTTAAAGAAAGTGCAAATGCATATGGATTATCAATGACTTCAAACTTGGAGATCATTCAAAAAGAGTTAGTCAAAAAAATTGGTGGTATACCATTGGCTGCACGAGTTTTGGGAAGGGCAGTAAAATTTGAAGGAGATGTTGAGAGATGGGAGGAAATGTTGAAAAATGTGTTAAGCACTCCACTCAAAGAGGAAAATTTTATTTTGTCTATATTAAAATTAAGTGTGGATCGTCTACCGTCATCTGCATTAAAGCAATGTTTTTCATATTGTTCAATTTTTCCCAAGGATTTTGTGTTTGAAAAACAAGAACTAATTCACATGTGGATGGCACAAGGTTTTCTTCAACCACAAGAAGGAAGGAACATGACAATGGAAACTGTAGGAGACATATACTTCAAGATCTTGTTGTCACACTGCTTATTTGAAGATGCCCATGAAACAAAGACAGAGGAATATGAGATACCTGATCTGCTTGAATTTGAAACAAGGCCAGAAGAATATAAGATGCATGATCTTGTACATGATATTGCGATAGAAATTTCAAGAGATCAAAATTTGCAACTAAATCCTAGCAATATATCAAAGAAGGAACTTCAAAAGGAGATTAAAAAGGTTGCATGCAAGTTACGCATGGTTGATTTCATTCGACGGATTCCTTGCAATATAGGCCAACTAACATTTTTTGATGTTGAGATAAGGAACTTTGTTTGTTTGCGAGTTTTAAAGCTATCAACGCTGCCTAGTGATAAGTTACCGAAGTCAATTGGTCAATTGAAACACTTGAGATATCTAGAAATTGCATGTTATTTAGGTAGATTAAAATTTCCAGAGTCTATTGTTTCTCTTCATAATTTGCAAACACTAAAGTTTCTATACTCATACGTTGAAAAATTTCCGATGAACTTTACAAATTTGGTAAGCTTAAGGCACTTGAAATTATGGTCAAATGTTGACCAAACGCCTCCACATTTAAGTCAATTAACTCAACTTCAGACATTGTCTCATTTTGTGATCGGGTTTGAAGAAGGTTGTAAAATTACTGAATTGGGTCCATTGAAAAACTTGCAAGGTTGTTTGAGTCTTTTGTGTTTGGAGAAAGTTGAAAGCAAAGAGGAAGCCAATGGAACAAACTTGGCAGAAAAGGAGAAGTTAAAAGATCTACACTTAAGTTGGTCCAATGAAAGAAAAGATAACAACAATTACAATGATTTGGAAGTGTTGGAAGGACTTCAACCAAACCAAAATCTACAATCATTAGGAATCTACAACTTTGCAGAAAGACGTTTGCCTAACAAGATTTTTGTTGAAAATTTAAGCGTGATAGGTTTGTATGGTTGTAATAATTGTGAAAAGCTTCCAATGCTTGGACAATTAAACAACCTAAAGAAACTTGAGATTTACAGCTTCCATGGCGTCCAAATTATAGACAACGAGTTCTATGGTAATGATCTAAACCAAAGAAGGTTCTTCCCAAAGCTTGAGATATTTGTAATGTGTGATATGATCAACTTAGAGCAATGGAAAGAAGTAATGACAAATGATGCATCATCAAATGTTACAATCTTTTCCAATCTTAAATGCTTGGAAATACGTGGATGTCCCAAATTAACAAAACTTCCAAATGGACTACACTTTTGTAGCTCCATTCGACGTGTGACAATAAACCAAGGTTCAAATTTGAGCATAAATATGAGAAATAAGCCGAAATTATGGTATTTAAACATTGGTCCATTAGATAAACTGCCTGAAGATTTATGTCATCTAATGAATTTGGGGGTAATGAGAATTGTTGGAAATATGCAAAATTATGATTTTGGCATCCTTCAGCACCTTCTTTCCCTTAAAAAAATTACTTTAGTCGAGGATGAGTTGAGCAACAATAGTGTAACACAAATTTCTGAACAACTTCAACACCTCACTGCCTTGGAATTTCTGTCCATTGAAAATTTTGGAGGCATTGAAGCTTTGCCAGAATGGTTAGGAAACTTTGTATGTTTGCAAACACTCAGTCTTTATAACTGCAAAAATTTGAAAAAACTGCCTTCTACAAAAGCAATGCTACGTCTCACTAAATTAAATCAATTGTATGCTTGCAAATGTCCGATGCTACTACTCGAAGAAGGTGATCCAGAGCGAGCAAAACTTTCCCACTTTCCAAACATGTTGGTTCAGCGCAACGGTTATCTGAAGTGTATTTAG

>Cucsa.338110

ATGGCCAAGAAAATCAAGAACATTACCGATACTTTAAATCAACATTATTGTGCGGCAAGTGCTTTTGGGCTAGTTGGTGTGGAAACTGTCACAGAAATAGAGCTTGCGCTCAATCAGATTCGAGAGACAACCTCAATTCTTGACTTCCAAGTCGAAGGAAGGGAGGCTGAAGTTTTGGAGCTACTTAAATTGGCGATTGACTCTACCAATGAACATCATATGTCTGTGATATCCATCGTTGGAATGGGTGGTCTTGGCAAAACAACTTTGGCCAAGATGATCTTCAATCATCGTGAAATTGAAGGACATTTTGATAAAACTATATGGGTTTGTGTGTCAAAACCATTTATTGTCACAAAAATTTTGGAAAAAATCTTTCAGGGTTTAACAAAAACTTGTAGTGGGTTGGAATCCAATAAGGAGGCCTTGCTTGGGAGGCTGCGAAAGGAGATGCAAGACAAGAATTATTTTCTTGTGCTTGATGATGTTTGGGATAATGAGAAACACTTGTGGGACGAGCTTAGAGGCTGTTTGAAACATATTGCTGGAAAACCTGGAAATACTATTGTGATGACCACAAGGAATGAAGAAGTAGCGACGATGGTGGAGCCAATTTCTATTTATCGTCTAAAAAAGTTATCCAATGATCAATGTTGGGCGTTGTTTAAAGAAAGTGCAAATGCAAATCAGTTGCCAATGAATTCGAAGTTGGAGATTATGAAAAAGGAGCTGGTTAAAAAAATGGGTGGTGTACCACTCGTGGCAAAAGTTTTAGGAGGTGCAGTCAAGTTTGAAGAAACTGAACTTGAAGAGGAAGATCATGAGATCAGTTGGATGACAAAAGTTGAAAGCATTGTAAGGAACATTTCATTAGAGGACAAAGATTTTGTTTTGTCCATATTAAAATTAAGTGTGGATTCTTTACCAAATCCCGTGTTAAAGCAATGTGTTGCCTATTGCTCAAATTTTTCCCAAGATTATGACTTTCAGAAAGATGACCTAATTAAAATGTGGATAGCACAAGGATTTATCCAACCCGGACAAGGAAGAGATAAGAACTTGCTAATGGAGGATATTGGAGAACAATACTTCAACTTCTTATTGTCTCGTTCCATATTTCAAGATGTCACTAGGGATGCGAATAAGAGAATTGTTGGGTTTAAGATGCATGATCTAATGCATGATATTGCTTGTGCAATTTCGAGTCATCAAAATGTAGAATCAAATCCAAATAATTTGAGTGGAAAAAGTGTAAGAAAGTTACGCACGTTGATTTGCAATGATGAAGTGATTAATTATTTGAATCAGAAAGACATTGTTTGTTTACGTGTTTTAAAGGTTATTTTTCAATCGCATACGGATTTGTGGATTCCAATAGACAAGTTGATTCATTTGAGATATCTTGATATTTCAGAATGTTCTATAAACAAGCTTCTTCTTGAATCCCTTTCTCTTCTTTATAATCTACAAACGCTAAAGCTTGGACAAAGTGGTCTACCGAAGAATTTGAGAAAATTGGTTAACTTAAGACATTTAGAATTTAAAATGTTTGGTGATACAGCAATGCCTTCAGATATGGGCAACTTGATTCATCTTCAATCATTGTCTGGATTTTTAGTTGGGTTCGAGAAGGGTTGTAAAATAGAAGAGCTTGGACCGTTGAAAAACCTGAAAGGTAAACTAACTCTTACAAATCTCTGGAGAGTGCAAAATAAAGATGAAGCTATGGCTGCAAAATTGGTGGAAAAGAAGAACTTACGTCATCTAAACCTATGGTTTTTCGAAACCGATAAGAGAGGAGAAGATGATGAAGATGGTATAGTACAAGTGTTGGAAGGACTTCAACCACACAAAAACCTACAATCATTGGAAATCCTTGGTTTTCGAGGAAAAGTTTTGCCTACTGGTATTTTTGTTGAAAATTTAGTAAAGATACGTTTGGGTCATTTTGAAAGATGTGAAGTGCTTCCCATGCTTGGACAGTTGCCCAATTTAAAGGAACTTGAGATTATGTACATGGAAAGTGTGAGAAGTATAGGGAATGAGTTCTATGGAGTTGACTCCAGCCACCAAAATTCTGTTGCTTTTCCACAGTTAAAGAAAGTCAGCATTTATGAGATGATGAACCTAGAGCAATGGGATGAAGCAACGGTGGTTCTTGCATCAAATCTCTTTGGATGTCTAAAAGAAGTTAGGATTAGGAGATGTAATCCATTGGCAAAGTTGCCAAGTGGGTTGGAAGGTTGCCATTCCCTTGAATATTTGAGCATCCGTGGTTGTTTTAATTTGATGCTAAATGTGCAAAATTTGCACAAATTATACCATTTAGAGATTGATGGGTTGAAAAGATTGCCAAAGGGAATGGACGGACTCACTCGCTTGAAAGAGTTGAAAATTGGAGGATGCATGCAAAATTATGAGTTTAGTTCCGTCATACACTTGGCTTCTCAGCTTGTTGAACTTGAGTTGTCTGGCCGTTATGGGTCAGTTGACACCCAACTTCCCCAACAACTTCAACACCTCACTAACTTGCAAGTATTAAAGATTACACAGTTTGATTGCATTGAAGCTCTGCCAGAATGGATTGGAAACCTCATCTCTTTGAAAACATTGAAATGCTCCTATTGCTTTAAGTTGAAAGAATTACCTTCGAGAGAGGCCATATTACGCCTAACCAAATTAGAAAATTTGGACATTTTTGAATGTCCAAAGCTACTAGTTGGGGAAGGTGACCAGGAGAGGGCTAAGCTTTCCCATCTTCCATCAAAATGTGTTCATAAATCTGAGTAA

>Cucsa.102240

ATGGCGCTGGAATTGGTGGGTGGGGCTGTTTTGGGGGCTGTCGTTGGGGAGCTATTCAAAGCGATCTTGAATCTGGGTGAAAGGGCCATCAGTTTCAATCCTGTTCTTAAGGATATCCGTTCCAAGCTTAATGCTATAATGCCTTTGGTGAAGCAAATCGATGAGCTTAATGATTATCTCGATTACCCAAAAGAAGAAACAGAGAAATTGAGGGGTCTGATGGATGAAGGGAAGCAGTTGCTTCTCCAGTGCGGCGATGTGAAATTGGGGGATCTTAATTATTTGAAGAGACCATCTTACACCCAAAAGCTTCGGGAATTGGATACTGCACTTCGAAGCTTCATGGATGTTTTGATGTTGCAGATGGCTAGAGATCAGAAGAAGAACATGAAGATGATGAACCAAATGATGGAGATCATTTGTAGACTTGATAATAGAGGTGGGTCGAGTAAACCTATGGATTTGTTTGTTCCACCATGTCTGGTTCCTCAACTGCGAGAAGAAACCGTTGGGTTGGAGAAGCCAGTTAAGGAGTTGAAGGTGAAACTTCTCAAAAATGGGGTTCAAATGTTGGTGGTGACAGCTCCTGGTGGCTGCGGAAAAACCACACTGGCCTTAAAATTTTGCCACGACAAAGAAGTCAAAG

GTACGTCAAAATCATTCCTTTTGAGGGAAAAAATGAAAAAAGTTTGTCTACATATTGAGCTTAGGAAATGAAATTATATTAAACTATTGGGTGAAAAAAACTTAATGGCGTCTCTTACAGGAGCTTTCTAATTTCACGTCTTGATAG

ATATATTCCAGGAGAAGATCTTTGTCCCAGTTTCAAGAAAACCAGATTTGAAGCTTATATTGAAAGATATAATTGAAAGCCTTAGAGGAATTCAATTGCCTGATTTGCAAAGTGATGAACGTGCATTCTGCTATTTAGAATTGTGGTTGAAGCAGACAAGTGTAAATCGTCCTGTTTTGATTGTGTTAGATGATGTGTGGAGTGGGCAAGAATCTGAAGTTCTTCTTGATAAGCTGTTTCAATTGCCTTGCTGCAAGATCTTGGTCACTTCTAGGTTTTATTTCCCAAGATTTAGTGAGTCTTATTATTTGGAACCTTTGAACCATGAGAATGCAGTACAACTTTTTCGTCGTGCAGCATCACTGGACAAAGGAATTTCTAAGCTCCCCGATGATGAAACT

GTAGAAAAGGCAAACTTTCTATTATCTGATTAGCTCTACTTTTTCTAATATTTTTATATCTGTCAGTTTCTTTTAAATGGCTATGTTTAAAAAGCTCTTTGAAATTGAATTTCAG

ATAATTGGGGGATGCAAGAGACTACCTCTTGCACTGAAGGTAATCGGGAGGTCTCTTTCCCACAAACCGACATCTGTTTGGAAAGTAACGGGGAGGAATTTGGCTAGAAGTGGCTCCATATTTGATTCTGACAATGAACTTCTTGAATGCCTTCAGAGCAGTTTGGATGTCTTGGATGATAACATGGTAACTAAGAAGAGTTTCATGGATTTAGGCTCTTTTCATGAAGATCAAAGAATTTCTGCTTCTACCTTCATTGACATGTGCACAGTTTTGTACACACTAGACGAAAGTGAAGCAATGGTTACCCTTGACGAACTATCCTCTCGAAGTCTAGTTAATTTTGTCACAGCGAG

GTAAATACTATGCTCGAATGTTTGTAATTTCAGTTGCTGATAGTGTTGCCTGTATTATTGATGTAAAGAGAATAGAAATTGATTTGTTTTGTGGGGTTGTTGATCAG

AAAATATGGATATGATGATGACTTTTATGAAGAGTACTCTTTTACTCAGCATGATATTCTCAGAGATTTGGCTATTCACTTGATGAATATGGAGCCCATAGAACAAAGGAAAAGATTGATCTTAGACATTAATGGAAATGATCTTCCCAAATGGTGGGTTGATCAAGAAAAGCATACTTCCTATGCTCGCCTTATATCCATAACCACAG

GTTTGTCTCTTTCTCTGTGATATTCCATTTTCTTTCCTTTTTCTTTATCACTTTCACAACTTACTCAAAGGGGTTTTGGATGTTGCAG

ATAAGAGATTCTCAGCAAGTTGGCCTGACATGGAAGCACCTGAAGTGGAGGTTCTGATTCTTAATCTTCAGTCAAGAACTTACAACTTGCCTGGGTTCATCAAAAGAATGAATAAGCTGAAAGTTTTGATAATCACATATTTTGGTTCTTTTCTAACTGAGGTGACAAGTGAAGATAATCAACTACTCGACAGCCTAACAAGTCTTGAACGAATCAGGTTTGAGCGGATTTCAGTTCCTATCTTTAGTAATCCAAACCCGAAACCACTGATAAATCTGCAGAAAATATCCTTCTTTATGTGCAAATTTGGTCAAACATTCATGGATCCTTCAACCCCAATCTCAGATTTGTTGCCAAACCTGCTGGAGATTTCCATAGACTTCTGCAACAATTTGAGTGAAGTCCCCAATAGGTTGTGTGAAATTGTCAGCTTGCAGAAGCTGAGCATTACAAATTGCCATGGACTATCTTCCTTGCCAGAAGATGTAGGGAAGTTGATTAATCTAAAAAATCTAAGGCTAAGATCTTGCATTCATTTAGAAGAGTTTCCAGAGTCGACAACGAAGCTTCGGGAATTAGTCCTGCTTGATATATCTAACTGTATTGGTCTTGCCAAGCTTCCCGAGAAGATTGGTGAATTTCATAATTTAGAAAAGCTTGACATGAGACACTGCTGGAGTTTGAGCAAGCTGCCACTGTCGATTGGAAAGCTGAAAAATGTGAAGTTTTTATGTGATAGAGAGGTTGGAGAGTGGTTGAGAAAGGTTGCACCTCGCCTTGCCAAACAGGTGAAAGTGCAAGAGGAAGAAGCCAACCTGGAGTGGCTTGGTTTTTGA

>Cucsa.133510

ATGGCGGGAGCTTTAATTGGTGGCGCGGCATTGGGTGTTCCGTTTAACGAGCTAGCGACCCTCTTGAAGAATTTTGGCGAGAGGGCGTGGAGTTTCAATTCTGTTCTTAACGAGACCGAATCCAAGGTAAATGATATAATTCCTCTGGTTAAAGAAATAGATGGTCTTAATGAATCCCTGGATTATCCAAGAGAAGAAACGGAGAAGTTGAAAAACTTATTAGAATATGCTGGAAAGCTACTTAGACGGTGTTTAAGAGTGGGGAAGGCTGATTTGATAAGGAAATCAAGTCATACAGAGAAGCTTCGTGAACTGAATGCCAGAATCAAAAGTTTCAGTGACGTTGTGTTGTTCCAAACGTCTAGAGACGGGAAGAAGACATTGAGTTTAGTGACTGAGATCAAGGAAGTCGTTCGCAGGCTTGATAGCAAATCTGGATTAAGCAATCCGGTGGATTTAGTTGTGACGGTTCCTGTGATTTCAGAAGAAAGTGTTGGGTTGGAAAAGCCTGTTGAGAAATTGAAGGCCAAACTATTTAGAGATGGGGTTCGATTGTTGGTAGTGACAGCTCCCGGAGGTTGTGGAAAAAGCACTCTGGCCGAAATTTTTTGTCACGACAAGCAAGTTAAAA

GTAAATAACTTAACCATTCTTCTGAAAAATGTGTAGTACAATTACAAAAGTCCTTTGCAGTTCTCACAAATGCTTTCTGATTCTGCATTTTCACAG

ATAAATTTCAGAGAAACATCTTGTTCCTCGTTGTCTCAAGCAAACCAGAAACGAAACGCATCTTAATATCTATAATTCAAAGACTCGGGGGGCCTATAGAATCTGGTTCTGTAAGTGATGATGAGGCATTCCGGTTGTTAGAAGTTCGGGTGGGGGAATTGAGTCCAAATCCTGTATTGATTGTGTTGGACGATGTCTGGGACGGTTCTGAATCAAACAAGCTTCTTGAAAAGTTCTCCCGATTACCCAACTGCAAAGTTTTGGTCACTTCTAGATTTAAGTTTCCTGCATTTGGTGAGTCGTATGATTTGGAACCTCTGGACCATAAGGATGCAATGGAGTTGTTTCGTCGCTGGGCATCGAGGGGTAACAGAGTGCTACAGTTCCCAGATGAAAGAATT

GTAGAAAAGGTAATTAGATAATCTGCTAATTTGATTAAAAAGACCCCTAAAAGAATTTTTTTTCCTCACCTTAGAACTCATGGACACAGTTATACAACCTATAGAAAAGATCCAGTTCAAGTAAGGTCGTAAAAACTTTTTCCATACAAATGCAACTGAAGTTTTGAATGACTGAATTGCAG

ATAGTGAGGGGTTGTAAGAGATTCCCACTTGCTCTGAAAGTGATTGCAGGATCACTTTCGGGTAGAGCCACTTCGGTTTGGGAAGTTACGGGGAGGAAATTATCTAGAGGAGATTCTATTCTGGGTTCTGAGAAAGAGCTTCAGAAGTGCCTCAAAGACACCTTAGATGCAATCCCAGATGACAAGATAGTTCTCAAGGAGTGTTTCATGGACTTAGGTTCATTTCCTGAAGATCAAAGAATTCGTGCGGCTACCTTCATTGACATTTGTGCAGTGTTGTATGAACAAGATGAATGTGAAACAATGTCAAACCTTGATGAGCTCTTCACCCGGACTTTAGTTAACACTGTCTCTTTGAG

GTAAAATTCTGGGTCTTTCAAGTAGAGATGTCCACGGGTCGGGGGCCGGGACGGTGATGCACTCCCGTCGCTGCCCCCTACTCCCATATATATATATATATATGTTTAAACAGAAACAAATATATATATATATGTTTAAACAGACACATAATATATATGCTTAAACAGAAACACATATATATATATGCTTAAACAGAAACACACACACACATATATGCTTAAACAGAAACACATCTATATATGTTTAAACAGAAACATATATTATAGACCGGAGAATGTATTCCCGTCCCTAGCCCCAAAATGTCAACGTGAAAAAATAATCCCCACTCCCTTCCCCATTTCCTGTGTATTCGGTCCTCGTTCAGGGTGGGTCCGTTGCCCCGCAGGTTAAATGACATCTCTACTTCCAAGTTGTGTTGGTTTATGTTGATCTTGCTTCTTGGCTACATAATTGACGTTTTTTCTTAAATAAAAACTGTGTTTTATTATAGTTTGATATGACAATTCTGGCAAAAAATTGGTGTTTCGTTAGTATTTTGTGGATTATGTTGTAAAACATATGTACAAGAAACAAAGGGATTCTTTTGGGTATGGTTTTCTTTCAATTATTTACCAGAAGAATCTTGTCTAGAATTGATATGTGCCGATATATTTTCTTCCCATCGTTTTAGACTAACTTGAAGAACTATAAACATAAATTGATCATTGATGTACACTCAAAAAAGTATTTCAAATTATCTTGTGTAAACTCTTACTTTATGAACAGAAAATAAAGATTGCTTAGTAGAAACGTATTCTACTATTGATTTATCCTTTTGTTCTGGGAAACAGTCTCTAAATCAACTTCAAATTTGACTCTCAAAGATATTTTGAGTAATCATAATTACTAACTCAACCTTAATGCATAAGTATCTGAATATAAACTGCAAGACTGTGGACTGTGCTTGACATCCATTTGGTTATGAAATTGAAGTTCTAGTCTTGTTGTGATAATTTTCCAG

AAATAAAGCGCATGAAGATGATTACTACAGCGAGTCCTATATTACACAGCATGACGTACTTAGAGAATTGGCTGTCCTTTTGACTAATGAGCAGCCAGTAGACCAAAGAACAAGATTGCTTGTGGATATTAACAAAAATGAATTTCCCAAATGGTGGTCTGTAAGACAGATGCAACCTGTGAAAGCCCGCCTTTTGTCCATAACAACAG

GTCCCTCCGTGGTCTCTCTCTCATGTTTCGATATGTCATTTTTCATATTGTCGTTGTACAATCTAACACTGGATGAGATGTCGATGGTGCAG

ATGAGAAGTTCTCATCATGTTGGCCTGATATGGAAGCACCTGAAGTTGAGGTGTTAATTCTAAATCCTGGGTCAGAAACTTACAAGTTACCTGATTTTGCAAAGAAAATGAACAGATTGAAAGCGCTGATAGTCAGGAATTACAGGTCCTTTCCAACTGAATTGACAAGTGATTATCAATTAATCAATTGTTTGTCAAGGCTAGAAAGAATCAGTCTTGAGCGGATTTCAATATCTTCTTTCATTGACCAGAACCTGAAGCCCCTGTGGCATCTTAAGAAGCTATCGTTCTTTATGTGCAAAATTGACAAAGCTTTCACAGACTGCTCAACTCAGATCTCATACATGTTGCCTAACTTACTTGAGATCTCCATAGATTTTTGCAACGATTTGGTGGCTTTCCCTGTCGGACTATGTGAAGTTGTCACATTGGAGAAACTGAGCATTACAAACTGTCATGCATTATCTTCGTTACCCGAGGAAATTGGGCAGTTGATTAATCTAAAAATTCTAAGGCTTAGATCTTGTATTCATTTGGAGAAGTTGCCAGAATCAATCTCAAGGCTCCGGGAATTAGTTTATCTTGACATATCTCATTGTGTTGGCCTTACCAAACTTCCAGATAAGATTGGCAACTTGCAGAAGTTGGAAAAGCTTAATATGTGGAGTTGCCCGAACATGCGCAAGCTTCCAAAATCAGTAGGAAATCTAAAAAATTTGAAGGAAGTAGTTTGTGAAAGCGAGATGAAAATATGGGTGAATTTTGTCGCACCTCGGCTTGGCAATGTGGTAAAAGAACACAAGGAAGAAATCAACTTGGATTTTCTAAATTGA

>Cucsa.123410

ATGGCGGTTACAGATTTCTTTGTTGGAGAGATAGCCACTGAGCTTCTCAGAATGATGGTACAACTTTCGACCAAATCCTGCCTTTGTAAAACGACGGCAGCTCAAATCGCCAATTCTATTCAACAAATTCTGCCGATTATTGAAGAGATCAAGTACTCGGGAGTTGAATTACCCGCTCATCGCCAATTTCAGTTAGATCGCTTCAGCGAAACTCTTAGAAGAGGCATCGAGATTTCCGAGAAGGCTCTTCAATGTGGCCGATTAAACATTTACAGAAACTTACGGCTCGCGAGGAAGATGGAGAAGCTTGAAAAGGATATATGTCGATTCATTAATGGCACCATGCAGGCGCATATACTGGCCGACGTGCATCATATGAGATTCCAGACCACCGAGCGGTTTGACCGGCTTGAAGGTGTTTTGTTGGAGCGGCGGCTTGAGTCGATGAAGATTAGAGCAGATGCTTCGGGAGAGGAAAGGTGGTGGGTTGAGGAGGCGTTTAAGAAGGCCGAGGAGGAGGAAAGGTATGAGAGTAATTTCGTGAATATAGGAACTGGATTGCGTGTGGGGAAGAGAAAATTGAAGGAGCTGGTGATTGGAAAGGAGGATTTAACGGCGGTTGGGATTAGTGGAATTGGGGGTTCGGGGAAGACTACTTTAGCTAGAGAATTCTGCAAAGATCCGGAAGTTCGAA

GTGAGTTGTTTTTTTTTTTTATGTGGCTATTTTCTCTTGTTTTATGCGATGATTTCGATTTTCGGTTCAAAACTTAGTAGTTTTTCTACTGAATCTATATGGGTTTCTGACTTTGAAGCAAAGTAGATTAGAAAATTTTCACTTGAATTGGAAATTTCTTGTTAGTAAAGTGAATAGTTGTTAGAATATCTATTTGATACCGTAGCTCTTAAAAAGGTAAATAGGACATCCTTTTTTGTGTTTCTTCTTGTAGGAATTGTCGGATTCTTATTGTTCTTGCATGTTGATTGCCACCTTTTCAG

GACACTTTAAAGAGAGAATTTTGTTCTTAACGGTGTCACAGTCCCCTGATGTGGAGCAGCTGAGGAGAACGATCTGGGAATTTGTGATGGGTAGTGATAGTGTCAATTCTAATAATTTGATTTTACATGGGAGGCCTTCAAATTCAGCGCTTTTGGTTCTGGATGATGTGTGGTCAATTTCAGTTCTTGAAAATGTTATTCCAAACGTAACTGGTTGCAAAACTCTTGTTGTTTCACGATTCAAATTCCCTGAAGTTCTTAGAGAAACTTATGAAGTAGAGTTGTTGAAAGAAAGTGAAGCAATTGCTCTGTTTTGCCACTCAGCTTTCGGACAACAGTCGATTCCTTTGTCTGCTAATCACAACTTGGTCAAACAG

GTAAAGAACACAACATTCTTTCTGTTGGATTAGACACAAGGCAGTTATCTTTAAAACGTCTAATTTAGTGGTTTTATTTCACTCATTTGTTTGCAAATTTTCTTTGAAG

GTTGTGAATGAATGCAAATGTTTGCCTCTGGCTCTTAAAGTCATAGGAGCATCACTCAGAGGACAGAGCGAGATGTTCTGGAATAATGCCAAGTCTAGGTTGTCACGTGGCGAGCCTATTTGCGAGTCCCATGAGAACAAATTGCTTCAAAGAATGGCAATCAGTATTGAACGCCTCTCGAGTAAAGTGAGAGAATGTTTCCTCGACCTGGGATGCTTTCCTGAAGACAAAAGAATTCCTCTTGACATTCTCATCAATGTTTGGAAGGAGTTACATGATCTTGATGACGAAGAAGCTCTTGCTGTTCTTTTCGAGTTATCTCAGAAGAATCTTCTTACGTTGGTGAAAGATGCACGGTATGGACACAACTATGTTACATCACTTATTTACGTAATCATTACAGACATTGCAATAT

GTTGGCTAAATACTTGATTGGTTGATATGCAGCGGTGGTGACATTTATAGCAGTTATTATGAGATGTATGTCACTCAACACGATGTATTAAGGGACCTTGCCCTTCATTTCAGTTGCCAGGAGAATGTGAACGACCGCAAGCGATTACTGATGCCAAAAAGCGACACAGAGCTTCCAAAAGAATGGTTAAGGAAATCGGAACAGCCATTTAATGCCCAACTTGTTTCAATTCACACAGGTAACTGATTACCCTTTATCAAAGGAAGATAAGACTTGCAAAAATAAGCATCCTGTAAAGCTTTTTAGGCAAAGAAATCCCTTGTCTGAGTTACCAAGACATGGCATTTCTATGATTATAAGCTTTTTATACCATGTCCTTAGAAGTATCGTCTCATCTTCATTACACATTTATGTTATAG

GTGAAATGGAAGAAATGGATTGGGCGCCTATGATATTTCCTGAAGCTAAAGTGCTCATTTTAAACTTCTCCTCGAGTGGATACTTCTTGCCTTCTTTTCTTTGCAACATGCCGAAGATAAGAGCATTAATTGTGCTAAATAACAATGCAACACATGCAACTCTCACCAATTTCTCAGTTTTTTCTAGTTTGGTCAACTTGAGAGGCATCTGGCTGGAAAAAATTTCCATGACACAACTATTCGATGCTTGCACGCCATTGAAACATCTAAGGAAGCTATCTCTTGTTTTCTGCAAGATCAACAACAGCCTCGACGAGTGGGCGGTAGATGTATCCCAGATCTTCCCGTTTCTTTTCGAACTCAAAATTGATCACTGCAACGACTTGCGTAAGCTACCTTCAAGCATTTGTGAGATGCAAAGTCTCAAGTGTCTTAGTGTCACCAACTGTCATAATCTCAGTCAACTCCCTACCAACTTATGGAAGCTGAAAAATCTACAAATCTTGAGACTTTTTGCTTGCCCACTCCTCAAAACTCTATCCCCAAGCATTTGTGTACTTTCTTGTCTAAAGTACATTGACATCTCCCAATGTGTTTACTTAACCAGCCTTCCTGAAGAAATTGGCAAGCTGACAAGCCTAGAGAAAATTGACATGAGAGAATGCTCACTCATAAGGAGACTACCTAGATCAGTTGTGTCTTTGCAATCTCTCTGTCACGTAATCTGCGAAGAAGACGTCTCGTGGCTATGGGAGGATTTGAAGAGTCATATGCCTAATTTGTACATTCAAGTCGCCGAGAAATGCTTCAACTTAGATTGGCTCAAAGAGTGA

>Cucsa.178360

ATGGAGTTGTGTGCCGGTGCCATTGTTAATCCAATCGCAGAAAAAATCGCCAACTGCACGGTGGATCCGGTTTTCCGGCAACTAGATTATTTGCTCCACTTTAAAACCAATGTGAATGATCTCAAAGATCAAGGCAAGAAGCTGGTGGAAACCAGAGATTTTGTTCAACATTCTGTCGACTCCGCCAAAACCAATGGGTACGAGATCGAAGTTATGGTCACTGAATGGTTGGGGATAGCTGATCAATTTAGTGAAGATGTCGATAGGTTTTTCAACGAAGCCGACGGCCGAAGTCTTCGATGGTGGAATATGCTATCACGCCATCGATTTAGTAGAAGAGCTACCAAATTGGCTGTGGCAGTTGATAAAGCCATTCAAGGTGGGAGTTTCGAGAGAGTTGGGTTCCGTGTAACTCCACAAGAAATTATGACGCTAAGGAACAATAAGAAGTTCGAAGCCTTTGAATCTAGGGTTTTGATTCTGAAGGAGATAATTGAAGCGGTTGGCGATGCTAATGCGAGGGTGATTGTGGTACATGGGATGGCGGGAGTTGGGAAAACCACCCTAGTTGAAGAAATTGCAAGATTGGCCAAGGAGGGGAAGCTTTTTGATGCTATAGCAATGGTGACTGTAAAGCACATTCCAAACATTAAGAAAATACAGGGGGAGATTGCTGATCAATTGGGGTTGAAATTTGAAGAGGAAAAGGAACGAATTAGGGCCGATCGACTACGTCGAAGGTTAGAGATGGAGAAGAAGGTGTTAGTGGTTTTGGATGATGTTTGGAGTAGGCTTGATTTGGAAGCTGTTGGAATTTCTAGCCATCACAAGGGATGTAAGATACTTGTAACTTCTAGAAAGGATGATTTGTTTTTCAATGATTTTGGTACTCAGAAAAATATATATATCAATATTCTGTCAAAAAAAGAAGCTAGGGATTTTTTCAACAAGGTGGCATGTGATTCTGTTGAATCTTCTGATGATACTGATCCTGAAATGGAAGCTGTTGCTACTGAATTGGCAGATGAATGTGGAGGATTGCCACTTTCTCTTGCAACTGTTGGACAAGCCTTGAAAGGTAAAGGGCTTCCAAGTTGGAATGATGCCTTGCAAGGAATGAAGTTTCCTGGCGAACCCAGTAACTATGGGGTGAATAAAGTGGCATATTTGTCTCTGAAAGTGAGTTATAGATCTCTAAACAGAGAAGAAGCCAGATCACTATTCTTACTATGTAGCTTGTTTCCAGAAGATTATCAAATTAACATCAAATACTTGTTGATGTATGCCATGGGTTTGGGGTTATTAAACGCCATGAGTTCTCTAGCAATGGCAAAATGGAGAATACTTTCTTTGGTTGATGAGCTCAAAACTTCTCACTTGTTGCTTGATGGGGTTGATAACGATTTTGTGAAAATGCACGATATAGTTCGAGATACAGCAATTTTGATTGCGTCGAAAATGAAGTCCAAGTATTTGGTTAGACATGGTGCTGGAGAGAGTTTGTGGCCCCCAATGGATGAGTTCAAAGATTACACTGCAATCTCATTAGGTTGCAGTGATCACTCGGAACTCCCAGAATTTATATGTCCACAGCTTAGATTCTTATTACTGGTAGGAAAAAGAACATCTTTGCGATTACCTGAAAAGTTCTTTGCAGGTATGCAGGAACTACGAGTTTTAGATCTCACTGGCTTATGTATTCAGCGGCTTCCACCATCAATCGACCAACTGGTAAATCTTCAAACATTGTGTTTAGATGACTGTGTTTTGCCAGACATGTCTGTAGTTGGTGAACTGAAAAAGCTTGAAATTCTTAGCTTGAGAGCATCTGATATTATTGCACTTCCTAGAGTAATTGGGGAACTTACCAATTTGAAAATGTTGAATTTGTCTGATTGTTCTAAACTCAAGGTGATCCCTGCTAACCTTTTATCTAGGTTGATAGGGTTGTCTGAGCTATACATGGACAATAGTTTTAAACATTGGAATGTAGGACAGATGGAAGGTTATGTTAATGCAAGGATTTCTGAACTAGACAACCTGCCACGGTTGACCACTCTACATGTCCATATTCCAAATCCCACCATTCTACCACATGCCTTTGTCTTTAGAAAATTGAGTGGTTACAGAATACTAATTGGAGATAGATGGGATTGGTCTGGCAATTATGAAACTTCAAGGACCTTGAAACTCAAGCTTGATAGTAGCATTCAGAGAGAGGATGCAATTCAAGCACTTCTAGAGAATATTGAAGATCTGTATTTAGATGAATTAGAAAGTGTCAAGAATATTCTATTCAGTCTAGACTATAAAGGCTTTCCGAAATTGAAAGGTTTGCGTGTCAAAAACAATGGTGAAATTGTGACTGTTGTCAACTCGGATAACATGCATCATCCACACAGTGCCTTTCCATTGTTGGAGTCCTTATTTCTGAAAAATCTAGCTGAACTTGGAAGCATTTGTCGTGGAAAGCTTCCACAAATGTCCTTCCGTAACTTGAAAAGAGTAAAAGTTGAAAGTTGTGACAGATTAAAATTTGTTTTCCCATCTTCTATGGTCAGAGGCCTTATACATCTTCAAAGCCTGGAGATTAGTGAATGTGGCATCATAGAAACTATAGTTTCGAAAAACAAAGAAACAGAAATGCAAATCAATGGTGATAAGTGGGATGAGAACATGATTGAGTTTCCTGAATTGCGTTCTCTGATACTTCAACATCTACCAGCCCTTATGGGTTTCTATTGTCATGATTGCATAACTGTGCCTTCAACCAAAGTGGATTCACGTCAAACAGTTTTTACTATTGAACCTAGTTTTCATCCACTTCTCAGTCAACAG

GTATGTTATCTATAATTGTACTTCTTCCATTGTTATCTTTGAGCTAATATAGACTAAATATATGCTATAGTGTCTAGGTTTTTTTTAAAATTTAGTTCATGTAGTTTAAAAGCTCTAACTTTCATCTCTTATGTTTTGACATTGTCCAAATTATTACATTTTTGTCGGTTGTATTGATTGGTGATAATCTTACATATTTGGAGAGTTGGACAAAAATTTGAAGGAGGAACTTGGATTATTGATGAGTAAGGTTTTCTAAAAAGATTTTTTTTTTGCCAATTTCTTATAAGGTTAAGTATGAAGTAGAATTTATTTTAGTTGGCTTAATTCCTCCTTTATGTTTGTTCCAAATCACTTTCTATCGTGGCTAAATATATGTTTTGGTTGTCTTTTCAATTTGGTTTCTAATGTTTAAAAGTTTCAATTTACTCTTATTATGTTTTAACATTGTTAGAAAATATGAAATTTGCTTGTGATGAACAAACGTGATATGATACTAATTGAGTTGGTAGAAAGTTTGAGAATAAAATTTTTCGTGTAAGTGAATTTTTTTTTCAATTCAGCCTCTATATTTTAAGAAAGTTGCCAAAGCTATAAAAAACAAATTCTTTTAGGAAACGTAATTGTTCTTCCAAATTTCTCATGGTCATAAGCCAATTTGAAATTTTCTAACAGAAGGGTATAATCGAAACATATTAAAACAAAAAGGACTAAACTAAAAAATTTTAAATATTAGAAAACCGAAATATATATTTAGCCTAATTATAGTTGATAATTGTTGGAAACTAAAGACTCGTTTGGTAACAAGTTTCCTGTTTCCTGTTTTCATTTTTTAAGAAATGGAGGTGTTTGGTAACGTTTTATGTTTCTCGTTCTAAAAAAAGTAGAAATATTTATCATTTTATAAGGAATTATTGAAAACAAAAAAAAGTAGTTTCTTCTATTCTCGTTTTTTAATTATTTCTATTGGTTTCTTTTTCCTTTTTCTAAATTGTTTCTATTGGTTTTCTTCTCTTTTTTCTCAAACACACTCCTGTTTTTTGTTCTAAAAAAAGAAGGAACAGGAAACCGGAAACAAGGAACAATGAAAAAAACAGGGAACGAAAATGTTACCAAACGAGCCCTAAGGAATTGAGTTGAAAGTTTTCAGAATTTAGAGACTAAATTGAAGAAACACCCAAACATATATTTGGCCTTTTTATCTTTCTATGGCCTTTAAATCTACAAATCCAACATTGTGAAATAATATAATCAGCCATGAACGATAATGGATTCTGCTTGCTTTCTGGTTGGAATGTTTCTCGTATTCATTCTCACAATTATGTATTGACTATTGATAAAAAAATTGTTAAAAACTGTTTTTTAATTATTATTTATTTTTTTATCTTCTTAATGTTTTGTAG

GTTTCCTTCCCCAAATTGGAGACATTAAAATTACACGCTTTGAACTCAGGAAAGATATGGCAGGATCAACTTCCTTCTAGCTTTTATGGCTTTAAAAATCTAACTTCTTTGAGTGTGGAGGGTTGTGCTTCAATAAAATATTTAATGACAATCACTGTGGCTAGAAGCCTTGTGAATCTTGAACGCCTTGAACTAAACGACTGTAAGTTGATGAAAGCTATAATCATTTCAGAAGATCAAGATCTGGACAACAATTACCCTTCCAAATCTATCTTGCAGAACAAG

GTACATTTTTAACCTCATCATACTTCTTCCGTCTATGTTTTGTTTATAAACTTTAAGCTTAATAATGTCTGTCTTAAAAATTTTCATTTATGTCCATTAACTTTAAGTTGTGGACAATCTATTTAGTGCCTATTGTTAACATAAGCAAGGTGATATGGTCATTACAACCTAATCTCCAAATATCTGATGTGATATGGTTCATTTTTGCTAGTATAAGCTTTCATGGTTTTGCTTTTGAAATCACCAAAAAGGCTTCATACCGATGGAAATAATTGTCTTCAATACTTATGATCATTTCCCTTTCCCTGTGTATGTTTAGAATGCAAATATAACCTTCTTATGGGATAAAAACTAATTGGTTACATAACAAACAAGTGATAAATACAAGTGATCGGATCTAAGATAGGTCAATATAGATGGGTTAAGTTAATACTTTTATGTCAGCTTTTGTTAAGATGTGCTAAATCATGTTATAATATGTTTATAATCATGCCATATCCAAAAAAAGAGGTATATATATTATTATAATTTTCTAAAAAGAGGTTCAGAGGCTTCCAATAGAATATTCAAAACTCAAGGACTTCAACTTGGTCATATATTTAAACTTACCATGTTAGTTTGATTTTTGAGCAAAAGTTATAATTAAACTGAAATGATGAATATAATGCAG

GATGTTTTTGCGAACCTGGAGTCCCTCTTAATCTCTCGCATGGATGCTTTGGAGACATTATGGGTCAATGAAGCTGCTTCAGGATCCTTTACAAAGCTGAAAAAAGTGGACATCAGAAACTGCAAAAAACTTGAGACAATCTTTCCAAATTACATGCTTAACAGAGTGACAAATCTCGAGAGATTAAACGTTACAGATTGCAGTTCCCTAGTGGAGATCTTTCAAGTGAAAGTCCCAGTTAACAATGGCAACCAAGTAAGAGACATTGGAGCTAACCATTTGAAAGAGTTGAAGCTGCTTCGTCTACCTAAACTAAAGCACATATGGAGCTCAGATCCACACAATTTTTTACGCTATCCATCTCTCCAACTTGTTCATACAATTCATTGTCAAAGCCTTTTGAATCTCTTCCCTGTATCCATAGCTAAGGATCTCATACAACTTGAAGTGCTTAAAATACAGTTCTGTGGAGTTGAGGAAATTGTTGCGAAACGAGGAGACGATGGAGATGGAGATGATGCTGCGTCGTTTTTGTTGAGTGGTTTGACATCATTGACTCTTTGGAATTTGTTCGAGTTCAAGAGGTTTTATCCTGGGAAATATACTTTGGATTGTCCATCATTGACAGCGCTAGATGTACGCCATTGCAAATCATTTAAGTTGATGGAAGGAACTTTGGAAAATTCGTCATCAATCTCATCCGCTGTTGAAAAGGTATTACATTCTCTCTAA

>Cucsa.237070

AATGGGGAAAAAAAATGAAATTGATTAAAACTTGAGATTATGTTTAAATTTCAATTTTGGATCTTTATTATGAGTTTCTTTACCTTTTAAAGGAATCGAAATAGATGTCATTTATAAATCAAACCATCCCGATTCAACTTATACTTATTTTGTATTATATTATTTCTCTAGATAATAAAGTTGATTTTCTAATCTATCTATGAACGTTACTAACACGTTATTCAATGATTCACTCAAATCCGCGAGTCATTCTTCTACTTTGTTCTTCATTCAAAACATATAATTTACATCTTTTACTTTCTCCAAAGTCTACAGATGAAAAAGGATAGAAAAAGAGAATAATGGAAACTGTAATCGCAATTTTAGGGACAGTTTGTGAGTACGCAGTTGCACCCATTGGACGTCAAGTAGGATATGTTTCTTCCTACAAAAAGAACATCAATGATCTTAAAGACCAACTTCAAAATCTTGTGGATACTAAAACAAGGCTACAACACATGGTCAATGAGGCAAGAAGTAGTGCGTACAATATCCAAAGTGATGTTTCATCATGGTTGAACCAAGTAGATAAAATCATTGAACAATCCAACGACATATTGTACAAGAATGAAAATGAATCAAATAGCAAGTATTGTTCCAATAAGCTTAACTTCATTCATCAATATCAAATGAGTAAGAAAGCTAAGAAGATGGTGAAAGTAATTTCACAAATTATAGAGAAAAGAAAGTTGATGTTTCACCAAGTTGGTTATCCTACACCTCTTTCAAGGATTCATGGAAGTTCTACTAGTAGTTCTCATGGCTATGATCAGATTTTGGAATCAAGAACATCAATCGCCAAGCAAATTAGAGATGCACTCGTTGATTGTAACGTGAATAAGGTTGGAGTATATGGTATGGGAGGTGTTGAAAAAACTACGCTGCTGAAACAAGTCACACCATTAGTGATGGAAGAAAAATTGTTTGATCACGTGATTATAGTGAATGTAGGTCAAACATTGGGGGTAGAAGGCATACAAGCTCAAATTGGAGATAAGTTAAGGTTGGAATTAAATAAGAAGGTAGAGAGTAAGGAGGGAAGAGCATCTTTACTACAAAATAAGTTGGAGATGGAAAGTAACGTCCTCTTGGTGTTAGATGATTTATGGAAGGGACTCGATCTAGAAGAGGTTGGAATTCCTTGTAGATCAGAGTCATGTGAAAAGGGATGTAAGATACTCATAACAAGTCGAGATAGAGATGTCTTAACTAATGAAATGGACACACAAGTTTATTTCGAGGTGAAGCCTTTAAGTGAAAAGGAATCATGGGAGTTTTTCAAGAACATGATTGGTGAGTTTGATAATAAATGCATAGAACTAATAGGGAAAGAGATGGTGAAGAAGTGTGGGGGATTGCCAATAGCACTTGCTACAATTGTAAAAACTTTGAAGGGGAAGGAAGTGCCTATTTGGAAGGATGCTTTGAAGCAATTGAAAAATCCTATTGCAGTGGATGTTAAAGGGGTGACTGAGTTATGACTATCTAAAATGTGAAGAAGCTCGATTACTATTTCTTCTCTGTAGTGTATTCCCAGATGATTATGAGATTTCTGTGGAAGACTTGCAAATATATGCCATGAGTTTGAGATTGTTAAACCAAGTGAATACCTGGGACGAAGCTAGAAACAGGGTAATTAAATTGGTTGATGATCTTAAAGCTTCTTCTTTACTTCTAGAATCCAATTCAAGGGATAATCATGTTAAAATGCATGATATCGTTCGTGATGTGGCAATATACATTGCATCAAAGGAAGCTAATATGTCTACATTGAGCTATGGTTTCGGGCTGAGTGAATGGCAAGAAAAGGATAGACATGGATTCTATAGAGCAATCTTTGGAAACTGTCACAACTTCTACAACTTTCCTCAAAACTTAGAGTTTCCAAAACTTGAATTGTTGATATTAGATGGACATGATTGGAGGGGAGAAAAACTTCAAATTTGTTACTCTTTTTTTGAAGGAATGAAAGAACTTAAAGTTTTGAACTTGTCAAGGATGTGTTTTCAACTACTAAGGAGGCCATCAATCCACTCTCTAGAAAATCTTCAAACATTATGCATGTCACATTGTACATTCAATGACATTGATGCAATAAGTCACCTAAAGAAGCTACAAATTTTGAGGATTGATAAATGTCCAATCACATTGTTACCTAAAAGCATGAGTCAATTGACACAACTTAAGGTACTACAAGTGTCAAATTGCCCTTTGAAAGTGATTCCTCCAAACACCCTTTCAAGCCTTTTGAAGTTACAAGCACTAGATATATGGACAAGCTTCAATGGATGGGGAGAAGAAGTATCACACAATAACAAATTGATCAATAATGCAAGGCTATCAGAGTTGAAGTGTCTACCACATCTAACAAATTTAAAGATACATATCTTGGACATCAAAATTCTTTCAGATCTAATATTTCTGAAAAATTTGAAGCTAGAAAGATTTGTTATCCATGTTGGTGAATTGAAAATGTCCCAAAGGTTGCAAGGATGTGAACAATATGCAACAACTTTGATGCTTAAAATCATAACATCATCATCCCAAATTGTTTCAATCGATCATCATGAGGTACTTGAAATACTATTGAAACAATGTGAAAATTTGTGTGTGGAAGGATCAACAATGGCTACAAATATTCATTTCAAACCAAAAAATGGAAATAATTATCCATATTTGAGGCATCTTACTCTCACTAAAGATTCAAAGTTTATTTGATTGGAAATGGTTGCTTTGCTCATTTTCCTTCTTTGGAGTTCTTGTCCCTTGAAAAAATGGAGAGCTTGGAGAATATAGTTCATGCAGATGTGTTTACAAGTCCTTTCAGAAAGTTGAGATCCATAAAAGTAATAAGTTGTAAGAGATTAAGGTATCTCTTCTCTTTCTCTATCTTTAAAGGCCTTGTGGATCTACAAAGGGTTTTTATATTTGATTGCAACATGATGGATGAGATACTTTGCATGGATAGTGAAGACTCAACAATTGCAGTTGAAGGCAATTCTGTAAGTTCCCTTGCAAACTTCGTAACTTAGCATACAGATATCTTGTTTATATTTGTTTTTTTTATTCTTTTTCGTTTCTAAATTTACGTCTAAATACAATTGTGGTCCTTGTACTTGGGGTCGGATTAATTTACCTCAATGTACTTTCAATGAAGAAAAAAACAACCTTCTTGATCCCTAGGTTTTAGTGAGTCTAGTTCACATTTAGTCTTTATGTTTAAAATGTTACGTTTAGTTCGTAAGTTTTGAGTTTTTTTCAATTAGTCCATTTGTTTCAAACTGTTTGGTCTCTTGAAATTTGAGTTATGTTTTTATTTCAATTTGGTCCCTAGGAATCAAGACTTGCTCTCCTAGCTTCAATTTTGCACTAAATACTCAATCCCTGTTTTTAGTGTTAATATCAGAAGAATTATAATTAATTAAGACTATATTTAAGTACATATGAAACTGAAACTGAAATTTTAACCTCAAAATTTGCTATTTTAGGGAAAAAGAACTTTAGGTGTTATTTTTTTCTTATTTAAAAAGAAATTCTTCAACCATATACGGTTACGTTGTAGATTGAGTGTCCTCAATTGAAGGATTTGACAATCATTGGAGCACATAATTTGAAGATGTTATGGCATAAAAATGGGTTGGCTCCAAATTTCTTCAGCAAACTCCAAAGGATAAGTATCAATAGTTGCAACACTTTAAGATACCGTGGGGTTGGCATCAGTGGCATTGGTGGTGGAGTTGAAGGGTGATCAGCAGAATTGGCAGGACTGATGGTGGTAGTAGATTTGTTAAACTTCCTTGTAGTATTAAAAGTAGAGGGGGGTGGGTTTATAAACTTACAAGTGAAAACTTCAAACATAGTTTAATCTATCTATCCTGCAAAGTTAAGTATCACGCAACTAATTAATTCACCAATAAACTCTTTTATATATTAAAAATTCAAATCAGCACCCTCACCAATATGTAATTCTATGTTCTATAATAATAACTATTCTTGGTTTTTATAAGAAAAATAGACCTGCACACACTTGTCATCAGATCTCATTCACGA

>Cucsa.326910

ATTGACTTGTAACGGAGAAGAAAATTGTTAAAAGTTATTTATTTATTGACTTGTGACAGAAGAAAAATGTTAGAATGTGAAATTTTAAGTGGTCTATATTTTATAGCAAAAAAAAAAAAAGATAACAAAAATATTTAGAAATATAACCTAGTAAAATGTCATTGAAAAAGATGCATTACCTCATGGGACACCAAGTGGAAGCTTCATCGTTTGAAAATATTTGGTTTTGTTGAGATGCGTTTAATCTTTCAGTTTCCTTCATATGGGTGATGAAGGAAGGTGGAAAAAGAGTTTTAAGCGTCGGACACTTTGTAATCTCCACTTTCTTTACATTGGGGAAGAAGGTCTGAAGTTGTGGGATTTGGATCGGTATTCCAAACATGCTTTAAGTTTGGTAGAAAAGATAGGTGTAAGATCGTGAAGGGGACTAGTACTTGGTTTACATCACCTGCACCACCACTACCACTCCATTCTTCAATTTCAAATATCCTTTCTACTAACTTACAATGATGTACTTCTAATGTATTTAAGAAAACAAGTAACGTAAGCATATGTGATGGAAATACGTATGTTAATTTGTTGCAACTTGAAATGTACATATATTGGAGTGTGCAATAAGGAATTTGCAGGTAATCCATTATTACGCCATAACGTCTTCAAATTGTTCTCTCCTCCAATATTAAATCCATCAACTGAGGAAATGAAACCTGCGAATTGCATTTTAGAAATTAATTATATAAATTTGGTGTTTTAATTTAAAAAACGAAAGAAATGATATATATGTTATCCGTAATTAGTTTTACCTGAAGATCAAGATGCATGGGTTATTTTCAATCCCACGTGGCATGTCTAAGTATTGATTTTGATTATTGTGAATGTAGATTTGAAGTTCCTTCAAACGGGGATACCAATTATAATTTCCATATGGAACAGTATGGTTATTATTGCAAAGAAGGTTTGTAAAACCCACCGAATCTTTTACAACTAAAATCTCACATTTTTGCAACACTATTTGGAGTACTCCACCAATTGAATGAATGGATGAGTTTATACCGAGCCCCAAGCTTGGTGTGTATCTCTCCTTCCTTGTGAAGTACACATCAACATTACCAACACAAAAACTAAATTCTTCCAACTTTTCTGCTTTTTCTACACTCAGTTCGTTTAACAAGATCTCAACATTCTGAATCCTTAACTCTAAACTGAAAAGGCGTGTCAGACGGTTCAATTCGGAAAGTTCATTCATTTTGAAGCCATCAAATTTCAACTCTTCCAGTTGTGTCATACTTTCAATGATGTTTGGAGGAAACTCCGACGGATTAAAGTCGTCGCATTTCAACACGTGTAGCACCTTTAGTTTTATCAATTCTCACATACATATACCCCACTCTCTAATCTCACATTTTTCCACATCTTCAAAATTTTCAATTGCTTTAATACTTTGACTAATGCAATCAATATCAATAACTCCACAATCTGACAACCATATAATGCTTCAAGGTTGTTAATTGATGCCCAACTTGATGGTTCAAAACTTATACTCATAGTTTCCACAGACAAAACTCGAAGCGCTTTCATTCTTTCAAAAAATGCAGTAGGAATTCGAATTTTATTACCCTCCCCGATGGATGATGATCCACGATTTGTTAATATTAACAATTGAAGGTTCGGAAACTCCAACTTTTGGGGGAAGGTATACACAATATTTCGCACAATCTGCATAAATTGCTTTATAAGATTCATGCATATCATCATCTTCTTCTTGCCAGTATTCCAGCCTTTGCCCACTCCTATACGTCAAGGACGTCATCTTATCTTTCTTTGATGCAACGTATTTCGCAAAATCGTGGACCACATCATGCATTTGAACACAATCATCTGATCCTTTCTTGGACTGCTCATCTTGAAGCAAATAGCAAGATTTAAGATCATCAACCAACTTGATTACTCTATTCCCTGCATCTTCCCAAGTATTTATGTGCTTTACCAATCCCATACCCATTGCATACATCTGCAAATCCTTCACTTCAATCTTGTAATCATCTGGAAATACACTACATAGAAGTAATACTTGCTCTGCTTCATCTTCTGTTGAATCGTAACTGAATTGGAGACAAGAATACAATTGCTCACTCACTCCTGAAATAGTCACTGTAACAGGATTTTTCAATGACTTTAACGCATCCTTCCATATCTGTACTCTTTTTCCCTTCAATGCTTTTCCTAGAATTTTAAGTGCAAGTGGTAACCCTCCACATTCTTTTACCACATCCTCTGCTATTTGTTGTATATTATGTCCATCTTCAACACACGAAGACTCACCAACTATTGTCACGAAAAAGTTCCTAGACTCTTCGTTGGTAAGAGAATTCACCTCAAATGTCTTTTCTGTGTTCATTTCATTGTTTAATACATGTTTAACTTGTCATAAGTATCTTACATCCTTCTTTTCTTGATTCTGAATGACAAGGAATTCCAAACTCTTTTTCAACATCGTGTTCCTTCCATATATCATCCAACATAAACAACATATTCTCTTTCCTCTCGGCAATATGAGTTCGTAGGCGACATGCTCTTACCTCCTCACATTCTATATTCAATTCCATATTTAACTCATCTTTAATTTGTTCTTGAATATTGAATACACTTTTGGATTCACCAACTTCCACTTGAATCACTCGATCAAACAAATTCTTTTCCAACACTAACTTCTTAACTTCATTCAATAAAGCAGTTTTTCCAACACCACCCATACCACATACTCCAACTGTGTCGACATTAGGGTTTGCAAGTGCCTCTTTTATTTCTTCTACTATTGAAGTTCTTGACGCGATAATTTGATAATCGGTAGGGAGAGTGGGAGAATTAGTATCGGGAGAAGGTACGGGATAACCAACATTATCTTTGTTAAATCCATTTCCTTCATTTATGAGTTCAATAATATTTTCCACCCTCTTTTTTGCTTTTCTGGATAATTGATATCGTTCAACAAAATTAAAGCATGAGGGGTTTTCATTTTGTTGTGCTATCTCCAATACATCCTTTACAATTGTCAACCATTTAGAAACTCCATCAAAAACTGCATTCCCTTTTGCCTTTATCCTGACTGACCAAAATTTGGACACTGTCCTTAACAGTCCCAAGAGCTTCAACTTTCTTTTCAACTTCCTTAATATTTTGCTTGTAGTGGAACAGGTAATCAAGTTGGTGTAAAATGGGTATTTTGCTTGTAGTGGAACAGGTAATCAAGTTGGTGTAAAATGGGTTTAATTGTGTACTCAACTACTACCCCAACTACAGCATCCGCCATTTTTTCCCCCCTTCAAATTCTCCTCTCCCTGTAGATTTGGACATGGTCAAATGGAATGAAACTTTGTTGCCGAAACTACCTAAAATATTCCTAACGTTAATATTTTTTTTTAAAAAAAGAAGTACCTTTAAATTTGATGTTTCAAAATTTCGATATGCCCTTAAAATTTTAAAGAAAAATATATTTAAAAACTAAGGATTGGGTTGGGA

>Cucsa.017490

CTTGAAAATCTTGAGACTACTAAAAAGGATGTGAATCAAAGGGTTGAAGAGGCAAAAGGCAAATCGTATACAATCTCTGAGGAAGTTTCAAAGTGGTTGGCCGATGTGGATAATGCAATAACCCATGATGAGCTATCCAACTCCAACCCATCTTGCTTTAACTTGGCTCAACGATACCAGCTAAGTAGAAAAAGGGAGAAGCAAGTGAATTATATTCTTCAACTCATGAACAAAAGAAACAGCTTTGTCGAAGTTGGATATCGTGCACCTCTTCCGGATACTGAGAATACTGTTGTTCCCGGAGATTACCAAGTTTTGGAATCAAAAACATTATTGGCTAAAGATATCAAGAATGCGCTTTCGAAACCTGAGGTCAATAAGATTGGTGTTTATGGTATGGCAGGTGTTGGAAAAACTTATTTTCTTAACGAAGTTAAGAAATTGGTGTTGAAAGGGGAAGACAGATTGTTTGATCGAGTTATTGATGTGCGTGTAGGTCGATTTAATGATGTAACAGACATACAAGAACAAATTGGTGATCAATTGAACGTAGAATTGCCAAAAAGTAAAGAGGGAAGAGCGTCTTTTCTACGGAATAATTTGGCGAAAATGGAAGGTAATATCCTCATTTTATTAGATGATTTGTGGAAGGAATATGATCTTTTAAAAGAGATTGGGATTCCATTAAGTAAAGATGGATGTAAGGTACTCATTACAAGTCGATCACAAGATATATTAACCAATAATATGAATACACAGGAGTGTTTTCAGGTGAGTTCGTTATCTGAAGAAGAGTCTTGGAAGTTTTTTATGGCAATCATTGGTGATAAGTTTGATACAATTTATAAGAAAAACATTGCAAAGAATGTTGCAAAAGAATGTGGAGGGTTACCGCTTGCACTTGATACCATTGCAAAAGCATTGAAGGGGAAAGATATGCACCATTGGGAGGATGCTTTAACCAAATTGAGAAATTCTATTGGAATGGATATTAAAGGGGTGAGTGACAAAGTTTATGCTTCACTTAGATTGAGTTATGATCATCTAGATGGAGAAGAAACAAAATTAATATTTCTTCTTTGCAGCGTATTTCCAGATGATTATAAGATTTCTATAAAAAATTTGCAAATGTATGCCATGTGTATGAGATTATTGAATAAAGTAAAAACTTGGGAGGATTCAAAAAATAGGGTCATGAAGTTGGTTAATGATCTAATATCGTCTTCTTTACTTCTCGAGGCTGAGAGCGATTCAAAAGACAAGTATGTTAAAATGCACGATGTGGTTCGTGATGTTGCGATACACATTGCATCCAAGGAAGGTAACATGTCTACATTGAACATTGGATATAATAAAGTTAATGAATGGGAAGATGAATGCAGAAGTGGTTCTCATCGTGCCATTTTTGCAAACTGTGATAACTTAAACAATCTTCCCCTAAAGATGAATTTTCCACAACTTGAGTTGTTGATATTAAGAGTTTCTTATTGGTTGGTGGAAGATAATCTTCAAATTCCATATGCATTTTTTGATGGAATGGTAAAGCTCAAGGTTTTGGACTTGACAGGAATGTGTTGCCTCAGACCATTGTGGACAACACCATCATTAAACAACCTTCAAGCATTGTGTATGTTGCGTTGCGAATTTAACGACATTGATACAATCGGAGAGCTAAAGAAACTGGAAGTTTTGAGAATCGTTAAGTGTAACATGCTAGATCACTTACCTCCAACTATGAGTCAATTGACACACCTTAAGGTACTAGAAGTTTTAAATTGCCCTAAATTGGAGGTGGTTCCTGCAAACATTTTTTCAAGTATGACAAAACTCGAAGAATTGAAATTACAAGACAGCTTTTGTAGATGGGGAGAAGAAGTATGGTACAAGGATCGATTGGTCAAGAATGTCACAGTTTCAGAATTGAATTGTCTGCCATGTCTATCTAATTTAAGTTTAGAAAGTTGGAATGTTAAGATTCTATCTGAAATAAGTTCACAAACTTGTAAGAAGTTAAAAGAATTTTGGATTTGTAGTAATGAATCAGATGATTTTATTCAACCCAAGGTTTCTAATGAATATGCAAGAACCTTGATGCTTAACATTGAATCCCAAGTTGGTTCAATTGATGAAGGACTTGAAATACTATTGCAAAGAAGTGAGAGATTGATTGTAAGTGATTCAAAGGGTAATTTTATAAATGCAATGTTCAAGCCAAATGGAAATGGCTATCCCTGTTTGAAGTATCTATGGATGATTGATGAAAATGGTAATTCAGAAATGGCACATTTAATTGGAAGTGACTTTACTTCTCTAAAGTATTTGATTATTTTTGGGATGAAGAGATTGGAGAACATTGTTCCTAGGCATATTTCACTAAGCCCTTTCAAGAAGGTTAAAACTATTGCAATTCAATTTTGTGGGCAGATAAGGAATCTTTTCTCATTCTCTATTTTTAAAGACCTTTTAGATCTTCAAGAGATTGAGGTGATTAATTGTGGTAAGATGGAAGGGATTATATTCATGGAAATTGGAGATCAACTCAACATTTGCTCTTGTCCTTTAACTTCTTTACAACTTGAAAATGTGGATAAACTTACAAGTTTTTGCACCAAAGACTTAATCCAAGAAAGTTCACAAAGTATCATTCCCTTTTTTGA

GTCAGGTATGTACTATCAAAATTAAGTTGTTTTACTCTTTTTTAGTCCACTTTGTTTGTTTATATATAATTCACTTCAAACAAAATAATTTAACAAACAGAACTATGCATTTATTTACAAATTTGGTGAGATTTTGAAAAAGAAAAAATTAAAACTTTCTTATGATAATCATTTCACTTTTATTTAAAAGTTATTTGCTTTTTGTTTTTTTGAATATTTGGTTTATATTTGTACTACTTTTACCGCTTTAAAATTTATATGTTTTTGTTACCTAATTTTTTGTTTTTTATAATGCGTTTTAGAAAACAAAAGTTACATTTTTAAACTAGAAAGTTTCAAAATAGTTGTTGAAAAATTAGTCTATGTCCATTAAGACTTCAAGTCTTTCTTTAATCTTAAAGGCAAAAATCATAATTGATTTGAAAGAAACGAACACCAATTTTAAAAATAAAATTCTAAATATTAACCTTATAAAACACGTTTTAATTTTTAGTTTTTGAAAATTTTGAAAATTGCAAATGTTAATCCATCTAGTTTATTTGTTTGTTGTATTGTTTATTTTGATTTCAACTTGATATTTTTTAAAGGAACTAAATTTTTTAAAACTAGAAAAGAATTTAAAAAGGTGAAAACCACCGTAAAGAAATTGAGAAGAAATAAACAGAATTTTCAAAAATAAAAATGAAATTCTTCTTAAACCACTCTGACAAATTGAGAAGAAACATATTTAG

ATTATTTTAAGATTTTATTTTTGAAATGGCAGGTTTCATTTCCTGAGTTGAATGATTTATCAATTGTTGGAGGTAACAATTTGGAGACGTTATGGCATAAAAATAATAACCCAACTACAGGTTCCTTTTGCAAACTCCAATCAATAAGAATTGAACAATGCACTCAATTAAGATGCATGTTTCCTTCAAACATGTTGACATCACTTGCTTCGTTACATACAATACAAATCATTTCTTGCGCGTCATTAAAAAGGATATTTGAAATTGAAAACCAAAGTTTTAATGACACAACAGTTTTGTGGTCATTGAATGAGTTGCATTTACTCAATCTACCAAATCTCAAACACGTATGGAGAAAAGACATCATCAAAATTTTGACATTTCCATCTCTAAAGAGAGTAAAAATCCATGGCTGTACTAAGCTAACACACGTCTGGAAGGACAACAATAAAGTAACCAGAAGCTTTGATAGCTTGGAGAGGATTGAAGTAGAAAAATGCAAGAATTTGAAGTATTTACTGCCATCATCAATTGCATTCTTAAACCTGAAGGAGCTTCACATCAAGAAATGTAATGGAATGATCAATTTGTTCAGCTCTACAGTGACAAAAAAGCTAGTGAATCTCAGCTCCATTAAAGTATCTTATTGTAAAGGAATGAGATGCATGGTTGAAGTAGATCAAGCAGAAAATGATGAAATTATTACTTTCAAGAAATTGAGTACGTTGGAATTAGATTATTTACCACGATTGGATAGCTTTTACTCTGGCAAATGCATGCTTGAGTTTCCCTGTTTGGAGAGTTTGGTTATAAAAAGATGTCCTGAAATGAAGACATTTTCGTATGGAGTAATAATCGCGCCAAGATTACAAACCTTGTGGATGAACGATAAAGAATTTGGAGTATCATCACCAGCATGTGGGATAAATGAAACCATACAAAATTTTCCGAGGCGAGTGGTATGTATGTTCAATTCTAATTAACTATTTTTTTTAGTTGTTTTCTAAATATAACAAATTGAATCAAACTACTTAAAAATATAACATAATACATAATGTGAAAGTCTATAAAAATGGATTACATTTTAAAAATTAAAAGTATATTTTGATTTTTGAAATTTAAAATTCAAGTTTAATAAAACTCACAGTGAGAATTTCAAAGAAATGGCTTAGTTTCCAAAAACAGAAAAGTAAAAAACCAAATGGTTATAGAATGAAGTCGGTATTTCTATTTATCTCAAAAACAAACTTAGAACACGTAATAAATTTAAAATAATATAAGAAGATTATCTCTAAATTTTAATATGTTTACGAAGTTTAAAATTATTATGAATTTTAAACTATTCATTTATTGCTTCTTCCTTTAAATTTCTTAGCTTCAC

>Cucsa.239860

TTCTACAACAGAAACAAGAATGAGATTAAAGAGCAACTTGAAAGTCTTGAGACTACTAAAAAGGATTTGGATCTAAGGGTTGAAGATGCAAAAAGCAAGGCATATACCATCTTTACGAAAGTTTCAGAGTGGTTGGTCGCTGCGGATGACGAAATAAAGAAATCTGATGAGCTATTCAATTCCAACCCACCTTGCCTTAACTTTCTCCAACGACACCAACTAAGTAGAAAGGCAAGGAAGAGGGCGACGGATATCCGCCGACTCAAAGACGGAGGAAACAACTTTCTGGAAGTTGGTTGTCCTGCCCCTTTACCGGATACTATGAATACTATTGTTCCTGAAGCTTATCAAACTTTAGGATCAAAAACCTCAATGGCCAAGCAAATTAAGGACGCCCTTGCAAAACCTGAGGTAAGAAAGGTTGGAATCTATGGTATGGGAGGTGTTGGAAAAACATATTTGCTCAAGGAAGTTAAGAAATTGGTGTTGGAAGAAAAATTGTTTGATCTAGTGATTGATGTGACTGTAGGTCAATCTAATGATGTAATGAATATGCAACAACAAATTGGAGACTTCCTCAATAAAGAATTGCCAAAGAGTAAGGAGGGAAGAACATCCTTTCTACGAAATGCATTGGTGGAAATGAAAGGTAATATCCTGATCACATTCGATGATTTATGGAATGAATTTGATATCATAAACGATGTTGGAATTCCGTTAAGTAAAGAAGGATGTAAGACACTTGTCACAAGTCGTTTTCAAAATGTTCTAGCCAATAAAATGAATATAAAAGAGTGTTTTAAGGTGACTTGTCTAGACGATGAAGAGTCTTGGAAGTTTTTTAAGAAAATTATTGGTGATGAGTTTGATGCAAAAATGGAAAACATTGCAAAGGAAGTGGCCAAACAATGTGGAGGATTACCACTTGCACTTGATATCATTGCAAAAACATTAAAGAGATCAAGACATATAAATTATTATTGGGAGGGAGTGTTAAGTAAGCTGAAAAATTCAATTCCGGTGAATATTGACGTGGGTGAAAAAGTTTATGCTTCACTTAAACTAAGCTATGAACATTTGGATGGAGAAGAAGTCAAATCACTATTTCTTCTTTGTAGCGTATTTCCAGATGATCATGGGATTTCAGTAAACGATCTGCAAATGTATGTGATGGGTATGGGACTATTGAAAATGGTAAATACTTGGAAGGAAGCAAGAGCTGAAGCACATTACTTGGTCGAGGATCTTACATCATCTTCTTTACTTCAACGACTTAAGAATAGAGATGTTAAAATGCATGATATAGTTCGTGATGTTGCAATATACATTGGACCAGACTTTAACATGTCTACACTTTACTATGGATATAGTACAAGTAGCAAAGGGCTAGATGAGGATAAATGTAGATCTTATCGTGCAATCTTTGTAGACTGTAAGAAGTTTTGCAACCTTCTTCCAAACTTGAAGCTTCCAAAACTAGAATTGTTAATATTAAGTTTTCCTTTTTGGGGGAAAGATAGAAATATTGACATTATGGATGCATATTTTGAAGGAATGGAAAATCTTAAGGTTTTGGACATTGAAGGAACAAGTTTCCTTCAACCATTTTGGACACCGTTAAAGAACCTTCGAACGTTATGTATGTCATATTGTTGGTGTGAGGATATTGATACAATTGGGCACTTAAAGCAATTGGAAATTTTGAGGATTAGTAATTGTAGAGGCATCACAGAATTACCAACGTCTATGAGTGAATTGAAACAACTTAAGGTATTAGTTGTGTCGCATTGCTTCAAGTTGGTGGTGATTCACACAAACATTATTTCAAGCATGACCAAATTAGAAGAGTTGGATATACAAGACTGCTTTAAGGAATGGGGAGAAGAAGTAAGGTACAAGAACACATGGATTCCAAATGCACAACTTTCAGAATTGAATTGTCTGTCACATCTTTCTATTTTAAGAGTACGTGTTTTGAAGCTTACCATTCTCTCCGAGGCTTTGAGTTCACAAATGTTGAAAAACCTAAGAGAATTCTTTATTTATGTTGGTACCCATGAGCCTAAGTTTCATCCTTTTAAATCATGGTCGAGTTTTGATAAATATGAAAAAAATATGTCCTTTAATATGAAATCGCAGATTGTTTCAGTCAACGGGACGAAACTTAGCATATTATTAGAAGGAACTAAAAGGTTGATGATTCTAAATGACTCCAAAGGTTTTGCAAATGATATTTTCAAAGCAATTGGAAATGGTTATCCCCTGTTGAAGTGTCTTGAAATTCACGATAATTCAGAGACACCACATTTGAGAGGAAATGATTTCACATCTTTGAAGAGGTTGGTTCTTGATAGAATGGTGATGTTGGAGAGTATTATTCCGAGGCATTCTCCAATAAATCCTTTCAACAAACTTAAATTCATAAAAATAGGAAGGTGCGAGCAGCTAAGGAATTTTTTTCCACTCTCTGTTTTTAAAGGGCTTTCAAATCTTCGACAGATTGAGATCTATGAATGTAATATGATGGAGGAGATTGTATCAATAGAAATTGAAGATCATATCACTATTTACACTTCTCCTTTGACATCTTTACGCATCGAGCGTGTGAATAAACTTACAAGTTTTTGCAGTACCAAATCATCCATCCAACAAACAATT

GTTCCCTTATTTGATGAACGACGGGTATGTGGACCATAAAACTCAGCTCTTTTGCCTTCTATTTGTTGGTGTGATAAATAAATTATGTTTGTTTGTAGCTATTTATTGGGTAATTTACATTAATTTGAAGACTAGTACTATTTTCAAATAATTTGACGGAGGTTTTAATTTGAAAAGAAAAAACTGAGAAATTAGTTTGAAAACATGTTAATTTAG

ATTTTATGTTATGATAATTATATTGATATATATATTTTTTTTGTATCTGAGGAGATTATAGTAAAATTATTTCAGGTTTCATTTCCTGAATTGAAGTATTTATCAATTGGTAGAGCAAACAATTTGGAGATGCTATGGCATAAGAATGGAAGTTCCTTTTCCAAACTTCAAACAATAGAGATTAGTGATTGCAAGGAGTTGAGATGCGTGTTTCCTTCAAATATAGCGACGTCACTTGTCTTTTTAGATACATTGAAAATCTATGGTTGTGAGTTATTGGAAATGATATTTGAAATTGAAAAGCAGAAGACTTCGGGAGATACAAAAGTAGTGCCATTGAGATACTTATCTTTAGGATTTCTAAAAAATTTAAAGTACGTGTGGGACAAAGATGTTGACGATGTTGTGGCATTTCCAAACCTAAAGAAAGTTAAGGTTGGTAGATGCCCTAAGTTGAAAATTATTTTTCCAGCTTCCTTCACCAAATATATGAAAGAAATTGAAGAGTTAGAAATGGTTGAGCCGTTTAATTATGAAATATTTCCAGTGGATGAAGCATCAAAGTTAAAAGAGGTAATTCATACATCATGTCATCAATGTCTGTATGTAGGCAAGAATTGTATATGCTTCAATTTGATACTAATTAACTTCTATTAT

GTTAATCTACAAAGAATTTTGTTATTACATTTTTTTTTTGGTGGAGCCAAATATATATATGTATGTGGATATTAAAAAAATAATGGTTTAGAGACTTTGTGTGTGTATTTCAGGTTGCATTGTTCCAAAGCTTGGAAACATTGAGAATGAGTTGTAAG

CAGGCTGTAAAAGAGAGGTTTTGGGTTATGTCAAAGTTCTTCAAACTCAAAAGTCTTGAATTGTTTGGTTGTGAAGATGGTAAAATGATTAGCTTGCCGATGGAAATGAATGAAGTATTATACAGCATTGAAGAATTGACAATTAGAGGATGCCTCCAGCTGGTAGATGTAATTGGAAATGACTATTATATCCAAAGATGTGCAAATTTGAAGAAGTTAAAATTGTATAATCTTCCGAAGCTTATGTACGTGTTGAAGAACATGAATCAAATGACTGCAACCACATTCTCCAAGTTGGTTTATCTTCAAGTAGGTGGTTGCAATGGAATGATAAATTTATTTAGTCCTTCAGTGGCAAAGAATCTAGCGAATCTCAATTCCATTGAAATATATGATTGTGGAGAAATGAGAACCGTAGTTGCAGCAAAAGCAGAGGAAGAAGAGGAAAATGTTGAAATTGTGTTCAGCAAGCTAACTGGTATGGAATTCCATAATTTAGCAGGATTGGAATGTTTTTACCCTGGAAAATGCACACTTGAATTCCCCTTATTAGATACGTTGAGGATAAGCAAATGCGATGACATGAAAATCTTTTCATACGGAATAACAAACACTCCCACTTTGAAAAACATCGAGATTGGAGAACATAACTCATTGCCAGTATTACCAACACAAGGGATAAATGACATTATCCATGCTTTTTTCACAATTGAGGTATGCATTATTTCATTTCAAAATACACTAAATAGTATTTCTCAATTTCTTGCTCAATTATATTATCAAAATCCACTTTGTGTGTGA

>Cucsa.088220

AACTTTCAAAAACTTAAGACTCAAGTAGAAAAGTTGAAAGATACAAGAGAATCTGTGCAACAAAACATCTATACTGCAAGAAGAAATGCTGAAGACATAAAACCTGCTGTTGAGAAATGGTTGAAAAACGTTGATGACTTTGTTCGAGAATCTGACAAGATATTAGCCAATGAAGGTGGACATGGTAGACTGTGTTCCACCAATTTAGTCCAACGACACAAGTTAAGTAGAAAAGCAAGCAAAATGGCGTATGAGGTTAATGAGATGAAAAATGAGGGGGAAGGTTTTAATACGGTCTCCTATAAAAATGCTATTCCATCGGTTGATTGTTCACTGCAAAAAGTATCTGACTTTCTTGACTTAGACTCAAGAAAATTGACTGCGGAACAAATCATGGATGCACTCTCTGATGATAATGTCCATAGGATTGGAGTGTACGGGATGGGGGGTGTTGGTAAAACAATGTTAGTGAAAGAAATTTTAAGAAAAATTGTTGAGAGTAAGTCTTTCGATGAGGTGGTAACATCCACGATCAGCCAAACACCAGATTTTAAAAGTATTCAAGGACAACTAGCTGACAAGCTAGGTTTGAAATTCGAACGAGAAACAATAGAAGGAAGGGCACCTAGTCTACGAAAGAGGTTGAAGATGGAGAGACGTATCCTAGTTGTGTTGGATGATATCTGGGAGTATATTGATTTGGAAACAATAGGAATTCCAAGTGTTGAAGATCATACAGGATGCAAGATATTGTTTACCTCTAGGAATAAACATTTGATCTCAAATCAAATGTGCGCCAATCAAATTTTTGAGATAAAAGTTTTAGGAGAAAATGAGTCATGGAATTTATTTAAGGCAATGGCTGGTAAAATTGTTGAAGCAAGTGATTTGAAGCCTATAGCCATTCAAGTTGTGAGAGAATGTGCAGGTTTGCCTATTGCTATTACTACTGTTGCTAAGGCATTACGAAATAAACCTTCCGACATTTGGAATGATGCCTTGGATCAACTTAAAAGTGTTGATGTGTTTATGACAAACATTGGAGAAATGGACAAGAAAGTGTATTTGTCACTAAAATTGAGTTACGATTGCTTGGGATATGAAGAGGTCAAGTTATTATTCTTGTTATGCAGCATGTTTCCAGAAGACTTTAGCATTGACATGGAAGAGTTGCATGTATATGCCATGGGCATGGGTTTCTTACATGGTGTTGATACTGTGGTAAAAGGACGACGTAGGATTAAAAAATTGGTTGACGATCTTATATCTTCTTCTTTGCTTCAACAATATTCTGAGTATGGGTACAATTATGTGAAAATGCATGATATGGTTCGTGATGTAGCTATATTTATTGCGTCTAAGAATGATCACATACGTACATTGAGCTATGTGAAAAGATTAGATGAAGAATGGAAAGAAGAGAGACTATTGGGTAATCATACCGTGGTGTCCATTCATGGTTTACATTATCCTCTCCCAAAGTTAATGTTACCCAAAGTTCAATTATTAAGGTTGGATGGACAATGGTTGAATAATACGTATGTGTCAGTGGTACAAACTTTTTTTGAAGAAATGAAAGAGCTCAAAGGTTTAGTATTAGAAAAAATGAATATATCCTTGTTGCAACGACCATTTGATCTTTACTTCTTAGCAAACATTAGAGTGTTACGTTTACGGGGATGTGAATTAGGGAGCATAGATATGATTGGTGAATTAAAAAGGCTTGAAATTCTCGATCTTAGTGGATCTAACATCATCCAGATTCCTACAACAATGGGTCAATTGACACAACTGAAAGTGTTAAATTTATCTAATTGTTTTAATAAGCTCGAGATAATTCCACCAAATATTCTTTCAAAGTTGACAAAACTGGAGGAATTACGTATGGGAACTTTTGGTAGTTGGGAAGGAGAAGAATGGTATGAAGGAAGGAAAAATGCTAGTCTTTCCGAGCTTAGGTTCTTGCCACACCTTTTTGATTTAGATTTAACCATTCAAGATGAAAAGATTATGCCAAAACACTTGTTTTCAGCAGAGGAGTTGAATCTTGAAAAATTCCACATTACTATTGGTTGTAAGAGAGAAAGAGTTAAAAATTATGATGGAATCATTAAGATGAACTACTCTAGAATATTGGAAGTCAAGATGGAATCAGAAATGTGCTTGGATGATTGGATAAAATTTTTGTTAAAGAGGTCAGAAGAAGTGCATTTAGAAGGATCAATTTGTTCAAAGGTTCTTAACTCTGAACTGTTAGATGCAAATGGCTTCTTACATTTGAAGAATCTCTGGATTTTTTATAATTCAGACATTCAACATTTCATCCACGAAAAGAACAAGCCTTTGCGAAAATGCTTGTCCAAATTGGAGTTCTTATATCTTAAGAACTTGGAGAATTTGGAAAGTGTAATTCATGGTTATAATCATGGTGAATCTCCTTTAAACAATTTGAAGAATGTAATCGTATGGAATTGCAATAAATTGAAAACCCTATTTTTGAATTGCATGTTGGATGACGTTTTGAATCTCGAGGAAATTGAGATCAATTATTGTAAAAAGATGGAAGTGATGATCACTGTGAAGGAAAATGAGGAGACAACCAACCACGTTGAGTTTACTCATTTGAAATCTTTATGTCTATGGACTTTACCACAACTTCATAAATTTTGCTCCAAAGTTAGCAATACCATCAACACATGTGAATCATTTTTCAGTGAAGAG

GTACATTCCATCAACACATATTTCTATGTTTAATTTCTATATACACATCTAAATTAAATTACCCTTTTGTTCATTTTATTGTTCCTCCCACAAGTTCCTTCTAGTATTTGTGGTTAGTAATGATATCAGGGATCAAATTTTATTCTTCTTTGTTTTAGAAGACACTCTTCTCTCAGTGTAGAGATGATGAACCCTTTCTTGTTTCATCCATTAATAATATTATTTTTAAACTACTGAAGTATATAAATGCATGTTAGGTGATGTTCCAGGAAGATTAAAAGAAGTATATTTTAGAATTTTCCTATCCAAATAAGCTGATTTGAAGTAATCGTTTTGCACAATTTGTTATGGTAATTTTATAAGACTATATACATTATATGTTGTTAATTAGTATCAGCTGTGATGATTGTTAATTACGTTGTTTCAAACTGAATTTTCAAGTATAAAAATTTGTAG

GTATCGCTTCCTAATTTGGAGAAGTTGAAAATTTGGTGTACAAAGGATTTGAAGAAGATATGGAGCAATAATGTACTCATTCCCAATTCCTTTTCCAAACTTAAGGAAATAGACATTTATTCATGCAACAATCTTCAAAAAGCATTGTTCTCTCCAAATATGATGAGCATTCTTACTTGCCTTAAAGTCCTAAGGATTGAAGATTGTAAATTGTTGGAAGGAATATTTGAAGTGCAAGAGCCAATTAGTGTTGTTGAAGCAAGTCCTATCGCCCTCCAAACTTTGAGTGAGTTGAAACTATATAAACTTCCAAACCTTGAGTACGTATGGAGCAAAGATTCCTGTGAGCTTCAGAGTTTGGTAAATATAAAACGTTTAACCATGGATGAATGTCCAAGACTTAGAAGAGAATATTCAGTCAAAATTCTCAAGCAACTTGAAGCACTAAGCATAGATATCAAACAATTGATGGAGGTTATTGGGAAGAAAAAGTCGACGGATTATAACAGGTTGGAATCAAAGCAATTGGAAACTTCTTCTTCCAAG

GTACATTATATATATATATATATATATCTTACAACACATCATAATTAAATTGTTATCACTAAATAATCACTTTCTAGGGAAAATCTTGTGGAGTGAAATATTGTTATTGGAGGTTCAATTGTTTATGTGAAAAAAGAATAAGCAACAAATCTCTTAAAGTAAAAGCTATGAATATAACAGTGTGATTTGTTTCTTTTTTCATGTAGATTGTGTACTTAGAGAAGTAAAAATATTTTTGAATTTGTTCTACTTTATAGAGCATATATATTCAGTTTAAATGTTATATTGCTTGTTAATTGTTATATATAGAATCTTAATTAACAAATTAGTAATTGGTCAG

GTTGAGGTTCTACAGTTGGGAGATGGTTCTGAGTTGTTTCCGAAGCTTAAAACTTTGAAGCTATATGGTTTTGTTGAGGATAACTCAACCCATCTGCCAATGGAAATTGTACAAAACTTATACCAATTTGAGAAGTTTGAATTAGAAGGAGCATTTATTGAAGAAATTCTCCCCAGCAACATATTGATTCCTATGAAAAAACAATACAATGCAAGACGATCTAAAACTTCACAGCGTAGTTGGGTTCTATCCAAGCTACCGAAACTTAGGCATTTGGGGAGTGAATGCTCACAAAAGAATAATGATTCAATTCTACAAGATCTGACCTCTCTGTCCATTTCAGAATGTGGTGGATTGAGTAGTTTAGTGTCATCATCGGTGTCTTTTACGAACTTGACGTTTCTTAAATTGAATAAATGTGATGGACTAACCCATTTGCTGGATCCTTCGATGGCTACAACGCTTGTGCAACTTAAACAGTTGAGAATAGGAGAATGCAAAAGGATGAGTCGTATAATTGAGGGAGGATCATCAGGTGAAGAAGATGGAAATGGTGAAATTATTGTATTCAACAACCTACAACTTTTAATCATTACTTCTTGTTCCAACCTAACAAGCTTTTATCGTGGAAGATGCATCATTCAATTTCCATGTTTGAAACATGTATCTCTTGAGAAGTGTCCTAAAATGAAGTCATTTTCATTTGGAATTGTAAGTACATCTCATTCAAAATATGAAAATGTTTCTTTAAAGAATGA

>Cucsa.091880

ATGGAAAGTATTCCTATTTCAATAATTGCAAAAATTTGTGAATACACTGTTAAACCTGTTGGACGTCAACTTTGTTATGTATGTTTCATTCATTCCAACTTTCAAAAACTCAAGAGTCAAGTAGAAAAGCTGACAGATACAAAAGGATCTGTGGAAGACAAGGTTTTTATTGCAAGAAGAAATGCAGAAGACATAAAACCTGCAGTTGAGAAATGGTTGGAAAAGGTTGATCGCCTTGTTAGAAAATCTGAGAAGATACTAGCCCATGAAGGTAGGCATGGTAGATTGTGTTCCACCAATTTGGTCCAAAGACACAAGGCAAGTAGAAAAGCAAGCAAAATGGCAGATGAGGTTCTTGAGATGAAAAATCAGGGAGAAAGTTTTGATATGGTATCCTTTAAAGGTCGTATCTCATTGGTTGAGAGTCCACTGCCAAAAGCACCTGACTTTCTTGACTTTGGCTCTAGAAAGTCAACAGTGGAACAAATCATGGATGCACTCTCTGATGATAATGTCCATAAGATTGGAGTGTACGGGATGGGGGGTGTTGGCAAAACAATGCTAGTGAAAGAAATTGTAAGAAAAATTGAGGAGAGTAAGAAGTCTTTTGATAAGGTGGTAACATCCACGATTAGCCAAACACCAGATTTTAAAAGGATTCAAGGACAACTAGCTGACAAGATAGGTTTAAAATTCGAACAAGAAACAATAGAAGGAAGGGCTACTTTTCTACGAAGGTGGTTGAAGGCGGAGAGAAGTATCCTAGTCGTGTTGGATGATGTCTGGGAGTATATTGATTTGGAAACAATAGGAATTCCAAGTGTTGAAGATCATAAAGGAATATGCAAGATCTTGTTTACCTCTAGGAATAAACAATTGATCTCAAATGATATGGGCGCCAATAAAATTTTTGAGATAAAAGTTTTAGGAGAAGATGAGTCCTGGAATTTATTTAAGGCAATGGCGGGTGAAATTGTTGAAGCAACTGATTTGAAGCCTATAGCCATTCAAATTATGAGAGAATGTGCAGGTTTGCCTATTGCTATTACTACTGTTGCTAAGGCATTACTAAATAAACCTTCCGACATTTGGAATGATGCCTTAGATCAACTTAAAAGTGTTGATGTGGGTATGGCAAACATTGGAGAAATGGACAAGAAAGTGTATTTGTCACTAAAACTGAGTTACGATTACTTGGGATATGAAGAGGTGAAGTTACTATTCTTGTTATGTAGCATGTTTCCAGAAGATTTTAACATTGATGTGGAAAAGTTGCACGTATATGCTATGAGCATGGGTTTCTTACGTGGTGTTGATACTGTGGTAAAAGGACGACGAAGGATTAAAAAATTGGTTGACGATCTTATATCTTCTTCCTTGCTTCAACAATATTCTGAGTATGGGAACAATTATGTGAAAATACATGATATGGTTCGTGATGTAGCCATACTAATAGCATCTCAGAATGATCACATACGTACATTGAGCTATGTGAAAAGATCGAATGAAGAATGGAAAGAAGAGAAATTGTCGGGTAACCATACTGTAGTGTTCTTAATTATTCAAGAATTGGATTCACCTGATTTCTCAAAGTTAATGCTACCTAAAGTTCAATTGTTCGTGTTATTTGGACCATCACCATCTATATATAATAGACATGTTGTGTCAGTGGTAGAAACTTTCTATAAAGAAATGAAGGAGCTCGAAGGTTTGGTAATAGAAAGGGTGAAAATATCCTTATCGCCACAAGCTCTTTACTCATTTGCAAACCTTAGATTATTAAGATTACATGACTGTGAATTAGGGAGCATAGATATGATTGGTGAATTAAAAAAGCTTGAAATTCTTGATTTTAGTAAATCTAACATCGTTGAAATTCCTATGACCTTTAGCAAATTGACGCAGTTAAAAGTGTTAAATTTATCTTTTTGTGATGAGCTTGAGGTAATTCCACCCAATATTCTTTCAAAATTGACAAAACTGGAAGAATTACATCTAGAAACTTTCGATAGTTGGGAAGGGGAAGAATGGTACGAAGGAAGGAAAAATGCTAGTCTTTCTGAGCTAAGGTACTTGCCACACCTTTATGCTTTAAATTTAACCATTCAAGATGATGAGATTATGCCAAAACACTTGTTTTTAGCTGGGGAGTTGAATCTTGAAAATTTTCACATTACTATTGGTTGTCAGAGACAAAAAAGACATATTGATAATAAGACCAATTTCTTTAGAATCAAGATGGAATCAGAAAGGTGCTTGGATGATTGGATAAAAACTTTGTTAAAGAGGTCAGAAGAAGTCCATTTGAAAGGATCAATTTGTTCAAAGGTTCTCCACGATGCAAATGAATTCTTACATTTGAAGTATTTATACATTTCTGATAATTTAGAATTTCAACATTTTATCCATGAAAAGAACAATCCTTTGCGAAAATGCTTACCCAAATTGGAGTACCTATATCTGGAAGAGTTAGAGAATTTGAAGAATATAATTCATGGGTATCATAGAGAATCTCTTTTTAGCAAGTTGAAAAGTGTAGTCGTAACGAAGTGCAATAAATTAGAAAAGCTCTTTTTCAACTGCATATTGGATGACATTCTGAGTCTTGAGGAGATTGCTATTCATTATTGTGAGAAGATGGAAGTGATGATTGTGATGGAAAACGAGGAGGCAACCAACCACATTGAGTTTACTCATTTAAAGTATTTATTTCTAACGTATGTACCACAACTTCAAAAATTTTGCTCCAAAATTGAGAAATTTGGACAATTAAGTCAGGATAACTCAATCAGCAATACCGTTGACATTGGTGAATCGTTTTTCAATGAAGAGGTAAATTCCATCAACACACTTTTCTAT

GTCTCTATATAATACATGTAAAAAATTAAATTACCTTTAATTTCTGTTCTTTTTATTGTTCCTCCCACACAAGTTCTTCCTTCTAGCTTTTGTGGTTCAGTAATGTATCAGAGATCAGATTCTATTCTTCTTTGTTTAAGAAGGTTCTTCACTCAACAAAGAGATGACTTTCTTGTTTCATCCATTATTAATAAAAAGATTTTTTCCTATCCAAGGAAGCTCGATCGTTTGAAATAACCATTTTACACAATTTGTTATGTTAATTTTTAAGGACTATAAACATTATTATATGTTGTTAGCATCAGTTGTGATGATGGTCAATTACGTTATCTTAAACTGAGTTTTCAAAGTATGCAAAATTGTAG

GTATCACTTCCTAATTTGGAGAAATTGGGAATTAAGTGTGCAGAGAATTTGACGATGATATGGTGCAATAATGTACACTTTCCTAATTCCTTTTCCAAACTGGAGGAAGTAGAGATTGCTTCATGCAACAATCTTCACAAAGTATTATTTCCTTCAAATGTGATGAGCATTCTTACATGCCTTAAAGTCTTAAGGATTAATTGTTGTAAGCTGTTGGAAGGTATATTTGAAGTGCAAGAGTCAAGTATTACAGATACAAGTCTTATTGTGCTCAAAAACTTGAGAGAGTTGAAATTATATAATCTTCCCAACCTTGAGTACGTATGGAGCAAAAATCCTTGTGAGCTTCTGAGTTTTGTAAATATAAAAGGTTTGGCCATTGATGAATGTCCAAGACTTAGAAGAGAATATTCAGTCAAAATTCTCAAGCAACTTGAAAGACTAACAATGGATATTAAACAATTGATGGAGGTTATTGAGAATCAAAAGTCAACCGATCATAATATGGTGAAATCAAAGCAATTGGAGACTTCTTCTAAG

GTAATAATAATTTCCAACTACACAACCCATCATGTTTATTTGATATTTCACAGAATTTGTTAAATCACTTCGTTATTTCTTGTTTATTAGGTAGCTCCTCAAAATCCGAGGAAAAATCTTGTGAAGTAAGATAAAAAGTTTCAAGATATATTGTTCAATTATTTCAAAAGAAATTATTTCGAATGAAAAAAATTTAATTTACTAATATAAAAATATTGTGATAAAAACCATGTTATGTTTGATATATAGACCTTAATTTAACATATGAATAAATAAAATAAGTGTATGATTATTAACTTATTACAATCAATTCTAATAATTATTTAATTTTTGTCACTTCAAGTTAAATTAATTTTAATTTTAATTCAAATAAATATATTTTTATAGGCAATGTATCAAATATATATGATATGCATGTTAATCTCATTTGACTTGATAGTTTTTTAATTATATTATTGATAGTAAGTAATCAATTATAAATATTGAATTGTTTGCCAATTAATTAAGCTAATCTCTATTAACTAATTAAATATATTTTATTCAGAGACATTTTTAAAAATAGCAAAATAAATTAAAATATTTGCAACCTATAACAAAATTTTGGATTATAACAATAATAGTTTTCTATTACTATAACATATCAATGATTATTAGTGGTAGAATTTGTTATTTTTAAAAACTCCCCTTTTAGTTAAGGAGAATCGATATAGATAGTAAAAAACATTAAAAAAAAAAACTATTTACAAATTTTTATTAACTAATTAAATTTACGAAATTTCTTTAAAATAGCAAATTTTACAAAATATTTACAATCTAGCAAATTCTATCACGGATAGTCATTGATATGCTATAATGATAGAGTATTATCATTATGATTGATAGAATCCAAAATTTTGTAAATATTTTAACTTATTTTGCTATTTTAAAAAATGTTTTTTTAAATATATTTATTTTAATAAATATTTACTCTAAATAATTTCAATATTTTACTATTTTTAAAATGTTTCTATATAAAATATACTTTTAGCTAATTAATTAATAATTTTGTGCATGAGTTATTGCATTCATTTAATAAACTTTTATTTGTAAAATTGAATCGAGTGAATATTTGAATGATTTGGATGATCAAAATCTACAATTATATTAATTTTGGTGAATTAATTTGAAGAAGTTATTTAATATAATTGTTATTTGCTCGCTCAGTTCATGACATGAAATTGTTATCCTTTGCGGTGGACAATACTTAATAATTTTAGTTCCAAATATGTAATCTTTACAGGAAATCTAGAAAAAAATAAATACATTCTCAACTTTATTATTTAACATAATATATATTCGTCAG

GTTGAGGTTCTACTTACGGGAGATGGTTCTGAGTTGTTTCCTAATCTTAAAGAATTGACGTTATATGGTTTTGTTGAGGATAACTCAACTCATTTGCCAGTGGAAATTGTACAAATCTTATACCAACTTGAGCACTTTGAATTGGAAGGAGCGTATATTGAAGAAGTTTTCCCCAGCAATATATTGATTCCAATGAAAAAACAGTACTATGCAAGATCTAAGAATTCAGTGCGTAGTTGGTTTCTATCTAAACTACCCAAGCTTAGGCATTTGTGGAGTGAATGTTCACAAAAGAATGCTTTTCCAATTCTACAAGATTTGAATGTAATAAGAATTTCAGAATGTGGTGGGTTGAGTTCCTTAGTTTCGTCATCAGTATCCTTTACAAACTTGACAGTTCTTAAAGTGGATAAGTGTGATAGACTAACCTATTTGTTGAATCCTTTGGTGGCTACAACCCTTGTGCAACTTGAAGAATTGACTTTAAGAGAATGCAAAATGATGAGTAGTGTAATTGAGGGAGGATCAGCTGAAGAAGATGGAAATGAGGAGACAACCAACCAGATTGA

GTTTACTCATTTGAAGTCTTTATTTCTAAAGGATTTACCACGACTACAAAAGTTTTACTCTAAAATTGAGACATTTGGTCAATTAAGCCGTGATAATTCCGAAAACCCTGAAACAACCACAATTCACAATCGCATTGGTGATTCATTTTTCAGTGAACAGGTGAACTCATAAATTCCAACACACTTCTTTCTGTATATGTACATCTACACACATCCAATCTTTTTTTCTTTTTATTGTTCCTCTGAATGACTTTTCTTTTCTTTTCTTTTCAGAAAACATTCTTCTTCTTTTTTTTTCAATGCAGAAATTTAAAATAAAAATTTCTATACACTGCTAGAACTTACAATATAACTGATCATCTCTCTTCAAACAATGCTGATGATGTTGTTAATTCATTGTATTGATAATGATGGTTATGTCGCCCCAACTGAATTTTCATGTATATGTAAATTTGTAGGAATCACTTCCTAATTTGGAGACATTGAG

AATTGATGGTGCAGAGAATTTGAGGATGATATGGAGTAATAATGTACTCATTCCTAATTCCTTTTCCAAACTCGAGGAAGTAGAGATTTATTCATGCAACAATCTTCAGGACGTATTATTTCATCCAAATATTATAAACATGCTTACATGCCTTAATACATTAAGGATTAAAAATTGTGAATTATTGGAGGGGATATTCGAAGTGCAAGAGCCGATTAGTGTTACAAAAACAAAAACAAATGCTATCGTGCTACCAAATAATTTGATAGAGTTGGAATTATATAATCTTCCAAACCTTGAGTACCTATGGAGTAAGAATCCAAATTTTGAACGGCTCGTGACTTTTGAAAGTATAAGAAGTTTGTCCATTGAAAAATGTTCAAAACTCAAAGGAGAATATTTTTTGTCAATCAAAACTTTCAAGCAACTTGTAAGACTGAAAATGGGTATTAGGCAATTGACAGTGGCTCTTGGGAAGGAAGTTAAGTCAGCAGATCATAGTATGTTATTGGAACCAAAGCAATTGGAGACTTCTTCTTCTAAGGTAAGTATATATTCTACAACACATCATGTTTATTCAATATTTCAGAAAATAAATTGTTATCATGACCTTAATTAG

>Cucsa.189390

ATGGATATAATTTCTCCTGTCGTTGGACCAATTGTGGAGTACACTTTAAAGCCTATTGGTCGTCAATTGAGTTATCTATTCTTCATTCGCCAACATATTCAAAACCTTGAGAGTCAAGTTGAATTGTTGAAGAACACTAAAGAATCGGTGGTTAACAAGGTTAATGAAGCGATAAGAAATGCTGAAAAGATAGAATCCGGTGTTCAAAGTTGGTTGACTAAGGTGGATTCCATCATTGAAAGATCTGAAACGTTACTAAAGAATCTTTCTGAGCAAGGTGGATTGTGCTTGAATTTGGTCCAGAGACACCAATTAAGTAGGAAAGCTGTAAAGTTGGCTGAGGAGGTTGTTGTGATAAAAATTGAGGGGAATTTCGATAAAGTCTCCTCTCCTGTAGCTCTTTCAGAGGTTGAGAGTTCAAAGGCAAAGAATTCTGATTTTGTCGACTTTGAATCAAGAAAGCCAACTATTGACAAAATCATTGCTGCACTTATGGATGATAATGTCCACACAATTGGAGTGTACGGGATGGGAGGTGTTGGCAAAACAATGCTAGTCCAAGAGATTTCAAAATTAGCTATGGAGCAAAAGCTATTTGATGAAGTAATCACATCAACTGTTAGTCAAACGCCAGACTTAAGAAGAATTCAAGGACAACTTGGTGATAAGCTTGGACTCCGATTTGAACAAGAAACAGAAGAAGGAAGGGCTCTTAAGTTACTAAATAGGTTGAAGATGGAACGTCAAAAGATCCTCATTGTACTTGATGATGTTTGGAAGCAAATTGACTTGGAAAAAATAGGAATTCCAAGCATTGAAGATCACAGTGGATGCAAGATCCTATTTACCTCTAGAGATAATGATGTTCTCTTTAATGATTGGCGCACATATAAAAATTTTGAGATAAAATTTTTACAAGAGGACGAGACGTGGAATTTATTCAGGAAAATGGCTGGTGAGATTGTTGAAACATCTGATTTTAAGAGTATAGCTGTTGAAATAGTAAGGGAATGCGCACATTTGCCCATTGCTATTACTACAATCGCTAGGGCATTGAGAAATAAACCTGCATCCATTTGGAAAGATGCCTTAATCCAACTAAGAAATCCTGTCTTTGTGAATATTAGAGAAATAAATAAGAAAGTGTATTCTTCCCTAAAGTTAAGTTACGATTACTTAGATTCTGAAGAGGCCAAATCACTATTTTTGCTCTGTAGTATGTTCCCAGAAGATTATATCATTGATTGTCAGGTCTTGCATGTATACGCTATGGGCATGGGTTTATTGCATGGTGTTGAGAGTGTAGCACAAGCACGAAATAGGATAACGAAATTAGTTGATGATCTCATATCTTCTTCTTTGCTTTTAAAAGAATCAAATGTCGATTTGGTTATGTATGTTAAAATGCATGATATAGTTCGTGATGTGGCTATAATAATTGCATCTAAAGATGATCGTATTTTTACACTAAGCTATTCCAAAGGATTATTGGATGAATCATGGGATGAAAAGAAACTAGTAGGTAAGCATACTGCAGTGTGCTTAAATGTTAAAGGTTTGCATAACCTTCCCCAAAAGTTAATGCTACCCAAAGTTCAGTTATTGGTGTTTTGTGGAACTTTATTAGGTGAACATGAGTTGCCAGGAACATTTTTTGAAGAAATGAAAGGGATGCGAGTTTTGGAAATAAGAAGCATGAAAATGCCCTTATTGTCACCATCACTTTACTCTTTGACAAACCTTCAATCGTTGCATTTGTTTGATTGTGAATTGGAAAACATAGATGTGATTTGTGAGTTGAACAAACTTGAAAATCTCAGCCTAAAAGGATCACATATCATCCAGATCCCTGCAACTATAAGTCAATTGACACAACTAAAAGTATTAGACTTATCAGAATGTTATGCACTAAAGGTAATTCCGCCTAATATTCTTGTAAATTTGACAAAGTTGGAAGAATTATATTTGCTAAATTTTGATGGTTGGGAAAGCGAAGAATTGAACCAAGGAAGAAGAAATGCTAGTATATCTGAGCTTAGTTACCTTTCTCAGCTTTGTGCTTTAGCATTACATATTCCAAGTGAAAAAGTTATGCCAAAAGAGTTGTTTTCAAGGTTTTTTAATTTGGAAAAGTTTGAAATTTTTATTGGTCGCAAACCTGTTGGACTTCACAAAAGGAAATTCTCAAGAGTGTTGTGTTTGAAGATGGAAACAACAAATAGTATGGATAAAGGAATAAACATGTTGTTAAAGAGGTCAGAAAGATTACATTTAGTAGGATCAATTGGTGCAAGGGTTTTCCCGTTTGAGTTGAATGAAAACGAATCTTCATATTTGAAGTATCTCTACATCAACTATAATTCAAATTTTCAACATTTTATCCATGGACAGAACAAGACTAATTTGCAAAAAGTCTTGTCCAATATGGAGCGTCTGGAATTGAGCTATTTGGAGAATTTGGAGAGTTTTTTTCATGGTGATATTAAAGATATTTCTTTCAACAACTTGAAGGTCATAAAGTTGTTAAGTTGTAATAAATTAGGAAGTCTTTTTTTGGATTCCAACATGAATGGCATGTTATTGCATCTTGAGAGGATTAACATTACTGATTGTGAGAAGGTGAAAACAGTTATTTTAATGGAAAGTGGAAACCCATCTGACCCTGTTGAATTTACAAATTTGAAGCGTTTAAGGCTAAATGGGTTACCACAACTTCAAAGTTTTTACTCCAAAATTGAACAATTGAGTCCTGATCAAGAAGCAGAAAAAGATGAGAGAAGCAGAAATTTCAATGATGGTTTACTTTTTAATGAACAGGTTTGCTTTCTTAATATAAATTCTCCAATTGCTTTAACTTTTAATAGTGAATATGTCAGATCCCTCCCCAGCACTCTCCCTTGCCTGATAAAGGTGTGAAGCTAGCAAGTATCGTCTGTCACGATACTCTTACTAGCCAGCTTGACTTGAAACAAACTTCTTTTAACGTTGCACTAATTGATAATATAAAATAAAACAACTCTAAGCTCAACACCAAACTTTAAATCACACAGCGGAAAACATTTTTAGCACTCATGTACACTTTAAATCCTACTTGCTTGA

>Cucsa.128110

ATGGCGGATTCAGTTCTGTTCAATGTTGCTGCAAGTGTTATTACTAAACTGGGATCTTCCGCACTTCGAGAACTTGGGTCTCTGTGGGGAGTCAACGATGAGCTCGACAAACTCCAAAACACTCTTTCGGCCATTAAAGCCGTCCTTCTCGATGCAGAGGAGCAACAGTCCAAGAGCCACACAGTCAAGGATTGGATTGCAAAGATCAAAGATGTTTTCTATGACATTGATGACTTGATTGACGAGTTCTCTTATGAAACCTTGAGAAGACAAGTTCTTACCAAGGATAGAACAATCACCAAACAAGTACGTATCTTCTTCTCCAAATCTAATCAGATTGCTTTTGGTTTCAAAATGGGTCAAACAATTAAAAAAGTTAGGGAGAAGCTAGATGCTATTGCGGCTATTAAAGCTCAACTTCACCTCTCTGTGTGTGCGAGGGAGGTACGAGATAATGAGCCAAGGAAGGTACGAGAGACGTCCTCATTCATACCCGAGGGGGAAATCATTGGTAGGGATGAGGATAGGAAATCTGTTATGGATTTTCTATTGAATACCAGCAACATCACAAAGGATAACGTTGAAGTTGTTTCCATTGTTGGAATGGGAGGATTAGGAAAGACAGCACTCGCTCAAACTGTCTATAATGATGAAAAAATAAACAATCGTTTTAAGTGGAAAATATGGGTGTGTATTTCTCAAGAATTTGATATCAAAGTAATTGTTGAAAAGATTTTAGAGTCTATTACGAAAACAAAACAAGAATCCCTTCAGTTGGATATATTACAAAGTATGCTTCAAGAGAAAATTTATGGAAAAAAATACTTGTTGGTCATGGATGATGTGTGGAATGTAGACCACGAGAAATGGATTGGTCTGAAAAGATTTCTGATGGGTGGTGCCAGCGGAAGTAAGATTTTGGTGACAACCCGTAATCTACAAACTGCCCAGGCTTCTGACACGGTTTGGTTCCATCACTTAAAAGAACTTGACAAGGATAACTCTTGGGCGTTGTTTAGGAAAATGGCATTCTTAAACAAAGAAGAAGAGCTTGAGAATTCAAATTTGGTTAGAATCGGTAAAGAGATTGTAGCAAAGTTGAAAGGTTATCCCCTTTCAATAAGAGTTGTTGGACGTCTGTTATATTTCAAAAACACAGAAATGGATTGGTCATCATTTAAGGACAACGAACTTGACTCAATTTTGCAAGAAGATGATCAAATTCAACCAATACTGAAGATAAGTTTTAACCACCTTCCACCTAAATTGAAGCAATGTTTTACGTACTGTGCTTTGTTTCCCAAGGATTATGAGTTTAAAAAGAATGGATTGGTAAAACAATGGATGGCACAAGGTTTCATTCAAGCACATAATAAAAAGGCAATTGAAGATGTGGGTGACGATTATTTTCAAGAGTTAGTGGGGAGGTCATTCTTTCAAGACATAAGAAAAAACAAATGGGGAGACTTAAAGTACTGTAAGATGCATGATTTGTTACATGATCTTGCGTGTTCGATAGGAGAAAATGAATGTGTGGTTGTAAGTGATGATGTCGGGTCCATTGACAAAAGGACTCGACATGCCTCATTTCTCTTGAGCAAGAGGCTAACAAGGGAAGTTGTATCAAAATCATCCATTGAGGTAACGAGTTTGAGAACATTGGATATTGATAGTCGTGCTTCTTTCCGTTCTTTCAAGAAAACTTGTCACATGAACCTTTTTCAATTACGAACATTGAATTTGGATAGATGCTGCTGTCATCCTCCTAAGTTTGTTGATAAGTTGAAACATTTGAGATATCTTAATCTTTCTGGTTTAAATGTAACTTTCCTTCCCAATTCTATTACCACATTGTATAATTTGGAAACACTTATCCTTCGTTACTGCCTTTGGCTAAGAAAATTGCCAAAAGATATTAACAATTTGATCAATCTCAGGCATCTTGATATTTATGATTGTTCCAGTTTGACTCACATGCCAAAAGGATTAGGTGGGATGACTAGCCTTCAGACAATGAGTATGTTTGTATTAGGAAAGAATAAAGGTGGTGATTTAAGTGCATTGAATGGACTTAAAAGCTTGAGAGGATTATTATGTATTAAAGGTTTACAATTTTGCACAACTGCTGATTTAAAAAATGTAAGCTACTTAAAAGAAATGTATGGAATTCAAAAGCTGGAATTACACTGGGATATAAAAATGGATCATGAAGATGCCTTAGATGATGGTGATAATGATGATGAGGGAGTTTTGGAGGGCTTAAAACCACATTCAAATATTCGCAAAATGATTATAAAAGGATACAGAGGAATGAAGTTATGTGATTGGTTTTCTTCTAATTTCCTGGGTGGTTTGGTTAGCATAGAGCTTTCACATTGTGAAAAATTGGAGCATCTCCCACAGTTTGATCAATTCCTATATCTCAAGCATCTTCTTCTTGGATACTTACCCAATATTGAATACATTGATAGCGGCAATTCTGTTTCTTCATCAACAACATTTTTTCCATCTCTCGAGAAGCTAAGGATTGAGAGCATGCCTAAGTTGAAAGGGTGGTGGAAGGGGGAAATTTCATTTCCAACAACAATATTACATCAACTCTCAGAATTATGTATTTTTTATTGTCCTCTGTTGGCTTCTATTCCACAACATCCATCTTTGGAATCATTGAGAATATGTGGTGTTAGTGTGCAACTTTTTCAAATGGTAATACGAATGGCTACAGACCTTTCTGAACATTCTTCTTCTTCTTCAACATTGTCTAAATTATCTTTCCTTGAGATTGGAACTATTGATCTTGAGTTCTTGCCAGTGGAGTTATTCTGCAATATGACACATCTTGAGTCTCTTATCATAGAACGCTGCAAAAGTTTACAAATGTCTTCTCCGCATCCTGTTGATGAGGATAATGATGTGGTATGGAAAAAACTCAGCAATCTCCGGACACTTCGGCTTGAGAGCATCCTCAAATTGGAGTATTTTCCCAAGAGTTTGAAATATATTACAAGTCTTGAAACTTTGAAGCTATCAAATTGTGAAAATTTAGTGAGTACGGAAGGGATTGGCGAACTCATTTCACTATCACATTTGGAAATTGATAGATGTCCTAATTTACCTATATTGTCGGAAGATGTCGGCGACCTCATTTCCCTATCACACTTGCTTATTTGGAATTGTCCCAAATTAACTTCCTTGTCAGAAGGAATCACTCGCCTCACTTCACTCTCAAGTTTGTGTCTTGAAGATTGTCCCAACTTAGTCTCCTTGCCCCAAGAATTTCTCCACCACCACAGCTCCTTACCAGGAGGACGGTTCTTGAGAATTTTGAACTGTCCCAAATTGCAGATTCAAGACAAGAAACAAAAGGAAGAAGAAGAAGAAGACCAGGAGGATTGGAATGAACTCATCCATGTATTGACCGGATGTAGGTAA

Additional *R* genes in cucumber Gy14

>Cucsa.017450

TCAACATACATCTATCCTACCCATTGTTGGTATGGGTGGATTAGGAAAAACAACTTTGGCAAAGTTAGTTTTCAACCATGAGTTGGTTAGACAACATTTTGATAAAACTGTATGGGTTTGTGTCTCTGAACCATTTATTGTCAACAAGATTTTGCTAAATATTTTACAAAATCTAAAAGGCACCATTTTTAATCTAATGGAGGGGATAGTAAGGAAGTTTTATTCGTGAACTCCAAAAAGAGATGCTTGGGCAACGGTATTTTCTTGTGCTTGACGATGTTTGGAACGAAAATTCTTTTCTATGGAATGAGTTGAAATGTTGTTTACTCAAGATCACTGAAAACTCTAAGAATAGTATTGTTGTGACTACAAGAAGTGCTGAAGTTGCAAAAATCATGGAAACATGTCATAGTTATTTTTTAAGTAAATTATCTAATGATCATTGTTGGTCCTTATTTAAAGAAAGTGCAAATGCATATGGATTATCCAATGACTTCAAACTTGGGGATCGTTCAAAAAGAGTTAGTCAAAAAAATTGGTGGCGTACCATTAGTTGCACGAGTTTTAGGAAGGGCAGTAAAATTTGAAGGAGATGTTGAGAGATGGAAGGAAATGTTGAAAAGTGTGTTAAAAACTCCAATACAAGAGGAAAATTTTGTTTTGTCTCATTAAAATTAAGTGTAGATCGTCTGCCATCATAAAGCAATGTTTTTCTTATTGTTCAATTTTTCCGAAGGATTTTGTGTTTGAAAAACAAGGACTAGTTAAAATATGGATGGCACAAGGTTTTCTTCAACCACAAGAAGGAAGAAATGTGACGATGGAAAATGTAGGAGACATGTACTTCAAGATCTTGTTGTCACAGTGCTTATTTCAAGTTGCCAATGAAATAAAGATGGGAGAATATTATAAGATGCATGATCTTGTACATGATATTGCAATAGCAATTTCTAGGGATCAAAATTTGCGACTAAATCCTAGCAATATATTGGAGAAGGAACTTCAGAAGAAAGAGATTCAAAACGTTGCATGCAAGCTACGCACGATTGATTTCATTCAAAAGATTCCTCACAATATAGATCATACACTTTTTGATCACTTTAAGATAAGGAATTTTGTTTGTTTGCGTATTTTGAAGATTTCTAGTGAGAAGTTATCCAAGTCAATTGGTCAATTGAAACACTTGAGATTTCAAGTAATTCAATGAGATTAAAATTTCCAGAATCTATTGTTTCGCTTCATAATTTGCAAACATTGAAGTTCCTACACTCAAAGATTGAAGAATTTCCGATGAACTTTACAAATTTGGCAAGTTTAAGGCACTTGGAATTATGGGTAAGTTCTGACAAAACGCCTCTACATTTAAGTCGTTTGACTCAACTTCAAATATTGTCTCATTTTGTAGTCGGGTTTGAAAAAGGTTGTAAGATTACTGAATTGGGTCGATTGAAAAACTTGCAAGGTAGTTTGAGTCTTTTGTGTTCGGAGAAAGTTGAAAGTAAAGAGGAAGCCAATGGAGCAAACTTGGCAGAAAAGGAGAATTTAAAAGAGCTGCACTTGAATTGGGATATGGAAAGAAAAGATAACAACAGTTACAATGATTTGGAGGTGTTGGAAGGACTTCAACCAAACCAAAATCTCCAATCATTAATAATCCACAGCTTTGCAGAAAGACGTTTGCCTAACAAGATTTTTGTTGAGAATTTAAGAGTGATACATTTGTATTCATCTTTTAATTGTGTAAAGCTTCCAATGCTTGGACAATTAAACAACCTAAAGGAACTTGAGATTTACAGCTTCCTTGGTGTCCGAATTATAGACAACGAGTTCTATGGTAATGATCCAAACCAAAGAAGGTTCTTCCCAAAGCTTGAGAAATTTGTAATGTATGAGATGATCAACTTAGAGCAATGGAAAGAAGTAATGGCAAATGATTAACATCAAATATTACAAGTTTTCCCAATCTTAAATGCTCGGAGATAAGTAGATGTCCTAAATTATTAAACATTCCAAAAGTTTTCTATGAAAATGGTATGCAACACTTTCAGTCATTGTCCGTTTCACACTGTAATAAATTGACAAAACTTCCAAATGGACTACACTTTTGTAGTTCCATTCGACATGTGAGAATAGACCAATGTTCAAATTTGAGCATAGATATGAGAAATAAGTCGAAATTGTGTTATTTAAACATTGGTCCGTTGAGGAAGCTGCCAAAAGATTTATGTCATCTCATGAATTTGAGGGAAATGGAAATCGTTGGAAATATGCATAATTATGATTTTGGCATCCTTCAGCACCTTCCTTCCCTTAAACAAATTACTTTAGTCGAGGATGAGTTGAGCAACAATAGTGTAACGCAAATTCCTCAACAACTCCAACACCTCACTGCCTTGGAATTTCTGTCCATTAAAAATTTTGGAGGCATTGAAGCTTTGCCAGAATGGTTAGGAAACTTTGTATGTTTGCAAACACTCGGTCTTTCTAATTGCAAAAAATTGAAAAAATTGCCTTCTACAGGAGCAATTCTACGTCTCACCAAATTAAAAAAATTGTATGCTTATAAATGTCCACAGCTACTACTTGACGAAGGTGAGATGA

>Cucsa.041670

ATGGATTCTGATGGGGTTGAATCAGAATCTACTCCGGCTATTTCCACCTGTCTCACCATTAAAATAGCTCCAACTTCCAGTAAACCGCCGGGGACGTCTTCCGATTTGGCATTGCCGGAGCTCAAAAGCTCAATCGAATCATCTCCTTATAACTCCCCTTCCCTTTTATCGCCGCCGTCATCGGCGTTTGTTTCGGCTTTGCAGTCGCCGTACATTTCGCCGAGGGCGGTGGTCCTTAAACCAGAAGAGAAGCCTATTCCGGCGGAGAGCACGGCGGCGCTGACTTGTCATTCACCGCTGGTTTCCCAATCTGAGGATATTCCGAGTAGTTCCTACACTCCTCCCTCGGACCAATATGAATATTCCGATGACCCTTCTGATTCTAAGGTTCAGTTTGTCGCCTGTGTTCCGGTCCCCGACTCCGCCCCGCCTCGCATTTCGTTCTCCTTCCCCGTTCCTCGGACCTCCTTCGCCAAATGCGGCGGCCCCCTCTCTCCTGTTTCCACCTCTAAACTCAGAAGCTGTGATGTCTACATTGGCTTTCATGGCCAAGCTAATGGCTTGATACGCTTCTGTAAGTGGCTCAAATCAGAACTTGAACTTCAAGGAATTGCCTGCTTCATTGCTGACCGGTCCAAGTACTCCGATAACCAAAGCCACGAGATCGCTGACCGCGTTATCAGCTCAGTAACTTTCGGAGTCGTCGTCCTCACAAGCTCTAGTTTCCATAACCATTTCACCTTGGAGGAGGTGAGATTCTTTGCACAGAAGAAGAACTTGATTCCATTCTTTTTCGACATGGAGTCGTCGGAGATCTCAAGCTTTCTCAACTATAATTCCATGGATAAAGAGTACAAAGAGACAGTGCAGGGATTGTTGAGGTTCCATGAATACAAGTTGGAAGCCAATGAAGGTAATTGGAGAAGCTGTATAGCTAAAGCGGCTGGGATTTTGAGGGGAAAGCTTGGGAGGATGAGTACTGAAAGTGATGTTGAAAGATATGAGGAGCTGCCATTTCCAAGAAACAGATGCTTTTTAGGGAGGGAAAAGGAGATTATGGAAATGGAAGCTACTCTGTTTGGTAATCGAAGCTATCACAAGCAAGACGGTACGGTTTCTACTCTGATCGTTGAAGGGAATTCCAGCCAGCAATCTGAAGGCTTAGCAGATGAAGAAAGTGAGCCCGTTAGTGTGAGGGGAAGTAGGTTCATCAATTTGGAGATAGGGAGGTCTGATAATCCAACTTTGGAGACATGGATTGAACCAGTTAAAGGAAGAAATTCATTCAAGAGATCGAAGCACAAAGAAATGGTCAAGAGTGGGAATCACAAGAGCATGAGCAGCAGCATAGTGTGCATCAATGGAAACCCTGGAATTGGAAAGACAGAACTTGCTCTGGAATTTGCTTATAGATACTCCCAAAGATACAAGATGGTTTTATGGGTCGGTGGTGAAGCTCGGTACTTTCGACAGAATATATTGAACTTATCTTTGAACTTAGGGTTAGACATAAGTGCTGATGCTGAAAAGGATAGAGGACGGTTTCGGAGTTTCGAGGAGCAAGAACAAGAGGCATTCAAAAGAGTCAAGAGGGAGCTGTTTGGAGACATGCCATATTTGTTAATCATTGATAATCTTGAGGCAGAAGAGGACTGGTGGGAAGGGAAAGATTTGAACGATTTGTTGCCGAGGAACACCGGAGGATCCCATGTGATCATCACTACGAGACTCTCTAAGGTGATGAGTTTTCGGATGATTAACATTCATCCATTGGCTTTGGCTGATGCAATGGTTTTGATGAGAGGAAGAAGGAAGAAAGAATACCCAGCAGATGAATTGGAATATCTGAAGAAGTTCGATGAGAGGCTTGGGAGATTGACATATGGACTTTGGGTGATTGGATCGCTGCTTTGCGAGCTTGCAATCACCCCGTCTTCTCTTTTTGAAGCTATTGAACAAGTACCCATCGATGAATGTTCTCCCTGCCCTTATATAAGCATAAACGAAGAGCACTACTGCAAAAGCAATCCCTTCCTGATGAAGATCATTTACTTTTCTTTTTCCATATTGGAGCAAACTAATGGACCGTTAGCATCCGGAATATTTCTGGTCGGTGCCTGGCTCGCCCCAGCACCCATCTCAGTGTCTGTACTAGCCACAGCAGCAAAGGATATGGCTGTCTCAAGAAAAGGGTTTAAAATTTGGAGTAAATACTTGAGTTTCATGTTCGGTTGTTGCTCCACTTGTTTAGCTTCACAAGCTTGGAAGAGTGAGGAAGAATCAGCTCTTCTTCTGATCAAGTTCGGGCTCGCCCGGAAGGCGAACAAGCAAACTGGTAGTTGGATCCAATTCCATCCCATAACTCAAGTGTTTGCCAAAAGAAAGGAGGGTTTATCAGCTGCCAAGTCTATAGTTCAAGGGATAAGGAAATGTAGTAGTAACACAATGGCAAACTTGGATCATTTATGGGCATCTGCATTTCTCGTTTTTGGTTTCAAATCTGAACCTCCATTTGTACAACTAAAGGCTGTTGATATGGTCCTATACATAAAAAAGGCTGCCCTTCCTTTGGCGATTCGGGCCTTCACAACCTTTTCGAGATGTAACTCAGCACTAGAATTGCTAAAGGTATGCACAAATGCACTTGAGGAAGTAGAGAAGTCATTTGTATCACAAATACAAGACTGGTGTGAAGGGTCCTTGTGTTGGAAGAAAAAGTTCCAAGGCTATCAACGAGTTGATGAATACGTGTGGCAAGATGTGACGTTGCTAAAAGCTACATTGCTTGAAACTCGAGCGAAACTACTACTAAGAGGTGGACACTTCGACAGCGCCGAAGAACTATGTAGAACTTGCATAAGTATAAGAACAGTCATGTTGGGACATAACCATGCTCAAACCTTGGCAGCACAAGAAACGTTAGCGAAGATTGTTCGGCTTCGGAGCAAGATCTGA

>Cucsa.091460

ATGGGTTCTTCTGTTGTTGGAGATGAATCATTTTCTTCTTCTCCCAATTTCAATTACGATTATGATGTGTTTTTTAGTTTCAGAGGAGAAGATACTCGCTCCAATTTTATCAGTCATCTTCATATGGCCTTGCGTCTAAAGGAAGTCAACGTTTTCATAGACGACAAACTCAAAAGGGGTGAACAAATTTATGAGTCTCTTCTCAAATTTATAGAGCGATCTAGACTTTCCCTCGTTATTTTCTCTAAAGATTATGCATCTTCAACTTGGTGTTTGGATGAACTGGTGAAAATAATTGAGTGTAAGAAATCCAAAGGACAAGCAGTTTGGCCAGTGTTCTACAAGGTGGATCCATCCGAGGTTCGAAAACAAACCGGTGGGTTTGGGGAAGCATTGGCCAAACATGAAGCTAATAAGTTATTGGCCAACAAGATTCAACCATGGAGGGAAGCTTTGACTTTTGCTGCTGGTTTGTCTGGTTGGGATCTAGCAAATAG

GTATTTCTTTTTTCTTTTTTTTGTTTTAATCTTTGAAGACTCATTGTCCAAGTGAAGTTTAAATTTATCTACTATTTCCAAATTCTGATTCTTTTTGTATGATGTTACTTTACAACAG

CAAGGATGAGGCTGAACTTATCCAAAAAATTGTTAAACGAGTGTTGTCTGCAGTAAATCCAATGCAATTACTACATGTAGCCAAGCACCAAGTAGGAGTTGATTCTCGACTAAGGAAAATTGAGGAGTTGGTCTCTCATATTGGGTCCGAGGGTGTTAATCTGGTGGGGTTGTATGGCATTGGAGGCATTGGTAAGACCACTTTGGCTAAGGCTTTGTACAACAAAATTGCTACCCAATTTGAAGGATGCTGCTTTCTACAAGATGTTAGACGAGAAGCTTCGAAGCATGGGCTCGTTCAACTACAGGAAACCTTACTCAATGAGATCTTAAAAGAGGATTTGAAGGTTATTGTCAGTCGTGATAGAGGAATTAACATCATAAGAAGTAGACTGTGTTCAAAGAAAGTTCTTATAGTTCTTGATGATGTGAATGATCTTGAGCAATTAGAAGCACTGGTTGGTGGGCGTGATTGGTTTGGTCAAGGTAGTAAAATCATTGTGACGACAAGGAATGAACATTTACTTTCTAGCCATGGATTTGATGAAAAGCATAAAATTCAAGAATTGAATCAAGACCATGCTCTTGAGCTTTTCAGTTGGCATGCTTTTAAGAAAAGTCATCCATCAAGTAATTATTTAGATTTTTCAAAGCGTGCTACAAGTTATTGTAAAGGTCTCTCTTTGGCCCTCGTTGTTTTGGGTTCTTTCCTTTGCGGCAGAGCTAAAGAAGAATGGAATGGTATACTAGATGAATTTGAAAACTCTTTGAGAAAAGATATTAAAGATGTACTTCAATTAAGTTTTGATGGACTTGAAGACAAAATAAAGGATATTTTCCTCGACATTTCTTGTTTATTCGTGGGAGAAGAATACAAGTGTGCTAAAAAAATGTTGAGTGCATGTCATTTGAACATAGATTTTGGAATTATGATACTCATGGATCTTTCACTTATTACGGTTGAAATGGATAGAGTGCAAATGCATGAGTTAATACAACAAATGGGTCGTAGCATAGTTCATAATGAATCATCTGAGCCTGGAAAGAGGAGTAGGCTGTGGTTGGAACACGACATTTGGGAG

GTGTTTGTTAATAATTCTGTGAGTAACTCTCTTACCTAAAGTATTGATAATTTACTTATTTTCAAGGCTTCACATGATAAGAAATGTTTGTCAAAAGTAAGTTGTAAGCAGTGTTTACTAAATACATGACTTTATGCAG

GGAACAGATGCAGTTAAAGCCATAAAGTTGGACTTGCCTAAATCCACAAGGCTAAATGTAGATCCACGGGCATTTGGAAGCATGAAAAATTTGAGATTGCTTATCATTCGAAATGCACGATTTTGTACAAAGATTAGGTACTTACCTAATAGCTTAAAGTGGATTGAATGGCATGGATTTGCTCATCGAACTTTGCCGTCCTGCTTCATTACCAAAAATCTTGTTGGACTTGATTTGCAACATAGCCTCATCAAAAGATTTGGGAAAAGACTTAAG

GTATGAAAATGAGGTTTAGTTTCTTCATTGTTTTTCTTAAAAACTTTTAATAGGCAGCTTAATTACATTTTTAAAAAAATCACAAATACATGGATTAGCAAAAAATTATCATCGATTAACTCTTGCTAGTCATATATTTTTTAAGAATTACAAAATATATCTATTAACGATAGATTCATCTATCGCACAACAATAGCTCCTACTAGTCATATAATCTATCATTAATAAACTCATATAAGTGATATGATCTACCATGGATAAACTCTAAGAACATCCGCTAATTTTTTTCTAAATTTGTAATTTTTTGTGTTATCCTTTTGAGTATGATAACCATCATGATGCAAAATAATGTATTGGGTAGATTCTTTTTATGTTATGTAACTTACTTGAACTAGATTTGTTGCATACAAGATTGTCCAATTATAATGTGTCCTTAAGTTCCAAAATTTTGATTTTGTTACGTTTTTATCTTTTTTTTAAAAAAAATTAAATTTGATTTAAGATCAAATCAAACTAAAATTTGAAACCAAATTTCCACTATAAATTGACTAAATTACAATTTTGTTGGTAATTAGTTTACAATCTGCTAAATTCAAAATAATATATTTGATGTAAAATTACCTTTTTAACATCTGAGTTTTCATGAATACGTGCCATTGGTCCATAAGTTTTAAAAATAGACATTTTTAATTTCAGATTTATAATAATAGAATAATTTGATCCTAAGTTTTTAAAAATAGGTTTAAAAGACCTTCAAATAATTTTATGCTTTTATTTTAAATAGTTATATGACATTGTAAATTAGAATAATATATGGCTTCTAAAATTCATATCCTCTCTAAAACTTTTTTTTTCTAGTTTTAAATATCATATAATTATTTTTAAAAAACCATAAAGTACTTTTAGGACGTTATAAACCTATTATTTCTTAAAACTCAAGTGAGTAAGAAGATACATTTTTAAAATTTAAGGACCAAATATGATTATTTTTATAAACTTGGATACTAAAATTCATATCATCTCTAAAACTAACAGTTGTAAAAGTGTGTCGTGGCTAACATGAACGTATAGCTCAACTGACATAGTTTTTGTACTATCAATTTTGATATTGGAGGTTTGATTCTCTCATGCTTTAATTACAATACCTTTTAAAAAGAAAGAAAAGTGTTGTTATTTTATGGGGCATTTGTAAAAATAGCAAAAACTTTTATGATAATGGGGTTTATGTCACTATATTTTCTAAATTACAAAAGTAACAAATTTAAAAGCAAATAGTCCTCTGATAGCTGTATGATATATTTAATTAATTGTCATATTTGTCAAAAAGAGGTGCTATGAAATGCTTTTTTTCTAATTTTTTTTGTCATCTAATGCAATTTTCCTTATTATATATGCAATATAATAGTTGTGGATTAATGCAAATAGATGATTAATCTTGATATGCAAATCGATTTACTTTTGTATATGGAGTTTTATTAGTTTGCCTAAATATTAATTAATGGTTATTATAG

ATTTTTTTTAAGGAAAATTTACATAAGCAGAAGAAAGTGGAAAGTATTTACATCCTATAGACAAAAAATGGATATTTTAGACTTGATTAAAATTTTGGTTATTCATTTTGAGCTTGGATTAAAATTTTGGACTGTAAATAGTTTGGTAATTCTTCTATTTTAAAAATTTTCCTTTTTTTAACATATATTCTATATATTTTTCTATATTTACAATTATTGTTTTAGCCAATATTTTATCTAAATTTTGTTTGGTATGCCAACTACATACCCATTTTCCTTTTGGTTTATTTTCCAATGTGTTTTAGGATTGTGAAAGGTTGAAGTATGTTGATCTTAGCTACTCTACTTTATTAGAGCAAATTCCTGATTTCACTGCAGCATCAAACCTTGAAGAGTTGTATCTCATCAATTGCACAAATTTAGGAATGATAGATAAGTCTATTTTCTCTCTCAATAAGCTTGTTGTCCTAAACTTTGATGGTTGTTCTAACCTTAAAAAGCTTCCAAGTGGCTACTTCATGTTAAGTTCTCTTAAAGAATTGAATCTCTCTTACTGCAAAAAACTTGAGAAAATTCCAGACTTATCTTCAGCATCAAACCTTACGAGCTTGCATCTCTACGAATGCACAAATTTAAGAGTGATTCATGAATCTGTTGGATCTTTGGATAAGCTTGACCATTTGAACCTTAGACAATGCACGAACCTGGTAAAGCTTCCAAGCTATCTTAGGTTAAAGTCTCTTCGATATTTATCACTTTCTGGGTGTTGTAAGCTTGAAAGCTTCCCAACAATTGCTGAAAGCATGAAATCTTTAAGGTCCTTGGATTTGGATTTTACTGCCATAAAGGAGTTACCTTCATCAATGGGATATCTTACCAATCTATCTCGATTAAACCTTGACAGTTGCACAGGCCTCATCTCCCTTCCCAATACAATTTCTTTGTTAATGTCCCTATTGAAACTTGACTTAAGAAATTGCAGGTCTCTTCAAGAAATTCCGAACCTTCCTCAAAACATACAAATTTTGAATGCGAATGGCTGTAAATTGGTGGGTAAAAGTCCAGATAACATTGGGGATATAATATCAAAAAAACAG

GTTCTGTTTCTTTCCATTCAATTTGTTATGATATTTTGTAAAAAGTTTTATGCATATCAAAATTCTTCAATTATTTTGTAG

GACCTCACATTGGGTGAGATTTCAAGAGAGTTCTTACTAACAGGCATAAGACTACATCAAATTTAGTGAGTGCTAGCTTTCGTCACTATCCAGACATGGAAAGAACTTTGGCTGCCTGTGTTAGTTTCACAGTGAATGGAGATTCATCTGAAAGAATTTCATGCAATATATTCATCTGCAATAGACTGCATTGTTCTTTTTCAAGATCATTTCTTCCATCAAAATCAGAATATGTGTGGTTAGTGTCAACTTCTCTAGCTTGGGGCTCCATGGAGGTGAATGATTGGAAAGAAGTTTTGGTTTGGTTTGAGGTTCATGATGAGGTAAATACAAGTATAAGAAGATGTGGTGTCCATGTCACTGAAGAGCTCCATGGGATAATACAAATGGATGTCAAGTGGCCGGTGGTAAATTATGCTGATTTTTATCAACTGGAGAAATTGCAAAATCT

GTAAGTTGATTGTTTACTCTTTTTTTATTTTGAGTGAAAGTAGGATTAGGTTAATATATGGACGGATATGATAATATGTTGTTTTAACTTAATGGCACACAG

GGATATTGAGGATCTTCTTGTCAAACGCTTTTTTGAAGAAATGTCCTCCTTGTCAAATTGCAAAGCAATGTTGCATGCAGGAAGTTATGCTCCAGAAGCAATAATTGGTTCCAACGTACAACCTATGGTTTTCCCGTTGCACGTATCATATAATGGTGATACAGTGATATGTGGAATGGAAGGCATGGCAAAAACTGCACTAGCCAACTCTGTATGCAACAAATTTAATTGGTCATATGATAATGATTGGAGACAACCTTTAGATAATCCTACAAGCTTTTACTTGGTCGAAGAATCACAGTACCGTTTCATGAGATATTCAGGCCTCGACAAGCGTGGAGGTTGTAAAAAAGGGACCAACATTATCGCAAACCATAGTACAATTACATCATCCAAAATGTATTACATATACTTTGAAAATTTGGATGATAGAGTATACAGATTTGTAA

>Cucsa.091530

AGCTATGATGTGTTTTTGAGTTTCAGAGGAGAGGATACTCGAGACAATTTCACCAGTCATCTTGACATGGCCTTGCGTCAAAAGGGTGTCAACGTCTTCATTGACGACCAGCTCGAAAGGGGTGAGCAAATTTCTGAAACCCTTTTCAAATCTATACACAAAACTTCCATTTCTATTGTTATATTCTCTGAAAATTATGCATCTTCGACATGGTGTCTGGATGAATTGGTGGAAATAATTGAATGTAAGAAATCCAAGGGTCAGGAAGTTTTGCCGATTTTCTACAAGGTGGATCCTTCGGATGTACGAAAACAAACTGGTTGGTTTGGAGGAGCATTGGCCAAACATGAGGCTAATTTCATGGAGAAGATTCCAATATGGAGGGATGCTTTAACTACTGCTGCCAACTTAGCTGGTTGGGATCTCGGAACCATAAGGTACAT

GTTTTTTACTGTTATTAAATGATTTTTTTAAAAAAAACAAAATAGTATTCTTTCAATAATTAATGACAAAAATAGTGTGGGAGCAAAAATATGAGAAAAATAGGTAGTGAAATAAATGGTAGATGTGGTTTACAACCTTATGAATGAAATCATCGTGAAAGGAACTTCACTCTTTTTGTTTCAACCTCTCAACTGCCCCAGGGTGCAGGAACTGGTTTTTGTATCTCTTTTTGCTTTGTCTTTTGGTTTTACTTTATACTTTTTGTATGCACTATTTGTGTTTATACTTTCCTTTGTATATATATGTGGAGGGGCGTTGGATTGGGGATTCTACTTTTTTTTCCATCATTTTCTACAAATGTAAACAAGTTGATTAAATATGTGGTCTATGCATTTCGAAGATTTAGTGATCATTTTTTTTAATAAGGGAATGGTGGGGATCGTGATATTGAGGTTTGCAAGACTTTTTTAGCTTTATTTTAAAAATATGTAGTTGATTAATAATTCTCTCTCTTTGTTGTACGTGTGTTCTTCTTAGTGTTTTATTAAATCTAAATAATTTTTTTAAAAGAGTGAGAACTCGTGCAAAGGTGTATGAGTTAGATAAAAGTCATGAATTAGGTCCATGAGTTGGGTTGTTTTTGGTGAAGATATCGATGTAGACATGAAAAAGAGATTTGAATTATTATTTGTTTTCTTATATTTTTATCATTTTTTTATATTTTCACTTTATTTAAGGACCCTATTGTTTGTCATTTATTTGGCCAAGTGCATTATTTAATTTGTGGAATTAACACTGTTTTTTCATATTCATTAGAAATTCTCACAATGTATAATTTAATCTCAGAAGATTTCTCTCATCATCTATTAATTTTGCAATGATTGTGTACTAATTAACAACAG

AAAGGAGGCTGATCTTATTCAAGTTATTGTTGAACGAGTGTTGTCTATATTAAATCAAACCCACACGCCCTTAAAGGTAGCTGAGTATCCAGTTGGAATTGATTACAAAATAGAATCCCTTTACTGGACACAAGAAATGTACAAGTCTGAATGTGTTGACATGGTGGGGATATATGGCATTCGAGGCATTGGTAAAACAACTTTGGCTAAAGCTTTATACAACAAAATTGCTAGCCAATTTGAAGGCTGCTGCTTTCTATCAAATGTTAGGGAAGCTTCAAAGCAATTCAATGGCCTTGCTCAATTACAGAAAAAGCTCCTTTTTCAAATCCTAAAGTATGATTTGGAGGTTGTCGATCTTGACAGGGGACATAATATCAAGCAAGCAGGTTCATCTCTTACCAATTCA

>Cucsa.128020

ATGGCTGGAAGAGAATCTTGGTGTTTTTCAAACGAAGAGGAGGTGATCGGGAGGAATGATGATAAGAAAAAGGTAAAGCACCTTTTATTGGATGATGTATGGAATGAAAGTGAAGAGAAATGGCATGGGTTGAAACCTTTGTTAATGAGTGGTGCGAAAGGGAGTAAGATTTTCATCACAATGCGTGATAGTAAAATAGCTGCAGAAATTGAAAGCATGACTTCTTTATTCACTTTAGAAGGCTTACCGAAGAGTAAATCTTGGTCATTATTTAGTAAAGTGGCATCCAAAGAAGGCAAGGTGCTTGAAAATTCAAACTTGTTACAGTTAGGAAAAGAAATTTCAGTGAAATGTGGAGGTGTTCCCTT

GTAACAAGATACGTTGGCCGCTTGCTATACTCTAAAACTTCAGAAGAAGAGTGGATATTTTTCAAGGATAACGAACTTTTAGAAATCATTAAGCAAGACAATTATATGACATAAGTATTAAAACTGAGCTATAACCTAAGAAGCAGTGACACGCCAGTTGGAATGTCATTATTATTATTCTTTTATTTTAATTTCGCACACAGCCCCGACATGGCTGGAAAAAAAAAACACAAAGGTTAGAAATGCCCATTAGCTCGGGCCAGCAATTATTAGGTTCTTTTTTCTTTAATTTTATCTTTTCAAATCATTTTTCACACATTTCTCCCATCTGCGGATTTTTCACAAATTCTCGCTGCCAACCCTCTCTGCCCTAACTTGTTGGGTCTTATGGCTCTAAACTTATATTTTGATGTTTTAATTGGAATATTTTACTATGTTTAGAGCATGACATATATTTAATTTTTTTTACTAAAAATTTGACGTGGTTGTGTCCTACTTTTTTTTTAAAATGATGTGTCACATATCACATGTGGTGCGTCTGTATCTGTGCTTCCTAGACTATAACCATCTCACACCAAGTTTGAAACAATGTTTTGCCTATTTATCCTTGTTACCCAAAAGGTAGAGCTTAAATATAAGAGATTTGATTAGACAATGGATGGCTCTAGGTTTAATAGAATCATCAAATGGAAGTAAATCCATGGAAGATACAGGAAAGGACTATTTCAAGGAGTTATGTTGGAGGTTTTTTGGAAAATTCCATCAATGAGTGTAACTTCGACAATAATGTCCACATGAATGATGTGATACGTGATCTTGCAACAAATGTAGCAGGAAAAAATATGTAGGTGAAAATCTCAATTCTAATTATGCTTTCGGTGAAAAGACTCGTCATGTTTCATATGTTCTATTCAAACTATGGTCAGATGTTCTATTCAACTTACGCAAGGCAAAGGATTAAGAACGTTCCAGCTATTGTCATCCAATTCTAAATACGAAAAGAAGAATGAGATCAATGAAGCTATTTTGGATGAAGTACTTTCCAGTTTTCCACGTTTGCAAGTATTGGGTCTTAATAACTCAAATAGTCGCTAAGTGCCAAACTTTATAAGAAGGTTTAGACATCTTCGATATCCAAACCTCCCTGAAAATATTATGAAATCACTTCCAAATTCAATCACTGAATTGCAAAACTTGCAAATGCTAAATCTAATGTACTGTAGCGAACTAATGGAATTGTCAAGGGACACCAGAAATCTTATTAATCTTAGGCATCTTGATTTCAATAGTTCTACCTTAACCCATATGCCCGAAGAGATGGGGAAGTTGAATTGTCCACAGACACTAAGTTATTTTGTCTTGGACTACGAAAGGTCTAATCAGCTGAGTGAACCGACTGTGTTGATCCATTTAAAAGGAGATTTAAGAATAAAAAATTTAGAGCAACTGAGTTACAATCCATCTGAACTCAG

TTTAGTAAACCTGAAAGACATAAAAGGCTTCAAGAATCTGGAACTAGAATGGAACCTAAGCCCAGATGATCAAGAATATGAAGGTGAGGATGATGAAACTTCAGCAATGGAAGGCCTAGAACGACATTCAAATGTTGAATCCTTGCACATTGACGGGTACAGCGGAGTAGGATTACCCAATTGGGTGTCCACCTTGCTCTTGAAGTTAACTAGAATTACAATTTATAAATGCCATAGATTGCAACATCTAACACAGATAGCCCATCTTCAGGCACTCACATTTCTATTTTTGGATGACATGAGCTCTCTTGAGTTCATAGACAAGAATGAACCATCTTCCTCGTCCTCTTTCCCATCTCTCGAGCTTCTAATTATCGAAAACATGCCGAATTTGGAAGGATGGTGGGAATTAGGGAACACTCAAAAAAATTGGTTACCACCAACTTTCTCTACGCTGATTTCCCTGCATATCTCTAGGTGTCCTAAGTTTAGATTCATGCCCAAGCCAGCTTCAACAGGAACAGTTGTGTTCTTACGTGATGTGAGCGTTCAATTGGAGACTACATTAGATCCATTGTGGGGTTTGGAATGTCTAACACTGGAAAAAATTAAGGATCTCAAATATCTAGAAACCATGGAATCTCAGCTAAACATCAGTTCTTTGCCAATACAACTTCGAGATTTAGAAATAAATAAATGCTCGAATTTGATGAGTTTACCTGAATGGATTAGCAGCATTACTTCGCTAGAGGAGCTGGAGATTATGAAATGCCCGAAACTAAAAGTAGCTTTTGCATCGGCGGTTGCCCCGAAGTAG

>Cucsa.133510

ATGGCGGGAGCTTTAATTGGTGGCGCGGCATTGGGTGTTCCGTTTAACGAGCTAGCGACCCTCTTGAAGAATTTTGGCGAGAGGGCGTGGAGTTTCAATTCTGTTCTTAACGAGACCGAATCCAAGGTAAATGATATAATTCCTCTGGTTAAAGAAATAGATGGTCTTAATGAATCCCTGGATTATCCAAGAGAAGAAACGGAGAAGTTGAAAAACTTATTAGAATATGCTGGAAAGCTACTTAGACGGTGTTTAAGAGTGGGGAAGGCTGATTTGATAAGGAAATCAAGTCATACAGAGAAGCTTCGTGAACTGAATGCCAGAATCAAAAGTTTCAGTGACGTTGTGTTGTTCCAAACGTCTAGAGACGGGAAGAAGACATTGAGTTTAGTGACTGAGATCAAGGAAGTCGTTCGCAGGCTTGATAGCAAATCTGGATTAAGCAATCCGGTGGATTTAGTTGTGACGGTTCCTGTGATTTCAGAAGAAAGTGTTGGGTTGGAAAAGCCTGTTGAGAAATTGAAGGCCAAACTATTTAGAGATGGGGTTCGATTGTTGGTAGTGACAGCTCCCGGAGGTTGTGGAAAAAGCACTCTGGCCGAAATTTTTTGTCACGACAAGCAAGTTAAAA

ATGGCGGGAGCTTTAATTGGTGGCGCGGCATTGGGTGTTCCGTTTAACGAGCTAGCGACCCTCTTGAAGAATTTTGGCGAGAGGGCGTGGAGTTTCAATTCTGTTCTTAACGAGACCGAATCCAAGGTAAATGATATAATTCCTCTGGTTAAAGAAATAGATGGTCTTAATGAATCCCTGGATTATCCAAGAGAAGAAACGGAGAAGTTGAAAAACTTATTAGAATATGCTGGAAAGCTACTTAGACGGTGTTTAAGAGTGGGGAAGGCTGATTTGATAAGGAAATCAAGTCATACAGAGAAGCTTCGTGAACTGAATGCCAGAATCAAAAGTTTCAGTGACGTTGTGTTGTTCCAAACGTCTAGAGACGGGAAGAAGACATTGAGTTTAGTGACTGAGATCAAGGAAGTCGTTCGCAGGCTTGATAGCAAATCTGGATTAAGCAATCCGGTGGATTTAGTTGTGACGGTTCCTGTGATTTCAGAAGAAAGTGTTGGGTTGGAAAAGCCTGTTGAGAAATTGAAGGCCAAACTATTTAGAGATGGGGTTCGATTGTTGGTAGTGACAGCTCCCGGAGGTTGTGGAAAAAGCACTCTGGCCGAAATTTTTTGTCACGACAAGCAAGTTAAAA

ATAAATTTCAGAGAAACATCTTGTTCCTCGTTGTCTCAAGCAAACCAGAAACGAAACGCATCTTAATATCTATAATTCAAAGACTCGGGGGGCCTATAGAATCTGGTTCTGTAAGTGATGATGAGGCATTCCGGTTGTTAGAAGTTCGGGTGGGGGAATTGAGTCCAAATCCTGTATTGATTGTGTTGGACGATGTCTGGGACGGTTCTGAATCAAACAAGCTTCTTGAAAAGTTCTCCCGATTACCCAACTGCAAAGTTTTGGTCACTTCTAGATTTAAGTTTCCTGCATTTGGTGAGTCGTATGATTTGGAACCTCTGGACCATAAGGATGCAATGGAGTTGTTTCGTCGCTGGGCATCGAGGGGTAACAGAGTGCTACAGTTCCCAGATGAAAGAATT

GTAGAAAAGGTAATTAGATAATCTGCTAATTTGATTAAAAAGACCCCTAAAAGAATTTTTTTTCCTCACCTTAGAACTCATGGACACAGTTATACAACCTATAGAAAAGATCCAGTTCAAGTAAGGTCGTAAAAACTTTTTCCATACAAATGCAACTGAAGTTTTGAATGACTGAATTGCAG

ATAGTGAGGGGTTGTAAGAGATTCCCACTTGCTCTGAAAGTGATTGCAGGATCACTTTCGGGTAGAGCCACTTCGGTTTGGGAAGTTACGGGGAGGAAATTATCTAGAGGAGATTCTATTCTGGGTTCTGAGAAAGAGCTTCAGAAGTGCCTCAAAGACACCTTAGATGCAATCCCAGATGACAAGATAGTTCTCAAGGAGTGTTTCATGGACTTAGGTTCATTTCCTGAAGATCAAAGAATTCGTGCGGCTACCTTCATTGACATTTGTGCAGTGTTGTATGAACAAGATGAATGTGAAACAATGTCAAACCTTGATGAGCTCTTCACCCGGACTTTAGTTAACACTGTCTCTTTGAG

GTAAAATTCTGGGTCTTTCAAGTAGAGATGTCCACGGGTCGGGGGCCGGGACGGTGATGCACTCCCGTCGCTGCCCCCTACTCCCATATATATATATATATATGTTTAAACAGAAACAAATATATATATATATGTTTAAACAGACACATAATATATATGCTTAAACAGAAACACATATATATATATGCTTAAACAGAAACACACACACACATATATGCTTAAACAGAAACACATCTATATATGTTTAAACAGAAACATATATTATAGACCGGAGAATGTATTCCCGTCCCTAGCCCCAAAATGTCAACGTGAAAAAATAATCCCCACTCCCTTCCCCATTTCCTGTGTATTCGGTCCTCGTTCAGGGTGGGTCCGTTGCCCCGCAGGTTAAATGACATCTCTACTTCCAAGTTGTGTTGGTTTATGTTGATCTTGCTTCTTGGCTACATAATTGACGTTTTTTCTTAAATAAAAACTGTGTTTTATTATAGTTTGATATGACAATTCTGGCAAAAAATTGGTGTTTCGTTAGTATTTTGTGGATTATGTTGTAAAACATATGTACAAGAAACAAAGGGATTCTTTTGGGTATGGTTTTCTTTCAATTATTTACCAGAAGAATCTTGTCTAGAATTGATATGTGCCGATATATTTTCTTCCCATCGTTTTAGACTAACTTGAAGAACTATAAACATAAATTGATCATTGATGTACACTCAAAAAAGTATTTCAAATTATCTTGTGTAAACTCTTACTTTATGAACAG

AAAATAAAGATTGCTTAGTAGAAACGTATTCTACTATTGATTTATCCTTTTGTTCTGGGAAACAGTCTCTAAATCAACTTCAAATTTGACTCTCAAAGATATTTTGAGTAATCATAATTACTAACTCAACCTTAATGCATAAGTATCTGAATATAAACTGCAAGACTGTGGACTGTGCTTGACATCCATTTGGTTATGAAATTGAAGTTCTAGTCTTGTTGTGATAATTTTCCAGAAATAAAGCGCATGAAGATGATTACTACAGCGAGTCCTATATTACACAGCATGACGTACTTAGAGAATTGGCTGTCCTTTTGACTAATGAGCAGCCAGTAGACCAAAGAACAAGATTGCTTGTGGATATTAACAAAAATGAATTTCCCAAATGGTGGTCTGTAAGACAGATGCAACCTGTGAAAGCCCGCCTTTTGTCCATAACAACAG

GTCCCTCCGTGGTCTCTCTCTCATGTTTCGATATGTCATTTTTCATATTGTCGTTGTACAATCTAACACTGGATGAGATGTCGATGGTGCAG

ATGAGAAGTTCTCATCATGTTGGCCTGATATGGAAGCACCTGAAGTTGAGGTGTTAATTCTAAATCCTGGGTCAGAAACTTACAAGTTACCTGATTTTGCAAAGAAAATGAACAGATTGAAAGCGCTGATAGTCAGGAATTACAGGTCCTTTCCAACTGAATTGACAAGTGATTATCAATTAATCAATTGTTTGTCAAGGCTAGAAAGAATCAGTCTTGAGCGGATTTCAATATCTTCTTTCATTGACCAGAACCTGAAGCCCCTGTGGCATCTTAAGAAGCTATCGTTCTTTATGTGCAAAATTGACAAAGCTTTCACAGACTGCTCAACTCAGATCTCATACATGTTGCCTAACTTACTTGAGATCTCCATAGATTTTTGCAACGATTTGGTGGCTTTCCCTGTCGGACTATGTGAAGTTGTCACATTGGAGAAACTGAGCATTACAAACTGTCATGCATTATCTTCGTTACCCGAGGAAATTGGGCAGTTGATTAATCTAAAAATTCTAAGGCTTAGATCTTGTATTCATTTGGAGAAGTTGCCAGAATCAATCTCAAGGCTCCGGGAATTAGTTTATCTTGACATATCTCATTGTGTTGGCCTTACCAAACTTCCAGATAAGATTGGCAACTTGCAGAAGTTGGAAAAGCTTAATATGTGGAGTTGCCCGAACATGCGCAAGCTTCCAAAATCAGTAGGAAATCTAAAAAATTTGAAGGAAGTAGTTTGTGAAAGCGAGATGAAAATATGGGTGAATTTTGTCGCACCTCGGCTTGGCAATGTGGTAAAAGAACACAAGGAAGAAATCAACTTGGATTTTCTAAATTGATGACAGCTTCTTATGAACCTTTTTTAAGGTATGCGTACAATTAAATATTATGGATCATGGGAGCATGGCGATGTATTTATAAGAGTTCATCTTCAAAGTATTGTTATGGTGGGAGAGAAATAAGAATCATTCAACAACTCGAGGACGGAAGAGACTTCTGTAAAGCTAAGCTTAAGGCTTTGTGCTATCGCTGCAACAAAGGACCTTTTTTGTCGAATGCTTCTTTTAGCATTCAAGCGCAAATTGCTGAAGAAAATCCAAAAAATGTGTGCATTTTCTTATTTTTATTTACGCTGCCTTGCCTCTCAAAAGCTGCGGCTAATTTCTTCAGAATTCAGTGGGAAGAATTATCAAGCAAGTAAAATCTGAACATATGTATGAAATATCTGGAATTAGAATCTCAAACTAGGCAACTTATAGATCAAACTTCTAAGTTTTTTTTAGTTCAACTGCTGCTGTGGTTGGGAATTGTATCATCACTCTTTAGAATGGTAATTGATGTATCATAGTACTTGACTGAAATCCACGAGCTTTTCCTTTAAGAGCAATCAATTGTTTTAAGTCGATAAAGTATGGTATAGCTCATCTTGTAATACTTTTGTTAGAGCTGCTTTGTGAACCATATGGGATTTTGGGTTTCATCAAAATGCAAGGTTTTGTATTGTGATCTTTGCAACATAACATTCATTTTGGCAATGGATTTTGA

>Cucsa.237470

ACAATACCAATGTATTAGATACAAAATGTAAAGTTCATGCTACTAATTAACTATGCATCCAACGCTTGCACATACTTGTTATCAATTGGAAGTCTTATTACAACTAAAAAGAGAGATGTACTTCATCAACTAAATTATAGAGATAAAGTGCTAGAATACAAGGTGGAGTTACTTTCTCGTGAAAGTGCTTACTCACTGTTTAGCAAGAATGCATTTGGAGGTGGCCCTTCCGATAAAGATGAACTTTGTAATGAAATTGTGGAAAAGGTTGGAAGACTTCCATTAGCTTTGAAAACCATTGGCTCCTATTTGCATAATAAGGAGTTGGATGTGTGGAATGAAACATTGAAGAGACTAGATGGAGTGGAGCAAGACTTCTGTGATACAGTATTGCAGAAAAGTCAGAAGAATTTACACTAATAATACTCAAGATCCA

>Cucsa.237480

ACAATACCAATGTATTAGATACAAAATGTAAAGTTCATGCTACTAATTAACTATGCATCCAACGCTTGCACATACTTGTTATCAATTGGAAGTCTTATTACAACTAAAAAGAGAGATGTACTTCATCAACTAAATTATAGAGATAAAGTGCTAGAATACAAGGTGGAGTTACTTTCTCGTGAAAGTGCTTACTCACTGTTTAGCAAGAATGCATTTGGAGGTGGCCCTTCCGATAAAGATGAACTTTGTAATGAAATTGTGGAAAAGGTTGGAAGACTTCCATTAGCTTTGAAAACCATTGGCTCCTATTTGCATAATAAGGAGTTGGATGTGTGGAATGAAACATTGAAGAGACTAGATGGAGTGGAGCAAGACTTCTGTGATACAGTATTGCAGAAAAGTCAGAAGAATTTACACTAATAATACTCAAGATCCA

>Cucsa.237500

ATGAGTACTTTTGATACTTTCATAAGTTTTAGAGGCGAAGATACTCGTAACACGTTCACCGGACATTTGTACAAGGCGCTGGTTGATTTTGGAATATCGACTTTTATGGACGACAAGAAACTCTTGATTGGAGATAGTCTTAGTGAAGATCTTATTGGAGCTATAGAAAAATCAGGGTCTTTCATTGTTGTTTTATCAGAGAACTATGCTTCTTCAAAGTGGTGCTTGAGAGAATTGGTGAAGATAATTGGTTGTATGGTGGAACAAAAGCGTCGAGTTCTTCCTGTATTTTACCACGTGAGTCCTCATGATGTTCGACATCAATCAGGGTGTTTCAAGAAAAGCTTTTGTGAATATGAAGAAATTCTTCAAGAGCTCAACGATAGGGAAGGAGATAAATATACGAAGGAGGTTCAAGAATGGAGGAGTGCGTTGACAAAAGTTGGCGAGCTCACTGGAGTAGTTGTAACAAAAGATAG

GTGATGTACCTATTCAGTTTTGATTTTTTTGTTGTTGCTAGTTCTACAATAGTAATTAGAATATAATGATAATCATAGACAAAGGACGGCCATATATGATAGACCACACTATCACTATCTATCACATATAGATACTAGTAGTTGTGATATATATAAATACATTTTGGTATCTATCATTAGATAGACGGGGATAGAAGTCTCTTAGCGTTCATCTCAGTGATAAAAATTGATACAAGTTTGTAGTTGATAGACCCATACTGAATAAAAGTCTATCGATGTCTATAAGTGAAAGAAACTGATAGAAGTTCATAACGGCTGATAAAGACTAATAGAAGTTTAACAATGTCTATCATTGATAAGATTGATAGAAATCTCTCATTGATATACTATAGTGAATAGAAAATTATTCAAATCAATCATAGAAGTCTATCAGTGATAGAAATTGATGGTTGTCGATCAAGAGGCTGATAGAAGTCTGTCAGTGTGTATCAATGATAGAAAGAGATAACACAAGTTTATCATTGATAAAAGTTTATAAAAGTCTTGCCTATTGGTGACAAAGAGTAAGTGTGATATGGTACTATCAATGATATAAGTGTATAACATTCAATCGATGTTAAACAATGATAGAAGGATTCTTTCTCTCACTTATCACCTAAAATCTACACATCTCAGATTTTCAATCTCTCGCGCTCTCTCTCAAGAAAAATTTGTTTTTAAAGAATTGTTAGTATATAGTAGTTAATGTTCACTGCTTTGTTTTTATAGAGACAAATCCACTGTTGAGGATAGACAATAATGATAGTGGGATAGACAATGCTAGATAGTATTTTATATAACCCTCTATCATATGACAGAACTAACCATTTAGTTTTGTTTCTAACGGATAAATTTTATCCATTTACATTAACAAATAGGTTTATAGTTTATAGTTTCTGTGTCAATCTATTAAAAAGAGAAAAGTGGTACTCTGTATTTATAGCTTTTTTTTTCTTTTGTTTTGATAGATGATTCATGCTAATTCTAAAATATTACTCTATTAGATTTTTTTCTTCTATGAAACCACTATCAACTAGACAATTTTATAATTGTCGATAAATAGTGATAAATTACTATTATCGTCTATCACCCACAAACATTGATTGACTACTATCGTCGTAATTGATAGTTGGTACATGGCAGATAATTTCCATAGCTATTATACATTCTTAACCAAATGTCGTCTTCTTCATCCACCCTTTCAATTTTATCTCATCCTTTGTTTTCTACTGTTCTCTCTCTCTTCAATCTCAGTATCCTGATCTACTTTCCCCATTTTCTTTGGACTTCCCTCCTTTGATATGTAATATTGAATATAGTATGCATGCAG

TCTTGAAGCCGCTAGCATCGACAAAATCACAGAACAACTAAGTTCTACGTTGCATCAACAAAAGTTAGTAAATTTGGACGAGCTCACTGAGTTAGTTGATATTGAACGTCAGTTATGCAAGATGGATAAGCTAAATGATTTGGAGCCAAATGTGGTACGTTTTATAGGGATAATAGGGATGGGCGGAATTGGTAAAACAACCATTGCTGAAGTTTTTTATGAAAAAGTTGCATATAAATTTGGAAAAAATTGTTGTTTTCTTCGCATTTATGAACACACTACTTTACTCTCACTTCAACAACAACTTCTTTCCCAACTTCTTCAAACAAAGGACATAATTATAAACAATCAGAATGAAGGAGCAAGCATGATTGGAAGTCGTTTGAAAAATAAGA

GTTTTGATTGTTCTTGATGGGGTGAAAGAAAAAAGTCAGTTAGAACAGTTAGTTGGAAATCCTAATTGGTTTGGTCAAGGGTCCAAAATCATCATTACAACCAGAAATAGGGATGTTCTTCGTCAGCCAAATTATAAAGATAAAATGGTTGAATACAAAGTGGAGTTTCTTGATAATAAAAGTGCCATGACACTCTTTTGCAAACAAGCATTTGGATCATGTGATCAGTTTCCCAGTAAGAATTTTGAGGACTTTTCTAAGGAGATTGTAGAAAGGGTTAAAGGACATCCACAGGTTTTGAGACAAATTGGGTCGTCTTTATATGATAAAGGTATAGAGATATGGAAAGAACAATTGAAGAGTCTTGAGGAAGATTACAACAATCGTATATTTAAGACATTAAAGATAAGTTTTGATGATTTAGGAAAGACAAGCCAAGAAGTTTTTCTTGATTTTGCATGCTTCTTCAATGAGAAGAAGAAAGAGAGTGTGATTGAAATACTTAAGAGTCTTGATTATAGACCTCATAGCGAAATACAATTGTTGGAAGATAGATGTCTCATTGAAGTAAGACGTGACAACACAATATTTATGCCTAAGTGCATTCAAGCTATGGGTCAACAGATTGAACGTGAAGCTGATAAACGGAGTAGGATTTGGCTTCCGAAAGATGCCCATGATGTATTTGATGAACCACATGTAAGACTGAAATATAAAAGTTTATATATATACATATATATATATAAATGTTTGTTTATCTTTCCCTTTCTTATATGTTTTAAATAATGTTTATTTACAGAGAG

TAAAGGACATAAAAGGTGTAGTCTTGAAATTGGAAGAGAAGCAAGAAGAAGTAAAGTTAGAGGGTAAGGTTTTTGAGGATATGAGAAGTTTAAAAATATTGGAAATTGGGAATGTAGAGGTGAGTGGAGACTTCACACATCTCTCAAAACAATTGAGATTGCTCAATTGGCAAAGCTATCCCTCACAATGTTTGCCATTACGTTTTGAATCAAGATATTTATTTCAACTTCTTTTGCCTCTAAGTCAAACAAGGCAACTTTGGAATGGTCAAAAG

GTTAGTACATACAAATATCATATCATATATATCATAGGTATAACTTTTTTTAAAATGCAACCCAACTGTGTTTTGTGACAG

GGATTTGAGAAATTGAAGGTTATTAATGTTAGCCGTTCGAAGAATTTACGAGAGACTCCTAACTTTACTAAGGTTCCAAATCTTGAAAGTTTGGACCTAAGTTATTGTCCAAGGTTGTGGAAGATTGATTCTTCTATTAGTCGTCTCAATCGTTTGACGTTGTTGGATGTATCGTATTGTATCAATCTTGAAAGCTTGCCATTTTCTAGAAGCTGCAAAAGCCTCGCAAGAATAAATTATGCTGGCTCAGGTCTTGAAGAAAGAGGTATATATACGTACCTTTCATTATGGTAA

>NBS_scaffold00894_2639023_2640408_2kFLseq

MTDKMEENEKQREVSKVLISFRGEDTRSNFTSHLNMALRQRGINVFIDNRISRGQEISASLFEAIEESKISIVIISQNYASSSWCLNELVKIIMCKELRGQVVLPIFYKVNPSQVRKQNGAFGEAFAELEVRFFDKMQAWGEALTAVSHMSGWVVLEKDDEANLIQKIVQQVWKKLTCSTMQLPRQFENLLSHVMIDGTRMVGLHGIGGMGKTTLAKTLYNRIADDFEGCCFLANIREASKQHEGLVRLQEKLLYEILMDDFIRVSDLYKGINIIRNRLCSKKILLILDDIDTSEQLQVLAGGYDWFGYGSKVIVTTRNEHLLDIHGFNKLRSVPELNYGEALELFSWHAFQCSSPPTEYLQLSKDAVNYCKNLPLALEVLGSFLYSTDQSKFKGILEEFAISNLDKDIQNLLQGIQKLMNLSLLTINQWNKVEMHDLIQQLGHTIARSKTSISPSEKKLLVGDDAMHVLDGIKDARAVKAIKLEFPKPTKLDIIDSTAFRKVKNLVVLKVKNVISPKISTLDFLPNSLRWMSWSEFPFSSFPSSYSMENLIQLKLPHSAIQHFGRAFMHCERLKQLDLSNSFFLEEIPDLSAAINLENLSLSGCISLVKVHKSVGSLPKLIDLSLSSHVYGFKQFPSPLRLKSLKRFSTDHCTILQGYPQFSQEMKSSLEDLWFQSSSITKLSSTIRYLTSLKDLTIVDCKKLTTLP

>NBS_scaffold01037_342640_345492_2kFLseq

MGDLLTFGVQETLKQAVTLVAKKIIASSEFKVVLEELKDDLLHAEWILHAIKTKHDHSLNDKITHWVNDLQLIVYEAEDMLDLFAYDDVERKIRSNKSEVEQVEETTSLLENYVVGREMEVESIVQDVTEASQQQLNSILPVYGTGGSGKTTLAQLVFNDERIGKQFHHTVWVCVSQPFVINEILQSILKKVSKSNDNRSKDDKDTLIRNLKEVMGGKRYFLVLDNVWNENKIFWEKLKECLMSIVEELGSSVLVTTRSRKIAEMMKETLDTYHLNKLTDDQCWSVFSYFAKANAVPITSNLELVREELSVDRLPKASIKQCFAYCSNFPKGYWFDKKQVIKMWMAHGFTRPDEGNNETMEDTGERYFNILLSYCLFQDADDDKWHIGRKFRMHDLIHDIACDVSSDKRLQLDHSSSSKWKGLTEEKKKIESKLRTVIDFGRNENVRELPNSISKLKHLRYLDISRCYSIKKLPESIVGLFCLTNDLLPTNLRRMVSLRHLEILLMGIDLPPKFEMPPYLSELVQLQTLFAFAVGFETGRKISELRGLRNLK

>NBS_scaffold01037_946157_948813_2kFLseq

MQLRVPKYPVGIDIQVDNLLFHVVSDELITMVGLYGIGGIGKTTLARALYNKIVDDFESCCFLANVREASNQYRGLVGLQNELLREILVDDSIKVSNLDIGISIIRDRLCSKKILLILDDVDTSEQLEALAGGRDWFGPRSMVIATTRNKHLLAIHEFDILQSVKGLNDDEALELFSWHAFKTSCPSSDYLDLSKRVVRYCKGLPLALEVVGEDINKVKLMLEACGCLCLEKRTTKLMNLSLLTIDESNQVEKVVYKFGFLRNNGSCHPFIEKLFTLIVHEYKKDGGIKELTLMNCEIPNWCRYKSMNNSLTFLFLVIDYLSWKKKTFIAPCVKFQVTTIDHERVQIECRVFINDIEVWGGMSSSIFDLRLISGKISRGEYLWMTVLHPCRHRLNSCGDDIMDSSPNSSIGMLDKIRVLFEINYPKCKDGNNLEKNLGKLKLEYANLEKGSN

>NBS_scaffold02023_358423_362135_2kFLseq

MEGCVSFRSFSFAITCESLETLVLSNCGLEFFQEFGCLMGYLTELHIDGTFINELSISITNLFSLILLNLRNCIRLPCLPTEIGSLSSLKTLILNGCKNLDKIPSSLGNVKPLEELDIGGTSISIIPFLENLRILNCERLKSNIWHSLASLPANYFSSRRDLNLSDCNLVDEDIPNDLKLFSSLEILDLSSNHFEKLSESIEQLINLKAFYLNDCPELKRVPKLPKSTKYVEGEKSLGMLRTSEGKFYFMTGSLACTMSVMSPPSNARLLKKKVKFLRFQEYSTKEVHITKDMGKQTNHKLVLAHKTSLVGMENQVEKACNLLDLERSKNILFVGIFGSSGIGKTTIAEVVYNTIVDEFQSVHERNQIQRLVGSPNWFAPGSRVIITARNRDVLHELNYRDQVQEYKVELLSRERAYSLFCENAFGDGGPSDKKDLCSEIVEKVERLPLALRTIGSYLHNKDLDVWNETWKRLDEVEQISLIQYCREIRRKLHQHHLRECYHLRIIHDSAVGRFLDKLVILDLEGCKILERLPRYISNSKSIEVMNLDSCRKIEQLFDNYFEKFPSHLKFESLK
