# Supplementary material for: Frequent loss of lineages and deficient duplications accounted for low copy number of disease resistance genes in Cucurbitaceae
Source: BMC Genomics. 2013 May 17;14:335. doi: 10.1186/1471-2164-14-335 (PMC3679737; doi:10.1186/1471-2164-14-335)
Supplement: Additional file 3 — Re-annotation of R-genes in melon. [file 1471-2164-14-335-S3.docx]

**Additional file 2:**

**Re-annotation of *R*-genes in melon (Introns were marked in red)**

>MELO3C000252

CCTTCAAGTTCATCGTAACTTACTTGAAGAAGATTTTGGATATCTTTGTCAAGGTTGGAGATTGCAAATTCCTCCAATATACCTCTAAATTTGGATTGATCAGTAGAATAAAGGAATGAACCTAAAACTTCAAGTGCTAAGGGAAGATTTTTACAATAATTTACGGCATCTTTTGAAAGTTGTAAATACTCGCTTGGTGGATGACTACACTGAAAAGCATGCCAGCTAAAAAGCTCAAGAGCTTCATCATAATTCAATTCAGGAACACTTCGCAATTTATTAAATCCATGAATATCAAGTAACTGTTCGTTTCTTGTTGTCACAATGACCTTACTTCCATATCCAAACCAATCGTATCCTCCGGCTAATGCCTGTAGTTGTTCACTTGTATCTATATCATCAAGAATTAAGAGAATCTTTTTGGAGCATAGTCGATTCCTTATGATGTTGATTCCTCTATAAAGATCACCAACTCTAATAAAGTCATCCATTAAAATCTCATAAAGTAGTTTCTCTTGGAGTTTAACAAGGCCCTCGTGTTGCTTTGAAGCTTCTCTAACATTTGCTAAAAAGCAATAGCCTTCAAAGTCATCAGCAATCCGATTGTATAATGTTTTGGCTAAAGTTGTCTTGCCCATACCTCCAATTCCATACACTCCAACCATTCTAGTTCCATCAATCATAACATGAGAGAGCAAATTCTCAACTTGTCTGTCAATTCCAACTGGATATTTAGTTACAGGCAACTGCATTGTTGAACAAGTTAATTTCTTCCAAACTTGTTGAACAATTTTTTGTATCAAATTAGCCTCGTCA

>MELO3C004259

ATGGGTTCTTCTGTTGTTCGAGTTGGATCATCTTTTTCTGATCCTAACTGCAATTATGATTATGATGTGTTTTTTAGTTTTAGAGGAGAGGATACTCGCTCCAATTTCATCAGTCATCTTCATATGGCCTTGCGTCTAAAAGAAGTCAATGTTTTCATAGACGACAAACTCAAAAGGGGTGAGCAAATTTCTGAGTCTCTTCTTAAATCTATAGAGCGATCTAGACTTTCCCTCGTTATTTTCTCAAAAGATTATGCATCTTCAACTTGGTGTTTGGATGAACTGGTGAAAATAATTGAGTGTAAGAAATCCAAAGGACAAGCAGTTTTGCCGGTGTTCTACAAGGTGGATCCGTCTGAAGTTCGAAAACAAACCGATTGGTTTGGGGAAGCATTGGCCAAACATGAAGCTAATAAGTTATTGACCAACAAGATTCAACCATGGAAGGAAGCTTTGACTTTTGCTGCTGGTTTGTCTGGTTGGGATCTAGCAAATAG

GTATTTTTTTTTTTTTTAATCTTCCAAGACTCATTGTCCAAGTGAAGTTTAAATTTATCAGCTACTATTTTCGATTTTCATTTTTATGATGTTACTTTACAACAG

CAAGGATGAGGCTGAACTTATCCAAGAAATTGTTAAACGAGTATTGTCTATAGTAAATCCAATGCAATTACTACATGTAGCCAAACATCCAGTTGGAGTTAATTCTCGACTAAGGAAAATTGAGGAGTTGGTCTCTCATATTGGGTTCGAGGGTGTTAACATGGTGGGGATGTATGGCATTGGAGGCATTGGTAAGACCACTTTGGCTAAGGCTTTGTACAATAAAATTGCTACCCAATTTGAAGGATCCTGCTTTCTACTAGATGTTAGACGAGAAGCTTCAAAGCATGGGCTCATTCAACTACAGAAAACCTTACTCAATGAGATCTTAAAGGAGGATTTGAAGGTTGTCAATTGTGATAAAGGAATTAACATCATAAGGAGTAGACTGTGTTCAAAGAAAGTTCTTATAGTTCTTGACGATGTGGATCATCGTGATCAATTAGAAGCATTGGTTGGTGAGCGCGATTGGTTTTGTCAAGGTAGTAAAATCATTGTGACGACAAGGAATAAACATTTACTTTCTAGTCATGGTTTTGATGAAATACACAATATTCTAGGATTGAATGAAGACAAAGCTATTGAGCTTTTTAGTTGGCATGCTTTCAAGAAAAATCATCCATCAAGTAATTATTTCGACCTTTCAGAACGTGTTACAAGTTATTGTAAAGGTCATCCTTTGGCTCTCGTTGTTTTGGGTTCTTTCCTTTGTAACAGAGATCAAGTAGAATGGTGTAGTATTTTAGATGAATTTGAAAACTCTTTGAACAAAGATATCAAAGATATTCTTCAATTAAGTTTTGATGGGCTCGAAGACAAAGTAAAGGATATCTTTCTTGATATTTCTTGTTTACTTGTGGGAGAGAAAGTTGAGTACGTTAAGGATACGTTGAGTGCATGCCATGTAAATCTAGATTTTGGAATTATAGTACTCATGGATCTTTCACTTATTACGATTGAAAATGACCAAGTGCAAATGCATGATTTAATAAAACAGATGGGTCATAAAATAGTTTGTGGTGAATCTCTTGAGCTTGGAAAAAGGAGTAGATTATGGTTGGAGAAAGACGTTTTGGAGGTGTTTAGTAGCAATTCA

GTGAGTAATTCTTATCTAAATTATCTTTAGTTTAATTATTTTCAAGACTCAACACCCCGTTAATCACTAAACGTGTAACTTTGTAG

GGAACAAGTGCAATTAAAGCCATAAAATTGGAGTTCCATAATCCCACAAGGCTAATTGTAGATCCACAAGCTTTTAGAAACCTGAAAAATTTGAGATTGCTTATCGTTCGAAATGCAAGATTTTGTGCAAAGATAAAGTACCTTCCCGAAAGCTTAAAGTGGATTGAGTGGCATGGATTTTCTCAACCATCTTTGCCTTCGCACTTCATTGTGAAAAATCTTGTTGGACTAGATTTGCAACATAGCTTCATCAAAGACTTTGGGAACAGACTTAAG

GTAACTTATATTTGTACGACTTGGGTGTATTTTTATCAGAAGTTTCTTCCTATTGTCTTTCAGTAAAATTAGAAACGGACAGAATGGACAAAACATTTTCACTTCATAGCAAAAATATGAAAATTAGAATTTTTTTTTTGGCTGTATTTGCAAATTTAGAAAAATATTGTAGATCTTTTTGCAACTCATTACAATTTCGAAAACTAATGTAAATAGACTATTAATCTTGATCTGGATTTCCATCACTGATTTGCTTTTATATATGGTGTATTATTAGTTTATCTTATAGACTGATTGATTGATGGTTATTATAG

GTTGGTGAATGGTTGAAGCATGTTAATCTTAGCTATTCTACGTCATTGAAGAAAATTCCCGATTTCTCTGCGGCATCGAACCTTGAAAAATTGTACTTAAGGGATTGCACAAATTTAAGAACAATTCATAGGTCCATTTTTTGTCTTGTGAAGCTTACTCTCCTGTGCCTTAGTGGTTGCTGTATGATTAAAAAACTTCCGACAAGTTGTTTCAAGTTATGGTCTCTTAAACATTTGGATCTCTCTGGTTGCACAAAACTTGAGAAAATTCCAGACTTTTCCTCTGCATTAAACCTCGAAATTTTGCATCTCAGTCGATGCACAAATTTGAGAACAATACATAACTCTGTTTTTTCTCTTCATAAGCTCATTTCCCTATATCTTGACTTTTGTTCCACTCTTAAAACGCTTCCAACAAGCTGCTTCATGTTAACGTCTCTCAACACTTTGACTCTTTATTCCTGTCAAAAACTTGAGGAAGTTCCAGACTTGTCCTCCGCATCAAACCTTAACAGTTTGAATGTCGAAAAATGCACAAATTTAAGAGGGATTCATGAGTCTATTGGATCATTGGATAGGCTTCAAACTTTGGTCTCTAGGAAATGCACTAACCTTGTAAAGCTTCCAAGCATCCTCCGGTTAAAGTCTCTGAAGCATTTAGATCTTTCCTGGTGTAGTAAGCTTGAAAGTTTCCCAATAATTGATGAAAACATGAAATCTTTAAGGTTTCTGGATTTGAGTTTTACTGCAATAAAAGATTTACCTTCATCAATTGGATATCTTACCGAGCTCCCTCGATTAAACCTTGGCAATTGCACAAGCCTCATCTCCCTTCCCAAAACAATTTCTTTGTTAATGTCCTTGTTGGATCTTGAATTAAGGAATTGCAGGTCTCTTCAAGAAATTCCAAACCTTCCTCAAAATATACAGAATTTGGATGCCTATGGCTGTGAATTGTTGACTAAAAGTCCAGATAACATTGTGGATATAATATCACAAAAACAG

GTTCTGTCTCTTTCAATTCAATTATTTATTCTGATCTGTTGTAAACAATTTTATGCATATGAATTTTTATTCAGTTTAG

GACCTCACATTGGGTGAGATTTCAAGAGAGTTCTTATTAATGGGCGTTGAGATTCCAAAATGGTTCAGCTATAAGACTACATCAAATTTGGTGAGTGCTAGCTTTCGTCACTATTCAGACATGGAAAGAACTTTGGCTGCATGTGTTAGTTTCAAAGTGAATGGAGATTCATCTAGAAGAATTTCATGCAATATATTCATCTGCAATAGATTCCACTGTTCATTTTCAAGACCATTTCTTCCATCAAAATCAGAGTACATGTGGTTAGTAACAACTTCTCTGGCATGGGGCTCCTTGGATGCGCAGGATTGGAATAAAGTTGTGGTTCTGTTTGAAGTTGATGATGAGGTTAATCTGAGTATAAGAAGCTATGGTGTCCATGTCACTGAAGAGTTCAATGGGACACAAACAGATGTCAAGTGGCCAGTGGTAAATTATGGTGATTTTTATCAACCGGAGAAATTGCAAAATCT

GTAAGTTCACTTTTACTTTGTACTTTTTCTTTTTGTTGGTTTTTTTGGAGTGAATCTATGATAGACTCCAAAAAGGGAGATGTGTGTATGATATGGTTATGCTGTTTTAATGGTACAG

GGATATTGAGGATATTCTTGTCAAACGTTTATTTGATGAACTCTCCTACTTGTCAAATTGCAAAGCAGTGTTGCATGCAGGAAGTTATGATCCAATAGTAATAACCGATTCCAACATACAACCTATGATTTTCCCATTGCATGTAACATATAGTGGTTATACAGTGATAAGCGGAATGGAAGGCATGGGAAAAACTGCACTCGCAAATTCTCTACGCAACAAATTTAAAAGGAAAGATAACAGCAATTGGGGACAATGTTTAAATGATTCTTCAAGATTTTACTTGCTCCAAGGAAGAAAATCCCGTATATTTTCAGGATATGCGAACCCCAGTAAGCGACGAATATCCTCCAAAAGGTATTATTACATAACCTTTGTAAATTTGGATGTTATAGAAGCTCAAAATGTAAATGCATGGTTTACGGCACAACGTTGGATTATATGTTGTTCTCCCCTTGAAAGTTTACGAAGATGTAGCCATTTTGTTATTACAAGTGTTGACCCATCCTTATGGCACACCTGTGGGGTTGATGACGTCCTCAGCTCAACGTTTCAAAAAAAATTCAGTGAACGGCGGGCTTATATCTTTGGAATACACTTACCAGACAATTTCCTCATGTAATCGATTAGAAGGGTATATGACCTTCAAACTTGAAATTAAGGATATCATCTTCAAACTTCTTTCTTTCTGTTCATAACGCAC

>MELO3C004260

ATGGATTCTTCCACGGAATCATCGACATTCAAATGGAGTTATGATGTGTTTTTGAGTTTCAGGGGAGAGGATACTCGCACTAATTTCACTAGTCATCTTGATATGGCTTTGCGTCAAAAGGGTGTCAACGTCTTCATAGACGACAAGCTTGAAAGGGGTGAGCAAATTTCTGAATCCCTTTTCAAATCTATACAGGAAGCTTTAATTTCTATTGTTATATTCTCTCAAAATTATGCATCTTCTTCCTGGTGTCTGGATGAATTGGTGAAAATAATTGAGTGTAAGAAATCCAAGGGCCAGATTGTTTTGCCAATTTTCTATAAGGTGGATCCGTCGGATATACGAAAACAATCTGGTACCTTCGGAGAAGCACTGGCCAAACATCAAGCTAAGTTCCAAACAAAGACCCAAATTTGGAGGGAAGCTTTAACTACTGCTGCTAACTTGTCTGGTTGGGATCTAGGAACCAG

GTATATATTTTTACAGACATTTTGTTTTTTCTATTCCAAATCTCATTTTTATGCTCTGTATGTATGTACGTATCTATGTGTATGTATGTTAATGAGCACACTACCAATTAATTTTAATCTTAGAAGATTCATTCTTTCTCACGATGCAATTTACTTAACTGTGTTCATCATCTTTTAATTTTGGTATATGATTATGCAATGAACAACAG

GAAGGAGGCTGATCTTATTGGAGATCTTGTTAAAAATGTTTTGTCTACATTAAATCGCACTTGCACGCCTTTATATGTAGCTAAGTATCCAGTTGGAATTGATTCAAAATTAGAATATATGAAGCTTCAGTCACATAATCTTTTTGAGAAGAGCAACAAATTCCATTATCAGACACAACATGAGTATGAGTTTGATACGGGTGTTTACTTGGTGGGGATATATGGCATTGGAGGTATTGGTAAGACAACTTTGGCTAAAGCTTTATACAACAAAATTGCTAGCCAATTTGAAGGTTGTTGCTTTCTATCAAATGTTAGAGAAGCTTCCAAGCAATTCAATGGCCTTGCTCAATTACAGGAAAGCCTACTCTATGAGATCCTAACGGTTGATTTGAAGGTTGTCAACATTGATAGAGGAATTAACATCATAAGGAATAGATTGTGTTCGAAGAAAGTTCTTATAGTTCTTGATGATGTAGATAAGCTTGAGCAGTTAGAAACATTGGTTGGTGGGCGTGATTGGTTTGGCCAAGGCAGTAGAATCATGGTGACAACAAGGAATAAACATTTACTTTCTAGCCATGGCTTTGATGAAATACACAATATTTTAGGATTGAATGAAGACAAAGCTATTGAGCTTTTTAGTTGGCATGCTTTCAAGAAAAATAGTCCATCAAGTAATTATTAG

>MELO3C004262

ATGGATTCTTCCACGGTTATAATAGAACCACCGACTTTCAAATGGAATTATGATGTGTTTTTGAGCTATAGAGGAGAGGATACTCGCACCAATTTCACCAGTCATCTTGATATGGCCTTGCGTCAAAAGGGTGTCAACGTCTTCATAGACGACAAGCTTGAAAGGGGTAAGCAAATTTCTGAAACCCTATTAAAATCTATACAAGAAGCTTTAATTTCTATTATTATATTCTCTCAAAATTATGCATCCTCTTCATGGTGTCTGGATGAATTGGTAAACATAATTGAGTGTAAGAAATCCAAGGACCAGATTGTTTTGCCAGTTTTCTATAAGGTGGATCCGTCGGATATACGAAAACAATCTGGTAGCTTCGGAGAAGCATTGGCCAAACATCAAGCTAAGTTCAAAACAAAGATCCAAATTTGGAGGGAAGCTTTAACTACTGCTGCTAACTTGTCTGGTTGGGATCTAGGAACTAG

GTATATATTTTTACAGACATTTTGTTTCTTCTATTCCAAATCTCATTTTTATGCTTTGTATGTATGTACGTACGTACATATGTATGTACCTATGTATGTATGTTTATGTGTATATATGTTAATGAGATCACTACCAAACAATTTTAATCTCATAAGATTCATTCTCTCTCAAGATTCAATTTAATTAATTATGTTCATCATCGTTTAATTTTGGTATATGATTATGCACTTGACAACAG

GAAGGAGGCTGATCTTATTGGAGATATTGTTAAAAAAGTGTTATCTACATTAAATCGCACTTGCATGCCCTTATATGTAGCTAAGTATCCAGTTGGAATTGATTCTAAACTAGAATATATTAAGCTTCGTTCACATAATATGTTTGAGAAGAACAACAAATTCCATTATCGGACACAACATGAGTATGAGTTTGATACTGGTATCTACATGGTGGGGATATATGGAATTGGAGGTATTGGTAAGACAACTTTGGCTAAAGCTTTATACAACAAAATTGCTAGCCAATTTGAAGGTTGTTGCTTTCTATCAAATGTTAGAGAAGCTTCAAAGCAATTTAATGGCCTTGCTCAACTACAAGAAAGCCTACTCTATGAGATCCTAATGGTTGATTTGAAGGTTGTCAACCTTGATAGAGGAATTAACATCATAAGGAATAGATTGTGTTCGAAGAAAGTTCTTATAGTTCTTGATGATGTAGATAAGCTTGAGCAATTAGAAGCATTGGTTGGTGGGTGTGATTGGTTTGGTAAAGGCAGTAGAATCATTGTGACCACAAGAAATAAACATTTACTTTTTAGCCATGGCTTTGATGAAATACACAATATTCTAGGATTGAACGAAGACAAAGCTATTGAGCTTTTTAGTTGGCATGCTTTCAAGAAAAATCGTCCATCAAGTAATTATTTAGACCTTTCAAAACGTGCTACAAGTTATTGTAAAGGCCATCCTTTAGCTCTCGTTGTTTTGGGTTCTTTCCTCTGCATTAGAGATCAAGCAGAATGGTGTAGTATATTAGATGAATTTGAAAACTCTTTGAACAAAGATATCAAAGACATTCTTCAATTAAGTTTTGATGGTCTCGAAGACAAAATAAAGGATATCTTTCTTGATATTTCTTGTTTACTTGTGGGTGAGAAAGTTGAGTACGTTAAAGATATGTTGGGTGCATGCCATGTAAATCTAGATTTTGGAGTTATAGTACTCATGGATCTTTCGCTTATTACGATTGAAAATGACAAAGTGCAAATGCACGATTTAATAAAACAGATGGGTCAGAAAATAGTTTGTGGTGAATCTCTTGAGCTTGGAAAGAGGAGTAGGTTGTGGTTGGTGCAGGATGTTTGGGAGGTGCTTGTTAATAATTCA

GTGAGTAACTCTTACCTAAAGTATCTTTAATTTACTTATTTCCAAGAGTTGACATGATGACGAAGGTTTGTCAAATGTTGTTAAATTACTAAACATATGACTTTTGCAG

GGAACAGACGCAATTAAAGCCATAAAGTTGGACTTTCCTAATCCTACAAGGTTAGGTGTGAATTCACAAGCTTTTAGAAAAATGAAAAATTTGAGATTGCTTATCGTTCAAAATGCAAGATTTTCTACCAAGATTGAGTATCTACCTGATAGCTTAAAGTGGATTAAGTGGCATGGATTTCCTCAACCAACTTTGCCTTCATGCTTCATTACGAAAAATCTTGTTGGACTAGATTTGCAGTATAGCTTCATGAAAACATTTGGGAAAAGACTTGAG

GTAAGTTTTATTTCTATATATGCCATAAATGGGTAGCTTACATTTTAAAATAATTCACAAATGCGCAAATCTATAATAATAGTTTTTTTTAATATAAAGTAAGGTGAGAGATAAGATATCTGAAGTTTATACGTTTGTGGAGTTCATATATGTTTAGGGAGTTTATATGTGTGGTGGAGAGTTGTTTTTTTTATGTTCATAGGCTTGTTTTAAGTTGTAGAGTTGAGTTCATAAGTTAAAGGTATTTTTGAATTATAAATAAGGAAAATTGTAAAAAATGACAAATTTGACAAAATATTTATAAAATATAGCAAAATTTTAGATTCTATCACGATAGACATTGATAGACACTAAATGTTTCTATTAGTGACATTGATGAACAGTAATAGAAGTCTATATCGATAGAATCCAAATTTTTGATATATTTTGTAAATATTTTACTATATTTGAAAATATCCTTATAAATAATATGTTTTTTATAACTAAATATTTTTATTTTTAAATTTATTTATCCAATAATTCTACTTATCTTTGTTTGAATAAAACACGAATTACATTTTTTTATTTAGTTTTTTTAGTTTTAATATTTTTTCTTTTTGATAATGAACAACTATTAACCCTATATTTTTGGCAGTAACACAGATAAATAAAATGTATAAAACAAGAGAGAAATCAAAACTTTTGATTGAGTAATTTTTCTTCATGAATTTCATCAATTTTCTTCTCTTCCAAGTTGGGGCCAACTATATTTTTCCTCTGTCACGATTTTTTTAATTGAATTTCCCTCATCATCTTTGCAACCAATCAAATGTCTCACAACATTTCTTCAACAATTTTGTATTCAATTCCTCTAAATTTGGAGGATAAAAAGAACATATCTCAATTTTATTAACTCTTGCTAGAAAATGTGTTAAAAAACAAATATTCATTTTATTAGTTTTTTTTTAGAAGTTTACGTAAATGTAACAAAAACAAAAAATATTTACGGCCCGTATAACAAAATAAGAAAAGCTCATGACGTGCAACAAACATGTTATCATAAATTTCATATATACCCTTGCGATTTATATCTTCTTTTATAATTTATTAATCTCGGGTCATCTTGGTGTAATTAATGCCCAAGATTGAATAACTAAAGGCAGAAAGTTTTTCAGCTCGGAGCATCTCAAGACATTGTATGCCCCACAAGTAAACAAGTTACATTTTAAACATTTCAAAACAAGTTGAGTGCACATAGTATCAAACTAAGAGTGTAGTTCTTTGTTTATATATATAGAAATATAACAACTTTCATTTGGACAAAAAATGAAAGAATATAAAGGCATCCAAAAAACCAAGCCACCAAAACACACCGACTAAAAGAAGGGGTCCAACGAACTAAAATGTTATCAACGGAATAAATATAAAAGGATGTAGAATTATCGAACCTAGCCAAAAGAGAAATGGTATCATATAATTTTTGGAAGAGGATAAATGATAAAGATGCGGAGATGAAGAACAAAAAGGATTTCCCCAAGACTTCCCCATCCATGAATTTTCACAAAAATACGCATCCTTACCCTACCCTAGAAAGCAACGAGTAAACTGAGAAAAAGTCGGAAGCTNNNNNNNNNNNNNNNNNNNNAAATCTGTTTTTTCTCTCGATAAG

CTTACTATCCTAAACCTCGCTGGTTGTTCTAACCTTAAAAAGCTTCCAAGAGGCTACTTCATTTTAAGGTCTCTTCGATATTTGAATCTCTCTCACTGCAAAAAGCTTGAGAAAATTCCAGATTTTTCTGCAGCATCAAACCTTGAAGAATTGTATCTTTTCAATTGCACAAATTTAAGAATGATAGATAAGTCTGTTTTTTCTCTTCATAAGCTTACTATCCTAAACCTTGATGTTTGTTCTAACCTTAAAAAGCTTCCAACGAGCTACTACAAGTTATGGTCTCTTCAATATTTGAATCTCTCTTATTGCAAAAAACTTGAGAAAATTCCAGACTTATCTGCAGCATCAAATCTTCAGAGCTTGTGTCTCCACGAATGTACAAATTTAAGACTGATTCATGAATCTGTTGGATCCTTGTATAAGCTTATTGACATGGACCTTAGCGGATGCACTAACCTTGCAAAGCTTCCGACCTATCTTAGGTTAAAGTCTCTTCGATATTTAGGACTTTCTGAGTGTTGTAAGCTTGAAAGCTTCCCATCAATTGCTGAAAATATGGAATCTTTAAGGGAATTGGATATGGATTTTACTGCCATAAAGGAGTTACCTTCATCAATTGGATATCTTACTCAGCTCTATAGGTTAAACCTCACCGGTTGCACAAACCTCATCTCCCTTCCCAATACAATTTATTTGTTAAGGAATCTTGACAAACTTCTTCTTAGTGGGTGTTCTAGATTTGAAATGTTTCCCCATAAATGGGACCCAACCATTCAACCAGTATGCTCTCCTTCAAAAATGATGGAAGCAACTTCGTGGAGCTTAGAATATCCCCATTTACTACCAAATGAAAGTTTGTGTTCCCATTTCACTTTGTTGGATCTTCAATCTTGCAACATATCAAATGCAAAATTTTTGGAAATTTTATGCGATGTTGCCCCTTTCTTATCTGATCTACGCTTGTCCGAAAACAAGTTCTCTAGTTTACCCTCATGCCTCCACAAGTTCATGTCCTTATGGAATCTTGAATTAAAGAATTGTAAGTTTCTTCAAGAAATTCCAAACCTTCCCCAAAACATACAAAATTTGGATGCCAGTGGTTGCAAATCGTTGGCTCGAAGTCCAGATAACATTATGGATATAATATCAATAAAACAG

GTTTGATTCTTTCCATTCATTTTGTTCCTATCTTTGCACGTAGACAATTTAATGCATATGAATTCTTATTCTCTATAG

GACCTCGCAATGGATGAGATTTCAAGAGAGTTCTTATTAACGGGCATTGAGATTCCAGAATGGTTCAGCTATAAGACTGCATCCAATTTGGCGAGTGCTAGCTTTCGTCACTATCAGGATATAGAAAGAACTTTGGCTGTCGGAGTTATTTTCAAAGTGAATGGAGATTCATCTGAAAGAGGGGTCCGAATTTCATGCAATATATTCATCTGCAACAAACTCCATTGTTCTTATTCAAGACCATTTCTTCCATCAAAATCAGAATATATGTGGTTACTAACAACTTCTCTAGCGTGGGGTTCCATGGAGGTGAATGATTGGAATAAAGTAATGGTATGGTTTGAGGTTCATGAAGTACATGGGGAGGTTAATGCAACTATAACAAGGTGTGGTGTCCATGTAACTGAAGAGCTCCCTGCGATACAAACAGATGCCAAGTGGCCGATGGTAAATTATGCTGATTTTTATCAACTGGAGAAATTGCAAAGTCT

GTAAGTTGATTGTTTACTTGTTACTTATTTTACTTTTTTTTTTGTAGTAAAAGTATGATAGATCTCAAAGGCGGAGATGTGTATGTTATGATTATGTGTTTGTCTAATGGCATAG

GGATATTGAGCATCTTCTTCTGAAACGGTTTTTTGAAGAAATGTCGTGCTGGTCAAATTGCAAAGCAATAATGTTCCATGCAGCAAATTATGATCCAGAAACAAACTCTGTATGCCACAGATTTAATTGGTCAGACGACATTGATTGGAGACAACCTTTAGATGATCCTATAAGCTTTTACTGGGTCCAAGAAAGACAGTACCGTTTCATGAGTTATTCGGGACTCGACAATCGTGGAGGTGGTGAAAAAGTGACCAATATTATCACAAATAACCACAGTACAATTTTATCCTCCAAAAGGTATTACATATTATACTTTAAAAATTTGGATGATAGAGTATATAGATTTTTAACTGCATGGGCTATAGCAAAGCCTCGTTGGATTGAGATTTACAGATATGATCATGATGTTGCAAGAAATTGTCATTTTGTCATTAAAAGGGTTGATCCATCCTTATGGCAACCCTGGGTTTGA

>MELO3C004266

ATGGATTCTTCCACTGTTGCAACAAAATCACCGACTTTCGGATGGAGTTATGATGTGTTTTTGAGTTTTAGAGGTGAGGATACTCGCACCAATTTCACCAGTCATCTTGATATGGCTTTGCGTCAAAAGGGTGTCAACGTCTTCATAGACGACAAGCTTGAAAGGGGTGAGCAAATTTCTGAAACTCTTTTCAAATCTATACAGGAAGCTTTAATTTCTATTGTTATATTCTCTCAAAATTATGCATCCTCTTCATGGTGTCTGGATGAATTGGTCAACATAATTGAGTGTAAGAAGTCCAAGGGCCAGATTGTTTTGCCAGTTTTCTATAAGGTGGATCCGTCGGATATTCGAACACAGACTGGTAGTTTCGGAGAAGCATTAGCCAAACATCAGGCTAAGTTCCAAATAAAGACCCAAATTTGGAGGGAAGCTTTAACTACTGCTGCTAACTTGTCTGGTTGGGATCTAGGAACTAG

GTATATATTTTCTTACCGACATTTTGTTTTTTCTATTCCAAATCTCATTTTTATGTTATGTTTGAATGTATATATGTATGTATATATGTACGTACGTATGTATGTGTATGTATGTATTTTAATGAGCACATTGACCAATTAATTTTAATCTTAGAAGATTCATTCTCTCTCAAGATCCAATTTAATTAATTGTGTTCATCATCTTTTAATTTTGGTATATGATTATGCACTTACCAACAG

GAAGGAGGCTAATCTTATTGGAGATCTTGTTAAAAAAGTGTTGTCTACATTAAATCGCACTTGCACGCCTTTATATGTAGCTAAGTATCCGGTTGCAATTGATTCTATACTAGAATATATGAAGCTTCGTTCACATATTAATCTTTTTGAGAAGAGCAACAAATTCCATTATCAGACACAACATGAGTATGAGTTTGATACTGATGTTAACATGGTGGGGATATATGGCTATAATTGGAGGTATTGGTAAGACAACTTTGGCTAAAGCTTTATACAACAAAATTGCTAGCCAATTTGAAGGTTGTTGCTTTCTATCAAATGTTAGAGAAGCTTCAAAGCAATTCAATGGCCTTGCTCAATTACAGGAAAGCCTACTCTATGAGATCCTAACGATTTATTTGAAAGTTGTCAATTTTGATAGAGGAATTAACATCATAAGGAATAGATTGTGTTCGAAGAAAGTTCTTATAGTTCTTGATGATGTAGATAAGCTTGAGCAGTTAGAAGCATTGGTTGGTGGGCGTGATTGGTTTGGCCAAGGCAGTAGAATCATTGTGACAACAAGGAATAAACATTTACTTTCTAGCCATGGCTTTGATGAAATACACAATATTCTAGGATTGAATGAAGAGAAAGCTATTGAGCTTTTTAGTTGGCATGCTTTCAAGAAAAATCATCCATCAAGTAATTATTTAAACCTTTCAAAACGTGCTACAAGTTATTGTAGAGGTCATCCTTTGGCTCTCGTTGTTTTAGGTTCTTTCCTTTGTACCAGAGATCAAGTAGAATGGTGTAGTATATTAGATGAATTTGAAAACTCTTTGAACAAAGATATCAAAGATATTCTTCAATTAAGTTTTGATGGGCTTGAAGACAAAGTAAAGCATATCTTTCTTGATATTTCTTGTTTACTTGTCGGAGAGAAAGTTAAGTACGTTAAGAATATGTTGAGTGCATGCCATGTAAATCTAGATTTTGGAATTATAGTACTCATGGATCTTTCACTTATGACGATTGAAAATGACAAAGTACAAATGCATGATTTAATCAAACAGATGGGTCATAAAATAGTTTGTGGTGAATCTCTTGAGCTTGGAAAGAGGAGTAGGTTGTGGTTGGTACAGGATGTTTGGGATGTGCTTGTTAATAATTCA

GTGAGTAACTCATACCCAAAGTATCTTTAATTTACTTATTTCTAAGACTTCAAATGATGACAAAGGTTTGTCAAATGTTGTTAATTTACTAAATATATGACTTTTTCAG

GGAACAGACGCAGTTAAAGCCATAAAGTTGGACTTTCCTAATCCCACAAAGTTGGATGTGGATCTACAAGCTTTTAGAAAAATGAAAAATTTGAGATTACTTATCGTTCAAAATGCAAGATTTTGTACCAAGATTGAGTACCTACCTGATAGCTTAAAGTGGATTAAGTGGCATGGATTTCCTCAATCAACTTTGCCTTCGTGCTTCATTACGAAAAATCTTGTCGGACTAGATTTGCAACACAGCTTCATAAAAACATTTGAGAAAAGACTTAAGGTAAATTTTATTTCTATGCCTTAA

>MELO3C004288

ATGAATCAAGCAGGTAGATCATCTTCTTCCTCATGTTTTAGATGGAGTTTCGATGTATTTTTAAGTTTTCGAGGGGAAGATACTCGATCCAACTTCACCAGTCATCTTAATATGACTTTACGTCAAAGAGGAATCAATGTCTTTATAGATAAAAAGCTTTCAAGAGGTGAAGAAATCTCTTCATCTCTTTTGGAAGCTATTGAGGAATCCAAAGTCTCAATCATTGTAATTTCCGAAAGTTATGCATCTTCGAGTTGGTGTTTGAATGAATTGGTGAAAATCATTATGTGTAACAAACTGAGAGGACAAGTTGTTTTACCAATTTTCTACAAAGTGGATCCATCTGAAGTAGGAAACCAAAGTGGAAGATTTGGAGAAGAATTTGCCAAACTTGAAGTTAGATTCTCGTCGGACAAGATGGAAGCATGGAAGGAGGCTCTCATTACTGTTTCTCATATGTCTGGATGGCCGGTTCTTCAAAGAGA

GTATTCTCTTTCTTCATATATATATACACTTCTACTTCTTCATATATATATATATACTTCTACTCTCTTGCTCTTCATATTCTATTGTTACTATTCTTAGCTTGTTGGTCAATGATAAAAAAGTAGTAGAAGAAGAGTGATATTATTTATTTTAGGTTCAGTTTGATAACATTTTTCGTTTTGTGCATCACACATTGTTCGAACAAAATTTGTTTTCAAAGTGGTACAACTACGTCTTTGAGTGAAAACAAAAACAAAACAAAATTGTTTTGCGTAATTTTCATTTTTAAAATTTATATGTCTTTTATCTTCTAATTTTTCAATTATGGTTTAAAAGAAAAATTCTAAAAACAAACAAAATAATGATTTTTAAAACTTAGTGTAAATTTTTTAAAATAGTAAACAAAATGGACAACAAATTATAGAAACTTATGGTGGGAAGGTAAGGTTTGAATACTTATTTTAAAATATTAAAAACCAAAAACCAAATAGTTATCAGGTAAGTCTTAGAGATTTTATTTTTCATTATCTTATTTATCATGTCCTTTTAATCAAAATTTTGTTAGAAATTTACACGAGATATAAATAGATGCAGAACAAACTAAATTTTATCTCAAACTCCCACTTCATAAGTTGAGAGCCAAATAAGCAGTTGATCATAATTTTTAATAATAAAACAAAGATAAAAATGAGAATGACACCGAAAATTTTATTGGTTCATTTCAAACTCAAAACTATATCCAAGTTTTGTGCAAGGTTCTACTATCATGGAGATTGAAAGAAAACCTTGAACACGTGTTTACAAATTTCTCTTTCCAAATAGACCAACAAAAGCTTATTTGATACCATTGACTCGATAACTTGAAATAGGGTAGTCCTTGACTCAAAATAGAAAAATACACACTGTAAGGATGTGCTTGTAGCCACTTAATTTTGTCTGACATTATTTAAAATCAATTTAATTGTTAGATTAATTTTTGCCATAATTTGGTTTATTTTTAATTTTGTGATTAACTACCTTTTTCTAAAAAAAAAAAATGTTATTTAACTACTTTTTAAGTTTTGTAGGTAGTTTGGTTAAAGTAACTAAGCCATTTAGTTAATTAAGGTAACTGTAGGTAGACAAATACCAAAGATGATAACTTTTTTTAATTGATTTTGGTTATTAAAGGCTCTATGCCATTTTGATATGAACGCTAAAGTTGGTATTATTTTCAAATGGAAAAAATCCCACCATTTTGAATTTTTAGCCATTTGAAGAAGTGATTTTGGCTATAAATAATATCTTCTTCTTCATACACACAACATCACATAATGAAAAGTTCACAAAAAAAAANNNNNNNNNNNNNNNNNNNNNNNNNNNNNNNNNNNNNNNNNNNNNNNNNNNNNNNNNNNNNNNNNNNNNNNNNNNNNNNNNNNNNNNNNNNNNNNNNNNNNNNNNNNNNNNNNNNNNNNNNNNNNNNNNNNNNNNNNNNNNNNNNNNNNNNNNNNNNNNNNNNNNNNNNNNCCTAGAAATTTTTACAACATTCACATCCTCTCTTCAAATCTAAATCTCTAACCTTCATAGCCCCTACTTGTAACCTTTATATCTTCTTACAAAAACCAAAAGATCTACAACATCCATTATTTTATCTTCTCTAAATCTTCATCTATCTTCCTACGATGCCAATAATTCAACACACAAAAAAATTCTAACTTAATTGGAGGTGTATGGTAGGGCTTTGGTTTCAATGATCCACACATCAAACAACAAGTGAATTTAGTGTGTTGGGATTTCAATCTCTCTCTAATTTCAATTTTTTCTTATTCTTCTTATTTTTTAATTTTTCTTTAAAGTTCTTTAATTTATTTTTTCTTAGTCTTATTATTCTCATTATTTTACTTATCTTTCTTCAAGCTACCATGAAAAATGCACAAAAAAAAAAAGAAAAAGAAAAGAAAGAAGAGAGAGAGCAATGATTAAATATTTGCTTATTATTTTTTGTTCTTATTTCTTTAGTCTCTTATAATTATTTATTTATTATCTTGTGATAGTTTTATTTATTTATTCATATGTTGTTATCTTTTATTTATTTATTATCATGTTTTTCCTTTATTAATTTATTCTTAATTTTTATTCTTTATATATATATATATATATAATTAGCATTTTTATTTATTTAATATTTTTCTCATCTTTATTATTGCTTCCTTAATATGTTCTTGACTTTTATATATTATTTAATATTGTATTTTCTTTCCTTCTTTTGTGATCAATTTAATATGTTATTGCTAATTTGTTTTTTAAAAAAATTGTTAATATTTTAAATTATTTTTCTTTAAAAACATCCAACGATGGAATATAGGAAATTTAGGAGTCCGATTTCTAAGAATTCTTGAGGTGAGAAAATCGATTCTTTGGAAGTTCGAGATTGATTTTCAATATGTTTTTATTAAAATCTCAATATGTTATTTTGTGTTTGCATAATTTGTCATGATGCTTCTTTACTCTTCTTTCTACTTATCTTATTTTCATATTAAGTCTTATTTTATTTCCTATTTAAAATTTTCAAGTTTTAAAATCCTTTCTAATTTAAAATTTTCCAAGTTTTAGAACCTTTTACAAAACACTTTTTTTTTTAAATCATTTAGAGTTTTTTCTTTTAAAGTTATAATTTTTCACGTTTTTCAAATGTTCAAAATTTAATCTATGATTTCATAAAATTTTTTAATCAATTTTTTAAAATAATTTATACTGTTTTTTTAAACAAAATCCTACGACGGAATTTTAGAAATTTGGGAGTTCGATTTTCTAGAATTCTTGAGGTGAGGGATCACATCTTTGGGAGTCCAAGATTTGTTAAAAAAAAATAAATTTTTAGTTAATGTTTTAAAAAAATCTATTTATTAGAAGGTTATTCCTACGATGGATTTTGAGAAATTGTACTAATTCCTCACTTTTTTGAGGTGAGATGATTTTCTTCAAACATTGTCTAATTAATGAAATGTTTTCCACTAGATCATTGTTCAAATATTGGAGTAACGAGGGGTAAAATAAGACATTAGTTTTTAAAGAAATTAAAGAATATTTTCAATTCAAAATTTGGAATTTGTTCACTGTTTTCTAAACGGATGTTGTGGGGTGCTAACACATTCCTCATACACAAATGACTCTCGAACTCAACTCTAGTATTTGCAGACCATTTTTTAATGATTTTATTTAAAAGGTTTTAATATATTTCGATGTCAAATCACACCATAAAAAAGATTGGTGATTACTCTTATTTTATTTTAAAAAACAAACCCATTTTGAGGACGTCGACCGTTGTGCGTCGTCTCGGGCACGTGGCGACAGTGCTTACTATCACTCTAATCACGACCCACAAATTTTTCTTTCATCAACAAAGAAATTGAGCTCGTCATACACCCACGCAAAATTTTCTTGCAAAATAAAGCTCACGGCGATGTCTTCCAATTTTGCTTCAAATCTTTGCTTTTTATAAAGAAATCCACCATATATCCACAATAACCAATTAACTTATTTCATTATAATCCACCTTCATGCAAACCATTCAAATGGTTCCTCATGCTTTAAATATTCAAATGGGGCTTGGTGTTTTGGGTTCCAAATTTCCTAACATATTTTTTGGTTGGATTTTGTTTGAGTTTATTATATTGTTGCTTTGATTTTTTGGCTTCTAATTTTGAGAACTATTTGAGGAAACAAATGTGTATTTCAAACAAAAATTTAGTGTAGTTTTTTTTCTTAAATGACAG

TGACGAGGCTAATTTGATTCAAAACATTGTTCAAGAAGTTTGGAAGGAATTAGATCGTGCAACGATGCAGTTGGATGTAGCTAAATATCCAGTTGGAATTGACATACAAGTTAGGAATTTACTCCCACATGTGATGTCTAATGGAACTACTATGGTTGGATTATATGGAATTGGAGGTATGGGCAAGACAACTTTGGCCAAAGCTTTATATAATAAAATTGCTGATGACTTTGAAGGTTGTTGCTTTCTGCCAAATATTAGAGAAGCTTCGAATCAATATGGGGGCCTTGTTCAACTTCAAAGGGAGCTACTTCGTGAGATTCTAGTGGATGATTCGATCAAAGTTAGCAATCTTCCTAGAGGAGTTACCATCATAAGAAATCGACTATACTCAAAAAAGATTCTTTTGATTCTTGATGATGTTGATACACGTGAACAACTACAGGCATTGGTGGGAGGACATGATTGGTTTGGACATGGAAGTAAGGTGATTGCGACAACAAGAAACAAGCAATTACTTGTTACTCATGGATTTGATAAAATGCAAAGTGTTGTTGGATTAGATTATGACGAAGCTCTTGAGCTTTTTAGCTGGCATTGTTTTAGGAATAGTCATCCCTTAAATGATTATTTGGAACTTTCAAAACGTGCAGTCGATTATTGTAAAGGTCTTCCCTTAGCTCTTGAAGTTTTAGGTTCCTTCCTTCATTCTATTGATGATCCCTTCAATTTCAAACGTATTTTGGATGAGTATGAAAAATATTACCTTGACAAAGAGATTCAAGACTCTCTTCGAATAAGTTACGATGGACTCGAAGATGAA

GTAAAAGAAATATTTTGTTATATTTCTTGTTGCTTTGTACGAGAAGATATCAACAAAGTTAAAATGATGTTAGAAGCATGTGGTTGTATATGTTTGGAAAAG

GGAATAACTAAACTTATGAATCTATCACTTCTTACCATTGGTAGATTCAACAGAGTTGAAATGCATGACATAATACAACAAATGGGTCGAACAATTCATCTTTCAGAAACTTCTAAATCTCATAAAAGAAAAAGATTGTTGATTAAAGATGATGCTATGAATGTCTTAAAGGGGAATAAG

GTAAGAATTATCGGTCAAACATCATCATTTTTATTATTTTGAAAATTAATGACTTGAAATGTTTGTTTTGCAACCTTTTGTAG

GAAGCAAGAGCAGTCAAAGTCATAAAATTTAATTTTCCTAAACCTACGGAGTTGGATATTGATTCAAGAGCTTTTGAAAAAGTGAAAAATTTGGTAGTACTCGAAGTTGGCAATGCCACATCTTCAAAAAGCACTACTCTTGAGTATCTACCTAGTAGTTTAAGGTGGATGAATTGGCCTCAATTCCCTTTTTCATCTTTGCCTCCAACCTACACAATGGAGAACCTTGTTGAATTGAAATTACCATATAGCTCCATCAAACATTTTGGGCAAGGATATATG

GTATTTATAATATAAATTATTGCATATGTTTTTAATTTCTATGTTGAAATTTGATTGTTTACTTTCTTCTTATATATTCATTGCAG

AGTTGTGAAAGGTTGAAGGAAATTAATCTTACCGACTCCAATTTTTTGGTGGAAATTCCAGATTTATCTACCGCAATAAACCTCAAATACTTGGACCTCGTAGGATGTGAAAATTTAGTAAAGGTTCATGAATCAATTGGATCTCTCAATAAACTTGTCGCACTTCATCTTTCTAGCAGTGTTAAGGGCTTTGAACAGTTTCCATCGCACCTCAAGTTGAAATCTCTTAAGTTTTTGTCAATGAAAAATTGTAGAATAGATGAATGGTGTCCTCAATTCAGTGAAGAAATGAAGTCTATAGAATATTTGTCCATTGGGTATAGTATTGTAACACATCAGCTATCTCCAACAATTGGATATCTTACTAGCCTAAAACATTTGACCCTCTATTATTGCAAAGAGCTTACAACCCTTCCAAGTACAATTTATCGTTTAAGCAATCTTACTTCTCTAATTGTGTTGGATTCTGATCTTTCAACATTTCCTTCCTTAAATCATCCGTCTTTACCTTCCTCACTTTTTTACCTAACCAAGTTACGTCTTGTAGGTTGCAAGATAACAAATTTGGATTTCTTAGAAACAATTGTTTATGTTGCCCCTTCATTGAAAGAGTTGGACTTGTCCGAAAACAACTTTTGTAGACTACCCTCGTGTATTATTAATTTTAAATCCCTGAAATATCTTTATACAATGGATTGTGAGTTGCTCGAAGAAATTTCAAAGGTTCCAGAAGGTGTAATTTGTACGAGTGCCGCAGGGTGCAAATCATTGGCTAGATTTCCCGACAACTTAGCTGATTTCATATCTTGTGGTAATTCTGCG

GTGCGTACCATATCTCTTTCTCATGACTTCACCATTATCTCTAGCTCATGTATATTTAATTTCATTCATATAATATATATTACTTATAACTATTTACTGATCTCATGGTGCAG

GAATGTTGTAAAGGTGGAGAATTGAAACAACTGGTATTAATGAATTGTGATATTCCAGACTGGTATAGGTACAAGAGTATGAACGATTCATTAACATTCTTTTTGCCAGCTGATTATCCAAGTTGGAAATGGAAGGCTTTGTTTGCTCCTTGCGTCAAATTTGAAGTTACTAATGATGATTGGTTCCAGAAGCTTGAATGTAAAGTGTTTATCAACGACATTCAAGTATGGAGTTCTGAAGAGGTGTATCCCAATCAGAAGGAACGAAGCGGGATGTTTGGAAAAGTATCACCAGGTGAGTACATGTGGCTGATAGTACTTGATCCTCATACACATTTCCAATCATATTCCGATGATATTATGGACAGGAGGTCACCGAAGATTATTGATCTAAATCAACCAAGTTTTGGGATTAATTCCTCACAAAGTATTTTGGGTAAAATTACGGTGTCCTTTCAGGTTACTCCATGGTATAAAGACGTTGTAAGTATAAAAATGTGTGGTGTTCATGTCATCATGTGGGAATGA

>MELO3C004289

ATGGGTTCTACCGCTGCTGGAGCCGAATCGTCGTCTTCTTCTCCCATTTTCAATTGGAGTTATGATGTGTTTTTGAGTTTTAGAGGAGAAGATACTCGCTCCAATTTCACCGGTCATCTTTACATGTTCTTGCGTCAAAAGGGTGTCAATGTTTTCATAGATGACGGGCTCGAAAGGGGTGAGCAAATTTCTGAAACCCTTTTCAAAACTATACAGAATTCTTTGATTTCTATTGTTATATTCTCTGAAAATTATGCATCTTCTACGTGGTGTTTGGATGAATTGGTGGAAATAATGGAGTGTAAGAAATCAAAGGGCCAAAAGGTTTTGCCAATTTTCTACAAGGTAGATCCTTCGGATGTACGAAAACAAAATGGTTGGTTTAGAGAAGGATTGGCCAAACATGAGGCTAATTTCATGGAGAAGATTCCAATATGGAGGGATGCTTTAACTACTGCTGCCAACTTATCTGGTTGGCATCTGGGAGCAAG

GTACATGTTTTTACTGTTATTTGTTCTTTTCTTCTACAAATCCCATTTTAAATATATTCTGTTTTTTTTTTTAAAAAAACAAAATAGTATTATTTCAATTGGGGTTTCTACTTTGCATATGTGGAGGGATGTTGGATTTGGGCTTCTACTTCTGTTTTTCTATCATTTTGTACCATTGTAAACAAGTTGATTAAATATGTCGTCTATGCATTTTGAGGATTTAATGATCATTTTTTAATAAGGGAATGGTTATATTGAGGTATGCAAGATTTTTTCTTTTTTTTTTTTTTTTGCTTTATTTTTAAAATATATACGTAGTATTAATAGTCCTCTCTTTGTCGTATGTTTGTTTCTTAGTGTTTTATAAATCTAAACAGTTTTTTGAAAAGAGAGAGAACTCAGGAATACTGTATGACTCATAGACAAGTAAAAGTCATGGACTAGGACCATGAGTTCTGTTATTTTTGTTGTTGAACGCATCGATGCTCACAAGGAATAGAGATTTGAATTATGGAAACTGTGGACCTCGTTCACTATTTTTCACAACTATTTTTGTCAATATTTTTTGCTTTCCTATATTTTTATCATTTGTTTATACTTTCACTTTATTCAAGGACGCTATCTGTTGCCATTTATTTCATTTATTAGAAATTATCACATTGTATAATTTAATCTCAGAAGATTTACTTTCTCAAGATGCAATTTAATTAGCTATGTTCTCTCATCATCCATTAATTTTGCAATGATTGTGTACTAATTAACAACAG

AAAGGAGGCTCATCTTATTCAAGACATTGTTAAAGAAGTGTTGTCTATATTAAATCACACCAAGCCCTTAAACGCAAACGAGCATCTAGTTGGAATTGATTCCAAAATAGAATTCCTTTATCGGAAAGAAGAAATGTACAAGTCTGAATGTGTTAACATGTTGGGGATATATGGCATTGGAGGCATTGGTAAAACAACTTTGGCTAAAGCTTTATACGACAAAATGGCTAGTCAATTTGAAGGTTGCTGCTATCTACGAGATGTTAGAGAAGCTTCAAAGTTATTCGATGGCCTTACTCAACTACAGAAAAAGCTACTTTTTCAGATCTTAAAGTATGATTTGGAGGTTGTCGATCTTGACTGGGGAATTAATATCATAAAGAATAGACTGCGTTCAAAGAAAGTTCTTATACTTCTTGATGATGTGGATAAGCTCGAGCAATTACAAGCATTGGTTGGTGGGCATGATTGGTTTGGTCAGGGTACTAAAATCATTGTGACGACTAGAAATAAACAATTACTTGTTAGCCATGGATTTGATAAAATGTACGAAGTTCAAGGATTGAGTAAACATGAAGCTATTGAGCTTTTTCGTCGGCACGCTTTTAAAAATCTTCAACCATCGAGTAATTATTTAGACCTTTCAGAGCGTGCTACAAGGTATTGTACAGGCCATCCTTTGGCTCTCATTGTTTTGGGTTCTTTCCTTTGTGACAGATCAGATCTAGCAGAATGGAGTGGTATATTAGATGGATTTGAAAACTCTTTGAGAAAAGATATTAAAGATATTCTTCAATTAAGTTTTGATGGGCTCGAAGACGAAGTAAAGGAGATTTTTCTTGATATTTCTTGTTTACTTGTGGGCAAGAGAGTTAGCTACGTTAAGAAAATGTTGAGTGAATGCCATTCGATTCTGGATTTCGGAATTACAAAACTCAAGGATCTTTCACTTATTAGATTTGAAGATGATAGGGTGCAAATGCATGATTTAATAAAACAAATGGGTCATAAAATAGTTCATGACGAATCTCATGATCAGCCTGGAAAAAGGAGTAGATTATGGTTGGAGAAGGACATTTTGGAGGTGTTTAGTAACAATTCA

GTAAGTTACTCTTACCTAAATTAATTTATCTTTAATTTAATTAATTGCTAGACTTTCACGTGATGAAAAATGTTTGTCAAAGTAATGTTCAAATTACTAAATCTATGACTTTGTAG

GGAAGCGATGCAGTGAAAGCCATAAAGTTAGTATTAACTGATCCCAAAAGGGTCATAGATTTGGATCCAGAAGCATTTAGAAGCATGAAAAATTTGAGAATACTTATGGTCGATGGAAATGTAAGGTTTTGTAAAAAGATTAAGTATCTACCAAATGGGTTAAAGTGGATAAAGTGGCATAGATTTGCTCATCCATCTTTACCCTCATGCTTTATTACAAAAGATCTTGTTGGACTGGATTTGCAACATAGCTTCATCACAAATTTTGGAAAAGGACTTCAG

GTAATATATTGCTCTACCTCATCTACATTTTGATTTGAAGCCTTTGCCTTCCATTTCTTAGAACATTAAAATGGGTAGCATACTTTTTTAAGAAATTAAATTCTTTGTGCTCTTTCTTTCTTTCTTTTTTTTTTTTTTCAAATATAACTTTGCGTAAAAAAATTTATAACTTTTTAGAAGTTACTAAATCCTTTTTAAATTGAATAATAGTATTTTATCTAAAGAATCTTCTCAAATATAGTAAAATTTTATATTATCAATTATGGATGTTGTCAAAACATTAATAGAAGTTTATCCGTATCCATTCTAAATTTTGTCATATTTCGTAAATATTTTTGTTCATATTTGACTATATTTGAAAACAACTCGTATAATTAAATTTAGTTGGGCTACATTTGTACTGCCTGTTTTCTTCCATTTCATTGAATAATTATTTAGTTTTTGTTTATATATACAAATCGGTAAATTTAAACATATTCATTTAACTATCAGACATTATTTATCCTAATTATTCTGCATATCCTAATTTGTATTTGTTTTTTTTATAATATTTTATTTTGATTATATTTGTATCAGTATTTACTCCTTTTCTTTTTTGTTTTTCTTTCCATGTGTATTTAG

AATTGTATGAGGTTGAAGTTGCTTGATCTTAGACACTCAGTTATTTTAAAGAAAATTTCTGAGTCCTCTGCAGCACCAAACCTTGAAGAATTGTATCTTAGCAACTGCTCAAATTTAAAAACGATTCCCAAGTCATTTCTTTCTCTTCGTAAGCTTGTTACCCTGGACCTCCATCATTGTGTAAACCTTAAAAAGATTCCAAGAAGCTACATTTCATGGGAGGCTCTTGAAGATTTAGATCTTTCTCACTGCAAAAAGCTTGAGAAAATTCCTGACATCTCTTCCGCATCAAACCTTAGAAGCTTGTCCTTCGAACAATGCACAAATTTAGTAATGATTCATGATTCTATTGGATCTCTGACTAAGCTTGTTACCTTGAAACTTCAAAACTGCAGTAACCTTAAAAAGCTTCCAAGGTACATTTCATGGAACTTTCTTCAAGATTTGAACCTTTCTTGGTGCAAAAAGCTTGAGGAAATTCCTGACTTCTCTTCTACATCAAACCTTAAACACTTGTCTCTCGAACAATGCACGAGTTTAAGAGTGGTTCACGATTCTATTGGATCTTTGAGTAAGCTTGTTTCCTTGAACCTTGAAAAATGCTCCAACCTTGAAAAGCTTCCAAGCTACCTCAAGTTAAAGTCTCTTCAAAATTTAACACTCTCTGGTTGCTGTAAGCTCGAAACGTTTCCAGAAATTGATGAAAACATGAAATCCTTATACATATTGCGCTTGGATTCTACTGCCATAAGGGAGCTACCTCCGTCAATTGGATACCTTACTCATCTTTATATGTTTGATCTTAAAGGTTGCACAAACCTCATCTCCCTTCCTTGTACAACTCATTTGTTAAAGAGTCTTGGCGAGCTTCATCTTTCTGGGTCTTCTAGGTTTGAAATGTTTTCCTACATATGGGACCCAACCATCAACCCAGTATGCTCTTCTTCAAAAATTATGGAAACTTCATTGACTTCCGAGTTTTTCCATTCACGAGTTCCAAAAGAAAGCTTATGTTTCAAACACTTCACATTGTTGGATCTTGAAGGTTGCAATATATCAAATGTTGATTTTTTGGAAATTTTATGTAATGTAGCATCTTCCTTATCTAGTATACTCTTGTCGGAAAACAACTTCTCTAGTCTACCTTCATGTCTCCATAAGTTTATGTCCTTGCGGAATCTCGAATTAAGGAATTGCAAGTTTCTTCAAGAAATTCCAAACCTCCCTCTGTGTATACAAAGAGTAGATGCCACTGGTTGCGTATCGTTGAGTAGAAGTCCAAACAACATTCTGGACATAATATCAAGCCAGCAGGTTCATCTCCTACCTATTCATTTTCCTTGTTCATATCAAATTCTTGTCATTTACACTAACATTCCATGCATAATAATTCTTATGCCATAGATCAATTTTGCATGGTTAAGGAATCGTCCTCGTGGCATAAGGGAGTTCGTTCTAATGAATAATGGGATTCCAGAATGGTTTAGCTATCAGATTGCATCAAATGCAATAATGGTTACTTTTCAACACAATCGCGATACAAAAATAACTTTGGCTACATCTGTTACTTTCCGAGTGGATGGAGATTCAGATCAAGGAATGGCCTTAGTTTCATGTAACATACTCATCGGCTGTAGACTCGACCGTCGTTATATGAGAAAATTTCCAAAATCAGCATCAGAATATACATGGTTAGTAGAAACTTCTGCAACATATCGTAGG

>MELO3C004290

ATGGCTTTGCGTCAAAGAGGAATCAATGTTTTTATAGATAATAAGCTTTCAAGGGGTGAAGAAATTTCTACATCTCTTTTGAAAGCTATTGAAGAATCCAAGATCTCCATTGTTATAATCTCTGAAAATTATGCATCTTCAAGTTGGTGTTTGAATGAATTGGTGAAAATCATTACGTGTAACAAATTGAGAGGACAAGTCGTTTTACCAATTTTCTACAAAGTGGATCCATCTCAGGTAGGAAAACAAAGTGGAAGATTTGGAGAAGAATTTGGGAAACTTGAAGTTAGATTCTCGTGGGACAAGATGGAAGCATGGAGGGAGGCCATGATTTCTGTTTCTCATATATCTGGATGGACGGTTCTTCAAAAAGA

GTATTTTTCTTTTTCATATATATCTTTCTAACTCTTTAGCTCTTCATATTCTATATATACTTTGACTATCTCAAGGTGAGAGCATGGTGGACTAAGAGCAAATGCAAAGAAAATGTGACATTTTAAGTTCCCATTCGATGACAATTTTATTTCCGAGTTTTTCATTTTTTAAATTTATATACTCTTGTTGTTTATCAAGGAAACATTTGAATTTTTAACTTTATATAGTTTTACAGAAAAAACAGAATTTGAAAATTATATAATATAATTGTTTGAAAATATTTACATTATAACAAAATTTTGTTGCGGTAGACTCGAGTTTATCTTTTTATAAAAAGTAAAATTTTACTACATTTGTAGATAACTTAGCTCATTATTTTATATTAATTAAGAACACATTACAGCTTCCGAGACTTATAAGTTGACTTAATTTTTAAAAATATTAGAAGAAGGCAGTAATTAACAAATCATAAAATCCCATGGGTGAAAATAATGGCATATGTACTTAATTAATTTTAAAAAACAAAAATTCAAATACCAAAAGTTATTTATACAAAGGCATTAGAATTGATTTTTGTAAAATTGGAATTATTTCATAATATTATTTAATTTGTTATTATTGTTCTTTCTCCAAATAGATATTACTTTAAAAGTTGGATTTAGTTTGACTTTATAATGCTGCTTTCACTTTCCCTGGCTTCTAATATTATAGGAATTATAAGTGAATTTCAAAACAACTTTTAGTGTATGTTTCTTTCTTAAATGGCAG

AGACGAGGCGAATTTGATTCAAAAAATTGTTCAAGAAGTCTCGAAGAGATTAAATCGTGGAGCAATACAATTGCGTATAGCTAAATATCCAATTGGAATAGACAGACAAATTAATAATATACTCTTTCAAGTTACGTCTGATGAAAAAATTACTATGGTTGGATTTTATGGAATTGGAGGTATTGGGAAGACAACTTTGGCCAAAGCATTATACAATAAAATTGCTAATGACTTTGAAGGATGTTGCTTTTTGGCAAATGTTAGAGAAGCTTCAAATCAATACCGGGGTCTTGTTGAACTCCAAAAAGAGCTACTTCGTGAGATTCTAATGGATGATTTAATCAAATTTAGTAATCTTGATGTAGGAATTAGCATCATAAGAGATCGACTATGCTCAAAAAAGATTCTTTTGATTCTTGATGATGTTGATACAAGTGAACAACTAGAAGCATTAGTGGGAGAACATGATTCATTTGGACCAGGAAGTATGGTCATTGTGACAACAAGAAACAAACATGTACTTGTTATTCATGAATTTGATATATTGCAAAGTGTTCAGGGATTGAAGGATGATGAAGCCCTCAAGCTTTTTAGCTGGCATGCTTTTAAGCAGAGTTGTCCATCAAGTGATTATTTAGACCTTTCAAAACGTGCCGTACGTTATTGTGATGGTCTTCCCTTGGCTCTTGAAGTTGTAGGTTCATTCCTTCACTCCATCGAACAATCCAAATTTAAACTTATATTGGATGAATATGAAAACCAATACCTTGACAAGGGCATACAAGATCTTCTTCGAATAAGTTACGATGGACTTGAAGATGAAGTAAAAGAAATTTTTCTTTATATANNNNNNNNNNNNNNNNNNNNGATATTGAATTTACTTTTATTCTTTTCTTATTTAAACATAGAAAGAAAGAAAAAAATGTATAAGGAAAATTTCATTTTTTTTTTTTGCTACCTTTTTATTGTTATTCTTTTTAAAAAAAAAAAAAAGCATTTTAACATTTTTCTTCTCTCTGTTAAAAAGAGAGAAAAAGAAAAAAGTTATAAGCAAAAAGTCTTATATTATTATTATTTTTATATTTATTATATATATAGTTTTTTCTTTTTCTTTGCCGTCATCCCTCTGTTTTCTTTTTTTTTTTAA

>MELO3C00429

ATGGTTGGATTATATGGAATTGGAGGTATCGGCAAGACAACTTTGGCCAAAGCATTGTACAATAGGATTGCAGATGACTTTGAAGGTTGTTGCTTTTTGCCAAAAATTAGAGAAGCTTCAAATCAATATGATGGCCTTGTTCAACTCCAAAAGAAGCTACTTTGTGAGATTCTAATGGATAATTCGATCAATATTAACAATCTTGATATAGGGATTAACATCATAAGGAATCGACTATGCTCAAAAAAGATTCTTTTGATTCTTGATGATGTTGATACGAGAGAACAACTAGAAGCATTAGCGGGAGGACATGATTGGTTTGGACACGGAAGTAAGGTCATTGCGACAACAAGAAACAAACAATTACTTGCTAGTCATGGATTTAATAAATTGGAAAAAGTTAACGAATTGAATGTCATTGAAGGCCTTGAACTTTTTAGTTGGCATGCATTTAGAAATAGTCATCCCTCAAGTGATTATTTAGACCTTTCAAAACGTGTCGTACGTTATTGTGATGGTCTTCCCTTGGCTCTTGAAGTTGTAGGTTCCTTCCTTTACTCTATCGAACAATCCAAGTTTAAACTTATATTGGATGAATATGAAACTCAATATCTTGACAAGGGCATACAAGATCCTCTTCAAATAAGCTATGATGGACTTGAAGACGAAGTGAAAGAAATTTTTCTTTATATATCTTGTTGCTTTGTAGGAGAAGATATCAACGAAGTTAAAAAGAAGTTAAAAGCATGTGGTTGTTTATGTCTGGAAAAGGGAACTACAAAACTCATGAATCTATCACTTCTTACCCTCGATGATTTCAACCAGGTTGAAATGCATGATTTAATACAACAAATGGGTCGCACAATTCATCTCTTGGAGACTTCTACATCTCATAAAAGAAAAAGATTGTTGATTAATGATGATGCTATGGATGTGTTAAATGGCAATAAG

GTAAGAACCATGGAGCAAAACTAATTTTTATATATGCTTATATTTATTTATTATGAGAACAACTTTTATTATTTTTCAATGTTTGTTTTGTAACCTGTAATTTTGT

AGGAAGCAAGAGCAGTTAAAGTCATAAAATTAAATTTTCCTAAACCTACGGAGTTGGACATTGATTCAAGAGCTTTTGAAAAAGTGAAAAATTTGGTAGTACTGAAAGAATGCTACATCTTCAAAAAGTATTGATGTGGAGTATGTACCTAATAGCTTAAGGTGGATTAATTGGCCTCATTTTCCTTTTTCATCTTTGCCTTCAACCTACACAATGGATAACCTTATGGAATTGAAATTGCCATATAGCTCCATCAAACATTTTGGGAAAGCATTCATG

GTATTACTTTTTCTCAATAATATTTGAGTTATATTAATTTAATTACAATGTCGGCTTATTTATTTGAAATTTAATTATATTTTCCTTTTATATTAATGGCAG

TGTGGTGAATGGTTGAAGGAAATTGATCTTAGTTTTTCTGAGTTTTTGGTGGAAATTCCTAATTTAACTGCTGCAATAAACCTTGAAATGTTGGATCTTCAAGGGTGTATAAATTTAGTAAAAATTCACGAATCTGTTGGATCTCTCAGTAAGCTTGTCGAGTTTTATCTTTCTAGTAATATTAAGGGCTTTGAGCAGTTTCCATCGTGCCTCAAGTTGGAATCCCTTTACACTTTGACATTGTACAGTTGTAGAATAGATGAACGATGTCCTCAATTTAGTGAAGAAATGAATAGCCTAGAACTATTATGGATTAAAGATAGTGTTGTAATTAATCAGCTATCTCCAACAATTGAATATCTTACTAGCCTACAACAATTGTGGATCATAAACTGCATGGGGCTCAAAAGTCTTCCAAGTACAATTCATCATTTACGTAATCTTACACTTTTATATGTCGATAGATCTGATCTTTCAACATTTCCTTCCTTAAATAATCCTTCTTCACCTTCCTTATTTCGCTGCCTACTTAACTTAACATCAATAACCCTTTGCCATTGTAAAATAAAAAATTTGGATTTCTTAGAAACAATGGTTCATGTTGTCCCTTTTTTGAGACAGTTGAATTTATCTGAAAACAACTTTTGTAGACTACCCTCATGTATTACTAAATTTAAATCCTTGAGATATCTTTATACAAGGGATTGTAAGTTGCTTGAAGAAATTCCAAAGGTTCCAAAAGGAGCAGTTACTATGAATGCTTCAGGGTGCATATTATTGGCCAGATTTCCTGACAACATACTTGATTTCATATCTTGTTATGATAATTATATGGTAGGTATCATTTCTTTCTCATGA

>MELO3C004292

ATGTATCGAGCAAGTGGATCGTCTTCCTCGCATGTTAGGTTGCCTTTTGATGTATTCTTAAATTTCAGAGGAGAAGATACTCGTTCCAGCTTCACGAGTCATCTTCATATGGCTTTGTGTCAAAAAGGTGTCAAAGTTTTTATAGATGATGACAAGCTTCCAAGGGGTGAAGAAATTTGTACATCTCTTTTGAAAGCCATTGAAGAATCAAAAATCTCCATTGTTATAATTTCAGAAAATTATGCATCTTCCCATTGGTGTTTGGATGAACTAATAAAAATCATCATGTGTAACAAATCCAATAATCGGCAAGTCGTTTTTCCTGTTTTTTACAAAGTGGATCCATCTCAAGTACGACAACAAAGTGGAAGATTTGGAGAAGAATTTGGCAAACTTCAAGTTAGGTTCTCCAACAAGATGCAAGCATGGAGTGAGGCTCTAACTTTCATCTCCAGTATGTCTGGATGGGATCTAAAAAATTA

GTATCTTTCTATATCCTTTTCTATTTCGTTTTCTTTTTTCGCTCTCCTGATCCATCTGTATTCTACCTATTTTGTTATTCGTAGAAGAGCATGTAGGCACGTGAAAAATAGAAGGAAATTGTTTTAATGGATAAAATTGCTCAAAAAATATATAAATTATATAGCAAAATTTCATAGTTTTTCGCAGTGATATACATATAATAGTGATTAATTATAGTAGAGACTATTACTGTCTATTAGCATCTATTAGACATATTTTGTTATTTTTGTAAATATTTTGGTTCGTTCTACTACATTTAACAACAATCCTAAATTTAGGTGCTCGATTTCATTTTTTAGCTTTTTTGTTAAATAATTTACATAATTTTAACAAAATACAAAAATATTTATGATCTGTATAACTAAAAATAAAAGCCCATGACTCCACCATTGTATTTTCATAAATTTCAGACTTGCCCTTGAATATTGCGAATGATTCGTCACTTCTCTTTCTTCTTCTTCTTTGCGATTTATTCATTGTCATTTGTGATTTTTTTCATCTTCTCTTCTAGATTTTTTTCTTCTATTTTTAAACTATTTACTCTTCTTTTTAAATCTCCTATTTCAATTTCATCATTTTCGGTAAGATTCTTTGAAGCTATTTCACGATTGTGTCAATGTTCTTCTTCATCTTCCTCATCATCGAGAATATCAAGATCGTTTAAATATCTTTTTAGATGATCAATTCTATCGTTTACTATGGTCAACACAATCGTTTACCTTGGTTCAACACGATCGTTTAATAATTGAAATCTTTTCTCATCATTTAAAAGGAAATACTGATTGTGTATATATAATGATCTAAACGATCGCTTATTACAATCATCACAATCGTTTAGAATGGTCAACACAATCGTTTACTATGGTCAACACGATTGTTAGATTTGAAGTTTTTAACAAACATTTACACTAGACTACACGATCGTTTGCCATGGTGAACATGATCGTTTAAATTTGAAATCTTTTTTTCCATTGTAAAAAAAAAAAAAACTACACAATCATGTATCATGATCTAAATGATCGTAAATCATTCATTTAGACTGTAATACACGATTGTTAGGAAGAAAGATTACTCACGCACGCATGTGGTCGATTAATTGCATGTTGACTGTGGTATTTTTGGTATTTTCCATTATGAGCTTGTGGACTTTTTCTATTTTTCAAATTGTTCTATACGGTGTAAAATATCTTACTGGTTTGTTATATTTTTGAAAAAACCTTTTTTAATGTTTTTAAATTTATATTTATTATTTTTTAAAAAATTGTAAATATAATTTCTACCTTATTTAAAGAAAACTTGAATTTCTAGTTGAATTCAAAAAATGAAAACAAGTTCTTCAAAATTATTATTATATTATTTTTTAAGAAAACTTTGTCTTAATATATAAAAATATTAGAATAAAATGAATAACAAATCATAAAAATTATTTATGGATGGAGGTAACAATATTTAAGGTGAGTTGCATAAATGACATTTAAGCAAAAAAAAAAAAAAAAAATTGTAGAATACATAACACAAGGATAAGATAATGGAAAATTGCATTAGATGATAAAAAAATCTATTAAAAAAAAAAAAAAAAAAAAACAGTCCATAGAACTTTTGTTTTTCCATATCTCCATCAGTGATAGACCATAAATGATAATCTATTACATATTGTTAATATGAGTCTATCAATGATACTACTTGTAAGAGTATATTAATGATAGACTATATTGTTGGTAGGAGTCGAGCATTGGTAATTTTGTAAAAATAATGTTACAGTTACCCGTTAATCGTAGGAGTCTATCATTGGTTAATTACCGTTGTTATACAATTTAAATTCTAATCCCCCATGAAACGTTGCAATTATTGTCTTTCAATTTATTTCATTTGTCTTTTCACCACACCTTTTAATGGATAGAAGTAGCGTTGGATTTAATTTGATTTAGTTTATTTGGTCGTTTTCCTTATCTCTTCTGCTTCTCAGTTTAAGAAATGTTGTTGAGAAAGAAAAGTCAATTTCATTCCTCTGTCACGTATGTTTAAAATTTTTCTTTAAAATGTCAG

TGAAAGTGAAGCAAGTTTGATTCAAATAATTGTTCAAGAAGTCAGGAAGAAATTAAAGAATAGCGGAACGACACAGTTAGATGTAGCTAAATATCCAGTTGGAATTAACATACAAGTTAATAATTTACTCCTACATGTTATGCCCAATGGAGTTACTATGGTTGGATTGTATGGAATTGGAGGTATGGGCAAGACAACTTTGGCCAAAGCTTTATACAATAGAATTTCTGATGACTTTGAAGGTTGTTGCTTTTTGGCAAATGTTAGAGAAGCTTCAAATCAACATTGGGGTCTTGTTGAACTCCAAAAGGCGCTACTTCGTAAGATTCTAATGGATGATTCAATCAAAATTAGCAATATTGGTATAGGAATTAGCACCATAAGGGATCTATTATGCTCAAAAAAGATTCTTTTGGTTCTTGATGATGTTGATACGCATGAACAACTACAGGCATTGGCTGGAGGACATCATTGGTTTGGACATGGAAGTAAGGTCATTGCGACAACAAGAAACAAGCAATTACTTGCTAGTCATGGATTTAATATATTGAGAAGAGTTAACGGATTAAATGCCATTGAAGGTCTTGAGCTTTTTAGTTGGCATGCATTTAAAAATAGTCATCCCTCAAGTGATTATTTACACCTTTCAAAACATGCTGTACATTATTGTAAAGGTCTTCCTTTAGCTCTTGAAGTGTTAGGTTCCTTCCTTAATTCTATTGATGATCAATCCAAGTTCAAACATATATTGGACGAATATGAGAACTCCTACCTGGACAAAGACATCCAAGATATTCTTAGAATAAGTTATGATGAACTTGAACAAGATGTAAAAGAAATTTTCCTTTACATTTCTTGTTGCTTCGTAAATGAAGACAAAAACAAGGTTCAAATGATGTTACAAGCATGTGACTGTCATTTCAGATTAGAAATGGGAATTAAGAAACTCACGGATCTGTCACTTATTAACATTGATATGTTCAATTGCGTTGAAATGCATGACTTGATACAACAAATGGGTCACACAATTCATCTTTTGGAGCCTTCTAATTCTCACAAAAGAAAAAGATTTTTGTTTGAAAAAGACGTCATGGATGTCTTAAATGGAGATACG

GTGAGAAGGTTTACACGAAATTTGTTTTGTATATATTGATTTTATTATTTCATAGATGACTTTAATTGAAAGTTTTATTTTGTGTCTTTGCAG

GAAGCAAGGGCTGTGAAAGCCATAAAGCTAAATTTTCCTCAGCCCACTGAGCTAGACATTGATTCAAGAGCTTTTGAAAAAGTGAAAAACTTGGTAGTGCTCAAAGTTCACAACGTCACATCTTCAAAAAGTCTTGAGTATCTACCGAGTAGCTTAAGGTGGATCATTTGGCCTAAATTTCCTTTTTCATCTTTGCCTTCAAGCTATTCAATGGAGAAACTTATTGAACTCACAATGCCAAGTAGCTTCATCAAACATTTTGGAAATGGATTTATG

GTACTAATTGATCATGTCTATATATACTTATCAATGATGCTTTAGTTTAATTAATTGATTTAAAATGTCAGCTAATTATTTGAAATTTCATTCTATTTTCTTTTGTACGTAATTCATTGCAG

AATTGCGAATGGTTGAAGCGTATAGATCTTAGCCGCTCTGAGTTTTTAGAAGAAATTTCTGATTTATCCAGTGCAATAAACCTCGAAGAGTTGGATCTTTCTTGGTGTAACAATCTTGTAAGAGTTCATGAATCAGTTGGATCACTAGGTAAACTTGCTACATTGGACCTTTCTAGTCATTCTAATGGCTTTACGCAATTTCCATCCAACCTCAAGTTGAAGTCCCTAAAAGAATTGGTAATGAAGGAGTGCAGGATTGTTAAACGGTATCCTCATTTCAGTGAAGAAATGAAGTCTAGTTTAGAAGAATTACGGATTGAGTATAGTTGTGTGACAGACCTATCCCCAACGATTGGACATCTTACTGGTCTCACACATTTGACGATCGTTGAATGCAAAGAGTTCACAACTCTTCCAAGTACCATTTGTCATTTAAGCAATCTTATTGCTTTAACTGTTATCAATTCTGAACTTTCAACTTTTCCGTTCTTATATTCTCGTTCCCTTGCCTTATTTCCCCACCTAATATGTTTAGATCTTAGTAACTGCAATATAACAAATTTGAGTTTCCTAGAATCCATCACACATGTCGCCCCTTCATTGACAGAGTTGTACTTGACTGGAAACGACTTTCGTAGCCTACCCTCGTGTATTGTTAATTTTAAATATTTAAGACATTTTGATATAAGGAATTGTCGGTTCCTTGAAGAAATTTTAAAGGTTCCTGAAGGCGTAATTTTTATGAATGCTCAAGGCTGCAAATCATTGGCTAGATTTCCAGACAATATAGCTGGATTCATATCTTGTGATTTG

GTACATATCATCTCTTTTCTCCTGGCCTTCATCTCTCTTGTACTTTTTTCATATTATATATATTTTAACGCATTCACTCTTTATATGGTGCAG

GAATTTGTAGATAGAAAATACAGACAACTCATATTAATGAATTGTGATATTCCAGAATGGTTCGATTACAAGAGTAGGAACAATTCGATAACGTTTCCTACGACATTTAATTATCCGGGTTGGAGATTGAAAGTGCTTGCTGCATGTGTTAAAGTTCAAGTTCATGATTGTGTTACTCAGTATCATAATACGGCGGAGCTTGAATGTCAAGTGTTCTTCAATGACATTCCAGTGTGGAGTTCTGAAGACGAGGAAAAATGTCTTGTAGAAGAGTCAAGATGGTTGAGCCTAGAAGCATCACCAAATGACTATACGTGGTTTATTGTACTCAATCCTCATAGAGATTTCTACCTAGATTTGGATGATATGATGGAGGGATCACCAGAGACTGATGTAAGTCAGCTATGTTTTGGAATTAATTCCATGGAAATGGACCATAATATTATACCAGATGATAATTGGAATTCTATTGGGGGAAGTATTTGGAAGAACTTTACGGTGTTGTTTACGCCTCGTCCCGAGCTTTCAGACGCTAAAGTAAGTATAAAAAGTTGTGGTGTTCATGTCATCATGGAGGAATGA

>MELO3C004294

ATGGATCGAGCAAGTGGATCATTTTCCTCACATAGATGGAGGTTTGATGTATTCTTAAGTTTTCGAGGGGAAGATACTCGTTTCAACTTCACAAGTCATCTTTATACGGCTTTACGTCAAAGAGGAATCAATGTTTTCATAGACGATAGCGAGCTCACGAGAGGTGAAAATTTCCCCTCATCTCTTTTGAGAGCTATTGAAGAATCAAAGATCTCGGTTGTTATAATATCTGAAAATTATGCAACTTCGAGTTGGTGTTTGAATGAACTGGTGTACCTTATTATGTGTAAGAAATTGAGAGGACAAGTTGTTTTACCGATTTTTTACAAAGTGAATCCATCTCAAGTACGGACACAAAATGGAGCATTTGGAGAAGCATTTGCTAAACTTGAAGTTAGATTCTTTGACAAGATGCAAGCATGGAGAGAGGCTTTGACTACTGTTTCCTTTATGTCTGGATGGGTGCTTCTTCAAAACGA

GTATTTCTTTCTTCGTCCTTCTACTCTTTTTTCCCTTCAAATTAATTTTTACTATTTTTTTGTAAGGAGATTCATGCTTTGGTCTAGAGGAAAAAGGAAATTAGAAAACTTGATATGTACATCTGATTTGATAACATTTAATTTCAGTTTGTATGCTTTTATGCTTTATACATTTTTATATGGTTGTTATCATCCAATTTTTCAATTTTTTGGTCAAAAAGAAACCCTTGAAATTTTAATCACTTCTAAAAACAAAAGCAATTTTTTAGAACCTAATTCTTTTATGTATCAAGAGGACTACTCTAAATATAGCAAAATAAACAAAAATATTTACAAATTTAGCAAAATATTATCGATAGATGTTAAGCGACATTAAAAGGCTATCATCTATCATTGATTCATGTTGATAAATCCGGGACTATGAAAATTCGTTATGTCTGAAAATATTTTTAGCAGTTTTGCCATTTAAAACAGTTTCCTTATCACGAGGCATGATGAAAGCGTTAAAGATGCATGTATCAATTTAGTTGAGATATCCGGGTGTGTACCTACTTATTCCTTGATTTCAAGTATTTTTTGCCTTTAAAAAAAATATCCCTCTTATCAGTACTTGATTTAATTTTCAAGAAAATTGAAAGAAAATAGATAACAAATCATAGAAACTTATGACTGTGGAATTAGAGTTTAATTGCTTAATTTTCAAAAGCTAAAAGTAAAAAATCAAATGGTTATCAAAACTACCATTGCCTTTTTGTTTCAAACTGTAATAATTCAGTTATATTATTTGTTTTGTCCTGTCTTATCTCCCTAATCATAGACATATTACTGGTTGGATTTAGTTTGAGTTTGTTATGTTGCTTTTATTGCATGGCTACTAATGCATGTTAGGAATTATTGTGAGGAAAAAATTGTGTGCTTCGGAACACTTTTCAGTGTATGCTTTTTTATTATATGGCAG

TGATGAGGCTAGATTGATACAAATAATTGTTCGACATGTCTGGAAGAAATTAACTTGTTCAACGGTGCAGTTGTCTGTAACTAAATATCCCGTTGGAATTGATAGACAAGTTAAGGATTTGCTCTCGCATGTCATAATTGATGAAACTAGAATGGTTGGATTGTATGGAATTGGAGGTATGGGCAAGACAACTTTGGCCAAAGCATTATACAATCGGGTTGCTGATAAATTTGAAGGCTGTTGCTTTTTAGCAAATATTAGAGAAGCTTCAAAGCAACACGATGGCCTTGTTCGACTCCAAGAGAAACTACTTTATGATATTTTAATGTATGACTTTGTTAGAGTTGGTGATGTTTATAAAGGAATCAACATCATAAGGAATCGACTATACTCCATAAGGATTCTCTTGATTCTTGATGATATAGATACAAGTGAACAACTACAGGTATTAGCTGGAGGATACGATTGGTTTGGACATGGAAGTAAGGTCATTGTGACAACAAGAAATGAACAGTTACTTGATATTCATGGATTTTATAAATTGAAAGAAGTTCCTCAATTGCATTTTGGTGAAGCTCTTGAGCTTTTTAGCTGGCATGCGTTTCACAATAGTTGTCCACCAAGCGAATATTCAACACTTCCAGAGGATGCTGTAAATTATTGTAAAAATCTTCCCTTGGCGCTTGAAGTTTTAGGTTCATTCCTTTATTCTACTGATCAATCCAAATTTAAAGGTATATTGGAGGAATTTGCAAACTCCAACCTTAACAAAGACATCCAAAAGCTTCTTCAAGTAAGTTACGATGAGCTTGAAGGTGATGTACAAGAAATGTTCTTGTTTATTTCTTGTTTCTTTGTGGGAGAAGATAAAACCATGGTTGAAATGATGTTGAAGAGTTGCGGTTGTTTATGTTGGGAAAATGGAATTAAGAAACTCATGAATCTATCCCTTCTTACTATTAACAGAATAAATAAGGTACAAATGCATGACTTGATAAAACAAATGGGTCACACAATTGCTCGTTCAAGGACTTTTATATCTCATTCAGAAAAAAAAATAATGGTTGAAGATGAAGCTATGCATGTCTTAGATGGCATTAAC

GTAAGAAGTTATTGCTCAATCTTTGTTTATGCTTATTTATTATGAGAGCAACTTTTATAATTTTGAAAAAACTTGAAATCTTTGTGTTTGTTTCATTTGCAG

GAAGCAAGAGCAGTTAAAGCCATAAAAATGGAATTTCCTAACCCGACAGAGTTGGACATTATTGATTCAAATGCTTTTAGTAAAGTAAAGAACCTCGCAGTACTCAAAGTTAAGAATGTCACATTTTCAAAAATTAGTACTCTTGATTCTCTACCTAATAGTTTAAGGTGGATGAGTTGGTCTGGATTTCCTTTTTCATCGTTTCCTTCAAGCTACTCAATGGAGAATATTATTCAACTCAAATTGCCACATAGCTCCATTAAACGTTTTGAAAAAGAAGCATTTACG

GTATTTCTATCAATAATTGAGTTTTATTAATTTCAATGTTTGTTTATTTACAATTTCATTATACTTTCTTTTTATATTCATTACAG

CATTGCAAATGGTTGAAGGAACTTGATCTTAGCAACTCCATCTTTTTGGAGGAAATTCCTGATTTATCTGCGGCGACAAATCTCGAAAAATTGTCTCTTTCTGGGTGTGAGAATTTAGTAAAGGTTCATAAATCAGTTGGATCTCTCGGTAAACTTGTTGATTTGTGTATTTCAAGCCATGTTTATGGTTTTGAGCAGTTTCCATCACCGCTAAAGTTGAAATCCCTTAAAAGATTTTCAACTTATCATTGTACAATAGTTCGAGGGTATCCACAATTCAGTAAAGAAATGGAGTCTAGTCTAGAACATTTGTGGTTTTATAGAAGTTCTATAACAGAGCTATCTTCAACAATTAGATATCTTACCAGCCTCAAAATTTTGTCCATCACAGATTGCAAAGAGCTTACAACTCTTCCAAGTACAATTTATGACTTAAGCAAACTTACATCCATAGAAGTCTCACAATCCGATCTTTCAACATTTCCTTTCTCATATTCTTGCCCTTCCTCACTTCTCCACCTAACAAGATTAGACCTTTATGAGAACAAGATAACCAATTTAGATTTTTTAGAAACGATCGCTCATGCTGCTCCATCACTGAGAGAGTTGAACTTGTCTAACAACAACTTTTCTATACTACCCTCATGCATTGTTAATTTTAAATCCTTGAGATTTCTTGAAACAATTGATTGTAAGTTGCTGAAAAAAATTCCAAAGATTCCAGAAGGCTTAATTTATTTGGATGCTCAAGGGTGCATATCATTGGCCAAATTTCCTGACAACTTAGCTGATTTCATATCATGTGATTCG

GTGCACATCTCATCTCTTTCTCTTGTCTTGATCTCTCTTGTAATTTTCTTTTCATACAGTAGTTAAGTTCATATGTGATTGTGAATTAATAATATTATTACTCTCAAACATTCACGTTGGTGCAG

GAACATGTAGATGGACAATTCAAACAACTTATATTAATGAATTGTGATATTCCAGATTGGTTCAGTTACAAGAGTAGGAACAATCCAATAACGCTTTTGGTGCCATCCAATGATCCAAGTTCGGAATTGAAGGTTTTTGCTGCTTGTGTCAAATTTCAAGTTAATCATGTTGACCAGGATCAATATATGGATCTTGAATGTAAAGTGTTCATAAACGACATTCAAGTATGGAGTTATGAAGAAGTACCCTTTCACGACGAATCGAGAAGTATATTGATAAAAGCATCACCACATGAGTATATGTGGTTACTAGTACTATATCCACATATAAATTTCCGACTAAATTCGGATGATATTATCAATAGATCACAGGAGATCAATCTACATCAGCCAAGTTTTGGGATCAATTCCATTGGAAGGGACAATAATAACTGTAATGTAGATGATGATTATCGGAGGAATCATATTGGGGAGAGTATTTGGAGGAAATTTACGGTGTCGTTTGGTGTTACTTCCAAATTTAAAGACTCCGAATTAAGTATAAAAACATGTGGTGTTCATGTCATCATGGAGGAATGGTGTGACTGAGGTAGCCTTTTTACTGTTGCCTGTAAAGGACCAATCAAATGAAATTTATACTATGATGCTCATTACT

>MELO3C00430

ATTGCGTTTTGTTTTTGTGAGAGAATGGGTTCTTCTGCTCTTCCAGTTGAATCTTCTTCTTCTTCTTCTTCTCCCAACTTTCTCTATTACTACGATTATGATGTGTTTTTTAGTTTCAGAGGAGAAGACACTCGCTCCAGTTTCATCAGTCATCTTCATATGGCCTTGCGTCTAAAGGGAGTCAACGTCTTCATAGACGACAAACTCAAAAGGGGTGACCAAATCTCTGAGTCTCTTCTCAAATCTATAGAGCGATCTAGACTTTCACTCGTTATTTTCTCTAAAAATTATGCATCTTCAACTTGGTGTTTGGATGAACTGGTGAAAATAATTGAGTATAAAAAATCCAAAAGTCAAGCGGTTTTGCCGGTGTTCTACAAGGTGGATCCGTCCGAGGTTCGAAAACAAACCGGTGGGTTTGGGGAAGCATTAGCCAAACATGAAGCTAATAAGTTATTGACCAACAAGATTCAACCATGGAAGGAAGCTTTGACTTTTGCTGCTGGTTTGTCTGGTTGGGATCTAGCAAATTG

GTATTTCTTTTTTTAATTTCCCAAGACTCATTGTCTAAGTGAAGTTTAAATTTATCTACTATTTCCTATTTTCAAATTTTTTTTTTAAGTATGATGTTACTTTACAACAG

CAAGGATGAGGCTGAACTTATCCAAGAAATTGTTAAACGAGTGTTGTCTGTATTAAATCCAATGCAATTACTACATGTAGCCAAGCATCCAGTTGGAATTGATTTTCGATTAAGGAAAATTGAGGAGTTGGTCTCTCATATTGGGTCCGAGGGTGTTAACATGGTGGGGATGTATGGCATTGGAGGCATTGGTAAGACCACTTTAGCTAAGGCTTTGTACAACAAAATTGCTAACCAATTTGAAGGATGTTGCTTTCTACAAGATGTTAGACGAGAAGCTTCAAAGCATGGGCTCGTTAAACTACAAGAAACCTTACTCAATGACATCTTAAAGGAGGATTTGAAGGTTGTCAGCCGTGATAGAGGAATTAACATCATAAGGAGTAGACTGTGTTCAAAGAAAGTTCTTATAGTTCTTGATGATGTGGATGATCGTGAGCAATTAGAAGCACTGGTTGGTGGTCGTGATTGGTTTGGTCGAGGTAGCAAAATCATTGTGACGACAAGGAATGAGCATTTACTTTTTAGCCATGGATTTGATGATCAAAAGCATAAAATTCAAGAATTGAATCAAGATCATGCTCTTGAACTTTTTAGTTGGCACGCTTTTAAGAAAAGCCATCCATCAAGTAATTATCTAGGCCTTTCAGAACGTGCTACAAATTATTGTAAAGGTCTATCTTTGGCACTCGTTGTTTTGGGTTCTTTCCTTCGTGGCAGAGATCAAGCAGAATGGAACTGTATATTAGATGAATTTGAAACCTCTTTGAGAAAAGATATTAAAGATGTTCTTCAATTAAGTTTTGATGGACTTGAAGACAAAGCAAAGGATATTTTCCTTGATATTTCTTGTTTACTTGTGGGAGAAGAATACAATTGTGCTAAAAAAATGTTGAGTGCATGCCATTTGAACATAGATTTTGGAATTATGATACTCGTGGATCTTTCACTTGTTACTATTGAAACGGATAGAGTGCAAATGCATGAGTTAATACAACAAATGGGTCGTAGCATAGTTCATAATGAATCATCTGAGCCTGGAAAGAGGAGTAGGTTGTGGTTGGTGCAGGACATTTGGGAGGTGTTTGTTAATAATTCA

GTGAGTAACTCCTACCTAAAGTATTTAATAATTTGGACTTCACCTGATAATATATAAATGTTTGTCAAAGTAATTAAATTGTCAGTAATGTTAAATTACTAAATATATGACTTTGTAG

GGAACAGATGCAGTTAAAGCCATAAAGTTGGACTTGCCTAATCCCACAAAGCTAAATGTAGATCCACAAGCATTTAGAAGCATGAAAAATTTGAGATTGCTTATCATTCGAAATGCACAATTTTGTAGAAAGATTAAGTACCTACCTAATAGCTTAAAGTGGATTGAGTGGCGTGGATTTGCTCATCGATCTTTGCCGTCATGCTTCATTACCAAAAATCTTGTTGGACTTGATTTGCGACATAGCTCCATCAAAAGATTTGGGAAAAGGCTCGAG

GTAAAATATATTTCTTTTTGCATCTGTATTTGACTGGAGATTTCTTCATTGATTTTCTTAAAAAGTTTTAATGGGTAGCTTAACTGCACCGGATAAAAAAAAATCACAAATAGGTGATTTAGCAAAATTTAACATGGATTAACCCGTGCTAATCACATACTTTTAAAGAATTTGCAAATTATATCAAAGTTTATTAATGATATAGTGATCTATCGCACAACCATAGACTTATGCTAGTCATATGATCTATCATTAATATACTCCTATTAGTGATATGATCTACATGGATTAATTCCAAGGGGTCTTTTGGGGAAAGGGTTGGATTATGGGAGTTAAGGTTATGATAAAACTAGTGTTATGACAAATCTAGGATTACGATAAAGATGTATTTAGTGGAAGGGTTATGGAGGAAGTATTATGATAAAATATGTTTGGGAGAAGGGTTATGTGGGTAGGGTTATGATAAATATATATATATATATATATATATATATATATATATATATATATATATATATATATATANNNNNNNNNNNNNNNNNNNNNNNNNNNNNNNNNNNNNNNNNNNNNNNNNNNNNNNNNNNNNNNNNNNNNNNNNTTGTCCTAGTTTTCATAAAATAATTTGCCTTAATTTATTTAACTACGTTATTCAATTTCAACATACACATTGTTTATTTTCAACATTTTTTAAGTATACGTTACGTTTCAATAATTTGGTTTTCATGAATATGACACACAACCTGTAAATATGTAAATAAGGGTAAATAACAATTGAAAATGCGAATTACCGAAAACAGAAAATAATAATCAACGCTTTTACAAACTCTAGTTTTGTTTTCTAGTGAAAAATTACGATGAATCTAGTTTTAGGTTTCTCTTCAATTTATTTTAGTCTACTTTTAAAATATTAACTTTATTTTTTATTCTTTTTAACGGTGATTTTTATACTTTCAAAATATTTATTTTAGTCATTTTGCAACAATTATTGGGTAAAAAATTCATGAATCTACCGCCTCTATAACAAAAAAATTTGTTATATTAATTTAACCAAGCTTAATTTAATAACCAAATTAACATTATGAAAGTGGGAAAATATAGACAAACTGCATCTAAATAATAGATTTGTTAAATTATAGCCCCACCAATTTTTAAATTATTCAAATAGTCCTTATGATTTTCTAATTGTTTTAATCAATTTATGGTAGTTTGTTAAAATTTTACCATTTTTTTTATTTAATTGATATTCCTTAGATATTTATCCTAGCTAACTAAAAAAAAACAATGAGATTGAGATTTATATTAAAATTTTAATTTCATAAATATATTGATATGAATTCAAACATAGAGTTGATTGAATGTAACTAAATTAAAAATTTAATTGTGAAATCACTATAAATTATCTAATTCAAGAGTAAAGGAAAAAATTTAAATTTTAATAATGTAATTATACCAAATTCATAATTAAAAAACAAGCTGTGTTTTTAAAATAAAAAGATTAAAATGAACAAAACTCATAAATATAAAAATTATAAATGAGAAAAAAATGAATTGAGGAAGGGTTGAAGAAAGGTTGAGGGGATAAAACTAAGGTTATGATAACCCTATAATTAAAAAGACTAAACTACATTCTCATTCTGTTTAAGGTATCAAATTAAATGGTTAGTTTACAATCTACTAAATTCAAGATAATATATTTTAGGTATAATGGTTACTTTACAATAATATTAATTAATTAATTACTTATAAACTAAAATTTAATTCAAAACTATGTTTAATTTAACTAATTCTCTCGTGCCTAAATTATATTTTTTAAACTTTTGTATTAAGTTGCATTTTAAACTTATAAAACACACTTACTATCGATATGTATCAAACTAATAGTTGTAAAAAGTGCATTGTTGCTAACATGAGTACAGCTGAACTGACATAATATTGATTAATGGTTATTATACAATTTCTTTTACCTATATTCTATATTTTTCTATATTTACGATTGTTGTTTTAGCCACTATTTTATCTAAATTTTGTTTTGTATGGCAATTACATACCTCCTTTTCCTTTGGTTTTTTTTTTCAATGTGTTTTAG

GGTTGTGAAAGGTTGAAGCATGTTGATCTTAGCTACTCTACTTTATTAGAGCAAATTAATGATTTCTCTCCGGCATCAAATCTTGAAGAATTGCATCTCATCAATTGCACAAATTTAGGAATGATAGATAAGTCTGTTTTTTCTCTCTATAAGCTTAGTGTCCTAAACCTTGATGGTTGTTGTAACCTTCAAAAGCTTCCAAGAGGCTATTTCATGTTAAGTTCTCTTAAAGAATTGAATCTCTGTTACTGCAAAAAGCTTGAAAAAATTCCAGACTTATCTGCAGCATCAAACCTTAAGAGATTGTATCTCCAAGAATGCACAAATTTAAGAGTGATTCATGAATCTGTTGGATCTTTGGATAAGCTTAATCATCTGGACCTTAGACAATGCACTAAACTGGTAAAGCTTCCAAGCTATCTCAGGTTAAAGTCTCTTTCCAATTTATTACTTTCTGGGTGTTGTAAGCTTGAAAGCTTCCCAACAATTGCTGAAAACATGAAATCTTTAAGGGAATTGGATATGGATTTTACTGCCATAAAGGAGTTACCTTCATCAATTGGATATCTTACTAACCTTTCTATATTAAAACTTAACGGTTGCACAAACCTCATCTCCCTTCCCAATACAATTTATTTGTTAAGGAATCTTGAGAATCTTCTTCTTAGTGGCTGTTCTATATTTGGAATGTTTCCCCATACATGGGACCCAACCATCCCAACCATCCAACAAGTATGCTCTCCTTCAAAAATGATGGAAACAGCTTCCTGGAGCTTAGAATTTCCCCATTTACTAGTACCAAATGAAAGTTTATGTGCCCATTTCACTTTGTTGGATCTTGAATCTTGCAACATATCAAATGCAAAATTTTTAGAATTATTATGTGATGTTGCCCCTTTCTTATCTGATCTACGCTTGTCTGAAAACAAATTCTCTAGTTTACCCTCATGTCTCCACAAGTTCATGTCCTTGTGGAATCTTGAATTAAGGAATTGCAAGTTTCTTCAAGAAATTCCAAACCTTCCTGAGAATATACAAAAAATGGATGCCAGTGGTTGTGAATCGTTGGCTCGAAATCCAGATAACATTGTGGATATAATATCAAAAAAACAG

GTTCGCCTCTAATTTCCATTCAATTTATATTCTTATCTTGTAAACAATTTAATGCATTATGAATTCTTGTTCTCTATAG

GACCTCACATTGGGTGAGATTTCAAGAGAGTTTTTATTAACGGGGATTGAGATTCCAGAATGGTTCAGCTATAAGACTACATCCAATTTAGTGACAGCTAGCTTTCGTCACTATCCAGACATGGAAAGAACTTTGGCTGCCTGTGTTAGTTTCAAAGTGAATGGAGATTCATCTAAAAGAGGAGCCCAAATTTCATGTAGTATATTCATCTGCAGTAAACTCCATTCTTCATTTTCAAGACCATTTCTTCCATCAAAATCAGAATATATGTGGTTAGTAACAACTTCTCTAGCGTGGGGTTCCATGGAGGTGAATGATTGGAATAAAGTTTTGGTCTGGTTTGAGGTTCATGAAGCACATAGTGAGGTTAATGCAACTATAACAAGGTGTGGTGTTCATGTCACTGAAGAGCTCCATGGGATACAAATGGATGTCAAGTGGCCGATGGTAAATTATGCTGATTTTTATCAACTGGAGAAATTGCAAAGTCT

GTAAGTTGATTGTTAACTTGTTATTTATTTATTCTTTCTTTTTTTTTTTTTTTTTTTTTTTTTTTTGGTAGTGAGAGTATATAATAGATCTCAAAGGGGGAGATGAGTATGTTATGATTTTGTTTGTTTAATGGCATAG

GGATATTGAGGATCTTCTTCTCAAAAGCTTTTTAGAAACAGTCTCTTGCCTGTCAAATTCCAAAGCAGCAATGTTACATGCAGGAAATTATGATCCAGAAGCAATAATTGATTCCAACATACAACCTATGATATTTCCATTGCACGTAACAAATAATGATGGCACATATATATGTGGAGGCATGGGAGGCACTGCACTTGCCAACTCTTTATGCAATAAATTTAAAGGGATGGAGGGCCAATGCGGTGAAGCTTTAGATAATTCTACAAGCTTTTTCCATATCAAAAGAAGACAACTCCTGAGCTATTCCTGGTCGCCGGCGGTCCACCATCGTAAGTGTGGAGATGGTGAAAGAGGAACGAATATCACAACCCACACAATATCCTCCAAACGCTATTTGATACTCCTTCGTGAAGCCAAGAGCTATCAGGATGTACATGACTGGTTTTATACACATTGTTGGATAAAAGCTTCATATTGCAGTTATGACGGAAGAGGTGATGGTGTGATTCTGATTGAAGGGGTTGATACATCCTTGCTCTGAAGCTCTCAATTTTTAGTAAAAATTTACATTTAAATGTCCTTTTAGTCTGTTTGGTACTTGTATTAATTTAAACTCTATGATAATTAACATCAATTTAAACGCTCAATAACTTTTGTAAGTGTACGTATCAATTGAACACTCTATTCTTATTTAATTTAGATTTTGTTCA

>MELO3C004303

ATGGTGGGGATATATGGCATTGGAGGCCTGGGTAAGACAACTTTGGCTAAAGCTTTGTACAATAAAATATCTAGCCAATTTGAAGGGTGCTGCTTTCTATCAAATGTTCGACAAGCTTCAAAGCAATTCAATGGCCTTGTTCAACTACAGGAAAACCTACTCTATGAAATCTTAAAGGATGATTTGAAGTTTGTCAATCTTGACAGAGGAATTACCATCATAAGGAGTAGACTGCATTCAAAGAAAGTTCTCATAGTTCTTGATGATGTGGATAAGCTCGAGCAATTAGAAGCATTGGTTGGTGGACGTGATTGGTTTGGTCAAGGTAGTAAAATCATTGTGACAACGAGGAATAGACATTTACTTTCTAGCCATGGATTTGATGAAATGCACAATATTCGAGGATTGTATCAAGACAAAGCTATTAAGCTTTTTAGTTGGCATGCTTTTAAGGAAAGTCATCCATCAAGTAATTATTTAGGCCTTGTCGAACGTGCTACAAGTTATTGTAAAGGTCATCCTTTAGCTCTTGTTGTTTTGGGTTCTTTCCTTTGTACTAGAGATCAAACAGAATGGATTAGTATATTGGATGAATTTGAAAACTCTTTGAGCAATAATATTAAAGATATTCTTCAATTAAGTTTTGATGGGCTCGAAGACAGAGTAAAGGATATTTTTCTTGATATTTCTTGTTTACTCGTGGGAGAGGAAGTTAACTACGTTAAGAATATATTGAGTGCATGCCATCTCAATGTAGATTTCGGAATTATAATACTCATGGATCTTTCGCTTATTACGGTTGAAAATGGTACGGTGCAAATGCATGATCTAATACAGCAGATGGGCCATAAAATAGTTTATGGTGAATCTCCAGAGCCTGGAAAGAGGAGTAGGTTGTGGTTGGCGCAAGACATTTGGGAGGTGTTTGTTAATAATTCA

GTGAGTAACTCTTACGCAAAGTATCGAAAATTTGCTTATTTCCAGGACTTTACACGACAACAAATGTTTGTCAAAGTAAGTTGTCAGTAATGTTAAATTACTAAATCTATAACTTTGTAG

GGAACAGATACAATTAAAGCCATAAAGTTGGACTTGGCTAATCCTACAAGGTTACACGTGGATCCACAAGCATTTAGAAGCATGAAAAATTTGAGAGTACTTATTGTTCAAAATGCACGATTCTCTACAAAGATTAAACACTTATCAGATAGCTTAAAGTGGATTAAGTGGCATGGATTTGCTCATCGATCTTTGCCGTCATGCTTCATTACAAAAAGTCTTGTTGGGCTAGATATGCAACATAGCTTCATCAAAAAATTCGGGAAAAGACTTGAG

GTAAATTATTCTTCTACGGGATGTTTACTTGATTGGAGGTTTCTTCATTGTTTTTATTAGAAATTAAATGGGTAGCTTTCATTTTAGAAAAGTTCAAAATATGTAGTTTTGTAATAAAAAAAATGATAATATGATAATTTTTAAAAATTAGTAAACGGGTAGCATTGTATTTTAAACAAACAATGTTATTTTGTTGATACACTGATCAAATGAAATCAATTTCATATCAAAAGGAAACAAAAAACAAAAACAATATTCACCATACTCGAACAAGCATTTGCCATAACGTAATTAAAGTCATGCTATATGATTACTTTATATTTGGTATATCAATCAACTTTGTATTTGGTAAATCAATCAACTTTACAAAGACAAGTCAAATATACTTTAAGATACATAAATGAGAGTAAAATTGAAATCATAGAAAATACGAAAATCAGATTCTTTTTTCAACTCATTACAATATTCCTTAACTAAAATAGTAGTAAGAAGTATGTTGCTATTTTATGCAATAGTTACGGATTAGTACTGTGGATTAATGTATATAGATTACTAATCTTGATATGGATTGAGTTTATCACTGATCTACTTATATATATCATTGATTTACTTTTGTATATGGGTATTATTAGTTTACTTGAATATTGATTATTGGTTATTATTGATTTACATATATTCTATTTTTCTATATTTACAAGCATTCTTTTAGTCGCTATTTCTGTAATATTTTATCTAAATTATGCTTCGTATGTCAGTTACGACCGTTTTTCCTTTTGGTTTCTTTTCCATGTGTTTTAAGATTGTGAAAGG

TTGAAGCATGTTAATCTTAGCTACTCTTCTTTATTAGAGCAAATTCCTCATTTCCCTGCGGCATCAAACCTTGAAGAATTGCATCTAAGCGAATGCACGAATTTAAGAAAAATTGATAAGTCTGTTTTTTCTCTTGATAAGCTTACTATCCTAAACCTTGATGGCTGTTCTAACCTTAAAAAGCTTCCGACAAGCTACTTCATGGTAAGGTCTCTTAAACATTTGAAGCTCTCTTACTGTAAAAAACTTGAGAGAATTCCAGATTTATCCTTAGCATCAAACCTTGAGAGTTTATATCTTGAAGATTGCACAAATTTAAGAATGATTCATGAGTCTATTGGATCTTTGGATAAGCTTGTTACCTTAGTCCTTAGACGTTGCTTTAATCTTGCAAAACTTCCTAGCCATCTTCACTTAAAGTCCCTACAATATTTAGGACTTTCTGGGTGTCGGAAGCTTGAAAACTTCCCAACAATTGCTGAGAACTTGAAATCTATAAAATTGTTGGATTTGGATTTTACTGCCATAAAGGAGGTACCTCCATCAACTGGATATCTTACTCAGCTCTCTCGATTAAATATCAACGGTTGCACAAACCCCATCTCCCATCCCAATACAATTTGTTTGTCAAGATTGTATTTGTTAAGGAGTCTTGAAAATATTTTTCTTAGAGGGTGTTCTAGATTTGAAATTGTTCCCCATGAATGGGGCCAAATCATCCAACCAGCATGCTCTTTCTCAAAGATGATGGAAACAACTTCATGGAGCTCAGAATTTCCCCATTGAC

>MELO3C004309

ATGGGTTCTTCCATTGTTGGAGTGGAATCATCAACTTCTTTGAGTTTCAAGTGGAGTTATGATGTGTTTTTGAGTTTCAAGGGAGATGATACTCGTTCTAATTTCACTAGTCATCTTGACATGGCCTTGCGTCAAAAGGGTGTCAATGTCTTCATAGACGACAAGCTCAAAAGGGGTGAGCAAATTTCTGAAACCCTTTTCAAAGCTATACAGGAAACTTTGATTTCTATTGTTATATTCTCTCAAAATTATGCATCTTCTTCATGGTGTTTGGATGAATTGGTGAAAATAATTGAGTGTAAGAAATCCAAGGGCCAGCTTGTTTTGCCAATTTTCTACAAGGTGGATCCTTCCGATGTACGAAAACAAACGGGTTGCTTTGGAGAAGCATTGGCCAAACATCAAGCTAATTTCATGGAGAAGACTCAAATATGGAGGGATGCTTTAACTACTGTTGCCAACTTCTCTGGTTGGGATCTAGGAACTAG

GTATATTTTTATTGACATTTTGTCTTTTCTTTTCCATATTTCATTTCCATTCCTCTAATTTTGTTGACAAAGCACACTAACAAATTCCTACTTGAAATAATTTCAACAATACAAGTGACTGATGTTAATCATAGAAGATTCACTCTCAAGATGAAGTTTAATTTTATCTACTATTTTTTCTCATGTTCATCAAGTTTTATTTGTTTACATATGATGTACTTAACAACAG

GAAGGAGGCTGATTTTATTCAAGATCTTGTTAAAGAAGTATTGTCTAGATTAAATTGTGCCAATGGGCAGTTATACGTAGCTAAGTATCCGGTAGGAATTGATTCTCAACTAGAAGATATGAAGTTACTCTCACATCAGATACGAGATGTGTTTGATGGCGTTTACATGATGGGGATATATGGCATTGGAGGCATTGGTAAGACAACTTTGGCTAAAGCTTTGTACAATAAAATTGCTAACCAATTTGAAGGTTTCTGTTTTCTATCAAATGTTAGAGAAGCTTCAAAACAATTCAATGGCCTCGTTCAACTACAGGAAAAACTACTCTATGAGATTTTAAAGGTTGATTTGAAGGTTGACAATCTTGATGAAGGAATTAACATCATAAGGAGTAGATTGCGTTCAAAGAAAGTCCTTATAGTTCTTGATGATGTGGATAAGCTCAAGCAATTGGAAGCATTGGTTGGTGGACGTGATTGGTTTGGCCGTGGTAGTAAAATCATTGTGACAACAAGAAATAGTCATTTACTTTCTAGCCATGAATTTGATGAAAAGTATGGTATTCGGGAATTGAGTCATGGCCATGCTCTTGAACTTTTTAGTTGGCATGCTTTTAAGAAAAGTCATCCATCAAGTAATTATTTAGACCTTTCAGAACGTGCGACAAGTTATTGTAAAGGTCATCCTTTGGCTCTTGCTGTTTTGGGTTCTTTCCTTTGTACCCGAGACCAAACAAAATGGAAAACTATATTAGATGAATTTGAGAACTCTTTGAGCGAAGACATTGAACATATTATTCAAATTAGTTTCGATGGGCTTGAAGAAAAAATAAAGGAGATCTTCCTTGATATTTCTTGTTTGTTTGTGGGAGAGAAAGTTAATTATGTTAAAAGTGTGTTAAACACGTGCCATTTCAGCCTAGATTTTGGAATTATAGTTCTCATAGATCTTTCGCTTATTACGGTTGAAAATGAAGAGGTTCAAATGCATGATTTAATTCGACAAATGGGCCAGAAAATAGTTAATGGTGAATCTTTTGAGCCCGGGAAAAGGAGTAGGTTGTGGTTGGTACACGATGTTTTGAAGGTGTTTGCTGATAATTCA

GTGAGTAACCCTTACCCAAAGTATCTTTAGTTTTCACTTATTTCCTAGACTTCATGATGGAAGATGTTTGTCAAATTAAGTTGTCAATTATGTTAAATTACTAAATTGATGTCTTTGTAG

GGAACGATTGCAGTTAAAGCCATAAAGTTAGACTTGTCTAATCCCACGAGGCTAGACGTGGATTCACAAGCTTTTAGGAACATGAAGAATCTGAGATTGCTTATCGTTCGAAATGCAAAATTTTCGACAAATGTTGAGTATCTACCTGATAGCTTGAAGTGGATTAAGTGGCATGGTTTTTCTCATCGTTCTTTGCCATTGTCCTTCCTTAAGAAAAATCTTGTAGGACTAGATTTAAGTCATAGCTTCATCAAAAATTTGGGCAAAGGATTTAAG

GTAATTATATATCTACCTGTATTTAGTTGGAAGCTTCTTCATTGATTTTCTTAGAAATTTTCATGGGTAGCTCTAGCTTAGTTTCTAAAGATTTGCAAATATTTTATGTTAAAAACAACAGCTTGCAAACATAAAGAGAGTTATGTTCATGTGAAAAATATTTGTTAATGCGATATATATGCTTTAATCACTAACTCGTTTACTTATGTAAATAACAGTGGTTTATTCTAAACCATAACTTTGATATTTTTTCTCTCATTTTGAAATAATGTCTAATTATGTCCGATCAATATTTACTTCCNNNNNNNNNNNNNNNNNNNNNNNNNNNNNNNNNNNNNNNNNNNNNNNNNNNNNNNNNNNNNNNNNNNNNNNNNNNNNGTATTATTTTTCTCTCATTTTGAAATAATGTCTAATTATGTTCCGTATTCTAATTATTTTACTTTCCTTTTTTTTTTTTTTTTGTTTCTTTTCCATGTATTTTAG

GATTGTAAAAGGTTGAAGCATGGTGATCTTAGTTACTCTTCTTTATTAGAGAAGATTCCCGATTTCCCAGCAACGTCAAATCTTGAAGAATTATATCTTAACAACTGCACAAATTTAAGAATAATTCCCAAGTCAGTTGTTTCTCTTGGTAAGCTTCTTACTTTAGACCTTGATCATTGTTCAAACCTTATAAAGCTTCCAAGCTACCTCATGCTGAAGTCTCTTAAAGTTTTGAAGCTTTCTTACTGCAAAAAACTTGAGAAACTTCCAGACTTCTCTACAGCTTCAAACCTTGAAAAGTTGTATCTCAAAGAATGCACAAATTTAAAAATGATTCATGATTCTATTGGATGTCTGAGTAAGCTTGTTACCTTGGACCTTGGAAAATGCTCTAACCTTGAAAAGCTTCCAAGTTACCTTACATTAAAGTCTCTTGAATATTTGAATCTTGCTCACTGCAAAAAGCTTGAAGAAGTTCCCGACTTCTCTTCTGCATTAAACCTTAAAAGCTTGTATCTTGAACAATGCACAAATTTAAGAGTGATTCATGAGTCTATTGGATCTTTGAATAGTCTTGTTACCTTGGACCTTAGACAATGCACTAACCTTGAAAAGCTTCCAAGCTACCTCAAGTTGAAGTCTCTTACACATTTCGAACTCTCTGGCTGCTGCAAACTCGAAATGTTTCCAAAAATTGCTGAAAACATGAAATCCTTAATGTCATTGCATTTGGATTCTACTGCCATAAGGGAGCTACCTTCATCAATTGGATTTCTTACTGCGCTTTTGCTATTAAACCTTAACGGTTGCACAAATCTCATCTCCCTTCCTAGTACAATTTATTTGTTAAAGAGCCTTAAGCATCTTTACCTTGGTGGGTGTTCTAGATTTCAAATGTTTTCCCATAGATGGGACCCAACCACCCATCCAGTATGCTCTTTTTCAAAAATTATGGAAACTTCATCGAGTTCAGAATTTCCCCATTTACTAGTCCCAAAAGAAAGCTTATGTTCCAAGTTCACCTTGTTGGATCTTCGATGTTGCAATATATCAAATGTAGATTTTTTGTAA

>MELO3C004311

ATGAATCTAGCAAGTGGATCATCTTCTTCCTCACGTTTTAGATGCAGTTTTGATGTATTTTTAAGTTTTCGAGGGGAAGATACTCGTTCCAACTTCACCAGTCATCTTAATATGGCTTTGCGTCAAAGAGGAATCAATGTTTTTATAGATGATAAGCTTTCAAGGGGTGAAGAAATTTCTGCATCTCTTTTGGAAGCTATTGAAGAATCCAAGATCTCGATCGTTATAATCTCTGAAAATTATGCATCTTCAAGTTGGTGTTTGAATGAACTAGAGAAAATCATTATGTGTAACAAATTGAGATGGGGAGTACAACTTGTTTTACCAATTTTCTACAAAGTGGATCCATCTCAAGTAAGAAAACAAAGTGGAAGATTTGGAGAAGAATTTGGAAAACTCGAAGTGAGATTCTCATCGGACAAGATGGAAGCGTGGAGGGAGGCAATGATTTCTGTTTCTCATATGTCTGGATGGCCGATTCTTCAAAATGA

GTAAATTTTCTTTTTTATATATATATCTTTAATTCTACTCTTCTGCTCTTCTTATTCTATTGTGTTATTATATTATGTATCTCACAGTGAGCATCCTGGACTAAGGGAAAACACCTTATAGTTTTCAAAACTTATTTGCCTTAATTTTTAAAAATAGTAGAAGAAGGCAATAACAAATCATAAATTCTTATATTATTTAATTTGTTATTATATTGTGCTGTCTCCTCCTTTCTATAATAGATATTATAACTTGGTTGGATTTAGTTTGACTTTATAATGTTGCTTTCATTTTCTCTGGCTTCTCATATTATAGAAATTAATTATAATATTAGATATTTGAAATCAACTTTTAGTGTATGTTTCTTTCCTAAAATGACAG

TGACGAGGCCAATTTGATTCAAGAAATTGTTCAAGAAGTCTTGAAGAAATTAAATCGTGGAACAATGTTGTTGCGTTTACCTAAATATCCTGTTGGAATAGATAGACAAGTTAATAATATACTCTTCCAAGTTATGTCAGCAGATGAAAAAATTACTATGATTGGAATATATGGAATTGGAGGTATCGGCAAGACAACTTTGGCCAAAGCATTGTATAATAGAATTGCTGATGACTTTGAAGGTTGTTGCTTTTTGGCAAAAATTAGAGAAGCTTCGAATCAATATGATGGCCTTGTTCAACTCCAAAAGAAGCTACTTTGTGAGATTCTAATGGATAATTCGATCAATGTTAGCAATCTTGATATAGGGATTAACATCATAAGGAATCGACTATGCTCAAAAAAGATTCTTTTAATTCTTGATGATGTTGATACGAGAGAACAACTAGAAGTATTAGCGGGAGGACATGATTGGTTTGGACCTGGAAGTATGGTCATTGCGACAACAAGAGACAAACATTTACTTGCTATTCATCAATTTAATATATTGCAAAGTGTCCAGGGATTGAACGATGGTTATGAAGCCCTTGAGCTTTTTAGTTGGCATGCTTTTAAGAGGAGTTGTCCGTCAAGTGATTATTTAGACCTTTCAAAACGTGCCGTACGTTATTGTTTAGGTCTTCCCTTGGCTCTTGAAGTTGTAGGTTCCTTCCTTTTCTCTACCGAACAATCCAAGTTTAAACTTATATTGGATGAATATGAAAACCAATACCTTGACAAGGGCATCCAAGATCCCCTTCGAATAAGTTATGATGGACTTGAAAACGAAGTAAAAGAAATTTTTCTTTATATTTCTTGTTGCTTTGTAAGAGAAGATATCTACGAAGTTAAAACGAAGTTAGAAGCATGTGGTTGTTTATGTTTGGAAAAGGGAACAACAAAACTTATGAATCTATCACTTCTTACCATTGATGAACATTCCAACCGGATTGAAATGCATGACTTAATACAACAAATGGGTCGCACAATTCATCTTTCGGAGACTTCTAAATCTCATAAAAGAAAAAGATTGTTGATTAAAGATGATGTTATGGATGTCTTAAATGGCAATAAG

GTAAGAACTATTAGATAAAACTAATATTTTATATATGCTTATATTTATTTATTATGAGAACAACTTTTATTATTTTGAATTGTTTGTTTTGATAATTTGTAG

GAAGCAAGAGCAGTTAAAGTTATAAAATTAAATTTTCCTAAACCTACAGAGTTGGAAATTGATTCAAGAGCTTTTGAAAAAGTGAAAAAGTTGGTAGTACTGGATATCCGCAATGCCACATCTTCAAGAAGTAGTGATCTGGAATATGTACCAAGTAGCTTGAGGTGGATGAATTGGCCTCATTTTCCTTTTTCATCTTTGCCTTCAACCTACACAATGGATAACCTTATGGAATTGAAATTGCCATATAGCTCCATCAAACATTTTGGGAAAGCATTCATG

GTATTAATTATTTTGTCAATAATATTTGAGTTCTATTATTTTAATTTCAATGTCATCTTATTTATTTGAAATTTGATTATGTTTTCTTTTTATATTACTTGCAGTGCGGTGGATGCTTGAAAAAAATTAATTTTAGAGGCTCCAAG

TTTTTAGTGGAAATTCCAGATTTATCTATTGCAATAAACCTCGAAGAGTTGGATCTTTTAGGATGTGTAAATTTAGTAAAGATTCATGAATCAGTTGGATCTCTTAGTAAGCTCGTCGAGTTTTATCTTTCTAGCAATATTAAGGGCTTTGAGCAGTTTCCATCGTACCTCAAGTTGAAATCCCTTAAAACTTTGTTTTTGTACAGATGTAGAATAGATGAATGGTGTCCTCAATTTAGTGAAGAAATGGATAGCCTAGAAGTATTGTTGATTTACAATAGTACTGTAATTAATCGGCTATCTCCAACAATTGGATATATCACTAGCCTAAAAAAATTGTGGATCATAAAGTGCAAGGGGCTCAAAACTCTTCCAAGTACAATTTATCGTTTAAGTAATCTTACTTGTTTGAGGGTCTTAGGATATAATTTTTCAACCTTTCCTTCTTTAAATGATCCTTCTTCACCTTTCTTATTTCCCTACCTAACATCAATAAAGCTTTTTGATTGTAAGATAACAAATTTGGATTTCTTAGAAACAATGGTTCATGTTGCCCCTTTTTTGAAAGAGTTGGACTTATCTAGAAACAACTTTTGTAAACTACCCTCATGTATTACGAGTTTTAAGTCCTTGATATATCTTTCTACAAGTTTTTGTAAGTTGCTTGAAGAAATTCCAAAGGTTCCAAAAGGAGTACTTTATATGAATGCTACAGAGAGCGCATCATTGGCCAGATTTCCTGACAACATACTTGATTTCATATCTTGTTATGATAATTATGCGGTACGTATCATTTCTTTCTCATGA

>MELO3C004312

ATGGCTTTGCGTCAAAGGGGAATCAATGTTTTTATAGATAATAAGCTTTCAAGGGGTGAAGAAATTTCTGCATCTCTTTTGGAAGCTATTGAAGAATCCAAGATCTCCATCGTTGTAATCTCTGAAAATTATGCATCTTCCAGTTGGTGTTTGAATGAATTGGTGAAAATCCTTATGTGTAATGAATTGAGAGGACAAGTAGTTTTACCAATTTTCTACAAAGTGGATCCATCTCAGGTAGGAAAACAAAGTGGAAGATTTGGAGAAGAATTTGCCAAACTTGAAGTTAGATTCTCGTCAGACAAGATGGAAGCATGGAGGGAGGCCATGATTTCTATTTGTCATATATCTGGATGGACGGTTCTTCAAAAAGA

GTATTTTTCTTTTTCATATATATCTTTCTAACTCTTTTGCTCTTCATATTCTATTGTTATTATATATACTTTGACTATCTCAAGGTGAGAGCATGCTGAGTTAAGGGAAAATCCAAACAAAAAGTGATTTTAAGTTCCCTTCCATAACAATTTTATTTTCAAGTTTTTCATTTTTAAATTTGTATACATTTGTTATATAACTACCAATTTTTTAGTTATCATGAGTTGAACTTTATTAAGGAAACATTTGAATTTTTAACTTTAACTTTATGAGAGATATATATACATACATACATATATATGAGAAATTTAAAATTGTTTTAACAGATATATAATTGTTGAAAATATTTACCTTTATTATAACAAAATTTTAATTTTTACATGTGATAGGTTGCGATAGGTTGCGATAGACTCAACTATGATTTACATTTTTATAAAAAATGAAATTTTGCTATATTTATAAATAACTTAATTACCTTATTTTCCTCTATTTAGTTTTCTTAATTTTAAAAATAGTAGAAGAAGGCATTAACAAATCATAAATTCTTATGGGTGAAAATAATGGCACATGTACTTAATTAATTTTCAAAAACAAAAATTCAAATACTAAAAACTATTTATACCAAGCCATTGGAATTATTTTGTGAAATTGGAATTATTCCATTATTATATTATATTTAATTTGTTATTATATTGTCCTGTCTCCTCCTTTCTATATAATGGATATTATTTGGTTTGATTTAGTTTGACTTATAATGTTGCTTTCATTTTCTTTGGCTTCTAATATTATAGGAATTATAAGTGAATTTCAAAACAACTGTTACTGTATATTTCTTTCTTAAATGGCAG

AGACGAGGCAAATTTGATTCAAAAAATTGTTCAAGAAGTCTTGAAGAAATTAAATCGTGGAGCAATACAGTTGCGTGTAGCTAAATATCCAGTTGGAATAGACAGACAAGTTAATGATATACTCTTCCACGTTATGTCTGCAGATGAAAAAATTACGATGGTTGGATTATATGGAATTGGAGGTATGGGCAAGACAACTTTGGCCAAAGCATTATACAATAAAATTGCTAATGACTTTGAAGGTTGTTGCTTTTTGGCAAATGTTAGAGAAGCTTCAAATCGATATCGGGGTCTTGTTGAACTCCAAAAGGAGCTACTTCGTGAGGTTCTAATGGATGATTCAATCAAAGTTAGCAATGTTGATATAGGAATTAGCATTATAAGGGATCGACTATGTTCAAAAAAGATTCTTTTGATTCTTGATGATGTTGATACGAGAGAACAACTAGAAGCATTAGCAGGAGGACATGATTGGTTTGGACCTGGAAGTATGGTCATTGCGACAACAAGAAACATGCCATTACTTTCTAGTCATGGAATATTTAATAAATTCAAAGAAGTTAACGGATTGAATGCCATTGAAGGTCTTGAGCTTTTTAGTTGGCACGCATTTAGAAATAGTGATCCCTCAAGTGATTATTTAGACCTTTCAAAACGTGCTGTACATTATTGTAAAGGTCTTTCCTTAGCTCTTGAAGTGTTAGGTTCCTTCCTTAATTCTATTGATGATCAATCTAAGTTTGAACGTATATTGGACGAATATGAGAACTTCTACCTAGACAAGGGCATCCAAGATATTCTTCGAATAAGTTATGACGAACTTGAACAAGATGTAAAAGAAATTTTCCTTTACATTGATTTTCCTCGACCTACCCAACTTGACATTGATTCGAGGGCTTTTGAAAAAGTGAG

>MELO3C004313

ATGGAAGCATGGAGGGAGGCAATGATTTCTGTTTCTCATATGTCTGGATGGCCGGTTCTTCAAAATGA

GTATTTTTCTTTTTCATATATATCTTTCTAAACTCTTTTGCTCTTTCATATTCTATTGTTATTATTTATACTTTGACTATCTCAAGGTGAGAGCATGCTCATGCAAAGAAAATGTGACATTTTAAGTTCCGTTGGATAACAATTTTATTTTTTAGTTTTACATTTTTTAAAATTTATATCACTGCCAATTTTTAATAATGAGTTTGAACTTTATTAAGAAAAACTTTAACTTTACAAAAAAAAACAAGATTTGAAAATTATATATATATGTATGAAATTTCAAATTGTTTTAAATGATATAATTGTTAAAGATCTTTACATTATAGCAAAATTTTACTTTTTACAATTATATATATAGGTTTCATGATTTAGTTGACTTAATTTTTAAAAATAGTAGAAGAAGGCATTAACAAATCATAAAATCTTATGGGCGAAAATAATGGCACATGTTCTTAATTCTCAAAAACAAAAATTATTTATACCAAGCCATTAGAATTAGTTTTTTGTAAAAATTGGAATTATTCCATTATATTATCTAATTTGTTATTATATTGTCCTATCTCCTCCTTTCTATAATGGATATTACTTGGTTAAGATTTAGTTTTACTTTATTATGTTGCTCATTTTATCACGCTTCTAATAATATAGGAATTATAAGTGAGTTTGGAAACAACTTTTAGTGTATGTTTCTTTCTTAAAATGGCAG

TGATGAGGCCAATTTGATTCAACAAATTGTTCAAGAAGTCTCGAAGAAATTAAATCGTGGAATACTGCAGTTGCGTTTACCTAAATATCCGGTTGGAATAGATAGACAAGTTAATAATATACTTTTCCAAGTTATGTCTGCAGATGAAAAAATTACTATGGTTGGATTATATGGAATTGGAGGTATCGGCAAGACAACTTTGGCCAAAGCATTGTACAATAGGATCGTTGATGACTTTGAAGGTTGTTGCTTTTTGGCAAAAATTAGAGAAGCTTCGAATCAATATGAGGGCCTTGTTCAACTCCAAAAGAAGCTACTTTGTGAGATTCTAATGGATAATTCGATCAATGTTAGCAATCTTGATATAGGTATTAACATCATAAGGAATCGACTATGCTCAAAAAAGATTCTTTTAATTCTTGATGATGTTGATACGAGAGAACAACTAGAAGCATTAGCGGGAAGACATGATTGGTTTGGACCTGGAAGTATGGTCATTGCGACATCAAGAGACAAACATTTACTTGCTATTCATGAATTTAATATATTTCAAAGTGTTCAGGGATTGAAGGATGATGAAGCCCTCGAGCTTTTTAGCTGGCATGCTTTTAAGATGAGTTGTCCATCAAGTGATTATTTATACCTTTCAAAACGTGCCGTACGTTATTGTGATGGTCTTCCCTTGGCTCTTGAAGTTGTAGGTTCCTTCCTTTACTCTATCGAACAATCCAAGTTTAAACTTATATTGGATGAATATGAAAATCATTATCTTGACAAGGGCATCCAAGATCCTCTTCGAATAAGTTATGATGGACTTGAAGACGAAGTGAAAGAAATTTTTCTTTATATTTCTTGTTGCTTTGTAGGCAAAGATATCAATGAAGTTAAAATAAAGTTAAAAGCATGTGGTTGTTTATGTTTGGAAAAGGGAACAACAAAACTTATCAATCTATCACTTCTTACCATTGTTGGATCCAACTGGATTGAAATGCATGATTTAATACAACAAATGGGTCGCACAATTCATCTTTCAAAGACTTCTAAATCTCACAAAAGAAAAAGATTGTTGATTAAAGATGATGCTATGGATGTCTTAAATGGCAATAAG

GTAAGAACTATCATGCAAACTTCGCATATGCTTAATTATTTATTATGAGAACAACTTTTATTATTTTGAAATTAAAATGACTTGAAATGTTTGCTTTGTAACCTATTGTAG

GAAGCAAGAGCAGTTAAAGTCATAAAAATAGATTTTCCTCAACCTACAGAGTTGGACATTGATTCAAGAGCTTTTGAAAAAGTGAAAAATTTGGTAGTACTGGAAGTTGTTAGAATGCTACATCTTCAAAAAGTATTGATCTGGAGTATGTACCTAATAGCTTAAGGTGGATTAATTGGCCTCATTTTCCTTTTTCATCTTTGCCTTCAACCTACACAATGGATAACCTTATCCAATTCAAATTGCCGTATAGCTCCATCAAACATTTCGGGAAAGCATTCATGGTATTATTTTCAGTCAATAATATTTGAGTTATATTAATTTAATTTCAATGTCGGCTTATTTGTTTGAAATTTAATTATATTTTCTTTTTATATTAATTGCAGTGTGCTGAATGGTTGAAGGAAATTGATCTTAGTTTTTCCACGTTTTTGGAGGAAATTCCTGATTTAACTGCTGCAATAAACCTAAAAAAATTGGATATTGGAGGGTGTGCAAATTTAGTAAAGGTTCATGAATCAGTTGGATCTCTTAGTAAGCTTGTCGAGTTTTATCTTTCTAGCAATATTAAGGGCTTTGAGCAATTTCCAACGTACCTCAAGTTGAAATCCCTTAAAACTTTGTTTTCGTACAGATGTAGAATAGATGAATGGTGTCCTCAATTTAGTGAAGAAATGGATAGCCTAGAAGTATTGTTGATTGATGATAGTACTGTGATTAATCAGCTATCTCCAACAATTGGATATCTTACTAGCCTAAGAGAATTGTTGATCAAAAATTGCATGGAGCTCAAAACTCTTCCAAGTACAATTTATCGTTTAAGTAATCTTACTTATTTAGGTATCTTTAGTTCTGATCTTTCAATCTTTCCTTCCTTAAATGATCATTCTTCACCTTCCTTATTTCCCTACCTAACATCAATAAAGCTTTTCGATTGTAAGATAACAAATTTGGATTTCTTGGAAACAATGGTTCATGTTGCCCCTTTATTGAAAGAGTTGGACTTATCTAAAAACAACTTTTGTAGACTACCCTCATGTATTATTAATTTTAAATGCTTGAAATATCTTTATACAAGTGATTGTAAGTTGCTTGAAGAAATTCCAAAGATTCCAGAAGGAGCAGTTACTATGAATGCTTCAGGGTGCGTATCATTGGCCAGATTTCCTGACAACATACCTGATTTCATATCTTGTAAGTTTTATGAGGTGCGTATCATTTCTTTTTCATGAC

>MELO3C010346

ATGGGTTCTTCTTCTGTTGGTGGAGCAGAATCATCATCGTCTTGTTCTTCAAATTTGAGGTGGAGTTATGATGTGTTTTTGAGTTTTAGAGGAGAGGATACTCGAGACAAGTTCATCAGTCATCTTGTTGTGGCCTTACGTCAAAAGGGCGTCAATTTCTTCATAGATGACAAGCTAGATAGGGGTCACCAAATTTCTAAATCCCTTCTCAAATCTATAGAGGAGTCTAGGATTTCTATCATTATTTTCTCCCAAAATTATGCATCTTCCACGTGGTGTTTGGATGAACTGGTGAAAATAATTGAGTGTATGAGATCGAAGAAACAAAGAGTTCTGCCAGTCTTCTACAACGTTTGTCCGACTGAGGTTGTAAAACAAACTGGTAGTTTTGGTGAAGCAATGGCCAAATATGAAACAAATCGGTTAATGATCAACAAGATTCAACCATGGAAGGAGGCTTTGACCACTGCTGCTACTTTGTCTGGTTGGGATCTTCCAAATTATTG

GTATTATTTTTTTTAGACTTTTTATATTCTTTTTCATATTTCTTTTACTTCTTCCCTCTTAAACGAGTGTAATAGGGGAAGTTGACTAAACTGGCCTAACAACTATAAAAAGGAAAAATAGCCTGCTTTTATAAAAGTCGTGGGCTTTTCTAAAAAGTAAAATTATCTTTTACTTTTCTAAGAACATGATTTCCCTTGTTGAGAGCATGTTGTCTGCTCTGAGACAGACTTTTGTTGATTCTAATTTTGATATTGAAATATTTAATCTCAAGATGAGCTTAGTTCATCGATAATTGACATGATCTTCCATCGTAGATGTGAGATGTTTGAGTCCTCAAGTTGTACTAAAAACAATCAGGTATAATTGATTTAAACGTTTCAAGTATTTAATTTTTAAAATAAGTCATTTTGGAAATTTTTTTATGAAATATTTGGCAACTACTCAAAATAGCTCTAAAACAATTTTAAAGTTTATTTTAAACAATTTTCTTTTATAAAGAAGCACTCCCAAAAAAGGCAAAACAAGTTATAATAATTAAGTTCATAGCACACACAAATTTATTGTCTGCAAAAATGGCAAAAACGAATCCAGCCCACATATAGAAGATGTAAAATGTCACAAATGACTTTGTTACTGATATATGTTTGATACATCTGATACACATCTGATACACCTAATGTTCCTTGTTACACCTGATACTCTACTTAATACCCCCGATATACATTATATACTACTTGATAACCTTTGGGCCGCACCATGATACTCTTTGATACACTCGATACCATTTGATACACTTGAAACACCTTGATACACCTCATACTCCTTGTTACACTTGATTCTTCTTGATACACTTGATGCACTGGTAACAAACTTGTTATGCTTAATTAGTACACTCAACAAATTTGATACGCTTGATGCATGTGATATGTTTGATACACTTGATGTATTGCAAATCCACACTTGTATTATTCACTGATACACTTTGATTAATTAAATACACTTGATATGCTTGAAGTCATACTTATACACTAAAAAAATTGATATACACTTGTAAACTTGATTTATATACTTGATAATGATTCACTTTGTTAATATGTTGATATGCTTTAATAATATGTTGTTGATATACTAGATAATGATACACTGAGTTCATACATATTCTTCATAATACTATAATAAATTGCATAAAATTTGAAAAAATGAAAATCACGTAGTAAGTATATCAACCATATATAATACATATAATATAAGTATACATTACAACACTGATACAAAGTAAACCAAACAAAACAAAACCTATGTAAAAATATATATATATATATATATATATATATATATATATATATATATATATATATATATATATATATATATATTTCAATCAACAACCATTATATTTTATATTACAATTAATGTACTATTGATATTCTAAAATCAAGATTAAGATGGAAAGAACAAATCGTTTTTTCCATATAAGAAAAGATGATTAAAGCCTAAAAAAGTGCGAGAAGAAATAAGGGATGATTAAGGCATTAAAAAGAGGAGAATAAATAAGTTATATATTGAATGGTAACCTGAGAATAATAACAAATTTAATATGGAGAATGTTTTAAAAAGTGAGAATTTTTCCATATTTGCAAAATTGTAAAACAACGTGGTTCTCAAAATGCTACCTATTGCAATTTCTCTTTTTATCAAAATAGCTTAAATAAAAAAAATAACATTTTTCAAAATATTTTTTTTTCTTTAGTCGATACAAATTACCATATATTTATTTACTATTTCTCATTTTATTTTTCATCACTATGTGTCCGTGTATAATGTGTAATCAAATGATGATAG

GAAGAATGAAGCTCATCTTATTCATGACCTTGTAGAGAAGGTGTCTATATTAAAACAAACACAATTACTAAATGTAGCCAAGCATCCTGTTGGAATTGATTCTCAACTTAAAGCTGTTGAGGAATTCGCCTCCCATGGTGTGTTAGATAATGGTGTCAACATGGTGGGGATACATGGGATGGGAGGGATTGGTAAGACAACTTTGGCCAAAGCTTTATACAACAAAATCACATATGAATTTGAAGCTTGTTGCTTTCTTTCAAATGTTAGAGAAGCTTCAGAGCAATTTAATGGTCTAGTTCAACTGCAAGAAAAATTACTCAGTGAGATCTTTAAGGATAATAACTTGAAGGTTGACAATGTGCACAAAGGAATGAATATCATGAAGGATCGATTGTGCTCAAGGAAAGTTCTTATAGTTTTGGATGACGTGGATAAGGACGATCAATTAGATGCATTGGTAGGTGGACGTGATTGGTTCGGTCGTGGTAGCAAAATCATTGTGACAACAAGAGATAGACATTTACTTGAAAGATATTCATTTGATAAAATACATCCTATTCAATTGTTGGATTATGTCAAATCTCTTGAGCTTTTTTGTTGGCATGCTTTTAAGCAAAATCATCCATCAAGGGATTATTTGGACCTTTCAGAACTTGTTGTACGCTATTGCAACGGTCTTCCTTTAGCTCTTGTTATTTTGGGTTCTCTACTTTGTAAGAGAGACCAAAAAATATGGAAAAGCAAATTAGATGAACTTAAAAACTTCCCGGAACCAGGTATTGAAGCTGTTTTCCAAATAAGTTTTAAGAGGCTTGAAGAAAATCCCCCAGTAAAGGAAATTTTCCTTGATATTTGTTGTTTTTTTGTGGGAGAGGATGTTAGCTACAGTAAGAATGTGTTAAAGGCATGTGATCCTTATCTAGAATCCAGAATTATAATTCTCATGGATCTTTCTCTTGTTACGGTTGAAGATGGCAAGATACAAATGCATGATTTAATTCGACAAATGGGTCAGACGATTGTACGTTGTAAATCTTCTAAGCCAGAAAAAAGGAGTAGGCTGTGGGTGGCAAAAGAAGCTGTCAAGATGTTGATAGAAAAATCC

GTGAGTAACCTTATGTAATTATTTTCGTTTACTTATGTCCAAAGACATTGTGATCACAAAAAAAACAATGGGGGTCAGCTTGATTATTTGTTAATAATGTTAGAATGACTTCATATTTAACTTTGTAG

GGAACTCATAAAGTTAAAGCCATAAAGCTAGACTTGCGCAGCAACCGTCCATTGATTGTTGAAGCAGAAGCATTTAGAAACATGGAAAATCTTAGGTTGCTTATCCTTCAAAATGCAGCAAAATTTCCTACAAATATATTCAAGTATTTACCTAATATTAAGTGGATTGAATACTCATCATCTAATGTTCAATGGTATTTCCCTATAAGCTTTGTTGTGAATGGCGGGCTAGTTGGACTAGTCATAAACGGTGTATCCAACAAACATCCAGGGATTATATTTGAG

GTAATTAAATTTTACAACTTCTACTTGTGTGCTTGATATCACTGCAAGTGTTTTTAATGAATTTTTCCGTGTGTTTTAG

GATTGCAAAATGTTGAAGCATGTTGATCTGAGTTATTGGCGGTTATTAGAGGAAACTCCTGACTTCTCTGTAGCATTAAACCTTGAAAAATTATATCTTAGAAGTTGCAAACGTTTGGAAATGATTCATGGATCTATTGCTTCTCTTAGTAAGCTTGTTACCTTGGACCTCGAAGGCTGTGAAAATCTAGAAAAGCTTCCAAGTAGCTTCCTCATGTTAAAGTCTCTTGAAGTTTTGAATCTAAGTGGATGCATAAAGCTGAAAGAAATTCCCGATTTATCGGCATCGTCAAGCCTTAAGGAACTACATCTTAGAGAATGCTATAATTTGAGAATAATTCATGACTCTGTTGGACGCTTTCTTGATAAACTTGTTATTCTAGACTTTGAAGGATGTAGAAACCTTGAAAGGCTTCCAAGATACATCAGCAAGTCAGGGTCTATTGAAGTTTTGAATCTCGATTCATGCCGAAAGATCGAACAAATTTTTGACAACTATTTTGAAAAGTTTCCAAGCCACCTCAAGTATGAATCCCTTAAAGTTTTGAATCTTAGTTATTGTCAAAATCTTAAGGGAATTACTGACTTTTCATTTGCATCAAACCTTGAGATATTAGATCTTAGGGGCTGCTTCTCTTTAAGAACGATTCACGAGTCCGTTGGATCTCTCGATAAACTTATTGCCTTAAAACTTGATTCTTGCCATCTACTTGAAGAGCTTCCTAGTTGCCTCAGATTGAAGTCTCTTGATTCTTTGAGTCTCACTAACTGCTATAAGCTTGAACAACTTCCAGAATTTGATGAAAATATGAAGTCTTTGAGGGAGATGAATTTGAAAGGTACAGCCATAAGGAAGTTACCCTCATCAATTAGATATCTTATTGGGCTTGAGAATTTGAACCTTAGTTATTGCACAAACCTGATTTCTCTTCCAAGTGAAATTCATTTGTTAAAGAGTCTTAAGGAACTTGATCTTCATGAGTGTTCTAGACTCGACATGTTTCCCTCGGGATCAAGCTTAAATTTTCCCCAACAAAGCTTATTTTCAAACTTGACTATATTGGATCTACAAAATTGCAACATATCAAATACAGATTTTTTGGAAAATTTATCTAATTTCTGCACTACCTTGAAGGAGCTAAATTTGTCCGGAAACAAATTCTGTTCTCTACCTTCCCTCCAAAATTTTTCATCATTGAGGCATCTTGAACTAAGGAATTGTAAGTTTCTTCGAAACATTGTGAAGATTCCACATTGTTTAACCCGAGTGGATGCTAGTGGTTGCGAATTGTTTGTAATAAGTCCTGACTACATTGCCGATATCATGTTCAGAAATCAGGTTCCTCTGTTTTAA

>MELO3C008730

ATGGTGGACGTCCCATCATCTTCGACCACCAGACGGTGGATGTACGACGTCTTCTTAAGCTTTCGAGGTGAGGACACTCGTCAGAATTTCACCAAACACCTTTACGACGCCCTCGATACTGCCGGAGTCAACACGTTTCGTGACGACGTTGAACTCCGGCAAGGAGACGCCGTGGGCTCAGAGCTTGTGGTAGCGATTAAAAAATCGAGGATAGCAGTCGTGGTGTTCTCCGATGGCTATGCCGACTCACAGTGGTGCCTAGGGGAGATCGCCGAGATCATGGATTGCCGGACTGTTGAGGGCCAACTGGTCCTTCCGATCTTCTATGAGGTGGATCCGTCGGACGTTAGGAAGCAGAAGGGGAGGTTTGCGGCCGCATTTGAGAAGCACGAGAAGAGATTTGGTGTAGATTCGGTGGAGGTCCTGCGGTGGAGGGCGGCGCTCAGAGAGGCCGCTAGCTTGTCCGGCTGGGACTTGAGGCAGCTAGCCGATGG

GTACGTATTATTATTGTTATTTTCCATCCTTTTATTTACCTTTCCTTGAAAATTGCAAAAACAATGGGCAAATGGGAATAATAAAAATAATAGATTAAAAAGAATGAGGAATCGTCAAAAACGTTGGAAACTATTTACGTATATATATATATATATAAAACTCGGATGTGTTAATAGAAAATTTGATAATTTTGTTGTTATGCTTAAATAGTTTCAATTTTTTTATTATATTTGAAAATGTCTTAAATATTTTAATTTAATTTAATTATCATGAACAGAAAAGAAGAATCAATTACAAAATATTTAAATGCCCCTTATAATTACTTTTTGAAATTTACGAGCAAGATATAAAACTAGATCAAAGCTATAACTAAAAAATAATTTTTTTTTTTACTTGAATGAATAAAATATCCCAGAATTATTCCAACCTAGTTTAACTAGCATAACTGAGGTAATATACATCCGCGGTGGATCCATAAAATATATTGATAGAGGACTAAGATTCGAAATTGAGAGAGACTCGAGCCCTCGAACTTATACTAAATCCTCTGTGTATATATCAAATTATAATACTATGATTAATATAATGGATTCAAGTTTTTTCTTTTTGAATTTTGTTAAGTTGGGGTTGTTTATGAAAAGTTTTTGGAATAAAATTAAAGCTGTATGGTTTTCACTTTCTCTGAATATTTTGGTTGAAGAGACATATTTCAATTTCATCACATTTTAAATTTTCGTGTATCAATTTGATATATAAGAATATATTTGACACTCATCTCAAATGGACTCTTACAACTTCAAATATTTTAAGTTAATCCTATATTTTGGAGTTCATTTATTTATTTTTTTTTTTTTGTAAATTAAAAGATAAAAGTTGTTCTCTCTTCAATTAGATTAAATCTTAAAATTTTAAGTTAATTAAGCTGAGCTCACGAATAAGAAAAATATATTTTCAAATTCATATAGGTGTTGGGTTAATAATATATGACTTGAGAGAGGGAGGTTGTTCTCTCGCTATATTTTCCAATTCTATTTTCAATACAATTTAGGTTAAATCAAAATTTAGGCTTTATTATGTGGTTTGACCTTTTCTTAATTTAGAAGTTTAAAATGTATTTTAATTCATAGTCCAGTTGTGATTTTTTTGGCCTTTTGTTTGATTTTGTTATTTCTCTTGTTTGTTTTATGGACCAATGATCTCTTACAATTTTCAGAATTGTCAAGAGGTTTGAATAAAAAAAAAAAAAAAAACAGATAACATCTATTAGTGTCAATTGAGATGGATGATGCTAGTGTATCATTGATTATTGTTATCAACTATGAGACTTTTACAATTTATAATATTTGTAAATTAGGTTAGTTTATTTTCTTTCTATATTTAAAAAATATCTCAAAATTAATTTAATATTTAATTTTACACTCTTAATTAATCATATTTTTACATAGTATTGAAATGAAAATTGGATTAATGAAAGAAAAAACATGTTCTAAATTAAGTAATTGGAAATCCACTCCAAATTAAACCTGCCATTCAATTTCACAATCAAGTTCCCGGCAAGAAAAATACAAATGTAGAAGAAAAGTTGTTGCCATTATAGAGACAAGAAAGTAAGCAAGGAATAGGATTTTGAAAATTCGACTTTTATTGTTCAATTGATTTATGGTTTTGAAAACTATGCATTGAATTAACATAACAAGAACCAAAAACATAAAGAAAAAAATAATGACAAAAGAAAACTATTCGAAACTCTTCCTTTATTTTTCTTTTTTCTTTTTTTTTTTCTTTTTAATAAAGAAAGATGAAACACGAAATAAAAGATGTAATGAGTTGTTTAAAGAAAGCTCCTTGAAGTTTCCAATTGGTGAATAGTACGTACTTGGTTAAATATGTTTAATATAATATTACTAACATTTCAAAAGTCAAAACTTCTTTCTTTTATTTTAATTCTGAAAACATTTTCTTTCTTAAAATATTAGTTTCCTTCTTATATCTCAATCATTCTTTCATTTCAATTCTTATAATTTTTAAATAATTCATTTTAGTTCAAACTCACTGTCCATTAAATATGATTAAAAAAAATCATATTTGTACTTTCAATTTCTCTTTCTTTCTTTCTTTATTTATTTTTTCATATCATCTTCTCCATCAAAAGCTTTCCCTCTTGTTCTTCTTTCTCTACGAATTTTATTAGTAGGGGTTCTGCAGGGAGGCCATAATTATATTTATGGGCATGACAGCCAACTGAGGTGCTGGAGAAAAATGCGTAATAATAACTTGATCGGTGAGCGGAAGCTATGAGACTGTTTTGCCATTGAGCTTCTAGCTAGTATTATTATTTTTTGGTGGATTTTTTTGATGCTAGAAGATATTGATCATGTTAACGGATTGGTTAATTATATATCTTAATGGCAAAAAAAAATAATATATATATATATTCTGATTGTCGACATTGACAACAG

GCATGAAGGAAAGTTCATAACCAAAATAGTGGAAAGGGTTCAAAGCGAACTGAGAGTGACATATTTGGAAGTCGCCATCTACCCCGTTGGCATTGATGTTCGTCTCAAACACTTGATCTCATTAATGGCCATTTCTACAAACCACTCCACTCTCGTCCTCGGCATCTATGGCATGAGCGGCATTGGCAAAACCACTCTCTCTAAAGCACTCTTCAACCACTTCTTCCACTTCTTCAATTCTAGATCTTTTCTCCCCAGCATCAACTCCATCTCTAACTCCTCTCCCGACGCTCTCCTTCGCCTCCAACAAACTCTCCTCTCCGATCTCCTCATCGCCACTAACCTCCGCTCTCGTTCCTCGACCACCACCGACTCCACCGTCGTTCGGATGCAGGAAAGACTCCAAAACAAAAAGGTCTTGGTTGTCCTCGACGACCTGGATCGTATCGAACAAGCAAATGCGCTAGCAATACGGGACCCAAGATGGTTTGGAAAGGGAAGCCGAATCATAATCACAACAAGAAACAAACAAATCTTGGACATTCTAAAAGTCGACAAAGTATACAACATGGAATCCAATCCACTGAACGACGAGGAATCATTGGAGCTTTTTAGCTACCACGCATTCCGGGAGCAAAATCCACCAGAGGAGCTTTTGGAATGTTCGAAATCCATCGTTTCGTACTGCGGAAATCTTCCTCTAGCTCTGGAAATCCTGGGTGGGTCATTCTTCGGAGGGAGACCGATGGAGGAATGGAGAAAAGCGTTGGAGAGACTGAAGATGATTCCGGCGGGGGATTTGCAAGAGAAGCTTCGATTAGGGTTTGAAGGATTGAGAGATGAGATGGAGAGGGAGATATTTCTTGATGTGTGTTGCTATTTTGTGGGAATGAAAGAGGAATTGGTAGTGAAGATTATGGATGGATGTGGAATGTATGGAGAAAGTGGATTGAGAGGATTGAAATGGAGGTGTTTGGTTGGTGTTGAGATTTGGAGTGGAAGGTTGAAGATGCATGATTTGGTTAGGGACATGGGGAGGGAGATTGTGAGGCAAACTTGTGTGAAGGAACCTGCTAGACGATCTAGGGTTTGGCTTTATCATGAGGCTCTCAAGATCTTACTCCATCAAACC

GTAAGTTAATTACTTCACTCATGCATTTTTATAGTATATAATTAATATGTAGTGAGATCATTGATTTAACAG

GGAACTGAAAACATTGAAGGACTTGCAATTGATATGGGCAAAGGAAACAAGGAGAAATTCAAATTGGAAGCATTTGGGAAAATGAGAAATCTAAGGTTACTCAAACTCAACTATGTGAATCTCATTGGAACTAATTTTGAGCAAATAATAAGCAAAGAATTAAGGTGGATTTGTTGGCATGGATTCCCTTTGAAGTCTATTCCAAGCTCATTTTATCAAGGAAACCTTGTTGCCATTGACATGAGGCATAGCAGCTTGATACATCCTTGGACTTGGAGGGATTCACAG

GTAAATTAATACAATTAATTAGCATTTTACTGATTTTATTTTCTAAATGATTGTTCATCTTTTTTTTTTTTTTTTTTTATTTAGAATAATTACTTTTCTCATCCCTTCTAAGTTTAGGGCTTAGTTTTCATTGATGCTCCTTCAATTTCAAAATCCTATACTCTTAGTTTCTTTTAGTTTGATTGCAATTTATTAGTCTCTAGGTTTAAAGATGATACCATTTTTACTTTTGACGTCTGAGCTTTGCATTTCAATTTTATCTCTAAATTTCAAAATATAATTACATTTTTTATTTAAATTTTTCAATAAGTAATCATTTAATTTTAATATTATTTTTTGTTTAGTATTCAAGATTGTATACGGTAGAGTAGTAATTCAAAAGCTTACGATTTTTAAAGTCAAAAGTAAATATATTTGTATCCAAGTCAGTGATGTCAAGTAAATTTAATCCTTAAAAATCAGAATATATATTTTGTTGAAATGCATTGATTATGTTATTTAGAGATTTAAACGACCAAATACGAAAAAGATCTCGGATATAACTTATTTTATATTTGTATTTTAAGTTCTCTTTAACAAAAATGTTCACGTGGATGTAGCTAACGTATTATTAGTAAATCACGCATCTGTGTGTTAACCTGTACTAATTTATGCTTTTATGTTTTCTTTATTAATTATCGATTCCATAACATTTTTAACTACTGCGTCTCTCTCACTCAAGTATGTACTTGTTTTGGGCAG

ATTCTTGAGAACCTAAAAGTTCTAAACCTAAGCCATTCCCAAAAGCTAAAGAAGTCCCCAAACTTCACAAAGCTCCCAAACCTAGAGCAGCTAAAACTCAAGAACTGCACAGCCTTATCAAGTCTCCACCCCTCCATTGGCCAACTTTGTAAGGTTCATCTCATCAACCTCCAAAACTGCACAAATCTTTCGTCTTTACCAACCTCCATCTACAATCTTCACTCCCTCCAAACTTTCATCATCTCTGGCTGCTCCAAGATTGACCGCCTCCACGACGACCTCGGCCACCTTGAATCCCTCACCACCCTTCTCGCTGACCGAACCGCCATATCCCACATCCCTTTCTCCATTGTCAAGTTGAAGAAGCTCACTGACTTATCTCTATGTGGTTGTAACAGCAGATCAGGGTATGTTAATCTTTTATTTTGTAACAATAATATATATGTTAGCTATATGATCAATATAATATTAAACTAAT

>MELO3C009850

ATGGCCGACGAGCTCCGACCTCGACACGGGAATTGGACTTACGATGTTTTCTTGAGTTTTAGAGGTGAAGATACTCGCAAAAACTTCACTGATCATCTCTACTACGCATTCAAAGATGCAGGCATCAATGTCTTTCGAGACGATCCAGGGCTCGAACGGGGTGAAGACATAAGTTCGGAGCTGGTGCGAGCTATCGAAGGATCGAAGGTGGCAGTTGTCGTATTCTCGGAAAGGTATGCGGAGTCGGGATGGTGTTTGGAGGAGTTGGTAAAGATCATGGAGTGTAGAAGGACTTTGAGACAACTGGTTTTCCCAGTATTTTATAATGTGGATCCTTCGAGTGTGAGGAATCAAAAGGGTGAATTTGAAGAGGCTTTTGTTAAACATGAAGTGCGTTATTTTAGGGATATTGATAAAGTTCTTAAGTGGAGAATGGCTCTCACTGAAGCTGCTAATTTATCTGGTTGGGATTTGAGAAACATTGCAGATGG

GTATTTATTAATTCCTTCCTTTCTTTTAAATTTAAAACAATAAAGTTTCAATAAATATTGTCCATTTTGTTTCAATGTTTTTAGTTTAGTCTCTACTGTGTGAATATATTAAAATCTGGTTGTCGAACAACTTTCTTAGTGTATTACTTTGTTCATCAGAATAGAAAAGTTTCTAAATAACCTTTTTGGTTTCTTAATGTATTACTTTGTTAATTTAAGAATTTACATAAACATATCATATACCAACCATTATATTTCAGAAGATGGATCTTCTCCTAAGTTTTGAAGAGTAACTTATCTGGATCTTGTTATATGGCAG

ACATGAAGCAAAGTTCATAAGGTTGATTGTTGAAAAGGTATCAAAGGAGGTGAACAGTAAATACTTATTCATAGCTCTTTATCCAGTGGGAATTGAATCGAGAATCAAACCTCTTTTATCACATCTTCATATTGGTTCAAATGATGTTAGGTTTGTAGGAATTTTGGGGATGGGAGGACTGGGTAAAACCACCATTGCAAAAGCACTTTACAACCAACTTTATCACAACTTTGAAGCCAAATGTTTCCTTTCCAATATCAAAGCTGAAACCTCCAACCAACCCAATGCTCTAATTCACTTACAAAAACAACTCCTCTCTTCCATCACAAATTCCACCGACATCAATCTCGGAAACATCGACCAAGGAATCACCGTGTTGCAAGAAAGACTTCGTTGCAAAAGGCTTCTTTTGATATTAGATGATGTAGACGAAATAAGCCAGTTAACTGCATTAGCAACAAGGCGTGATTTGTTTGGTTCAGGTAGTAGAATTATCATAACAACTCGAGATCAACATCTGCTAAATCAGCTCGAAGTAGACGAAATTTGTTCCATCGATGAAATGGATGACGATGAAGCACTTGAACTCTTTAGTTGGCATGCTTTTCGCAATAGTTATCCATCAGAAACCTTTCATCAACTTTCAAAACAAGTGGTCACGTATTGTGGAGGATTGCCATTAGCTCTCGAAGTGTTGGGTTCTTTCCTTTTTGGTAGAAGTAGAGAAGAATGGGAAGATACATTGAAGAAATTGAAGAAAATCCCAAACGATCAAATTCAAAAAAAACTTAGAATAAGCTTTGATGGGCTAAATGATCATACTTACAAAGATATATTTCTTGATGTGTCATGTTTCTTTATTGGAATGGAAAGGAACTATGTTGAACAAATATTAGATGGATGTGGATTTTTTCCAAGAATTGGAATTAGTGTTCTTCTTCAAAGATGTCTTTTGACAATTGGTGACAAAAACAGATTAATGATGCATGATTTGTTAAGAGATATGGGGAGAGAAATTGTTCATGAAAATTTCCCAAAATGCCCTGAGAGACATACAAGACTTTTTCTTCATGAGGAAGTGCTTTCTGTACTTACAAGACAAAAG

GTAAGGAAAGAAAGTCCTATTTAATTTGATCTAATTTTATTTTTTAGTTTAATATTTTTAATTTCATTGTTTTAG

GGAACTGAAGCAACTGAAGGCCTAAGTCTGAAGTTGCCAAGATTTAGCAAGCAAAAGTTGAGCACAAAAGCATTTAATGAAATGCAAAATTTGAGGTTACTTCAACTTAATTTTGTCGATGTAAATGGAGATTTCAAGCATATTTCTCAAGAGATAAGATGGGTTTGTTGGCACGGATTTCCTTTGAAGTTTTTGCCTACAGAATTTCATATGGACAAATTGGTTGCGATGGACTTGAGATATAGCCAAATCAGATTCTTTTGGAAGGAGTCTAA

GTACATATTTTACTTTTGAATTCAAATGTTTTACTTTGGTCCTACCCTAGAATATAGCTAAATCAGATTCTTTCAACGAGTCTTTATCACGGCTAATGTTAAAGTAGTGTGGATATTATTAAATTTACCATAACTAATCAACTTAACATTTTGAATCAATCACAGCAATTTAACATATAGTATCACAGCAATGCCATTTTCTTCTCAATCAACAATTTTTCCTGATCTCTTTGTTCTCTTTTGTGTGAACATGCAG

TTTCTCAAGAATTTGAAGTTCCTCAATCTAGGCCATTCTCATTACTTAACCCACACTCCAAATTTCTCCAAACTCCCCAATCTAGAGAAACTCAGCCTCAAAGATTGCAAGAATTTAATTGAATTGCACCCTACAATTGGAGAATTAAAAGCCCTCATTTCCCTGAACTTAAAAGATTGCAAATCCCTCAAATCACTTCCAAATAGTTTCTCCAACTTAAAATCTCTACAAACTCTCATTATTTCAGGTTGTTCAAAGCTTAATAGTTTGCCAGAAGATTTAGGAGAAATTACATCATTAATAACTCTAATAGCTGATAACACACCAATCCAAAAAATTCCTAATACAATTATAAACTTAAAAAACCTCAAATATTTATCTTTATGTGGGTGTAAAGGGTCACCATCAAAATCATCATTCTCTTCAATGATTTGGTCTTGGATTTCACCAAACAAATTATATAAAAACTACTCATCAATTATTCTCCCTTCTTCATTACAAGGCTTAAACTCCTTAAGAAAATTATGCCTTAAAAATTGCAACTTGTCAAATAACACAATTCCAAAAGATATCGGGAGTTTACGTTCTTTGAGAGAATTGGATTTGAGTGAGAATTTATTCCACAGTTTGCCATCAACTATTAGTGGCCTTTTGAAACTTGAGACACTTTTGTTGGACAATTGCACTGAACTTCAATTTTTACCAAATTTGCCACCACATTTGAGTTCATTGTATGCATCAAATTGTACTTCATTGGAAAGGACTTCAGATTTGTCAAATGTGAAGAAAATGGGATCTTTGTCTATCAGTAATTGTCCTAAACTTGTGGAGATTCCTGGCTTGGACAAATTATTGGATTCTATTAGAGTTATTCACATGGAAGGATGTAGCAACATGTCCAATTCCTTCAAGGATACCATTCTACAGGTTCTAATCTCTCTCTATCTTTAAT

>MELO3C022154

ATGTTCCTTTCTTCTTCTTCTTCTTCTTCTTCTTCAATTGGTAAATGGAAATTTGACGTGTTCTTGAGCTTTCGAGGCGAAGATACACGTGGCGGTTTCACGGATCATCTCTACAAAGCCTTAACACAAAAGGGAATTTCGACATTTAGAGATGAAAATGAGATCCAAGAAGGTGAAGACATTTCTTCAAATCTGTTGGATTCCATTGAAGCCTCGAGATTTGCTATCGTTGTGGTTTCGGAAAATTATGCATCTTCAAGATGGTGCCTGGAGGAATTGGTTAAGATCTTTGAATGTGAAGAGAAGCTTGGAATGGATGTTTTACCAATTTTTTATAAAGTGGATCCTTCTCATGTGAGAAACCAAAGAGGAAGGTTTGAAGAAGCTTTTGTTAAACATGAAATGAGATTTGGAAGAGATGATATTAAGGTTCAAAAATGGAGGAGGCTTCTCACCAAGCTTGCTAACCTCAAAGCTTGGCTTTCTCAATCTTG

GTAATTAACTTAACTATTTTCTCATTTATTTTCATTTTTCAGATATCTACTTTTTTTATATATATAATTTTTTTTAGAAATAAAGTATCTTCTCTAATTAATTAAAATCTTAGACAAATTACGAAATAAATTATCTTAGTATATCGTAAATAGACAGTGATATTTTGATATATTTCGTAGATATTGTGGTTTATCTTGCTATATTTGAAAATAACGCTTAGATGTGGCCAATAGTCAGAAAAGTTATACATGATTACGATGTTTAGCGAGTTCGATTGAAATTTCGTGAGAGAAATTTAAGCTAAAAATTTGGTCCATTTTTTTTAACTTTTTCTTCTAGTTTTATTTGTCTTCTAGATTTCTTTGACCTTAATTAATATATGCATTTTAGTTTAATTTTGTGCTGAAAGTTTGATTGAAATTTACAAAATAAGAGTTCTGTATATATTCGATAATATCGATTAAGGCTTTTCCAGACTAAAGAAAATGATTAAAATTCATAGGTCCAATATACACAGTTCTATACATTTTGGGTTTCTTTTTTTCCTTAATGTTTTTTCATTTATAGAACAAGTATTATCAAATACATAAATTTTTACCTTTTGATCTCTTCCATCTAGTTTAGTTATTTGTTCTTTAAATTGAAATAATTGTACTATTGTCCCAAGTTTGAGACCTAGAATGGAATCAAGTTTTACTTATTTTTAAAGCCTTTTAAGTGTTTTGACAAACCTTTAGCTTAGCTTGTGTTTATCTGTTTGTAAAAACTTATTGAAATGAATGATTGTGTATTTTTGTTGGGCTCTCAACACTCTCGACTTTTGCTACTACACACGTCAACGCTCTTGATCCTTACACTTAACACTCGCCACTATAAATTACTTTCAATAATAATATAAAAGGCAAGATTTCGAGGAGCAAGGAAACGGAAACAAGGAGCAAGAGCTAGCAAGGAAAAAAGAAAAGAAACGTGAGCAAAAATGATGGGCGAGAGAATAGAGACAATAAGAGCCTAAAATCCTAAACCCTAAGATGCAAGATAAGCAAGAACAAGGATGGATGGAAAGTATGTTTCTGATTAAAAAAAAAAAAAAAGAGCAAGAACGGAAGGAATTAGCCAGAGAATGGGATCTCACTCGAATGCATAATATTATGTTTCGGTCATTTCCTTGTTGATGATCACACCAAGCAAAAAAGTCAAAAATTAAACTTTGGTATAGCTTGGGTGCATCCATCCTTTGTGCAATCCGACTAGCACCAGGTCAATAGAAATATGTATCACATTCTACTTTTGTTTTCTATTTATTATGTGTTAAAATTATATCTTTCATATGTAAAAGATAGATCAAAAGCATGAAACTAGCTAGACCGAACCTAATCATGGTTAAAAAACCGAAAAGAATTAAACACGTAATTTCCTATAAACTTATGAAGTAAGCTTTCTCTAGTCACAATTGCTCATCCACACGTCAGAAGGGCACAATGGGAATTAGGTTATGTAGAATGTACCAAGTTGAAACATTTTAAATATTAGGGACAAAAAAGTTGATAAATATCTCAAAGCAAAAACTGTGTAAAGACTTTTCATACCACCCAAAAAGCAATGTAGTTAGACTCAGTCAACAAACCACTCATATATACAAAAGTAAACTGCCCTCATATTGAATTATTTAATTATAACTATCAAATGTTCTTGATTGTGATTGAACTGGAAAAAAAAAAAAAAAAAAGATTGAAAGTTGCATTGTAATTGTAAATATAATATTTTAAAGAACCATTATTTATTAATAAATAATTAGCATCTGTGATAATAATAAACCCAACATGAGTTTTATTTATGTGATAGTGTTATATACGTTAACATTATTCAATTATAAATTATGTGTATTAATTAGTCATTAACGATAATAAGGTTTTGCTTTTGATGATCACCATGGATTTGAATTTAGGGAGGTTTGACTTTTGAAACCAAGTCCCATGTGTGGTATGAGGTTTATTTTGCATAGATGTCTTGAGGGTTTGAAAAAAAAAATTCATTCGCAAACAAGAGTTTAGCTTACATTTAGTGTAAGGTGAGTGAAACTGAAAGGTCCATGGTTCAAATCTACTTACGGTTAATTTATACTAAAAATTTTGAAAAAAAAAAAAAAGGTTCACTTCATTCACTTTCTTCACTTCCCATGCATACCTCTCATGAGTGGCTGCCAAAAGCATAAACGATCTCTCCTTTGTCCCGAGAGATCCAATTCATGAGTTAAGGTTTAGGTTTAGGGTTTAGACCACGTTGGTCTTAGTTTGTCATTTATGCCTTGTCGATGGTTCATGGTTGTTGATTTGATTGGTTTGTTGTTTGAATAATTCATTTTTTTTTGTCTGTGTCCTCAATCATTCTACTTGCTTCGTTGATGGTTGAACTTTCCTTGGATCCTTTTTATGGGTTGTCAAATATGCATTGTTCATTAGTGTTGTTGGTCGTAGATCATTTGTTGCAATTGTCTGTATCCGGTGTGGTTTCAATAATCAAAATTCTTTGTGATCATGGATCACTTGTATGATTTTATCTAGTATTATAAAACATATAGAGTGGTCAAAATGAGAAATGAGAAGAGAAATAAAAAGAAAAGAAAAGAGAGAAGAATAAAGAAGAAGAGGAAGTGTCAATAGTTTACGAGAGGTAGAAGAAATAAAGGAATTGTTTTATGGTTTTTTAAAAGGGGCTCTTGTAAATTTAGCAAAATTAGTTTGATAAATTAGGTTCATAACACACTATTTTCAGATTTGCAAAAATGGCAAAATTCAAGCCCAGCTTACATTTGAGAAATGAAAATTACAAAACTACCCACTAGAATTATCAACATTTTTATGCATCACTTGATGTGTTGTTGATACACTTGTTATGCTTTGCTAATACACTCGATGAATTAAATAACACTTGATACACATCATTGGTTTGAGATATACTTGATATGTTGTTGATACATTTGTTAAACTTTGCAAATACACTTAATAAATTAAAGACACTTGGTGCACTAAGCACGATACATTACATTGTCGATATACTTGATTAGGTTTAACACACTTAATAAGTTTGATACACTTAATAAGTTTGACACACTTAATACACTATTGATAAACATTTTATACTTCACCGATACGCTTGACAGATTTATTACACTTGTTGCATTTCATATGTTTGAGACCCTTGATCGATATACTATTGGTACATTTTGCTATATCTACTGATATGCTTAATTGATTAAAATACACTTGATACACTTGAGGTTCTGATGATACACTTAATATACTTCACTAGCATACTTTGATACATATTGTATGTTTCCTACACTAAGTTACATCATTCATATACTTAATAATACTACATTGAATTGCATAAAATTTGAAAAAAAATAATGGAAATCATGTAGTAAGTATATCAACCAACTAATATATATAATATAAGTACATCACAAAACTAATATACAAAACTGATACAAATTGGGAGAAAAAATAATGATTGGGGTCTTAAAAAGTGGGAGAAGAAATATGAAATGATTAGGACATTAAAAAAAGAGAAGAAATAAGTTATTGAATGATAGTTTGAGAATAATAAAATATTTAAGATGGAATGTATTTTAAAAATTGAGAATGTTGTCATATTTGAAAGCAACCATTTGAAAGAAATTTGAAGGAAGAACTTGGATTTAAAAAAAATGATGATTGAAAATCTTGATAAAAAATGGTGAAATTAAAAGAAGGATGAGATGAAGATGCCGAAAACAGAGATTGAGCAAATAATAGTTTTTTTCTTATTATTTTTTTAATCTTTGAGAGAACCTACAGAAGACCAACCGAAACATAGTGTTTGAGAAAAACGAATATTTTTTATTATTAGATTAATTTTTCATTATGAATACAAATATGATACAAAGGTTTACAAAAAAGAAGAAGAGTTAATTAATGGAAGATGAAGCATTAGAGGGAGATGATCAATAGAGAGAGAAGAATGTATAGGACAGTTTGGGAATAATCAAAAANNNNNNNNNNNNNNNNNNNNNNNNNNNNNNNNNNNNNNNNNNNNNNNNNNNNNNNNNNNNNNNNNNNNNNNNNNNNNNNNNNNNNNNNNNNNNNNNNNNNNNNNNNNNNNNNNNNNNACAAAGGTTTACAAAAAGAAGAAGAGAAAATGGAAGATGAAGCATTAGAGGGAGATGATCAATAGAGAGAGAAGAATGTATAGGACAGTTTGGGAATAATCAAAATTTTAAAATATAAAATATCTTCAAAATATTTTAGAGATTAAAATTTGTCATATTTGCAAAATTTGAAAAGTATGTGTCATAGTTACTAAAACCTATTCTCAACCCATTACAATTTTTATTGTATTTAATTACATCAATTATGGTAAATTTGAAATGATGTTTGTGATGGATTTATGTATCTTCATTGTTTATTTTCTTGAAATTAG

GTCACATGAATCAAATATCATTGAAGAAATCACGACAACAATATGGAAAAGATTGAAACATAATTTGACAGTCATTAAGGAAGACCAACTAGTTGGAATCAATTCTAAACTAAACAAACTTTCTTCACTTTTGATCCCAAACTCTGATGAGGATGAGGATGATGATGTGATCTTTGTGGGAATACATGGAATGGGTGGCATTGGTAAGACTACAATAGCTAAGGTTTGTTATCAGCGAATTCGTGATGAATTTGAAGCTCATTGCTTCCTCTCTGACGTTCGAGAGAATTATTTCAGAACCTCTGGCGACCTTCCATATTTACAAACCAAACTCCTTTCAAGGATGTTTTCATTTAAAAACAACCACATATTGGATGTTGAAGAAGGTATCGCTATGATCAATAAAGCCATTTTTCGAAAAAAGACACTTCTCGTCCTTGATGATGTGGATTGTTCGGATCAAATCATGGGATTGATTCCAAACAAAAGCTCTTTTGGCAATGGAAGTAGAATCATCATCACAACACGAAATGCGGATTTACTTTCGAACGAATTCGGGGTGAAAAGAATTTTTGAAATGGATGAACTTAAATATGAGGAAGCTCTTCAACTCCTTAGTTTGAGTGCTTTTATGAAAACGTGTCCAAAAGAAGGTTACTTAGAACACTCTAAGAAGATTGTAAAGGTTGTGGGAGGCCACCCTCTTGCACTCAAATTGTTAGGGTCGTCTCTAAGAAACAAAAATTTGAGTGTGTGGAATGAGGTGATAGAAGAGGTTGAAGGAGGTGGGAATATTCATGAAAAAATTTTCAAGTGTCTTAAAGTGAGTTATGATGGGTTGGATGAATGGGAGAAAGAGATATTTCTTGATGTTGCTTGCTTCTTCAATGGGAAGAGAAGAGAAGTTGTAGAAGAGATATTAAATGGATGTGGTTTCTATGCCAAAACAAGGATCGAACTTCTTATTCAAAAATCTCTCTTAACTCTTTCTTACGACAACAAATTGCATATGCATGATCTATTGCAAGAAATGGGTCGAAAGATTGTTCGCGATAAGCATGTTCGAGATCGATTAATGTGCCACAAGGATATAAAAAGCGTG

GTAAGATATATATTAATGTTGACCTATTTGGATGGAATGACTTTCTACATTCTTAAAAAAATGCAAATTAAAAGAGAATATATAGTATCTTTAAATGTTTAGATGGTAACCATTTGATTTTATGTTATTTTATTTTTTGAAAAACCAAATGGTTTCAG

GTGACAGAGACATTGGTCCAAAGCATATTTTTCAAATCAAGTTCAAAGAACATGGTTGAATTTCCAATTTTATTTTCAAGAATGCACCAACTTAGGCTGCTTAATTTTCACAATGTGAGACTGAAAAACAAGTTGGAATATTGCATTCCAAGTGAGTTAAGGTATTTGAAGTGGAAAGAATATCCATTGGAGTTTCTGCCAATCAATAGCTCTGAAGAATGTAAGCTTATTGAGCTTCACATGTGCCATAGCAATCTCAAACAATTTTGGCAACAAGAAAAG

GTAACAATCTCATACCCATATATCTAAAGTTTTATTTGGAAGAAGTTATTATGATATCTTTTAGTTTCAAATAATCTTTACTTTTAACAG

AATTTGGTGGGGCTGAAGTATATCAAACTCAATAGTTCTCAGAAGTTGTCCAAAACACCAAACTTTGCAAACATTCCAAATCTCAAAAGATTAGAGCTTGAAGATTGCACAAGTTTAGTCAACATTCATCCATCAATTTTCACTGCAGAAAAACTCATTTTCTTGAATTTGAAAGATTGCATCAATCTCACCAATCTTCCATCCCGCATTAACATCAAGGTTCTTGAAGTCTTGATTCTCTCTGGTTGTTCAAAAGTAAAAAAAGTCCCTGAATTTTCAGGTAACACAAATAGATTACTCCAACTCCATTTGGATGGTACCTCCATATCAAATCTACCATCATCAATTGCAAGCTTGAGTCATCTAACAATATTGAGTTTAGCCAACTGCAAAAAGTTAATCAACATTTCGAACGCAATGGAGATGACATCTCTCCAAAGCTTAGATGTTTCTGGATGTTTGAAGCTGGGAAGTAGAAAAAGAAAGGCGGACGATGGCGAATTGGGGGAGCTCGATGTGAGAGAAACCACACGAAGAAGAAGAAACGATGACTCTAACAATATTTTCAAAAAGATCTTCCTTTGGTTATGCAAAACTCCAGCTAGTGGCATTTTTGGGATCCCATCATTAGCTGGTTTGTACTCTCTTACAAAACTAAACTTGAGGGATTGCAACCTTGAAGAAATCCCACAAGGGATTGAGTGTTTGGTGTCATTGGTAGAGCTCGACTTGAGTGGCAATAGTTTCTCTCATCTTCCAACAAGCATATCAAGACTTCATAACTTGAAAAAACTGAGGATAAACCAATGCATAAAGCTTGTACAGTTCCCAAAGTTACCTCCAAGGATCTTGTTTTTGATGTCAAAGGATTGCATTTCATTGAAAGATTTTGTAGATATTTCAAAAGTTGATAATTTATATATAATGAAAGAAGTGAACCTTTTGAACTGCTACCAGTTGGCTAACAACAAAGGCTTCCATAGATTGATCATTTCTTGGATGCAGAAGATGCTTTTTCGAAAAGGAACATTCAACATCATGATTCCGGGGAGCGAGATTCCTGATTGGTTTACAACAAGGAAAATGGGATCTTCGGTATGCATCGAGTGGGATCCAGATGGCCCAAACACCAACATGATTCGATTTGCGCTCTGCGTCGTTTTTGGTCTGAGTGAGAAAATCGACGTTGTCAATGTTCCTTCCTTTGCCATTATCGCATCAGTGACTGGAAAAGACCGTAACGACTCGAATTTGAAGAATGGAGGTGATCTTCTGATTGGTGGATTTCCTGTTGCAGGGATGAAGAAGTTAGACCATATATGGATGTTTGTTTTGCCACGAACTGGGACTCTGGTAAGAAAGATTAGCAACTATAAAGAGATTAAGTTTAGATTCTTACTTCAAGCAGCTAATTATAGACAATCAATTACCCCAAATGTCAAAGTGAAGGAGTGTGGAGTTGGTTTGATAAATTTGGAAGAAGAGAAGGAGGCCATGAAACGGTATGCTTCTCACATTATCTTGAGAAACAAGAACTTATTGTTGGATTATTGATGTAGCTTATTAGTATAAAATTATCAGTTGATTAATGATGATTCAACCTGGTATCAAAGTATAAGGTTCTGTATTGAAACTTGCTTAATTTCAATAATCATGAGCGACGTAATTAGAAATTGCATTCCCCTTTCAACAATTGCATTCTTAGGTAGAA

>MELO3C022143

ATGGCTTCCTCCACCACCACCAAGGAATCATCTCCTTTTTCTTCTTCTCCTAGATACATATTTGACGTCTTTCTCAGCTTCAGAGGCGTGGACACTCGCAATAACATCACAAATCTTCTTTACGAAGCTCTGAGGCGACAAGGCATCATTGTTTTCAGAGATGACGATGAGCTCGAGAGAGGGAAGGCTATTGCTAACACTCTAACCAACTCGATTAGGCAATCCAGGTGTACCATTGTTATTCTCTCTAAAAGATATGCAGATTCAAAATGGTGCTTGAGGGAGTTGGTTGAGATTGTCAAATGCAAGAATTCCTTCAATCAAATAGTTCTTGTGGTTTTCTACAAAATTAAGCCCTCCGATGTCAACAGCCCTACTGGGATTTTTGAGAAATTCTTTGTTGATTTCGAGAATGATGTTAAGGAGAATTTTGAAGAGGTTCAGGACTGGAGGAACGCCATGGAAGTGGTTGGAGGTCTCACTCCATGGGTTGTAAACGAACA

GTATGATTCTTTGCTCCCTTTCTCTCATCGTCGTCATTATTCCGTTTAGCTATTGTATTTGGAATTTGAAATTCATGTTTGAGTGATGTTGGATGTTAAAGAAATGGACAATCAAAGTTGGATAGAAAACGTAAAGCAAGATAGAATCGACACATATTTATGAAGGAGAGCAATTTATTTCTGGAGAGAATAATTCATTCTATATAAACTCTCTTACGTTAATGGTGCATCCTTTATTATTCTCTCTAATAATAAACTACTCTCCTTGGGTGGACGTAGTTAACACAGTGATGGTGAACCATGAACCTGTGTGTCGATTATCTTCATCTTTTCATTTTTTGTCCATCATTGATTTCGATTCCCAGTCTGTTTAGTCACTCGCAAGGGTTCGAGCCGAATCTCAGAAAGAAGATCAGATCTTTTGCGGAAAGGGGGTGCGTCAGTGGCTTAGCTTGCTTCATGACCCCTATTGAAATGAAAGAACTTCTCTCCGATTCTAGTACCAGAATTGTGTCTGTTGATCGCGGAAAGTCTAAACTTTTATCTTAACCTTCTAGACATGAAGGCATAGGATTAGTTAGAAGAAACTTGCTTACGTGAATGATAGTTGTTTGTATTATTTGCAG

GACCGAAACAGAGGAAGTCCAAAAGATTGTTAAGCATGCTTTCGATCTTCTGCGTCCTGATTTGCTTAGCCATGATGAGAATTTGGTTGGCATGAACTTGAGATTAAAAAAAATGAATATGCTTATGGGCATAGGGCTGGATGATAAGCGCTTTATTGGGATATGGGGGATGGGTGGAATAGGCAAGACAACTATTGCTAAAGCTGTTTTCAAAAGTGTCGCTCGTGAATTCCATGGAAGTTGCATTCTGGAAAATGTTAAGAAAACTTTAAAGAATGTTAGAGGCTTGGTGTCCTTGCAGGAGAAACTTCTTTCCGATACTCTAATGAGAGGAAAAGTTCAAATTAAAGATGGTGAGGGAGTTGAAATGATAAAGAAAAACTTAGGAAATCGAAAAGTTTTTGTTGTTCTCGATGATGTTGATCATTTTAGCCAGGTGAAAGATCTGGCAGGAGGAGAAGAGTGGTTTGGTTGTGGAAGTAGAATCATCATTACAACAAGAGATGAAGGTTTGCTTCTTTCTCTTGGAATTGATATAAGATACAATGTTGAGAGTTTCGGTGATGAAGAGGCTCTTCAGCTCTTTTGCCATGAAGCATTTGGAGTGAAGTTCCCTAAGAAAGGTTATTTGGATCTTTGTATGCCATTTGTAGAATATGCTGAGGGCCTTCCATTGGCAATCAAGGCCCTTGGGCATTCTTTGCACAATAGATTGTTTAAGTCATGGGAAGGTGCCATTAGAAAGTTAAATAATTCTTTAAACAGGCAAGTATATGAAAACTTGAAAATTAGTTATGATGCACTTGGAAAGGAAGAGAGGAGAATTTTTTTGTATATTGCCTGTTTTCTTAAAGGACAGAGCAAAGACCAAGTCATTGACACATTCGTGAGTTTTGAAATTGATGCTGCTGATGGACTTCTTACCAGAAAAAAGGCTGCCGATGTACTTTGTATAAAAGAAACTGCTGCTGATGCACTAAAAAAATTGCAGGAGAAATCCCTCATAACTGTGGTGAATGACAAAATACAGATGCATAATTTACACCAAAAACTAGGCCAAGAAATCTTTCGTGAGGAGTCATCGAGGAAAAGTAGTAGGCTATGGCATCGAGAGGATATGAACCATGCTTTAAGGCATAAACAG

GTAAAACTTCAATAAGGAAATGTATGCAATGTTACTCATAGTTCAAATACAACCATTTGACTTTGATTTCTTCTCGTTTCATATCCTTAG

GGAGTTGAAGCTATTGAAACCATTGCCTTGGACTCAAACGAGCATGGAGAGTCACACTTAAATACCAAGTTCTTTTCAGCAATGACCGGTCTAAAAGTGTTGCGTGTTCATAATGTATTCCTTTCTGGAGATCTTGAATATCTCTCAAGCAAGTTGAGACTTCTCAGTTGGCATGGATATCCCTTCAGAAATTTGCCATCGGATTTCCAGCCAAATGAACTATTGGAACTCAATTTACAGAATAGCTGCATTGAAAATTTTTGGAGAGAAACAGAG

GTAGGTAGTGTAGCTTTTATCAATAATATTCTTTATTTTCTTTGGAGGTTGAATTCATGATATCATAACATTCTTGTAACCATATCTTTTCTTTAACAG

AAGTTGGATAAATTGAAGGTAATAAACCTTAGTAATTCCAAGTTTTTATTGAAGACCCCTGACCTGTCAACGGTGCCAAATCTTGAGAGGTTGGTCTTGAATGGTTGTATAAGACTACAAGAGCTTCACCTATCTGTCGGCATTCTAAAGCATCTAATCTTTTTGGATCTTAAGGACTGCAAATCTCTCAAAAGCATTTGTTCTAATATTTCTCTTGAATCACTCAAGATTCTCATTCTTTCTGGTTGTTCAAGACTTGAAAATTTTCCAGAGATTGTGGGAAACATGAAACTTTTGACAGAGCTTCATTTAGATGGCACTGCTATTCGAAAATTGCATGCCTCAATTGGAAAACTTACAAGCCTTGTTTTGTTGGATCTTAGAAACTGCAAAAATCTTCTTACACTTCCAAACGCGATCGGTTGCTTAACATCCATTAAACATCTCGCATTGGGTGGCTGCTCAAAGCTTGACCAAATTCCTGACAGCTTGGGGAACATTTCTTGTTTAAAGAAACTTGATGTGAGTGGTACTTCTATTAGTCATATCCCATTGTCTCTACGACTTTTGACGAACCTCAAAGCATTGAATTGCAAAGGCCTATCCCGAAAATTATGTCATTCATTATTCCCATTATGGAGTACGCCGAGGAATAACAATTCACATTCATTTGGTTTGAGGTTGATAACTTGCTTTTCGAATTTTCATTCAGTGAAGGTTTTGAATTTTAGTGACTGCAAGCTGGCAGATGGAGACATACCCGACGACCTCAGCTGTTTGTCTTCATTACACTTTCTGGATCTAAGCAGGAACCTCTTCACCAACCTGCCTAATAGTTTGGGTCAACTTATCAATCTCAGATGCCTTGTTTTGGACAACTGCAGTAGGCTCAGGTCATTACCGAAGTTCCCAGTCAGCTTACTTTATGTACTCGCAAGGGATTGTGTGTCACTGAAAGAAGACTATAACAAAGAAGATCGCGGGCCTATGAGCGAAACAGAAGTAAGGGTCCTTAGTTACCCCTCATCAGCTGAAGACCAAAACTCTAAAATCTCTCAGATGATATCAAGTATGTGCACAGCTTGGGAGAATGGGGGTTGAG

>MELO3C022148

ATGTTTCTAGATATAACTTTTAAACAATTTTGTAGTTCTGAAGAAGGGGCCATCAAAGAAATTGTGAATCATGTTTTCAACAAATTGCGTCCTGATTTGTTTCGGTATGATGATAAATTAGTTGGAATTAGCCAAAGACTGCACCAAATAAATATGCTTTTAGGAATAGGTTTGGATGATATACGCTTTGTTGGAATATGGGGAATGGGTGGAATTGGCAAAACGACCCTTGCTAGAATCATTTACCGAAGTGTTTCCCATTTATTTGATGGCTGTTATTTCTTAGACAACGTCAAAGAAGCTTTGAAGAAACAAGGCATAGCTTCATTACAAGAAAAGCTTCTAACAGGAGCTTTAATGAAGAGAAACATTGACATCCCTAATGCTGATGGAGCTACATTAATCAAGAGAAGAATAAGTAATATCAAAGCTCTTATAATTCTCGACGATGTTGACCATCTAAGCCAACTTCAACAGTTAGCTGGCAGTTCAGATTGGTTTGGTTCAGGAAGTCGAATCATCGTTACAACGAGAAACGAACATCTACTCGTGTCTCATGGAATTGAAAAAAGGTACAAAGTTGAGGGGCTGAATGTTGAAGAAGCTCTTCAACTTTTTTCACAAAAAGCATTTGGAACAAACTATCCAAAGAAAGACTATTTCGATCTCTCTATACAAGTTGTGGAATATAGTGGAGATCTTCCATTAGCAATTGAAGTTCTTGGATCTTCTTTACGTGATAAATCAAGAGAAGTATGGAAAAATGCAGTGGAGAAGTTAAAAGAAATTCGTGACAAGAAAATATTGGAAATATTAAGGGTTAGTTATGATTTGCTAGACAAATCAGAGAAGGAAATATTTCTAGATCTTGCATGTTTTTTCAAAAAGAAAAGTAAAAAGCAAGCAATTGAAGTGCTTCAAAGCTTTGGATTTCAAGCTATTATTGGACTTGAAATTTTGGAGGAAAGATCTCTTATTACTACACCACATGAGAAGATACAAATGCATGATTTGATACAAGAAATGGGTCAAGAAGTTGTTCGTAGAATGTTTCCGAACAATCCTGAAAAACGTACTAGGCTGTGGCTTCGTGAGGATGTTAATCTTGCTCTAAGTCATGATCAG

GTAAGATCTTCAATCGAGTTCAGGTTAATTCAATAAATTTTATTCTACTTTGGTCTCCAAACTTCATACCTATATTTCATCTATTTACTATTGGATTGAATTTAAAAGGATATAAAACTACTTTTGTTGTACTTGAAATCAATTTCTTCAAGATAGTTAGCCAAACCCGATTGTGTTAGTTTATCAATCTAATGTTCAATTTGTTCCTTGAGCTTTAAGACCATTTTTTTTAGTCCTTGCTACGATTTTGTAATATACTATCCTAGCAAATCTACTATCCGCTCATTAATTGAAGCATGCTATAAATGCATGTACTTGAAGTTGCATTATCTAAGGTCCCATTTGTTAACGATTTCAGTTCTTACAATGGTTTGCATCTTTCTCAAGTACAAATGTTGAATATTATTCTTAGTCAAATTCCAAAATAAAAACAAGTTCTTAGAAAAATACTTTTTTTAGTTTTCAAAAATTGATTTGGTTTTTTAAAGCAATGATAAAAGTATAAAGTAGATAACAAATTAGCAAAGACGTTAGAGGGTGAAATTGTTGTTCATGGACTTATATTTTCAAAAAACTAAAAACAAAAAACATAGTTAGCAAACGGAACTCTATTGCATTTGAAAATTTATATATTTAAAGTGACATTTAAAAGTGTAGTGACCAAAATTATACAATATGATACGTTTGAACCATATTTTATCTACATGAGTTTTTTTACAG

GGAGCAGAAGCAATTGAAGGAATAGTGATGGATTCAAGTGAGGAAGGAGAATCACATTTGAATGCCAAAGTCTTTTCAACAATGACCAATCTTAGGATATTGAAAATAAACAATGTTTCCCTTTGTGGAGAACTTGACTATCTCTCTGATCAGCTGAGGTTCCTCAGTTGGCATGGCTACCCTTCAAAGTATTTACCTCCAAATTTCCATCCCAAAAGCATATTAGAACTTGAATTGCCCAACAGCTTCATTCACTATCTTTGGAAAGGCTCAAAG

GTAAATTAAAAGTTCAGCACAAAAAACTTCCATAAATGAATGAGAGATTGAACCTCGCTGATTTTAAACAAAAGAGTGTGTATATCAATTTATCATCGAGCAATATATTCACCTTGGCAACTGTTTAATTGAGTGTATTCTTTGCTCTTCCTTTGCTGTTTTTTATATATATCATCATGTGCATATATAGAGCTTATCTCTAGCTCTTTGACGAACTATATGCTTTGTACTTGATTATGTATATCCTATATTTTGTTTTTGGATGTGGGACATAAAGAGGGTTGCTAGATATTTGTCAATCTAGTTGTGTGATATACCAGTGCATCGACTGATCCTTCTTTATATTATATGTAGTTGTGTGATACACCAGAACTTCTTTTTTGTTACTTCATTTTGTTTAAAGCTTGTATTAATCACTTTCTAATTTTTCTGTGATGTGTTTTTTGCAG

AGATTGGACAGATTGAAGACAGTAAATCTTAGTGACTCTCAGTTCATATCCAAGACACCTGATTTTTCAGGGGTTCCAAATCTAGAAAGATTGATCTTGAGTGGTTGTGTAAGACTGACAAAACTTCATCAATCTCTAGGTTCTTTAAAGCGCCTAATTCAATTAGATCTTAAGAATTGCAAAGCCCTAAAAGCTATTCCTTTCAGTATTAGCTTAGAATCACTCATAGTTTTAAGTCTTTCAAACTGTTCAAGCCTAAAAAATTTCCCAAATATTGTTGGAAACATGAAAAACTTAACAGAGCTTCATTTAGATGGAACATCCATACAAGAATTACATCCATCAATAGGACACTTAACAAGACTTGTCCTATTAAATCTTGAAAATTGCACAAATCTTCTAGAACTTCCAAACACAATTGGTTCTTTAATATGCTTAAAAACTCTCACGTTACATGGCTGCTCAAAACTTACTAGAATTCCAGAGAGTTTAGGATTTATTGCTAGCTTGGAGAAGCTTGATGTTACTAATACTTGTATAAATCAAGCTCCATTGTCCCTTCAACTTTTGACGAATCTCGAAATACTAGATTGTCGAGGTCTTTCTCGTAAATTCATCCATTCATTATTTCCATCATGGAATTCTTCATCATATTCTTCTTAATTAGGATTGAAGTTGACATATTGTTTATCTAGTTTTTGTTCAATGAAGAAATTGAATTTGAGTGATTGTAGTTTAAAGGATGGGGATATACCAGATAACCTTCAAAGCTTGCCTTCACTTGAAATTCTTGATTTAAGTGGAAACAGTTTTAGTTTTCTACCTAAAAGTGTTGAACATCTTGTGAATCTTAGAACTCTTTATTTGGTAAATTGTAAAAGGCTTCAAGAATTGCCAAAACTCCCACTTAGTGTTCGTAGTGTAGAAGCAAGAGATTGTGTTTCACTTAAGGAATATTACAATCAAGAAAAGCAAATGCCTTCAAGTTCAACAGGTATGGCTGTTATAAGTTGTCCTATAACGGATGAAGAACATAATTTCAAGATCGATAGAGTCAATTTGTCAAGTATTCATCTTCGTACAATGGTTCAAAGATATATTGAGGTACACACACTCTCTCTCTCTCTTTACATCTTTTTAAATTTTGAAAACATTTATTTCCATTTCATTGCGAGAAAATTACCTTTTAGGTTTAGATTATTTCATTTGTGTGCTATCTAG

>MELO3C022149

ATGTGTATCCAACCATTCCTCTATATCCTGATTATTCATGTATGTGCAACTTATTTTGAAATAATGTTTCTAGATATAACTTTTAAACAATTTTGTAGTTCTGAAGAAGGGGCCATCAAAGAAATTGTGAATCATGTTTTCAACAAATTGCGTCCTGATTTGTTTCGGTATGATGATAAATTAGTTGGAATTAGCCAAAGACTGCACCAAATAAATATGCTTTTAGGAATAGGTTTGGATGATATACGCTTTGTTGGAATATGGGGAATGGGTGGAATTGGCAAAACGACCCTTGCTAGAATCATTTACCGAAGTGTTTCCCATTTATTTGATGGCTGTTATTTCTTAGACAACGTCAAAGAAGCTTTGAAGAAACAAGGCATAGCTTCATTACAAGAAAAGCTTCTAACAGGAGCTTTAATGAAGAGAAACATTGACATCCCTAATGCTGATGGAGCTACATTAATCAAGAGAAGAATAAGTAATATCAAAGCTCTTATAATTCTCGACGATGTTGACCATCTAAGCCAACTTCAACAGTTAGCTGGCAGTTCAGATTGGTTTGGTTCAGGAAGTCGAATCATCGTTACAACGAGAAACGAACATCTACTCGTGTCTCATGGAATTGAAAAAAGGTACAAAGTTGAGGGGCTGAATGTTGAAGAAGCTCTTCAACTTTTTTCACAAAAAGCATTTGGAACAAACTATCCAAAGAAAGACTATTTCGATCTCTCTATACAAGTTGTGGAATATAGTGGAGATCTTCCATTAGCAATTGAAGTTCTTGGATCTTCTTTACGTGATAAATCAAGAGAAGTATGGAAAAATGCAGTGGAGAAGTTAAAAGAAATTCGTGACAAGAAAATATTGGAAATATTAAGGGTTAGTTATGATTTGCTAGACAAATCAGAGAAGGAAATATTTCTAGATCTTGCATGTTTTTTCAAAAAGAAAAGTAAAAAGCAAGCAATTGAAGTGCTTCAAAGCTTTGGATTTCAAGCTATTATTGGACTTGAAATTTTGGAGGAAAGATCTCTTATTACTACACCACATGAGAAGATACAAATGCATGATTTGATACAAGAAATGGGTCAAGAAGTTGTTCGTAGAATGTTTCCGAACAATCCTGAAAAACGTACTAGGCTGTGGCTTCGTGAGGATGTTAATCTTGCTCTAAGTCATGATCAGGTAAGATCTTCAATCGAGTTCAGGTTAATTCAATAAATTTTATTCTACTTTGGTCTCCAAACTTCATACCTATATTTCATCTATTTACTATTGGATTGAATTTAAAAGGATATAAAACTACTTTTGTTGTACTTGAAATCAATTTCTTCAAGATAGTTAGCCAAACCCGATTGTGTTAGTTTATCAATCTAATGTTCAATTTGTTCCTTGAGCTTTAAGACCATTTTTTTTAGTCCTTGCTACGATTTTGTAATATACTATCCTAGCAAATCTACTATCCGCTCATTAATTGAAGCATGCTATAAATGCATGTACTTGAAGTTGCATTATCTAAGGTCCCATTTGTTAACGATTTCAGTTCTTACAATGGTTTGCATCTTTCTCAAGTACAAATGTTGAATATTATTCTTAGTCAAATTCCAAAATAAAAACAAGTTCTTAGAAAAATACTTTTTTTAGTTTTCAAAAATTGATTTGGTTTTTTAAAGCAATGATAAAAGTATAAAGTAGATAACAAATTAGCAAAGACGTTAGAGGGTGAAATTGTTGTTCATGGACTTATATTTTCAAAAAACTAAAAACAAAAAACATAGTTAGCAAACGGAACTCTATTGCATTTGAAAATTTATATATTTAAAGTGACATTTAAAAGTGTAGTGACCAAAATTATACAATATGATACGTTTGAACCATATTTTATCTACATGAGTTTTTTTACAGGGAGCAGAAGCAATTGAAGGAATAGTGATGGATTCAAGTGAGGAAGGAGAATCACATTTGAATGCCAAAGTCTTTTCAACAATGACCAATCTTAGGATATTGAAAATAAACAATGTTTCCCTTTGTGGAGAACTTGACTATCTCTCTGATCAGCTGAGGTTCCTCAGTTGGCATGGCTACCCTTCAAAGTATTTACCTCCAAATTTCCATCCCAAAAGCATATTAGAACTTGAATTGCCCAACAGCTTCATTCACTATCTTTGGAAAGGCTCAAAGGTAAATTAAAAGTTCAGCACAAAAAACTTCCATAAATGAATGAGAGATTGAACCTCGCTGATTTTAAACAAAAGAGTGTGTATATCAATTTATCATCGAGCAATATATTCACCTTGGCAACTGTTTAATTGAGTGTATTCTTTGCTCTTCCTTTGCTGTTTTTTATATATATCATCATGTGCATATATAGAGCTTATCTCTAGCTCTTTGACGAACTATATGCTTTGTACTTGATTATGTATATCCTATATTTTGTTTTTGGATGTGGGACATAAAGAGGGTTGCTAGATATTTGTCAATCTAGTTGTGTGATATACCAGTGCATCGACTGATCCTTCTTTATATTATATGTAGTTGTGTGATACACCAGAACTTCTTTTTTGTTACTTCATTTTGTTTAAAGCTTGTATTAATCACTTTCTAATTTTTCTGTGATGTGTTTTTTGCAGAGATTGGACAGATTGAAGACAGTAAATCTTAGTGACTCTCAGTTCATATCCAAGACACCTGATTTTTCAGGGGTTCCAAATCTAGAAAGATTGATCTTGAGTGGTTGTGTAAGACTGACAAAACTTCATCAATCTCTAGGTTCTTTAAAGCGCCTAATTCAATTAGATCTTAAGAATTGCAAAGCCCTAAAAGCTATTCCTTTCAGTATTAGCTTAGAATCACTCATAGTTTTAAGTCTTTCAAACTGTTCAAGCCTAAAAAATTTCCCAAATATTGTTGGAAACATGAAAAACTTAACAGAGCTTCATTTAGATGGAACATCCATACAAGAATTACATCCATCAATAGGACACTTAACAAGACTTGTCCTATTAAATCTTGAAAATTGCACAAATCTTCTAGAACTTCCAAACACAATTGGTTCTTTAATATGCTTAAAAACTCTCACGTTACATGGCTGCTCAAAACTTACTAGAATTCCAGAGAGTTTAGGATTTATTGCTAGCTTGGAGAAGCTTGATGTTACTAATACTTGTATAAATCAAGCTCCATTGTCCCTTCAACTTTTGACGAATCTCGAAATACTAGATTGTCGAGGTCTTTCTCGTAAATTCATCCATTCATTATTTCCATCATGGAATTCTTCATCATATTCTTCTTAATTAGGATTGAAGTTGACATATTGTTTATCTAGTTTTTGTTCAATGAAGAAATTGAATTTGAGTGATTGTAGTTTAAAGGATGGGGATATACCAGATAACCTTCAAAGCTTGCCTTCACTTGAAATTCTTGATTTAAGTGGAAACAGTTTTAGTTTTCTACCTAAAAGTGTTGAACATCTTGTGAATCTTAGAACTCTTTATTTGGTAAATTGTAAAAGGCTTCAAGAATTGCCAAAACTCCCACTTAGTGTTCGTAGTGTAGAAGCAAGAGATTGTGTTTCACTTAAGGAATATTACAATCAAGAAAAGCAAATGCCTTCAAGTTCAACAGGTATGGCTGTTATAAGTTGTCCTATAACGGATGAAGAACATAATTTCAAGATCGATAGAGTCAATTTGTCAAGTATTCATCTTCGTACAATGGTTCAAAGATATATTGAGGTACACACACTCTCTCTCTCTCTTTACATCTTTTTAAATTTTGAAAACATTTATTTCCATTTCATTGCGAGAAAATTACCTTTTAGGTTTAGATTATTTCATTTGTGTGCTATCTAG

>MELO3C022152

ATGGCTTCTCCAGCAACAATAATGGAGAGAAGAGCTTCAATTACATCCCTATCTTCTCCTCCTCCTCCTAATTATTATTCTATCTCTCTTCCTCTTCCTCCCTTACGAAACTACGACGTTTTCCTCAGCCACAGAGCTAAGGATACCGGGCAAAGTTTCGCAGCCGATCTTCATGAAGCGTTGACAAGCCAAGGAATTGTAGTTTTCAGAGACGACGTAGACGAAGAAGACGGAGAGAAACCGTATGGTGTAGAGGAGAAGATGAAGGCCGTGGAAGAATCGAGATCTTCGATCGTGGTTTTTTCGGAGAACTATGGGAGTTTTGTTTGCATGAAGGAAGTAGGGAAGATTGCAATGTGTAAGGAGTTGATGGATCAATTGGTTCTTCCGATATTTTACAAAATAGATCCAGGCAATGTGAGGAAGCAGGAGGGGAACTTTGAGAAGTACTTCAATGAACATGAAGCCAATCCTAAGATTGATATTGAAGAAGTTGAGAACTGGAGATATTCTATGAATCAAGTTGGCCATCTCTCTGGATGGCATGTCCAAGATTCCCA

GTTAAGTAATAATTATATATTCACTCTCTTCAACTTTTTCTTTTTAATTTAAGTATGTTTTGTGGTTCATATTTACAAGGGAAGCACAGATAAAAGTCATCTGCCTAGGATTTGATATTCTAAAATTATCTTAACATTCATATGTTGTAGGATAGAGTTGTTTGGTAAGACAACCTGATGTATGGGTTCTTTCTTGAACACTAAGAGATGAAATATTTTTAGAGAAAAAAAGTTGAAAAGGAATTCTGAGAAAAATGCTAATAGAAATTTTCTTTTGTTTAAAAATTTTTTTTTTAAAACTTTGAGGGAAAAGGAGAAAAGTTAGAATAAATGTTTGAAGCCTCAAAAGATTTAGTGATATTTTAAGGGTGTAAAAATTTTTTTTTTCTTTTTAAGAAAGTTGTGTTTTAAGTTGGAGAAAGAAGAGCAGAAGAGCTCTTCCAAAAATAATAAAAAATACTAAATGAAAAAAAATTATTACACTTAATTTAAATATCTGATTGAGTTTAGAAGGGGACATGAATAAATCGATATTACATCTTAGTAAGAAAGGATCATGAGAACATGAAAGTAATAAAATTGGAGCAATTCTATATTAGGTGACGTCCTTCTGTGAATTTTCATAGGATGCCCTAACCTAGCTTGTAATGGATTTAATTTTTTTTTAAAGGAAATTATATATTTGTTTTTGGCAGAATCTCTTATAATCAAATTTTGTCTTTTCTGCCTAAACATTTATATTTTTAAACGAATTCCAAAAACAATAACATTTTCAAAATTTAGCATTCCGATAGAGATGATTACTAAAAATGAAAAGTATTATATGCATATAG

GTCTGAAGAAGGGAGCATAATCGATGAAGTTGTGAAGCATATATTCAACAAATTGCGTCCTGATTTGTTTCGATATGATGATAAATTAGTTGGAATTACCCCAAGATTACACCAAATAAATATGCTTTTGGGAATAGGTTTAGATGATGTACGATTTGTTGGAATATGGGGAATGGGTGGAATTGGCAAAACTACACTTGCTAGAATCATTTACAAAAGTGTTTCTCATTTATTTGATGGATGTTATTTCTTGGACAATGTCAAAGAAGCTTTGAAGAAAGAAGACATAGCTTCATTACAGCAAAAGCTTATAACAGGAACTCTAATGAAAAGAAACATTGACATCCCTAATGCTGATGGAGCTACATTAATCAAGAGAAGAATAAGTAAGATTAAAGCTCTTATAATTCTTGACGATGTCAACCATCTAAGCCAACTTCAAAAATTAGCTGGCGGTTTGGATTGGTTCGGTTCGGGAAGTCGAGTCATTGTTACAACGAGAGACGAACATCTCCTGATTTCGCATGGAATCGAAAGACGATACAATGTTGAAGTGCTGAAAATTGAAGAAGGTCTTCAACTTTTTTCACAAAAGGCATTTGGAGAAGAGCATCCAAAGGAAGAGTATTTTGATCTTTGTAGCCAAGTTGTAAACTATGCTGGAGGACTTCCATTAGCAATTGAGGTTCTTGGATCTTCTTTACATAATAAACCAATGGAGGATTGGATAAATGCAGTGGAAAAGTTGTGGGAAGTTCGTGATAAGGAAATTATAGAAAAGTTAAAAATCAGTTATTATATGTTAGAGGAATCTGAACAGAAAATTTTTCTAGACATTGCATGTTTTTTCAAGAGGAAGAGTAAGAACCAAGCAATAGAAATTCTTGAAAGTTTTGGATTTCCTGCTGTTCTTGGACTAGAAATATTGGAGGAGAAATGTCTTATTACTGCACCACATGATAAGCTACAAATACATGATTTAATACAAGAAATGGGTCAAGAAATTGTTCGCCATACCTTTCCGAATGAGCCAGAAAAACGAACTAGGTTGTGGCTTCGTGAGGATATCAATCTCGCTCTAAGTCGGGATCAGGTAACTATATATATTTTTTTAATTCATAGGAAAACTTGGCATGTGGGAATGATATATATCTATCTGTATATATGTATGATGAATTACTAAT

>MELO3C025516

ATGACAGAGAAGGACAAGTGTTTGACATATGGGATTCGGAGAAGGCATTCAGAAAACGAAAAAGTAGACGGGAACAAAGAGATGGGAACAAAGAAAGGGCTTGGAGAGAAGGGAGAAGGATTACGAGAAAGAGGGAGAAGGATTAAGCAGAGTAGAAAGAAGGA

GTGAGGAACAGTAGACAGAGGAGTGTCGAACGGTAAAAGGGAGATCTGATGAGGATAGAAGAGTCAGTAAAGATTATGGAAACCTTAAGACTAGAATTTCCATAATCCGAACCCCAAACTTGGAGTGGGCTAAAATAATCAACTCCACTCCCCTCTAGTTTGGGGCCCAAACACACTCTTAACGTGTTTAGCAATATTTTAGATTATCTTATCGCAGTAATATGTATAGAATCTAAAATTTTGCTATATTTTGTAAATATTTTTAACAGTTTTGTCATTTACAATAATTATTTTTAAAGAAAATTTATTTTGTTAAAAAAAAAAACGTTAAAAGACCTAAGTGATAATAAGAATTTTTTAACAACCTTTTTCCATTAAATAATGTTTGTTTTTCTCCTAGTTTCTTTCAAATCATTTTTCATATTTTCTATCAACAATGACACTTTTATTTGAAAACAACCCAAACATATAAATTTTTAGTGGAAAACAAAAAGGAAAGAAAGAATGAGGTTTTTTAAAATTTCTGCCCCATGGCTTTGAGAACTCTACTTTCATGAAAATTATGTAG

GTCGGAAGCAGGGGCCATCGATGAAATTGTGAAGCATATTTTCAACAAGTTGCGTCCTGATTTGTTTCGATATGATGATAAATTGGTTGGAATTAGCCCAAGACTACACCAAATAAATATGCTTTTGGGAATAGGTTTAGATGACGTACGCTTTGTTGGAATATGGGGGATGGGTGGAATTGGCAAAACTACTCTTGCTAGAATCATTTACAGAAGTGTTTCCCATTTATTTGAAGGATGTTATTTCTTAGACAATGTCAAAGAAGCTTTAAAGAAAGAAGGCTTAGCTTCATTACAAGAAAAACTTCTAACAGGAGCTTTAATGAAAAGAAACATTGACATCCCTAATGCTGATGGAGCTACGTTAATCAAGAGAAGAATAAGTAATCTAAAAGCTCTTATAATTCTTGACGATGTCAACCATCTAAGCCAGCTTCAAAAGCTAGCCGGCGGTTCTGATTGGTTCGGTCCGGGAAGTCGAGTCATCGTTACAACAAGAGATGAACATCTTCTGATTTCACACGGGATCGAAAGACGATACAATGTCGAAGGGCTAAAAATTGAAGAAGCTCTTCAACTTTTTTCACAAAAGGCATTTGGAAAAGACCATCCAGAAAAAGGGTATTTTGATGTTTGTAGCCAAGTTGTAGATTATTGTGGAGGGCTTCCATTAGCAATTGAGGTTTTTGGATCCTCTCTACGAAATAAACCAATGGAGCAATGGGAAAATGCTGTGGAAAAACTGAAGGAAGTTTGTGATAAAAAAATTCTTGAGAAGTTGAAAATTGGTTATTATATGTTAGAGAAATCTGAACAGAAAATTTTTCTAGACATTGCATGTTTTTTCAAGAGGAAGAGTAAGAGACAAGCAATCGAAATTCTTGAAAGTTTTGGATTTCCTGCTGTTCTTGGACTGGAAATATTGGAGGAAAAATCTCTAATCACTGTGCCACATGACAAGATACAAATGCATGATTTAATACAAGAAATGGGTCAAGAAATCGTTCGTCAAAACTTTCCGAACGAGCCTGAAAAACGAAGCAGGTTGTGGCTTCGAGAAGATATAAATCTTGCTCTAAGTCGTGATGAG

GTACGATTCTAACCTAATCATACTTTTAAGCCATCAATCTATAATATGACATTGGTTCTTATACTTTTAAAATTATGTTACTACGTTCCTACGGTTAAAAAAAGTAAAAAAAAGAAAAAAAAAAAAAAATACTCCTTTTGTATTGATTTGCTCCATTTGTATCAAAAAACATTAACTATTACTGCTGAATTTTTTAGTTTAATTTCGATCTGGCCTGTGTTTCAAATATATTCATGTCAAATGTTATATATGATGAATAAATTTGTCAG

GGAACAGAAGCAATCGAAGGCATAATGATGGATTTGGATGAGGAGGGAGAATCGCATTTGAATGCCAAATCCTTTTCAGCAATGACAAATCTAAGAGTATTGAAAGTGAACAATGTTCATCTCTGTGAAGAAATTGAATATCTTTCTGATCAATTGAGGTTTATCAATTGGCATGGTTACCCTTTAACGACCTTACCATCAAATTTCAATCCCACAAACCTCTTGGAGCTTGAGTTGCCTAATAGCTCCATTCAGAATCTTTGGACTGCCTCAAAG

GTAAATTAAGGTTAATAATAAATTGTTTATAATGTTGCCTCTACCATTTTTACTTCATTCGTTAGAATTTGGGGAATAATATCTCTCTAGAACAAAAATAATTGATATTTTAACCATAACCACAATTTATTGGACGATATTTTATCGTGGGTAGCCCTTCGGAGCACAAAATCCAATCTTTTCTTGTAATTATTGTGATGCCTCTCCATTAGCTCTTAAAAGGGTCTTTAATTTCTCTACACCCTTGTTTTGGTTATCCTTTATGTGTTTGAGATTTTATCTAACCATCCACTGCATGTTGAACTTCCATGTCAAATGATTGCATCATTATTGGATATCTAAGATACGATACCTAATAAAGATATTATACTAGTTAAGATATCAACGTGTACACTATAATATCCCATTTACAAATGAATTTTTCTTTAAAAGAATTGTTATTTTGGCATTATTTGGTTTTTACTATTATATTTTTTTGCAG

AGCTTGGAAACATTGAAAGTGATAAACCTAAGTGATTCTCAATTCCTATCCAAGACCCCTGATTTGTCGGGAGTTCCATATCTTGAAAGGTTGGTTTTGAGTGGCTGTGTAGAATTACACCAACTTCACCACTCTTTGGGGAATCTAAAGCATCTAACTCAATTGGACCTCAAACATTGCAAGAAATTAACAAGTATTCCTTTCAATATTTGCTTAGAATCTCTCAACACTTTTGTTCTTTCAGGCTGTTCAAATCTCACCCATTTCCCAAAAATCTCAGCAAACATGAACCATTTATTAGAGCTTCATTTAGATGAAACATCCATAAAAACTTTGCATTCATCAATAGGACATTTAACAGGACTCGTTCTATTAAATCTCAGAAATTGCACAAATCTTCTAAAACTTCCTACCACTATTGGCTGTTTAACATCTTTGAAAAGCTTAAATTTACATGGCTGCTCAAAACTTGATAGTCTTCCAGAGAGTTTAGGAAATATTTCTTGTTTGGAAAAGCTTGATATTACTAGCACTTGTGTAAATCAAGCTCCAATGTCTCTTCAACTTTTGACTAAACTAGAAATACTAAATTGTCAAGGACTTTCTCGCAAATTTCTACATTCATTATTCCCTACTTGGAATTTCACTAGAAAATTCTCCAATTCACAAGGGTTGAAAGTGACAATTTGGTTTAATTTTGGTTGTTCTTTGAGGGTTTTAAATTTGAGTGATTGTAATTTGTGGGATGGAGATTTACCTAATGACCTTCATAGCTTAGCTTCATTGCAAGTTCTTGATCTAAGCCAAAACCATTTTACCAAATTACCAGAAAGCATTCGTCATCTTGTTAATTTGAGAGGTCTATTTTTGGTGGAATGTTTTCATCTATTGTGTTTGCCAAAGCTTCCATTAAGTGTTAGAGATGTGGATGCAAGGGATTGTGTTTCACTAAAAGAATATTACAATCAAGAGAAACAAATTCCTTCAAGTGAAATGGGAATGACTATTATTCGTTGTCCTATAACCAATGAACCAACTCAAAGCTACAAAATTCATCAGCCTGCCCTTTCTGCCATTCACCTAAGGACAACAACCCAACGATACCTTGAGGTAATTTCTCTACCTTTTACTTTTTATGCCAAATCCTTAAACATGTTTTTGCAACTTTTGCCTTTTCATAATTAAT

>MELO3C002877

ATGAATCCACCTTCAACCTCACCAACTTCATCTTCTCCTCCAAACACATGCCAAGTCTTCATAAGTTTCGCCACCGCTGACAGTAAATTTGTTTCCGAATTGGCCAAAGAGTTTAGGAATATCAGGCTAAGGACTTTTATGAGCAGTGGAGATGATGGTGAGATTATACATGAAGATGCAATGAAAAAGTCAAGGCATTTTGTTGTTGTTCTAACAAAAGATTATGTCAATTCCATCAAGTGTTTGAGAGAGTTGAGCAGAATAATGGAATACAAAGATGAAGGTTATGAAGAAGTCATCCCTGTGTTTTACCAAGATCCATCTTGTTTGGTAAAGGAATTACAAGATTTTGGAAAGAGATTAGAAGATTACGAAGAAATCATAAAAGAAGAAGGATTGATTGAGTTCACCCACTGTTGGTTTCTGAAAACAGATTTAGCAAAAAGGCTTAACAAATCATTTAGCGAAAAGGAAGAGAACAAGTCAAACCTTCTGATGGAGGTTCCAAGATGGAAATTTGCTTTGGAAAGAATCAACAATTTGCCAAAATCAATATTTGTAGGAGAAGAAAG

GTAAAGTCTCAAAAACCCATTAATTTATTAACTTTATGTCTTGTTTAATTTCTATTTTTTATTGTTCAAGCTAAGTTCAAAATTTTGTGAATATGTGTCGATAATTAACTTCTTGGACTGGGTTTTTATTGAATTAAGCTACAAAGGCTTGTTCATTTAGTTACTAGGGAATATTAGTAACCGTATGAAAAATCATTTCAGGGAAACAGTTTCTGGCATGCTTGTGGAATGATTTTTTAAGTATTTAAATAAGTGTTTAGGAAAACAAAAAATGTTTATATAGATATTTAGAAATTCAATCCAAATGGACTTTTGAACTTACTTGGATTTTTGTTTGCATGTTTCTTGCTGGTGGTGTGTATAGAATCCAATGTTATTAATCCTTTGATAGTCATGTAGATTTTTAAATCTATTTACTATTTGGTTATATATTTTTACATTACATCAAAATTAACTTAAAATTATTAAAAGGAGTTTTCAAAAATAATAAAAATAATAAAAATAATAAGAAAAATTGTTAAAAATGATAAATTTGACAAAATATTTATAAGATATAGAAAATTTTAGATTTTATCAATGATAAACATTGATATGCTTTAATCAGTCATATATTGATTGATAATAATAGAAATCTATCAATATTTATTATTGATAGAATCCAAAATTTTGTTATATATTGTAAATATTTTAGTTTATTTTATTATTTTAAATGTTATAGAAAACAAACTATTTACTTTTGAAAAAATTTACTAAAAGTTAAAAATTTATTAATTTTTTTTATGAAACGTAAATATTTTATCTAATTTACTATTTTTGAAATTTTATATTATTAAAATTAGTATAGAATTAATTTGGGAGTTATTTTGTAATGGCCAAAATGGCTTTTGTTTGGTAATTGATGTTTATGAATGTTTGATAGGGGAATGGTTAAAAATGAGACACAATTCACAAAATATTTACATTTTGCCGTAAAATGGTGGGCAGTAGGTTTTTATTTTAGAAAAATCTAAAATAATAATTTAGAAGTTGTTTTCAGCCGTTAAATGTGCTAATCCACCAAACCCCACTAAATTTGTTCCACTTAAATCGTTGTTTTCTAAAAAGTAAAAATTTTCTCATCACTTTTTATTACTTTTTTTAAATTCTTCATTTTTATAATTAAGAAATTATTATAAATGACAATATGGTTAGAAAATATTTATATAATATAACAAAGTTTTCGACTCTATGAGTAATAAACATTAATAATATACCGTCTATTGATGCCTACTAAATTTTACTATATATCAATGTCTATTATGTAAAATTTATAAATTTACTACATTTATAAATATTTTTATTTATTTTATTATATATTTGAAAATGTCAGTCCATACATTTAATCCTAAAACTACTTATAATCAAACACATTCATTCAAAATGATTTGTAGATACATGAACATAATTTTTAAGTTTGAAACAAATCATCAATATAGTTGCATGAAAACTAATTGTACAAAATCAATTATGTTTAAATCGTTTTTACCCGATAATTGGATTTAGGGTTAAAGAACAATTGTAAATTTTTAAAAAATGTTTATTTAATAATGAAAATTTTTCATAAATATAACAAATCAGTAAAATATTTATGGCGGGCATAACAAAGCGAGTTAGTTATTTTTTAAGTATTTTATATTTGTCCCTTTTATCGTCCTCCTTCTCTTTTTTCACTGTGATTTGATTTTTTTTCTTTCCATTGTCTTTTTTCTCTTCCTCTTCTTTCTCTTCCAAACTGTTATTTCGTTCAAAATCGTGTATCAAATATAAAATATCTATTTTTTAAAACTGTTATTTGGTTGTTCAAGATCGTGTATTAAATATAAAAGACTTGAAAAAATGACGTTTAGATTTGACTAAATTTAAACGATCGTTTACAAACAAAAAATAGTCAAATCTAAATTACAATCGTGTACAAAGAATCTTAAAAATCGTTTAGATTTGAGCAGTCAAATCCAAATGATCGTGTATCAAATGAATCTTAAAAAAAATCGTTTAGTCAAATCTAAATGATTGTGAGTCTGTCAACTTTTTTCGTTTTCGAAATTGTTTTATAAAGTATAAATATTTTACTACTTTGTTATATTTTGAATTGAAAAAGAGTCTTGTTTTTTCCTAATACTTTCGTAATATTTCTAACCAATCCATTGTTGTCTTTTTATCTTAAAATTTTCATATAAGACATGATCCACGACGGCCGATATAATATTAGCATTGGTTAACGTCTAACAAACTTTTCCACCCCTCCAATATAATAATCTAAATAAATAAATAAATAAATAATAATAATATTATTAATAATAAAGAATAATAAGTGGGGTTAAATTATTTGTCCATTTTCAGGTATTGACCACATTATTTTAACATAAGAACAAACATTTTCATTTTCTCGTGTTTTTTATATAGTAATACAAGAAATATTGATGTTGATCCGAAAATACATACAAGTTGTCTATATGGAAATAAAAGGAAACTAAATCTTTTTTAATTAAGCATGTTGGTGTTGCGTGCAAGCATTGGTAGCCTCACCCCATTTTTCAGTGTCCTCTTCTATTTCTTTATTCGTTACTCATTTTCAAAATATGTTTACTGGTTGTCGCTCCCCAACCTACGATGACACTCATCTTTTATATATACTTTTTTCAAAATTATTTCACCTAAAATAATTCACATAACCTTGTGATTCACAATCATCGCCCTACCGTAGTATTTATGATTTGTACTTATTTCTTTGGTTTAACTTCTTTGAATGTTTGATTTTTAAAATATATTACAACGTGTAAAGTTGAGACCTATGATAATTAATGTATTGATAAAATGAGTTAGTTTAAAAGAAGGGTATAGTTATAATAGATAATAATTTTAGTAATATAATTATCAACTATATTGTCTTGCCTAACACATTACCTTTCAATAATCAAATGTGACTAAGGGTTTTGCTTAGATTATTTGTCAAAGAAAAAAGTATGTTTTTTTTTTTTTTTCAGAAAGTCATTTTTATTTAAATGTTTCTTAAATGCCCATGTGTTTGGTAGTGTTTTATCCATGCAGGGTTATTTAGTATTTAGTTTGCTTTTACTCAAAAGTGTTTGTATTTATGTCTAAATATTTTGAAGTAATAATAAGCTCACATTGATGTACATAAAAATGCCTTTGCTATTCAAAATTATTTTTATCTTGTTTTCTTTTTATTCGTATGTAATTAGGGTGGCATATACCTACTCCCTCAATGCATTGATCTTGAATATTCAGTCGAATTATTTGTTTATGTAGTAGATCTTGCTTTGTCTTTTTAATATACAAAAATTGACATTGTTGATGATTGTTAGAATTACGATTTAAAATGGCTAAACTAAGCATACAACAAATATACTATTATTTTTGTAAGTTTTTGTAGCCATTTAAAACACGATCAAATTTGCATATTCACCATACTGACAACTAACACACTGGTACTAATTTGCTTTCAATATGCAG

CCTTGAATCTGATGTCATAAAAGAAGTCACAGAAATCATCTATAAAGTCAAAGCATCTCCTAAACTGGTTGGAATCTCTCCTCCATTGCATCAAATGGATAACCTTCTTAACTTAGGCTCAAATGACATACGTTTTATTGGAATAGTTGGATTGGGTGGTATTGGTAAAACAACCATTGCTAAAGTTCTTTACGAGAAGCTTGAACATAAGTTTAGGTACTGTTGCTTTCTTAGGGATTTTGACCAAAAATTAGTCTCAATTCAAAAGAAACTACTTTTGAGACTATGTGGGAAATTTGACATAGTAATTAAGAATGAGGATCATGGAGCAAAGTTGATCAAGAAGTGTTTGAGGAACAAAAAGGTTTTGATTGTTCTTGATGGGGTTGATGAAAGAAGACAAATAGAAAAGTTAGTTGGATCAAGTCAGGATTGGTTTAGTCCAGGGAGTCGAATCATCATTACCACTAGAAATAGAAATCTTTGTTGTCAACCTAAGTATAAACATAAAATGTTGGAGTACAATGTTGAATTTCTCGATCACCACAATGCCTTCTCGCTCTTTTGCAATCATGCATTTGGAGAAAATCACGAGCCATTGGATGAGAATTTTAGGTATCTTGCTGAGGAGATGGTAAAAATGGTTGAAGGACATCCGTTAGCTTTGATTAAAATTGGATCCCATCTACATGGTAAAGATATAGACAAATGGGAAGAAAGTTTAGAGAAGGTTCATAAATTACTTTACAAATATCTTTTCTGTGAGGTATTCAGGACAAGTTATGAAGAATTAGATGATGAGAGCCAGCAAGTTTTTCTGGATTTGGCATGTTTCTTCAATAATGGGATGAGTATGGATAGAGCGATTGAAATACTTGAGAGTTTCGGTTATAGATCACCCTACAATAAGTTAAACTTGTTGACTCAAAGAAATTTAATTCAAGTTTCTCATGGAATGGTACAAATGCATACCTTGGTTCATTGTATGGGTCGAGGAATTGTACAAGGTGAGAGAGAAACTCAAAGTAGAATTTGGCTTCGAAAACATATCCGTCATAT

GTTTGGCAAAGAAAAGGTAAGAAACAATTATTAGCTTTGTTTCATTTTAGGTTACGTTTGGAAGTACATAAATATTAACTTTTGTTTTGATCAAAATCAATACTTTGAAGTTTGAATATCACTCTCAAACATACACAACGTACTCACGTACAGTTCATTGCTGTTTTCGTTCAAGTAATTATTATTCTTGTTTATCTTTTCAGGGATTAGAAGATGTTGAAGGAATAGTAGTTATGGACATGGAGGAAGAAGAATTAG

TATTGGATGCGAAGTCATTTGCATATATGAACAAGTTGAAGTTATTAGAAATCAACAATGTGAGGGTTGATGAAGATATTCAATTTTTGTCAAATAAATTGGGAATTCTCAGGTGGAACGGATATCCTTCAAAGTATTTGCCCTCGACTTTTCAACCACAATCTCTGCTTGAATTGCACTTGCCCAATAGTAAAGTTGTGCGACTTTGGGAGGGAAGAAAG

GTACGTACATATATATACATAATTAATTATATAGTTTAATTGATTACTGTTTGACACCCTCTACGGCTCTACCTATATATATCATTAAAAATGCCAACACTTCCTAACTATTTAGTGAAAACTAACGTTTTTAAAAGTATAGGCCATCTGGATATCAATCCGCTAAATTGAAATAAAACATAAACTTCGATCATAAAAATGAAACATCTTGTAAGAGAAATTAAATTGAAATAAAAACTTAAAACTGCACTAAAATTTGAATATTTTGAAATTTAGAAACTGAGACAAGAAAGGTACTTTTGTTTGCTCTTATATTTTTGTAATTGCAATAAGTAGTGATTTTAGAGCTAATAATCGAGTGTATGAAAATACTTTTAAAAAGTTACAAATATAGCAAAATTTATTAAGGATCAATTAGATCGAATATATGGTTTTGTCTGAAATCTCGTGGCAATATCTATGAAACAAATAGGGACATTGTTCTAAGTGATACACTTTGTATCTACGATAGAATCCTATCAAATGATAGATTATATAGTTGATAGGTTCTTATTAATGATATAATCAAAAAGGCTAAATCTAAATTATACTTGATTAATTTTCAACTATTTTTTTTTTTTTTTTTTTTTTTTATTTTTTAGGGTAAGAATTTGAGAAGTAGAATGATATCTAATAGGATGTTGTGGACTTGTTTTTGACAG

GAATTTATTTGGTTGAAAGAGATTGATGTGAGTGGATCAGAGAATTTGGTTGAGACCCCTGATTTCTCAAAGCTTCCAAATCTCCAAAGGTTGATATTAAGGAACTGTGCAAGATTGTGTGTCATTCATCCTTCCATTACTACTCTCAACCCTCTTGTTTTAGTTGATATGTCAAATTGTGTCAATCTCAAAACATTTCCATCTAAACTAATAACTTGCAAAAGACTCCAAACGTTGGTTCTTTCCAATTCAGGCCTTAAATCTTTCCCAAAGGTTGAAAAACCAACCAAATCTTTAACTCAACTTCACCTTGATGGAACTCTTATACAAGACCTTCCTTCATCATTTGGACTTCTAACTCACTTAACTTTATTGAACCTAAGGGATTGTACTAAGCTATCAAGTCTTCCAACATCAATTTGTAAACTGATATTACTTCAAACTCTCAATTTGAATGGCTGCAAAAACCTTCACCAAATTCCTTTCACTTTGGGGACAATACAATCTCTTACCATGCTTGACATTGGGGAACATTTATAGATCAAGCACCAGATGCTATCATTTGTTTAAGGAGTCTTGAAACTTTGAATTGTGAAAGGCTATCAAGAAATATTTGGTGGTCATTGAGTAATTTGGTTGGGAGTACTAATGGTCTTTTGCCTATTAGAGATTTGAATCTAAGTGATTGCAATCTTGTGGACGAAGATATTCCTGATGATATCAAATGTCTTTATTTATTGGAAATTCTTGATCTTAGCAAGAATTCTTTTGTAAGGCTCAAACAAAGTCTCACTCAACTTACTAACTTAAAAGCATTGTACTTGAATGATTGCTTTAACATTCAGCCACAACTATTGCCAAAGCTTCCAACAAGTTTGCAATATGTGGGAGGACAAAACTCGAAGGTAATTCGTGTATCCTTAATCTTCATCTTTTTTTATTATTACCACTATTATATTGTTTTAAATAAATTCTCTCTTCAGAGCAGCGTTGATATAATACTAAATTTGTCTCACTCATCAACTTAA

>MELO3C022144

ATGCAGAGTTCATCATCGTCTTCTTTGGATCGTCCTAAGATGAACTACGACGTGTTCATAAGCTTTAGAGGTAGAGATGTTCGTCACACTTTTGCAGGATATTTGTATGATGCTTTGAATCGTTTGGGGATAAAAGCTTTCCTGGACAACAAGAGGTTTTTAATTGGAGATGATCTTCATGACTTATTCAAAATAATCGATGAATCAAGGTCAGCAATTGTTGTTCTTTCAGAAGACTATGCTTCTGCTAAATGGTGTTTGAGAGAGTTGACTAAGATAATGGATTCCATGGGAACCTCGATGGAGCGTGTTCTTCCTGTATTTTATCATATTGATCCATCAATTGTTAAAGATCAATCTGGAACTTTTAAGACAAGTTTTGATGAACATGAAGCCAATGCTTTAAAGGAAATTGATAATCAAGAGAAGGAAAAGCGCTTGAAGGAACTCCAGAATTGGAAAAATGCACTGAAGAAAATTGGCAATCACACTGGAGTTGTCATCACTAAGAACAG

GTAAATTTCAAACGACTATTTGTTTTTTCTTAAAAATTACCATCTCTTTTGATTGTTTTGATTTGGTCTCTTATCAAATTTTAATCTGCTTCCTATTCTCCAGTGTATATGTGAAGATAAGTTGAATATTTGTATTTGCACTTATTGGGATATGAGTTGGATATTTGTTGGTTGTTCCAAGAGACTCATATTAGCACGAGGATTAGAGAGAAAGTTATAAAATTATGAGATGACTAAATGTTACAGAAATTTCAGCTATCATTTGTATTAAAATCATAATATTATATATGCATTGATCAACTTCTGAAAATTGAATATTACTATTTAATTTACAGCATTCACTTAACATTAAGTTTAATTATATTTTGCAG

TTCTGAGGTAGATATAGTAAATAAAATTGCAAGTCAAATATTCGATGCATGGCGTCCTAAGTTGGAAGCATTGAATAAGAATTTAGTTGGAATGACATCCCGATTGCTCCATATGAATATGCATCTTGGTTTAGGATTAGACGATGTACGCTTTGTTGCGATAGTAGGAATGGGTGGTATTGGTAAAACAACTATTGCTCAAGTCGTTTTTGATTGCATTCTTTCAAAGTTTGACGATTGCTGCTTTCTAACGTTACCTGGAGGTGATTCAAAGCAAAGTTTAGTGTCATTACAACGGGAAATGCTTTCTCAAATTTTTCATAAAGAAGATTTTAAAATATGGCATGAGAATCATGGAGTAGAGATGATTAAAAATCGACTGAGTGGTAGAAAGGTTCTTATTGTTCTTGATGGTGCCGAAGAGAGAAGGCAGTTAGAAATGTTGGCTGGAAGCACTGAGTGGTTTGGTCCTGGAAGCAGAATCATCATTACAACTAGAAATAAAGGATTATTGTGCCATCCTAATTATGATGAAATGAAAGAATACAATGTTGAGGAACTAGATCATGATAGTGCCCTTCAACTCTTTTTGAAGCATGCATTTGGTAGTAATCATCAAAACAAGGACAGTTTCATGGATCTTAGTAACGAGATAGTTGAGAAAGCTAAAAGACTTCCATTAGCTTTAAGAGTGATTGGATCTTCTTTGTATGGTAAAGAGATTACAATATGGAGAGAAACGTTGAAAAGGCTGATCAAAGTGGATGAAAGAAATTTTTTTGATATATTGAAAATAAGTTATGATGGATTGGGAGTAGAAAGCCAACAAGTTTTTCTTGACATTACATGTTTCTTCAATGGAAAAAATGAAGATAGAGTAAATGAAATATTGGAGAGTTTTGGTTATAGTCCTAATAGTGAACTACAGTTATTGATGCAAAGATGTTTAATTGAAGTTTCACACAAGAAAATATTGGTGCATGATTTAATTCTTGAAATGGGTCGAGAAATTGTGCGTAAGGAGTCCCTCACTCAACCAGAAAAACAGAGTAGGATTTGGCTTCATGAAGATCTTTACTGCAGGTTTGCTGAAAAACAT

GTAAGAAAAATTGGTTAACTTTATAATTCTTGTACCTAATATTCTTCTATGCCTTCAAAAGATAATTATTATTAATGTTTTTTTTAATTATTGTTTGTTAG

GACTTAATGCATATTCAAGGGATAGTTTTAAGTTTGGAAAAAGAAATGGAAGAATCAATAGAATTGGATGCCGAATCCTTTTCAGAGATGACCAAACTAAGAATACTGGAAATAAATAATGTGGAGCTCGATGAAGACATTGAATATCTCTCTCCACTTTTGCGGATAATTAATTGGCTTGGCTATCCTTCGAAGAGTTTGCCCCCAACGTTTCAATCGCGGTATTTGTTTGAACTACTCTTGCCTCATAGTCAACTTTTACGAGTTTGGGATGGAAAAAGG

GTTAGCTTTTTAAAAAACATTTTAGTTTTTAATACTTTTTAGTTTGTTTTTGATTTCAATTTGGTCCCTATGGTTTTATTTATTATACATTTATTTTTTCTTAACAG

AGATTTCCGAAGCTGAAATTAATTGATGTTAGTAACTCAGAACACTTGAGGGTGACACCTGATTTTTCTGGGGTTCCAAATCTTGAGAGGTTGGTTCTATGTAACTGTGTTAGATTGTGTGAGATTCATCCCTCCATCAATTCCCTCAACAAACTCATTTTACTGGATTTAGAGGGTTGTGGTGATCTTAAACATTTTCCAGCAAATATAAGATGTAAAAATCTCCAAACACTCAAACTTTCTGGTACAGGTCTTGAAATTTTTCCAGAGATAGGACATATGGAACATTTGACTCATCTTCATCTTGATGGATCCAAGATAACCCATCTTCATCCTTCAATTGGGTATCTAACTGGCTTAGTTTTCTTGGACCTATCCACCTGTTTAGGCCTTTCTAGTCTTCCTTTTGAAATTGGTAACTTGAAGTCTTTGAAAACCCTCCTTTTGAAATATTGTAAAAGACTTGATAAAATCCCTCCAAGCTTAGCAAATGCTGAATCCTTGGAGACTCTTTCTATTAGTGAAACCTCAATAACCCATGTTCCATCAAGCATTATTCATTGTTTAAAGAACCTAGAAACGTTAGATTGTGAAGAACTATCACGTGGAATTTGGAAGTCATTGCTCCCCCAATTGAACATTAATCAAACAATAACTACTGGTTTGGGTTGTCTCAAAGCTCTAAATTTAATGGGTTGTAAACTTATGGATGAGGACATTCCTGAAGATCTCCATTGCTTTTCCTCATTAGAAACACTAGATCTCAGCTATAATAACTTCACAACACTCCCTGATAGTCTTAGCCACCTCAAGAAGTTAAAGACATTGATCCTGAATTATTGCACTGAGCTCAAAGACTTACCAAAGCTTCCAGAAAGTTTGCAATATGTAGGAGGAGTAGACTGCAGATCAATGTCAGAACAATATTATAACAAAATTTTGCTTATCCCTTCTAGTTCTGGACACCAACTTTACCTTACTTTTATCATTCCTTCCAAGGATGCGGATGTAGAATGTGTCATGAATGAGTTCCAACATTCGATATTTACTCGAAGATCGTTTGAG

>MELO3C022145

ATGGCTGCAGGTTCCTCATCTCATCCTTCTCAAATGGCTTTTGATGCTTTCTTAAGTTTCAACAGAGACGAGGAAGACGATGGCTACAGATATTTCATTAAGGGTTTATATGAGACTCTTAGTGAATGGGGAATCAAGTTGTTTATGGATGATGGTAAGAAGATGTTTACTGATGACGAGGTTAATCTTAGCGATAATATAGTGAAAGCAATTGAAGGATCAATCACTTCCATTGTTGTTCTATCAAAGGGGTATGCTACTTCCAAGTGGTGTTTGAGAGAGTTGGTTAAGATAATAGTTCAGAAAGACAAAACCAAACACCAAGTCCTTCCTTTGTTTTATGGCGAGAAGTACGCAATAAGACCTGCAATTTTCTCGTTCGGAAGATATGGCCCAAGTCCATTAATAGACGTCAGGAAGATCGATGGTTATAGCAAAAAGAGCTCTAGGGAAGCTAAATGTAAAGCTGCTCCTGAAAATTTCATTTATAGTGAGGCAGAAATAGATTTGATGAATGACCACAAGTCGAGGTTGGCTCTGTCAGAAGTTTTCCGTCTCCCTGGAGTAGATATACCCCGTTTTCCTGGAGTAGATGTACCCCGTTTTCCTGGAGTAGATATGCAACCAAAATTTCT

GTAAGTATAAACAGATCTTTTAAACCATATTTACTATGGCGTTATATATCCACTTTTAGTTTCGATGATAAGTACAAAAGATTGATTTGACCTCTAACTTGCCCCCATTTCTCATTCTATTACCATTCTTTTTTTATCTTTGATCGATTCGATTTCGTTGCTCAAATTTGAATCAAATAATAATTGAATTATGCACCAAAAACTAGTCCGGGTGTTGAACTTAGGGAGAACGTTAGTTTCTCAAACCAAATTATTTTCCGTCCGAGGAAACCAAGGGGGGTTTGAATGGTTTTGATCACAAAATAATGGAAAATAACGTAAGAATAAAATTTAGAAGATGTTCAATAACTTAAAAATCCTAGCTTGGGTCTAAAGGAAATTCCCTATTTAGATTTATTCGATTGGCATTACCATTACTAGACCTTAATATTAAATCTCAACCAATTTAATTAGATCTATTAAATCTATACACAATCAAGTGTCACAATATTGTATAAAAGTTCAAGTGGAAAATTGTTCGCACGGATTTCGTCGCCAAACCAGTGCCAAATCATAAATCAAATCACACACACCAAAAACTAGTCTTTGTGGCAAAATCTAGGTCGATCACAAGGAGTAGCCAATTCTCAACAAATTAATTTTCAACCATGAGGTAACCGAAAGGGAGGATTTGAGTTGAGATTTTGAGCAAGTAAATACATCTAGTCAGGCAACAAAAAAATAAAATTTAGGAATTCTATTTTAAATCAATCTAGTTTGGGTTGTTGCGATCTCTCCCTTGTCCTAAGAATTTATTCATAGAAGTATCATATTACCTAGTCATGCATGTGTGACCTTCGATCACCTAACCAATTTAATTGGAACTGTCAATTAAATCGTCTTAAGGTTCTAAATGTTTAATCTAATAGCTTGCAATATTCAATTTCTCAATTAGAAATCAAATTACTGAAAACTAAATAAAGTTTAAATAAATGTAATGGTTAATCATGCTTTCTAAAAAAATACAAATGCAAAGAAAAAGGAATTTACTCTTGGTGAAGTCACTATATCTACTACCATGACACCAAAATTGCAATCACCACCACTTGGAACCTCCTTCTGCTTTGAGTAATGGAGATTTTTGTTGGTTGGGAACCAAAAGAATTTTTGGGAAGAAGAGAACTTTTTTTTCTTTTTTATTTATGAGAGAATTACATAGAACTTAGAATTGCTTCGTCTTGTATCTACTACTTACTAAGGCGACAGAGGCCAAAGATTGGGATAGGTGGGATGTTTTCCCACATTCCCGAAATAACTCCCACGTCATTTAATTAAAATTAAAATTAAATAAAGTTTACATTTCATAAATATAACAAAAACACAAAAATATTTACCGTCCGTGTAACAAAATCAATAAACTTATGAAGCCGGTCATTTTTTAAAATATTCTAGGTTTGCCCTTCCTTTCCATCTTCTTTCTTCTCTTTTCCTTTCCATCGCTTTTTTCTCTTCTTCTGCAATTTTTCTTTTATATTTTTTTGAAATCATGATTTTTGTTCTTTCAATCCTAAATCTTGTATTATTTTTCTTTCAAACTGTTATTTGGTTCAAGATCGTGTAATATAAAAGATCAATCATTGGGATATTGGCTAATACTAAATCTAATTAACAAATACAAAAGATCTTGAAAAAAAAATGATTGGATATTGGTACAAATCTAAAAGATCGTGTCCCAATCTAAACAATCGTGTAAATATTAAAAAAATTGTTGGTACACAAGTCAAATCTAAACAATCATGTACCAGCGAATATATTACGTGCAAGTGGCTTATTAATCGCATGTTGGCAGGAGCATTTTTTTTATTTTCTATTGTGGGCCTGTGACCTTTTTCCGTTTTTGAAATTGTTGTATACAGTGTTAATATTTTACGGTTTGTTATTATTTTTAAAAAACCACTTAAATAAATTAATATTAAAATAATTAATTAAATCTTATTTAATTAAGATTATATTTAATTAAAATATAATAAACCGATAAAATATTTACACTTTGTATAACAACTTCGAAAATAGAAAGAGCTCATAGGCCCACAAAAGCAAATATAAAAAATACTTCAGTTAAAACTTCAGTTAAAACGCGATTAATCTACCATACGCACGTGTGAGTTTTTTGAAAAATGATACTATGGGTCAGTTGGGTGCACCCGGGCATCTCCACTAGGTGGACACCCCCTTAGCACCCTTATCATTCTCGTTTCATTAATAAAGATAAGAATTCAGTACAAGAAGCAGGATAAGGATACAAGCAACGAAGTACAGGGGCTAGAGATAAGCCCAATTAAACAAAAGCAAATACACACTACAAGAAAATTGAGCGTTCCCGACGCAGAAAAGGACGTCGGCATCAAGAACGTCAGGAATAAAGGCTTTCCCGACGCCGTCAACAATGCGTCGGGAGACGCGTCAGGAAAACACTCTTTCTCGATGCATCACGAAGGCGTCGGCAAGTATACGTCGGGAAAAGGGTAAATTAATTTTAAAAAACGAACTATTCCCGACGTCGTGAACAGTGCGTCGGGAGCGGCATCGGGAATAGGTGTTTTTCCCGATGCAGCATCAAACGTCGAGCAAAACGTTTAATGAGCGTCGGGAATTCCCCTTTTCTCGACGCTTTTTGGGGGATTTCCCGACGCCACATCACTGCGTCGAAAAATCCCCTTTAAATCGTTTCATTACAGAACCGAAACCGAGAGGGGAAAAGAGAAGACAAGCCGAAAGCGTCGTCGCCATTTCCGTCGCCCTTCCGCCATTGTTCCGCCACTGTCCATCGCCGCCCTCCCTCGCCGTTGTCCCTCGCCGCCGTTTGCCACTTTAGGTAAGTGGAAAATGGAAATTTATGTACACTTATCGCAATTAAGCCTGGATTATATAATTTGAGAAAAGCTTTGATCTACTGCTGCAAAAGCCAGTTAACGCAAGAATATCTTCCCAAAGTTCTGCTGCTTCCTTCTTAGCTTGACTGAAGATCCTGCTGTTTCTTTCTAGCCATATTTTCCATAATATCACAGCAAGAGTATTAAACGTAATGGATCCTTTTTGGCTTTTGATATTTATACTACATATATCCTTACAAAGCGATGCCACATCAGTAAGGGAGTTATTCCATTTCAGAATAGACTTGGCCTTAATCCACAGTTTCTTCGAATAGGAGCAGTTGATGAAGAGATGGTTAAGATCTTCCTGTTGTTTGTTGCATAAATAACACCAATTCGGATTCAGATACCAGTTTGGGAGGCGTGTTTGCAGTTGATCAGCCGCATTAATACATCCCTGTATAAGCATCCACAGGAAAATTTTGCATTTTTTTGGAATCTCAGCCTTTCAAAGAGTTCTGTATAAATTTAATTTGGTTGGAAATTTACAAAACAAGGATCGATTTCAGCAATTTCTTTTTTGACCGATGTCGTGTCAAAGTTGCCTTTTGTATTTAATTTCCAAATAGGTCGAGAGGCTCCTCTTTCTGGCAGAGGCACTGTAAGCCCATATTTAATATCATCCCATAACTGATTTTCATGTTCGCGAAGAGGACGGTTGATTTGAATATCCCAGTCATTGGTTATTGGGTTCCATACATCTTTCACGTTGCCATTTTTAATCTTGGATAGAGCAAATAGTCGGGGAGCATATAAGGCCAGGGGGGCTTTGGTATTCCAATTATCGTGCCAAAAAGAGATTATACCCCATCGTTCACCTGCCATTCGATGTGATTATTGAACCAGTTAGCACAATTAGCTATAGCTCTCCAAAGAGCCTTGTTGCTGCCAAACTTTCCCTTAATTGGGATTTTGCCAACAAATTCCTGATTATATTTGGCGATGATCAGTCTTTTCCAAAGGGGATCCTTTTCATACAAGAATTTCCAAAGTCATTTGCATAAGAGTGCGAAATTTGTGTTGGTGACTGTATTTATACCCAGACCCCCCATATCCTTCAGCTTTGTGATTTTAAACCATCTGATTAGGTTGATATTGTGGCTATTTGATGCTCCTTTCCATAAAAAATATCTCCAAGCCAACTCTATTTTTTTTCTATGCCTTTAGGAGATTTGAACACTGACAGTTGATACGTAGGGAGACTTTCTAAGGTAGAGTTGATCATAGTGATTCTACCCCACTTAGAAAGATGAGAATATTTCCAATTGCTTAACTTTTTCTGTATCTTCTGTAGAATGTTATCCCATAATTTAAGTGAAGATGGTTTTGCTCCCAATGGCATACCGAGGTAATTAATCGGCAAGTATTCCTTCTTGAGCCCCCAACTCTCAACAACCGAATTCGCTCTACTAGTGTTCACATTTATGGGAGAAATTGTGGATTTGTTCTTATTAATGTTCAGTCCCGAAGCCGATTGAAAGAGGTATAGAGCAATCCTTAGATTTGTGATGTATTCTTCACTATCTTCAACGAAAATGAGTATGTCATTTGCAAATAGTATGTGTGTAAGGTTGAGATTTAGGCCCATATTGACACCTCCTATTTTCTCTTTTTTCTCCAAATCAGTGATTAGCCTGTGAGGTAATCCATTGCCAATACAAATATAAAGGGGGATAAAGGGTATCCCTGTCGAATTCCCCTGGATGGTTGGATTCTACCTCGAGGTTTGCCATTGATAAGAACAGAGTATTGTACACTTGAAAGACAATTTGCAATCATTTTCCTCTAATCTGAAGAGTAATTCTTTTTCATCAGAATATAATCAATGAAGCGCCAGCTGATTTTATCAAAAGCTTTTTCAATGTCCAGTTTGATAAGAAAACCTTTGATTTTTTTGGCTCTCCAAAAATCTATTACTTCATTTGCGATCAGTATGGCCTCAGTGATTTGTCTTCCTTTAACAAAAGCCATTTGAAACTCAAATATAGTGTTCAGCAGTGTGGGCTTCAATCTTTCAGCCATGACCTTACCAATCAATTTATAAACAGCTATAGTGAGGCTGATAGGACGGCAGTCTGTGGCTGATTCGCACTTTTCTTTTTTCGCGATGAGGACTATATGTGTGTCGTTGACCGCTTTATTGATAACTTTGCATGAATGGAAATCCTTGAATATATCCATGATGTTTTTTTCATGAATTTTTAGGACTTTTTCAGGAATTCCATAGTGAAGCCATCCGGACCCGATGCTTTATTATTTTCAAAGGACTTCAGGGAGTTCCAAATTTCCAATTCATTGAAAGGATTTCCAAGCTGATGGTTGTTGTGGTCATTAATTGGAGCCCAATCAAGGTTGTCTATCAAGATTTGATTTTTTCTATCGGCAGAATATATCTTTTAAAATGAAGTACGAATGCCTCTTTTATATCACCATCTTTTCTATAATTTTTCCCCTAAGAGTTAATAACATTAGATATAATGCTTCTCCTTTGTCTGGTTGAACAGATTTTATGGAAAAATGTGGAGTTTTCATCCCCCTCAAGATTCCACATTCTCTTACACTTTTAAGCCCACATTTGAGTTTCTTTGAAGGTATCTTGACAAAGTTCAGTCTTTAGGGCCGTTCTTTTGTTGCTAAGTACTTTTGTGATTTTTCCTTCAGCCTCTAATTTGTCAATCATATCAATCTCCCTTATCCATTCACTTTTGTTCTTCTCGGTCTGTCCCTTTTTATTCTTGCTCCATTGTTTAATTTTGGTAGCTAGTTGCTTAAGTCTTCGCATAAAAGAGTATCCAGCAAACCCAGGTTGATATGTATTGGTTCACCATAATTCCAAATTTTTCTGGAACTCCATCTCATTAAGAAGAGCATTAGTGAACCTGAAGGGGGAGGGGCCCCATTTAAAGGAAGAGCATTCAAGAATGATGGAAAATGGTTTGAGCTGATTCTGGAGAGAGTTTTAATGTAATGGCAAGAGAAGGATCTTTCCCATTCAGGAAAGTATAGAAATCTGTCTAGTCTGGACAGGGTGGGCTGAGCTCTTAGATTGGACCAAGTGAATTTTGCATTGGTAAGAGGAGGGTCTATTAAATTGCAACTGTTTACGAACGTGTTAAATTTCCTCATGCTAAAAGAAGTTGGGTTCTTGGCAGAAGTTTCTCCATTCCATCTTACAACATTAAAATCTCCCCCAAGAATCCAGCTAAGAAGGCAAATGGCTTTGAGATTTTCAAATTCCTCCCAGAACATTGATCGATTTTTCCTTTTAGCAGGTCCATATATGGCCGACAGCCACCAGCTGGTTCCATCTTGATAACAAATTTTGATTGATATAGAGAAGTTGGTGTTATGTATGGAATGTCTAAGGTCATCCCACATAATAATAATGCCACCGGATCTACCGTTTGATTTAAGAAAAATCCATTTTATGTTGAAAGAGCTCCAAAGAGACTTTATAATTTCTTTTTTTTTTTTTAACATTGGCTAATTTGGTTTCAGTCAATATTACAAAGTAGGGACAATAGGCATAAATAACTGATTTTATTTGAGCTCTTTTATGGGCTGAGTTTAGCCCTCTAACATTCCAGGAGATCATCTTCATGGGGAAACAATTTATCCCATGGTGGATTGTTCCTCATCAGTTATTACATCTTCATTTTCCCTTTCAAAGATTAAATTTGGTGGAACAAATTTTATAGGTTCTACAATATTCACACAAGAGGGGGTAAATTCAGAATTAAATTCAGAAGTCAGCCTAAGATTATTTTCATTGAGCCACTTGGTCAGTTTCTTCCTAAAGGTCTCTTCCTCTATATTCTTCTCATCATGCTCCTCTTTGCTTTGTTTACTCCAATCTTGGTCACTCGTGACCATGTTCCCCAGGTTGTCCTTCACAATTTCATATTTGGTCGGAGACGTAGGTGATGGAGTGTACATTGAATCCATAGGACTAGAAAAATTTGTTTCAGAAAGAGGGGATATATGGCCAAAGTCAACGACCAATTCCATTACTTCTAACTTTTCTTTTCCCTTCAGTTTCTGTCTTTCTTTTCCAGCAGTTTCTTCTTCTGTTTTTTGGCTCTTTACCCGATAAACTTTCTTTTCTTTGTAAGTATCAGCCCGTTTTGCTCTTGCTTCAATGCATTTATTGGCATATCCCTTTAGGCTTTCGATCTTCAGGGGTTTTGTTGGGGCATTTTGTGTGAAATAATAGACATTTTTTGGGGAATTAAATGAAACTTTTCTTTTGGTATTGATAGGGAAGAGCTTTGGAGGGGACTCATCATGGCATATCTGTTTTCCTTTTTTCTTTTCAGTCATTTCAGCTGTCGGGCCTGTCTTCATACTTTCTGTCTTGTTATTGTTGTCGTTGTCTCCATCGTAATTCAAATTCAAAAAATTTCCAGTGGTATTGAATTTTTTATTCCTTTTTGTGGGCCCGGTATTTCGGCGAGCATTCAATATGGATTTCTTGTTCTTTGATGTTTCTGGTGAGGTCGCTCGGTTGGTAATAGCATGGTTTTTTTCAAATGAGTACTGTTCAGCATTGAGGTTGAACTCATTGAAGTTTTCAGCAGCTTTTCTTGTAAAAGTTCCATGAATTATGCGATTTCTTTCTCTCAGCCATCTTCCTTCTGGGTGGGTATTACTGTGTGAACAATATACTCATTTCCTTCTTCATCATGGATTTTAATAAAGGCAGGTAAGAATCCCGTGTAGTTGTCTCTCACCTTAATGAGAGCTTCTGTCAATTCTTTTTTGTCGGCAGTTTCTGGAGCCACTTCAATAAAACCACCGCATGCTTCTCCAATCTGAGAAAAAACTAACATATTCCAAAGATGTAACGGAATACCTCTAACTTTTATCCATCCACCGTAGCTAAGAATGAGTTTTGGATCAGCGTGTAAAACTTTCGACCACATTTCAAATTTAACATAGTAAGGGCCGACTGTGTTCCACCCTTTGTTTTTGTAGAGCATTTTTGCTAAGTTGTGGTCTTTAATAAAGAGGAGAGTTTTTTCAGCATGAAAGGGCCTGTAGCGAAAGTCGAATCCATTTGGTCTGTTTGTTCTCTGGGTTTTTCCTATAATTTTATCCAGTCGTCGTGAAAGTAACGCCTCGACAAGACAACCAACCTTGTCCCAGTCAAACTCTTCATTTTTGCTTTCTTTCTTCACTTCTTTTTTCACTTCAGATTTAACTGTTGGGAAGTTGGTTCTCCGAGTGGTTTTGTTGGCTGTAGAAAGATATTCTGATTCTGAGCTGGTTGAGCCGGACACAACTTCGGCGTAAGACCTTCTGGGGGAGTCCATGTCAGAGGATGTAGAATAATTCTTTGGACTTTTATCTCGACGTGAGGAGGTTTTCAAAACTGGCTCTTCTTTCTATATTCTTTTTGCAAGTCAACATATCAAAGAAATGGGCCCATCCAGATTTATCCATACCTTCTGCGACCAGTATGCAACATCTACGTTCCCTATCATCAACTCTATAGATCTCCGCGATGTAACCTTTCCTGTTGTAAATTTTCTTGACCCACAGGTAAAAATCGACATACCTTTTTTCATTAAAGAATCTTGTTGTTCGGGGGGTGTTCAGCAGTGCTTTGAAGGTGTTTTTTTAGCCATTCAAGAGCCTCAAGAGTAATGGCTATGGATAAGGACTTGTAAGGGCCTACCTCAGTGATTAAAAGGTTCAAATCTCTTGATCTTTTATCCAAGGAGGTCCAATGAGAGCACAAATTCTTTTTTCTCCACAGTACAGTAGAGAGGGAGTTGGTTAGGGAGAACCATTGGTAAAGAACAGTAGAGAGGAGTAGAAAGGAGGATAAAAGATTTCAGAAAGAGAATAAGTTAAAGGATGTGAGTAGTGTACCTTTTGTGTTGTGGAATTAATATGAACCTCCAAGCGAAGTTATTGGTTGTTGGGAGTAAATGATTCTAATTTGAAATCATCATGGATCAGTAATAGAGGTGCAGCTACCCAGTTTGTTGAAACGACGGCTACGGAGATGAAGCGGCGGATGGAGAGAGGATTGTTTGTAGAGAGAGAATCCATCTTTTTTTTTACACACGATTAAGTTGTCGTTCACTTTTTAAGTTGGACACGCACGTGTGAGTTATTAATCTTCTTCTAAACGATTGTGTACTACGCTGTAAACGAATGATCTACGATCGTCTAGGTCATGATATACGATCGTGTAGTTCCTTTTAAACAATGGAAAAATGACTTCTAATCTAAACGATCAAGTTAACCATGCTAAACGATCGTGTCAACTGTAGGGTCCAAATTTTTACCTATAACAGTTTCATAAAGAAACTGATTTGAATCAATTTGATAATTATAGGTTCTTTGAGAGACTCCAGCACAAACTCGAATTCTCCTTTTGAGTCACTTGAGTCTGAATTGCAATGCTAGTCCTAATCTTACTGCACCACGCACCAATTTCAAACCTAGAACTCAAACATGTATTTTGCGGCAAAAATGATTCCAACTTATAAAATGATACATAATGTAGTGATCAGATACTAATAAAAATATAACATCATTAATATCATTCATAATTTAAAAAGGTTAACAAATACGATGGATTTTGCATAGTGGATTAAAGACTTGGGTGTAGGAATTCCTGTTAAGAGAGAGGAGAGAAAAAAAAAGCCGGCGTTGAGTGTTAAATGCTCATAACTTTTAACGCGAATAAAACCATCCCAAAAAACTTTAAAAAGGTCACTACTTTGGTTACAAAAACTCAAAAATTAAATCACAAATAAAGTTTAATAATAAAAATTTTATATTATCGTTAATAATGAAACCTTTCATACTGTCAACAATTCCTTCGCCCACAAATTGTCTTCTATTTTGTAGGATTGTTTTCCTATATATATTTGGTTACTGGCAATTAACTTGACGGAGTTTGCACATGCAG

CTATACAAATATCCAAATCAAATCCATCGCAAAGCAGATAATTGATCACTTGCTTAGTCTTAAGCTCGAAGCCAAAGAAGGGACTTTATTTGAAATGCCACCTCGATTAAGAACAATGGAAATGCTCTTTGGCTTAGGCTCAAATGACATACGTGTTATAGGGATAGTAGGGATGAGGGGTATTGGTAAAACAACCCTTGCGGAACATATTTTTAACCATTATTTTAAATATTTCTCAGTAGGAAAATATTGCTTTCTTCACATTGTTGGACGCAGTATAGTCTCCTTGCAACAACAACTACTTGATCAACTTGGTTGCTCAAATTTTTTCAGTTACCAACTTTGGGATGAGGATCTGCTAGTAATATTTATGATGGAGTGTTTGAGTTCACTTAAAAATGTGCTTATTGTTTTTGATGGAATAAGTGAAATAAGTCAACTAAAAATGTTAGCTGGCAGCCCCGATTGGTTTGGCGAAGGGAGTCGAATCATTATTACAACCACAAATAAAGAGATTTTTCGCCACCCTAATTTCAAAGACAAAGTGCAAGAATATAATGTAGAATTACTTTCTCATGAGGCTGCCTTCTCCCTCTTTTGCAAGCTTGCATTTGGAGATCACCCTCCTTCCGAGGATATGAAGGATCTTTGTAATGAGATAATCGAAAAGGTTGGAAGACTCCCATTAGCTTTGGAAAAAATAGCTTTTTCATTATATGGTCATGACATGGATATATGGGAAGATACATTGAAGAATTTTCACAAAGTAGTTTATGATAATATTTTCTCTGATATATTAAAGTCAAGTTATGAAGGATTAGAAGCAGAGAGCCAACAAATTTTCTTAGATTTGGCATGTTTCCTCAATGGAGAGAAGGTGGATAGAGTGATTGAAATACTTCAAGGCTTTGGTTATAGCTCACCTCAAACTAATTTGCAAATGTTGGTCGATAGATGTCTTATTGATATTTTAGACGGCCATATACAAATGCATATTTTGATTCTTTGTATGGGCCAAGAAATTGTGCGCCGCAAGATGGGAAATTGTCAACAAACTAGGATTTGGCTTCGAGATGATGCTCGTCGTATATTTCATGAAAACAAT

GTAAGACATTGTGTACTTTATAATGCTATATTAACTATTGTTTTGAGTCCTTGTAACTAATTATTGCTATATTAATTATGTTCTGGGTTTGCCAG

GAATTAAAATATATTTGTGGAATAGTGATGGACTTAGAGGAGGAAGAAGAATTGATATTGAAGGCCAAAGTATTTGCAGATATGTCTGAGCTAAAAATTTTACGAATCAACAATGTGCAACTTTCGGAAGATATTGAATTTCTGTCAAATAAATTGACATTGCTCAACTGGCCTGGCTATCCTTCAAAGTATTTGCCATCGACATTTCAACCACCATCTCTGCTTGAGTTACACTTGCCTGGTAGTAATGTTGAACGACTTTGGAATGGAACACAG

GTTAGTATATATATATATATGCTAATTGATGTATTATTTGTCTCTAAATTTTGCATCATATGCTAAACTTATTAAATGTGTTGAGGACTCCTTAAATCTTTGTCCCATATATATTTGAAATGAATAACAAAGTCACGAGTTCTGATGTCTGAAAGAATTAGGATGAAATCCTACTTCTATGTTTCTTTTCATATTGTTGTGTATTCCATATTTTTCTTCACCTAAAAGATAATAAAACTGTTTCTCCATCAGTCTGCAGACGTAATTAACACATAGTGATCAACATAAATCTTTAAGTCAAATTCTTCTTTGCAAATCAATTAGTTTCTCTCATGACAAAGCTGAATCTTATTTGAACTATATACAATAAATTATTATATATGTCTGCATTACAAAATTAATGTCTAACATAGTAGATGCAATTCATTTGTTTTGCAACAGAAATTTAAGAACTTGAAGGAGATTGATGCAAGTGATTCAAAATATTTGGTTGAAACTCCTAATTTTTCAGAGGCTCGAAACCTTCGACGATTGATTTTAAGAAATTGTGGAAGACTAAAAGAGGTTCATTCTTCAATAAATAGTCTCCATCGTCTAATTTTATTTGATGTGGAG

GGTTGTGTCAGTTTCAAAAGCTTCTCATTTGTTATCACTTGCGAAAGTCTCAAAACTTTAGTTCTTTCTAACTGCGGTCTAGAGTTTTTTCCAGAGTTTGGATTTCCGATGGGGTATTTGACTGAGCTACACATTGATGGGACATCCATAAATGAACTTTCTCCCTCAATTAAAAATCTACTTGGCTTGGTTTTATTAAACCTGGGGAATTGTATTAGACTTTCTAGTCTTCCAACTGAAATTGGTAGCTTGAGTTCACTTAAAACTCTCATTCTGAATGGTTGCAAAAACTTGCACAAACTTCCACCAAGTTTGGAGTATGTAAAGCCTCTTGAGGAGCTTGACATTGGGGGAACATCCATAAGCACTATTCCTTTCGTGGAAAATCTAAGAATTTTGAACTGTGAAAGGCTGAAAAGCATTATTTGGCATTCTTTAGCTAGTTTGCCAACAGAATATTTTAGTTCACTCAAAGATTTAAATTTAAGTGATTGTAATCTTGTGGATGAAGACATTCCTAGTGATCTTGAACTCTTTTCCTCGTTGGAAATTCTAGATCTTGGCAGCAATCATTTTGAAAGACTGTCAGAAAGCATTGAACAACTTATTAACCTTAAAGTATTGTACTTGAATGATTGCCACAAACTAAAGCAATTACCCAAGCTTCCACAAAGTATAAGATATGTGGGAGGAGAAAAGTCCTTGGGCATGTTAACAACTTCTCAAGGTAAAGTGCTTTCTTTCTTCTTCATCTTTTTATTTATTTTTCTTTTTCTTGGGTGTTTCCAATTCCACAATATATTATTAATTAGTTTTATTTCATGA

>MELO3C022146

ATTTTTGAAAAACTTCATCATAGAAAATTCATTGATTGGAAGATGAGTTTTGATAGTTTCATAAGTTTTAGAGGCGAAGATACTCGTAATACGTTTACGGGGCATTTGTACAAGGAATTGGTTGGATTAGGAATAACCACTTTTATGGATGATAAGAAACTCTTGATTGGAGATAGTCTTAGTGAAAAACTTATTAAAGCAATCGAAAAATCAGATTCCTTCATCGTTGTTTTATCAGAGAACTATGCTTCTTCAAAGTGGTGTTTGAGAGAATTGGCAAAGATAATAGATTGTACGGATGAACAAAAGCATCGAGTACTCCTTCCTGTATTTTACCACGTCAATCCTCATGATGTTCGTCGTCAATCAGGGTGTTTCGAGAACAGCTTTCGCTTACACGAAGAACTTCTACGAGAACTCGACCATATGGAAAGAGATAAATACATGGAGGAGGTTCAACAATGGAGGAGGGCTTTCACAAAGGTTGGCGATCTCACTGGAGTAGTTGTGACAAAAGATAG

GTGATGTAATTTCAACTACTTTAATCTCTTTTTTGCACAGAAGTTCATTAGTTTTGAACCAACTTCGATAAGTATCTAAGGGAGTGTTCGGCTTTTGGCCATAGGGTTTGAAATTAGGGGGAGAAGCCAAATCCCCTTAATGGAACATTGGAACAAAATTCTTGGCCAAATTAAGAAATTAAATCACACTGAGAGAATATGTTATATATATATATATATATTTATTTATTTATTTATGAATATCATAGGTTTGAGAAAAATTTAACTTCATCCCTGACCTCTTATCAATGGTCGTTGGGAACAATGGTTGGTCGACTTTCTGTTGGAAGCGTTTGATGAACACACCAAATAAATAATCATCAAATAGAATATTTGATAGAAAACAAAAACTTTTTGGAAGACTTATTGTGTGTATTCAACTCACACTTGGATAATTTAGATTACAACATTTCAATCATATTTATAGGATTGTCATGAGAATAACTCATACCACAATGAAATACAACAAATAAAATCTTATGTTTAACTAAAGTGAAACTCTTACTAAATTAACTCTAACATTTTAACTCACATCCTTCTCTAACTTACATAAATTAACTCACATTTATTTAAAACATGTTTAATTTTCAACACTTTCGTGATCAACCATAGGCAATTGATCGTCTTGACCACCAATGATCGAACTACTGACAACTTTTATGTTCTTTTTTTTTTTCTTCTTTCTTCTTCCTTCTTTTCCCTTCAACTTATCATTCCGTTCTTCTTCATTACCCACGAAAAGAAAAATTAATGATTAACTTTTAAATTTTGTGCCTAATAAATTATTTGATGTATTAGAAAAAGTTAATAGGTTGCTAAAATGTACATTTTGATTTGACAAGTACCCTTTGATGTAATATATATTATTTGTTCACACAATGCAG

CGTTGAGGTGGCTAGCATTGGCAAAATCACAAACCAACTACTTGATATGTTATTGCATCATCAAAAGTTAGTACCTTGGGACGAGCTCACTAAGTTAGTTGATATTGAACGTCAGTTATTCAAGATGGAGAAGCTAAATGATTTGGAGCCAAATGTGGTACGTTTTATAGGGATAATAGGGATGGGCGGAATTGGTAAAACAACCATTGCTGAAGTTTTTTATGACAGAGTTGCACGTATTTTTGGAAAAAATCGTTGTTTCCTTCGCATTTATGAACACACTACTTTACTCTCACTCCAACAACAACTTCTTTCCCAACTTCTACAAACAAAGGACCTAATTATAAACAATGAGAATGAAGGAGCAAGAATGATTGGGAGTCGTTTGAAAGATAAAAGAGTTCTAATTGTTCTTGATGGGGTGAAAGAAAAAAGTCAGTTAGAACAGTTAGTTGGAAATCCTAATTGGTTTGGTTCAGGGTCCAAAATCATCATTACAACTAGAAATAGAGATGTTCTTCGCCAACCAAATTATAAAGATAAAATGGTTGAATACAGTATGGAGTTTCTTGATACTAAAAGTGCCATGACACTCTTTTGCAAACATGCATTTGGATGTGGGTTTCCCAGTAAGAACTTCGAGGATTTTTCTAAGGAGATTGTAGAAAGGGTTAAAGGACATCCACAAGCTTTGATACAAATTGGGTCGTCTTTATATGATAAAGGTATAGAGATATGGAAAGAAGAATTGAAGAGTCTTGAGGAAGATTACAACAATCGCATATTTAAGACGTTAAAGATAAGTTTTGATGATTTAGAAAAGACAAGCCAAGAAGTTTTTCTTGATTTGGCATGCTTCTTCAATGAGAAGACGAAAGAGAAAGTGATTGAAATACTTAAGAGTTTTGATTACAGACCTCATAGCGAAATACAATTGTTGCAAGATAGATGTCTCATTGAAGTTAGAAGTGACAACACAATATTTATGCCTAAGTGCATTCAAACTATGGGTCAACAAATTGAACGTGAAGCTGATAAACGGAGTAGGATTTGGATTCCGAAGGATGCCCAGGATGTATTTGATGAACCACAT

GTAAGATTGAAATATAAAAGTTTATATATATACATATAAAAGTTTGTTTCTTTCCCTTTCTTATGTTTTAAATAATGTTTATTTACAG

AGAGTAAAGGACATTAAAGGTGTAGTCTTGAAGTTGGAAGAGAAGCAAGATGAAATAGAGTTGGAGGGTAAGGTTTTTGAAGATATGAGAAGTTTAAAAATATTGGAAATTGGGAATGTGGAGGTGAGTGGAGACTTCACACATCTCTCAAAACAATTGAGATTGCTTAATTGGCATAGCTATCCCTCACAATGTTTGCCATTAAGTTTTGAATCAAGATATTTATTTCAACTTCTTTTGCCTCTAAGTCAAACAAGACAACTTTGGAATGGTCAAAAG

GTTAGTACATACAAATATCATATCATTAATTTTTTTTTTTTTAAAAAATGCAACCCAACTGTGTTTTGTGACAG

GGATTTGAGAAATTGAAGGTTATCAATGTTAGCGGTTCGAAGAATTTACGAGAGACTCCTAATTTTACTAAGGTTCCAAATCTTGAAAGTTTGGATCTAAGTAATTGTACAAGATTGTGGAAGATTGATTCTTCCATTAGTCGTCTCAATCGTTTGACATTGTTGGATATAACATGTTGTATCAATCTCAAAAACTTGTCATTTTCTAGAAGCTGCAAAAGCCTCATAACAATAAACTATGTTGGCTCAGGTCTCGAAGAAAAAGGTACATGTAACTTTCATTATGGTAAATAG

>MELO3C009806

AAGATAAATGACAATCCATTCATTCCATTACGGAGGGAAAGAAGATTCTGTTCATTAAGAACAAACACAAAACCACTTTCACTTGGTTTCCTTTTGTAAAGCAAAAGAGAAAAGGTATGGGAGTTTTTTGTTTATTATGTTTGTTTGCTTGATTACAAAGAAAACGAATACAGTAGCCGAGAAATTGAGCAAAGTAGCAGTTTCTTACTTGATTCCAAATGCAAGGAATTTAGGGCGATTTTAGATAACGATGCATTTTAAGGACGGAAAAACTGACAACGAGAACCAATCTCACAAAATTGAACTCTGACAATGGTTGGACTTCTCGGCAGTGTGGCCGGAAATCTGCTCGGAAGGATAATTGAAGCCGCCGACCGACGAGAGTTTCGTGCTATCCGAAGAGAACTGAAAAACCTCGAAACAGCTGTGTTGGATCTTAAGGCCAGACTCCGAGACGCCGAGGAGAAGCAGGCTAGCGATCCTGAACTCTATGATCAGCTTAGAAAACTCAAACATGCGTTTTCAATGGCAGACAGTGTAATTGAGGAATTGGAATGCGATTATTTGAAGTGGAGAGTGCAGAATCGAAAGAACGATGTTGACGATAAAGGATACCAATTCTCTTCTTGTTTCTCCTCTAATTTCCCCATTTCTTCATGTAATACGGTCGCTAAATTCCAGCAACTCACAGAAGAATTACGTTTGATTGAGGAAACCATGTCTAAATTCTCTCTGGTTGAAGATGAAGATGAATATATCAAAAATTTGAAGGGTGAAATGACTTTGCGGACCTCCATTACTGGTTCGCAAGCTTTCGCTAGGCTTCTGCGCTTGAGGAAAGAGGCGATTCTCTCTAATGTGGATTCCATTTTTGGTAGAGATAAAGTACAAGAGAGTATCATTAAGGAACTTGTGAATGATGAACAAAAATCTCCCCGTATTCTTTCAATCCAAGGAGATGGAGGGATGGGAAAGACGGCTCTGGCCAAGTTAGTCTATAATGCAGACGAAGTGTTTGATCATTTTGACAAGAGAATATGGGTATGCGTTTCTGAAGATTTTGATATTCAGAGAATCATAAAGGAGGTTCTGATTTCTGCAACTGGAGAAAATGTTACCACCGTTGCCTTAACCGAAAGTCGCTTACGAATCCGGCTCCAGCGGTACTTTTTCGGCAAAAAAATCTTGCTTGTTTTGGATGATTTTGGGAATTTGGATCACGATAGAGTATCAGAACTGAAAGAAATCGTGAAGATGGGTGTTGATGGCAGCAAGATAATGATAACCACTCGCAGCCATAAAACTCCAAATGTTGCTGCGACACACAAGATTGACAAACTCGACAAGACGATATCTATGCAAATATTCGAAGATACATTTGGAGGCAACGAGCTTAGCAACGATGTGGATCTCAAAAACCTTGTGGCAGAATGTGGAGGAGCTCCTTTGGCAATCAAATGTTTGGCTGGACTGCTCTCTTCGAAACCGAGCGATGGTGCTAATAGTCCGAATGTCAAGGACTTGAGTGAAAAATGGAAACTGGAGGAGGCGAACTACGGTGGTGGCGTTTCATGTGCACTAAGACTGAGTTATGATCTAATGCCATCTTATTTGAAACCTTTTTTTCTTTGCTTTTCATTGTTGCCGAGAGATAATGTGTTCTTCTCATTTGAGCTAATCCAGTTATGGTTGGAACAAGGATTCCTTCCTTCAGGTACCAAAGATGATCCTGAAGAAATTGGGGAGAAATATTTCAAGGAATTGTGGGATCGCCGTTTACTCGTTGATGTTGAGGAGCACACTCTTGGATATTGGTTCAAAATCCATAACCTTGTACATGATCTTGCAGTCCAAAAGGCTGAGGGACAAAAGAACCTCGGAATTTTTCATCAGCTTTCATTTGTCGATTGCAAGAGCAATATCCCTCCGTCGACAAGCTATAATGACATTCATTTTCTTTCCATTCCAGTTGTAGGTGGTGCGGAACCAAAGATCAATGGAGACCCTCTTTTCAAATGCATCACCAAGTTCAAGCAGCTAAGGTTTTTGTACTTGTGCAACTCTTCTCTGGAAGAAATTCCAACCTCCATAGGCACGCTGAAACATTTGAGGTGTTTAGATTTGCGAGGGAGTCAACGGCTGAAGAGGTTGCCAGAATCAATTTGCAAACTACAGAGCCTACGGACTTTGATTCTTGCCTTCTGCTCAGAGCTTGAAGAGCTTCCCAGAAACATAAAGAACTTGATAAGCCTCAGATTCTTATGGGTCCAAACAAAGCAAGCCAGCTTGGGAAAAGATCAAATAGGAAGCTTAACATCGCTTCGTTTTCTCGCCATTGGAAGGAGTGATAACTTGACTCACTTGTTTGAAGATATCGACAAACTCAATTTCCTCAAAACACTGATCATTTATGATTGCAAATCGCTGCTAACACTGCCAAAAGGCTTGGAAAACGTGGAATCGTTATGTAATATGGGAATATGGGGATGTGAGCGGCTGAGATTTACATTCTCACTGGCTTCACTTAACCTTAAGAAACTGATACTCAGACAACTTACAGCAGTGTCCAGTTTGCCTAAGTGGCTGGCCCATTTGGATGACACTTTAGAAGTGCTAGAAATTGGAGAGTTCCCCACGCTAAGAGAATTGCCTATGTGGTTTTCAAATTATTGGGAACTTCGAATTCTTGGGATCTCCAACTGTCCTCAGTTGAATCACGAATGCTTCCATCATGAACTACCAATTTATCGTGATAAGATTGAGGAGTTGAGGGTCACATTTTGTGGGTCTTCGAGCAAGTCTTCGTGGAAAAAAAGTATGGAGGAAATCAAAAATAAAAACCCGAATATCTCTTACATCCGTGCCATTTATGTGGACTCCAAAAGAATAATGCCACTAGAAGAATCAACAGAAAAACCTAAGGAAGCTGAGACAAAACAGGATGATGCAAATAACAATATGAGTCATGTTGACATTGGATTACTTTCAAAGATAGAACAGGCGCATGCGAATAACAATGTAAGTCATCCTGGGACTAAACAACCTTCAATGAACAAACATGATGATGCAAATAACAATACGAGTCATTCTGGGATTGGACTATTTTCAGAGACAAAACAGGAGCATGCAAATAAAAATGACAATGTAAATGAGAATGAGACCGGTAAGGTCTGTTTGGGAGATAATGACCATGCTGAAGCTGACCAAGCTATGGTCACTACATATGAGGGTTTCTGA

>MELO3C005506

ATGAACGGCGTACTCCAAAATTCCGAGATCCAACCCACCGCGGCCGCTGACCCAAGAACCCAAATCTTCAAATGGGTTACACAAACCGTCGACGGATCCACCGTCCACGGCGTCGAAAACGAGCTTCTAGTTCTACAGAAAATGCTCGAGAAACCAACTATCGGCGGCGGCGGAGACGGCTTCAGAGCTATTGGAATCATCGGAGTACGAGGTATCGGAAAATCAACAATTGCTCGAGCCTTTCTCCAAAAGTCAGAAGTAAAATCCAAGTTCCTCCCCAGAATTTGGATCTCGATGTCGAGAAATTTCACAGAAGACGACGATCCCAAAATCGCTCTTCTGAAGAGAATCCTGATCACTCTCGGAGTTGACACGAAGTTTCCCGGCGGCGAAACACTCGGCAGCCTCCTCTACGCTCTCCGACTTCAACTGAGAGGCAAACGATATCTGATTGTGCTGGATGATGTTCAAGAATTTAAAACAGAGAAAGAACAGAATGATTGGTACTGGGATTTGAATTCTTGTGAGAAGATTGGGGAGAAATTAAGAGATGGGTTTCCTAAAGGGAATGGAGGGGTTGTGATTTTGACGAGTAGAAGTGAGAAAGCTGCAAAAGCTATGGTGGGAGAGGGAAATTTGAGGTGTTTGGTTCCTCAGAAAGACCCAGAAAGTTTCTGGGAAATTTTCCGGCAAGAAGTTGTGAAAGATGGAGTTTCGATCCCTGATGAAATCTTGAATTTCAAGGAATTGAAAGTGAAATTGCTGAAGAAATGTGGTGGACTTCCGTTGATCGCTAAGATGATGGGAGAGATTCAATTC

>MELO3C009694

ATGGATGCGGAAGAGAAGCAAGAACATAGTCGTCATCTACGTAATTGGCTAAAGGAGCTTCAAAATGTATTTTTCCAAATTGAGGGCTTCATAGATGAACTCAAAGAGGAAGTCTATAAAACCGAGGGTATTGGTAAACAGGTACTTGCTCCTTTCTCGTTCTCCATTTGTCAAAGAGCACGTACTCAAAAAATGGAGAAACTATTTGACCATTTAGATGCAGTTGTGGCAAAAATGTATGAATTTGATCTTAAAGAAAGGCACACTGGTGCCATAAAAATGGAGACAACAGACTCTTTCCTTACTGCCACTGAAGTTTCAACAAGTCTCATGAAACCAAGCTGGAAAGTACTTTACCCCTCGACTAGTGCTCCGAAGTCTTATCAGCACGAGCGGTATCGAGAGATTCTGAATGATTTCAAAGAATCTACTTTAGGGTTCTTCCACATAGTTGGAGAAGCAGGTATAGGTAAGAGCACACTTGCCAAATTCATTTACAATGATCCAGAAGTAGAGGAAATGTTTACATCAAGATTGTGGGTTTGTGTGAAAGAGGAATTTGATACGCAGAGATTGATGACAGAGATACTCAACTTTTCATATTCTCCAGCAACTTGTGACAATTTGACTGAGACAAAATCGTGCCCCACAGTTCAAGTTCAACAATATCTGAGAGAGAAAACTTTTCTGCTTGTTTTTCAAGACCTTTCAATCAAGAACCTAGATAATTCTTCCCTGTTTACAAGTTTATTGAGGATGGGAAAGCCTGGTAGCAAAATCATAGTGACCACTCAGAATGAGGAAATTGCAAATGCTATAAAACTACCAAAGGTCATGGTTGAGGTCAACAAGGTTGAGCAACAATCAGAGCAAAATGAGAGTCAGACAGCCCTAGACGCAGTTACTATAGAGACTTCAAATGTTAACAATGCTGGCGAGTCCACTCAAGCTAACCCTCTGGATAAGCTAGATCAATCTATCACATATCAAACAATATTCAAAGTTAAAAGGCTGTCAGAAGAAGATTCCCTTTCTTTATTCAAAGATTATGCTTCTATAGTTGAAGATGACAAAGAAGGTATAATAGAAACTCTGGAGAAATGTAATGGAGTACCATTGGCAATAAAGTGTCTGGGGAGCATGTTATCTTTGGAAACTTCAGCAACTAAATGGATGGAGGACACTGACCAACAAGAGGAAGTTAATGAGTCTTCCAGTACATTTAGTATACTTAAACTATGCTACAATCAGATGCCCTCACACCTGAAGCCTTGCTTTCTTTATTGTTCTCAATTACAAACCGATAGCATACTCTCTTCAAATGATGTCATTCAGTTATGGATGGCAAACGGACTTCTCCATTCACGCCAAGAGAATTACTTATCCTTGGAAGACATAGGTGAGATTTATTTCAAAGAACTATGCTCAAGATGTTTCCTTCAAGATGTTGAAGAATATGGTCTTGGCTATTGGTTTAAAATGCACCCTCTCATTCGAAAACTTGCACGAATACTCATACAAGAACAAACTAAGGACTTGATATGCATTAAACCAGTCACTAAGGTCACATCTATAGCCTTCCCAGTAAGAGATGAGGTACCATCTAGTTCATTTCTAGCTGAAAAATGCATATCAAAGTTCCAATACTTAAGATTATTGCATTTAGGCTACACAGATCTACAGGAAATTCCAAATACTATAGAAACACTGAAGCACCTAAGATACCTAGACTTGCAGGGAAATAAGAAAATCAAGCGGCTACCAAATGCAATCTGTAATCTACAATATTTGCAGACCTTGATTCTTGCATCTTGTTTTGCACTTCAAGAATTGCCAAAAGATATATGGAAATTGAGCAAGCTCAGATACCTGTGGGTAACATCAAACAATCTTCATTTGCACAAAAATGGAGTAGGAACCATGAATTCTCTAAGATTCCTCGCAATTGGAGGATGTGACAAACTTCAAGATCTATTCGAACAGCCATCATGCCTTGTACGCCTTGAAACCCTAATGATTTACAATTGTAAATCTTTGCAGTTGTTGCCAAATGAGATGGGGTCTCTAATCTCATTACAAAATTTGGTGATATGGAGTTGCGAACAACTTACACTGAAGGGTTTAGAGAAAGTCGACTTCAGCCTCCAAAGATTCACAATCAGAGAGCTTCCAAAAGTTAATAAATTGCCTGAATGGCTTCAAAGGTCAACCGAAACTCTAAGAGTCCTGGAAATCATTGATTGTCCCATCAGAGTGGAGGAAGAGGGAATCAAAATGTACAAAGCAGTTGAAAGTAAGATAATTCAAGGAGCTGTAGACGTCACTAGGAATTTGGTATGGCGAAGTCCAATGGTAGCGAAGAACGTACAGAAGATAGGTAACTACTATTAAGTCCTCCATTCAAATGCCTGAGCGATTGAGAAGAAAGAGCGGAATTGTGAACTTCTATAGAGCGGAATTGTGAACTTCTA

>MELO3C015353

ATGCTTCGAGAGAAAATTGATGGAAAAAAATACTTGCTTGTCATGGATGATGTGTGGAATGATAACCGGACGAAATGGATTAGTCTAAAAGCGTTTCTTATGGGTGGAGCTAAGGGAAGTAGGATTTTGATCACAACTCGTACTCATCAAGTTGCACATACTTCTGACACAGTTTTGTCCCATGATTTAAGTGAACTAGACAAGGACAACTCTTGGGAGTTGTTTAGAAAAATGGCATTTTCCAACGAATCGGAGGTGCTTGAAAATTCAAAGTTGGTTTTAATCGGTAAGGAGATTGTGGCAAAGTTGAAAGGTAATCCTCTTGCAATAAGGGTAATTGGGAGCTATCTGTATTCTAAAAGGTCCGAAAAAGATTGGTTGTCATTCAAGGACAACGAACTTGGCAAAATCATACAACAGGAAAATGAGATCCAATCCATACTAAAGATCAGTTTTAACCACCTCTCATCCAGTTTGAAGCAATGTTTCACCTATTGTGCTTTGTTCCTTAAAGATTATAAGATTCAAAAAGATGACTTGATAAAACAATGGATGGCACAAGGCTTCCTTCAACCACAGAACAAGAAGACAATGGAAGATGTTGGTGATGATTATTTCAAAGAACTAATGGGGAGATCATTTTTTCAAGACATAAGAAAAAACAAATGGGGAGAAATCAAGGAGTTCAAGATGCACGACATCATACACGATCTTGCATGTTCCGTGGTAGAAAATGATTGTGTGCTTGCTAATGATGACACTAAGTCCATTGACAAAAGGACTCGACTTGTGTCAATTTCAAAGACAAGATGGGAAGTCGTTAAAGAATCATTAATCAAGGCAAAAAATTTGAGAACATTGAATAATGCTAGTGAGAATTATGTTGGTGGCAAAATCGAAATCGACCTCTCTAATCATCTACGGTTACGAACATTGAATTTGGAGTCTCATTATTATTATTTGGATATTCCCAAGTGTATTGGTAAGATGAAACATTTGAGATATATTAATATTTCTCACTCTGATATTGATTTCCTTCCCAGGGGAGTTACAGAACTATACCATTTGGAAACACTCATCATTCGTGATTGTATGAAGCTAAGAGAACTGCCAAGTGATATTAAGAATCTAATCAATCTTAGGCATCTTGATATTAAGAATCTGATCCATTTTGATGTGCCTTGGTATCGACGTGGTTGGAGTTATATGCCAAAAGGAATGGGTTCAATGACTACCCTTCAAACGATGAATTTGTATGTATTGGGAGAGAATAAAGGCGGTGAGTTAAGTGAACTCAATGGATTGATTAACTTGAGAGGATCATTAAGTATTCGAGAATTGCAGTTTTGCAAACCCATTGGTTTAGAAAATGCTAAATACCTTGAAGAAAAGTCTGGAATTCGAAAGTTGAAATTACATTGTAAGATCTTTGGAAGGAAATTATCTAAAATTGATTATGAAGATGAAAAAGTTTTAGAGTGCTTGAAACCACATCCAAATCTTCAGAAAATATGCATAAAAGGATACAGAGGAGTGAAGTTATGTAATTGGTTCTCATTTGGTAATATAGGTAGTTTGGTCAACATAAAGCTTTGGAATTGTGAAAAATTGCAACATCTCCCTCGATTTGATCAATTTCCTTTTCTCAAACATCTTCATCTCGAAGGTTTACCGAATATTGAGTTTATTGATAATAAAAATTATGTTTCTCATTCATTAACAACTTTCTTTCCCTCCCTTGAGAAACTAAGCATCATTGATTTGCCTAAGTTGAAAGAATGGTGGAAGGGGGAATTCATTGATCAAACTACCTCATTTCCAACGATTTTACATCACCTTTCTGAATTGACTATTTTCAATTGTCCACAGTTGGGTTCTATTCCAAAACATGGACCTTTGCATTCATTGGACATAAGTGATATCAGTTTGCAACTTTTTGAGTTGGTCATGGAAATGGCTACTACAAACATTATTGTCGGATCACAGGATTCTTCTTCTTCAGCTACTACATCATTATCTTCTCTACGTATTTCGAACATGGATTTTGAGTTTGTAGAACTATATGACTTATTCTCCAATATGACACATCTTGAGTTTCTTTACCTATTAAAATGCAAGAATATGAAGATGTCTTCTTCTCTTGATGGAGTGATATGGAAAGGACTTGGAAGTCTTCGTAGACTTATTTTGTGGAGCATCCCTGATTTGGAGTATTTGCCAAAGGGTTTGCAATATGTGACAACTCTTCAATATTTGGAAATAAGTGATTGTCCAAATTTGGTATCTATTGAAGGGATTGAGCATCTCACTTCACTATCAAATTTGCAAATTTATCATTGTCCTAATTTAACTTCATACCCTCAAGAAATGAGTCATCTCACTTCACTAAATTAT

>MELO3C015354

ATGGCAGAATCAATTCTGTTCACCCTTGCAGCAAATATTGCAACCAAGTTGGGTTCTTTCCCACTCCACGAGCTTGGATTGTTGTGGACCGGTTTCCATGAGGAGCTTGATAAACTCAAAGACACTCTTTCCGCCATCCAAGCAGTACTTCTCGACGCAGAAGAGAAGCAGTACAAGAGTTATGCTGTGAAGGAATGGGTTTCAAGGCTCAAAGATGCTTTCTACGATATCGATGATTTGATGGATGAGTTCTCCTATGAATCCATCAAAAGACAGGTTATGATCAAACATAGAACTAACAACAAACAAGTACGTATTTTCTTCTCAGAATCTAATCAAATTGCATTCCGTTTGAAGATGGGTTATAAAATCAAAAGGGTCAGGGAGAAACTCGATATTGTTGCTATTGATAAAGCTCAATTCAATCTTTCTGAGTATACAAGGGAGATACGAAACGACGAAACTACGAAACGACCGGAGACTTCCTCTTTTATACTTGAAGGAGAAGTAATTGGTCGAGATGATGACAAGAAAGGTATTGTACATTTTCTATTGGATACCAACGTCGCACAGGAAAATGTTGCTGTGGTTGCCATTATTGGAATGGGAGGATTAGGAAAGACCGCCCTTGCTCAATCTATCTACGGCGATATGAAGGAAAATAAACATTTTGAATTGACAATGTGGGTGTGTATTTCTGAAGAATTTGATGTCAAAATAATTGTTGAAAAGATTATAGAGTCTCTCACGAAAAAGAGACCTGAGCCCAACCTTCAACTCGATACGTTGCAAAATATGCTTCGAGAGAAAATTGATGGAAAAAGGTACTTGCTTGTCATGGATGATGTGTGGAATGTTAACCGAGAGAAATGGATTAATCTAAAAGCGTTTCTTACTGGTGGAGCTAAAGGAAGTAGGATTTTGATCACAACTCGTACTCATCAAGTTGCACATACTTCTGAAACAGTTTTGTTCCATCATTTAAGTGAACTAAACAAGAACAGCTCTTGGGAGTTGTTTAGGAAAATGGCATTTTCTAACGAATCAGAGGTGCTTGAAAATTCAAAGTTAGTTATAATCGGAAAGGAGATTGTGGCAAATTTGAAAGGTTCTCCTCTTGCGTTAAGGGTAATTGGGAGCTATCTATATTCTAAAAAGACTGAAAAGGATTGGTTGTCATTCAAGGACAATGAACTTGGCACAATCATGCAGCAGGAAAATGAGATTCAATCCATACTAAAGATCAGTTTTAACCAACTCTCATCCGGTTTGAAGCAATGTTTCACCTATTGTGCTTTGTTCCCTAAAGACTATGAGATTCAAAAAGATGATTTGATAAAACAATGGATGGCACAAGGCTTCATTCAACCACAGAATAAGAAGACAATGGAAGATGTCGGTGATGATTATTTCAAAGAATTATTGGGGAGATCATTTTTTCAAGACATAAAGAAAAACAAATGGGGAGAGATCAAGGAGTTCAAGATCCACGACTTCATGCATGATCTTGCATGTTCTGTTGTTGAAAATGTATGTGTGCTTACTAATGATGACACCAAGACCATTGACAAAAGGACTCGACATGTGTCAATTTCGACCTTCATCTCAAAGACAAGATGGAAAGTCATTACAGAATCATTAAAAGAGGCAAAGAATTTGAGAACATTGAATTATGCTTGCGATAAAATCGACCTCTCTAACCATTTGCAGTTACGAACATTGAATGTGGATTTTCTTTATCATGTTCCCAAGTGTATTGGTAAGATGAAACATTTGAGATATGTTAATCTTTCTAATACTTGGATTGATTTCCTTCCGAAGTTTATTACAAAATTATATAATTTAGAAACACTCATCCTTCGTAACTGTCAAAGACTAATGGGACTGCCAAGTGATATTAAGAATTTGATCAATCTTAGGCATCTTGATATTATGCATTGTAACTCAAATTGTTGGAGTTCTATGCCAATGGGATTGGGTTCGATGACTAGCCTTCAGACAATGAATTTGTTTGTATTGGGAGCGAAATGGGGTGGTGAATTAAGCGAACTGAATGGACTTAATAGCTTGAGAGGATCATTAAGAATTAAACAATTGCAATTTTGCATAACTCCTAATTTAGAAAATGCTAAATACCTTGAAGAAAAGTCTGGAATTCAGAAGTTGGAATTACATTGGGATTCTTCTTATGGAATAGGGCGTAATACTTTTAATGACGAAGATGAAAAAGTTTTAGAATGCTTGAAACCACATCCAAATCTTCAAAAAATACGCATAGAAGGATACAAAGGAGTGAAGTTATGTAATTGGTTCTCATTTGGTTCTATAGGTAGTTTGGTCACCATAAAGCTATGCGAGTGTGAAAAATTGAAACATCTCCCTCAATTTGATCAGTTTCCTTTTCTCAAGCACCTTCATCTCGAAGATTTACCGAATATTGAGTTTATTGATGATAACAATTATGTTTCTTCTTCGTTAACAACTTTCTTTCCCTCCCTTGAGAAACTAAGCATCATTCAGTTGCCTAAGTTGAAAGAATGGTGGAAAGGGGAATTCATTGATCAAACTACCCCATTTCCAACGACTTTGCATCACCTTTCTCGATTGAAGATTAATCATTGTCCACAGTTGGGTTCTATTCCACAACATGGACCTTTGAGGTCATTGGACGTAAGTGGTGTTAGTTTGCAACTTTTTGAGTTGGTCATGGAAATGGCTACTACGAACATTATTGTTGGACAAGATTCTTCTTCTTCAACTACTAGATCATTATCTTCTCTAAGTATTTGGGACATGGATTTTGAGTTTTTACAATTACATGACTTATTCTCCAATATGACACATCTTAAGTCTCTTGTCATAGGAAATTGCAAGAATATAAAAATGTCTTCTTCTCGTGATGGTGTGATATGGAAAGAACTTGGAAGCCTTCGTAGACTTGATTTGTGTAGCATCCCTGAATTGGAGTGTTTGCCAAAGGGTTTACAATATGTGACAACTCTCGAATGTTTGAAACTATATGATTGCCGAAATTTGGTATCTATTGAAGGGATTGAGCATCTCACTTCATTATCACTGTTGGAAATTGAATATTGTCCTAATTTAATTTCCTACCCTCAAGAAATGGGTCAACTCACTTCACTATCACGTTTGAGAATCAATGGTTGTCCCAATTTACCTTCCTTGCCAGAAGGGCTTCGCCATGTGACTTTCATGTATTATGAGGACCTACCGACCCCAAGGTATGCACTAAATTTATTTTCTATTAATAATTATAATTTTTGCATCGATCCCAAGGTATTGGAATGTCTTGAATTCTATTTATGTTATTTACAACTTCGGCATAGAGTTTAA

>MELO3C023566

ATGGCCCAAATAATTCTCCCAAATCTTGCTGCGCGGATCTTGGCAAAACTGAGCTCTCTACTTTCTGAAGAATTTGGAACGCTATGGGGACTCAAGGATGATGTGAACAAACTCACATGCACTGTTTCAATTATTCGAGCTACACTTGTTGATGCAGATACGTGGATAACTCACTTAGAATCTGTAGTGGATTGGCTCGAACAGATGGAAGCTGTTCTTAAAGATGCGGAAGATGTGCTTGATGAATTCTCTGCTGAGGCTATCCGTCGAAGAGTGATGACAAGAGGGAGAAATGCAAAACAGGTTAGAATCTTCTTCTCCAACTCTAACCAACTTGTATTTAACTTTAGGATGGCACGTCAAGTCAAGAAAATTAGGGAGAGGCTAGATGCTATTGGTCATCTTAGAAATTATCTTTGGAGTCGAAATTACTTTGATCAAAAAACACAACAATACGATTTATCCGATCAAACGTGGATGGGAAGGGATACTCACTCTTCTATAAGAGGAGAGGAATTAATTGGTAGGGATGATGCTAAGAATTCCCTAAAAGACCTCTTACTAAATCGTGCGAAAGACCACATTTCATTCATTGCAATAGTTGGAATGGGTGGGATAGGCAAGACAACACTAGCTAAGTTTCTCTACAATGATCCAGAGTATCCCCGCATTTTGATATACTAATGTGGGTTTGGGTGTCTGAACAATTTCACGTAAACATACTAGTTGAAAAAATAATAATGTCAGCAACAAAATCCGATAGAAATCCTAACGTACACGTGATGGATAGAAATCCTAATGTACACGCGATGGATTTTTTACAACGTGAACTTAAAAAGGTGATTCAAAATAAGAAGTATTTGTTGGTTATGGATGACGTATGGAACGACCAAAACGAATATGCATGGAGAGAATTGAAAAATTTGTTAATGGATGGAGCTGAAAGAGGGAGTAAGATTATCATCACAAAACGTGATAGTAGAGTAGCTACCGAGATCAAAGATATGACAAAGTCGATCACTTTAGATGTCTTAGATGAGGACAGTTCTTGGTCATTGTTTAAAAAAGTGGCATTTAAGCAAGGCCAAGACTCAAAATATCTAGAAGTGGAACAATTGGGGAAAGAAATTTTAAAAAAATGTGGAGGTGTTCCTCTCGTAATAAGACACATTGGACGCTTGCTATCCTCTAAAACTTCTAAAGAAGAGTGGATGTCCTTCAATGATAACGAAAGTTTGGAAGTCACCCAACAAGACAATCATATGACATCCATATTAGAATTGAGCTACAACAATCTCCCACCCCATTTGAAGCAATGTTTTGCCTATTCATCATTGTTTCCCAAAGGATACAAAATAAAAATAAATGAATTGATTGGACAATGGATTGCTCAAGATTTTATTGAATCATCATCTGGAAGAAAATCTTTGGACAATATAGGGAAGGACTACTTTGACGAATTATGTTCGAGGTTTTTTTATGAAAATTTAAGTGGCGACAACAATTTTGATGATTATGTTCGTATGCACGATGTGATGCGTGAGCTTGCAAAGAAGGTAGCAGGAAATAAATATTATGCTCGTGGAGATTCCAACAATAGTTACGTTGTGAGTGAACAAACTCGCCATATTTCATTTGACTATAAAATTGAATTATGGAGGGATGTTCTTTCTAAATTACTCACGGCTAAAGGACTAAAAACGTTTCTCATATTACATCATCTCTATGAGAAGAAAAATACGGTCGATAGAGAGACTTTGGATAAATTATTTTCCAGTTTTCCACGTCTACGGGTATTAGGTCTTCATGATTCGAAAATCAGTACAGTGCCAGATTCCATAGAAAAGCTTACAGACCTTCGATATCTAGACCTCTCGGAAAATGATATAGAATCACTCCCAAATTCCATCACCAAATTGCAAAATTTGCAAACATTAAAGCTAACAGAATGTCATAAGTTAAAGGAATTGCCGAGGAACACCAAAAAACTCGTAAATCTCAGGCATCTTGCTTTTGATCATTGTATTCAAATAACCCATATGCCAGAAGGGATGGAGAAGTTGACCAGTCTACAAACAATAACTTTATTTGTGTTTGACTGCAAAAAGTCCAATAAGTTATCGGAATTGAATGAGCTCAATTATTCGAAAGCAGAGTTAAAAATCACGGGTTTAGAACAATTGAGGTCTATCGACTTTATCCTACCTGAAGTCGGCTTGGTAAACTTGAAGCACGGTTGGCGAAGTTTAAAACTGGAATGGGAACTTAGTGGGGGTGATGAATATGAAGGTGAGGGAGATAAAACAATAATGGTATGCTTAATACCTCATCCATATCTTGAATCCTTGAGCATTAAAGGGTACTGTGGAGTAGGATTACCCGAATGGGTGTCCGCCTGGCCGTTCTGGAAGTTGACTCAAATTGAAATTGTAAATTGTCATAGATTGCAATATCTGCCTTCAATTGAGCACCTTCATGCACTCAGCAATCTACGTTTACAGAACTTAAGATCTCTCGAGTATATAGATTTTGATTCTTCGAACTCATCATCGTCAGTTTTTTTTCCATCTCTCAAGTTTATATGGTTGCAAGATCTGCCTAATTTGGTAGGATGGGGCCGTGACGATGCATATACATTGTCAGATCAGTTGGCTCCAACTTTTCCTCAGCTTTGTTTCCTGGAAATCTTCGACTGTCCGAAGTTAATTTCCATTCCCAAGCTACCTTCCATTAGAGCATATGTCCATTTATGTCATGTTGGTGTTCCGTTGGTGAAGAGGGTAGGTTTAGTACTGTGGCGTTTGGGGCAGCTAAAACTAGAACAAATTCTGGGTATGGAACTTTTACCGGAGGAATTTTACAAATATTACGTAACTTTTTATGAGGATATCTCGATGTCCCCTGGGCTTCGTTGGTTGGAAATAATTAAATGCCCCAATCTCACGAGTTTACCAACATGGATTAACCGTATCATTTCTCTTGAAACGCTGCGTATTATTAGCTGTCCGAATTTAAAGTCACTACCTGCGGAAATTGGAGAACTCCAATATTTGCTGGAGCTTCAGATAAAGGACTGCCCAGAACTTGAAGAGAGATGCAAGCAGGGAGGAGAGGATTGGCCTAAAATTTCTTCCATCCCCTACATTACTCTCGGCCAAATTGCCACTGTCAATGACACACCATATCTTTCAGGTTTTCTTCAC

>MELO3C023568

ATGGCTGAAGCTATTCTCTACAACTTTACTGCAGACATCATATTCACATTGGGCTCTTCCGCACTCCAAGAGATTGGATCCCTATGGCGTGTCAATTCTGAACTTCACAAACTCAAACACTCTCTTTCTGCAATTCAAGCCGTGCTTCACGATGCAGAGGAGCAACAATCCAAGAACAATCAAGTCAAAGATTGGGTTTTAAAGCTTCAAGATGTTTTGTACGAGATTGATGACTTGATCGACGAGTCCTCTTACCAAACCTTGAGAAGGCAAGTTCTGGCCAAACACCGTAGATACAGAAAACAAGTACGTATCCTCTTCTCCAAATTTAAATCTAATTGGAAAATAGGCCACAAAATCAAGGAAATTAGGCAGAGGCTGCAAGCTATTAATGAAGATAAAAATCAATTTAGCTTTCGTAAGCATGTGATAGAGAGAAGAGATGATGATGAAGGGTTGAGAAAGAGACGGGAGACTCACTCTTTTATACTTGAAGACGAAGTGATTGGTAGGAATGATGACAAGGAAGCAGTCATAGATCTTCTACTAAATTCCAACACCAAAGAGGATATTGCAATAGTTTCCATTGTTGGAATGGGAGGATTGGGAAAGACTGCCCTTGCTCGATCTATTTATAACCATAAGAGGATAATGACTCGATTTTCGTTGAAACTATGGGTGTGTGTTTCTGACGCATTTGATCTCGAAATTATTGTCCAAAAGATAATAGAGTCTGCAACCGGGAAGAAGTCTGGATACCTTCAAATAGATTCATTACAATGTGAGCTTAGAAAGCACATTGATGGAAATAAATATTTGTTTGTCATGGATGATGTGTGGAATGAGAAAAAAGAGGAATGGTTGCATCTAAAAGAATTGCTTATGGCCGGTGCAAAGGGTAGTAGGATTTTGATCACAACACGCAGTGAACAAGTTGCTAAAACTTTTGACTCTACTTTTGTTCATCCATTACAAATTTTGGATGCATCTAATTCTTGGTTGTTGTTTCAAAAGATGACAGGTTTGGAAGAACTTTCAAATAATCAAGAGGCTGAGATTGATCAAACGAATTTAAACTTGATCCAAATTGGCAAGGAGATTTTATCAAGGTTAAAAGGTGTTCCGCTCGTAATAAGAACTATTGGAGGACTTTTAAAAGATAATAAATCAGAAAGATTTTGGTTGTCTTTCAAGGATAAGGAACTTTATCAAGTTTTGAGACAAGGACAAGACGCTCTAAAAGAAATGAAATATATTCTTGAGCTTAGCTATAAATATCTTCCTGCTAACTTGAAGCAATGTTTCTTATATTGTGCTTTGTTTCCCAAAGATTTTGAAGTTCCAAAGATTTCACTTATACTATTATGGAGGTCACAAGGTTATATTCAACCAAATGGCAATAAGGACAATAACCTCGTTGATATCGGTGATGGTTACTTCATGGAGTTATTATCAAGGTCATTTTTTCAAGACGTTAAAAAAGATGATTCTGGAGACATAGTAGCATGTAAGATGCATGATATGATGCATGATCTTGCTTGTTCGTTAACAAATAATGAATGTGTGCATGGACTGAAGGGGAATATCATCAACAAAAGAACTCGTCACTTTGTTTTGGAAGACAAAGATTATCATGAAGATCAACTTATGAGACCATTATCTAAGGCAACACATTTGAGGACATTATTTTATAGAAAAGAAGAAGATGTAATTGGTTCACAATGGAACTTTGAAGAAACCTTCCGTAGTATTTTCCGATTTCGAACGGTGCACTTAGAAGGGTATGATCAAAACCCAAAAGCAAAAGCTTTGGAGTTTATCGGTAAATTTAAACATTTGAGATTTTTGAGTATTACTTATTCAAATATTCCGTATCTTCCAGATTCCATTACGAAGTTGTATTATTTAGAAACATTTTTCTTTCAATGTTCACATAATTTAGAAAATTTACCCAATGATGTGGGAAACTTGATCAACCTTAAGCATTTGGATCTTTCTCATAATTCTACTTTAGAATTCCTTCCCGATTCTATTACTGAGTTGTGTAAGTTGGAAGCACTTATCCTTAAGGGTTGTGAAAAATTAAAGAGATTGCCGAAAAATATAAAAAATTGCAACAATCTTAGGCAACTTGATCTTTCTCACAATGAGAGAATAGAATTTCTTCCTGATTCTATTACTCAATTGATTAATTTGGAAACACTT

>MELO3C023575

ATGGCTGAAGGAATTCTCCTCCAACTTGCTGGGGAGATCTTGACAAAACTGATCTCCTTACTTTCTGAAGAATATGGAATGCTAAGCGGACTCAAGGATGATCTTGACCAACTCAAGAGCACTGTTTCAATGATTCAGAGTACACTTATCGATGCGCAAAACTTGCCAACTGGATTAGACTCTGTAGCAACTTGGCTCGAAAAGTTGAGACACGTGCTTCATGATGCGGAAGATTTGGTTGATGAATTCTGTGCTGAGCTTCGCCATCGAGAAGTTATGACAAGGGAAAAACATGCAAAAAAGGTCAGGATTTTCTTCTCTAGCTCTAATCAACTTGCATTTAACTATAAGATGGCACGTCGAGTAAAAAAAGAATTAGGGAGAGGTTAGATGTTATTGATAAGGAAAAAAGTTATCTTTGGAGTCATAGCTTTGTTCAGGGGAGACAACAAGATGTACCTAGTCCAATTAGAATGAGAATGAGCTTGAGTAGGGAAACTCACTCTTCTTTAAATAAGGAGAGAGTGATTGGAAGAGATGATGATAAGAGGTACTTAAAGAGCCTTTTACTAGTAGATGATATGAGTCTCAAAGACAATGTTTCACTCATTGCAATTGTTGGAATGGGTGGAATAGGGAAGACAACCTTGGCTAAATCTCTCTATAACGATGGGGACGTATCCAAGCATTTCGAGATAAAAATGTGGATTTGGGTTTCTAAGCAGTTCGTTGAAAAAATAGTAGTGAAAAAGATACTAGAGTCAGCAACCAAAATCCCTCCTATTGGACAAGAGTTGGATACTGTACAAAGTCAGCTCCAAGATGTGATTGGAGGTAAGAAGTATTTGTTGGTTATGGATGAAATAATGAATGAAAGTGAAGATGAATGGAAGAAATTGAAAGATTTGTTAGTGGTTGGGAAGAGAGGGAGTAAGATTATAATAACCAAGCGTGATCGTAAAGTTGCATCAGAAATCGAAGGAATGACCACGTCAGTGACTTTAAAAGGCTTGTCTGAGGACCGTTCTTGGTCATTGTTTAGAGAAGTGGCATTTGAAGGAAACCTCCCAAATCCAATAAATCCAAAGTTGGAACTAATGGGGAAAGAAATTTCAAGTAGATGTGGAGGTGTTCCACTTGCAATAAGACATATGGGACGCTTTTTATGCTCAAAAAGATGTGAAGAAGAATGGATGTCTTCTCTGAGTGACAAACTTTTGGAAGTCACCCAACAAAACAATGATGTGGCATCCATATTAGAATTGAGCTATAACGATCTCCCACTACATTTGAAGCAATGTTTTGCCTATTCATCCTTGTTCCCCAAAGTACATAAACTAAAAGTAAATGAGTTGATTAGACAATGGGTTGCTCAAGGTTTTATCGAATCATCAAAGGGAGAGAAATCCATGGAAACTATAGGAAAGAACTACTTTGATGAATTATGTTGGAGGTTTTTTTACGAAAATTCTAGTGATGAGAATAATTTCGATGATTATGTTTATATGCATGATGTGATGTATGAGCTTGCAAGGAAGGTAGCAGACAAGAAATTATATGTATGTGGAGATATCAATAATAATTATGTTGTTAGTGAACAAACTCTGCATTTTTCATTTGATTATAAAATACAATCATGGCAGGACGTTCTGTCCAAATTGTGCAAGGCTAAAGGATTAAGAACATTTATGTTCTTACATTCTCCCTATGAGGAGAAGAATGAGGTCAATGAAGCTAATTTGGATGAACTATTTTCAAGTTTTCCACGTTTGCGAGTATTACTTCTTGGTGGATCCAATATTCATGTACTGCCAAATTCTATAAAGAAGCTTAAACTTCTTCGATATCTCGACCTCTCCGAAAACAACATGAAATCACTGCCAAATTCCATCACTGAATTGCAAGATTTGCAAACTTTAAAGCTGACTGGCTGTTATGCGCTTAAAGAAATGCCAAGGGATATCAACAAGCTTGTAAATCTCAGACATCTTGATTCTCGGTGTTGTTCACAAATAACTCATATGGCGCCGGAAGGGATGGAGAAGTTAAGTTGTCTACAAACAATAAGTTTATTCGTGTTTGATTGCACAAAGACGAATAAGCTACGGGAGTTGAATGAGCTCAATAATTTGACAGGAGAGTTGAAAATCACAGGTTTACAGCAGTTGAGGTCTACTCCATATGAAGTCAGTTTAGTAAACCTCAAAGACAAAAAATGTTTGCGAAGTTTGAAACTGGAATGGAAAATGGGACTGTACAAACACGAAGACGAGGCAGATGAAAGAGTAATGAAAGGCTTAGAACCAAATCCAAATGTTGAATCCTTGGGCATTAAAGGGTACAACGGAGTAGGATTACCCAAGTGGATGTTCAACTGCCATTTGAAGTTAACTGAAATTGAAATTGTAAGTTGCCATAGATTGCAACATCTACCTCAGTTCCATCACCTTCAAACTCTCAAGACTCTATGTCTACGGGACTTAAGATCTCTCAAGTTCATAGATAAGTTTGACAATCCATTTTCATCATCAATGTTTTTTCCATCTCTAAAGTCTCTATGTTTAGCAAATATGGCTAATTTGGAAGGATGGTGGGAATTAAGCCGAGCCGTAGCAGGGGAGACTTCTGAGAACATTCAATGGTTGCCTCCAACTTTTCCTGAGCTTGAGATTCTAGACATCTACCAATGTCCAAAGTTAAATTCTATGCCCAAGCTACCTTCTTCCACTAAAGCATATGTTAGTTTATGTGATGTTGGTGTTCAGTTGGTGATTAGTACAATAGGTCCAGTATTGTTGTGTTTGAGAACTCTAAGACTTGAAGAAATTAAAAATCTGAATTGTCTACCATTTCAGCAAAACATAAATCATTCTTTGGTTATCTCCTCAACAACGACCTCTCCTATTCCCCTCAAATATCTACAAATGGATAAATGCCTCGATCTCGTGACTTTACCAGAATGGATTTACATCTTTACTTCTCTTGAAACGCTGAGTATTTCGAAATGTCCGAAATTAAAATCACTACCAAAGGGAATGCAACGACTCGAATCTTTGAAACGTCTTTGCATAGAGGACTGCCCAAGACTAGAAGAAAGATGCAAGGAGGGAGGAGAGGATTGGCCGAACATTTCACATGTACCCAACATTGTTTTCTTCACGTCACAGTCTTCTTCAGGTCTGTCCC

>MELO3C023576

ATGGGTCAACAAATTAAAGAAGTTAGGGAGAAACTAAATGCTATTGCTGATGATAAAGATAAACTCCACCTTTCCATGCGTATGGGGGAGATACAAGGCGATGAGTTGAGAAAGATACGTGAGACTTCATCTTTTATTCCTGAGGGAGAAGTGATTGGTAGGGATGACGACAAGAAAGTTATTATAGATTTTCTATTGGATACCAAAACCAAAAAAAAGGATAATGTTGAAGTGATTTCCATAGTTGGTATGGGAGGATTAGGAAAGACCGCACTTGCTCAATCTGTCTATAATGATGAGAAGATAAACAAACATTTTCAATTGAAATTATGGGTCTGTATTTCTGAAGAATTTAATGTCAGGACAATTGTTGGAAATATAATAGAGCTTTTCGAGGAAAAGAAACTTGAACCCCTCCAATTGGATAAATTACAAAGTATGCTTTGA

>MELO3C023578

ATGGCGGATTCAATTCTTTTCAATGTTGCTGCTAGTGTTATTACTAAATTGGGATCTTCTGCACTTCGAGAACTTGGATCTCTGTGGGGTGTCAACGATGAGCTCGATAAACTCCAAAACACTCTTTCGGCCATTAAAGCCGTGCTTCTTGATGCAGAGGAGCAACAATCCAAGAGCCACACAGTCAAGGATTGGATTTCAAAGATTAAAGATGTTTTCTATGACATTGATGACTTGATTGACGAGTTCTCTTATGAAACTTTGAGAAGACAAGTTCTTACCAAAGATAGAACAACCACCAAACAAGTACGTATCTTCTTCTCCAAATCTAATCAGATTGCTTTTGGTTTCGAAATGGGTCAAACAATTAAAAAAGTTAGGGAGAAGCTAGATGCTATTGCGGCTGATAAAGCTCAACTTCACCTTTCTGTGAGTGTGAGGGAGGTACGAGATAATGAGCCAAGGAAGGTACGAGAGACTTCCTCATTCATACTCGAGGGGGAAGTCATTGGTAGGGATGAGGATAGGAAATCTATTATGGATTTTCTATTCAATACCAACAACATCATAAAGGATAACGTTGAAGTTGTTTCCATTGTTGGAATGGGAGGATTAGGAAAGACAGCACTTGCTCAAGCTGTCTATAATGATCGAAAAATAAACAATCATTTTAAGTTGAAAATGTGGGTGTGTATTTCTGAAGAATTTGATATCAAAGTAATTGTTGAAAAAATTTTAGAGTCTATTACGAAAACAAAACAAGAGTCCCTTCAGTTGGATATATTACAAAGTATGCTTCAAGAGAAAATTGATGGGAAAAAATACTTGTTGGTCATGGATGATGTGTGGAATGGAGACCACGAGAAATGGATTGGTCTGAAAAGATTAATGGGTGGTGCTAGAGGAAGTAAGATTTTGGTGACAACCCGTAATCTACAAGTTGCACAGGCTTCTGACACTGTTTGGTTCCATCACTTAAAAGAACTTGACAAGGACAACTCTTGGGCGTTGTTTAGGAAAATGGCATTCTTAAACAAAGAAGAAGAGCTTGAGAATTCAAATTTGGTTAGAATGGGTAAAGAGATTGTAGGAAAGTTGAAAGGTTATCCCCTTGCAATAAGAGTAGTTGGACGTTTGTTATATTTCAAAAACACAGAAATGGATTGGTCGTCATTCAAGGACAACGAA

>MELO3C023579

ATGGCCCTTGAAGGAATTCTTTCCGGTGTTGGAGTGGAAATCTTGAAGAAGCTTAGCTCTCAAGCTTCAGCATACCTTGGAAGGCGATGTGGTGTTAAGGATGATCTTGACAAACTAAGGAGCAATGTTAAATCCATTCAAGCTGTACTTCGTGTTGCAGAGCAACTCCAAGGCAATGATCATTCTTTAACCGATTGGCTCGAAAAGTTGGGAGACGTCTTTTACGATGTTGAGGACGTGCTTGATGAAATCTCTACTGAGGCTCTCCGTCGAGAAGTGATGACGAGAGGAAAAAATGCAAAGCAGGTTAGAATCTTCTTCTCCAATTCTAACCAACTTGCATTTAGCTATAGGATGGCATGTCAAGTCAAGAAAATTAATGAGAGGCTAGATGTTATTTCCCAAGAAAAAGAAAAGTTTCATTTCAATAATCCAGTTGGGATACAAAATGTTTTATCTTATCCAAAAGGAATGCAAAGGAATTCTCAGTCATCTTTAAGAGTGGATCAGAAAATATTTGGAAGGGTTGACGATATGAACAACCTTAAAAAAAAAAAATTACTAGCGGAGGATGACAAGGTGAAAGCTAACGTTTCATTCATTGCTATTGTTGGAATGGGTGGAATTGGCAAGACAACCTTGGCCAAATCTCTCTACAATGACAAACAAGTCTCTGATTGTTTTGAGACAAGAATTTGGATTTGGGTTTCTCATCAATTCGACACAAAAACAATATTGGGAAAGATAATTGAATCGGCAACCGTAAAGAAACCAAAGGTAGATGAATTGGAACCTTTAAATACAAAGCTTCAAGAAGTGATTGGAGGAAAGAAGTATTTGTTAGTTATGGATGATGTATGGAATGAAAACGAAAATGAATGGGAGAATTTGAAAAGCTCGTTAATGCATGGTGCAAGAGGGAGTAAGGTTTTGATCACAAAGCGTGACAGTAAAGCAATTTCAAGAATTGAAACAATTCCTCTAAAAGACTTAACTGAGGATTCTTCTTGGTTGTTGTTTAAAGAAATGGCATTTGAAGAAAGCGACTTAGAGTCAACAAATCAAAACTTGATAAAATTGGGTAAAGAAATTTCAAAAAAATGTGGAGGTATTCCTCTTGTAATAAGACATATAGGACGCTTATTAAATGGAAAAACTTCCGTGGAATATTGGGAGTTCATCAAAGAAAATGACCTTTTAAATGTCACTCGTGAGGAGAACAATAATGATGGTCATGTGATATCAACATTAAAATTGAGCTATAACCATTTGTCACCAAATTTGAAGCAATGTTTTGCCTACTCATCCTTGTTTCCCAAAGAATACAGAATTACACCAACTGAATTGATTGGACAATGGATAGCTCAAGGTTTTATCGAATCATCAAATGAAGGAAAATCTGTAGAGGATATTGGGAAGGAGTACTTAAATGAATTATGTTGGAGGTTTTTCTTTGAAATAACTTCTACTTCTAGAGAGTTTTCTTTTGAAGATAATGAATTATGTTGCATGCATGATGTGATGCGTGATCTTGCAAGGGAGGTAGCTGGAAAGAAATTGTACGTACGTGGAGATCCAAATAATGAATATGTTGTGAGTGAACAAACTCGTCATATTTCATTTGAATATGTAATAGTATCATGGAAGGATGTTTTATCCAAATTGCACCAGGCCAAAGGATTAAGAACGTTTCTTTCGCTTGCGGAGAGTCCAATAACAAATGGAGTTTTGGACAGACTAGTTTCCAACTTTCCACGTTTACGAGTATTACAAATCTTTGATGTGTCGAAGTCTATAAAAAAGCTTAGACATCTTCGATATCTAAAGTTTACTCGTATGGATGCTGGGAAATCACTTCCAAACTGCATCACGGAATTGCAAACACTAGATCTAACCGATTGTTTTTCCCCAGTGTATCTGCCAAGGGATATTAAAAATCTTGTAAATCTCAGGTATCTTCTTAGTTATTCAAAATATATCGATGCGGTGGAAATAATGGAGAAGTTGACTAGTCTACAAACAATACGTTACGTTTTGCTTGATTGTAAAAAATTTGATAAGGTAAAGAAATTCAGTAAGATGAATTGTTCTATAGAATCTAGTTTACAAATCATAGGTTTGGAGCAGTTGAGGTTTTTTACATCTAGAGTCAAATTAGTAAACCTTAAAAACAAAAGAGTCCCACATGTGAAACTGGAATTTAAGAATGATAATACATATGACGGCGATGATGATGAAACAATATTGGAAGGCTTTGAACCACATCGAGATGTTAAATGCTTGGATATTGAAGGGTATTGTGGAGTAGGATTACCCAATTGGGTGTCCACCTTACATTTGTTAACTGAAGTCTTTATCAAAAATTGTGATAGATTGCAACATCTGAATCAGCTCTCTCATCTTCAAGCTCTTAAAATGTTACATTTATGGGGCTTAAAATGTGTCATGATGAGTATATCAGAATGGATTGTCACCCTTACATCTCTTGAAGTTATGGATATCCGTAATTGTTGGAAATTAAAATCACTTCCAAAGGAAATGCAACAACTCAAATGTTTGAGGAAACTTCGCATATATGGGTGCCCAGAACTTAAGGAGAGATGCAAGGAGGGAGGACAGGATTGGCCTAACATTTCCCATATTCCCGAATTGTATTTACATTTCTGA

>MELO3C027066

TGGGTCAACAAATTAAAGAAGTTAGGGAGAAACTAAATGCTATTGCTGATGATAAAGATAAACTCCACCTTTCCATGCGTATGGGGGAGATACAAGGCGATGAGTTGAGAAAGATACGAGAGACTTCATCTTTTATTCCTGAGGGAGAAGTGATTGGTAGGGATGACGACAAGAAAGTTATTATAGATTTTCTATTGGATACCAAAACCAAAAAGGATAACGTTGAAGTGATTTCCATAGTTGGTATGGGAGGATTAGGAAAGACCGCACTTGCTCAATCTGTCTATAATGATGAGAAGATAAACAAACATTTTCAATTGAAATTATGGGTCTGTATTTCTGAAGAATTTAATGTCAGAACAATTGTTGGAAATATAATAGAGTTATTCGAGGAAAAGAAACTTGAGCCCCTCCAATTGGATAAATTACAAAGTATGCTTCGAGAGAAAATTAATGGAAAAAGGTACTTGTTGGTCATGGATGATGTGTGGAATGAAAGCCATGAGAAGTGGATTGATCTAAAAAGATATCTAATGGGTGGTGCAATGGGAAGTAGAATTTTGATCACAACACGTAGCCAACAAGTTGCACAGACATCTGACACAGTTTCATTTCATCATTTAAAAGAACTCGACAATCACAACTCTTGGGTGTTGTTTAGAAAAATGGCATTTTTAAACGAAGAAGAAGAGATCGAGAATTCAAATTTGGTCAAAATCGGTAAGGAGATTGTAGCAAAGTTGAAAGGTTCTCCTCTTGGAATAAGAGTAGTTGGCCGTTTGCTATATTTCAAAAACACGGAAAAGGATTGGTTGTCATTCAAGGACAATAATGAACTTGGCACAACTTTACAACAAGAAAATCAGATTCAACCAATACTGAAGATTAGTTTTGACCACCTTCCATCTAACTTAAAGCAATGTTTTATGTATTGTGCTTTGTTTCCTAAAGATTATGAGTTTTGGAAGGATGAATTGGTAAAACTATGGATGGCACAAGGTTTCATTCAACCACATGGTAAGAAGGCAATTGAAGATGTTGGGGATGATTATTTTAAAGAGTTAGCGGGGAGGTCGTTCTTTCAAGACATAAGAAAAAATAAATGGGGAGACATCAAGAAGTGTAAGATGCATGATTTGATACATGATCTTGCGTGTTCGATGGTAGAAAATGAATGTGTGGTTGTAAGTGATGGTGTTGGGTCTATTGACAAAAGGACTCGACATGTCTCATTTTTCTATCGCCGAAGGCAAATGTCGAGGGAACTAGTACCTAGATTGTTCACTAAGGCAAAGAAGTTCAGAACATTGAGTTTGAATTCAGCTTCTATTTCTTTTCTGAAAACAATGTATCGCATTAATCTTTTTCGATTACGAACATTGAATTTGAAATACTGTTGTCATCCTCCTAAATTTATTGATAAGTTGAAACATTTGAGATATCTTAATCTTTCTCACTTGCGTATAGATTTCCTTCCAAAGTTTATTACCAAATTGTATAATTTGGAAACACTTATCCTTCGCTACTGCAAATGGCTAAGAGAATTGCCAAAAGATATTAGCAATTTGATGAACCTTAGGTATCTTGATCTACATGGATGCTTCCGTTTGACTCGCATGCCAAAAGGGCTAGGTGAGATGAGTAGCCTTCAGACAATGAATTTGTTTGTATTAGGAAAAGATAAAGGTGGCAATTTAAGTGAATTGAATGAACTTAAAAGCTTGAGAGGATCGTTATGTATTCGAGGATTACAATTTTGCACAACTATTGATATAGAAAATGTGAAATATTTTGAAGAAAAGTCTGAAATTCGAAAGTTGAAATTACATTGGGACACAGACAAGATGAAGCCAAAAATTGATGATGCCTCATATGCTGAAGATGAGAGGATTTTGGAGTGCTTAAAACCACATTCAAATGTTTGCAAAATGAGTATAAAAGGATATAGAGGTATAAAGTTATGTGATTGGGTGTCTCCTGATTATTTCCTGGGTGGTCTGGTTAGCATAGAGCTTTGTCATTGTGAAAAATTGGAGCATCTCCCTCAATTTGATCAATTTCCATGTCTCAAGAATCTTGATCTTGAGGACTTATCCAATATCGAATACATTGATGATAGCAATTCTGTTTCTTCATCAACAACTTTTTTCCATCTCTTGAGAAACTAA

>MELO3C027140

ATGCGGTCAAGGTTTGTTGGTTTTTCAAGGCTTAAGGATGTTTTGTACGAGATTGATGACTTGATCGACGAGTCCTCTTACCAAACCTTGAGAAGGCAAGTTCTGGCCAAACACCGACGAAACAGAAAACAAGTACGTATTATCTCCAAATTTAAATCTAAATGGAAAATAGGCCACAAAATCAAGGAAATTAGGCAGAGGCTGAAAGCTATCAACGAAGATAAAAATCAATTTAGCTTTTGTAAGAATGTGATAGAGAGAAGAGATGATGATGAAGGGTTCAGAAATAGACGGGAGTCTCACTCTTTTATACTTGAAGACGAAGTGATTGGTAGGAATGATGATAAGGAAGCAGTCATATATCTTTTACTAAATTCCAACACCAAAGAGGATATTGCTATAGTTTCCATTGTTGGAATGGGAGGATTGGGAAAGACTGCCCTTGCTCAGTCTATTTATACCCATTACGGTATGACTAATGATGGCATGTTTCAGATGAAGTTATTGGTGTGTGTTTCTGAAGAATTTGATCTGAAAATTATTATCCAAAAGCTAATAGAATCTGCAACCGGGAAGAAGCCTGAATCATTCCTTCAAATAGATTCATTACAATGTGAGCTTAGAAAGCAAATTGATGGAAAGAAATATTTGCTCGTCATGGATGATGTGTGGAATGAGAAAAAAGAGGAATGGTTACATCTGAAAAGATTGTTGATGGGTGGTGCAAAGGGTAGTAGGATTTTGATCACAACACGCAGTGAACAAGTTGCTAAAACTTTTGACTCTACTGCCACCTATTCCTTACAAACTTTGGATCCATCCAATTCTTGGTTATTGTTTCAGAAGATGACAGGTTTGGAAGGACGTTCAAATAATAAAAAGACCAAGCTTGATCAAATGAATTCAAACTTGATGCAAATCGGCAAGGAGATTTTGTCAAG

>MELO3C006780

ATGGCTGATTTTGTCTGGACATTTGCACTGCAAGAGATTCTCAAGAAGACATTGCACCTTGCAACCCAGCAAATCCGTCTGGCCTGGGGTTTCAAGCAGGACCTCTCTAAACTTCTCGACTCACTACTCTTCTTCGAAGCCATTCTTCGCGATGTCGATCGAACAAAATCTGACCGAGAATCGATCAATATTTGGGTGACTAAGCTTCAGGATTTAGTGCTCGATGCCGAAGTTGTACTGGACGAGCTCTCCTACGAGAACCTTAGGCGAGAAATGGACGTCAATGGAAATTCTAAGGAAAGGGTACGTGATTTCTTTTCGTTATCGAATCCCTTGATGTTTAGGTTGAAAATGGCGCGTAAAATTAGAACCATCACCCAAGTTTTGAATGAGATTAAAGGCGAGGCTAGTGCTGTTGGGGTTATTCATAAAGGGGGGAATAATGAAATAGTGGCTGATAATGGCCAAATTCCAGAGACTGACTCATTTCTTGATGAATTCGAAGTTGTAGGAAGAAGGGCTGATATATCCAGAATAGTGAACATTGTTGTTGATAATGCCACTCATGAAAGGATTACTGTGATTCCTATTGTGGGAATGGGTGGTCTTGGAAAGACCACTTTGGCAAAAGCAGTCTTCAACCATGAGCCTGTGAAAGCGCATTTTGATGAAACTATCTGGGTGTGTGTGACTGCAACTTTTGATGAAAAGAAGATTTTAAGAGCAATTTTGGAATCTCTAACAAATTTTCCAAGTGGTTTGGATAGTCAGGATGCTATACTTAGAAGGCTACAAAAGGAGCTGGAAGGGAAAAGGTACTTTCTTGTGCTGGATGATGTGTGGAATGAAAATGTTAAACTGTGGAACAATTTCAAGAGTCTTCTGCTAAAGATTACAAATAGTATTGGGAATAGAGTTCTTGTGACAACTAGAAGTGAGGAAGCTGGAAAAATCATGGAAACATTTCCCAGTTATCATTTAGAAAAGCTATCAGATGATGAATGCTGGTCAATATTCAAGGAAAGAGCATCAGCAAATGGATTACCACTGACCCCAGAATTGGAAGTTATTAAGAATGTGCTTGCAGAGCAGTTTGGAGGCATTCCATTGGTTGCGAAAGTTCTGGGAGGGGCTGTACAATTTAAGAAAAGAACAGAGACTTGGTTGATGTCAACATTGGAAACCCTTATAATGAATCCACTTCAAAATGAAAATGACGTTTCATCTATTTTGAGATTAAGCGTGGATCATCTGCCAAACTCATCATTGAAACAATGCTTTGCCTACTTTTCTAATTTTCCCAAGGGTTTTAACTTTGAAAAGGAACAACTAATCCAATTTTGGATGGCAGAAGGGTTCATTCAACCTTCTGATAAAGTAAGCCCTGAAACCATGGAAGATATAGGAGATAAATACTTCAATATCTTGCTGGCCCGTTCCTTATTTCAAGATATTGTCAAAGATGAGAATGGTAAAATTACACACTGTAAGATGCATCATCTTCTACATGATCTTGCTTATTCTGTTTCAAAACGTGAAGCATTGGGTTCTAATCTTAATGGTCTAGTTGATGATGTTCCTCAAATTCGACAATTATCCCTGGTTGGCTGCGAGCAAAATGTAACGTTGCCTCCTAGAAGGAGCATGGAGAAGTTGCGTTCTCTATTTTTGGATAGAGATGTGTTTGGCCACAAGATTTTAGGTTTCAAGCGCTTGCGTGTTCTGAACATGTCCCAATGTGAAATACATAACTTACCAACTTCAATCGGAAGGTTAAAGCATCTAAGGTATATTGATGTCTCAAATAATATGATAAAGAAACTTCCAAAATCTATTGTTAAGCTTTATAAATTGCAGACCCTGAGGCTGGGTTGTTTTCGGGGAGAAGCCCCCAAAAAATTCATAAAATTGATCAGCTTGAGACATTTCTATATGAATGTTAAAAGACCAACAACTAGGCACATGCCTTCGTATTTAGGCAGGTTGGTTGATCTTCAATCCTTGCCTTTTTTTGTTGTTGGGACAAAGAAGGGTTTCCATATAGAAGAGCTTGGATACTTGAGGAATCTCAGAGGTAAATTAAAGCTTTACAATCTTGAATTAGTAAGAAATAAGGAGGAAGCCATGAGGGCAGATTTGGTGAAAAAGGATAGGGTGTACAAATTGAAACTGGTATGGAGTGAAAAAAGAGAAAATAATAATAACCATGACATTTCTGTTTTAGAAGGACTTCAACCACACAACAATCTTCAATACTTGACAGTTAAAGACTTTATGGGAGAACTTTTTCCAAATCTTACTTTTGTTGAAAATTTGGTACAAATTTCTCTAAAAAATTGTAGCAGATGTCGAAGAATTCCAACATTTGGACATTTACCTAATCTTAAGGTTCTTGAGATTTCTGGATTGCACAACCTAAAATGTATAGGAACGGAATTTTATGGAAACGAATATGAAGAAGGAAGTTTGTTTCCAAAATTGAAAAGATTTCATCTTTTGGACATGAAGAATCTTGGACGTTGGGAAGAAGCAGCAGTGCCAACAGAAGTTGCAGTTTTTCCTTGTCTTGAAGAGTTGAAAATTTTCGACTGTCCTAGACTAGAAATTGCACCTGATTACTTCTCGGCTCTTAGGACATTAGAAATTGATGATGTCAACAACCCAATTTCACAGATCACTCTTCAGACATTTAAACTACTTGGTATTATACACTCTGGCAACCTGAGTGGTTTGCCTGAGGAGTTACGTGGTAATCTGTCATCTCTTGAGGAGTTTAAGGTTTGGTATTATCTTCACTTGAAAACTTTTCCAACTATTGAGTGGCTCACTGATATTTTGAAATGCAAGATCGGATATGACACAAAGTGGACAAATATTCAATCTCATGGGCTAGAATCGTACACTTCTGTGAATGAATTGTCCATTGTTGGGCACTCTGATCTCACATCAACCCCAGATATAAAAGCTTTATGTAATCTTTCGTCTTTAACAATTAGTGGCTTGAAGAAATTGCCAAAAGGATTTCACTGCCTCACTTGCTTGAAAAGTTTGTCAATTGGTGGATTCATGGAGGGGTTTGATTTTAGGGCTCTTTTGCATCTCAAGTCTCTTGAAAATCTTGCAATGATAGATTTTGGTAGTGCAGAAAGCACTCTTCCTGATGAGCTTCAACACCTAACTGGCTTAAAGCACTTGAAAATTGTTGGATTTCAGGGCATTGAATCTCTGCCAGAGTGGTTAGGAAATCTTAACTCATTGGTAAGTTTGCATATAGAGAGTTGCAGAAAATTGAGAGAGCTTCCAGAAGCCATGGGTTGCCTCGCCAAATTGGAGGAACTGCGGAGTTTTAATTGCCAAGAGTTGAGGGTTTACCAAGACGAATCAGAATGGGCCAAAATTTCTTACATTCCAAGATTCATATCATTCAATTATTGGGTTGATGAGTAA

>MELO3C017700

ATGGCTGAATTCCTATGGACATTTGCTGTGGAAGAGACGTTGAAGAGAACGGTGAAAGTTGCTGCTCAGAAAATTGCTGTGGTTTGGGGTTTGGAAGATGAGCTTTCTAATCTAAGCAAATGGCTACTTGATGCTGGAGCCCTTTTGCGCGATATCGATAGGGAAATACTTCGCAAGGAATCGGTGAAGAGATGGGTAGATGGGCTTGAAGATATCGTTAGTGAAGCTGAGGATCTTTTGGATGAGCTAGCTTATGAAGATCTTCGAAGAAAGGTGGAAACAACTTCAAGGGTGTGTAATAATTTCAAATTTTCTTCTGTTCTTAACCCTCTTGTTCGTCATGATATGGCCTGTAAAGTGAAGACAATTACTAAAATGTTAAAACAACATTATCGTAAATCTGCTCCTTTAGGGCTTGTTGGGAAGGAATCCATGGAGAAAGAAGATGGAGGTAATAATCTTAGGCAGATTAGGGAAACAACTTCGATTCTGAATTTTGATGTTGTGGGGAGGGAAGCTGAAGTTTTAGACATATTGAGATTGGTGATTGATTCTAGTAGTAATGAGTCTGAGCTTCCTTTGTTGATTGTACCGATTGTAGGGATGGGTGGAGTTGGAAAAACAACTTTGGCGAAATTGGTTTTTCGTCATGAGTTGATCAAGAAACATTTTCATGAAACAATATGGGTATGTGTGTCGGAAAACTTCAACATCGACGAGATTTTGGTAGCAATTTTGGAAAGTTTGTCGGATAAAGTTCCAACCAGAAGGGAAGCTTTACTTCGAAGGCTTCAAAAAGAGTTGCAAGACAAAAGATGTTTTCTTGTTTTGGATGATGTTTGGAATGAAAGTTCTAAGTTGTGGGAAGAGTTAGAAGACTGTTTGAAAGAGATAGTTGGGAAATTTGGAATCACCATTATGGTAACTACAAGGTTGGATGAAGTTGCTAATATTATGGGAACAGTTTCCGGTTATCGTTTGGAAAAGTTACCTGAAGACCATTGTTGGTCCTTATTTAAGAGAAGTGCAAATGCAAATGGAATAAAAATGACTCCAAAGTTGGAGGCTATTCGAATAAAGTTACTTCAAAAAATTGATGGCATACCGCTAGTTGCAAAAGTTTTGGGAGGAGCCGTGGAGTTTGAAGGAGATCTTGATAGGTGGGAGACCACACTTGAAAGTGTAGTAAGAGAAATTCCAATGAAACAAAAAAGTTACGTGTTATCCATATTACAATTAAGTGTGGATCGTCTACCCTTTGTGGAAAAACAATGTTTCGCCTATTGTTCAATTTTCCCTAAAGATTGTGAAGTTGTTAAAGAAAGTTTGATTAGAATGTGGATAGCACAAGGGTTTATTCAACCACCAGAAGGAGAGAACATGATGATGGAGGATCTGGGAGAAGGGTACTTCAACTTCCTCTTATCTCGCTCCTTATTTCAAGATGTCGTCAAGGATAAGTATGGGAGCATTACTCACTTTAAGATGCATGATCTAATACATGATGTTGCCCTTGCCATTTTGTCAAATCGTCAAAAGATGGTATTAGATCCTACTCATTGGAATGGGAAAACAGCAAGAAAGTTGCGCACCTTAATTTACAATAACCAAGAGATCCACCATAAACTTGCAGACTGCGTTTTCTTGCGTGTTTTAGAAGTGAATTCCTTACGTATGATGAATAACTTACCAGACTTCATTGCTAAGTTGAAACACTTGAGATACCTTGACATTTCATCATGTCCTATGTGGGCTATTCCCCACTCTATTACTACGCTTTTCAATTTACAGACACTGAAGCTTGGAAGTATAGAAAATCTTCCAATGAATTTGAGAAATTTGGTTAGACTACGTCACTTAGAATTCCACGTCTACTACAATACAAGGAAAATGCCTCCTCATATGGGTGAGTTGATTCATCTTCAAACATTGTCCGGGTTTGTTGCAGGGTTTGAGGAAGGTTGTAAAATTGAAGAACTCGGAAATTTGAAAAATTTGAGGGGTAAATTGCAACTTTCAAATCTTGAGCAAGTAAGGAGTAAACAAGAAGCTATAGCTGTGAAATTGGTCGATAAAAAAAACTTACGTGAGCTAACTTTTGAATGGAGTATTGATCTTTTACGAGAATTTAGCAGCTACAATGACTTCGAAGTGTTGGAAGGACTTCAACCACCTAAAAATCTCACTTCTTTGAAAATTACCAACTTTGGAGGGAAATTTTTGCCTGCTGGTACTTTTGTTGAAAATTTGGCGTTCCTATGTTTGTATGGTTGTACAAAATGTGAAAGGCTTCCAATGCTTGGACAATTACCCAACTTGCAAGAACTTGGTATTTGTTTCTTGGATCGTGTGATAAGTATAGGGAATGAGTTTTATGGCAATGACTCCAACCGAAGGGGTTATTTTCCCAAGTTGAAGAAGTTCGACTTAGGATGGATGTGCAACCTAGAGCAATGGGAATTAGAAGTGGCAAATCATGAATCAAATCATTTTGGTTCTCTTCAAACTCTAAAGTTGGATAGATGTGGCAAATTGACAAAACTGCCAAATGGGTTAGAATGTTGCAAATCTGTTCGTGAGGTGATAATATCAAACTGTCCTAACCTTACCTTAAATGTGGAGAAAATGCATAGCCTGTCTGTTTTATTAATAGATGGGTTGAAATTTTTGCCAAAAGGATTAGCTCACCTCCCTAACTTGAAGACCATGATGATCACAGGATGCATAGAGGATTATGATTATAGCCCTTTCCTAAACTTGCCTTCTCTTACAAAACTTTACTTGAACGATGGCCTTGGAAATGCCACCCAACTTCCTCAACAACTTCAGCATCTCACTGCCTTAAAGATTTTAGCCATTGAAAATTTTTATGGCATTGAAGTTCTTCCTGAATGGTTGAGAAAGCTTACATGTTTGGAGACTTTGGATCTTGTTCGTTGCAAAAATTTGAAACGGTTGCCTTCAAGAGAAGCCATGCTATGCCTCACCAAATTAAAGGATTTCAAAGTTATGGCATGTCCCTTGTTGCTACTTGGGGGCCAAGCTGACCAAGAAGGTGCCAAATATCTTCATATTCCAGCCTACCTTTGTCATGTATATCAATCTAGAGGA

>MELO3C017701

ATGGCGGATTTCCTATGGAGCTTTGCTGTAGATGAAGTGTTAAAGAAGACAGTGAAGCTTGTGGCAGAGCAGATTGGGATGTCATGGGGGTTTAAGAAGGATCTTTCAAAACTAAGGGACTCTTTACTCATGGTTGAAGCCATCCTACGTGATGTTAACAGAATCAAGGCAGAACATCAAGCCTTGAAGCTATGGGTGGAGAAGCTTGAGCATATCGTTTTTGAAGCCGATGTTTTACTCGACGAGCTCTCTTATGAAGATCTTCGACGCAAGGTGGAAACCAGACCGGTACGTAGCTTCGTTTCATCCTCCAAAAATCCTCTTGTTTTTCGCCTCAAAATGGCCAATAAAATTAAAGCTATTGCTAAAAGGTTAGACGAGCATTATTATGCGGCGAGTATCATGGGGCTTGTTGCTATAACATCCAAAGAAGTCGAGTCTAAACCTAGCCAAATTCTAGAGACAGACTCGTTTCTTGATGAGATTGGAGTTATAGGGAGGGAAGCTGAAGTATTAGAGATAGTGAATAAACTACTTGACCTTAGCAAACAAGAAGCAGCTCTATCTGTTTTATCTATTGTTGGTGTAGGTGGATTAGGAAAAACATCTTTGGCGAAGGCGATATTTCATCATGAAATGATAAGGGAGAATTTTGATAGACTGATATGGGTGTGTGTTTCTGAACCTTTTGTTATCAACAAGATTTTAAGAGCAATTTTGGAAACTCTTGATGCTAATTTTGGTGGCTTAGACAATAAGGAAGCTTTACTTCAAGAGCTTCAGAAATTGTCGAGGAACAAAAAGTATTTTCTCGTACTTGACGATGTTTGGAATGAAAATCCTGATCTGTGGAATGAGTTAAGGGCTTGTTTGCTAAAGGCCAACAAAAAATTTGGAAGTGTTATTGTTGTGACAACTAGGAGTGATGAAGTTGCAAATATTGTGGAGACAAATCATCAAAGACATCATTTGAGAAAGTTATCAAATGATTGTTGTTGGACTTTATTTGAAAAATGTGCATTTGGAAGTGATTTGCCAGTGACTCCAAGAGTTGATCATGTAGTTAGAGAAGAGCTTGTTAAAAAATTTGGTGGCATACCTTTGGTTTTGAAAGTGTTTGGAGGAATGGTGAAATTAGACGGGAATGATTATTGTGAAGGATTGCAATCAACTTTGAAAAATTTAATCGTAAGTCCATTACAAGATGAAAATCGTATTTTATCTACCATAAAATTAAGTGTAGACCGACTGCCATCATCTTCATTGAAGCAATGTTTTGCCTTTTGTTCAAGCTTTCCACGAGGCTTCTTATTTGTAAGAGAACAACTTGTTCAAATGTGGATAGCTCAAGGGTTTATTCATGTACCCAGTGGGAGCAATGTAACGATGGAGGATATTGGGGCAAACTACTTCAATACTTTATTCTCGGTCCTTGTTTCAAGATGTCGTCAAAGATGA

>MELO3C017703

ATGGCGGAATTCCTTTGGACTTTCGCAGCTCAAGAACTGTTGAAGAAGACAGTGAAGCTCGCAGCAGAACAGATCGGCCTGGCATGGGGTTTCAACAATGAGCTCTCAAACCTCAGAGACTCCCTACTTATGGTGGAAGCCATTCTTCGTGATGTCGACAGAATTAAGGCAGAGCATCAAGCTGTGAAGCTATGGGTAGAGAAGCTTGAAGCTATTGTTTTCGAAGTCGATGTTCTACTCGATGAGCTCGCTTACGAAGATCTTCGCCGCAAGGTTGAATCCCAAAAAGAGGCGATAGTAAGTAATTTCATTTCTTTCTCCAAAACCCCTCTTGTTTTTCGTCTCAAGATGGCCAATAAAATCAAGAACATTGCTAAGATGTTGGAAAAACATTATTCTGCTGCTAGTACTGTGGGGCTTGTTGCTATATTATCTAAACAAACTGAACCTGATTTTAGCCAAATTCAAGAGACAGATTCGTTTCTTGATGAGCATGGAGTTATTGGAAGAGAAACTGAAGTTTTGGAGATCGTGAATGTATCTGTCGATCTTAGCTATAGGGAGGGGTTGTCTGTTTTGCCAATTGTTGGTATGGGGGGATTAGGAAAAACAGCTTTGGCTAAGGTAATATTCAATCATGAATTGATAAAGGGGAATTTTGATAGAACTGTATGGGTGTGTGTTTCAGAACCTTTTCTTATCAAGAAGATCCTAAGAGCAATTTTGGAAACTCTTAATTCTAATTTTGGTGGCTTAGATAGTAAAGAGGCCTTACTTCAAGAGCTACAGAAGTTGTTGAATGACAAAAAGTATTTTCTAGTTCTTGATGATGTTTGGAATGAGAATCCTATCCTTTGGAATGAGTTGAAAGGTTGTTTGTTAAAGATTAGCCAAAGATCTGGAAATGTTGTTGTTGTGACTACTAGGAGTGACAGAGTTGCTGAAATCATGGAGACACATTCTAGATATCACTTGACGAAACTATCTGATGACCATTGTTGGTCTTTATTCAAGAAATATGCATTTGGAAATGAATTGCTAGGAATTCCTGAATTGGATATTGTTCAGAAAGAGCTCGTTAAAAGATTTGGTGGCATACCATTGGCTGTAAAAGTGATGGGAGGAATTGTTAAATTTGACGAGAATCATGAGGGATTGCAGAAATCTTTGGAGAATCTAATGAGACTTCAATTGCAAGATGAAAACCATGTTGTATCCACGATAAAGTTAACTGTAGATCGCCTACCATTGTCATCGTTGAAACAATGTTTTGCTTACTGTTCCAATTTTCCAAAAGACTTCAAGTTCAGAAGAGAAGCCCTTGTCCAGATGTGGATAGCACAAGGATTTATTCAACCCTCTTTGGGAAGTGATGAAATGATGGAGGATATTGGTGAGAAGTACTTCAATGTTTTGTTATCTCGCTTCTTGTTTCAAGATATTGTCAAGGATAATAGAGGGAGAATTATATTCTGTAAGATGCATGATCTTATACATGATGTTGCATGTGCTATTTCAAATTCTCAAGGATTGAAATGGGATCCTTCAGATTTGTTGGATGGAGAACTAAAAACACCAGATTGTAATGAAAATCATTCTAGAAAGTTGCACATGTTGACATTTGATAGTCATGTGTTTCACAATAAGGTCACAGACTTTATCCACTTGCGGGTTTTAATTGCACATTCGTGGTTTATATGTAAGTTACCAAATTCAATTGCTAAGTTGAAGCATTTGAGGTATCTTGACATTTCATATTCTACCATAAGGGAGCTACCAGATTCCGTTGTTCTGCTTTATAATTTGCAAACATTGAAGCTTTCAAGATTTTTTAACGACCTTCCAAAAAATTTGAGGAAGTTGGTTAGTTTAAGACATTTAGAATTTTTCTCTGATCCTTGTAATACTAAACAAATGCCTCAACATTTGGGTAAATTGATTCAACTCCAAACGTTGTCTGGTTTTGTAGTTGGGTTTGATGATGGATGTAAGATAGAAGAGCTCAGATCTTTGAGAAATCTTAAAGGTAATTTAAACCTTTTATGTCTTGAGCGAGTGAAAAGTAAAATGGAAGCCATGGCTGCAAATTTGGTGGAGAAGGGGAATATTTCATTTCTGTATTTTTATTGGACTTTGAGAAGTGAAAGATCGGAAGGAAGCAACTACAATGATTTGAACGTGTTAGAAGGACTTCAACCGCATAAAAATCTTCAAGCTTTGCGAATTCGAAACTTTTTAGGTAAACTTCTGCCAAATGTTATTTTTGTTGAAAATTTAGTCGAGATATATCTACACGAATGTGAAATCTGTGAAACTTTACCAACACTCGGGCAGTTATCAAAGCTTGAAGTACTTGAACTTCGTTGTTTATGTAGTGTAAGAAGTATTGGAGAAGAATTTTATGGGAATTACCGTGAGAAGAGGATTTTATTCCCGACATTGAAAACATTTCATATCTGTGAAATGATCAATCTAGAGAATTGGGAAGAAATAATGGTTGTATCAAATGGCACAATCTTTTCCAATCTTGAAAGCTTGAACATTGTTTGTTGTCCAAGATTGACGAGCATTCCAAACCTTTTTGCATATCATCATGAGAGTTCATTTCCAAGCTTTCAACTTTCGGCAAAGCTTCGATCTCTAAAGATTTTGGGATGTGAAAGTTTGCAAAAACAACCGAATGGCTTAGAATTCTGCAGCTCCCTTGAAAACATGTGGATAAGCAACTGTTCTAACTTGAATTACCCTCCAAGCTTGCGAAATATGCAGAATTTAACTTCTTTAAGCATAACCGAGTTTCGAAAGCTGCCAGACGGTAATTAG

>MELO3C021831

ATGGGTGATTTCCTATGGACTTTTGCTGTGGAAGAAATGTTGAAGAAGGTGTTGAAGGTTGCACGGGAGCAAGCTGGCCTAGCATGGGGCTTCCAGAAACATCTCTCCAAGCTCCAAAAATGGCTACTCAAGGCTGAAGCTTTCTTACGCAATATCAACACGAGAAAACTACATCATGATTCTGTGAGGATGTGGGTGGATGACCTTCGGCATCTTGTTTATCAAGCCGATGATCTATTAGACGAAATTGTTTATGAACATCTTCGACAAAAGGTCCAAACAAGAAAAATGAAGAAGGTGTGTGATTTTTTTTCTCCTTCTACCAATGTTTTGATCTTTCGTCTTAACATGGCAAAAAAAATGATGACTCTTATAGCATTGTTAGAAAAGCATTACCTTGAGGCTGCTCCTTTAGGACTTGTGGGGAATGAAAACGTAAGACCAGAGATCGATGTTATTAGTCAATATCGAGAGACAATTTCAGAACTCGAAGATCATAAGATTGTGGGGAGGGATGTTGAAGTTGAAAGTATAGTGAAACAAGTGATTGATGCTAGCAATAATCAACTTACATCTATCCTACCCATTGTTGGTATGGGTGGATTAGGAAAAACAACTTTGGCAAAGTTAGTTTTCAACCATGAGTTGGTTAGACAACATTTTGATAAAACTGTATGGGTATGCGTCTCTGAACCATTTATTGTCAACAAGATTTTGTTAGATATTTTACAAAATCTAAAAGCCGCCTGGCATTTCTAATGGAGGGGATAGTAAGGAGGTTTTACTTCGTGAACTCCAAAAGGAGATGCTTGGGCAAACATATTTTCTTGTGCTTGACGATGTTTGGAACGAAAATTCTTTTCTATGGGGTGAGTTGAAATATTGTTTGCTCAAGATCACTGGAAACTCTAAAAATAGTATTGTTGTGACTACAAGGAGTGCTGAAGTTGCAAAAATCATGGGAACATGTCCCGGTCATCTTTTAAGTAAATTATCTGATGATCATTGTTGGTCCTTGTTTAAAGAAAGTGCAAATGTATATGGACTATCAATGACTTCAAACTTGGGAATTATTCAAAAAGAGTTGGTCAAAAAAATTGGTGGCGTACCATTGGTTGCACAAGTTTTGGGAAGGACAGTAAAGTTTGAAGGAGATGTTGAGAAATGGGAGGAAACGTTGAAAAGTGTGCTAAGAATTCCGGTGCAAGAGGAAGATTTTGTTTTATCTATATTAAAATTAAGTGTGGATCGTCTACCATCATCTGCATTAAAACAATGTTTTTCATATTGTTCAATTTTTCCCAAGGATTTTGTGTTTGAAAAACAAGAACTAATTCAAATGTGGATGGCACAAGGTTTTCTTCAACCACAAGAAGGAAGAAACATGACAATGGAAACTGTAGGAGACATATACTTCAAGATCTTGTTGTCACACTGCTTATTTCAAGATGCCCATGAAACAAGGACAGAAGAATATAAGATGCATGATCTTGTATATGGAACAAGGACAGAAGAATATAAGATGCATGATCTTGTACATGATATTGCAATGGCAATTTCAAGGGATCAAAATTTGCAACTAAATCCTAGCAATATATCGAAGAAGGAACTTCAAAAGAAGGAGATTAAAAATGTTGCATGCAAGCTACGCACGATTGATTTTAATCAAAAGATTCCTCACAATATAGGTCAACTGATATTCTTTGATGTGAAGATAAGAAACTTTGTTTGTTTGCGTATTTTAAAGATATCGAAGATGTCTAGTGAGAAGTTACCGAAGTCAATTGATCAATTGAAACACTTGAGATATCTAGAAATTGCAAGTTATTCAACGAGATTAAAATTTCCAGAGTCTATTGTTTCGCTTCATAATTTGCAAACATTAAAGTTCCTATACTCATTTGTTGAAGAATTTCCAATGAACTTTTCAAATTTGGTAAGTTTAAGGCACTTGAAATTATGGGGAAATGTTGAACAAACGCCTCCACATTTAAGTCAATTGACTCAACTCCAAACATTGTCTCATTTTGTAATTGGGTTTGAAGAAGGTCGTAAGATTATTGAATTGGGACCATTGAAAAACTTGCAAGATAGTTTGAATCTTTTGTGTTTGGAGAAAGTTGAAAGTAAAGAGGAAGCCAAAGGAGCAAACTTGGCAGAAAAGGAGAATTTAAAAGAGCTAAACTTAAGTTGGTCCATGAAAAGAAAAGATAACGATAGTTACAATGATTTGGAAGTGTTGGAAGGACTTCAACCAAACCAAAATCTCCAAATATTAAGAATCCACGACTTTACAGAAAGGCGTTTGCCTAACAAGATTTTTGTTGAGAATTTAATAGAGATAGGTTTATATGGTTGTGATAATTGTGAAAAGCTTCCAATGCTTGGACAGCTAAACAACTTAAAGAAACTTGAGATTTGCAGCTTCGATGGCGTTCAAATTATAGACAACGAGTTCTATGGTAATGATCCAAACCAAAGAAGGTTCTTCCCAAAGCTTGAGAAATTTGCAATGGGTGGTATGATGAACTTAGAGCAATGGGAAGAGGTAATGACAAATGATGCATCATCAAATGTTACAATTTTTCCCAATCTTAGAAGCTTGGAGATAAGGGGATGTCCCAAATTAACAAAAATTCCAAACGGATTACACTTTTGTAGTTCCATTCGACGAGTGAAAATATACAAATGTTCAAATTTGAGCATAAATATGAGAAATAAGCTGGAATTATGGTATTTACACATTGGTCCGTTAGACAAGCTACCAGAAGATTTATGTCATCTCATGAATTTGGGGGTAATGACAATTGTTGGAAATATACAGAATTATGATTTTGGCATCCTTCAGCACCTTCCTTCCCTTAAAAAAATTACTTTAGTCGAGGGTAAGTTGAGCAATAATAGTGTAAAACAAATTCCTCAACAACTTCAACACCTCACTTCCTTGGAATTTCTGTCAATTGAAAATTTTGGAGGCATCGAAGCTTTGCCAGAATGGCTAGGAAACTTGGTATGTTTGCAAACACTCTGTTTTCTTTGTTGCAGAAATTTGAAAAAACTACCTTCTACAGAAGCAATGCTACGTCTCACTAAATTAAATAAATTGTATGCTTGCGAATGTCCAATGCTACTACTCGAAGAAGGTGACCCAGAGCGAGCAAAACTTTCCCACTTTCCAAACGTGTTGGCTCACCGCAACACGTTCGAGAGTTGTAGGTTTTTT

>MELO3C024725

ATGGGTGATTTCCTATGGACTTTTGCTGTGGAAGAAATGTTGAAGAAGGTGTTGAAGGTTGCACAGGAGCAAACTGGCCTAGCATGGGGCTTCCTTAGAAACATCTCTCCAAGCTCCAAAAATGGCTACTCAAGGCTGAAGCTTTCTTACGCGATATCAACACGAGAAAATTGCATCATGATTCTGTGAGGATGTGGGTAGACGATCTTCGACATCTTGTTTATCAAGCCGATGATCTATTAGACGAAATTGTTTATGAACATCTTCGACAAAAGGTCCATACAAGAAAAATGAAGAAGGTATGCGATTTCTTTTCTCCTTCTAGCAATGCTTTCATCTTTCGTCGTAACATGGCGAACAAAATGATGACTCTTGTAGAATTGTTAGAAAAGCATTACAATGAGGCTGCTCCTTTAGGACTAGTGGTGAATGAAAATGCAAGACCAGAGATTGATGTTATTAGTCAATATCGAGAGACAATTTCAGAACTCGAAGATCATAAGATTGTGGGGAGGGATGTTGAAGTTGAAAGTATAGTGAAACAAGTGATTGATGCTAGCAATAATCAACTTACATCTATCCTACCCATTGTTGGTATGGGTGGATTAGGAAAAACAACTTTGGCAAAGTTAGTTTTCAACCATGAGTTGGTTAGACAACATTTTGATAAAACTGTATGGGTATGTGTCTCTGAACCATTTATTGTCAACAAGATTTTGTTAGATATTTTACAAAATCTACAAGGTGGCATTTCTAATGGAGGGGATAGTAAGGAGGTTTTACTTCGGGAACTCCAAAAAGAGATGCTTGGCCAAACATATTTTCTTGTGCTTGACGATGTTTGGAATGAAAATTCTTTTCTATGGGATGAGTTGAAATATTGTTTGCTTAAGATCACTGGAAACTCTAAGAATAGTATTGTTGTGACTACAAGGAGTGTTGAAGTTGCAAAAATCATGGGAACATGTTCTGGTCATCTTTTAAGTAAATTATCTGATGATTATTGTTGGTCCTTGTTTAAAGAAAGTGCGAATGCATATGGACTATCAATGACTTCAAACTTGGGGATCATCCAAAAAGAGTTGGTCAAAAAAATCGGTGGCGTACCATTGGTTGCACGAGTTTTGGGAAACGCAGTGAAATTTGAAGGAGATGTTGAGAGATGGAAGGAAATGTTGAAAAGTGTGCAAAGAACTCCAGTGGAAGGGGAAAATTTTGTTTTGTCTATATTAAAATTAAGTGTGGATCGTCTACCATCATCTACATTAAAGCAATGTTTTTCATATTGTTCAATTTTTCCCAAGGATTTTGTATTTAAAAAACAAGAACTAGTTCAAATGTGGATGGCACAAGGTTTTCTTCAACCACAGGAACGAAAAAACTTGACAATGGAAAATGTAGGAGACATATACTTCAAGATCTTGTTGTCACATTGCTTATTTGAAGATGCCAATGAAACAAAGACAGAAGAATATTATAAGATACATGATCTTTTACATGATATTGCAATGACAATTTCAAGGGATCAAAATTTGCAACTAGATCCTAGAAATATATTGGAGAAGGAACTTCAGAAGAAGGAGATTCAAAATGTTGTATGCGGGTTACGCACAATTGATTTCATTCAAAAGATTCCTCACAATATAGATCAGACACTTTTTGATGTTAGTATAAGGAACTTTGTTTGTTTGCGTGTTTTGAAGATATCTAGTGATAAGTTACTGCAGTCAATTGGTCAATTGAAACACTTGAGATATCTAGAAATTTCTAGTTATCCTCCAATGATATTAAAATTTCCAGAGTCTATTGTTTCACTTCATAACTTGCAAACATTAAAGTTCTATTTATCAATGATTAAAGAATTTCCGACCAACTTCACAAATTTAGTAAATTTAAGACACTTGAAATTCTATCTGATTGGTTGCAAAACGCCTCCATATTTAAGTCAATTGACTCAACTTCAGACGTTGTCTCATTTTGCAGTCGGATTTGAAAATGGTTGTAAGATTACTGAATTGGGTTCATTGAAAAACTTGCAAGGTAGTTTGAGTCTTTCGTGTTTGGAGAAAGTTGAAAATAAAGAGGAAGCCAATGGAGCAAACTTGGCAGAAAAAGAGAATTTAAAAGAGCTACACTTAAATTGGGACATGGAAAGAAAAGATAACAATAGTTACAATGATTTGGAAGTGTTGGAAGGACTTGAACCAAGCAAAAATCTTCGATCATTAAAAATCCACTACTTTGCAGGAAGACGTTTGCCTAACCATATTTTTGTTGAGAATTTAAGAGAGGTAAATTTGCGTGGTTGTAATAATTGTGAAAATCTTCCAATGCTTGGACAATTAAACAACCTAAAGAAACTTGAGATTTACAACTTCCAAGAACTCCAAATTATAGACAACGAGTTCTATGGTAATGATCTAAACCAAAGAAGGTTCTTCCCAAAGCTTGAGAAATTTGTAATGTGGGATATGATCAACTTACAGCAATGGGAAGAAGTAATGACAAATGATGCATCATCAAATATTACAATTTTTCCCAATCTTAGAAGCTTGGAGATATGTAGATGTCCCAAATTATTAAACATTCCAGAAGTTTTTGATGAGAATAATGTGCAACACCTTGAATCATTGATCGTTTCACACTGTAACAAATTAACAAAACTTCCAAATGGACTACACTTTTGTAGTTCCATTCAACGTGTGAAAATAGACCAATGTTCAAATTTGAGCATAAATATCAGAAATAAGTCGGAATTGTGTTATTTAAACATTGGTCCGTTGGAGAAGCTGCCAGAAGATTTATGTCATCTCATGAAGTTGAGGGGAATGAAAATTGTTGGAAATATGCAGAATTATGATTTTGGCATCCTTCAGCACCTTCCTTCCCTTAAACAAATTAATTTAGTCGAGGATGAGTTGAGCAATAACAGTGTAACACAAATTCCTCAACAACTTCAACACCTCACTGCCTTGGAATTTCTGTCCATCGAAAATTTTGGAGGCATTGAAGCTTTGCCAGAATGGTTAGGAAATTTTGTGTGTTTGCAAACACTTAGTCTTTTTAATTGCAAAAATTTGAAAAAACTGCCTTCTACAGAAGCAATGCTATGTCTCACCAAATTAAATCGATTGAATGCCTGTCGATGTCCACAGCTACTACTCGGCAATGGTGACATGGAGGGGGCGACACTTTCCCACCTTCCACAAATTTCGATTTATCGCGATAGTTATGTGAGTTCAATTTAG

>MELO3C024732

ATGGCGAAAAAAATGATGACTCTTGTAGAATTGTTAGAAAAGCATTACAATGAGGCTGCTCCTTTAGGACTAGTGGTGAATGAAAATGCAAGACCAGAGATCGATGTTATTAGTCAATATCGAGAGACAATTTCAAAACTCGAAGATCATAAGATTGTGGGGAGGGATGTTGAAGTTGAAAGTATAGTGAAACACGTGATTGATGCTAGCAATAATCAATTTACATCTATCCTACCCATTGTTGGTATGGGTGGATTAGGAAAAACAACTTTGGCAAAGTTAGTTTTCAACCATGAGTTGGTTAGACAACATTTTGATAAAACTGTATGGGTATGTGTCTCTGAACCATTTATTGTCAACAAGATTTTGTTAGATATTTTACAAAATCTAAAAGGAAGCACTTCTAATGGAGGGGATAGTAAGGAGGTTTTACTTCGTGAACTCCAAAAAGAGATGCTTGGCCAAACATATTTTCTTGTGTTTGACGATGTTTGGAACTAA

>MELO3C025519

TTTAAACACAAAAATCCTTGGAAAAAATTGAACAATATGAAAAACATTGCTTTACTGCAGATGATGGTAGACGATCCGCACTTAATTTTAATATAGACAAAAAAAAGTTTTCCTCTTGTATGGGAGTTATTAGCACACTTTTCAACATTTCCTCCCATCTCTCAACATCTCCTTCAAATTTTACTGCCCTTCCCAAAACTCGTGCAACCAATGATACACCACCAATTTTTTTGACCAACTTTTTTTTAATGATCCACAAGTTTGAAGTCATCGATAGTCCATATGCATTTGCACTTTCTTTAAACAAAGACCAACATTGATCACCAGATAATTTACTTAAAAGATGACCATGACACATTCCCATGATTTTTGCAACTTTGGCACTCCTTGTAGTCACAACAATACTATTCTTAGAGTTTCTAGAGATCTTGAGCAAACAATATTTCAACTCCTCCCATAGAAAACAATTTTTGTTCCAAACATCATCAAGCACAAGAAAATATCTTTGCCCATGCATCTCTTTTTGGAGTTCACGAAGTAAAACCTCCTTACTATCCCCTCCATTAGAAATGCCGCCTTTTAAATTGTGTAAAATATCTAGCAAAATCTTGTTGACAATAAATGGTTCGGACACAGAAACCCATACAGTTTTATCAAAATGTTCTCTAACCAACTCATGGTTGAAAACTAACTTTGCCAAAGTTGTTTTTCCTAATAAACCCATACCAACAATGGGTAGGATTGATGTAACTTGATTTCTAGCATCAATCACTTGTTTCACTATACTTTCAACTTCAACATTCCTCCCCACAATCTTATGATTATCGAGTTCCGAAATTGTGTGTCT

>MELO3C007539

ATGGCGCTGGAATTGGTGGGTGGAGCTGTTTTGGGGACTGTGGTTGGGGAGCTATTCAAAGCGGTCTCGAATCTGGGTGAAAGGGCCATTAGTTTCAATCCTGTTCTTAAGGATATCCGTTCCAAGCTTAATGCTATATTCCCTTTGGTGAAGCAAATCGATGAGCTTAATGATTATCTCGATTACCCAAAGGAAGAGACAGAGAAATTGAGGGATCTAATGGATGAAGGGAGGCGGCTGCTTCTCCAGTGCGGCGATGTGAAATTGGGGGATCTTAATTATTTGAAGAAGCCATTTTACACCAAAAGGCTTCGGGAATTGGATACTGCACTTCGAAGTTTCATGGATATTTTGATGTTGCAGATGGCTAGAGATCAGAAGAAGAACATGAAGATGATGAACCAAATGATGGAGATCATTTGTAGACTTGATAATAGAGGTGGGTCGAGTAAGCCTATGGATTTGTTTGTTCCACCATGTCTGGTTCCTCAACTGCGAGAAGAAACGGTTGGGTTGGAGAAGCCAGTTAAGGAGTTGAAGGTGAAACTTTTCAAAAATGGGGTTCAAATGTTGGTGGTGACAGCTCCTGGTGGCTGTGGAAAAACCACACTGGCCTTAAAATTTTGCCATGACAAAGAAGTCAAAG

GTACGTCCAAATCATTCCTTTTGAGGAAAAAATGAAAAAAGTTTGCCTATATATTGAGCTTAGGGAATGAAGTTATATTAAACTATTGGATGATAAGAACTTAACGGCATCTCTTACAGGAGCTTTCTAATTTCTCGTCTTGATAG

ATATATTCCAGGAGAAGATCTTCTTTGTTGCAGTTTCAAGGAAACCAGATTTGAAGCTTATATTGAAAGATATAATTGAAAGCCTTAGAGGCATTCAATTGCCTGATTTGCAAAGTGATGAACGTGCATTCTGCTATTTAGAAATGTGGTTGAAGCAAACAAGTGTAAATCGTCCTGTTTTGATTGTGTTAGATGATGTGTGGAATGGACGAGAATCTGAAGTTCTTCTTGATAATCTGTTCCAATTGCCTTGCTGCAAGGTCTTGGTCACTTCTAGGTTTTATTTCCCAAGATTTAGTGAGTCTCATTATTTGGAACCTTTGAACCGTGAGAATGCAATACAACTTTTTCGTCGTGCAGCATCATTGGACAAAGGAATTTCTAAGCTCCCCGATGATGAAACT

GTAGAAAAGGCAAACTTCCTATTATCTGATTAGCTTTACTTTTTATAATATTTTTATATCTGTCAGTTTCTTTTAAATGGCTAAGTTTAAAGATCTCTTTGAAATTGAATTTCAG

ATAATTGGGGGATGCAAGAGACTCCCTCTTGCACTGAAGGTAATCGGAAGGTCTCTTTCCCGCAAACCAACATCGGTTTGGAAAGTGACAGGGAGGAATTTGGCTAGAAGTGGCTCCATATTTGATTCTGACAATGAACTTCTTGAATGCCTACAGAGCAGTTTGGATGTCTTGGATGATAACATGGTAACTAAGCAGAGTTTCATGGATTTAGGCTCTTTTCATGAAGATCAAAGAATTCCTGCTTCGACCTTCATTGACATGTGCACAGTTCTGTACAAACTAGACGAAAGTGAAGCAATGGTTATCCTCGACGAACTATCCTCTCGAAGTCTAGTTAATTATGTCACGACGAG

GTAAATACTATTCACAAATGTTTGTAATTTCACTTGCCGATAGTGTGACCTGTATTATTGATGTAAAGAGAATAGAAACTGATTTATTTTGTGGGGTTATTGATCAG

AAAATATGGATATGATGATGACTTCTATGAAGAGTACTCTTTTACTCAGCATGATATTCTTAGAGATTTGGCTATCCACTTGATGAATATGGAGCCCATAGAACAAAGGAAAAGATTGATCTTAGACATTAATGAAAACGATCTTCCCAAATGGTGGGTTGATCAAGAAAAGCATCCTTCCTATGCCAACCTTATATCTATAACCACAG

GTTTCTCTCTCTCTCTCTCTCTCTCTCTCTCTCTCTCTCTCTCTTCTTTCTTTCTTTCTTCTTCTTCTTACTTCTACTACTNNNNNNNNNNNNNNNNNNNNTATCTATAACCACAGTTCTCTCTCTCTCTCTCTCTCTCTCTCTCTCTGATATTCCATTTTCTTTCCTTTTTCTTTATCACTTTCACAACTTACTCAAAGGGGTTTTTGATGTTGCAG

ATGAGAGATTCTCAGCAAGTTGGCCTGACATGGAAGCACCTGAAGTGGAGGTTCTGATTCTTAATCTTCAGTCAAGAACTTACAACTTGCCTGGGTTCATCAAAAGAATGAATAAGCTGAAAGTTTTGGTAATCACATATTTTGGTTCTTTTCTAACTGAGGTGACAAGTGAAGATAATCAACTACTCGACTGCCTAACGAGTCTTGAACGAATTAGGTTCGAGCGGTTTTCAGTTTCTATCTTTAGTAATCCAAACCCGAAACCGCTGATAAATTTGCAGAAAATATCCTTCTTTATGTGCAAATTTGGTCAAACATTCATGGACGTTTCAACCCCAATCTCAGATTTGTTGCCAAACTTGCTGGAGATTTCCGTAGACTTTTGCAACAATTTGAGTTCAGTCCCCAATAGGTTGTGTGAAATTGTCAGCTTGCAGAAGCTGAGCATTACAAATTGCCATGGACTATCTTCCTTGCCAGAAGATGTAGGAAAGTTGATTAATCTAAAAAATCTAAGGCTAAGATCTTGCATTCATTTAGAAGAGTTTCCCGAGTCGACAACGAAGCTTCGGGAATTAGTCCTGCTTGATATATCTAACTGTCTTGGTCTTGCCAAGCTTCCTGAGAAGATTGGTGAATTGCATAATTTAGAAAAGCTTGACATGAGGCACTGCTCAAGTTTGCGCAAGCTGCCATTGTCGATTGGAAACCTGAAAAAGGTGAAGTTTTTATGTGATAGAGAGGTTGGAGAGTGGTTGAAGAAGGTTGCGCCTCGCCTTGCCAAACAGGTGAAAGTGCAAGAGGAAGAAGCCAACCTGGAGTGGCTCGGTTTTTGA

>MELO3C016725

ATGGCGGTTACAGATTTCTTTGTTGGAGAGATAGCCACTGAGCTTCTCAGAATGGTGGTACAACTTTCGACCAAATCCTGCCTTTGTAAAACGACGGCAGCTCAAATTGCCAATTCTATTCAACAAATTCTGCCGATTATCGAAGAGATCAAGTACTCGGGAGTTGAATTACCCGCTCATCGTCAATTTCAGTTAGACCGCTTCAGCGAGACTCTTAGAAGAGGCATCGAGATTTCCGAGAAGGCTCTTCAATGTGGCCGATTAAACATTTACAGAAACTTACGGCTCGCGAGGAAGATGGAGAAGCTCGAAAAGGATATATGTCGATTCATTAGTGGCACCATGCAGGCGCATATACTGGCCGATGTGCATCATATGAGATTCGAGACCACCGAGCGGTTTGACCGGCTTGAAGGTGTTTGGTTGGAGAGGCGGCTTGAGTCAATGAAGATTAGAGCAGATGCTTCGGGAGAGGAAAGGTGGTGGGTTGAGGAGGCGTTGAAGAAGGCCGAGGAGGAGGAAAGGTATGAGAGTAATTTGGTGAATATAGGAACTGGATTGCGTGTGGGGAAGAGAAAATTGAAGGAGCTGATGATTGGAAGGGAGGATTTAACGGCGGTTGGGATTAGTGGAATTGGGGGTTCGGGGAAGACCACTTTAGCTAGAGAATTCTGCAAAGATCCGGAAGTTCGAA

GTGAGTTGCTTTTCTTTGGCTATTTTCTCTTATTTTATGCGAATGATTTCGATTTTCGGTTCAAAACTTAGTAGTTTTTGTACTGAATCTATATGGGTTTCTGACTTTGAAGCAAAGTAGAGCAGAAAATTTTCACTTGAATTGGAAATTTCTTGTTAGTAAAGTGAATAGTGTTTAGAGTATCCATTTGATACCGTAGCTCTTAAAAGGGTAAATAGGAAATCCTTTTTTTGTGTTTCTTTTTGTAGGAATTGTCGGATCCTAATTGTTCTTGCATGTTGATTGCCACCTTTCCAG

GACACTTTAAAGAGAGAATTTTGTTCCTAACAGTGTCACAGTCCCCTGATGTGGAGCAACTGAGGAGAACGATCTGGGGATTTGTGATGGGTTGCGACAATGTCAATTCTAATGATTTCATTTTACATGGGAGGCCTTCAAATTCAGCGCTTTTGGTTTTGGATGATGTGTGGTCAATTTCAGTTCTTGAAAATCTGATTCCAAATGTAACTGGCTGCAAAACTCTTGTTGTTTCACGATTCAAATTCCCTGAAGTTCTTAGAGAAACTTATGAAGTAGAGTTGTTGAAGGAAAGTGAAGCAATTGCTCTGTTTTGCCACTCGGCTTTCGGACAACAGTCTATTCCTTTGTCTGCTAACCACAACTTGGTCAAACAG

GTAAAGAACATAACATTCTTTCTGTTGGATTAGACACATGACAATTATCTTCAAAATGTCTAATTTAGTGGTTTTATTTCGCTCATTTGTTTACAAATTTTCTTTGAAG

GTTGTGAATGAGTGCAAATGTTTGCCTCTGGCTCTTAAAGTAATTGGAGCATCACTCAGAGGACAGAGCGAGATGTTCTGGAATAATGCCAAGTCTAGGTTGTCACGTGGCGAGCTTATTTGCGAGTCCCATGAAAACAAATTGCTTCGAAGAATGGCAATCAGTATTGAACGCCTCTCGAGTAAAGTGAGAGAATGTTTTCTCGATCTAGGATGCTTTGCTGAAGACAAAAAGATTCCTCTCGACATTCTCATCAATGTTTGGAAGGAGTTGCATGATCTTGATGACGAAGAAGCTCTTGCCGTTCTTTTCGAGTTATCTCAGAAGAATCTTCTTACGTTGGTGAAAGATGCACG

GTATGGACACAATTATGTTACATCACTTATTTATGTAATTACTACAGACATCGCAATATGTTGGCTAAATACTTGATTGGTCGATATGCAG

CGGTGGTGACATTTATAGCAGTTATTATGAGATGTATGTCACTCAACACGATGTCTTAAGGGACCTTGCCCTTCATTTCAGTTGCCAGGAGAATGTAAATGACCGCAAGCGATTACTGATGCCAAAAAGCGACACAGAGCTTCCAAAAGAATGGTTAAGGAAATCAGCACAGCCATTTAATGCCCAGCTTGTTTCAATTCACACAG

GTAACTGATTACCCTTTATCGAAGGAAGATAAGAGTTATAAAAATAAGCATCCTGTAAAGCTTTTTAGGCAAAGAAATTCATTGTCTGAGTTACCAAGACATGGCACTTCTATGATTATAAACTTTTTATACCATGTCCTTGAAGTATTGTCTCAGCTTCATTACACATTTATGTTATAG

GTGAAATGAAAGAAATGGATTGGACGCACATGATATTTCCTGAAGCTAAAGTGCTCATTCTAAACTTCTCCTCGATTGGATACTTCTTGCCTTCTTTTCTTTGCAACATGCCGAAGCTAAGAGCATTAATTGTGCTAAATAACAATGCAACACATGCAACTCTCTCCAATTTCTCAGTTTTTTCTAGTTTGGTCAACTTGAGAGGCATCTGGCTGGAAAAAATTTCCATGACACGACTATTCGATGCTTGTACGCCATTGAAACATCTAAGGAAGATATCTCTTGTTTTCTGCAAGATTAACAACAGCCTTGACGAGTCAGCGATACATCAGATCTTCCCGTTCCTTTTCGAACTCAAAATTGATCACTGCAACGACTTGTTTAAGCTACCTTCAAGCATTTGTGAGATGCAAAGTCTGAAGTGTCTTAGTGTCACCAACTGCCATAATCTCAGTCAACTCTCTACCAACTTATGGAAGCTGAAAAATCTACAAATCTTGAGGCTTTTTGCTTGCCCACTTCTCACATCTCTACCCCCAAGCATTTGTGTACTTTCTTGTCTAAAGTACATTGACATCTCCCAATGTGTCTACTTAACCAGCCTTCCTGAAGAATTCGGTAAGCTGACAAACCTAGAGAAAATTGACATGAGAGAATGCTCGCTCATAAGGAGACTACCTAGATCAGTTGTGTCTTTGCAATCTCTCTGTCATGTAATCTGCGAAGAAGACGTCTCGTGGCTATGGGAGGATTTGAAGGGTCACATGCCTAATTTGTACATTCAAATCGCCGAGAAATGCTTTGACTTAGATTGGCTCAACGAGTGA

>MELO3C004320

ATGGACATCCTTATTTCAGTCACCGCAAAAATTGCTGAATACACTGTTGAGCCTGTTTTACGCCAACTTCGTTATGTATTTTTCATTCGTTCCAACTTTCGAGAACTTAAGACTCAAATAGAAAAGCTGAAGATTACAAGAGAATCTGTGCTACACAACATCCATTATGCAAGAAGAAATGCTGAAGACATAAAACCTGCCGTTGAGGAATGGTTGAAAAAGGTTAATGACATTGTTGGAAAATCTGAGGAGATATTAGCCTATGAAGGTGGACATGGTAAACTGTGTTCCACCAATTTGGTCCAACGACACAAGTTAAGTAGAAAAGCAAGCAAAATGGCCTATGAGGTTGGTGAGATGAACACCGAGGGGAAAAGTTTTGATACAGTATCCTACAAAATTGTTATCCCATCGGTTGGTTGTTCACCGACAAAAGTACCTGACTTTCTTGATTTTGACTCAAGAAAGTCAATTGTGAAACAAATCATGGATGCACTCTCTGAAGATAATGTCCATAGGATCGGAGTGCACGGGATGGGGGGTGTTGGAAAAACAATGCTAGTGAATGAAATTTTAAGAAAAATTGGGGAGAGTAAGAAGCTTTTTGACGAGGTGGTAACATCTACGATCAGCCAAACATCAGATTTTAAAAGAATTCAAGGAGAACTAGCTGACAAGCTAGGTTTGAAATTCGAACAAGAAACAATAAAAGGAAGGGCTTCTATTCTAGAAAAGAGGTTGAAGATGGAGAGAAGTATCCTAGTCGTGTTGGATGATGTCTGGGAGAATATTGATTTGAAAGATATAGGAATTCCAAGTGTTGAAGATCATACGGGATGCAAGATCTTGTTTACCACTAGGAATAAAGATTTGATCTCAAATCAAATGTGCGCCAATAAAATTTTTGAGATAAAAGTTTTAGGAGAAGATGAGTCATGGAATTTATTTAAGACAATGGCAGGTGAAATTGTGGAAGCAAGAGATTTGAAGCCTATAGCCATTCAAATTGTGAGAGAATGTGCAGGTTTGCCTATTGCTATTACTACTGTTGCTAAGGCATTACGAAATAAACCATCCGACATTTGGAATGATGCCTTAAATCAGCTTAAAAGTGTTGATGTGGGTATTGCAAACATTGGAGAAATGGAAAGGAGAGTGTATTTGCCACTAAAACTGAGTTATGATTACTTGGGATATGAAGAGGTGAAGTTATTATTCTTGTTATGCAGCATGTTTCCAGAAGACTTTACCATTGACGAGGAAGAGTTGCATGTATATGCCATAGGCATGGGATTCTTACATGGTGTTAATACTGTGGAAAAAGTACGATGTAGGATTAAAAAATTGGTTGAGGATCTTATATCTTCTTCTTTGCTTCAACAATATTCTGAGTATGGATGCAATTATGTGAAAATGCATGATATGATTCGTGATGTAGCCCTATCAATTGCATCTAAGAATGAACACGTACGTACATTGAGCTACGTGAAAAGATCGAATGAAGAATGGGAAGAAGAGAAACTATCGGGTAATCATACCGCAGTGTTCATTGATGGTTTACATTATCCTCTCCCGAAGTTAACGTTACCCAAAGTTCAATTATTAAGGTTAGTTGGACAATCTTGGGAACATAAGTTTGTGTCGGTGGTAGAAACTTTGTTTGAAGAAATGAAAGAGCTCAAAGGTTTAGTATTAGAAAACGGAGCATAG

>MELO3C004321

ATGGACATCCTTATTTCAGTCACTGCAAAAATTGCTGAATACACTGTTAAGCCTGTTGGACGCCAACTTGGTTATGTATTTTTCATTCATTCTAACTTTCAAAAACTTAAGACTCAAGTAGAAAAGCTGAAGATTACAAGAGAGTCTGTGCAACACAAGATCCATAGTGCAAGAAGAAATGCTGAAGACATAAAACCTGCCGTTGAGGAATGGTTGAAAAAGGTCGATGACTTTGTTCGAGAATCTGACGAGATATTAGCCAATGAAGGTGGACATGGTAGATTCTGTTCCAGCAATTTGATCCAACGACACAAGTTAAGTAGAAAAGCAAGCCAAAAGGCATATGAGGTTCTTGAGATGAAAAATGAGGGGGAAAGTTTTGATACAGTATCCAATAAAAATGTTATCCCATTGGTTGATTGTTCACTGCCAAAAGTACCTGACTTTCTTGACTTTGACTCAAGACAGTCGATTGTGAAACAAATCATGGATGCACTCTCTGATGATAATGTCCATAGGATTGGAGTGTATGGGATGGGGGGTGTTGGCAAAACAATGCTAGTGAAGGATATTTTAAGAAAAATTGTGGAGAGTAAGAAGCCTTTTGATGAGGTGGTATTATCCACGGTCAGCCAAACACCAGATTTTAGAAGTATCCAAGGACAACTAGCTGACAAGCTAGGTTTGAAATTCGAACAAGAAACAATAGAAGGAAGGGCTACTATTCTACGAAAGAGGTTAAAGATGGAGAGAAGTATCCTAGTTGTGTTGGATGATGTTTGGGAGTATATTGATTTGGAAACAATAGGAATTCCAAGTGTTGAAGATCATACGGGGTGCAAGATCTTGTTTACCACTAGGATTAAACATTTGATCTCAAATCAAATGTGCGCCAATAAAATTTTTGAGATAAAAGTTTTAGGAAAAGATGAGTCATGGAATTTATTTAAGGCAATGGCAGGTGACATTGTTGATGCAAGTGATTTGAAGCCTATAGCCATTCGAATTGTGAGACAATGTGCAGGTTTGCCTATTGCTATTACTACTGTTGCTAAGGCATTACGAAATAAACCTTCTGACATTTGGAATGATGCCTTAAATCAGCTTAAAAGTGTTGATGTGGGTATGGCAAACGTTGGAGAAATGGAAAAGAAAGTGTATTTGTCACTAAAACTGAGTTATGATTGCTTGGGATATGAAGAGGTGAAGTTATTATTCTTGTTATGCAGCATGTTTCCAGAAGACTTTCCCATTGACGTGCAAGAGTTGCATGTATATGCCATGGGCATGGGATTCTTACATGGTGTTGATACTGTGGTAAAAGGACGATGTAGGATTAAAAAATTGGTTGATGATCTTATATCTTCTTCTTTGCTTCAACAATATTCTGAGTATGGGTGCAATTATGTGAAAATGCATGATATGGTTCGTGATGTAGCCCTATTAATTGCATCTAAGAATGAACACGTACGTACATTGAGCTATGTGAAAAGATCGAATGAAGAATGGGAAGAAGATAAACTATTGGGTAATCATACCGCAGTGTTCATTGATGGTTTACATTATCCTCTCCCGAAGTTAACGTTACCCAAAGTTCAATTATTAAGGTTAGTTGCACAATAT

>MELO3C004324

ATGGACATCCTCATTTCAGTCACTGCAAAAATTGCTGAATACACTGTTGAGCCAGTTGGACGCCAACTTGGTTATGTATTTTTCATTCATGCCAACTTTAAAAAACTCAAGACTCAAGTAGAAATACTGAAAGACACAAAAGAATATGTGCAACAAAATATTCGTACTGCCAGAAGAAATGTAGAAGACATAAAACCTGCAGTTGAAAAATGGTTGAAAAAGGTTGATGACATTGTTGGAAAATCTGAGGAGATATTAGCCTATGAAGGTGGACATGGTAGACTGTGTTCCACCGATTTGGTCCAACGACACAACTTAAGTAGAAAAGCAAGCAAAATGGCCTATGAAGTTCTTGAGATGAACACCGAGGGGAAAAGTTTTGATACAGTATCCTATAAAATTGTTATCCCATCGGTTGATTGTTCACCGCCAAAAGTACCTGACTTTCTTGATTTTGACTCAAGAAAGTCGATTGTGGAACAAATCATGGATGCACTCTCCGAAGATAATGTCCATAGGATCGGAGTGCACGGGATGGGGGGTGTCGGAAAAACAATGCTAGTGAAAGAAATTTTAAGAAAAATTGGGGAGAGTAAGAAGCTTTTTGATGAGGTGGTAACATGTACGATCAGCCAAACACCAGATTTTAAAACTATTCAAGGACAACTAGCTGACAAGCTAGGTTTGAAATTCCAACAAGAAACAATAGAAGGAAGGGCTCCTATTCTACGAAAGAGGTTGAAGATGGAGAGAAGTATCCTAGTTGTGCTGGATGATATCTGGGAGTACATTGATTTGGAAATAATAGGAATTCCAAGTGTTGAAGATCATGCGGGATGCAAGATCTTGTTTACCTCTAGGAATAAACATTTGATCTCAAATGAAATGTGCGCCAATAAATTTTTTGAAATAAAAGTTTTAGGAGAAGATGAGTCATGGAATTTATTTAAGGCAATGGCAGGTGAAATTGTTGAGGCAAGTGATTTGAAGCCTATAGTCATTCAAATTGTTAGAGAATGTGCAGGTTTGCCTATTGCTATTACTACTGTTGCTAGGGCATTACGAAATAAACCTTCCGACATTTGGAATGATGCCTTAGATCAACTTAAAAGTGTTGATGTGGGTATGGCAAACATTGGAGAAATGGACAAGAAAGTGTATTTGTCACTAAAGTTGAGTTATGATTGCTTGGGATATGAAGAGGTCAAGTTACTATTCTTGCTATGCAGTATGTTTCCAGAAGATTTTGACATTGATATGGAAGAGTTGCATGTATATGCCATCGGCATGGGATTCTTACATGGTGTTGATACTGTCCTAAAAGGACGACGTAGGATCAAAAAATTGGTTGATGATCTTATATCTTCTTCGTTGCTTCAACAATATTCTGAGTATGGGCGCAATTATGTGAAAATGCATGATATGGTTCGTGATGTAGCCCTATTAATTGCATCTAAGAATGATCACATACGTACATTGAGCTACGTGAAAAGACCGAATGAAGAATGGGAAGAAGAGAGACTATCGGGTAATCATACCGCAGTATTCATTTATGGTTTACATTATCCTCTCCCGAAGTTAACGTTACCCAAAGTTCAATTATTAAGGTTTGTTGGACAATGGATGGAAGATAAGCGTGTGCCGGTGGTAGAAACTTTGTTTGAAGAAATGAAAGAGCTCAAAGGTTTAGTATTAGAAAACGTGAATATATCCTTGATGCAACGACCATCTGATCTTTACTCCTTAGCAAACATTAGAGTATTACGTTTGCAAGAATGTGGATTAGAGAGCATAGATATGATTGGTGAATTAAAAAAACTTGAAATTCTTGATTTTAGTAAATCTAACATCACACAAATTCCTACAACCATGAGCCAATTGACACAACTCAAAGTGTTAAATTTATCTTCTTGTAATCAACTCAAGGTAATTCCACCAAATATTCTTTCAAAGTTGACAAAACTGGAAGAATTAAGTCTGGAAACTTTTGATAGATGGGAAGGAGAAGAATGGTATGAAGGAAGGGAAAATGCTAGCCTTTCTGAACTCAAGTGCTTGCCACACCTTTATGCTTTAAATTTAACCATTCAAGATGAAGAAATTATNNNNNNNNNNNNNNNNNNNNNNNNNNNNNNNNNNNNNNNNNNNNNNNNNNNNNNNNNNNNNNNNNNNNNNNNNNNNNNNNNNNNNNNNNNNNNNNNNNNNNNNNNNNNNNNNNNNNNNNNNNNNNNNNNNNNNNNNNNNNNNNNNNNNNNNNNNNNNNNNNNNNNNNNNNNNNNNNNNNNNNNNNNNNNNNNNNNNNNNNNNNNNNNNNNNNNNNNNNNNNNNNNNNNNNNNNNNNNNNNNNNNNNNNNNNNNNNNNNNNNNNNNNNNNNNNNNNNNNNNNNNNNNNNNNNNNNNNNNNNNNNNNNNNNNNNNNNNNNNNNNNNNNNNNNNNNNNNNNNNNNNNNNNNNNNNNNNNNNNNNNNNNNNNNNNNNNNNNNNNNNNNNNNNNNNNNNNNNNNNNNNNNNNNNNNNNNNNNNNNNNNNNNNNNNNNNNNNNNNNNNNNNNNNNNNNNNNNNNNNNNNNNNNNNNNNNNNNNNNNNNNNNNNNNNNNNNNNNNNNNNNNNNNNNNNNNNNNNNNNNNNNNNNNNNNNNNNNNNNNNNNNNNNNNNNNNNNNNNNNNNNNNNNNNNNNNNNNNNNNNNNNNNNNNNNNNNNNNNNNNNNNNNNNNNNNNNNNNNNNNNNNNNNNNNNNNNNNNNNNNNNNNNNNNNNNNNNNNNNNNNNNNNNNNNNNNNNNNNNNNNNNNNNNNNNNNNNNNNNNNNNNNNNNNNNNNNNNNNNNNNNNNNNNNNNNNNNNNNNNNNNNNNNNNNNNNNNNNNNNNNNNNNNNNNNNNNNNNNNNNNNNNNNNNNNNNNNNNNNNNNNNNNNNNNNNNNNNNNNNNNNNNNNNNNNNNNNNNNNNNNNNNNNNNNNNNNNNNNNNNNNNNNNNNNNNNNNNNNNNNNNNNNNNNNNNNNNNNNNNNNNNNNNNNNNNNNNNNNNNNNNNNNNNNNNNNNNNNNNNNNNNNNNNNNNNNNNNNNNNNNNNNNNNNNNNNNNNNNNNNNNNNNNNNNNNNNNNNNNNNNNNNNNNNNNNNNNNNNNNNNNNNNNNNNNNNNNNNNNNNNNNNNNNNNNNNNNNNNNNNNNNNNNNNNNNNNNNNNNNNNNNNNNNNNNNNNNNNNNNNNNNNNNNNNNNNNNNNNNNNNNNNNNNNNNNNNNNNNNNNNNNNNNNNNNNNNNNNNNNNNNNNNNNNNNNNNNNNNNNNNNNNNNNNNNNNNNNNNNNNNNNNNNNNNNNNNNNNNNNNNNNNNNNNNNNNNNNNNNNNNNNNNNNNNNNNNNNNNNNNNNNNNNNNNNNNNNNNNNNNNNNNNNNNNNNNNNNNNNNNNNNNNNNNNNNNNNNNNNNNNNNNNNNNNNNNNNNNNNNNNNNNNNNNNNNNNNNNNNNNNNNNNNNNNNNNNNNNNNNNNNNNNNNNNNNNNNNNNNNNNNNNNNNNNNNNNNNNNNNNNNNNNNNNNNNNNNNNNNNNNNNNNNNNNNNNNNNNNNNNNNNNNNNNNNNNNNNNNNNNNNNNNNNNNNNNNNNNNNNNNNNNNNNNNNNNNNNNNNNNNNNNNNNNNNNNNNNNNNNNNNNNNNNNNNNNNNNNNNNNNNNNNNNNNNNNNNNNNNNNNNNNNNNNNNNNNNNNNNNNNNNNNNNNNNNNNNNNNNNNNGATGA

>MELO3C008573

TGGATATAATTTCTCCTTTCATTGGAGCAATTGTGGAGTACACTATACACCCCATTGGTCGTCAATTGAGTTATCTATTCTTCATCCGCCGAAATATTCAAAACCTTAAGAGTCGGGTTGAAACGTTGAAGTACCTTAAAGAATCGGTGCTTCATAAGGTCAACGAGGCGAGAAGAAATGCTGAAAACATAGAATCTGGTGTTCAAAATTGGTTGACTAAGGCGGATTCCATCATTGAAAAATCTGAAACATTACTAAACAATCTTGCTCAACAGGATGGATTGTGCTTGAATTTGGTCCAAAGACACAAATTAAGTAGGAAAACTGTGAAGTTGGGTGATGAGGTTGTTGAGATAAAAAATGAGGGAAATTTCGATAGAGTCTCCTATCGTGTAGCTCTTTTAGAAGTTGAGCTTGAGAGTTCAAAGGCAAAGACTTCAGATTTTGTTAATTTTGAATCAAGAAAGCCAACTATTGACAAAATCATTGGTGCACTTATGGATGATAATGTCCATAAAATTGGAGTGTACGGGATGGGAGGTGTTGGCAAAACAATGTTAGTGAAAGAGATTTCAAAATTAGCTATGGAGAGAAAGCTATTTGATGAAGTGGTCACATCGACCATTAGTCAGACACCAGATATAAAGAGAATTCAAGGACAACTTGGTGATAAACTTGGACTCAAATTTGATCAAGAAACAGAAGAAGGAAGAGCTCTTATGTTACAGAAAAGGTTGAAGATGGAACGAAGGATCTTCATTGTACTTGATGATGTTTGGAAGCAAATTGACTTGGAAACAATAGGAATTCCAAGTATTGAAGATCACTTAGGATGCAAGATTCTATTTACCTCTAGAGATTTTAGTGTACTCTTTAACGATATGTGTGCAGATGAGATTTTTGAGATAAAAGTTTTACAAGAAGACGAGACATGGAGATTATTCAAGAAAATGGGTGGTGAGATTGTTGAAACATCTGATTTGAGGAGTATAGCTGTTGAAATAGCAAGGGAATGTGCACGTTTGCCCATTGCTATCACTACACTTGCTAAGACATTGAGAAACAAACCTTTGTCGATTTGGAAAGATGCCTTAACCCAATTAAAAAATCCTGTGGTAGTGAATATTAGAGGAATGAATGAGAAAGTGTATTCTTCACTTAAGTTAAGTTATGATCAATTAGATTGTGAAGAGGCCAAATTACTACTTTTACTATGTAGTATGTTCCCAGAAGATTGTATCATTAATAATGTGGAATACTTGCATGTATATGCTATGGGCATGGGTTTTTTGTATGGTGTTGACACTGTGACTCAAGCACGACATAGGATAACAAAATTGGTTGATGATCTCATATCTTCTTCTTTGCTTCTAAAAGAATCAACTGATGGTTTGGGTGAGTGTGTTAGAATGCATGATCTCATTCGTGATCTAGCTATACTAATTGCCTCAAAAGACGATCATATTCGTACACTAAGCTTTTCCAAAGGATTGGATGAATCATGGCCAGAAAAAGAAATGTCAGGTGATCATACAGTAGTGTACTTAAATGTTGAAGGATTGTGTAACCCTCCGAAAAAGTTAATGTTACCCAAAGTTCAATTATTGGTGTTACATGGACCATTGTTATTGGATAGATATGAGTTGTCCAAAACCTTTTTTCAAGAGACAAAGGAGCTCAAAATTGTAGAAATAATGGACATGGAATTTTCCCTCGAGACGACGACATTTCACTCTTTTGAGAAGCTTCAAGCATTGCATCTATTTAGTTGTAGATTGGGAAATATAGATAGGATTGGACACTTAAATAGCCTTGAAATTCTCAATTTTCGAGGATCAAACATCAGAAAAATTCCTATGTCGATAAGCCAATTGACGCAATTGAAAGTGTTGGGTTTATCATATTGTTCTAACTTAAAGGTAATTCCACCTAATGTTCTCTTTAATTTGAAAAATTTAGAAGAGTTGTATTTGCGAGGGTTTGATGGATGGGAAAGAGAAGATTTGAATGAAGGAAGAAAAAATGCTAGTCTTTCAGAGCTCAAGCACCTTGTCCGCTTGTGTGTTTTAACATTGTGGATTCAAGATGAAAATACTATGCCAAAACAGTTGTTTTCAAGATTGTTGAATTTGGAAAAGTTTGATATTACCATTGGTTGTGCACCTCGTGGATTTTGGTCAAGGGAAATCTCAAGAGTCTTGTGCTTGAAGATGGCCGAAACAGGAACTGACATTGATAATGGGATAAACATGTTGTTAAAGAGGTCTGAAGAATTGCATTTAGTAGGATCAGTTGGTGCAAGGGTCCTCCCTTTTGAGTTAAAAGAAAATGAAACTTTACATTTGAAGAAACTCTATATTTATGATAATTCAAAATTTCAACATTTTAACCTTGAGCAAAAGAATCCTTTCCAAAATGTTTGGTCTAAATTAGAGTATTTAAAATTAAGCAACTTGGAGAATTTGGAGAGTATATTTCATTGTGATCATGTCAGAGGATCTCAATTAAACAAGTTGAAGGTGATAAAGTTGTTAGGTTGTAATAAATTGAGAAGTCTCTTTTACTACTCCATCTTGGATGACCTGTTCCATCTTGAGGAGATTAAAATCATTGGTTGTGCGATGATGAGAACAATCGTCGGAAATGAGAAGGCAACCGAAAAAATTGAGTTGGCGAGTTTAAAGTATTTAACGCTAATGGACTTACCAAGACTTCATAGTTTTTTCTCTAAAATTGAGAAACACGAACAATCATGTCTTGATAATCTACAACCAGACAAGACAAGCAGAAACAATGACTCATTTTTCAATGAATTGGTGAGCTCTAAAACTTAA

>MELO3C008693

ATGGCCATGTGTGCCGGCGCCATTCTTAATCCAATCGGAGAAAAAATCGCCAACTGCATGGTGGATCCCGTTTTCCGGCAACTAGATTATTTGTTCCACTTTAAAACCAATGTGAATCGTCTCAAAGATCAAGGCAAGAAGCTGGTGGAAACCAGAGATTTTGTTCAACATTCTGTCGACTCCGCCAAAACCAATGGAGATGAGATCGAAGTTATGGTCACTGAATGGTTGACCATAGCTGATCAATTTAGTCAAGATGTCGATAGGTTTTTCATCGAAGCCAACGGCCGAAGTCTTCGATGGTGGAATATGTTTTCACGCCATCGATTTAGTAGAAGAGCTACCAAATTGGCTGGGGTAGTTGATGAAGCCATTCAAGGTGGGGGTTTCGAGAGAGTTGGGTTCCGCAAACCTCCACAAGAAATTATGACGCTAAGGAACAAGTTCGAAGCCTTTGAATCTAGGGTTTTGATTCTGAAGGAGATAATTGAAGCGCTTGGCGATGCTAATGCGAGGGTGATTGTGGTACATGGGATGGCGGGAGTTGGGAAAACCACCCTAGTTGAAGAAATTGCAAGATTGGCTAAGGAGGGAAAGCTTTTTGATGCTATAGCAATGGTGACTGTAAAGCAGACTCCAAACATTAAGAAAATACAGGGGGAGATTGCTGATCAGTTAGGGTTGAAATTTGAAGAGGAAAAGGATCGAATTAGGGCTGATCGACTACGTCGAAGGTTAGAGATGGAGAAGAAGGTGTTACTGGTTTTGGATGATGTTTGGAGTAAGCTTGATTTGGAAGCTGTTGGAATTTCTAGTCATCACAAGGGATGTAAGATACTTGCAACTTCTAGAAAGGATGACGTGTTTTTCAATGATTTTGGTAATCAGAAAAATATATATATCAATATTCTGTCAAAAAAAGAAGCAAGGGATTTTTTCAACAAGGTGGCATGTGATTCTGTTGAATCTTCTGATGATACTGATCCTGAAATGGAAGCTGTTGCTATTGAATTGGCAGATGAATGTGGAGGATTGCCACTTTCTCTTGCAACTGTTGGACAAGCCTTAAAAGGTAAAGGGCTTCCAAGTTGGAATGATGCCTTGCAAGGAATGAAGTTTCCTGGTGAACCCAGTAACTATGGGGTGAATAAAGTGGCATATTTGTCTCTGAAAGTGAGTTATAAATCTCTAAACACAGATGAAGCCAGATCATTATTCTTACTATGTAGCTTGTTTCCAGAAGATTATCAAATTAACATCAAATACTTGTTGATGTATGCCATGGGTTTGGGGTTATTAAACGCCATGAGTTCTCTAGCAATGGCAAAATGGAGAATACTTTCTTTGGTTGATGAGCTCAAAACTTCTCACTTGTTGCTTGATGGGGCTGATAGCGATTTTGTGAAAATGCACGATATAGTTCGAGACACAGCAATTTTGATTGCGTCGAAAATGAAGTCCAAATATTTGGTTAGACATGGTGCTGGAGAGAGTTTGTGGCCCCCAATGGATGAGTTCAAAGATTACACTGCAATCTCATTAGATTGCAGTGATCACTCGGAACTCCCAGAATTTATATGTCCACAGCTTAGATTCTTATTACTGGTAGGAAAAAGAACATCTTTGCGATTACCTGAAAAGTTTTTTGCAGGTATGCAGGAACTAAGAGTTTTAGATCTCACTGGCTTATGTATTCAGCGGCTTCCACCATCAATCGACCAACTGGTAAATCTTCAAACACTGTGTTTAGATGACTGTGTTTTGCCAGACATGTCTATAGTTGGTGAATTGAAAAAGCTTGAAATTCTTAGCTTGAGAGCATCTGATATTATTGCACTTCCTAGAGTAATTGGGGAACTTACCAATTTGAAGATGTTGAATTTGTCTGATTGTTCTAAACTCAAGGTGATCCCTGGTAACCTGTTATCTAGGTTGACAGGGTTGTCTGAGCTATACATGGACAATAGTTTTAAACATTGGAATGTAGGACAGGTGGAAGGTTATGTTAATGCAAGGATTTCTGAATTAGACAAACTGCCACGGCTGACCACTCTACATGTGCATATTCCAAATCCCACCATTCTACCAAATGCCTCTGTCTTTAGAAAATTGAGTGGTTACAGAATACTAATTGGAGATGGATGGGATTGGTCTGGCAATTATGAAACTTCAAGGACCTTGAAACTCAAGCTTGATAGTAGCATTCAGAGAGAGGATGCAATTCAAGCACTTCTAGAGAATATTGAAGATCTGTATCTAGATGAATTAGAAAGTGTCAAGAATATTCTATTCAGTCTAGGCTATGAAGGCTTTCCGAAATTGAAATGTTTACGTGTAAAAAACAATGGTGAAATTGTGACGGTTGTCAACTCGGATACTATGCATCATCCACACAGTGCCTTTCCATTGTTGGAGTCCTTATTTCTGAAAAATTTAGCTGAACTTGGAAGCATTTGTCGTGGAAAGCTTCCACAAATGTCCTTCCGTAACTTGAAAAGAGTAAAAGTTGAAAGTTGTGACAGATTAAAATTTGTTTTCCCATCTTTCATGGTCAGAGGCCTTATACATCTTCAAAGCCTGGAGATTAGTGAATGTGGCATCATAGAAACTATAGTTTCGAAAAGCAAAGAAACAGAAATGCAAATCAATGGTGATAAGTGGGATAAGAACATGATTGAGTTTCCTGAATTGCGTTCTCTGGTACTTCAACATCTCCCAGCGCTTATGGGTTTCTATTGTCATGATTGCATAACGGTGCCTTCAACTAAAGTGGATTCACGTCAAACAATTTTTACTATTGAACCTAGTTTTCTTCCACTTCTCAGTCAACAG

GTATGTTATCTATAATTGTACTTCTTCCATTGTTATCTTTGAGCTAAATATAGACTAAATATATGTTATAGTGTCCAAGGTTTTTAAAAATTAGTTCATGTAGTTTAAAAGCTCTAACTTTCATCTCTTATGTTTTGACATTGTCCAAATTATTACATCTTTGTCGGTCGTATTGATTGGTGATAATCTTACGTATTTGGAGAGTCGGACAAAAATTCGAAGTAGGAACTTGGATTATTGAAAAGTAAAGTTTTCTGAATAGATTTTTTGCCAATTTCTTAGAAGGTTAGCTATAAATTAGAATTTATTTTAGTTGGCTTAATTCCTCCTTTATGTTCGTTCCAATTCACTTTGTATCATGTCTTATCATCAATCAATTTTAACACTCACCATCAAAAAGAGTATAAATAATAGGTAAATATATACGTTTTGGTTTATTGTTTGGGTGTCTTTTCAATTTGGTTAGAAGTTTCAATTTAGTCCTATTATGTTTTAACATTGTGTAATGTACCTTCATTGTTAGAAAATATGAAATTTGCGTGTGATAAACAGGCTTGTGATGAACAAACGTGATTACCAAGGCTATCCATAAAAAAAAAAAATTGATTCTTTAGGAAACGTAACTGATCTTCCAAATTTCTTATGGTCATAAGCCAATTTGAAATTTTCTAACGAAGGGTATGATCGGAACATATTAAAACAAAAAGGACTAAACTGAACATTTTTAAATATTAGAAACCAAAATGTATTTAGCCTAATTATAGTTGATAATTGTTGGAAACTTAAGGAATTGAGTTGAAACTTTTCAGAATTTAGAGACTAAATTAAAGAAACCCCCAACCATTGAGGATTGAATCGTATATTTGGCCTTTATCTTTCTATGGCCTTTAAATTTACAAATCCAACATTGTGAAATAATACAATTAGCCATGAACGATAATGGGTTTTGTTTGCTTTCTGGTTAGATTGTTTCTCGTCTTCATTCTCACAATTATGTATTGAGTCTTTGACTATTGATAAACAAATTGTTATTTTCGTAATGTTTTGTAG

GTTTCCTTCCCCAAATTGGAGACATTAAAATTACATGCTTTGGAATCGGGAAAGATATGGCAGGATCAACTTCCTTCTAGCTTTTATGGCGTTAAGAATCTAACTTCTTTGAGTCTGGAGGGTTGTGCTTCAGTAAAATATTTAATGACAATCACTGTGGCTAAAAGCCTTGTGAATCTTGAACGCCTCGAACTAAACGACTGTAAGTTGATGAAAGCTATAATCATTTCAGAAGATCAAGATCTGGACAACAATTACCCTTCCAAATCTATCTTGCAGAGCAAG
GTACACTATTAACCTCATCCATATTTCTTCCTTCTATGTTTTGTTTATAAACTTTAAGCTTAAAATGTCTGTCTTACAAATTTTCATTTATGTCCATTAACTTTAAATTATGGACAATCTATTTAGTGTCTATGGTTAACATAAGCAAGGTGATATGGTCATTACAACCTACTCCTCAAATATCTCTATAATAATGTGGTATTGTCCATTTTTGTTGGTATAAGCTCTTATGGTTTTGCTTTTGAAATCAACAAAAATGTTTCATACCAAAGGAGATAATTGCCTTCACTTATGTACTCATGACCATTTCCCATTCCCTAACTATGTTTAGAATGCAAATATAACATTCTTATGAGAATATAACATTCTTATGAGATAAAAACTGATTGGTTATACAGCGAAGAAGTGATAAATGCAAATGACCGGATTAGAAATAGGTCAATATAGATGGGTTAAGTTAATATTTTTATATGTCAGCTTTTGCCAAGATATGGTAAATCATGTAATATGTTTATAATCATGTCATATCCAAAAAAGAGGTATATATATTATTATAATTTTCTAAAAAAAGGTTCAGAGGCTTCCAGAATATTCAAAACTCAAGGACTTCAACTTGTTCATATATTTAAACTTACCGTACTAGTTTGATTTTTGAGCAAAAGTTATAATTAAATTGAAATGATGAATATAATGCAG

GATGTTTTTCCGAACCTGGAGTCCCTCTTAATCTCTCGCATGGATGCTTTGGAGACATTATGGGTCAATGAAGCTGCTTCAGGATCCTTCACAAAGCTGAAAAAAGTGGACATCAGACATTGCAAAAAACTAGAGACAATCTTTCCAAATTACATGCTGAACAGAATGACAAATCTCGAGAGATTAAACGTTACAGATTGCAGTTCCCTAGTGGAGATATTTCAAGTGAAGAAAATCCCAGTTATCAATGCCAACCAAGTAACAGCCGTTGGAGCTAACCATTTGAAAGAGTTGAAGCTGCTTCGTCTACCTAAGCTAAAGCACATATGGAGCTCGGATCCACACAAATTCTTAAGCTATCCATCTCTCCAACTTGTTCATACAATTCATTGTCAAAGCCTTTTGAATCTCTTCCCTGTATCCATAGCTAAGGATCTTGTACGACTTGAAGTGCTTAAAATACAGTTCTGTGGAGTTGAGGAAATTGTTGCTAAACGAGGGGATGATGGAGATGGAGATGATGATGCGTCGTTTGTGTTGAGTGGGTTGACATCATTGACTCTTTGGAATTTGTTCGAGTTCAAGAGGTTTTATCCAGGGAGATATACTTTAGAATGTCCATCATTGACAGCGCTAGATGTACGCCACTGCAAATCATTCAAGTTGATGGAAGGAACTTTGGAAAATTCATCATCAATCTCATCCGCGGTTGAAAAGGTATTTACATTTTCTCTAATTCTTATATGCTATTTAGCGTCCTTTTTATAAT

>MELO3C024717

ATGGACATTCTAGTCTCAGTCATTGCAGCAACAATTAAACCTATTGGACATCAATTAGGTTACCTTGTTTGCTACAACAGAAACAAGAAGGAGCTTAGAGACCAACTTGAAAATCTTGAGACTACTAAAAGGGATGTGAATCAAAGGGTTCAAGAGGCAAAAGGCAAATCATATACAATCTCTGAGGAAGTTTCAAAGTGGTTGGCCGATGTGGATAATGCAATACTCCATGATGAACTATCTAACTCCAACCCATCTTGCTTTAACTTGGCTCGACGATACCAGCTAAGTAGAAAAAGAGAGAAGCAAGTGAATTATATTCTTCAACTCATGAACAAAAGAAACAGCTTTGTCGAAGTTGGGTATCGTGCACCTCTTCCGGATACTGAGAATATTGTTGTTCCCGGAGATTACCAAGTTTTGGAATCAAAAACATCATTGGCTAAAGATATCAAGAATGCGCTTGCGAAACCTGAGGTCAAAAAGGTTGGTGTATATGGTATGGCAGGTGTTGGAAAAACTTATTTGCTTAACGAAGTTAAAAAATTGGTGTTGAAGGGGGAAGACAGATTGTTTGATCGAGCAATTGATGTGCGTGTAGGTCGATTTAATGATGTAACGGAAATACAAGAACAAATTGGTGACCAATTGAACATAGAATTGCCAAAAAGTAAAGAGGGAAGAGCGTCTTTTCTACGGAATAATTTGGTGAAAATGGAAGGTAATATCCTCATTTTATTAGATGATTTGTGGAAGGAATATGATCTTTTAAAAGAGATTGGGATTCCATTAAGTAAAGAAGGATGTAAGGTACTCATGACAAGTCGATCACAAGATATATTAACCAATAATATGAATACACAAGAGTGTTTTCAGGTGAGTTCGTTATCTGAAGAAGAGTCTTGGAAGTTTTTTATGGCAATCATTGGTGATAAGTTTGATACAATTTATAAGAAAAACATTGCAAAGAATGTTGCAAAAGAATGTGGAGGGTTACCACTTGCACTTGATACCATTGCAAAAGCATTGAAGGGGAAAGATATGCACCATTGGGAGGATGCTTTAACCAAATTGAGAGATTCTATTGGAATGGATATTAAAGGGGTGAGCGACAAAGTTTATGCTTCACTTAGATTGAGTTATGAACATCTAGATGGAGAAGAAACAAAATTACTATTTCTTCTTTGCAGCGTATTTGCTGATGATTATAAGATTCCTATAAAAGATTTGCAAATGTATGCCATGGGTATGAGATTATTGAATAAAGTAAAAACTTGGGAGGATTCAAAAAATAGGGTAATGAAGTTGGTTAATGATCTAATATCATCTTCTTTACTTCTCGAGGCCGAGAGCGATTCCAAAGACAAGTATGTTAAAATGCACAATGTGGTTCGTGATGTTGCGATACACATTGCATCCAAGGAAGGTAACATGTCTACATTTAACATTGGATATAATAAAGTTAATGAATGGGAAGATGAGTACAGAAGTGGTTCTCATCGTGCCATTTTTGCAAATTGTGATAACTTCAACAATCTTCCCCTAAAGATGAATTTTCCACAACTTGAGTTGTTGATATTAAGAGTTTCTAATTGGTTGGTGGAAAATAATCTTCAAATTCCATATGCATTTTTTGATGGAATGGAAAAGCTTAAGGTTTTGGACTTGACAGGAATGTGTTGCCTCCGACCATTGTGGACAACACCATCATTAAACAACCTTCGAACATTATGTATGTTGCGTTGCGAATTTAACGACATTGATACAATTGGGGAGCTAAAGAAACTGGAAGTTTTGAGAATCGTTAAGTGTAACATGCTAGACCATTTACCTCCAACTATGAGTCAATTGACACAACTTAAGGTACTAGAAGTTTTAAATTGCTCTAAATTGGAGGTGGTTCCTGCAAACGTTTTTTCAAGTATGACAAAACTCGAAGAGTTGAAATTACAAGACAGCTTTTGCAGATGGGGAGAAGAAGTATGGTACAAAGATCGATTAG

>MELO3C000062

ATGGACATCCTTATTTCAGTCATTGCAAAAATTGCTGAATACACTGTTGAGCCTGTTGGACGCCAACTTGGTTATGTATTTTTCATTCGTTCCAACTTTCAAAAACTTAAGACTCAAGTAGAAAAGCTGAAGATTACAAGAGAGTCTGTGCAACACAAGATCCATAGTGCAAGAAGAAATGCTGAAGACATAAAACCTGCCGTTGAGGAATGGTTGAAAAAGGTCGATGACTTTGTTCGAGAATCTGACGAGATATTAGCCAATGAAGGTGGACATGGTGGACTCTGTTCCACCTATTTCGTCCAACGACACAAGTTAAGTAGAAAAGCAAGCAAAATGGTAGATGAGGTTCTTGAGATGAAAAATGAGGGGGAAAGTTTTGATATGGTATCCTATAAAAGTGTTATCCCATCAGTTGATTGTTCACTTCCAAAAGTGCCTGACTTTCTTGACTTTGAGTCAAGAAAGTCGATTATGGAACAAATCATGGATGCACTATCTGATGGTAATGTCCATAGGATTGGAGTATATGGGATGGGGGGTGTTGGCAAAACAATGCTAGTGAAGGATATTTTAAGAAAAATTGTGGAGAGTAAGAAGCCTTTTGATGAAGTG

>MELO3C002574

ATGCAACGTTATTTTTCAACTGTGGCCAAAAACTACCTAACTTCCTCGTCCAAAATCCTCAGTCGTCGCAACGATATCCGATCCTTATCGCGCCAATGTGATGTCTTCATAAACCACCGTGGCGTCGACACGAAACGTAACATCGCCGGATTGCTTCATGACCATTTTTCTAGAATAGGTCTTCGCCCATTTTTAGATAGTAAGAGCATGAAACCGGGGGATAAATTGTTTGGTGAGATTGAAGAAGGTATTCGGAGTTGTAAGGTAGGACTCGCGGTGTTCTCACCACGGTATTGTGAATCCTATTTTTGTTTGCATGAACTTGCCCTAATGGTGGAGAACAAAAAGAAAATCATTCCAATTTTCGTGGACGTTCGACCGTCTCAACTTCGTGTTGAATACAACTATAGTTGCCCCAAGAAAGAGTTGCAAAGGTTCAACTGGGCACTTGGGGAAGCCAAATACACTGTTGGACTCACTTTCGACACTGTCAACGGGGATTGGTCGGAGCTGTTGAGGAAAGCTTCAAACGCCGTGATTGATAATCTAATCGTCGGCGAAGGGGCCGGAGAGATGCCGGATAATTAG

>MELO3C004258

ATGGCCTTGCGTCAAAAGGGTGTCAACGTCTTCATAGACGACAAGCTCGAAAGGGGTGAACAAATTTCTGAAACCCTTTTCAAATCTATACAAGAAGCTTTAATTTCTATTGTTACATTCTCTCAAAATTATGCATCTTCTTCGTGGTGTTTGGATGAATTGGTGAAAATAATTGAGTGTAAGAAATCCAAGGGCCAGATTGTTTTGCCAATTTTCTATAAGGTGGATCCATTGGATATACGAAAACAGACTGGTCGTTTCAGAGAAGCATTGGTCAAACATATGCCAAAGTTCCAAACAAAGACCCAAATTTGGAGGGAAGCTTTAACTACTATGGCTAACTTGTCTGGTTGGGATCTAGGAACTAGGAAGGAGGCTGATCTTATTGGAGATGTTAAAAAAGTGTTGTCTACATTAAATCGCACTTGCATGTCCTTATATGTAGCTAAGTTTCCGGTTGGAATTGATTCTAAATTAGAATATATGAAGCTTCGTTCAAATAATCTTTTGGAAAAAAGCAACAAATTCCATAATCGGACACAACATGAGCATGAGTCTGATACTGGTGTTTACATGGTGGGGATATATGGCATATTGGAGGTATTGGTTGTCAACCTTGATAGAGGAATTAACATCATAAGGAATAGACTTCATTCAAAGAAAGTTTTTATAGTTCTCGATGATGTAGATAAACTTGAGCAATTAGAAGCATTGGTTGGTGGGCGTGATTGGTTTGGCCAAGGCAGTAGAATCATTGTGACCACAAGGAATAAACATTTACTTAATAGCCATGGCTTTGATGAAATGCACAATGTTCGAGGATTGAATCAAGACAAAGCTATTGAGCTTTTTAGTTGGCATGCTTTCAAGAAATGTTGTCCATCAAGTAATTGTGTGGACCTTTCAAAACGTGCTACGAGTTATTGTAAAGGCCATCCTTTGACTCTCGTTGTTTTGGGTTCATTCCTTTGTACCAGAGATCAAGCAAAATGGAGTAGTATATTAGATGAATTTGAAAACTCATTGAACAAAGATATTAGAGATATTCTTCGGTTAAGTTTTGATGGACTTGAAGACAAAGTAAAGGATATCTTTCTTGATATTTCTTGTTTACTTGTGGGAGAGAAAGTTAAGTACGTTAAGAATATGTTGAGTGCATGCCATGTAAATCTAGATTTTGGAATTATAGTACTCATGGATCTTTCACTTATTACGATTGAAAATAACAAAGTGCAAATGCATGATTTAATACGACAAATGGGTCATAAAATAATCAATGATGAATCTTCTGAGCTTGGAAAGCGAAGTAGATTGTGGTTGGGAACAGACACAATCAAAACCATAAAATTGGACTTGCCTAATCCCACAATGCTAAATGTGGATTCACGAGGTTTTAGAAACTTGAAAAATATGAGACTGCTTATAGTTCGATATGCAATATTTTCTTCAAAGATTGAGTACCTACCTAATAGCTTGAAGTGGATTAAGTGGCATGGATTTGCTCAACCATCTTTGCCTTCACGCTTGATTATGAAAAATCTTGTTGGACTAGATTTGCAGCATAGCTTCATCAAAAAATTTGGGAAAAGACTTGAGGTAAATTGTATTTCTATAGCATTAATGGGTAGCTTACATTTTTAA

>MELO3C004295

ATGGCTTTGCGTCAAAGAGGAATCAATGTCTTTATAGATAACAAGATTTCGAGGGGTGAAGAAATTTCTGCATCTCTTTTGGAAGCTATTGAAGAACCCAAGATCTTCATTGTTATAATCTCCGAAAACTATGCATCTTCCCGTTGGTGTTTGAATGAATTGAAAGTGGATCCATCTCAAGTACGGATACAAAGTGGAAGATTTGGAGAAGAATTTGCAAAACTTGAAGTTAGATTCTTCAACGAGATGCAAGCATGGAGGGAGGCCTTGATTACTGTTTCTCATATGTCTTGA

>MELO3C004304

ATGGGTTCTTCCACTGCTGTAACAGAATCCATCGCTTTCGAGTGGAGTTATGATGTTTTTTTTTTTAGTTTTAGAGGAGAGGATACTCGCACCAATTTCACCAGTCATCTTGATATGGTCTTGCGTCAAAAGGGTGTTAACGTCTTTATAGACGACAAGCTCCAAAGGGGTGGATCCGTCGGATATACGAATTGTAACTTCGGAGAAGCATTGGCCAAACATCAGGCTAAGTTCCAAACAAAGACCCAAATTTGGAGGGAAGCTTTAACTTCTGCTGCTAACTTGTCTGGTTGGAATCTAGGAGCTTATAGGAGGGAGGCTGATCTTATTTGA

>MELO3C004317

ATGGCAGGTGAAACTGTGGAAGCAAGTGATTTGAAGCCTATAGCCATTCAAATTGCGAGAGAATGTGCAGGTTTGCCTATTGCTATTACTACTGTTGCTAAGGCATTACGAAATAAACCATCCGACATTTGGAATGATGCCTTAAATCAGCTTAAAAGTGTTGATGTGGGTATGGCAAACATTGGAGAAATGGAAAGGAAAGTGTATTTGCCACTAAAACTGAGTTATGATTGCTTGGGATATGAAGAGGTGAAGTTATTATTCTTGTTATGCAGCATGTTTCCAGAAGACTTTCCCATTGACGTGGAAGAGTTGCATGTATATGCCATGGGCATGGGATTCTTACATGGTGTTGATACTGTGGAAAAAGGACGATGTAGGATTAAAAAATTGGTTGATGATCTTATATCTTCTTCTTTGCTTCAACAATATTCTGAGTATGGGTGCAATTATGTGAAAATGCATGATATGGTTCGTGATGTAGCCCTATTAATTGCATCTCAGAACGATCACATACGTATATTGAGCTATGTGAAAAGTTTAAATGAAGAATGGAAAGAAGATAGACTATCGGGTAATCATACAACAGTGTCTATTGATGGTTTACATTATCCTCTCCCGAAGTTAACGTTTCCCAAAGTTCAACTATTAAGGTTAGTTGCACAATCTTGGTGGGAACATAATGAGAGTGTGTCGGTGGTAGAAACTTTTTTTGAAGAAATGAAAGAGCTCAAAGGTTTAGTATTAGAAAACGTAAATATATCATTGATGCAACGAACATCTGATCTTTACTCCTTAGCAAACATCAGAGTATTACGTTTGCAAAGATGTCAATTATTAGGGAGCATAGATTGGATTGGTGAATTAAAAAAGCTTGAAATTCTTGATTTTAGAGGATCTAACATCTCACAAATTCCTACAACCATGAGCCAATTGACACAGCTGAAAGTTTTGAATTTATCTTTTTGTGAACAACTCGAGGTAATTCCACCAAATATTCTTTCAAAGTTGACAAAATTGGAAGAATTAAATCTGGAAACTTTTGATGGATGGGAAGGAGAAGAATGGTATGAAGGAAGGAAAAATGCTAGCCTTTCTGAACTCAAGTGCTTGCGACACCTTTATGCTTTAAACTTAACCATTCAAGATGAAGAAATTATGCCAGAAAACTTGTTCTTAGTTGGGAAGTTGAAGCTTCAAAAATTCAACATTCGTATTGGTTGCCAAAGCAAATTAAAGTATACTTTTGCATACCAGAACAAGAACAGAATCAAAAACTTCATTGGAATCAAGATGGAATCAGGAAGGTGCTTGGATGATTGGATAAAAAATTTGTTAAAGAGGTCGGACAATGTGCTTTTGGAAGGATCGGTTTGTTCAAAGGTTCTCCACTCAGAATTGGTAGGTGCCAATAACTTCGGATAA

>MELO3C004319

GAATTCCAGGTGTTGAAGATCATACGGGATGCAAGATCTTGCTTACCTCTACGAATAAACATTTGATCTCAAATCAAATGTGCACAAATAAAATTTTTGAGATAAAAGTTTTAGGAGAGGATGAGTCATGGAATTTATTTAAGGCAATGGCAGGTGAAATTGTGGAAGCAAGTGATTTGAACCCTATAGCCATTCAAATTGTTAGAGAATGTGCATGTTTGCCTATTGCTATTACTACTGTTGCTAAGGCATTACGAAATAAACCTTCTGACATTTGGATTGATGCCTTAGATCAACTTAAAAGTGTTGATGTGGGTATGGCAAACATTGGACAAATGGACAAGAAAGTGTATTTGTCACTAAAATTGAGTTACGATGGCTTGGGATATGAAGAGGTAAAGGTTATTATTCTTGTTATGCAGCATGTTTCCAGAAGACTTTAG

>MELO3C004323

ATGGACATCCTTATTTCTGTCATTGCAAAAATTGCTGAATACACTGTTGAGCCCTTGGACGCCAATTTGACTCAAGTAGAAAAGCTGAAGATTACAAAAGAATCTGTGAAACACAAGATCCATGCTGCAAGAAGAAATGCTGAAGACATAAAACCTGCCGTTGAGGAAAGGTTGAAAAAGGTTGATGACTTTGTTCGAGAATCTGACGAGATATTAGCCCACGAAGGTGGACATGGTAGACTCTGTTCCACCTATTTGGTCCAACGACACAAGTTAAATAGAAAAGCAAGCAAAATGGTAGATGAGGTTCTTGAGATGAAAAATGAGGGGGAAAGTTTTGATACGGTATCCTATAAAAGTGTTATCTCATCGGTTGATTGTTCACCGTCAAAAGTACCTGACTTTCTTGACTTTGAGTCAATTGTGGAACAAATCATGGATGCATTCTCTGATGATAATATTCATAGGATTGGAGTGTACGGGATGGGGGGTGTTTGCAAAACAATGCTAGTGAAGGAAATTTTAAGAAAAATTGTGGAGAGTAAGAAGCCTTGTGATGAGGTGGTACCATCCACGATCAGCCAAACACCAGATTTTAAAAGTATTCAAGGACAACTAGCTGACAAGCTAGGTTTGAAATTCGAACAAGAAACAATAGAAGGAAGGGCTCGTATTTTACAAAAGAGGTTGAAGATGGAGAGAAGTATCCTAGTTGTCTTGGATGATGTCTGGGAGTATATTGATTTGGAAACAATAGGAATTCCAGGTGTTGAAGATCATACGGGATGCAAGATCTTGCTTACCTCTAGGAATAAACATTTGATCTCAAATCAAATGTGCACAAATAAATTTTTTGAGATAAAAGTTTTAGGAGAGGATGAGTAA

>MELO3C004354

GGTTTGATTTCGCTTTCAAGTATTACATTTACAGGTATTGCAAGGATGATTTTTTGTGTAGAGATTTTGCAGTGCGAGTACAAATTTGAGGCCTAATTTTGGACAAATAAGAATACGTTTGGAGGGTTGAAGAAGAGTAGTTAATAATGGTTATCATCTACCAAAAAAAGCATAGGACCAAAACATCTGCATCTTTCCTTGGTGGCTTTGATAAAGGAAGTTCCACCACTTGTTTAACCCGTAAAGAGGATTGAGATGAAGAGATTGAAGTAGTAAGGATTCCAGAGTGGAGAGGGGGTAAGGCAGGAGAGTGGCATTAATTAGGATGGGAAGTAAAAAAGAAAGGTTGGGGGTAAAACGCAGCTTTCGAGAGGCAAGGCAGCGTAAATAAAAATAGAAAAATTAAAATAAGAAAATGCACACATTTTTTTGATTTTCTTCAGCAATTTGCACTTGAACGCTAAAAGAAGTATTCGACAAAAAAGGTTATTTGTTGCAGCGATAACACAAAACCTTAAGCTTAGCTTTACAGAAATCTCTTCCCTCCTCAAGTTGTTGAATGATTCTGATTTCTCTCCCACCATAACAATACTTTGAAGATGATCTCTTATAAATACATCGCCACGCTCCCATGATCCATAATATTTAATTGTACGCGTACCTTAGAAAGGTATATAAGAAGCTGTCATGCATATAGCCAATCCAAGTTGACTTCTTCCTTCTGTTCTTTTACCACGTTGCCAAGCCGAGGTGAGACAAAATTAACCCATTTTGCTATCTCCCTTTCACAAACTACTTTCTTCAAACTTTTTAGATTTCTTACTGATTTTGGAAGCTTGTGCATGTTCGGACAACTCCACATATTAAGCTTTTCCAACTTCTGCAAGTTGCCAATCTTATCTGGAAGTTTGGTAAGGCCAACACAATGAGATATGTCAAGATCAACTAATTCTTGGAGCCTTGAGATTGATTCTGGCAACTTCTCCAAATGAATACAAGATCTAAGCCTCAGAATTTTTAGATTAATCAACTGCCCAATTTCCTCTGGTAACGAAGATAATCCATGACAGTTTGTAATGCTCAGTTTCTCCAATGTGAAAATTTCACATAGTCCGACAGGGAGAGCCACCAAATCGTTGCAAAAATCTATGGAAATCTCGAGTAAGTTAGGCAAAATGGATGAGATCTGGGTCGAGCAGTTCGTGAAAGCCTTATCAATTTTGCACATAAAGAATGATAGCTTCTTAAGATGCAACAGGGGCTTCAGGTTATGGTCACTAAAAGAAGATATTGAAATCCGCTCGAGACTGATTCTTTCTAGCCTTGACAAACAATTGATTAATTGATAATCACTTGTCAATTCAGTTGGAAAGGACCTGTAATTCCTGACTATCAGCACTTTCAATCTGTTCATTTTCTTCGCGAACTCAGGTAACTTGTAAGTTTCTGACCCAGGATTTAGAATTAACACCTCAACTTCAGGTGCTTCCATATCAGGCCAATATGATGAGAACTTCTCATCTGCACCATCAATATCTCATCCAGTGTTAGATTGTACAACGACATAATGAAACGACATATCAAAACATGAGAGAGAGAGACCACGGAGGGACCTGTTGTTATGGACAAAAGGCGGGCTTTCACAGGTTGCGTCTCTTTTTCAGACCACCATTTGGGAAATTCATTTTTGTTAATATCCACAAGCAATCTTGTTCTTTGGTCTACTTGCTGCTCATTAGTCAAATGGACTGCCAATTCTCTAAGTATGTCGTGCTGTGTAATATAGGATTCGCTGTAGTAATCATCTTCATGTGCTTCATTTCTGGAAAATTATCACAACAAGACTAGAACTTCAAGTTCATAACCAAATGGTTATCAAGCACAGTCCACAGTCTTGCTGTTTATATTCAGATACTTATGCATTAAGGTTGAGTTAGTAATTATGATTACTCCAAATATCTTTGAGAGTCAAATCTGAAGTTGATTTAGAGAATGTTTCCCAGAACAAAAGGATAAATCAATAGTAGAATATGTTTCTACTAAGCAATCTTTATTTTCTGTTCATAAAGTAAGTGTTCACACGAGATAATTTGAAATACTTTTTTGGGTGTACATCAATGATCGGTTTATGTTTATAGTTCTTCAAATTAGTCTAAAAAGATAGGAAGAAAATATATATATACGTATCAATTATAGACAAGATTCTTCTGGTAAATAATTGAAAGAAAACCATACCCAAAAGAATCCCTTTGTTTCTTGTACATATATTTTACAACATCATCCACAGAATACTAACGAAACACCAATTTTTTGCATGCGTCATGCATATCAAACTATAATTAAACACAGTTTTTATTTAAAAAAACGTCAATTATGTAGTCAAGAAGCAAGATCAACATAAACCAAAACAACTTGGAAGACCCAGAATTTTACCTCAAAGAGACAGTGTTAACTAAAGTCCGGGTGAAGAGCTCATCGAGGTTTGACATGGTTTCACATTCATCTTGTTCATACAACACTGCACACATGTCAATGAAGGTAGCCGCACGGATTCTTTGATCTTCAGGAAATGAACCCAAGTCCATGAAACACTCCTTGAGCACTATCTTGTCATCTGGGACGGCATCTAAGGTGTCTTTGAGGCACTTCAGAAGCTCATTCTCAGAACCCAGAATAGAATCTCCTCTAGATAATTTCCTCTCCGTAACTTCCCAAACCGAAGTGGCTCTACCCGAAAGTGATCCTGCAATCACTTTCAGAGCAAGTGGGAATCTCTTACAACCCCTCACTATCTGCAATTAGACATTCAAAACTTCAGTTGCATTTGTGTGGAAAAAGTTTTTACGACGTTACTTGAAAGGGATCTTTTCTATAGGTTGAATAACTGTGTCCTTGAGTTCTAAGGTGAGGTAAAAAAAAATTACTTTTTTTAGGGGTCTTTTTAATCAAATTAGGAGATTATCTAATTAGTAATTACCTTTTCTACAATTTTTTCATCTGGGAACTGCAGCATTCTGTTACCCCGCGATGCCCAGCGATGAAACAACTCCTTTGCATCCTTATGGTCCAGAGGTTCCAGATCATACGACTCACCAAATGCAGGAAACTTAAATCTAGAGGTGACCAAAATTTTGCAGTTGGGTAATCGGGAGAACTTTTCAAGAAGCTTGTTTGATTCAGAACCGTCCCAGACATCGTCCAACACAATCAATACAGGATTTGGACTCAATTGCCCCACCAGAAGTTCTAACAACCGGAATGCCTCGTCATCCCTTACAGAATCAGATACTACAGGCCCCCCGAGTCTTTGAATTATAGATTTTAAGATGCGTTTCGTTTCTGGTTTGCTTGAGACAACGAGGAACAAGATGTTTCTCTGAAATTTATCTGTGAAAATGCAGAATCAGAAAGCATGTGTAAGAACTGCAAAGCACTTTTGTAATTGTATTATACATTTTTCAGAAGAATGGTTAAGTTATTTACTTTTAACTTGCTTGTCGTGACAAAATTTTTCGGCCAGAGTGCTTTTTCCACAACCTCCGGGAGCTGTCACTACCAACAATCGAACCCCATCTTTAAATAGTTTAGCCTTCAATTTCTCAACAGGCTTTTCCAACCCAACACTTTCTTCTGTAATCACAGGAACCGTCACAACTAAATCCACCGGATTGCTTAATCCAGATTTGCCATCAAGCCTTAGAACGACGTCCTTGATTTCAGACGCTAACCTCAATGTCTTCTTCCCGTCTCTAGACGTTTGCAACAACACAACGTCCTTGAAACTTCCAATTTTGGTATTCAATTCACGAAGCTTCTCTGTATGACTTGATTTCCTTAACAAATCAACCTTCCCCACTCTTAAACACCGACTAAGTAGCTTTTTAGCATCTTCTAATAAGTTTTTCAACTTCTCCGTTTCTTCTTTTGGATACTCCAGGAATTCATTAAGATCATCTATTTCTTTAACCAGAGGAATTATATCATTTACCTTGGATTCCGTCTCCTTAAGAACAGAATCGAAACTCATCGCCCTTTCGCCAAAATTCTTCAAGATGGTCGCTAGCTCGTTAAACGGAACACCCAATGCCGCGCCACCAATTAAAGCTCCCGCCATTTTCTTTCTCCTGCTATTTCCAAAAGAAAATTCAAACAAAACTCAATTTCTGAAACCTACTTTAACGACCGTAATTAAAACCAGAAACATAATCCAACAAGGCATTTAAGAATCAGATACCTTTATAGCTATCACTCGGAGCTCCGCTTTCGAATGACGTGAAGTTCTCAGCTCGGTTGTCGGAGCTCCGCCGTGGACGGTGACCAATTGGTT

>MELO3C005508

AATGAATCAAAGTTGAAATAGGTGAACCTTCTTCATAGTAATCTTCGAAATTTCAAAATTTTCAATTGGACTTCACTGACCTTTCTGCAGCAATGGCTTCAACGGAAATCTCAACTATTTCCCAAATGATCCAATCGCTATCGGAGCTGCACAAAAATCTCTCCACCGCCCTCCGAAATTACACCGCTGGCGATCAGATCGAGGAGGAGCATCAAACCCCCAAATTCAAGAAATTATTGAACAGCATCGAGCACTTAAAGGAGGCACTGGAGACGACAAAGGAGCTCGATAAGAAGCTCAATGACCCAATTCAGAGCATAAATACCTGTCTCGAGGAGGTTATCAACAGCGTCGAAGCAGCTCAGAGAATCGAAGGGAATTTTCTTGATGCCATAAGTAAGGATCTGAAAACGTTGAAGTTCCGGATCCCTTCTTACCACAAATTTTCGGTTCCAGCCCGTTTGATTGATAGGGGGAGTGACACGCCAGGGCAGAGTGAATTCAAGTTGCCAAATTTGTATGACGATGAGGTGTTTGAGGAAAGTCCTGCTTTTGTAGAAATTCAGGAAATCTATAATGGCTTTACTGATGATCTTTTTAAAAAGTGTTTTTTGTATTTTGCTGTGTTTCCTGACAATGTTGTGTTAAAGAAGCGGTTTCTTACACATTGGTGGATTGGGGAAGGCCTACTTGACTCTTCAGATAATGGGGATGAAACGCCTGAAGTTCTTGCTGGTAATATTCTTAAGGAATTTGCAGAGAAAGGTTTGATTGTGCCAGTGATAGAAGAAGAAAATAAGGTCAAGAGGAGATTTAGAATTCCCCCTCTTGTGCGTTCTGCTGCAATTAAACTGGCCAAACAAAAGGAGTTTTTAGATTATGATACTGGGGACAACCCAACTGGGAAATCTTCCGACTGTGACAGGATTTTTCTAGTGAAGGGGGGAGGCTTTCACCCACCGAAAGCACCGACGAAGGATCGGAAATTGGAAAAAACAATGGAAGTAATCTTCAACGTTAGCCAACCTTTCCCTGATTCTGCATTGGAGTGGTTAGCTAAGGGAGGGGAAGTAGACATGAGAACTGTCAAAGTTGTGGAGTGGTTACGAAAGCTGAGAAACTTAAAAGTTCTTTACTTGGGGAGATGGCAGAGTGCAGTTGATGAGCAGCATATTGAAGTCGAAAACCTTGAATTCTTAAAAGGTTTGAAGAAAATGAAAAAACTAAGGCTTTTGAGCCTCCAAGGGATTTCCTGGATCAATAGGCTTCCAAAGTCCATAAGAACATTGAGTGATCTCAGGGTTTTGGACTTGAAATCTTGTTTCAATCTTGAGAAACTTCCTCATAGCATAGGATCTCTCAAAATGCTTACACATCTAGATGTCTCTGGGTGCTATATGCTCAATGGGATGCCCAAGAGTATATCTGCACTAACTGAATTGAGAGTCCTGAAGGGGTTTGTCACAGGAAATTCAAATCTTAATGATCTAAAAGGCTTAAAGAAGCTGAGAAAGTTAAGCATCAACACAAGCAGACAGGATTTTCCTAATGAAACCGATCGACGTGTTCTCCAAGGACTTGGGGAGCACGGTAAGCTTCGAAACCTGACAATCGCATGGGGGGCAGAAGACGTGAAGCAACAATCTTCGAGTGAATGGAACATTGTACGACAAGTATCCAAGAAGTTAAGCAAACAATTATCCAAGACATTGACCAAACAAATGAATCAGTCTGGCTATGAGATCATAGAATTTCCAAAAGAGCTAGAGAAGCTTGAGATGGAATGTCTTCCAAAGGAAGAGCTACCCCCATGGCTAAGTCCTTCAAAATTGACAAACCTCAAGAGACTCTACATTAGAGGAGGGAAACTAGCAGGGCTCGGGAACGAGACGTGGAATGCCGAGGTTGTTCGTCTGAAATACATGGCAGATCTGAAGATAGATTGGAGAGAACTTCAGAAAATATTTCCAAATTTGAGTTACTTCCAGAGGGTAAAATGTCCAAGAGTTACTTTTTGTCCCTGTGATGCCAATGGAGTTTGGATGAGGCCATTGCAATAG

>MELO3C009693

ATGCAGTATACCATGTTGAGCCTTAAAACAATTCTTATGGATGCTGAAAAGGAAGAATATAGTCATCATCTAAATGATTGGCTACAGGAACTTCAAAATGTATTTTCTCAAATCGAGGGATTGCTATATGAATTCAATGGGAAAGTCAAAAAACAAGAGGCTACTGGAAAATGGGTATTTGTTCTTTCCTTTAACTCCAGTCGAATTGAGCAAACTAAAAAAATGATGAAACTATGCGACGATTTGGATGAAATTGCATCCCAAATGTATGACTTCAATCTAACAAACATGGAGACAACACACTCCTTTCTTAGTGCTACTGAAGTTTCGACGAGACTCATGAAACCAAGTTGGCAATTGCTTTACCCGTTGACTAATGCTCCCAAAGTTTTCCATGACAAACGATATAGTAACTTTCTGGATCATTTCAAAAAATCTACTCTCGGATTCTTCCACATAGTTGGAGAGCCAGGTATAGGTAAGACCACACTTGCCAAATTCTTTTACAACGATCCAGAAGTGGTGAAAACGTTTCCATCAAGATTGTGGATTTGCGTGAAAGAGGAATTTGATCCACGGAGATTGATAAAAGAGATGCTTGATTTTTCACATTGTCAAGCAACATATGATAACTTGACTGAGAAACAATTGTGCTTTGCAGTTCAACAATTTCTGAGGGATAAAACATTTCTGCTTGTTTTTCAAGATATTTCAATCAAGAACCTAGGTGATTGTTCCATATTTAAAAGCTTATTGGGGATGGGAAACCCTGGCAGCAAAATCATAGTGACCACTCAGAATGAGGAAATTGCTGAAGCTATCAGGCTAGGAAAACTCTGCAAGAACGAGAGCCAGGAAGTTCCGTCCCCAGAAGCCACAGAACCCTCAGATGTTAACAATGACGAAGCATACAAGGATAATATGACATATCAAAAAATTTTTGAAGTTGAGAGGTTGTCAAAGGAAAATTCATTGTCTTTATTCAAAGTTCATGCTTTCACAGAAACACAGGAAGCACAAATCCCAAATCTCACAAAAATACAAGAAGTAATTGAGCAGAAATGTCACGGGGTTCCTTTGGCAATAAAGTGCCTGGGGGGTCTGCTATCGAAAACTAGTATAGCTGAGTGGAATGGTGTCATCGACAAGTTATGGGAACATGAAGAAGGGGAGGATGGGAATAAGAGTATTTTACCTGTGCTTAGATTATGCTATGATCGAATGCCTTCACACCTACAACGTTGTTTTCTTTATTGTTCCCAATTAACAAAAGATCGCATATTGTCTTCGAATGATGTGATTCAATTATGGATCGCAAGCGACCTCCTACCCAAAGAGAATTACTTATCTTTGGAAAAAATAGGAGAGAATTATTTCAAGGAACTATGCTCAAGATGTTTCCTACAAGAACTTGAGGAATATGGTCTTGGCTATTGGTTTAAATTGCACCCTCTCATTGAAAAACTTGCACGTATACTCACACAAAAACAGGTCTTCCCAGTCGCAAACACCAAATGTATAGCCTTCACAGTAAGAGATAAGGTGCCCCCTAGTGCATTCCTAGCCAATACATGCATCGACAAGTTCAAATACATAAGACTACTGTATTTAGGCAATGCAAACCTACGGGAAATTCCAAATGCTGTAGAAAAACTGGTACAGCTCAGATACCTAGACTTGCAAGGGAATAAGAGACTCAAGCGGCTTCCAAATTCAATCTTTAATCTAAAAAATTTACAAACCTTGATTCTTGCATCTTGTTCCGCACTTGAAGAACTGCCCAACCATATTAAGCAATTGATCAACCTGAGATACCTCTGGGTAACAGCAAACAACCTCCGTCTGCACAAAAATGGAGTTGGAACCATGACTTCTCTTCGATTTCTCGCAATTGGAGGGTGCGAAAACCTACAAGATCTATTCGAACAGCCCTCATGCCTCGTACGCCTAGAAACCCTAATGATTTACGATTCCAAAACATTGAAATTGTTGCCAAACGAGATAGGAGCGCTTATATCACTAAAGAATTTGGTGATTTGGAGTTGCAAACAACTTACACTGACGTTGAAAGGAGTCGAATTCAGGCTTCGAAGATTCACAATCAGAGAGCTTCCAAGAGTGAGAAAGTTGCCGGAATGGATTCAAAGATTCACCGAAAGTCTAAGAGTTTTGGAAATCATCGATTGTCCCATCGAATTGAAGGATGATGAGTTCAAATCATACAAATCACTTGAACGGTTATCAATTCATGGAGCTGTGAGGATCAAAAATCTTAACGGGGAATACTGCATCGATTTTCAAAATTCCGTTAGGCGTAGGGAAGTGACGAAGGAAATGAAGACATGTTACTATTACTAA

>MELO3C009695

ATGTCGAGCCTCAAAGCAATTCTTTTGGATGCTGAAGAGAAGCAAGAACAAAATCAACGTCTACATGATTGGTTAAAGGAACTTCAAAATGTCTTTTACCAAGTTGAGGACTCCATAGATGAATTCAAATGGGAATTCTTCAAACAAAAGGACACTGGAAAACAGGTACTTGCCCATTTCTCGTGCTCTAGTCGAATTTCTGCAAATAAATTGAAACAGAATTTTAAAAGGAACAAAGTATGCGACGAGCTCAATAAAATTGCAGCCAACATGTATGAATTTCATCTCAAAGTAAAGCACATCGGTTCCATAAGTATGGAGACAACACACTCTTTCCCTAGTGCTTCAGAAATTTCAACAAGACACCTGAAACCAAGTTGGCAATTGCTTTACCCTTTAAAACATGCTTCCAGAAATTATGACGAAGCATATGATGGCATTTTGAATGTTTTCAATAAATATACTCATGGGTTCTTCCACATAGTTGGGGAAGCAGGTATAGGTAAGAGCACAATGGCCAGATTCTTGTACAATCATGAAAATGTAGTTGATAGGTATACTAGAAGATACTGGGTTTGTGTGGAAGAAGGCTTTAATACACATAGATTGATGAAAGAGATTTACAGTCATGCAGACAATAAAGAAACTCGGGAGGACTTGACGACAGAACAATTGCTTTCTAAGTTTATACGACTTCAGAGAGAGGAAACCTTTTTGCTCGTCTTTCAAGACCTTTCAATCACCAACTTGGATAAGTGTTCCACTTCAGTGTTAATTAAATTATTGGAGATGGGACACCATGACAGCAAGATCATAGTGACCACACAAACTGAGGAAATTGCCAAAGATATAGATGTCCATGGCTACAAGACTGAGATACAATCAAAGAAAAATCTGGCGAATGGTGACGAAGTATCTGCAGACAAACAGTCATTAACAAAAAATACTCAGAACCAAGCAGTTCCAGGTTCACAAACTGAGGAAACTGCAGGCGCTATAGCAAACATGATCTATGAGACTGAGAAAAACTTAGAGAAAAATCCGGTGGCTGGTGACGGAGAATCTGAAAGCAGCCGATTGATAACAAAAGATGCTCAGAATCTAGACGTTTCAATTCCACAAGTTACAGAATGTGAACCTGATAGGGGATATCAAACAATTTTCAAACTTAAGAAGCTGTCAAAGCAAAGTTCATCTCTTTTATTCAAAGAATATGCTTTCAGAAACAGTCCAGAAGTACAAAATCCAGAACTCACTAAAATAGTTGATCAACTTTTGGAGAAATGCATGGGAGTTCCTTTGGCAATAAAGTGTCTGGGAAGCTTGCTATCTTCAAAAACTAGCATAGCTGAGTGGAAAAACATCGAGAAAAAGTTGATGCCCCAAGAGAAAAAGGAAGACGGTATTTTACATGTACTCAGAGTTTGCTATGATCAAATGCCCTCACAATTGAAGCCTTGTTTCCTGTATTGTTCTCAATTACCAAACGATCGCATATTTTCTTCAAATGATATGATTCAGTTATGGATGGCAAATGGGCTCCTCCATTCACCTGAAGAGAAGAACTTAGCTATGGAAAATATAGGTAAGAAGTACTTCATGGAGCTATGGTCAAGATGTTTCATTCAAGAAATTGAAGAACATGGGCTTGGCTACTGGATTAAATTGCACCCTCTCATCCAAAAACTTGCACACAAAATCACACAAGAACAATCTGAGGGCTCGTGGGGCAACAATCATTCCAAAGAAATCACTGAAATAAGATCCATAGCCTTTCAAGAAAGAAATATGGTGCTACCTAATGCATCCCTAACTGAAAAGTGCATCTGGAGGTACAAATGCTTAAGATTGTTGTATTTAGGCAATGCAGACCTTCAGGAAATTCCAAATTCCATAGGAACACTCAAGTACCTGAGATACCTCGACTTGCATGGCAATCAGAAAATCAAGCATCTACCAGACTCAATATGTGATCTACAAAGTTTGCAAACCTTAATTCTTGATTCTTGTTCCGCACTTGAAGACCTGCCGAAGGATATAAGGAATTTGATCAGCCTGAGATACTTGTGGGTAACAACAAACAAGCTTCATCTGCACAAAAACGGAGTTGGAACCATGACCTCTCTGCGATTTCTTGCCATTGGAGGGTGCAAGAACCTAGAAAATCTATTTGAACGGCCAGATTGCCTTGCAGGCCTTGAAACCCTAATGATATATAATTGCAATACCTTGAAATTGTTGCCAAACGAGATGAGGTATCTAAAATCACTACAAAATTTGATGATTTGGAGATGCAAGCAACTTACACTAAACTTAGAAGAAGTCGAATTCAAGCTTCAAAGATTCACGGTCAAGGAGCTTCCAAAAGTGGAAAGCTTACCTCGATGGCTTGAAAATTCGATAGAGACTTTGAGAACCTTGCAGATCATCAATTGTCCCATAAGATTAATGGAACTACCGGTAGATAAAAAGTATAGAGCACTGGAAATTTTCTTAATTCATGGTTCGGTAAGGTTTGATACAATGCCAGATTACGACTTTGAACACCGGAATTTGACAATCTTTCGTGGTAATGAGATGAATATACATTATTAG

>MELO3C016529

ATGAACCGTGTGGCGGTCGGCGGTGTCGGGAACATTACGCCGAGGCAGTTTCTAGCTCATATGGGGAAAAAGGAGATGAAGGGTATGATTCTGAAGCCTTGTGATGTGTTCATAAACCATAGAGGGGTCGACACAAAGAAGACGGTGGCGGCATTGTTGTACGACCGGCTGGTTCGGGTGAGGCTGCGGCCGTTTTTGGATTACAAGAATTTGAGGCCTGGAGAGAAACTGTTTGATGAAATTCATGGGGCTATTAGGCAATGTAAAGTTGGTGTAGCTGTGTTTTCTCCTCGCTATTGTGAGTCTTATTTTTGCCTTCATGAGTTGGCTATGATCATCGAGTCCAACAAGAAGGTTATTCCTATATTTTGTGATATAAAAGCATCGCAACTTCGCATCGAGGATCATCATGGTTATGAAACTGACGAGGTGAGGAGGTTCAATTGGGCTCTGCAACAAGCTAAAAGCTTCGATGGACTCAAATTTGATTCGTCAACAGAGTAA

>MELO3C017692

ATGGCTGAGTTCCTATGGACTTTTGCTGTCCAAGAAGTGTTGAAGAAGGTATTGACTCTTGCAGCTGACAAAATTGGTTTGGCATGGGGCTTGGAAAAGGAGCTTTCAGAGCTCTCCCAATGGCTACTCAAATCAGAAGCTATTTTAGGTGACATTAACAGGAAAAAACTACACCCTAGTTCTGTGAGACTGTGGGTGGCAGATCTTCAACTTGTTGTTCATGAAGCGGACGATCTATTGGATGAGCTTGTTTATGAACATCTTCGTACGAAGGTGGAAAAAGGATTGATTAACAAGGTATGTTATTCCGTGTCAAGTGGCTCTAATATTTTCATTATCTTTCGCTTCAAAATGGTCAAAAAAGTTAGGATTATTATTGAAAAGTTGCGTAAATGTTACTTCGAGGCGGCTCCTTTAGGACTTGTTGGTGAAGAATTCATAGAAACAGAGAATGATCTCGGTCAAATTCGAGAGACAATATCGAAACTTGACGATTTTGAAGTTGTTGGAAGGGAGTTTGAAGTTTCAAGCATAGTGAAACAAATGGTTGATGCTAGTGATCGATATGTTACAACTATCTTACCCATTGTGGGTATGGGTGGAATCGGAAAAACAACTTTGGCAAAGACAATCTTCCATCACGAGGAGATCAGAGGACATTTTGACGAAACGATATGGATATGTGTGTCCGAACCATTTCTTATCAACAAAATTTTGGGAGCAATTTTACAAATGATAAAGGGTGTTTCTAGTGGCTTGGATAATAAAGAGGCTTTACTTCGAGAGCTTCAAAAGGTGATGCGAGGTAAAAGATATTTTCTTGTACTTGATGATGTTTGGAATGAAAATCTTGCTTTATGGACTGAATTGAAAAATTGTTTACTGAGTTTCACTGAAAAATCTGAAAACGGTATTATTGTGACTACTAGAAGTGTTGAAGTTGGAAAGATTATGGAGTGTACTCTTTCTAGCCATCATTTGGGAAAATTATCTGATGAACAATGTTGGTCTTTGTTTAAAAAAAGTGCAAATGCAAATGAATTGCCAATGAATTTAGAGTTGAAGGATATTCAAGAAGAGTTGGTGAAAAGATTTGGTGGTGTACCATTGGTTGCAAGAGTTTTGGGAGGGGCACTGAAATTTGAAGGGGTCTATGAGAAATGGGTGATGTCTCTTAGAACCACAACAAGTATACCATTACAAGATGAAGATTTAGTTTTATCCACATTAAAATTAAGTGTAGATCGTCTACCATCCTTCTCGTTGAAGCGATGCTTTGCATATTGTTCAAATTTTTCTAAAGGTTTTAAATTTAGAAAAGAAGAGCTAATTCAAATGTGGATGGCACAAGGGTTCATTCAACTACATGAAGGAATAAACAACATAACGATGGAGGAAAATGGAGAAAAATACTTCAACATCTTGTTGTCTCGCTCTCTATTTCAAGATATCATCAAGGATGATAGAGGAAGAATTACTCATTGTAAGATGCATGATCTTATCTATGAAATTGCATGTATCATTTCAAATTCTCAAAAGTTGCAACAGGAACAAATTGATTTGTTGGATAAAGGAAGTCGCACCAATCATAGGATAAACAACGCCCAAAATTTACGCACACTCATTTGCAATAGACAGATGCTTCACAAGACTATTTATGGGAAGATTGCTAATTGTACTCGCTTGCGAGTTTTAGTCGTGGATTCATCTATTACAAAATTGCCAGAGTCGATTGGTAAGATGAAACATTTGAGATATCTCGACATTTCAAATTCAAAGATAGAGGAACTTCCAAATTCTATCTCTTTGCTTCATAACTTACAAACACTGAAGTTTGGAAGCTCAATGAAACACCTTCCACAGAATTTGAGCAAGTTGGCTAATTTAAGACATCTAAAGTTCTCAATACCACAAACGCCTCCACATTTGAGCCGATTGACTCAACTTCAAACATTGTCTGGTTTCGCAGTTGGATTCGAGAAGGGTTGCAAAATAGGAGAACTTGGATTTTTGAAAGACCTCAAAGGTAGATTAGAACTTTCAAATCTCGATCGAATTGAAAATAAAGAGGAAGCCATGAGTTCCAAATTGGTAGAAAAGAACTTATGTGAGCTATTGTTGGAATGGGATTTGCATATTTTAAGAGAATGTAGCAGCTACAATGACTTAGAAGTGTTGGAAGGGCTTCAACCACACAAAAATCTACAATTCTTGAGTATCATAAACTTTGCTGGCCAACTTTTGCCTGCTGCCATTTTTGTTGAAAATTTAGCTGTGATACATCTAAGACATTGTGTCAGATGTGAAGCACTTCCAATGCTTGGACAATTACCTAATTTAGAGGAACTAAATATTTCCAACTTACTTTGTCTAAGAAGTATTGGGAATGAATTCTATGGAAATTATGATCATCCCAACAACAACCATAAGGTTTTATTTCCCAAGTTGAAGAAATTTGTACTCTCTCACATGCACAATCTAGAGCAATGGGAAGGATTAGTATTCACATCAAAGAAAGACGCAATTTTTCCTCTTCTTGAAGACTTGAATATTCGTCATTGTCCTATATTAACAAGTATTCCAAATATTTTTAGATGTCCTCTTAAAAAGCTACATATTTATGGATGTGATGAAGTGACAAGATTGCCAAAAGATCTACAACTGTGCACTTCCATTGAGGATCTAAAGATTGTTGGGTGCCTTAAATTGATACTAAATGTGCAAAATATGCACGGTTTGTCTCGTTTCTCTATAAATGGGTTGCAAAAGTTTCCCCAAGGGCTGGCTAATCTCAAAAACTTGAAAGAAATGACGATCATTGAATGCTCACAAGATTGTGACTTTAGTCCTCTGATGCAACTTTCTTCACTTGTAAAGCTTCATTTGGTTATTTTCCCAGGGCGCGCGACCGAGCAACTTCCTCAACAACTTGAGCATCTCATTGCCTTAAGATCTTTGTATATCAATGATTTTGATGGAATTGAGGTTCTACCGGAATGGTTGGGAAACTTTACATCTTTGGAAGCTTTGGGACTTTGTAATTGTAGAAATTTGGAACAGTTTCCTTCAAAGAAAGCCATGCAATGTCTCACCCAATTAGTCCGCGTGGATGTTCTTGGATGTCCACAACTGTCCGAGTTCGCAGATTTTGTCCCATGA

>MELO3C022157

ATGGAAGCAATTGAGGAATCAAGAACAGCTATTGTGGTTTTATCACAAAACTATTCTACTTCAAGATGGTGCTTGAGAGAATTGGAGAAGATTATGGAATCCATGGACGACGGAACAAATCGAGTTCTTCCTGTGTTTTACCATGTAGATCCTTCTCATGTTCGTCATCAATCTGGACCTTTTGAGAGAAGCTTTGTTGAATATGAAAATAATGGACAAGACTCACAAGAGCAGGTTCATCGGTGGAGGGATGCTTTCGCTAGAGTTGGCCATCTTGCAGGGGTCGTAGTAAACAAAAACAGCCCTGAAGTGGACAGTATCAACCGAATCACCAATCAAATATTTGATAAGTTGCGAAGACCTATGTTAATAGGCCCTAATCAATTGAATTACTTGGTTGATATGCGAAGTAAGCTAAGGGATATCAATAACCTACTTGACTTTGAATCAGATGAAGTACGATTTATAGGAATAGTTGGAATGGGTGGTATTGGTAAAACAACTATTGCAAAAGTTTTACACGACAGTATTGCATTTACATTATTTGGTGAAAATTCTTGCTTTGTCACTATGTCTGGGCGTGATATCGTCACGGTCCAATGTCTACTACTCTCTCGACTTCTTGGAACTAGGGAGAATATTAACATTTTAGAAAAGAATGAAGGAGCAAACATGATTAAAGATTGTTTGAGTAGGAGAAAGGTTTTGATTATTTTTGATGGGGTGAATGATAGAGAGGAATTAGGATACATAGCCGGAAGTTTTGATTGGTTTGGTCGAGGAAGTCGAGTCATCATTACCACTAGAAATAAAAATGTTTTTTCTCACCCCAATCATGAACAAGTTCAACTCTACAATGTGAAACCACTTGATTACAACACTTCATTCTCACTTTTTTGGAAGCATGCATTTGATCAACAAAGTGGGGGTCCAAGTGAACAACAATTCATACAACTTAGTCAGAATATAGTGGAAAAGGTCGAAGGAAATCCACAAGCATTGGAACAAATTGGATCATTTTTGCGTGGTAAAGATATTAATGTATGGAAAGAAGAATTGAAGAGCCTTGTTTTAGTTGATAATGAACGTCTCTTCAAAATATTAAAGATAAGTTTTGATCAATTAGGGACAAAAGGCCAACAAGCTTTTCTTGATTTGGCATGTTTCTTCAATGGAAAAAGTACAGGCAAAATTATTGAAATACTTGCGAGTTTAGAATACAATTCCCCCAGCGAAGTACTAAAGTTGTTGTGTGATAGATATCTTATTGAAATTAGAGATGGAGACACAGTATGTATGCCCAATTTGATACAAGAAATGGGTCGAGAAATAGAACGAAAAAAACGTCAAAGAAGCAGGATTTGGCTTAGAAGAGATGCTTTCGACATATTTGATGAACAACATGGAGTAAAAGACATAAAAGGTGTTGTGTTGGACAAGAGAGACACAGAACCAAACTTAAAGTTGAAGGCTAAACAATTACAAGATATGAGCCGTTTAAAAATATTAGAGATTGACAATGTGCAGCTGAGTCCAAGAAATCAAAATGATCTCTCAAATCAGCTTCGATTGCTCCACTGGGATGGCTTTCCTTCAGACACTTTGCCACTAAATTTCGAAGCACCATATTTATTTGAACTTCTCTTGCCTAATGCACAAACCACTCATCTTTGGAAAGAACTAAAGGGATTTAAGAAATTAAAGGTAATCGATGTTAGCAATTCCCAAACTTTGGTGGAGACACCGAATTTGAGTGCTGTTCCAAATCTAGAAAGATTGATTCTATGTAATTGTACAAGATTGAAGAAAATTGATAATTCAATTACAAAATTGAGACTTCTAGTTTTAGTAGACCTCACAGGCTGTGTTCGCCTCGAAACATCCGAGTGCATCGATATCTTGAAGAGTCGCCCAACAGTAGAACTTCGTGGCTTAGTTCTACAGTGTCGCCAATCATTGAAAGGTATTTGTTATATTCGTAAATTTTTTCACTTCTTTTTTAAAATTCAGGATCATTTTGTGTAA

>MELO3C022580

AAACAGAGGCTGCTCTTAATCAGATTCCGGAGACAACCTCAATTCTTGACTTTGAAGTTGAAGGAAGGGAAGCTGAAGTTTTGGAGATACTAAAATTGGTGATCGACTCTAGCGATGAAGATCATACGTCTGTGCTATCCATTGTTGGAATGGGTGGTCTTGGCAAAACAACTTTGGCCAAGATGGTTTTCAATCATGATGCCACTGAATGGGTTTGTGTATCTAAACCATTTATTGTCGTGAAAATTTTGGAAGCAATCTTTGAAGGTTTAACGAATACTAGTAGTGGTTTGAACTCTAGGGAAGCCTTGCTTAATAGACTCCCAGAGGAGATGCGCGAGGAAAAAAGTATTTTCTTGTACTTGACGATGTTTGGGATTAAGAGAATGGCTTGTGGGAGGAGAGCTTATTGGCAATTTGAAATATATTGCTGGAAAATCTGGAAATAG

>MELO3C023577

ATGGATGATGTGTGGAATGAAAGCCATGAGAAGTGGATTGATCTAAAAAGATATCTAATGGGTGGTGCAATGGGAAGTAGAATTTTGATCACAACTCGTAGCCAACAAGTTGCACAGACTTCTGACACAGATTCATTTCACCATTTAAAAGAACTCGACAATCACAACTCTTGGGTGTTGTTTAGAAAAATGGCATTCTTGAACGAAGAAGAAGAGATTGAGAATTTAAATTTGGTCAAAATCGGAAAGGAGATTGTAGCAAAGTTGAAAGGTTCTCCTCTTGGAATAAGAGTAGTTGGCCGTTTGCTATATTTCCAAAACACAGAAAAGGATTGGTTGTCATTCAAGGACCATAATGAACTTGGCACAACTTTACAACAAGAAAATCAAATTCAACAAATACTGAAGATTAGTTTTGACCACCTTCCATCTAACTTAAAGCGATGTTTTATGTATTGTTCTTTGTTTCCTAAAGATTATGAGTTTCGAAAGGATGAATTGGTAAAACTATGGATGGCACAAGGTTTGATCATTCAACCACGTAATGAAAAAGCAATTGAAGATGTTGGGGATGATTATTTTAAAGAGTTATTGGGGAGGTCGTTCTTTCAAGACATAAGAAAAAATAAATGGGGAGACATCAAGAAGTGCAAGATGCATGATTTGATACACGATCTTGCGCTTTTGATGGTAGAAAATGAATGTGTGCTTGTAAATGATGGTGTTGGGTCCATTGATAAAAGGACTCGACATGTCTCATTTCTCTACAACCGAAGAAGGCCAATATCGAGGGAACTTGTACCTAGATCGTTCACTCAGGCAAAGAAGTTGAGAACATTGGATTTGACTTTTAATTCTATTTCCTCTCTGAAAAAGTATCATATTAACCTTTTTCGATTACGAACATTGAATTTGGATAGATGTTGTTGTCATCCTCCTAAGTTTATTGATAAGTTGAAACATTTGAGATATCTTAATCTTTCTGATTTGGATGTAAGGTTCCTTCCAACATTTATTACCAAATTGTATAATTTGGAAACACTTATCCTTCGATACTGCAGATGGCTAAGAGAATTGCCAAAAGATATTAGCAATTTGATGAATCTTAGGCATCTTGATCTACAAGGATGCTCCCGTTTGACTCACATGCCAAAAGGGCTAGGTGGGATGAGTAGCCTTCAGACAATGAATTTGTTTGTATTAGGAAAAGATAAAGGTGGCAATTTAAGTGAATTGAATGAACTTAAAAGCTTGAGAGGATCATTATGTGTTCGAGGATTACAATTTTGCACAACTATTGATATAGAAAATGTGAAATATTTTGAAGAAAAGTCTGAAATTCGAAAGTTGGAATTACATTGGGACACAGATAAGAAGAAGCCGAGAATTGATGATGCCTCGTATGCTGAAGATGAGAGGACTTTGGAGTGCTTAAAACCACATTCAAATGTTGGCAAAATGAGTATAAAAGGATATAGAGGCATGAAGTTATGTGATTGGGTATCTTCTGATTATTTCCTGGATGGTCTGGTTAGCATAGAACTTTGTCATTGTGAAAAATTGGAGCATCTCCCTCAATTTGATCAATTTCCATATCTCAAGAATCTTCATCTTGAGGACTTAACGAATATCGAATACATTGATGATAGCAATTCTGTTTCTTCATCAACAACTTTTTTTCCATCTCTTGAGAAACTAAGGATTAAGAAAATGCCTAAGTTGAAAGGGTGGTGGAGGAGGGGGGAAATCCCATCGAATTACTCTGCTCAATACAATGCCTCTCTTCCAACAGCATTACATCAGCTTTCACAATTATGGATTTTGGATTGTCCTCAGCTGGCTTTTATTCCACAGCATCCACCTTTGCAATCATTGGCAATAGGGGGTGTTGGTTTGAAAGTTTTTGATATGGTAATAAGAATGGCTACAAACCTTGCTGCGGATTCTTCTTCTTCTTCAACTCTGTCTAAATTATCTACTCTTGAGATTGAAAATATTGATATCAAGTTCCTGTCAGAGGCGTTAAACTGCAATATGAAAGATCTTGAGTCTCTTACCATACAAAGTTGCAAATATTTACAAATGTCTTCTTCCCATCTCGTGTATGAGGAAGATGATAGGCTATTGTACTGGAAAGAACTTAGCAATCTCCGCAAGCTTCGTTTTTGGGACATTCCCAAATTGGAGTATTTGCCAAAGGGTTTGGAACATTTGACAGCTCTTGAATTTTTGCTTCTAATAGGTTGTGAAAATTTAGTGAGTATTGAAGGGATCGGCCAACTCAGTTCACTATCAGGTTTGCGTATTCTTGACTGTCCCAAATTACGTTTGTTGCCAGAAGAGATCGGCCACCTCGTTTCCCTATCACACTTGTCGATTTTGAAATGTCACAATTTAACTTCATTGCCGGAAGGAGTATGCGGCCTGACTTCATTGTCCTATTTGGCGATTTATGATTGTCCTAATTTAAGGACGTTGCCGGAAGGACTTAGCCAAATCCGCTCCTTAAAAGGATCATTATATGTGTTAAAATGCCCCAAATTGAGGAAGAGTTGGAAGAAGCAAAACAAATACAGAAGGTTGAAGAAGATGTTTAACTTGGACAACAAACAAACAGGTGAAGATGATCGCACTGAAATCACCTTTGTCAAGACTCGACGTAGCCCTGACAGCTTATACCGTCATTACAAACATAAATTACATTATTGGTTTTAAAAGCTGCAAAAACC

>MELO3C023580

ACCTCACTTGTTTATCAACCTAATCTGAGTTACCACCCTTCCTCCAAAATTTTGCTCCTTTTTCATCAACCATGGCTGAAGCTATTCTCTACAACGTTACTGCAGACATCATATTCAAATTGGGCTCTTCCGCACTCCAAGAGCTTGGGTTGTTGTCGGGTGTCAATGATGAGCTCGACAAACTCAAACACTCTCTTTCTGCCATTCAAGCCGTGGTTCTAGATGCGGAGAAGCAGCAGTCCAAGAGGCATGCTGTCAAGGCTTGGGTTTCAAGGCTTAAGGATGTTTTGTACGAGATTGATGACCTGGTGGACGAGTCCTCTTACGAAACCTTAAGAAGGCAGGTTTTGGCCAAAGAACAGAGAAACAGAAAACTAGTACGTATCCTCTTTTCCAAATTTAAATCTAATTGGAAAATAGATCACAAAATCAAGGATATTAGGCAGAGGCTACAATCTATTAATGATGACAAAAATCAATTTAGCTTTTCTGAGCACGTGATCGAGAAAAGAGATGATGAAGAGTTTAGAAAGAGACGGGAGACTTACTCTTACATACTTGAAGAGGAAGTGATCGGTAGGAATGATGACAAGGAAGCAGTTTTAGATCTTCTATTAAATTCCAACATCACAGAGGATATTGCAATTGTTTCCATTGTTGGAATGGGAGGACTGGGAAAGACTGCCTTGCTCAATCTATTTATACCCATCACGATATGA

>MELO3C024743

ATGGGTGATTTCCTATGGACTTTTGCTGTGGAAGAAATGTTGAAGAAGGTGTTGAAGAAACATCTCTACAAGCTCCAAAAATGGCTACTCAAGGCTGAAGCTTTCTTACGCGATATCAACACGAGAAAATTGCATCATGATTCTGTGAGGATGTGGGTAGACGATCTTCGACATCTTGTTTATCAAGCCGATGATCTATTAGACGAAATTGTTTATGAACATCTTCGACAAAAGGTTCATACAAGGAAAATGAAGAAGGTATGCGATTTTTTTTCTCCTTCAAGCAATGCTTTCATCTTTCGTCGTAACATGGCGAAAAAAATGATGACTCTTGTAGAATTGTTAGAAAAGCATTACAATGAGGCTGCTCCTTTAGGACTAGTGGTGAATGAAAATGCAAGACCAGAGATCGATGTTATTAGTCAATATCGAGAAACAATTTCAGAACTCGAAGATCATAAGATTGTGGGGAGGGATGTTGAAGTTGAAAGTATAGTGAAACAAGTGATTGATGCTAGCAATAATCAACTTACATCTATCCTACCCATTGTTGGTATGGGTGGATTAGGAAAAACAACTTTGGCAAAGTTAGTTTTCAAACATGAGTTGGTTAGACAACATTTTGATAAAACTGTATGGGTATGTGTCTCTGAACCATTTATTGTCAACAAGATTTTGTTAGATATTTTACAAAATCTAAAAGGTGAAGCATTTCTAATGGAGGGGATAGTAAGGAGGTTTTTCTTCTTGAACTCCAAAAAGAGATGCTTGGCCAAACATATTTTCTTGTGCTTGACGATGTTTGGAACGAAAATTCTTTTCTATGGGATGAGTTGA

>MELO3C025517

ATGGCTTCTCCATTAATCATTGAGAGTAGAGTCTCTTCAATAGCATCCTTATCTTCTCCTCCGCCTCCTTATTCTCTCTCCTTCCCTCTTCCTCCCTTACGAAACTATGACGTTTTCCTCAGCCACAGAGCTAAGGACACTGGATGTAGTTTCGCGGCCGATCTCCATAAAGCTTTGACATCTCAAGGAATTGTAGTTTACAGAGACCACGAAAACGAAGAAGGGAGAGGGAAACCGTTAGTGGAGAAGATGAAAGCGGTGGAAGAATCGAGGTGTTCGATCGTGATTTTTTCAGAGAACTATGGGAATTTGGTTTGCATGAAGGAAATAGAGAAGATAGTAATGTGTAAAGAGTTGATGGATCAATTGGTTCTTCCCATATTTTACAAAATAGATCCAACCAATGTGAGGAAGCAAAAGGGGAACTTTGAGAAGCATTTTAATGAACATGAAGCAAATCATGAGATTGATATTGAAGAAGTTGAAAGCTGGAGGTATTCAATGAAACAAGTTGGCCATCTCTCTGGATGGCATATCCAAGATTCTCAGTTAAGTAAATTATTCACTCTCTGCACTTTGTTTTCACTCTCTCCGGCTTCATATTATTTTTTTAGAAAATTCTCTCTCCTTTTCCTGTAG

>MELO3C027385

ATGGCAGGTGAAACTGTGGAAGCAAGTGATTTGAAGCCTATAGCCATTCAAATTGCGAGAGAATGTGCAGGTTTGCCTATTGCTATTACTACTGTTGCTAAGGCATTACGAAATAAACCTTCCGACATTTGGAATGATGCCTTAGATCAGCTTAAAAGTGTTGATGTGGGTATGGCAAACATTGGAGAAATGGAAAAGAAAGTGTATTTGTCACTAAAACTGAGTTACGATTGCTTGGGATATGAAGAGGTGAAGTTATTATTCTTGTTATGCAGCATGTTTCCAGAAGACTGTAGCATTGACGTGGAAGGGTTGCATGTATATGCCATGGGCATGGGATTCTTACATGGTGTTGATACTGTGGTAAAAGGACGACGTAGGATAAAAAAATTGGTTGATGATCTTATATCTTCTTCTTTGCTTCAACAATATTCTGAGTATGGGTGCAATTATGTGAAAATGCATGATATGGTTCGTGATGTAGCCCTATTAATTGCATCTAAGAATGAACACGTACGTACATTGAGCTATGTGAAAAGATCGAATGAAGAATGGGAAGAAGAGAAACTATTGGGTAATCATACTGCAGTGTTCATTGATGGTTTACATTATCCTCTCCCGAAGTTAACGTTACCCAAAGTTCAATTATTAACGTTAGTTGGACAATCTTGTTGGGAACATAATAAGCGTGTGTCGGTGGTAGAAACTTTTTTTGAAGAAATGAAAGAGCTCAAAGGTTTAGTATTAGAAAACGTAAATATATCATTGATGCAACGAACATCTGATCTTTACTCCTTAGCAAACATCAGAGTATTACGTTTGCAAAGATGTCAATTATTAGGGAGCATAGATTGGATTGGTGAATTAAAAAAGCTTGAAATTCTTGATTTTAGAGGATCTAACATCTCACAAATTCCTACAACCATGAGCCAATTGACACAGCTGAAAGTTTTGAATTTATCTTTTTGTGAACAACTCGAGGTAATTCCACCAAATATTCTTTCAAAGTTGACAAAACTGGAAGAATTAAGTCTGGAAACTTTTGATAGATGGGAAGGAGAAGAATGGTATGAAGGAAGGGAAAATGCTAGCCTTTCTGAACTCAAGTGCTTGCCACACCTTTATGCTTTAGAATTAACCATTCAAGATGAAGAAATTATGCCAAAAGACTTGTTTTTAGTTGGGGAGTTGAATCTTGAAAAATTCAACATTTGTATTGGTTGCCAAAGCAAATTAAAGTATACTTTTGCATACCAGAACAAGAACAGAATCAAAAACTTCATTGGAATCAAGATGGAATCAGGAAGGTGCTTGGATGATTGGATAAAAAATTTGTTAAAGAGGTCGGACAATGTGCTTTTGGAAGGATCAGTTTGTTCAAAGGTTCTCCACTCAGAATTG

>MELO3C027424

ATGGATCGTTCAAGTGGATCATTTTCTTCGCATTTTAGATGGAGCTTCGATGTATTCTTAAGTTTTCGGGGGGAAGATACTCGTTTCAACTTCACGAGTCATCTTTATACGGCTTTACGTCAAAGAGGAATCAATGTTTTCATAGATAACAAGCTTACAAGAGGTGATAAATTTCCTCCATCTCTTTTGAAAGCTATCGAAGAATCGAAGATCTCGATTGTTATAATCTCTGAAAATTATGCATCTTCGAGCTGGTGTTTGAATGAACTGGTGCACATCAATATGTGTAACGAATTGAGAGGACAAGTTGTTTTACCAGTTTTTTACAAAGTGAATCCATCTCAAGTACGGAAACAAGATGAAGCATTTGCGGAACTTGAA
