# Supplementary material for: Frequent loss of lineages and deficient duplications accounted for low copy number of disease resistance genes in Cucurbitaceae
Source: BMC Genomics. 2013 May 17;14:335. doi: 10.1186/1471-2164-14-335 (PMC3679737; doi:10.1186/1471-2164-14-335)
Supplement: Additional file 4 — Gene structure model in Cucurbitaceae. [file 1471-2164-14-335-S4.docx]

Additional file 4:

Gene structure model in Cucurbitaceae (Introns were marked in red)

T1-A(4-exon):

Same gene structure in other plant families: Not find

>Cla019863

ATGGGTTCTTCAACTGATGCAACAGAATCACCAGCTTTCAAATGGACTCATGATGTGTTTTTGAGTTTCAGAGGAGAGGATACTCGCTCCAATTTCACCAGTCATCTTGACATGGCCTTGCGTCAAAACGGAGCCAACGTCTTCATAGACTACAAGCTCGAAAGGGGTGAGCAAATTTCTGAAACCCTTTTAAGAACTATACAGGAAGCTTTGATTTCTATTGTTGTATTCTCTCAAAATTATGCATCTTCTTCCTGGTGTCTGGATGAATTGGTGAAAATAATGGATTGTAAGCAATCCAAGGGCCAGATTGTTTGGCCAATTTTCTATAAGGTGGATCCGTCGGATGTACGAAAACAAACAGGAAGTTTTGGAGAAGCACTGGCCAAACATCAGGCTAACTTCAACCCCAAGACCCAAATTTGGAGGGAAGCTTTAACTACTGCTGCTAACTTGTCTGGTTTTGATCTAGGAAATTATGG

GTAATATATTTTTACACACATTTTGTCTTTTCTTTTCCAAACCTCATTTTTATGCCCCCCTTTTTTTTTTATTTTTATTCTTATATAGCACACTAACCAATTTTAATCTTACAACATTCATTCTCTCTCAAGATGCAATTTAATTTTGGTATGATTATGTACCTGACAACAG

GACAGAGGCTAAACTAATTAAAAAACTGGTAGACGAAGTGTCTGCTGTATTAAATCGCAAATGCACGCCATTATTTGTAGCTAATTATCCAGTTGGAATTGATGGTCGGCTACAACATATGAACTTACAATCACGTGTGTCTAAGTCTGGTGTTTACATGTTGGGGATATGTGGTATTGGAGGCCTTGGTAAGACAACTTTGGCTAAAGCTTTATACAACAAAATTGCTACCCAATTTGAAGGTTGCTGCTTTCTACCAGATGTTAGAGAAGCCTCAAAGCAATTCAAAGGCCTTGTTCAACTACAGGAAAACCTGCTGTTTGAGATCTTAAAGGATAATTTGAGGGTTTTCAATCTTGACAGGGGAATTAATATCATAAGGAATAGACTGCGTTCAAAGAAAGTTCTTATAGTTCTTGATGATGTGGATAAGCGTGAGCAATTAGAAGCATTGGCTGGTGGGCGTGATTGGTTTGGTGAAGGCAGTAAAATCGTTGTGACCACAAGGAATAGCCATTTACTATATAGCCGTAAATTTGATCAAGTGCACTTCATTGAAGGATTGAATGAAGACGAATCTTTTGAGCTTTTTTGTTGGAATGCTTTCGAGGAAAGTCATCCATCAAGTAATTCTTTGTTAGACCTTTCAAAACGAGCCACAAGTTATTGTAAAGGCCATCCTTTAGCTCTGGTTGTTTTGGGTTCTTTCCTTCGTACCAGATATGAAACAGATTGGACTTGTATATTAGATGGATTTGAAAACTCTCTGGGTAAAGATATTAAAGATGTTCTTCAATTAAGTTTTGATGGGCTGGAGGACAAAGTAAAGGATATCTTTCTTGATATTTCTTGTTTCTTTGTGGGAGAGGAAGTTGAGCGTGCTAAGAGGATGTTGAACGCATGCCATCAGAATCTAGATTTCAGAATTGTGGAACTCGTGGATCTTTCACTTGTTACGATTGAAGGTGGGAAAGTGCAAATGCATGAATTAATAAAACAGATGGGTCATCAAATAGTTTGTGATGAATCTTCTGAGCCTGGAAGAAGGAGTAGGTTGTGGTTGATGGAACCCATTTTGGAGGTGTTGGATAACAATTTA

GTGAGTTAACTCTTACCTAAACTATCTTTAATTTACTTATTTCCAGGATTAAACTACAAAATAAATAATATTACTTTGCAG

GAAAGCGATGCAATTAAAGGCATAAAGTTGGAGTTGGATTATCCCACAAGGGTAGCCGTGGATCCACAAGCTTTTAGAAACATGAAAAATCTAAGATTGCTTATTGTTAAAAATGCAAGAGTTTCTACAGAGATTAATTACCTACCCAGTAGCTTAAAGTGGATACAGTGGCATGGATTTGCTCAACCATCTTTGCCTTCCCACTTCATTATGAAAAATCTGGTTGGACTTGATTTGCAACATAGCTTCATCAGTGAATTTGGGAAAGGACTTCAG

GTCAATTTTAATGCCTACCTTACTTCCCTAAAACATCTTGTTTTCTCATAATAATCTTCCAACATAACATTAAATTCATAATATATATGGTTACTTTGTTTAATTGTTGATTTCACTTTAATGTGCTAACAAAAATTAATCATATTAAAATAAATTTCATTCTATATCCTTAACAATTATTAATCATTTTAATTACAACTAGAGTGATTTTCAAATATATCAAAATGAACCCAACTATTTACAAATATAGCAAAACATTTCCATGGTTGTTCATCACTAATATACGATAACTTTTTGCTATATTTATAAAATATTTTCAACAGTTTTGCTATTTAAAATAATTACCCTTAAAATTAACCAATATATATTTAATAAATGTCATATTAGTTATAATTTAATATTTAATCATTTTTAGTTAGATAATAATAAGAAAATATATTTTAATAAACAATATATTATATAAAAAGTGAAAAAAAAGAGATAAAACTAGGGTAATTGTTTTAAATGGCAAAACTGCTAGAAGTATTTACAAATATAACAAATATAGATTCTGATACACACCTATCATTAATAAACCGTGACAGTAATAGTAGTCTATTAATGTCTGTTAGTGATAAACATGACATTTTGCTATATTTGTAAATATGTTGGGTCATTTTGCTACATTTGAAAATAATTCATAAAACTATGATAAGTTAATAAAGAATATTAATAATAATAATAAAAAATAAAAAAAAAACTAAAATAAGTTTTATAAATATGATATGTTAATAATGAATGAAGCAATAAAAATTAATCAGAATAAACATGATTATATGTGATTAATGAAGGATCAAATTAAATAGATTATTAATCTTGATATGGATTGAGTTTATCACTGATTTTGGTATGAAATATTATGAGTTTACCTAGATATTGATTAATGGTTGTTACAGATTTACATATATTCTATTTTCTCTGTATTTACAAGTATTTTCGTAATATTATTTTATCTGTTTATTTTCCATGTGTTTTAG

GATTGTGAAAAGTTGAAGTATGTTGATCTTAGCCACTCTACTTTATTACGCCAAATTCCTGATTTCTCTGCAGCATCAAACCTTGAAGAATTATATCTCAGCAATTGCACAAATTTAAGAACAATAGATAAGTCTGTATTTTCTCTTAATAAGCTTACTATCTTAAAGCTTGATGGTTGTTCTAACCTTAAAACCCTTCCAACAAGCTACTTCCTGTTATGGTCTCTTCAACATTTGAATCTCTCTTATTGCAATAAACTTGAGAGAATTCCAGACTTCTCCTCAGCATCAAACCTTAAGAGTTTGTATCTCGAAGAATGCACAAATTTAATAGAGATTGATGAGTCTGTTGGATCTTTGGATAAGCTTGTTGCCTTGGTCCTTACAGGATGCACCAACCTTGTAAAGCTTCCAAGCCGTCTTAGGTTAAAGTCCCTTGATTATTTAGGACTTTCTAGGTGTCGTAAGCTTGAAAACTTCCCAACAATTGATGAAAACATGAGATCTTTAAGGTTGTTGGATTTGGATTTTACTGCCATAAAGGAGCTACCTTCATCAATTGAATATCTTACTAAGCTCTGTATATTAAACCTTAACAACTGCACAAACCTCATCTCCCTTCCCGAAACAATTTATTTGTTAATGTCCTTATGGAATCTTGACTTAAGGAATTGCAAGCTTCTTCAAGAAATTCCAAACCTTCCCCAGAATATACAGAAGATGGATGCCACTGGTTGCAAATCGTTGGCTACAAGTCCAGATATCATTGTGGATATAATATCAAGAAAACAGGTTCCTCTCTTTCCATTCAATTTGTTCTTCTCTTGTAAACAGTTTTATGCATATGAATTCTTATTCTCGTAG

T1-B (5-exons):

Same gene structure in other plant families:

LOC100796191 TMV resistance protein N-like [*Glycine max*(soybean) ]

Gene ID: 100796191, updated on 1-Feb-2013

>Csa006711

ATGGGTTCTTCCACTGCTGCAACAGAATCCATGGCTTTTGAATGGAGTTATGATGTTTTTTTGAGTTTCAGAGGAGAGGATACTCGTACCAATTTCACCAGTCATCTTGATATGGCCTTGCGTCAAAAGGGGGTCAACGTCTTCATAGACGACAAGCTCGAAAGGGGTGAGCAAATTTCTGAATCCCTTTTCAAATCTATACAGGAAGCTTCCATTTCTATTGTTATATTCTCTCAAAATTATGCATCTTCTTCCTGGTGTCTGGATGAATTGGTGAACATAATTGAGTGTAAGAAATCCAAGGGCCAGAATGTTTTCCCAGTTTTCTATAAGGTGGATCCGTCGGATATACGAAAACAAACTGGTAGCTTCGGAGAAGCACTGGCCAAACATCAGCCTAAGTTCCAAACAAAGACCCAAATTTGGAGGGAAGCTTTAACTACTGCTGCTAACTTGTCTGGTTGGGATCTAGGAGCTTATAG

GTATATATTTTTACGTAAATCTTGCTTGTCCTATCTTTTCCAGATCTCATTTTCAATGCCATGTCTTTTTTTTTTTTACTCATTTATCAACGAGCACACTGCACAACTTTTGTCTTAGAAGATCCATTCTCTCGCAAGATGCCATTTAATTAACTGTGAACATCATTCTTTAGTTTTGTTATCATTACGTATTTAACAACAGGAG

GGAGGCTGATCTTATTCGGGATCTTGTTAAGGAAGTGTTATCTACAATAAATCGCACTCGCACACCCTTATATGTCGCCAAGTATCCAGTTGGAATTGATTCTCAACTAGAATACATGAAGTTTCACTCACATCATCTCAACAAGGGAAACAAATTCCAATATTGGACACAAAATGAGTATGAGTCTGATATTGGTGTTTACATGGTGGGGATATATGGCATTGGAGGCCTTGGTAAGACAACTTTGGCTAAAGCTCTATACAATAAAATAGCTAGCCAATTTGAAGGGTGCTGTTTTCTATCAAATGTTCGACAAGCTTCAAACCAATTCAATGGCCTTGTTCAACTACAGCAAAACCTACTCTATGAAATCTTAGAGGATGATTTGAAGTTTGTCAATCTTGATAAAGGAATTACCATCATAAGGAATAGACTGCGTTCAAAGAAAGTTTTGATAGTTCTTGATGATGTGGATAAGCTCGAACAACTAGAAGCATTGGTTGGTGGACGTGATTGGTTTGGTCAAGGTAGTAAAATCATAGTGACGACGAGGAATAGTCATTTACTTTCTAGCCATGGATTTGATGAAATGCACAATATTCAAGGATTGAATCAAGACAGAGCTATTGAGCTTTTTAGTTGGCATGCTTTTAAGGAAAGTCATCCATCAAGTAATTATTTAGACCTTGCCGAACGTGCTACAAGTTATTGTAAAGGTCATCCTTTGGCTCTTGTTGTTCTGGGTTCTTTCCTTTGTAATAGAGGTCAAACAGAATGGAGAAGTATATTGGATAAATTTGAAAACTCTTTGAACAATGATATTAAAGATATTCTTCAATTAAGTTTTGATGGGCTGGAAGGTGGAGTAAAGGATATTTTTCTTGATATTTCTTGTTTATTTGTAGGGGAAAAATACAATAATTGTGCTAAAAAAATGTTGAGTGCATGCCATTTGAACGTAGATTTTGGAATTATGATACTCATGGATCTTTCACTTGTTACGATTGAAAAGGATAGAGTGCAAATGCACGGATTAATACAACAGATGGGTCATAGCATAGTTCATAATGAATCATTTGAGTCAGGAAAGAGGAGTAGATTGTGGTCGGAGCGGGACATTTGGAAC

GTGTTTGTTAATAATTCGGTGAGTAACTCTTTCCTAAAATATCGAATATTTTAATTATTTTCAGGACTTCACCTGATAATAAATGTTTGTGAAAGTAAGTTGTCAATAATGTTAAATTACTAAATCTATGACTTCGTAG

GGAACAGATGCAATTAAAGCCATAAAGTTAGACTTGCCTAATCCCATAAACGTAAATGTAGATCCAAAAGCATTCTTTAGAAGCATGAAAAATTTGAGATTGCTTATCATTCGAAATGCACAAGTTTGTACAAAGATTAAGTACCTACCTAATAGCTTAAAGTGGATTGAGTGGCAAGGATTTGCTCATCGAACTTTCCCGTCGTGCTTCATTACCAAAAATCTTGTTGGACTTGATTTGCGACGTAGCTTCATCAAAAGATTTGGGAAAAGACTTGAG

GTAAAATATATTTCTTTCCTTATGTGTATTTGATTGGAGGTTTCTTCATTGTTTTTCTTGAAAACTTTTAATGGGTAGCTTAACTACATTTTACAAAAAAAAATCACAAATGCGATTTTAGCAAAATTTATCATGGATTAACTTCTAGTAGTCATATATTTTTAACAAATTTATAAAATATATAACAAAATTTATCAATGATAGACTGATCTATCGTACAACAATAGGCTCCTACTAGTCATATGGTCTATCATTAATATACTCCTATTAATGATATGATCTACCGTGGCTAAAATTTAAAAGTTGAAGCTTTACAATTTTTTGGATTGTAATATATATGTTATCATTTTGAGGGTTGATTTTTTTTAATGCAATTATCATAATTCAAAATAATATATTGGGTAGATTCTTTTTATGTTATGACTTTACTTGAATAAGATTTGTTATATAGAAGCTAAGATTGTCCAATTATATTGTGTGTCCTTTCAGTTACAAGATTTCGTATTAGGTTAAGTTTTTAATTTTTTTAATTTTAAATTTGATTTAAGATCAAATCAAACTAAAAATTGAAATCAAATTTCGAGTGTAATTAAATTGACTAAACTACATTCTCAATCTGTTTAAGATATCAAATTAAATGGTTAGTTTACAATCTACAAAATTCAAGATAATATATTTTAGTCATAATGGTTACTTTAAAAAATAATAATTAATTAATAAATTACTTTTAAACTAAAAACTTAATTCAAAACTATGTTTAATTTAAACTAATTATTCTTTAGGTTTTTTAAGCTTTTTGGGGTGGATTACGTTTCAAACTTATAAAACTGAACATCAAAATAATTGTAGTCAAGAGTCCGTTATTTATCAACATGAGTGTAGTACAACTGACTTAATGTTTGTACTATCAATTGGATTGATTACTCATTTATTTTTTGTATATAAAATTTTATAATTAGTTTGCCTAAATACGATTAAAGGTTATTTATAGATTTTTTTTACCTATATTTTATATTTTCTATATTTACAATTATTTTTTTAGCCACTATTTTATCTAAATTTTGTTTGGTATGCCAATTATATTACATTACATACCCCTTTTCCTTTTGGTTTCTTTTTCAATATGTTTTAGGATTGTGAAAGGTTGAAG

CATGTTGATCTTAGCTACTCTACTTTATTAGAGAAAATTCCTGATTTATCCGCTGCATCAAACCTTGAAGAATTGTATCTCATCAATTGCACAAATTTAGGAATGATAGATAAGTCTGTTTTCTCTCTCAATAAGCTTACTGTCCTAAACTTTAAAGGTTGTTCTAACCTTAAAAAGCTTCCAAAAGGCTACTTCATGTTCAGTTCTCTTAAAATATTGAATCTCTCTTACTGCCAAGAACTTGAGAAAATTCCAGACTTATCTTCAGCATCAAACCTTCAGAGCTTGCTACTCAACGGATGCACAAATTTAAGAGTGATTCATGAATCTGTTGGATCTTTGAATGAGCTTGTATTGTTGGACCTTGGACAATGCACTAACCTTTCAAAGCTTCCGAGCTATCTCAGGTTAAAGTCTCTTGTCTATTTGGTACTTTTTGGGTGTGGTAAGCTTGAAAGCTTTCCAACAATTGCTGAAAACATGAAATCTTTAAGGTGCTTGGATTTGCATTCCACCGCCATAAAGGAGTTACCTTCATCACTTGGATATCTTACTCAACTCGATAAATTACACCTTACCGGTTGCACAAATCTCATCTCCCTTCCCAATACAATCTATTTGTTAAGGAATCTTAACGAACTTCATCTTGGTGGGTGTTCTAGATTTGAAATGTTTCCCCATAAATGGGTCCCAACCATCCAACCAGTATGCTCTCCTTCAAAAATGATGGAAGCAGCTTCGTGGAGCTTAGAGTTTCCCCATTTAGTAGTACCAAATGAAAGCATATGTTCCCATTTCACTTTGTTGGATCTTAAATCTTGCAACATATCAAGTGCAAAATTTTTGGAAATTTTATGTGATGTTGCCCCTTTCTTATCTGATCTACGTTTGTCCGAAAACAAATTCTCTAGTTTACCCTCATGTCTCCACAAGTTCATGTCCTTATCGAATCTTGAATTAAGGAATTGTAAGTTTCTTCAAGAAATCCCAAACCTTCCCCAAAACATACGAAACTTGGATGCCAGTGGTTGCAAATCGTTGGCTCGAAGTCCAGATAACATTGTGGATATAATATCAATAAAACAG

GTTTGATTCTTTCCATTCATTTTGTTCCTATCTTTGCACATAGACAATTTAATGCATATGAATTTTTATTCTCTATAG

GACCTTGAATTGGGTGAGATTTTAAGAGAGTTCTTATTAACGGACATTGAGATTCCAGAATGGTTCAGCTATAAGACTACATCCAATTTGGTGAGTGCTAGCTTTCGTCACTATCCAGACATGGAAAGAACTTTGGCTGTCGCTGTTAGTTTTAAAGTGAATGGAGATTCATCTGAAAGTGAGGCCCAAATTTCATGCAATATATTCATCTACAATAAACTCCGTTGTTTGTTTTCAAGATCATTTCTTCCATCAAAATCAGAATATATGTGGTTAGTAACAATTTCTCTAGCGTGTTCCCTGGAGGTGAATGATTGGAATAAAGTTTTCGTCTGGTTTGAGGTTCATGAAGCACATGGTGTAACTGTAACAAGGTATGGGGTCCATGTCACTGAACAACTCCATGGGATACAAACGGATGTCAAGTGGCCGATGGTAAATTATGCTGATTTTTATCAACTGGAGAAATTGCGAAGGGATCTGTAA

T1-C (6-exons):

Same gene structure in other plant families: Not find

>Csa006744

ATGGATTCTTCCACTGTTGCAACAGAATCACCGACTTTCAAATGGACTTATGATGTGTTTTTGAGTTTCAGAGGAGAGGATACTCGCACCAATTTCACCAGTCATCTTGATATGGCCTTGCGTCAAAAGGGTGTCAATGTCTTCATAAACGACAAGCTCGAAAGGGGTGAGCAAATTTCTGAATCCCTTTTCAAATCTATACAGGAAGCTTCCATTTCTATTGTTATATTCTCTCAAAATTATGCATCTTCTTCCTGGTGTCTGGATGAGTTGGTGAACATAATTGAGTGTAAGAAATCCAAGGGCCAGAATGTTTTCCCAGTTTTCTATAAGGTGGATCCGTCGGATATACGAAAACAAACTGGTAGCTTCGGAGAAGCACTGGCCAAACATCAGCCTAAGTTCCAAACAAAGACCCAAATTTGGAGGGAAGCTTTAACTACTGCTGCTAACTTGTCTGGTTGGAATCTAGGAACTAG

GTATATATTTTTACAGACATTTTTTTTTTCCATTCCAAATTTCATTTTTATGCCCATGATTTGTATGTATGTACGTATGTATGTATTTACGTATGTATGTAAATAAGCACACAGACCAAATTTTAATTTTAGAAGATTCATTCTCTCTCAAGATGCAATTTAATAATTAAATGTGTTCATCCCTTTTAATTTTGGTATGATTATGCACTTAACAACAG

GAAGGAGGCTGATCTTATTGGAGATCTTGTTAAAAAAGTGTTGTCTGTATTAAACCGCACTTGCACGCCCTTATATGTAGCTAAGTATCCGGTTGGAATTGATTCTAAACTAGAATATATGAAGCTTCGTTCACATAGTCTTTTTGAGAAGAGCAACAAATTCCATTATCGAAAACAACATGAGTATGAGTCTGATACTGGTGTTTACATGGTGGGCTTATATGGCATTGGAGGCATTGGTAAGACAACTTTGGCTAAAGCTTTATACAACAAAATTGCTAGCCAATTTGAAGGTTGCTGCTTTCTATCAAATGTTAGAGAAGCTTCAAAGCAATTCAATGGCCTTGCTCAACTACAGGAAACCCTACTCTATGAGATCCTAACGGTTGATTTGAAGGTTGTCAACCTTGATAGAGGAATTAACATCATAAGGAATAGATTGTGTTTGAAGAAAGTCCTTATAGTTCTTGATGATGTAGATAAGCTTGAGCAGTTAGAAGCATTGGTTGGCGGGCGTGATTGGTTTGGCCAAGGCAGTAGAATCATTGTGACGACAAGGAACAAACATTTACTTTCTAGCCATGGCTTTGATGAAATGAAAAATATTCTAGGATTGGATGAAGACAAAGCTATTGAGCTTTTTAGTTGGCATGCTTTCAAGAAAAATCATCCATCAAGTAATTATTTAGACCTTTCAAAACGTGCTACAAGTTATTGTAAAGGCCATCCTTTGGCTCTCGTTGTTTTGGGTTCTTTCCTCTGTACCAGAGATCAAGTAGAATGGTGTAGTATATTAGATGAATTTGAAAACTCTTTGAACAAAGATATCAAAGATATTCTTCAATTAAGTTTTGATGGTCTGGAAGACAAAGTAAAGGATATCTTTCTTGATATTTCTTGTTTACTTGTGGGAGAGAAAGGTAAGTACGTTAAGGATATGTTGAGTGCATGTCATGTAAATCTAGATTTTGGAATTATAGTACTCACGGATCTTTCATTTATTACGATTGAAAATGGCATAATGCAAATGCATGATTTAATAAAACAGATGGGTCATAAAATAGTTTGTGGTGAATCTCTTGAGCTTGGAAAGAGGAGTAGGTTGTGGTTGGTACAGGATGTTTGGGAG

GTGCTTGTTAATAATTAAGTGAGTAACTCTCTTTAACATACTTATTTATAATATTTCAAATAACGAAGAAGGTTTGTGAAATGTTGTTAAGTTACTAAATATATAACTTTTTCAG

GGAACAGACGCAGTTAAAGGCATAAAGTTGGACTTTCCTAATTCCACGAGGCTGGATGTGGATCCACAAGCTTTTAGAAAAATGAAAAATTTGAGATTGCTTATTGTTCAAAATGCAAGATTTTCTACAAAGATTGAGTACCTACCTGATAGCTTAAAGTGGATTAAGTGGCATGGATTTCGTCAACCAACTTTTCCTTCGTTCTTCACTATGAAAAATCTTGTTGGACTAGATTTGCAACATAGCTTCATCAAAACATTTGGGAAAAGACTTGAG

GTAAAGTTTATTTCTATATATGCAATAAATGGGTAACTTACATTTTAAAATAATTCACAAATAACACAGACCTATAATAATAGTTTTGGAATGTAAGGAGTTTTTTGTGGCGTTAAATTTTGGATTCTATTAATGATAGTCATTGATAAACTTCTATCACCGTAGTATATTAGTGATAGAATTTAAAAATTTTGCTAATTTGCTATTTTTTAAAATTTCTCCCTTATTATTAGTTTGCCTAAATACTGATTAATGCTTATTTATAGAATTTTTTTACCTATATTATTCTATAATTTTTTATATTTACAATTATTGTTTTAGGCACTGATTTATCTAAATTTTGTTTGGTATGCTAATTACATACCTCTTTTCCTTTTGGTTTATTTTCCAATGTGTTTTAG

GATTGTGAAAGGTTGAAGCTTGTTGATCTTAGCTACTCTACTTTCTTAGAGAAAATTCCTAATTTCTCTGCAGCATCAAACCTTGAAGAGTTGTATCTCTCCAATTGCACAAATTTAGGAATGATAGATAAGTCTGTTTTCTCTCTCGATAAGCTTACTGTCCTAAACCTTGATGGTTGTTCTAACCTTAAAAAGCTTCCAAGAGGCTACTTCATGTTAAGTTCTCTTAAAAAATTGAATCTCTCTTACTGCAAAAAACTTGAGAAAATTCCAGACTTATCTTCAGCATCAAACCTTACGAGCTTGCATATCTATGAATGCACAAATTTAAGAGTAATTCATGAATCTGTTGGATCTTTGGATAAGCTTGAAGGTTTGTACCTTAAACAATGCACTAACCTGGTAAAGCTTCCAAGCTATCTCAGCTTAAAGTCTCTTCTATGTTTATCACTTTCTGGGTGTTGTAAGCTTGAAAGCTTCCCAACAATTGCTGAAAACATGAAATCTTTAAGGACCTTGAATTTGGATTTTACTGCCATAAAGGAGTTACCTTCATCAATTAGATATCTCACTAAGCTTTGGACATTAAAACTTAATGGTTGCACAAACCTCATCTCCCTTCCCAATACAATTTATTTGTTAAGGAGTCTTAAGAATCTTCTTCTTAGTGGCTGTTCTATATTTGGAATGTTTCCCGATAAATGGAACCCAACCATCCAACCAGTATGCTCTCCTTCAAAAATGATGGAAACTGCTTTGTGGAGCTTAAAAGTTCCCCATTTCCTAGTACCAAATGAAAGTTTTTCCCATATCACTTTGTTGGATCTTCAGTCTTGCAACATATCAAATGCAAATTTTTTGGATATTTTATGTGACGTTGCTCCTTTCTTATCTGATCTACGCTTGTCCGAAAACAAATTCTCTAGTTTACCCTCATGTCTCCACAAGTTCATGTCCTTATCGAATCTTGAATTAAGGAATTGTAAGTTTCTTCAAGAAATTCCAAGCCTTCCTGAGAGTATACAAAAAATGGATGCCTGTGGTTGTGAATCGTTGGCTCGAATTCCAGATAACATTGTGGATATAATATCAAAAAAACAG

GTTTGCCTCTAATTTCCATTCGATTTATATTCTTACCTTGTAAACAAATTAATGCATTGAGAATTTTTGTTCTCTATAG

GACCTCACAATGGGTGAGATTTCAAGAGAGTTTTTATTAACGGGGATTGAGATTCCAGAATGGTTCAGCTATAAGACTACATCCAATTTGGTGAGTGCTAGCTTTCGTCACTATCCAGACATGGAAAGAACTTTGGCTGCCTGTGTTAGTTTCAAAGTAAATGGAAATTCATCTGAAAGAGGTGCCCGGATTTCATGCAATATATTCGTCTGCAATAGACTCTATTTTTCATTGTCAAGACCATTTCTTCCCTCAAAATCAGAATATATGTGGTTAGTAACAACTTCTCTAGCGTTGGGTTCCATGGAGGTGAATGACTGGAATAAAGTTTTGGTCTGGTTTGAGGTTCATGAAGCACATAGTGAGGTTAATGCAACTATAACAAGGTATGGTGTCCATGTCACTGAAGAGCTCCATGCGATACAAACGGATGTCAAGTGGCCGATGGTAAATTATGCTGATTTTTATCAACTGGAGAAATTGCAAAGTCT

GTAAGTTGATTCTTTACTTGTTAGTTATTTACTTTTTTTTGTTTGGAGTTGAAGTATGATAGATCTCAAAGGGGAGATGTGTATGTTATGATTATGTTTGTTTAATGGCATAG

GGATATTGAGGAACTTCTTCTCAAACGCTTTTTTGAAGAAATGTCGTGCTGGTCCAATTCCCAAGCAATGTTATATGCGGCAAATTATGATCCAGAAGCAATAATCGATTCGAATATACAACCTATGATATTTCCATTGCACGTAACATATAATGGTGAGACATTTATATGTGGAATGGAAGGCATGGGAGACACTACACTCGCCAACTCTTTATGCAATAAATTTAATTGGCCAAATGACAACGTTCGGGCAAGAGAAGCTTTAGATAATTCTACAAGCTTTTTGCATTTTCGAGGAGGAAAGTTTAATGGAGGTTCCTGGTCATCGTCCCACCACCGTAAGCGTGGAGATGGTGAAAGAGGAACCAATATCACAACCCGCACAATATCCTCCAAACGCTATTTGATACTCTTTCATAAAGCGGGGAGCTATGATGATTTATTTAACTTTGCTGGTAGCCACCGTTTGATTGCAGGTTCTCGCAGTTATGACAGTCTTAACGGAAGAGGTGATGTTCGGCTTCTGATTGAAAGGGTTGATACATCCTTGCTCTGA

T2 (4-exons):

Same gene structure in other plant families: *Solanum lycopersicum* bacterial spot disease resistance protein 4 (Bs4) gene, Bs4-MM allele, complete cds

GenBank: AY438027.1

>MELO3C008730

ATGGTGGACGTCCCATCATCTTCGACCACCAGACGGTGGATGTACGACGTCTTCTTAAGCTTTCGAGGTGAGGACACTCGTCAGAATTTCACCAAACACCTTTACGACGCCCTCGATACTGCCGGAGTCAACACGTTTCGTGACGACGTTGAACTCCGGCAAGGAGACGCCGTGGGCTCAGAGCTTGTGGTAGCGATTAAAAAATCGAGGATAGCAGTCGTGGTGTTCTCCGATGGCTATGCCGACTCACAGTGGTGCCTAGGGGAGATCGCCGAGATCATGGATTGCCGGACTGTTGAGGGCCAACTGGTCCTTCCGATCTTCTATGAGGTGGATCCGTCGGACGTTAGGAAGCAGAAGGGGAGGTTTGCGGCCGCATTTGAGAAGCACGAGAAGAGATTTGGTGTAGATTCGGTGGAGGTCCTGCGGTGGAGGGCGGCGCTCAGAGAGGCCGCTAGCTTGTCCGGCTGGGACTTGAGGCAGCTAGCCGATGG

GTACGTATTATTATTGTTATTTTCCATCCTTTTATTTACCTTTCCTTGAAAATTGCAAAAACAATGGGCAAATGGGAATAATAAAAATAATAGATTAAAAAGAATGAGGAATCGTCAAAAACGTTGGAAACTATTTACGTATATATATATATATATAAAACTCGGATGTGTTAATAGAAAATTTGATAATTTTGTTGTTATGCTTAAATAGTTTCAATTTTTTTATTATATTTGAAAATGTCTTAAATATTTTAATTTAATTTAATTATCATGAACAGAAAAGAAGAATCAATTACAAAATATTTAAATGCCCCTTATAATTACTTTTTGAAATTTACGAGCAAGATATAAAACTAGATCAAAGCTATAACTAAAAAATAATTTTTTTTTTTACTTGAATGAATAAAATATCCCAGAATTATTCCAACCTAGTTTAACTAGCATAACTGAGGTAATATACATCCGCGGTGGATCCATAAAATATATTGATAGAGGACTAAGATTCGAAATTGAGAGAGACTCGAGCCCTCGAACTTATACTAAATCCTCTGTGTATATATCAAATTATAATACTATGATTAATATAATGGATTCAAGTTTTTTCTTTTTGAATTTTGTTAAGTTGGGGTTGTTTATGAAAAGTTTTTGGAATAAAATTAAAGCTGTATGGTTTTCACTTTCTCTGAATATTTTGGTTGAAGAGACATATTTCAATTTCATCACATTTTAAATTTTCGTGTATCAATTTGATATATAAGAATATATTTGACACTCATCTCAAATGGACTCTTACAACTTCAAATATTTTAAGTTAATCCTATATTTTGGAGTTCATTTATTTATTTTTTTTTTTTTGTAAATTAAAAGATAAAAGTTGTTCTCTCTTCAATTAGATTAAATCTTAAAATTTTAAGTTAATTAAGCTGAGCTCACGAATAAGAAAAATATATTTTCAAATTCATATAGGTGTTGGGTTAATAATATATGACTTGAGAGAGGGAGGTTGTTCTCTCGCTATATTTTCCAATTCTATTTTCAATACAATTTAGGTTAAATCAAAATTTAGGCTTTATTATGTGGTTTGACCTTTTCTTAATTTAGAAGTTTAAAATGTATTTTAATTCATAGTCCAGTTGTGATTTTTTTGGCCTTTTGTTTGATTTTGTTATTTCTCTTGTTTGTTTTATGGACCAATGATCTCTTACAATTTTCAGAATTGTCAAGAGGTTTGAATAAAAAAAAAAAAAAAAACAGATAACATCTATTAGTGTCAATTGAGATGGATGATGCTAGTGTATCATTGATTATTGTTATCAACTATGAGACTTTTACAATTTATAATATTTGTAAATTAGGTTAGTTTATTTTCTTTCTATATTTAAAAAATATCTCAAAATTAATTTAATATTTAATTTTACACTCTTAATTAATCATATTTTTACATAGTATTGAAATGAAAATTGGATTAATGAAAGAAAAAACATGTTCTAAATTAAGTAATTGGAAATCCACTCCAAATTAAACCTGCCATTCAATTTCACAATCAAGTTCCCGGCAAGAAAAATACAAATGTAGAAGAAAAGTTGTTGCCATTATAGAGACAAGAAAGTAAGCAAGGAATAGGATTTTGAAAATTCGACTTTTATTGTTCAATTGATTTATGGTTTTGAAAACTATGCATTGAATTAACATAACAAGAACCAAAAACATAAAGAAAAAAATAATGACAAAAGAAAACTATTCGAAACTCTTCCTTTATTTTTCTTTTTTCTTTTTTTTTTTCTTTTTAATAAAGAAAGATGAAACACGAAATAAAAGATGTAATGAGTTGTTTAAAGAAAGCTCCTTGAAGTTTCCAATTGGTGAATAGTACGTACTTGGTTAAATATGTTTAATATAATATTACTAACATTTCAAAAGTCAAAACTTCTTTCTTTTATTTTAATTCTGAAAACATTTTCTTTCTTAAAATATTAGTTTCCTTCTTATATCTCAATCATTCTTTCATTTCAATTCTTATAATTTTTAAATAATTCATTTTAGTTCAAACTCACTGTCCATTAAATATGATTAAAAAAAATCATATTTGTACTTTCAATTTCTCTTTCTTTCTTTCTTTATTTATTTTTTCATATCATCTTCTCCATCAAAAGCTTTCCCTCTTGTTCTTCTTTCTCTACGAATTTTATTAGTAGGGGTTCTGCAGGGAGGCCATAATTATATTTATGGGCATGACAGCCAACTGAGGTGCTGGAGAAAAATGCGTAATAATAACTTGATCGGTGAGCGGAAGCTATGAGACTGTTTTGCCATTGAGCTTCTAGCTAGTATTATTATTTTTTGGTGGATTTTTTTGATGCTAGAAGATATTGATCATGTTAACGGATTGGTTAATTATATATCTTAATGGCAAAAAAAAATAATATATATATATATTCTGATTGTCGACATTGACAACAG

GCATGAAGGAAAGTTCATAACCAAAATAGTGGAAAGGGTTCAAAGCGAACTGAGAGTGACATATTTGGAAGTCGCCATCTACCCCGTTGGCATTGATGTTCGTCTCAAACACTTGATCTCATTAATGGCCATTTCTACAAACCACTCCACTCTCGTCCTCGGCATCTATGGCATGAGCGGCATTGGCAAAACCACTCTCTCTAAAGCACTCTTCAACCACTTCTTCCACTTCTTCAATTCTAGATCTTTTCTCCCCAGCATCAACTCCATCTCTAACTCCTCTCCCGACGCTCTCCTTCGCCTCCAACAAACTCTCCTCTCCGATCTCCTCATCGCCACTAACCTCCGCTCTCGTTCCTCGACCACCACCGACTCCACCGTCGTTCGGATGCAGGAAAGACTCCAAAACAAAAAGGTCTTGGTTGTCCTCGACGACCTGGATCGTATCGAACAAGCAAATGCGCTAGCAATACGGGACCCAAGATGGTTTGGAAAGGGAAGCCGAATCATAATCACAACAAGAAACAAACAAATCTTGGACATTCTAAAAGTCGACAAAGTATACAACATGGAATCCAATCCACTGAACGACGAGGAATCATTGGAGCTTTTTAGCTACCACGCATTCCGGGAGCAAAATCCACCAGAGGAGCTTTTGGAATGTTCGAAATCCATCGTTTCGTACTGCGGAAATCTTCCTCTAGCTCTGGAAATCCTGGGTGGGTCATTCTTCGGAGGGAGACCGATGGAGGAATGGAGAAAAGCGTTGGAGAGACTGAAGATGATTCCGGCGGGGGATTTGCAAGAGAAGCTTCGATTAGGGTTTGAAGGATTGAGAGATGAGATGGAGAGGGAGATATTTCTTGATGTGTGTTGCTATTTTGTGGGAATGAAAGAGGAATTGGTAGTGAAGATTATGGATGGATGTGGAATGTATGGAGAAAGTGGATTGAGAGGATTGAAATGGAGGTGTTTGGTTGGTGTTGAGATTTGGAGTGGAAGGTTGAAGATGCATGATTTGGTTAGGGACATGGGGAGGGAGATTGTGAGGCAAACTTGTGTGAAGGAACCTGCTAGACGATCTAGGGTTTGGCTTTATCATGAGGCTCTCAAGATCTTACTCCATCAAACC

GTAAGTTAATTACTTCACTCATGCATTTTTATAGTATATAATTAATATGTAGTGAGATCATTGATTTAACAG

GGAACTGAAAACATTGAAGGACTTGCAATTGATATGGGCAAAGGAAACAAGGAGAAATTCAAATTGGAAGCATTTGGGAAAATGAGAAATCTAAGGTTACTCAAACTCAACTATGTGAATCTCATTGGAACTAATTTTGAGCAAATAATAAGCAAAGAATTAAGGTGGATTTGTTGGCATGGATTCCCTTTGAAGTCTATTCCAAGCTCATTTTATCAAGGAAACCTTGTTGCCATTGACATGAGGCATAGCAGCTTGATACATCCTTGGACTTGGAGGGATTCACAG

GTAAATTAATACAATTAATTAGCATTTTACTGATTTTATTTTCTAAATGATTGTTCATCTTTTTTTTTTTTTTTTTTTATTTAGAATAATTACTTTTCTCATCCCTTCTAAGTTTAGGGCTTAGTTTTCATTGATGCTCCTTCAATTTCAAAATCCTATACTCTTAGTTTCTTTTAGTTTGATTGCAATTTATTAGTCTCTAGGTTTAAAGATGATACCATTTTTACTTTTGACGTCTGAGCTTTGCATTTCAATTTTATCTCTAAATTTCAAAATATAATTACATTTTTTATTTAAATTTTTCAATAAGTAATCATTTAATTTTAATATTATTTTTTGTTTAGTATTCAAGATTGTATACGGTAGAGTAGTAATTCAAAAGCTTACGATTTTTAAAGTCAAAAGTAAATATATTTGTATCCAAGTCAGTGATGTCAAGTAAATTTAATCCTTAAAAATCAGAATATATATTTTGTTGAAATGCATTGATTATGTTATTTAGAGATTTAAACGACCAAATACGAAAAAGATCTCGGATATAACTTATTTTATATTTGTATTTTAAGTTCTCTTTAACAAAAATGTTCACGTGGATGTAGCTAACGTATTATTAGTAAATCACGCATCTGTGTGTTAACCTGTACTAATTTATGCTTTTATGTTTTCTTTATTAATTATCGATTCCATAACATTTTTAACTACTGCGTCTCTCTCACTCAAGTATGTACTTGTTTTGGGCAG

ATTCTTGAGAACCTAAAAGTTCTAAACCTAAGCCATTCCCAAAAGCTAAAGAAGTCCCCAAACTTCACAAAGCTCCCAAACCTAGAGCAGCTAAAACTCAAGAACTGCACAGCCTTATCAAGTCTCCACCCCTCCATTGGCCAACTTTGTAAGGTTCATCTCATCAACCTCCAAAACTGCACAAATCTTTCGTCTTTACCAACCTCCATCTACAATCTTCACTCCCTCCAAACTTTCATCATCTCTGGCTGCTCCAAGATTGACCGCCTCCACGACGACCTCGGCCACCTTGAATCCCTCACCACCCTTCTCGCTGACCGAACCGCCATATCCCACATCCCTTTCTCCATTGTCAAGTTGAAGAAGCTCACTGACTTATCTCTATGTGGTTGTAACAGCAGATCAGGGTATGTTAATCTTTTATTTTGTAACAATAATATATATGTTAGCTATATGATCAATATAATATTAAACTAAT

T3 (5-exons):

Same gene structure in other plant families:

Phytozome Locus name : ppa021718m [*Prunus persica* ]

> Csa002355

ATGGCCGACGAGCTCCGACCTCAACACGGGAATTGGACTTACGATGTTTTCTTGAGTTTTAGAGGTGAAGATACTCGCAAGAACTTCACTGATCATCTCTACTACGCATTCAAAGATGCAGGCATCAATGTGTTTCGAGACGATCCAGAGCTCGAACGGGGTGAAGACATAAGTTCGGAGCTCGAGCGAGCGATCGAAGGGTCGAAGGTGGCAGTTGTCGTATTCTCGGAAAGGTATGCGGAGTCGGGATGGTGTTTGGAGGAGTTGGTAAAGATCATGGAGTGCAGGAGGACTTTGAGACAACTGGTTTTCCCAATATTTTATAATGTGGATCCTTCATGTGTGAGGAAGCAAAAGGGTGAATTTGAAGAGGCTTTTGTTAAACATGAAGTGCGTTATTTTAGGGATATTGATAGAGTTCTTAAGTGGAGAATGGCTCTCACTGAAGCTGCTAATTTATCTGGTTGGGATTTGAGAAACATTGCAAATGG

GTATTTATTGATTCCTTCCTTTCTTTCAAGTTTTGAATGTTAATTAGCATATAGAAGTTTCTAAATAACCCTTTTGGTTTTTTGAAATTAAGCTTGGGAATACTTGGTTAAATTGCAAATTTGGTCCAAGAAAGTTATAGTTTAGTTTCTATGATAACTTATAAAAATCTGATAAAACACCAGTTTTAGAAAACATGTTCTTTGTTTTTGGAAATTGAACTCACAATTCATATGGACTGAAAAATTTACATTAACATATCATATACCCAACAATTATATTTGAGTCTTCTCCTAAGTTTCGATGAGTAACTTATCTAAATCTTGCTATATGGCAG

ACATGAAGCGAAGTTCATAAGGTTGATTGTTGAAAAGGTATCAAAGGAGGTGAACAGTAAATACTTATTCATAGCTCTTTATCCAGTGGGAATTGAATCAAGACTCAAACTTCTTTTATCACATCTTCATATTGGTTCAAATGATGTTAGATTTGTAGGAATTTTGGGGATGGGAGGACTGGGTAAAACCACCGTTGCAAAAGCACTTTACAACCAGCTTTATCACAACTTTGAAGCCAAATGTTTCCTTTCCAATATCAAAGCTGAAACCTCCAATCTAATTCACTTACAAAAACAACTCCTCTCTTCCATCACAAATTCTACCAACATCAATCTTGGAAACATCGACCAAGGAATCGCAGTGTTGCAAGAAAGACTTCGTTGCAAAAGGCTTCTTCTGATATTAGACGATGTAGACGACTTAAGCCAGTTAACTGCATTAGCAACAACTCGTGATTTGTTTGCTTCAGGTAGTAGAATTATCATAACAACTCGAGATCGACATCTGCTAAATCAGCTTGAAGTAGACGAAATTTGTTCCATCGATGAAATGGATGACGATGAAGCACTTGAACTCTTTAGTTGGCATGCTTTTCGCAATAGTTATCCATCAGAAACCTTTCATCAACTTTCGAAACAAGTGATCACTTATTGTGGAGGATTGCCATTAGCTCTCGAAGTGTTGGGTTCTTTCCTTTTTGGTAGAAGTAGAGAAGAATGGGAAGATACACTGAAGAAATTGAAGAAAATCCCAAACGATCAAATTCAAAAAAAGCTTAAAATAAGCTTTGATGGGCTAAACGATCATACTTACAAAGATATATTTCTCGACGTGTCATGTTTCTTTATTGGAATGGAAAGAAACTACGTTGAACAAATATTAGATGGGTGTGGATTTTTTCCAAGAATCGGAATTAGTGTTCTTCTTCAAAGATGTCTATTAACAATTGGAGACAAAAACAGATTAATGATGCATGATTTGTTAAGAGATATGGGGAGAGAAATTGTTCGTGAAAATTTTCCAAAATACCCTGAGAGACATTCAAGACTTTTTCTTCATGAGGAAGTGCTTTCTGTTCTTACAAGACAAAAG

GTAAGAAAAGAAAGTCCTATTTAAGTTGATCCAATTGTATTTTTAAGCTTAATATTTTTTATTTCATTGGTTTAG

GGAACTGATGCAACTGAAGGCCTAAGTTTGAAGTTGCCAAGATTTAGCAAGCAGAAGTTGAGCACAAAAGCATTTAATGAAATGCAAAAATTGAGGTTACTTCAACTTAATTTTGTTGATGTAAATGGAGATTTCAAGCATATTTCTGAAGAGATAAGATGGGTTTGTTGGCACGGATTTCCTTTGAAGTTTTTGCCTAAAGAATTTCATATGGACAAATTGGTTGCTATGGACTTGAGATATAGCCAAATCAGATTCTTTTGGAAGGAGTCTAAG

GTACACATTTTACTGTTGAATTAAAATGTTTTACTTTGGTTCTATACCTAGAATATAGCCAAATCAGATTCTTTCAATGAGTCTTTTTCACTGCTACTGTTAAAGTAGTGTTGATATTATTGAATTTACCGTAACTTATCAACTTTAACATTTTGGATTAATCACTGTTATTATAGCATCATAGCAATGCCATTTTCTTCTCAATTAACGATTTTTCCTGATCTCTTTGTTCTCTGTTGTGTGAACATGCAG

TTTCTCAAGAATTTGAAGTTTCTTAATCTAGGCCATTCTCATTACTTAACCCACACTCCAAACTTCTCCAAACTCCCCAATCTAGAGATACTCAGCCTCAAAGACTGCAAGAATTTGATTGAATTGCACCCTACAATTGGAGAATTAAAAGCCCTCATTTCCCTAAACTTAAAAGATTGCAAATCCCTCAATTCACTTCCAAATAGTTTCTCAAACTTAAAATCCTTACAAACTCTCATTATTTCAGGTTGTTCAAAGCTCAATAGTTTGCCAGAAGATTTAGGCGAAATTACATCATTAATAACTCTAATAGCTGATAACACACCAATCCAAAAAATCCCTAACACAATTATAAACTTAAAAAACCTCAAATATTTATCTTTATGTGGGTGCAAAGGGTCACCATCAAAATCATCATTCTCTTCAATGATTTGGTCTTGGATTTCACCAAAGAAATTATCTCAAAACTACACATCAATTCTTCTCCCTTCTTCATTACAAGGCTTAAACTCCTTAAGAAAATTATGCCTTAAAAATTGTAACTTGTCAAATAACACAATTCCAAAAGATATCGGGAGTTTGAGTTCTTTGAGAGAATTGGATTTGAGTGAGAATTTATTCCACAGTTTGCCATCAACTATCAGTGGCCTTTTGAAACTTGAGACACTTTTGTTGGATAATTGCCCTGAACTTCAATTTATACCAAATTTGCCACCACATTTGAGTTCATTGTATGCATCAAACTGTACTTCATTGGAAAGGACTTCAGATTTGTCTAATGTGAAGAAAATGGGATCTTTGTCTATGAGTAATTGTCCTAAACTTATGGAGATTCCTGGCTTGGACAAATTATTGGATTCTATTAGAGTTATTCACATGGAAGGATGTAGCAACATGTCCAATTCCTTCAAGGATACCATTCTACAG

GTTCTAATCTCTCTCTCTTTATTAATTGAAAACGAACACAAAATTAGAATAACTAAAATAAACATCGTTTTGTTTTGATTTGAAAG

GGATGGACAGTTAGTGGATTTGGAGGAGTATGTCTTCCAGGCAAAGAAGTTCCAGATTGGTTTGCATACAAAGATGAAGGTCACTCAATATTTTTAGAATTGCCTCAGTATAATAATTCCAATTTAGAAGGCTTCATTGTTTGCATAGTTTACTGTTCTTGTTTTAACAACACAGTCTCAACTGACCTTCCAAGTTTATCAGTCATTAATTACACAAAATCTTCCATTACAACCAACAAACCTCTTACCAATGATGTAATAATGTCAACTCAAGATCACTTGTGGCAAGGCCATTTATCTAACAAAGCCTTCAAGATGGAACCTGGCGATGAAGTCGAGATCATCGTTGATTTCGGTGCTGAAATCACCGTGAAGAAAATTGGCATCTCGCTTGTGTTTGACAAGTATGTCGATCAAACAATGTTAGAGTTTGCATCCACCTCTAATGATGATGATGTCGTCGTGGATAACCAAGATGAAAATGTAAGTGAAAAGGATGGAGAAGTTGGGAGCAAGAGAGGTTTTGACGAGAATGATGATGAAGGATTGAAAAATTCATACCAAATTCCCAAAAGGTTGAAGTGTGAGATTGATTCTAACATGAAAATTGATGAGGAGTAG

T4 (4-exons):

Same gene structure in other plant families: *Solanum lycopersicum* bacterial spot disease resistance protein 4 (Bs4) gene, Bs4-MM allele, complete cds

GenBank: AY438027.1

> Cla012434

ATGCTCCTTTCTTCTTCTTCTTCTTCTTCTTCTTCATCTCAAACTAGTAAATGGAAATACGACGTGTTCTTAAGCTTCAGAGGCGAAGATACACGTGGCGGCTTCACAGACCATCTCTACAAAGCCTTAATTCAAAAGGGAATTTTCACATTCAGAGATGAAGACGAGATCGAAGAAGGAACAGACATTTCTTCAGATCTGTTGGCCGCCATTGAGGCCTCCAGATTTGCAGTGGTTGTGGTTTCAGAAAACTACGCTTCTTCAAGATGGTGCCTCGAAGAATTGGTTAAGATCTTCGAATGCGAAGAGCGGGATGGGATGGCTGTTTTACCAGTTTTTTACAAAGTGGATCCTTCTCATGTCCGAAAACAGAGTGGAAGCTTTGGAGAAGCTTTTCTTAAACATGAATTCAGATTTGGGAAAGATGATGATAAGGTTCGAAAATGGAGGATGCTTCTCACCAAGCTTGCCAACCTCAAAGCTTGGCTTTCCCAATCTTG

GTAACTAACTATTTATCATTTTATTGGTAATCATTTGGTTTTTTTTTTTTAATAAAATCTATTTTTGCTCCATTATTTATTTATTTTTTATTATTATTATTATTTATGTTTTACATCTTTTTTAAGCGAAGAAAAAAAAAGTTTTTGTGAAATTTTAAAAATAAAATCAAAATTTAAAAATTTATTCTTTTAGTTTTCAAAATTAATTTTGATTTTTTTAAGACGATAGGCAAAAAGTGGATAACAAAGCATAAAATTTAAGCCTTATTTGATAATCATTTTATTTTTTGTTTTACGGTTTAAACCCATAAAAACTCATTTCCATCTCTACAATTGTTTTTTTTAACCTATTTTTTTACGAGTCTCTTATCTAAAATTTGAAATCTAATAGTAGTAATTTTTAAAAATGTGTTTTTTTTTGGAATTTGGCTCATAGTTTAACTTTTATATTAAAAAAAAGATGCAAATCATGGTAAAAAATTAAAAGAAATTAAACAATGCAAATCATGGTAAGAAATTAAAAGAAAATATGCTTAATTTTCAAAAACAAAAAACTAAAAGGAACTAAAAGTTGCCAATTAATGAGCCCTTAGAGATGGGAAGAGTGTTAACGTGTAATTTTGAAAACTAAGAAAGTAAATTTATGTATTTTCAAAATCAGAACCAAATTTTGAAAACTAAAAAAGTAAAATTTAAAAACCTTTTTGTTTTGTTTTTCCATTAGCTAAAAAATAAATATGAAAGATCATAGTAAGAAATTGCTAGAAAATAAAAGCAGTTTTCGAAAACAAAAAACCAAATAGTTACAAGACGAGGCTCAATATCTTCATTAATCTTAATTGAAATTGTTGGACTAAAAATAGGTTTAATAGATCCATCATTTTTGCTTTCATAAAATTTTTAATATAGTTTAAATGTGGTTGATAACTTGATAGTTGGTAAAGTTGACATGAGTATGAGTATGATATTTAAGCGAGTTGATAGAAATTTTGAATAGAATTTAGGCTACGGAAAAGATATTAAAATTCATCGTTCTAATTTCAGAAAATTTGGTACAGATTTTTTGCTTTTTTCTTTATCCTGTAGTTTTATTTGTCCTTTAGATTTCTTTGATCTTTATATACATTTTAGTGTTCGTGCTCAAAATTTTATTGAAATTTTCAAATACGAGTTTTGTTCAATGATTAAGGTATCAGTTCCTTTCCTTGATGTTAGCAGGCTTTTCCAATTAAAGAAAATGATTAAAATTCTTAGGTCAAATATATATTTTATGCATTTTTTTTTCTTCTTTTCTTTAATGTTTTTCATTTAGACTAAGTATATTATCTTTGATTAATTGATGCCTGTTAAATATTAGAGAGAAGAGTTGAATTAAATATCTTAAAGCACTAAACTACGTAAGCCTTTTCTAGGTAGCCAAAAACAATATAGTAGACTCCGTCAACAAACCAATCATATCAAAGTAAACTGGCCTCATATTAATTAATTATAGATGTCAAAGTTCTTGATTGTGATTGAACTGAAAAGTTTATTAATAATCAATTAGCATCTTTAATGACAATAAGCCAACATGTTTTTGTTTTATATGATAGTGACATTCCTTAACATAAATAATCAATTATATAATCATGTATTAGTCAGTAGCTATAATAAGGGCCCTTTTACCCATGAATTAGAATGAGAAAGGTTTGACTTTTGGGTTGTTGGCATAAAATTTGTAGGATTAAGAAACCAAACAAATAGGTTTGGAAGAGAAGCTTTAGAACAAATGTCATGGGTTTGAAAAAACATTTCACTTCAATCACCTTAGCTTCCTATCTCTTCATCTCCTACTTCTGATGAGTGGCTGTCAAAAATTGTGCATTGAGAAGAGAAAAAGAGAAGAAAATGAGGGTGAGACTAAAGAAGAGGAGAAAAAGAAAGAAAGCGGTTGCCAACAACAATGGTTGGTGACGGCTCTTAAGAAATTGTTTGAGAGCTAGAATGTAATCAGATTATTGGGAATCAAAACAGCGCTGGAATACGTGAGGTATCAAGGGCGGAATGGAAATGAGATCGTGAAACATGAAAACGATAGGAAAAAGGGTTTAGTTAAACACGGTAGGAATAGAATGAGTTTCGCTTGTTTCAAACACCCATTCCATTACAAGCCTCGAAACAGCCCCTAAAGAAATTAGAAAATTATTTAGTAATAAAAAATAGGTCACGTTGTCTGTTAGTAACCAACGAAAAACTGACAAAGAGACATAGTTTCGACAAAGTTACTTTTGTTTTGAAAATTTTAGTAGGAATTCAAGAATTCTTTTAACATGGTGAAAACCAAAATTGAGATGAAAAAACAGAGATCCAAAAGAATCTACCTATTGACTATCTATAACTACTTATTTTCGCGTGCTCTAATTTTTAGAGTATTTTACCTAAAATGGTAAATTTGAAGTGATATTTCAAAAAGATTTATGTATCTTCATCCATTTTCTTGTGATTAG

GTCACATGAATCAGAAATCATTGAAGAAATCACCACAACAATATGGAAAAGAATTAAACCGAGTTTGAGAGTCATTAAGGAAGACCAACTAGTTGGAATCAATTCTAAACTAAACAAACTTTCGTCACTTTTGAACACAAACTCAGATGATGATGACGTGATCTGTGTGGGAATACATGGAATGGGTGGCATTGGTAAGACCACAATAGCTAGGGTTTGTTACGAGCGAATTTGTGACGAATTCGAAGCTCATTGCTTCCTCTCCGACGTTCGAGAGAATTTCGAAACCTCCGGCCTTCCATATTTACAAACCAAACTCCTTTCAAGGATGTTTTCATTTAAAAACAATCACATATGGGATGTTGAAGAAGGCATTGCTATGATCAACAAAGCCACTTTTGGAAAAAAGACACTTGTTGTCCTAGATGATGTGAATTGTTCGGATCAAATCATGGGGTTGATTCCAAACAAAAAATCTTTTGGAAATGGAAGTATAATCATCATTACAACAAGAAATGCAGATTTACTTTCGAATGAATTTGGTGTAAAAAGAATTTTTGAAATGGAAGAACTTCAGTATGAGGAAGCACTTGAACTCCTTAATTTGAGGGCTTTCATGAAAACATGTCCAAAAGAAGGTTACTTAGAACATTCCAAGAAGATTGTGAAACATGTGGGAGGCCACCCTCTTGCACTAAAATTATTGGGGTCATCTCTAAGAAACAAAGATTTGAGTGTGTGGAATGATGTTATAGAAGAGGTTGAAGGAGGTGGGAATATTCATGATAAAATTTTCAAGTGTCTTAAAGTGAGTTATGATGGATTGGATGAGTGGGAGAAAGAGATATTTCTTGATGTTGCTTGCTTCTTCAAGGGGAAGAGAAGAGAAGTTGTAGAAGAGATACTAAATGGATGTGGTTTCTATGCCAAAAGAAGGGTTGAACTTCTTATTCAAAAATCTCTCTTAACTCTCTCTTATGACAATAAATTGCAGATGCATGATTTATTGCAAGAAATGGGTCGAAAAATTGTTCGTCATAAGCATGTTCGAGATCGATTATGGTGCCTCAAGGATATAAAAAGTGCG

GTAAGATATATATATACACACACATATATTAATCGATTTGTTTGGTTGGAATGACTTTCTAAAGTTCTCGAAATATGCAAAATAAAAGTATTTTTTAATGCTCAAACCCTCGTTTGATAACCATTTGATTTTTGGTTTAGGGTTTTTGAAAATTAAACTTATAAACACTATTTCTACCGGCGTGTTTTGTTATATACTTTCTACTAATGTTTTAGAAATTCAAGCTAAATTTTGAGAGTAAAAAATAGCTTTCAAAATTTTGTTTTTGGTTACAAATTTTGTCTAGGAAATCTCAAGCGTTCTTTAACAAAGATGAAAATGATAATTGAAAAATTGAGATGAAATAAGCATTATTAATATAAAAAATATAAAACAAAAAATGCGAAAGTCATGTTTGGTAACAATTTAGTTTTTAGTTTTTGAAGTTTTATGCTTGTTTTGTTTCAACTTTTCAATAGGCTTTTCACATTTTTGAAAGAAGCACCTGAACTTCTAGTTAGATTCTAACAACAAAAACAATTTTATGGAAACTTAGTAGAAAGTGGATTGCAAAGCATAGAAACTTATGTGTACAACTAGTGTTTATAGGGTTATTTTAAAACACTAGAGACCAAAAAAACCAATTGACCTTCAAAACTTATTCAAAGTAAACTCTGTATGTACATTTGTGATCTCTCTCTAGTAACAAATTTGTTTGGTGTTGCATTTGGTATCTTATGGTTTCAG

GTTCCAGAGGCATTGGTCCAAACCATATTTTTCAAGTCCAGTACAAGAAACATGGTTGAATTTCCAATTTTGTTTTCAAGGATGCACCAACTTAGGCTGCTTAATTTTCACAATGTGAGACTTAAAAATAAGTTGGAATATTGCATTCCAAGTGAGTTAAGATATTTGAAGTGGAAAGGATATCCTTTGGAGTTTCTGCCATTCAATAGTTCTGAAGAATATAAGCTTATTCAGCTTCACATGTGCCATAGCAATTTGAAACAATTTTGGCAAGGAGAAAAG

GTAACAATATTATACTATATATCAAGTGTTGGAAGTAAATGATTTTTATTTTCTGCTATATATATTCATTAGTTCAACTTGTGGTCAAAGATCTTTTTTAATTCCATTGAACCATGCTTTAAGTTGGCCATATTATTTTCACATGAATAGATTGTAGCATATACATCCACGCACAAGCTTTATCAAAGTTAAACAATCTAATTTCCTTTAGGGTAGTCATATAGTTAGAAAAAGAATATCAACTTCTTTTCCGTTTTTTAAAAAATACGCATAGAAGTATTCCGTTTTTTAAAAAATACGCATAGAAGTTGCAATATTACTCTAGACTACCCTTGCATACGCGTTTCGAAACTATTATTGGAGTAGATATTCATTAAAATGTAGGACAAAAATTAGGAGTTAGAATGATATGTGGTGGTGACTTGAAATGGTGTGGTACCCCAATTTCATTTCAAGTATGGTCATTGATATAAACTATTTTAAGTCCTAATTTCATTCAAAAACTCTAACCGATTATCTAGTTTTGAAATGTTTATGAAAGATAAAATATTGTTATTACCATTTTTGAATGAATAGAGATTTTCTTTGAAACAGCTGGCAACGTATTGGGGCATTTCTTTTAATTTTGCAGCCATAAAAAGAAAACTCTTAACATTGTGGACTATATAACGTAGATTTAGCCTTAAAAAGTTGCATATTTTTAAGCACATACATCCCGTATAAGAATGATAGTTACATATCATAACAAAGACTTCATTTGTGACAAGTGACTTCTTTTAGGTTGCTTTTTTCTTTTTAATTATAAACAAATTAGTACCATTTTCAAGTGTTGTTCTTTTGTTTAACTTCTATCAGTCCTTACTTGTAACAG

CATTTAGAGGAGCTGAAGTATATCAAACTCAATCATTCTCAAAAGTTGTCCAAAACTCCAAACTTTGCAAGAATTCCTAATCTCAAAAGATTAGAGCTTGAAGGTTGCACAAGATTAGTCAACATTCATCCATCCATTTTCACTGCAGAAAAACTCATTTTCTTGAGTCTGAAAGATTGCATCAATCTCACCAATCTTCCGTCTCACATTAACATCAAGGTTCTTGAAGTCTTGATTCTCTCTGGTTGCTCAAAAGTCAAGAAGATCCCTGAATTTTCAGGCAACACAAATAGATTACTCCAACTCCATTTGGATGGCACCTCCATATCAAACCTACCTTCATCAATTGCAAGCTTGAATCACCTAACAGAGTTAAGTTTAAACAACTGCAAAAAGCTAATCAACATTTCAAACACCATGGACAAGATGACATCTCTCCGAAGCTTAGATCTTTCCGGATGTTCGAAGCTCGGAAATAGAAAAAGAAAGGCAGACGATGTCGAATTGATGGAGCTCGACGTGAGAGGAACCGCAAAAAGAAGAAGAGACGACGATGATAACAATATCTTGAAAAAGATGTTCTTTTGGTTATGCAAAGCTCCAGCTAGTGGCATTTTTGGGATCCCATCACTGGCTGGTTTATACTCTCTTACAAGACTAAACTTGAGGGATTGCAAACTTGAAGAAATCCCACAAGGGATTGAGTGTTTGGTGTCTTTGGTAGAGCTCAATTTGAGTGGCAATAATTTCTCTCATCTTCCAACAAGCATATCAAGACTCCATAACTTGAGAAGATTGAACATTAACCAATGCAAAAAGCTTGTGCATTTCCCAGAGCTACCTCCAAGGATCTTGAGGTTGATGTCAAAAGATTGCATTTCATTGAAAGATTTTCTAGATATTTCAAAAATTGACCATTCATATTTCATGAGAGAAGTGAATCTTTTGAACTGCGACCAATTGGCTGACAACAAAGGGCTCCATAGATTGATCATTTCGTGGATGCAGAGGATGTTCTTTCGAAAAGGAACATTCAATATCATGATTCCAGGGAGTGAGATTCCCGATTGGTTTACGACGACGAAAATGGGATCTTCGGTATGCATTGAGTGGGATCCAAATGCGCCAAACGCCAACATGATTCGCTTTGCGCTTTGCGTTGTTTGTGGTCTGAGCAACGAAAACGACATTGTCAATGTTCCGTCGTTCGCAATTATTGCATCGGTGACTGGAAAAGATCGCAACGACACGAATTTGAACAATGGAGACCTCATGGTTAGTGGATTCACTGTTTCAGGGATGAAGAAGTTAGACCATATATGGATGTTTGTTTTGACACGAACTAAGAGTCTGGTAAGAAAGATTAGCAAATGTAAAGAGATTGAGTTTAGATTCTTGGTCCAAGCTAATTATAGTGAAGCTGTTACCCCAAATGTCAAACTGAAGAAGTGTGGAGTTAGTTTGATAAATATGGAAGAAGAGAAGGAAGCCATGAAACGGTATGCTTCTTATATCATCTTGAAAAACAAGATGAAGTCATTGTCGAAGTATTAA

T5 (4-exons):

Same gene structure in other plant families:

Phytozome Transcript name : mrna34024.1-v1.0-hybrid [*Fragaria vesca* ]

The sequence was re-annotated and added in this file.

> Csa009602

ATGGCTTCTCCAGCAATAATGGAGAGAAGAGCTTCAATTAAATCCTTATCTCCTCCTCCCTATTCTATCTCTCTTCCTCTTCCTCCCTTACGAAGATATGACGTTTTCCTCAGCCACAGAGTTAAGGATACCGGGAGTAGTTTCGCAGCTGATCTTCATGAAGCTTTGACAAACCAAGGAATTGTAGTTTTCAGAGACGGCATAGACGACGAAGACGCAGAGCAACCATATGTAGAGGAGAAGATGAAGGCCGTGGAAGAATCGAGGTCTTCGATCGTGGTTTTTTCAGAGAACTACGGGAGTTTTGTTTGCATGAAGGAAGTAGGGAAGATTGTAACGTGTAAGGAGTTGATGGATCAACTGGTTCTTCCTATATTTTACAAAATAGATCCAGGCAATGTGAGGAAGCAAGAGGGGAACTTTAAGAAGTACTTTAATGACCATGAAGCCAATCCTAAGATTGATATTGAAGAAGTTGAGAACTGGAGATATTCTATGAATCAAGTTGGCCATCTCTCTGGATGGCATGTCCAAGATTCCCA

GTTAAGTAATAATTATATATTCACTGATCTCTTCAGCTTTTTCTTTCTAATTTACGTATGTTTTGTAGTTTATAATTACAAAGTTTACAAAAGGAAGCACAAATGTGTTTTTAAACGTCATCTCTAAAATTTAGATTTTAAACCAAATTCCAAAACAATAACAATATTTTCAAAATTTAGAGATGATTACTAAAATTGAAAAGTATATATATGTATATAG

GTCTGAAGAAGGGAGCATAATCAATGAAGTTGTGAAGCATATATTCAACAAATTGCGTCCTGATTTGTTTCGATATGATGATAAATTAGTTGGAATTTCCCCAAGATTACACCAAATAAATATGCTTTTGGGAATAGGTTTAGATGATGTACGCTTTGTTGGAATATGGGGAATGGGTGGAATTGGCAAAACTACAATTGCTAGAATCATTTACAAAAGTGTTTCTCATTTATTTGATGGATGTTATTTCTTGGACAATGTCAAAGAAGCTTTGAAGAAAGAAGACATAGCTTCATTACAACAAAAGCTTCTAACAGGAACTCTAATGAAAAGAAACATTGACATCCCTAATGCTGATGGAGCTACATTAATTAAGAGAAGAATAAGTAATATTAAAGCTCTTATAATTCTTGACGATGTCAACCATCTAAGCCAACTTCAAAAATTAGCCGGCGGTTTAGATTGGTTTGGCTCAGGAAGTCGAGTCATCGTTACAACGAGAGACGAACATCTCCTAATTTCACATGGAATCGAAAGACGATACAATGTTGAAGTGCTGAAAATTGAAGAAGGTCTTCAGCTTTTTTCACAAAAGGCATTTGGAGAAGAGCATACAAAGGAAGAGTATTTTGATGTTTGTAGCCAAGTTGTAGACTATGCTGGAGGACTTCCATTGGCAATTGAGGTTCTTGGATCTTCTTTACGTAATAAACCAATGGAGGATTGGATAAATGCAGTGGAAAAGTTGTGGGAAGTTCGTGATAAGGAAATTATAGAAAAGTTGAAAATTAGTTATTATATGTTGGAGAAATCTGAACAGAAAATTTTTCTAGATATTGCATGTTTTTTTAAGAGAAAGAGTAAGAAACAAGCAATAGAAATTCTTGAAAGTTTTGGATTTCCTGCTGTTCTTGGACTAGAAATATTGGAGGAGAAATGTCTTATTACTACACCACATGATAAGCTACATATGCATGATTTAATACAAGAAATGGGCCAAGAAATTGTTCGCCAAAACTTTCTGAATGAGCCCGAAAAGCGAACTAGGTTGTGGCTTCGTGAGGATGTCAATCTCGCACTAAGTCGAGATCAG

GTAACTATATATATATATATTGTTTTAACTCATAGGAAAACTTGGCATGGGAATGATTAAATAAGTACATTATTATTTTCATCTAAATTAGTTTCAACTTTAATTTGCCTCTAACATTTTAAACTTCATCTGTTATGAATTAG

GAAGGAGAATCACATTTGAATGCCAAAGCCTTTTCAGAAATGACAAATCTAAGAGTATTGAAATTGAACAATGTTCATCTTAGTAAAGAAATTGAATATCTGTCTGATCAACTAAGGTTTCTCAATTGGCATGGTTACCCTTTAAAGACCTTACCATCAAATTTCAATCCCACAAATCTATTGGAGCTTGAGTTGCCAAATAGCTCTATTCACCATCTTTGGACTGCTTCAAAG

GTACATCAAAACAACAGTAGTAATTAAATAAGTACATTATTATTTTCATCTAAATTAGTTTCAACTTTAATTTGCCTCTAACATTTTAAACTTCATCTGTTATGAATTTTTTTCTTAAATCCCAAACCAAAAATAGAAGAGGATAAATTGGAAATTTCAAAGTTTTATGCAATTTTTATCATTCTGCATGAATATTTTGCATAATTTGGATTGTTTTTTTTTTTTTTTTTTTATCAACTTTGGCTTTCGAGTAAAGTGCAATGAGTGAAAAGTATTTATGGATCATAAGGATATTTTAACCATATTTGCAAATTATGATTGAAATTTTGCTAACAATGAAATTTTGTGGTATTGTATTTTATTTGCAGAGCATGGAAACATTGAAAGTGATAAACCTAAGTGATTCTCAG

TTCCTATCAAAGACACCTGATTTTTCAGGTGTTCCAAATCTTGAAAGATTGGTTTTAAGTGGCTGTGTAGAACTTCACCAACTTCACCACTCTTTGGGTAATCTAAACCATCTAATTCAATTGGACCTCAGAAATTGCAAGAAATTAACAAACATTCCTTTCAATATTTCCTTAGAATCTCTCAAAATTTTGGTTCTTTCAGGCTGTTCAAATCTCACCCATTTCCCAAAAATCTCATCAAACATGAACCATCTACTAGAGCTTCATTTAGACGAAACATCCATAAAAGTTTTGCATTCATCAATAGGACATTTAACATCACTTGTTTTATTAAATCTCAAAAATTGCACAGATCTTCTAAAACTTCCTTCCACTATTGGCTCTCTAACATCTCTAAAAACCCTCAATTTAAATGGCTGCTCAAAACTTGATAGTCTTCCAGAGAGTTTAGGAGATATTTCTTCCTTAGAGAAGCTTGATATTACAAGCACTTGTGTAAATCAAGCTCCAATGTCATTTCAGCTTTTGACCAAACTAGAAATACTAAACTGTCAAGGACTATCTCGCAAATTTCTTCATTCATTATTCCCTACTTGGAAGTTCACTAGAAAATTCTCCAATTATTCTCAAGGGTTGAAAGTGACAAATTGGTTTACATTTGGTTGCTCTTTGAGGATTTTGAATCTGAGTGATTGTAATTTGTGGGATGGAGATTTACCTAATGACCTTCATAGCTTAGCTTCATTGCAAATTCTTCATCTAAGTAAAAACCATTTTACCAAATTGCCTGAAAGCATCTGTCATCTTGTGAATTTGAGGGATCTATTTTTGGTGGAATGTTTTCATCTTCTGAGTTTACCAAAACTTCCGCTAAGTGTTAGAGAAGTAGAAGCAAGAGATTGTGTTTCACTAAAAGAATATTACAATAAAGAGAAACAAATTCCTTCAAGTGAATTGGGAATAACATTTATCCGATGTCCTATTTCCAATGAACCATCTGAAAGTTATAATATTGATCAGCCACACTTTTCTGCCATTCACGTAAGGACAACGACTCAACGATACATTGAGGTAATTAACTTCTTTACTTTTCTTTTCTCATCATAA

T6 (4-exons)

Same gene structure in other plant families:

hypothetical protein PRUPE_ppa020421mg, partial [*Prunus persica*]

GenBank: EMJ20597.1

>MELO3C021852T1_full_length_(reversed)

ATGATGGATTCTTCAAACTCTTCTTGGTCTTACGATGTGTTTCTAAGTTTTAGAGGTGAGGATACTCGGAAATCGTTTATGGGTCATCTGTATGAAGCTTTGGTGAGGGCTGGAATCAATGTCTTTAGAGACACCGAGGAGCTGAAGAGGGGGATGCAAATTTCTCAAGAACTTTGTGAAGCAATCAACGGATCAAGGTTTTTTGTTGTAGTTTTTTCCAAAAATTATGCATCTTCTAGTTGGTGTTTAGATGAGGTTGTTCAAATTATGAGTTGTATGGAAGGTGGCAAAGGGCAAATGGTTTGGCCACTGTTTTATCATATTGATCCTTCTGAAGTTAGGAAACAAACAGGCTTATTTGGAGAAGCATTTATTGTACATAAAGAAAGATTCAAGGAAGACTTGAAAAAGGTAGAGAGATGGAGAGAAGCTCTAACTAAAGCTGCCAACTTATCAAGCTGGCATTTGTATGACAG

GTAACAAATAATAACGTGGAATTCTATCTCAAAACCAACGAAAAATAATCTATCTTGTAAAGATTACAAGGTCTCTTGATTTTTCCAGTGTTATTCTAATTATTCTTTTATTTATTATCATTTTCTATATCTCACTATCTATATATATTGTGGTTTTTTTTTAATGTAG

TTCGTGTGATGAGGCTACTCTTGTCAAAGAAATTGTTCATGAGATATCCACTAAGCTTAACAAGACGCTCTTAACAATTGCCAAGAATCCAGTTGGGATTGGTTCTCATATTGAGAAGCTAAACTCCGCATTAGATTTAGAGTCCAATGATGTTCGAATGATTGGAATTTATGGCCTTGGTGGAATAGGCAAGACAACTGTTGCAAAAGCTGTTTATAATTGTATTGCATATCGGTTTGAAGGCTGTAGTTTTCTTCCTAATATAAAAGAAAAATGCAACTTCTCGAGGGATGATGAGCTAACTAAATTGCAAGAATCTCTTCTTCAAGACATCTTGTTGATTAAAATGCATAGGTCTATAAGTTTTTCCGATGAAGGAAGTAATGTGTTGAGGCATAGACTAAGAAACAAAAAGGTACTTATTGTTCTCGATGACGTGGATCATTCAGATCAGCTAGAAAAACTAGCAGGACATTTGAGTTGGTTTGGTTCGGGAAGTAGGATAATGATAACAACAAGAGATTTTCATCTTCTAAATAAACTCAACATTAAGTGGACCTACAAGGTTGACAAGTTAAATCATAGAGAAGCACTTGAGCTATTTTGTTGGAATGCATTTAACAATCCTGTTCCTGATGTGAGTTTTGAAGAGCTTTCAAATGTTGCAGTAGAACATGCAAATGGACTTCCTCTTGCCCTTGCTGTGGTGGGAGGTTCTCTTTGTAATTGTAGTAAACTTGAGTGGGAAAGCCAATTGAAGAAGTTGAAAAAGATTCCAAATCAAGATGTTCATAATAAACTTAAACTAAGTTTTGATAAACTTGGTGATCTTCAGAAGACAATTTTTCTAGATATTGCATGTTTCTTTAGAGGGTATCCAACAGAAGATGTGTACTCTTTTTTAGATGCTTGTGATTTCTACCCCCGTTCTGAAATTGAAGTGCTTATTAAGAAATGTCTTATATATATTGAATACAATAGATTCCAAATGCATGACTTGATTCAACTAATGGGCAAAGAGATCGTTCGTCAAGAATCTCCTTCAGATGTGGGAAGACGTAGTAGATTGTGGTATCACCAAGATGTTCTTCAAGTTCTACAAGAAGATGAG

GTATGATTTATTCCTAGCCTCGCACTTAGATAAATAGAATATACCTTTCACATATCTTTGTTTGGGTGCTTAAAGCACTGAGTGAATTTAGAAGCCTGAAATTAATATGTTGAAGCTATGAAGTCCGGTATGTAAGGTTGTGAAGATGAGGTTTGCTGATAATTTGCAAAAGTGAAAAAAAGGAAGATGAAGTGTCTCGGTAAATATGCAACTTCTCACCATTGGAGTTGAGTTATTTAGCATTCACTTATGAAGTCCGTGGCAAAACACCCTTTGTATATGTTTCACAAAATCATTACTTTTCATTTATACTTATTTATATATATAGTGTGAGGCCCATATATGTTATCTACAATTAAAGAACTTTTGCTTGTCAGCTAGATATATTGAATGGCTTCTTGTGTTTTCAG

GCAACAGAAAAAGTTGAGGGGATAGTTTTAGAATTGCCCAAAGCAGAAGAAGTAGTGTGGTTGGATGCTAAGTCATTTTCAAAAATGACAAAATTGAGGTTGCTTATAATACGGAATAATGTTAATGTTATTGGGACTCCTCGATGCTTTTCTAACTACTTAAGATGGTTTGAATGTCATCGTTCTTCATCTTCTCTGGTACCATATACTTTTAATCCAAAAAACCTTGTTGTATTGGTCATCTCACATTATCCTATAATGCACCTCGTAGCTAACCTCAAG

GTAATGTGATTATTACTTTGATCTATATATACGTTTTCTATTTATCTTTGATTGTCAAGTTTTTCAACCAGAGAAAAACAAAAATAAGAATTAGAAAATAAAAACCACAACCCTTAGGAAGGATTTCTACTAAAATTATTAGAAAATCACAAAAAAAAATTCTCTCAGGATCTCAATCACGAAACTACTCTTTTTCAATGTTTTTACAACAGTCACACCATTATTTTCACCCTCTAATTTAAGAATACGAAAAAATAATTTAACTAGAGTTACCCTACATAGGATAGATGTTAAAATGTCTTGACTGTGTTATATGGCCTTTGAACAGCCAAGTACGAGTTTAGAGAAGTACAAAGTATAAACCTTTAACTCTAGAAAAAGGCTCTACTATTTTGTATCTTGTAACCTGCTCTCTTGTAAAGATGTTCTTCTTCTAACTAATGATGTACATAACACCCAAATAGTGAACCACATAAAGTAGCGGTGGGTTAGATTGTTTACTATTCAATGTTTCCTTATTCTTTATTTGTCAATTTCCTAACAGTTGATTTGTTTTGTTTGACAG

TGTTTTGAAAGCATAAGGCAGTTAAGTTTAGAAAATTGTGAAATGCTGACTGAAATTGGCGATATATCGTTAATGCCGAATCTTATCAAATTAGAAATAATCAACTGCTTTAACCTCGAGTGTTTTTCAAGTACGGGATTGAAATCGAAAACTCTTCAATGCCTTCATCTTAAATCTTGCTACAAGCTGAAAAAGTTCCCTGATATTGTTGAAGAAATGGAATGCCTTCAAATACTTAGTTTGGAAGAAATTGGTGTAACAGAACTACCTAAATCCATTGAAAAACTAAGTGGTCTTAGGCATCTAGAGTTGAGCTTGTGTGAGGACTTGTTGCAGCTTCCAAGTATTATCTACGACCTTAAAATGCTTAATTCTCTTAAACTGAGTAAGTGCCTCAAGCTACAGGAATTTCCTCAGCCTTCAAACTCCAGTACTAATGTAAAGAAGTTCTTACCGTTAAGGTTGTTGAATCAAAAGAATCTCCTTTCAACTTCAGTTACCTACATGATTTATTTCAACTTGAATTATATTTGTAAATGAG

T7 (4-exons):

Same gene structure in other plant families:

*Solanum tuberosum* nematode resistance-like protein (Gro1-2) gene, Gro1-2-P40 allele, complete cds

GenBank: AY196153.1

> Csa009775

ATGCAGAGTTCATCATCGTCTTCTTTGGATCGTCCTAAGATGAACTATGATGTGTTCATAAGCTTTAGAGGTAGAGATGTTCGTCACACTTTTGCAGGATATTTGTACGATGCTTTGAATCGTTTGGGGATAAAAGCTTTCCTGGACAACAAGAGGTTTCTAATTGGAGATGATCTTCATGACTTATTCAAAATAATCGATGAATCAAGATCAGCAATTGTTGTTCTTTCAGAAGACTATGCTTCTGCTAAATGGTGTTTGAGAGAGTTGACTAAGATAATGGATTCCATGGGAACCTCAATGGAGCGTGTCCTTCCTGTGTTTTATCATATTGATCCATCAATTGTTAAAGATCAATCTGGAACTTTTAAGACAAGTTTTGATGAACATGAAGCCAATGTTTTAAAGGAAATTGATAATCAAGAGAAGGAGAAGCGCTTGAAGGAACTCCAGAATTGGAAAAGTGCACTGAAGAAAATTGGCAATCACACTGGAGTTGTCATCACTAAGAACAG

GTAAATTTCACACGACTATTTGTTTTTTCTTAAAAACTATTATCTCTTTTGATTGTTTTGATTTGGTCTCTTGTCAATTTTGAATCTGCTCCCTATTCTCCTATGTATGTGTGAAGATAAGTTGAATATTTGTATTCGGTTGGCTTATTCGGATATGAGTTAGATATTTGTTGGTTGTTCCAAGAGACTCATATTAGCACGATCAATTTAGGATTAGAGAGAAAGTTATAAAATTATGAGATGACTAAATGTTAAAGAAATTTCAGCTATCATTTGTATTAAAATCATAATATCATATATGCATTGATCAACTTCTGAAAATTGGATATACTATTTACAGCATTCACTTAACATTAAGTTTAATTATATTTTGCAG

TTCTGAGGTAGATATAGTAAATAAAATTGCAAGTCAAATATTCGATGCATGGCGTCCTAAGTTGGAAGCATTGAATAAGAATTTAGTTGGAATGACATCCCGATTGCTCCATATGAACATGCATCTTGGTTTAGGATTAGACGATGTACGCTTCGTTGCGATAGTAGGAATGGGTGGTATTGGTAAAACAACTATTGCTCAAGTCGTTTTTGATTGCATTCTTTCAAAGTTTGAAGATTGCTGCTTTCTAACATTACCTGGAGGTGATTCAAAGCAAAGTTTAGTGTCATTACAACGGGAAATGCTTTCTCAAATTTTTCATAAAGAAGATTTTAGAATATGGCATGAGAATCATGGAGTAGAGATGATTAAAAATCGACTGAGTGGTAGAAAGGTTCTTATTGTTCTTGATGGCATCGAAGAGAGAAGGCAGTTAGAAATGTTGGCTGGAAGCATCGAGTGGTTTGGTCCTGGAAGCAGAATCATCATTACAACTAGAAATAAAGGATTATTGTGCCATCCTAATTATGATGAAATGAAAGTATACAATGTTGAAGAACTAGATCATGATAGTGCCCTTCAACTCTTTTTGAAGCATGCATTTGGTAGTAATCATCAAAACAATGACAGTTTCATGGATCTTAGTAATGAGATAGTTGAGAAGGCTAAAAGACTTCCATTAGCTTTAAGAGTGATTGGATCTTCTTTGTATGGTAAAGATATTACAGTATGGAGAGAAACGTTGAAGAGGCTGATCAAAGTGGATGAAAGAAATTTTTTTGATGTATTGAAAATAAGTTATGATGGATTAGGAGTTGAAAGCCAACAAGTTTTTCTTGACATTACATGTTTCTTCAATGGAAAAAATGAAGATAGAGTAATTGAAATATTAGAGAGTTTTGGTTATAGTCCTAATAGTGAAGTACAATTACTGATGCAAAGATGTTTAATTGAAGTTTCACACAAGAAAATATTGGTGCATGATTTAATTCTTGAAATGGGTCGAGAAATTGTGCGTAAGGAGTCCCTCACTCAAGCAGAAAAACAGAGTAGGATTTGGCTTCATGAAGATCTTTACTGCAGGTTTGCTGAAAAACAT

GTAAGAAAATTGGTAATTTTTGTACCTAATATTCTGGCCAAGCCCACTAATTCTAACAATTATTAATGTTCTTTTTTAATTATTGTTTGTTAG

GACTTGATGCATATTCAAGGGATAGTTTTAAGTTTGGCAAAAGAAATGGAAGAATCAATAGAATTGGATGCTGAATCCTTTTCAGAGATGACCAAACTAAGAATACTGGAAATCAGTAATGTGGAGCTCGATGAAGACATTGAATATCTCTCTCCACTCTTACGGATAATTAATTGGCTTGGCTATCCTTCGAAGAGTTTGCCCCCAACGTTTCAATCCCGCTATTTGTTTGAACTACTCTTGCCTCATAGTCACCTTTTACGAATTTGGGATGGAAAAAAG

GTTTGCTTTTTAAAAACCGTTTTTGTTTTTAGTACTTTTTAGTTTGTGTTTGATTTCAATCTGGTCCCTATAGTTTTATTTAATATACATTTTTTTTTTCTTAACAG

AGATTTCCAAAGCTGAAATTAATTGATGTTAGTAACTCAGAACACTTGAGGGTGACACCTGATTTTTCTGGGGTTCCAAATCTTGAGAGATTGGTTCTATGTAACTGTGTTAGACTGTGTGAGATTCATCCCTCCATCAATTCCCTCAACAAACTCATTTTACTGGATTTAGAGGGTTGTGGTGATCTTAAACATTTTCCAGCAAATATAAGATGTAAAAATCTCCAAACACTCAAACTTTCTGGTACAGGTCTTGAAATTTTTCCAGAGATAGGCCATATGGAACATTTGACTCATCTTCATCTTGATGGATCCAATATAACCCATTTTCATCCTTCAATTGGGTATCTAACTGGCTTAGTTTTCTTGGACCTATCCTCCTGTTTAGGTCTTTCTAGTCTTCCTTGTGAAATTGGTAACTTGAAGTCTTTGAAAACCCTCCTTTTGAAATATTGTAAAAAACTTGATAAAATCCCTCCAAGCTTAGCAAATGCAGAATCCTTGGAGACACTTTCTATTAGTGAAACCTCAATAACCCATGTTCCACCAAGCATTATTCATTGTTTAAAGAACCTAAAAACGTTAGATTGTGAAGGACTATCACATGGAATTTGGAAGTCATTGCTCCCCCAATTCAACATTAATCAAACAATAACCACTGGTTTGGGGTGCCTCAAAGCTCTAAATTTAATGGGTTGCAAACTTATGGATGAGGACATTCCTGAAGATCTCCATTGCTTTTCTTCATTAGAAACACTAGATCTCAGCTATAATAACTTCACAACACTCCCTGATAGTCTTAGCCACCTCAAGAAGTTAAAGACATTGAACCTGAATTGTTGCACTGAGCTTAAAGACTTACCAAAGCTTCCAGAAAGTTTGCAATATGTAGGAGGAATAGACTGCAGATCGATGTCAGAACGATATTATAACAAAATTTTGCTTATCCCTTCTAGTTCTGGGCACCAACTTTACCTTACTTTTATCATTCCTTCCAAGGATGTGGATGTAGAATGTGACATGAATGAGTTCCAACATTCGATATTTACTCGAAGATCATTTGAGGTATGCATAAAAACATTGAAATATACTGCCTTTTGA

N1 (1-exon):

Same gene structure in other plant families:

RCOM_0751360 leucine-rich repeat containing protein, putative [ *Ricinus communis* (castor bean) ]

Gene ID: 8260632, updated on 11-Aug-2012

>Csa002698

ATGGTTGGACTTCTCGACAGTGTGGCCGGAAATCTGCTCGGAAGGATAATCGAAGCCGCCGACCGACTAGAGTTTCGTGCTATCCAAAGCGAATTGAAAAACCTCGAAACAGATGTGTTGAATCTTAAGGCCAGACTCCGAGACGCCGAGGAGAAGCAGGCTAGTAATTGTGAACTCAATGAACTGCTTAAAAACCTCAAAAATGTGTTTTCAAGGGCAGACATTGCAATTGAGGAATTGGAATGCGATTATTTGAAGTGGAGAGTGCAGAATCGAAAGAACGACGTTGACGATAAGGGATGCCAGTTCTCTTCTTGTTTCTCCTCCAATTTCCTCATTTCTCCATTTAATACCGGCAGTAAATTCCAGGAAGATCTTAAAATAATTACCTCCGAATTACGTTCGATTGAGAAAGCCATGTCTAAATTCTCTCTGGTTGAAGATGAAGATGAATATATAAAAAAATTGAAGGGTGAAATGACTTTGCGGACCTCCATTACTGGTTCGCATGCTTTCGCTAGGCTTCTGCGCTTGAGGAGAGAGGCGATTCTCTCTAATGTAGATTCCATTTTTGGTAGAGATAAAATACAAGAGAGTATCATTAAGGAACTTGTGAATGATGAACAAAAATCTCCCCGTATTCTTTCAATCCAAGGAGATGGAGGGATGGGAAAGACGGCTCTGGCCAAGTTAGTCTACAATGCAGACGAAGTGTTTGATCATTTTGACAAGAGAATGTGGGTATGCGTTTCTGAAGATTTTGATATCCGGAGAATCTTAAGGGAGGTTCTGATGTCTGCAACTGGAGAAAATGTTACCACTGTTGCCTTAACCGAAAGTCGTTTACGAATCCGGCTCCAGCGGTACTTTTTTGGCAAAAAAATCTTGCTTGTTTTGGATGATTTTGGGAATTTGGATCCCGAAAGAGTATCAGAACTGAAAAAAATCGTGAAGATGGGTGTTGGTGGCAGCAAGATAATGATAACCACTCGCAGCGATGAAACTCTAAATGTTGCTACGACACACAAGATTGACAAACTCGACGAGACGATATCTATGCAAATATTCGAAGATACATATGGAAGCGAAGGGCTTAGCGAAGGGCTTAGAGACGATTTGTATCTCAAAAACCTTGTGGCAGAATGTGGAGGAGCTCCTTTGGCAATCAAATGTTTGGCTGGACTGCTCTCTTCAAAACCGAGCGATGGTGCTAAGAGTCCAAATGTCAAGGACTTGAGTGAGAAATGGAAACAGGAAGAGGCAAACAACGGTGGTGGCGTTTTATGTGCACTAAGACTGAGTTATGATCTAATGCCATCTTATTTGAAACCTTGTTTTCTTTGCTTTTCAGTGTTGCCGAAAGATAATGTGTTCTTCTCATTTGAGCTAATCCAGTTATGGATGGCACAAGGAATCCTTCCTTCGGGTACCAAAGATAATCCTGAAGAAGTTGGGGAGAAATATTTCAAGGAATTTCGGGATCGCCGTTTACTCGTTGATGTTGAGGAGCACACTCTTGGATATTGGTTCAAAATCCATAGCCTTGTACATGATCTTGCAGTCCAAAAGGCTACGGAACAAAAGAACCTCGGAAATTTTCATATGCTTTCATTTGTCGATTGCGACAGCATCCCTTCGTCGACAAACTATGATAACACTCGTTTTATTTCCATTCCCGTGGTAGGAGGTGCGGGACCAAATATCAATAGTGACCTTTTCAAATGCATCACCCAGTTCAGGCAGCTAAGGTTTTTGTACTTGTGCAACTCTTCTCTGGAAGAAATTCCAACCTCCATCGACACGCTGAAACATTTGAGGTGTTTAGATTTGCGAGGGAGTCAACGTCTGAAGAGGTTGCCAGAATCAATTTGCAAACTACAGAGCCTACAGACTTTGGTTCTTGCATTCTGCTCAGAGCTTGAAGAGCTTCCCAGAAACATAAAGAACTTGATCAGCCTCAGATTCTTATGGATACAAACAAAGCAAGCCCGCTTGGAAAAAGATGAAATAGGAAGCTTAACATCCCTTCGTTTTCTCGCCATTGGAAGGAGTGAAAACTTGACTCACTTGTTTGAAGATATCAACAAACTCAATTCCCTCAAAACACTGATCATTTATGAGTGCAAATCGCTGCTAACACTGCCAAAAGGCTTGGAAAACATGAAATCTATATGTAATATGGGAATATGGGAATGTGATCGGCTGAGATTTACATTCTCACTGGCTTCACTTCACCTCAAGAAACTGATACTCAGAGAACTTACAGCAGTGTCCACTTTGCCTAATTGGCTGTCCAATTTGGATGGTACTTTAGAAGTGCTAGAAATTGGAGAGTTCCCCACGCTAAGAAAATTGCCAATCTGGCTTTTAAACTTTTGGGAACTCCGAATTCTTGGGATCTCCAACTGTCCTAAGTTGAAGCATGATTCCTTCCCTCCTGAGCTAAATTATTTTTGTGATAAGATTGAGGAGTTGAGGATCACATTTTGTGGGTCTTTGAGCAAGTCTTTGTTGAAAAAAAGCATGAAGGAAATTGAACCTGAAAGCCGGGTAATCTTTTACATCCATACCATTTATGTGGACTCCAAAAGAATGACGCCACCAGTAGAATCAACAGACGAACCTAAGGAAGCAGAGACAAAACAGGATGATGCTTATAACAATGCAAGTCCTCCTGGGACTGAACAACCTTCAAAGACTAAACATGATGATGCGAATAACAATATGAGTCATCCTGGGATTGGACTACTTTCAGAGTCAAAACAGGAGCATACAAATAACAATATAAACGAGATTGAGACTGTTAAGGTTTGTTTGGGTGATAATGACCATGCTGAAGCTCACCAAGCTATGGTCACTACATATGAGGGTTTCTGA

N2 (1-exon):

Same gene structure in other plant families:

POPTRDRAFT_569471 nbs-lrr resistance protein [ *Populus trichocarpa* ]

Gene ID: 7485214, updated on 25-Aug-2012

>MELO3C009694

ATGGATGCGGAAGAGAAGCAAGAACATAGTCGTCATCTACGTAATTGGCTAAAGGAGCTTCAAAATGTATTTTTCCAAATTGAGGGCTTCATAGATGAACTCAAAGAGGAAGTCTATAAAACCGAGGGTATTGGTAAACAGGTACTTGCTCCTTTCTCGTTCTCCATTTGTCAAAGAGCACGTACTCAAAAAATGGAGAAACTATTTGACCATTTAGATGCAGTTGTGGCAAAAATGTATGAATTTGATCTTAAAGAAAGGCACACTGGTGCCATAAAAATGGAGACAACAGACTCTTTCCTTACTGCCACTGAAGTTTCAACAAGTCTCATGAAACCAAGCTGGAAAGTACTTTACCCCTCGACTAGTGCTCCGAAGTCTTATCAGCACGAGCGGTATCGAGAGATTCTGAATGATTTCAAAGAATCTACTTTAGGGTTCTTCCACATAGTTGGAGAAGCAGGTATAGGTAAGAGCACACTTGCCAAATTCATTTACAATGATCCAGAAGTAGAGGAAATGTTTACATCAAGATTGTGGGTTTGTGTGAAAGAGGAATTTGATACGCAGAGATTGATGACAGAGATACTCAACTTTTCATATTCTCCAGCAACTTGTGACAATTTGACTGAGACAAAATCGTGCCCCACAGTTCAAGTTCAACAATATCTGAGAGAGAAAACTTTTCTGCTTGTTTTTCAAGACCTTTCAATCAAGAACCTAGATAATTCTTCCCTGTTTACAAGTTTATTGAGGATGGGAAAGCCTGGTAGCAAAATCATAGTGACCACTCAGAATGAGGAAATTGCAAATGCTATAAAACTACCAAAGGTCATGGTTGAGGTCAACAAGGTTGAGCAACAATCAGAGCAAAATGAGAGTCAGACAGCCCTAGACGCAGTTACTATAGAGACTTCAAATGTTAACAATGCTGGCGAGTCCACTCAAGCTAACCCTCTGGATAAGCTAGATCAATCTATCACATATCAAACAATATTCAAAGTTAAAAGGCTGTCAGAAGAAGATTCCCTTTCTTTATTCAAAGATTATGCTTCTATAGTTGAAGATGACAAAGAAGGTATAATAGAAACTCTGGAGAAATGTAATGGAGTACCATTGGCAATAAAGTGTCTGGGGAGCATGTTATCTTTGGAAACTTCAGCAACTAAATGGATGGAGGACACTGACCAACAAGAGGAAGTTAATGAGTCTTCCAGTACATTTAGTATACTTAAACTATGCTACAATCAGATGCCCTCACACCTGAAGCCTTGCTTTCTTTATTGTTCTCAATTACAAACCGATAGCATACTCTCTTCAAATGATGTCATTCAGTTATGGATGGCAAACGGACTTCTCCATTCACGCCAAGAGAATTACTTATCCTTGGAAGACATAGGTGAGATTTATTTCAAAGAACTATGCTCAAGATGTTTCCTTCAAGATGTTGAAGAATATGGTCTTGGCTATTGGTTTAAAATGCACCCTCTCATTCGAAAACTTGCACGAATACTCATACAAGAACAAACTAAGGACTTGATATGCATTAAACCAGTCACTAAGGTCACATCTATAGCCTTCCCAGTAAGAGATGAGGTACCATCTAGTTCATTTCTAGCTGAAAAATGCATATCAAAGTTCCAATACTTAAGATTATTGCATTTAGGCTACACAGATCTACAGGAAATTCCAAATACTATAGAAACACTGAAGCACCTAAGATACCTAGACTTGCAGGGAAATAAGAAAATCAAGCGGCTACCAAATGCAATCTGTAATCTACAATATTTGCAGACCTTGATTCTTGCATCTTGTTTTGCACTTCAAGAATTGCCAAAAGATATATGGAAATTGAGCAAGCTCAGATACCTGTGGGTAACATCAAACAATCTTCATTTGCACAAAAATGGAGTAGGAACCATGAATTCTCTAAGATTCCTCGCAATTGGAGGATGTGACAAACTTCAAGATCTATTCGAACAGCCATCATGCCTTGTACGCCTTGAAACCCTAATGATTTACAATTGTAAATCTTTGCAGTTGTTGCCAAATGAGATGGGGTCTCTAATCTCATTACAAAATTTGGTGATATGGAGTTGCGAACAACTTACACTGAAGGGTTTAGAGAAAGTCGACTTCAGCCTCCAAAGATTCACAATCAGAGAGCTTCCAAAAGTTAATAAATTGCCTGAATGGCTTCAAAGGTCAACCGAAACTCTAAGAGTCCTGGAAATCATTGATTGTCCCATCAGAGTGGAGGAAGAGGGAATCAAAATGTACAAAGCAGTTGAAAGTAAGATAATTCAAGGAGCTGTAGACGTCACTAGGAATTTGGTATGGCGAAGTCCAATGGTAGCGAAGAACGTACAGAAGATAGGTAACTACTATTAAGTCCTCCATTCAAATGCCTGAGCGATTGAGAAGAAAGAGCGGAATTGTGAACTTCTATAGAGCGGAATTGTGAACTTCTA

N3 (1-exon):

Same gene structure in other plant families:

RCOM_1292200 Disease resistance protein RGA2, putative [ *Ricinus communis* (castor bean) ]

Gene ID: 8278984, updated on 11-Aug-2012

>MELO3C015354

ATGGCAGAATCAATTCTGTTCACCCTTGCAGCAAATATTGCAACCAAGTTGGGTTCTTTCCCACTCCACGAGCTTGGATTGTTGTGGACCGGTTTCCATGAGGAGCTTGATAAACTCAAAGACACTCTTTCCGCCATCCAAGCAGTACTTCTCGACGCAGAAGAGAAGCAGTACAAGAGTTATGCTGTGAAGGAATGGGTTTCAAGGCTCAAAGATGCTTTCTACGATATCGATGATTTGATGGATGAGTTCTCCTATGAATCCATCAAAAGACAGGTTATGATCAAACATAGAACTAACAACAAACAAGTACGTATTTTCTTCTCAGAATCTAATCAAATTGCATTCCGTTTGAAGATGGGTTATAAAATCAAAAGGGTCAGGGAGAAACTCGATATTGTTGCTATTGATAAAGCTCAATTCAATCTTTCTGAGTATACAAGGGAGATACGAAACGACGAAACTACGAAACGACCGGAGACTTCCTCTTTTATACTTGAAGGAGAAGTAATTGGTCGAGATGATGACAAGAAAGGTATTGTACATTTTCTATTGGATACCAACGTCGCACAGGAAAATGTTGCTGTGGTTGCCATTATTGGAATGGGAGGATTAGGAAAGACCGCCCTTGCTCAATCTATCTACGGCGATATGAAGGAAAATAAACATTTTGAATTGACAATGTGGGTGTGTATTTCTGAAGAATTTGATGTCAAAATAATTGTTGAAAAGATTATAGAGTCTCTCACGAAAAAGAGACCTGAGCCCAACCTTCAACTCGATACGTTGCAAAATATGCTTCGAGAGAAAATTGATGGAAAAAGGTACTTGCTTGTCATGGATGATGTGTGGAATGTTAACCGAGAGAAATGGATTAATCTAAAAGCGTTTCTTACTGGTGGAGCTAAAGGAAGTAGGATTTTGATCACAACTCGTACTCATCAAGTTGCACATACTTCTGAAACAGTTTTGTTCCATCATTTAAGTGAACTAAACAAGAACAGCTCTTGGGAGTTGTTTAGGAAAATGGCATTTTCTAACGAATCAGAGGTGCTTGAAAATTCAAAGTTAGTTATAATCGGAAAGGAGATTGTGGCAAATTTGAAAGGTTCTCCTCTTGCGTTAAGGGTAATTGGGAGCTATCTATATTCTAAAAAGACTGAAAAGGATTGGTTGTCATTCAAGGACAATGAACTTGGCACAATCATGCAGCAGGAAAATGAGATTCAATCCATACTAAAGATCAGTTTTAACCAACTCTCATCCGGTTTGAAGCAATGTTTCACCTATTGTGCTTTGTTCCCTAAAGACTATGAGATTCAAAAAGATGATTTGATAAAACAATGGATGGCACAAGGCTTCATTCAACCACAGAATAAGAAGACAATGGAAGATGTCGGTGATGATTATTTCAAAGAATTATTGGGGAGATCATTTTTTCAAGACATAAAGAAAAACAAATGGGGAGAGATCAAGGAGTTCAAGATCCACGACTTCATGCATGATCTTGCATGTTCTGTTGTTGAAAATGTATGTGTGCTTACTAATGATGACACCAAGACCATTGACAAAAGGACTCGACATGTGTCAATTTCGACCTTCATCTCAAAGACAAGATGGAAAGTCATTACAGAATCATTAAAAGAGGCAAAGAATTTGAGAACATTGAATTATGCTTGCGATAAAATCGACCTCTCTAACCATTTGCAGTTACGAACATTGAATGTGGATTTTCTTTATCATGTTCCCAAGTGTATTGGTAAGATGAAACATTTGAGATATGTTAATCTTTCTAATACTTGGATTGATTTCCTTCCGAAGTTTATTACAAAATTATATAATTTAGAAACACTCATCCTTCGTAACTGTCAAAGACTAATGGGACTGCCAAGTGATATTAAGAATTTGATCAATCTTAGGCATCTTGATATTATGCATTGTAACTCAAATTGTTGGAGTTCTATGCCAATGGGATTGGGTTCGATGACTAGCCTTCAGACAATGAATTTGTTTGTATTGGGAGCGAAATGGGGTGGTGAATTAAGCGAACTGAATGGACTTAATAGCTTGAGAGGATCATTAAGAATTAAACAATTGCAATTTTGCATAACTCCTAATTTAGAAAATGCTAAATACCTTGAAGAAAAGTCTGGAATTCAGAAGTTGGAATTACATTGGGATTCTTCTTATGGAATAGGGCGTAATACTTTTAATGACGAAGATGAAAAAGTTTTAGAATGCTTGAAACCACATCCAAATCTTCAAAAAATACGCATAGAAGGATACAAAGGAGTGAAGTTATGTAATTGGTTCTCATTTGGTTCTATAGGTAGTTTGGTCACCATAAAGCTATGCGAGTGTGAAAAATTGAAACATCTCCCTCAATTTGATCAGTTTCCTTTTCTCAAGCACCTTCATCTCGAAGATTTACCGAATATTGAGTTTATTGATGATAACAATTATGTTTCTTCTTCGTTAACAACTTTCTTTCCCTCCCTTGAGAAACTAAGCATCATTCAGTTGCCTAAGTTGAAAGAATGGTGGAAAGGGGAATTCATTGATCAAACTACCCCATTTCCAACGACTTTGCATCACCTTTCTCGATTGAAGATTAATCATTGTCCACAGTTGGGTTCTATTCCACAACATGGACCTTTGAGGTCATTGGACGTAAGTGGTGTTAGTTTGCAACTTTTTGAGTTGGTCATGGAAATGGCTACTACGAACATTATTGTTGGACAAGATTCTTCTTCTTCAACTACTAGATCATTATCTTCTCTAAGTATTTGGGACATGGATTTTGAGTTTTTACAATTACATGACTTATTCTCCAATATGACACATCTTAAGTCTCTTGTCATAGGAAATTGCAAGAATATAAAAATGTCTTCTTCTCGTGATGGTGTGATATGGAAAGAACTTGGAAGCCTTCGTAGACTTGATTTGTGTAGCATCCCTGAATTGGAGTGTTTGCCAAAGGGTTTACAATATGTGACAACTCTCGAATGTTTGAAACTATATGATTGCCGAAATTTGGTATCTATTGAAGGGATTGAGCATCTCACTTCATTATCACTGTTGGAAATTGAATATTGTCCTAATTTAATTTCCTACCCTCAAGAAATGGGTCAACTCACTTCACTATCACGTTTGAGAATCAATGGTTGTCCCAATTTACCTTCCTTGCCAGAAGGGCTTCGCCATGTGACTTTCATGTATTATGAGGACCTACCGACCCCAAGGTATGCACTAAATTTATTTTCTATTAATAATTATAATTTTTGCATCGATCCCAAGGTATTGGAATGTCTTGAATTCTATTTATGTTATTTACAACTTCGGCATAGAGTTTAA

N4 (1-exon):

Same gene structure in other plant families:

POPTRDRAFT_595138 cc-nbs-lrr resistance protein [ *Populus trichocarpa* ]

Gene ID: 7470323, updated on 25-Aug-2012

>MELO3C006780

ATGGCTGATTTTGTCTGGACATTTGCACTGCAAGAGATTCTCAAGAAGACATTGCACCTTGCAACCCAGCAAATCCGTCTGGCCTGGGGTTTCAAGCAGGACCTCTCTAAACTTCTCGACTCACTACTCTTCTTCGAAGCCATTCTTCGCGATGTCGATCGAACAAAATCTGACCGAGAATCGATCAATATTTGGGTGACTAAGCTTCAGGATTTAGTGCTCGATGCCGAAGTTGTACTGGACGAGCTCTCCTACGAGAACCTTAGGCGAGAAATGGACGTCAATGGAAATTCTAAGGAAAGGGTACGTGATTTCTTTTCGTTATCGAATCCCTTGATGTTTAGGTTGAAAATGGCGCGTAAAATTAGAACCATCACCCAAGTTTTGAATGAGATTAAAGGCGAGGCTAGTGCTGTTGGGGTTATTCATAAAGGGGGGAATAATGAAATAGTGGCTGATAATGGCCAAATTCCAGAGACTGACTCATTTCTTGATGAATTCGAAGTTGTAGGAAGAAGGGCTGATATATCCAGAATAGTGAACATTGTTGTTGATAATGCCACTCATGAAAGGATTACTGTGATTCCTATTGTGGGAATGGGTGGTCTTGGAAAGACCACTTTGGCAAAAGCAGTCTTCAACCATGAGCCTGTGAAAGCGCATTTTGATGAAACTATCTGGGTGTGTGTGACTGCAACTTTTGATGAAAAGAAGATTTTAAGAGCAATTTTGGAATCTCTAACAAATTTTCCAAGTGGTTTGGATAGTCAGGATGCTATACTTAGAAGGCTACAAAAGGAGCTGGAAGGGAAAAGGTACTTTCTTGTGCTGGATGATGTGTGGAATGAAAATGTTAAACTGTGGAACAATTTCAAGAGTCTTCTGCTAAAGATTACAAATAGTATTGGGAATAGAGTTCTTGTGACAACTAGAAGTGAGGAAGCTGGAAAAATCATGGAAACATTTCCCAGTTATCATTTAGAAAAGCTATCAGATGATGAATGCTGGTCAATATTCAAGGAAAGAGCATCAGCAAATGGATTACCACTGACCCCAGAATTGGAAGTTATTAAGAATGTGCTTGCAGAGCAGTTTGGAGGCATTCCATTGGTTGCGAAAGTTCTGGGAGGGGCTGTACAATTTAAGAAAAGAACAGAGACTTGGTTGATGTCAACATTGGAAACCCTTATAATGAATCCACTTCAAAATGAAAATGACGTTTCATCTATTTTGAGATTAAGCGTGGATCATCTGCCAAACTCATCATTGAAACAATGCTTTGCCTACTTTTCTAATTTTCCCAAGGGTTTTAACTTTGAAAAGGAACAACTAATCCAATTTTGGATGGCAGAAGGGTTCATTCAACCTTCTGATAAAGTAAGCCCTGAAACCATGGAAGATATAGGAGATAAATACTTCAATATCTTGCTGGCCCGTTCCTTATTTCAAGATATTGTCAAAGATGAGAATGGTAAAATTACACACTGTAAGATGCATCATCTTCTACATGATCTTGCTTATTCTGTTTCAAAACGTGAAGCATTGGGTTCTAATCTTAATGGTCTAGTTGATGATGTTCCTCAAATTCGACAATTATCCCTGGTTGGCTGCGAGCAAAATGTAACGTTGCCTCCTAGAAGGAGCATGGAGAAGTTGCGTTCTCTATTTTTGGATAGAGATGTGTTTGGCCACAAGATTTTAGGTTTCAAGCGCTTGCGTGTTCTGAACATGTCCCAATGTGAAATACATAACTTACCAACTTCAATCGGAAGGTTAAAGCATCTAAGGTATATTGATGTCTCAAATAATATGATAAAGAAACTTCCAAAATCTATTGTTAAGCTTTATAAATTGCAGACCCTGAGGCTGGGTTGTTTTCGGGGAGAAGCCCCCAAAAAATTCATAAAATTGATCAGCTTGAGACATTTCTATATGAATGTTAAAAGACCAACAACTAGGCACATGCCTTCGTATTTAGGCAGGTTGGTTGATCTTCAATCCTTGCCTTTTTTTGTTGTTGGGACAAAGAAGGGTTTCCATATAGAAGAGCTTGGATACTTGAGGAATCTCAGAGGTAAATTAAAGCTTTACAATCTTGAATTAGTAAGAAATAAGGAGGAAGCCATGAGGGCAGATTTGGTGAAAAAGGATAGGGTGTACAAATTGAAACTGGTATGGAGTGAAAAAAGAGAAAATAATAATAACCATGACATTTCTGTTTTAGAAGGACTTCAACCACACAACAATCTTCAATACTTGACAGTTAAAGACTTTATGGGAGAACTTTTTCCAAATCTTACTTTTGTTGAAAATTTGGTACAAATTTCTCTAAAAAATTGTAGCAGATGTCGAAGAATTCCAACATTTGGACATTTACCTAATCTTAAGGTTCTTGAGATTTCTGGATTGCACAACCTAAAATGTATAGGAACGGAATTTTATGGAAACGAATATGAAGAAGGAAGTTTGTTTCCAAAATTGAAAAGATTTCATCTTTTGGACATGAAGAATCTTGGACGTTGGGAAGAAGCAGCAGTGCCAACAGAAGTTGCAGTTTTTCCTTGTCTTGAAGAGTTGAAAATTTTCGACTGTCCTAGACTAGAAATTGCACCTGATTACTTCTCGGCTCTTAGGACATTAGAAATTGATGATGTCAACAACCCAATTTCACAGATCACTCTTCAGACATTTAAACTACTTGGTATTATACACTCTGGCAACCTGAGTGGTTTGCCTGAGGAGTTACGTGGTAATCTGTCATCTCTTGAGGAGTTTAAGGTTTGGTATTATCTTCACTTGAAAACTTTTCCAACTATTGAGTGGCTCACTGATATTTTGAAATGCAAGATCGGATATGACACAAAGTGGACAAATATTCAATCTCATGGGCTAGAATCGTACACTTCTGTGAATGAATTGTCCATTGTTGGGCACTCTGATCTCACATCAACCCCAGATATAAAAGCTTTATGTAATCTTTCGTCTTTAACAATTAGTGGCTTGAAGAAATTGCCAAAAGGATTTCACTGCCTCACTTGCTTGAAAAGTTTGTCAATTGGTGGATTCATGGAGGGGTTTGATTTTAGGGCTCTTTTGCATCTCAAGTCTCTTGAAAATCTTGCAATGATAGATTTTGGTAGTGCAGAAAGCACTCTTCCTGATGAGCTTCAACACCTAACTGGCTTAAAGCACTTGAAAATTGTTGGATTTCAGGGCATTGAATCTCTGCCAGAGTGGTTAGGAAATCTTAACTCATTGGTAAGTTTGCATATAGAGAGTTGCAGAAAATTGAGAGAGCTTCCAGAAGCCATGGGTTGCCTCGCCAAATTGGAGGAACTGCGGAGTTTTAATTGCCAAGAGTTGAGGGTTTACCAAGACGAATCAGAATGGGCCAAAATTTCTTACATTCCAAGATTCATATCATTCAATTATTGGGTTGATGAGTAA

N5 (5-exon):

Same gene structure in other plant families:

LOC100853370 probable disease resistance protein At5g66900-like [ *Vitis vinifera* (wine grape) ]

Gene ID: 100853370, updated on 1-Feb-2013

>Csa010121

ATGGCGCTGGAATTGGTGGGTGGGGCTGTTTTGGGGGCTGTCGTTGGGGAGCTATTCAAAGCGATCTTGAATCTGGGTGAAAGGGCCATCAGTTTCAATCCTGTTCTTAAGGATATCCGTTCCAAGCTTAATGCTATAATGCCTTTGGTGAAGCAAATCGATGAGCTTAATGATTATCTCGATTACCCAAAAGAAGAAACAGAGAAATTGAGGGGTCTGATGGATGAAGGGAAGCAGTTGCTTCTCCAGTGCGGCGATGTGAAATTGGGGGATCTTAATTATTTGAAGAGACCATCTTACACCCAAAAGCTTCGGGAATTGGATACTGCACTTCGAAGCTTCATGGATGTTTTGATGTTGCAGATGGCTAGAGATCAGAAGAAGAACATGAAGATGATGAACCAAATGATGGAGATCATTTGTAGACTTGATAATAGAGGTGGGTCGAGTAAACCTATGGATTTGTTTGTTCCACCATGTCTGGTTCCTCAACTGCGAGAAGAAACCGTTGGGTTGGAGAAGCCAGTTAAGGAGTTGAAGGTGAAACTTCTCAAAAATGGGGTTCAAATGTTGGTGGTGACAGCTCCTGGTGGCTGCGGAAAAACCACACTGGCCTTAAAATTTTGCCACGACAAAGAAGTCAAAG

GTACGTCAAAATCATTCCTTTTGAGGGAAAAAATGAAAAAAGTTTGTCTACATATTGAGCTTAGGAAATGAAATTATATTAAACTATTGGGTGAAAAAAACTTAATGGCGTCTCTTACAGGAGCTTTCTAATTTCACGTCTTGATAG

ATATATTCCAGGAGAAGATCTTTGTCCCAGTTTCAAGAAAACCAGATTTGAAGCTTATATTGAAAGATATAATTGAAAGCCTTAGAGGAATTCAATTGCCTGATTTGCAAAGTGATGAACGTGCATTCTGCTATTTAGAATTGTGGTTGAAGCAGACAAGTGTAAATCGTCCTGTTTTGATTGTGTTAGATGATGTGTGGAGTGGGCAAGAATCTGAAGTTCTTCTTGATAAGCTGTTTCAATTGCCTTGCTGCAAGATCTTGGTCACTTCTAGGTTTTATTTCCCAAGATTTAGTGAGTCTTATTATTTGGAACCTTTGAACCATGAGAATGCAGTACAACTTTTTCGTCGTGCAGCATCACTGGACAAAGGAATTTCTAAGCTCCCCGATGATGAAACT

GTAGAAAAGGCAAACTTTCTATTATCTGATTAGCTCTACTTTTTCTAATATTTTTATATCTGTCAGTTTCTTTTAAATGGCTATGTTTAAAAAGCTCTTTGAAATTGAATTTCAG

ATAATTGGGGGATGCAAGAGACTACCTCTTGCACTGAAGGTAATCGGGAGGTCTCTTTCCCACAAACCGACATCTGTTTGGAAAGTAACGGGGAGGAATTTGGCTAGAAGTGGCTCCATATTTGATTCTGACAATGAACTTCTTGAATGCCTTCAGAGCAGTTTGGATGTCTTGGATGATAACATGGTAACTAAGAAGAGTTTCATGGATTTAGGCTCTTTTCATGAAGATCAAAGAATTTCTGCTTCTACCTTCATTGACATGTGCACAGTTTTGTACACACTAGACGAAAGTGAAGCAATGGTTACCCTTGACGAACTATCCTCTCGAAGTCTAGTTAATTTTGTCACAGCGAG

GTAAATACTATGCTCGAATGTTTGTAATTTCAGTTGCTGATAGTGTTGCCTGTATTATTGATGTAAAGAGAATAG

AAATTGATTTGTTTTGTGGGGTTGTTGATCAGAAAATATGGATATGATGATGACTTTTATGAAGAGTACTCTTTTACTCAGCATGATATTCTCAGAGATTTGGCTATTCACTTGATGAATATGGAGCCCATAGAACAAAGGAAAAGATTGATCTTAGACATTAATGGAAATGATCTTCCCAAATGGTGGGTTGATCAAGAAAAGCATACTTCCTATGCTCGCCTTATATCCATAACCACAG

GTTTGTCTCTTTCTCTGTGATATTCCATTTTCTTTCCTTTTTCTTTATCACTTTCACAACTTACTCAAAGGGGTTTTGGATGTTGCAG

ATAAGAGATTCTCAGCAAGTTGGCCTGACATGGAAGCACCTGAAGTGGAGGTTCTGATTCTTAATCTTCAGTCAAGAACTTACAACTTGCCTGGGTTCATCAAAAGAATGAATAAGCTGAAAGTTTTGATAATCACATATTTTGGTTCTTTTCTAACTGAGGTGACAAGTGAAGATAATCAACTACTCGACAGCCTAACAAGTCTTGAACGAATCAGGTTTGAGCGGATTTCAGTTCCTATCTTTAGTAATCCAAACCCGAAACCACTGATAAATCTGCAGAAAATATCCTTCTTTATGTGCAAATTTGGTCAAACATTCATGGATCCTTCAACCCCAATCTCAGATTTGTTGCCAAACCTGCTGGAGATTTCCATAGACTTCTGCAACAATTTGAGTGAAGTCCCCAATAGGTTGTGTGAAATTGTCAGCTTGCAGAAGCTGAGCATTACAAATTGCCATGGACTATCTTCCTTGCCAGAAGATGTAGGGAAGTTGATTAATCTAAAAAATCTAAGGCTAAGATCTTGCATTCATTTAGAAGAGTTTCCAGAGTCGACAACGAAGCTTCGGGAATTAGTCCTGCTTGATATATCTAACTGTATTGGTCTTGCCAAGCTTCCCGAGAAGATTGGTGAATTTCATAATTTAGAAAAGCTTGACATGAGACACTGCTGGAGTTTGAGCAAGCTGCCACTGTCGATTGGAAAGCTGAAAAATGTGAAGTTTTTATGTGATAGAGAGGTTGGAGAGTGGTTGAGAAAGGTTGCACCTCGCCTTGCCAAACAGGTGAAAGTGCAAGAGGAAGAAGCCAACCTGGAGTGGCTTGGTTTTTGA

N6 (5-exon):

Same gene structure in other plant families:

Phytozome Locus name : MDP0000190871 [*Malus domestica* ]

>Csa009413

ATGGCGGTTACAGATTTCTTTGTTGGAGAGATAGCCACTGAGCTTCTCAGAATGATGGTACAACTTTCGACCAAATCCTGCCTTTGTAAAACGACGGCAGCTCAAATCGCCAATTCTATTCAACAAATTCTGCCGATTATCGAAGAGATCAAGTACTCGGGAGTTGAATTACCCGCTCATCGCCAATTTCAGTTAGATCGCTTCAGCGAAACTCTTAGAAGAGGCATCGAGATTTCCGAGAAGGCTCTTCAATGTGGCCGATTAAACATTTACAGAAACTTACGGCTCGCGAGGAAGATGGAGAAGCTTGAAAAGGATATATGTCGATTCATTAATGGCACCATGCAGGCGCATATACTGGCCGACGTGCATCATATGAGATTCCAGACCACCGAGCGGTTTGACCGGCTTGAAGGTGTTTTGTTGGAGCGGCGGCTTGAGTCGATGAAGATTAGAGCAGATGCTTCGGGAGAGGAAAGGTGGTGGGTTGAGGAGGCGTTTAAGAAGGCCGAGGAGGAGGAAAGGTATGAGAGTAATTTCGTGAATATAGGAACTGGATTGCGTGTGGGGAAGAGAAAATTGAAGGAGCTGGTGATTGGAAAGGAGGATTTAACGGCGGTTGGGATTAGTGGAATTGGGGGTTCGGGGAAGACTACTTTAGCTAGAGAATTCTGCAAAGATCCGGAAGTTCGAA

GTGAGTTGTTTTTTTTTTTTTTTTCTTTTTTTTTTTTCTTGTTTTATGCGATGATTTCGATTTTCGGTTCAAAACTTAGTAGTTTTTCTACTGAATCTATATGGGTTTCTGACTTTGAAGCAAAGTAGATTAGAAAATTTTCACTTGAATTGGAAATTTCTTGTTAGTAAAGTGAATAGTTGTTAGAATATCTATTTGATACCGTAGCTCTTAAAAAGGTAAATAGGACATCCTTTTTTGTGTTTCTTCTTGTAGGAATTGTCGGATTCTTATTGTTCTTGCATGTTGATTGCCACCTTTTCAG

GACACTTTAAAGAGAGAATTTTGTTCTTAACGGTGTCACAGTCCCCTGATGTGGAGCAGCTGAGGAGAACGATCTGGGAATTTGTGATGGGTAGTGATAGTGTCAATTCTAATAATTTGATTTTACATGGGAGGCCTTCAAATTCAGCGCTTTTGGTTCTGGATGATGTGTGGTCAATTTCAGTTCTTGAAAATGTTATTCCAAACGTAACTGGTTGCAAAACTCTTGTTGTTTCACGATTCAAATTCCCTGAAGTTCTTAGAGAAACTTATGAAGTAGAGTTGTTGAAAGAAAGTGAAGCAATTGCTCTGTTTTGCCACTCAGCTTTCGGACAACAGTCGATTCCTTTGTCTGCTAATCACAACTTGGTCAAACAG

GTAAAGAACACAACATTCTTTCTGTTGGATTAGACACAAGGCAGTTATCTTTAAAACGTCTAATTTAGTGGTTTTATTTCACTCATTTGTTTGCAAATTTTCTTTGAAG

GTTGTGAATGAATGCAAATGTTTGCCTCTGGCTCTTAAAGTCATAGGAGCATCACTCAGAGGACAGAGCGAGATGTTCTGGAATAATGCCAAGTCTAGGTTGTCACGTGGCGAGCCTATTTGCGAGTCCCATGAGAACAAATTGCTTCAAAGAATGGCAATCAGTATTGAACGCCTCTCGAGTAAAGTGAGAGAATGTTTCCTCGACCTGGGATGCTTTCCTGAAGACAAAAGAATTCCTCTTGACATTCTCATCAATGTTTGGAAGGAGTTACATGATCTTGATGACGAAGAAGCTCTTGCTGTTCTTTTCGAGTTATCTCAGAAGAATCTTCTTACGTTGGTGAAAGATGCACG

GTATGGACACAACTATGTTACATCACTTATTTACGTAATCATTACAGACATTGCAATATGTTGGCTAAATACTTGATTGGTTGATATGCAG

CGGTGGTGACATTTATAGCAGTTATTATGAGATGTATGTCACTCAACACGATGTATTAAGGGACCTTGCCCTTCATTTCAGTTGCCAGGAGAATGTGAACGACCGCAAGCGATTACTGATGCCAAAAAGCGACACAGAGCTTCCAAAAGAATGGTTAAGGAAATCGGAACAGCCATTTAATGCCCAACTTGTTTCAATTCACACAG

GTAACTGATTACCCTTTATCAAAGGAAGATAAGACTTGCAAAAATAAGCATCCTGTAAAGCTTTTTAGGCAAAGAAATCCCTTGTCTGAGTTACCAAGACATGGCATTTCTATGATTATAAGCTTTTTATACCATGTCCTTAGAAGTATCGTCTCATCTTCATTACACATTTATGTTATAG

GTGAAATGGAAGAAATGGATTGGGCGCCTATGATATTTCCTGAAGCTAAAGTGCTCATTTTAAACTTCTCCTCGAGTGGATACTTCTTGCCTTCTTTTCTTTGCAACATGCCGAAGATAAGAGCATTAATTGTGCTAAATAACAATGCAACACATGCAACTCTCACCAATTTCTCAGTTTTTTCTAGTTTGGTCAACTTGAGAGGCATCTGGCTGGAAAAAATTTCCATGACACAACTATTCGATGCTTGCACGCCATTGAAACATCTAAGGAAGCTATCTCTTGTTTTCTGCAAGATCAACAACAGCCTCGACGAGTGGGCGGTAGATGTATCCCAGATCTTCCCGTTTCTTTTCGAACTCAAAATTGATCACTGCAACGACTTGCGTAAGCTACCTTCAAGCATTTGTGAGATGCAAAGTCTCAAGTGTCTTAGTGTCACCAACTGTCATAATCTCAGTCAACTCCCTACCAACTTATGGAAGCTGAAAAATCTACAAATCTTGAGACTTTTTGCTTGCCCACTCCTCAAAACTCTATCCCCAAGCATTTGTGTACTTTCTTGTCTAAAGTACATTGACATCTCCCAATGTGTTTACTTAACCAGCCTTCCTGAAGAAATTGGCAAGCTGACAAGCCTAGAGAAAATTGACATGAGAGAATGCTCACTCATAAGGAGACTACCTAGATCAGTTGTGTCTTTGCAATCTCTCTGTCACGTAATCTGCGAAGAAGACGTCTCGTGGCTATGGGAGGATTTGAAGAGTCATATGCCTAATTTGTACATTCAAGTCGCCGAGAAATGCTTCAACTTAGATTGGCTCAAAGAGTGA

N7 (4-exon):

Same gene structure in other plant families: Not find

> Csa012278

ATGGAGTTGTGTGCCGGTGCCATTGTTAATCCAATCGCAGAAAAAATCGCCAACTGCACGGTGGATCCGGTTTTCCGGCAACTAGATTATTTGCTCCACTTTAAAACCAATGTGAATGATCTCAAAGATCAAGGCAAGAAGCTGGTGGAAACCAGAGATTTTGTTCAACATTCTGTCGACTCCGCCAAAACCAATGGGTACGAGATCGAAGTTATGGTCACTGAATGGTTGGGGATAGCTGATCAATTTAGTGAAGATGTCGATAGGTTTTTCAACGAAGCCGACGGCCGAAGTCTTCGATGGTGGAATATGCTATCACGCCATCGATTTAGTAGAAGAGCTACCAAATTGGCTGTGGCAGTTGATAAAGCCATTCAAGGTGGGAGTTTCGAGAGAGTTGGGTTCCGTGTAACTCCACAAGAAATTATGACGCTAAGGAACAATAAGAAGTTCGAAGCCTTTGAATCTAGGGTTTTGATTCTGAAGGAGATAATTGAAGCGGTTGGCGATGCTAATGCGAGGGTGATTGTGGTACATGGGATGGCGGGAGTTGGGAAAACCACCCTAGTTGAAGAAATTGCAAGATTGGCCAAGGAGGGGAAGCTTTTTGATGCTATAGCAATGGTGACTGTAAAGCACATTCCAAACATTAAGAAAATACAGGGGGAGATTGCTGATCAATTGGGGTTGAAATTTGAAGAGGAAAAGGAACGAATTAGGGCCGATCGACTACGTCGAAGGTTAGAGATGGAGAAGAAGGTGTTAGTGGTTTTGGATGATGTTTGGAGTAGGCTTGATTTGGAAGCTGTTGGAATTTCTAGCCATCACAAGGGATGTAAGATACTTGTAACTTCTAGAAAGGATGATTTGTTTTTCAATGATTTTGGTACTCAGAAAAATATATATATCAATATTCTGTCAAAAAAAGAAGCTAGGGATTTTTTCAACAAGGTGGCATGTGATTCTGTTGAATCTTCTGATGATACTGATCCTGAAATGGAAGCTGTTGCTACTGAATTGGCAGATGAATGTGGAGGATTGCCACTTTCTCTTGCAACTGTTGGACAAGCCTTGAAAGGTAAAGGGCTTCCAAGTTGGAATGATGCCTTGCAAGGAATGAAGTTTCCTGGCGAACCCAGTAACTATGGGGTGAATAAAGTGGCATATTTGTCTCTGAAAGTGAGTTATAGATCTCTAAACAGAGAAGAAGCCAGATCACTATTCTTACTATGTAGCTTGTTTCCAGAAGATTATCAAATTAACATCAAATACTTGTTGATGTATGCCATGGGTTTGGGGTTATTAAACGCCATGAGTTCTCTAGCAATGGCAAAATGGAGAATACTTTCTTTGGTTGATGAGCTCAAAACTTCTCACTTGTTGCTTGATGGGGTTGATAACGATTTTGTGAAAATGCACGATATAGTTCGAGATACAGCAATTTTGATTGCGTCGAAAATGAAGTCCAAGTATTTGGTTAGACATGGTGCTGGAGAGAGTTTGTGGCCCCCAATGGATGAGTTCAAAGATTACACTGCAATCTCATTAGGTTGCAGTGATCACTCGGAACTCCCAGAATTTATATGTCCACAGCTTAGATTCTTATTACTGGTAGGAAAAAGAACATCTTTGCGATTACCTGAAAAGTTCTTTGCAGGTATGCAGGAACTACGAGTTTTAGATCTCACTGGCTTATGTATTCAGCGGCTTCCACCATCAATCGACCAACTGGTAAATCTTCAAACATTGTGTTTAGATGACTGTGTTTTGCCAGACATGTCTGTAGTTGGTGAACTGAAAAAGCTTGAAATTCTTAGCTTGAGAGCATCTGATATTATTGCACTTCCTAGAGTAATTGGGGAACTTACCAATTTGAAAATGTTGAATTTGTCTGATTGTTCTAAACTCAAGGTGATCCCTGCTAACCTTTTATCTAGGTTGATAGGGTTGTCTGAGCTATACATGGACAATAGTTTTAAACATTGGAATGTAGGACAGATGGAAGGTTATGTTAATGCAAGGATTTCTGAACTAGACAACCTGCCACGGTTGACCACTCTACATGTCCATATTCCAAATCCCACCATTCTACCACATGCCTTTGTCTTTAGAAAATTGAGTGGTTACAGAATACTAATTGGAGATAGATGGGATTGGTCTGGCAATTATGAAACTTCAAGGACCTTGAAACTCAAGCTTGATAGTAGCATTCAGAGAGAGGATGCAATTCAAGCACTTCTAGAGAATATTGAAGATCTGTATTTAGATGAATTAGAAAGTGTCAAGAATATTCTATTCAGTCTAGACTATAAAGGCTTTCCGAAATTGAAAGGTTTGCGTGTCAAAAACAATGGTGAAATTGTGACTGTTGTCAACTCGGATAACATGCATCATCCACACAGTGCCTTTCCATTGTTGGAGTCCTTATTTCTGAAAAATCTAGCTGAACTTGGAAGCATTTGTCGTGGAAAGCTTCCACAAATGTCCTTCCGTAACTTGAAAAGAGTAAAAGTTGAAAGTTGTGACAGATTAAAATTTGTTTTCCCATCTTCTATGGTCAGAGGCCTTATACATCTTCAAAGCCTGGAGATTAGTGAATGTGGCATCATAGAAACTATAGTTTCGAAAAACAAAGAAACAGAAATGCAAATCAATGGTGATAAGTGGGATGAGAACATGATTGAGTTTCCTGAATTGCGTTCTCTGATACTTCAACATCTACCAGCCCTTATGGGTTTCTATTGTCATGATTGCATAACTGTGCCTTCAACCAAAGTGGATTCACGTCAAACAGTTTTTACTATTGAACCTAGTTTTCATCCACTTCTCAGTCAACAG

GTATGTTATCTATAATTGTACTTCTTCCATTGTTATCTTTGAGCTAATATAGACTAAATATATGCTATAGTGTCTAGGTTTTTTTTAAAATTTAGTTCATGTAGTTTAAAAGCTCTAACTTTCATCTCTTATGTTTTGACATTGTCCAAATTATTACATTTTTGTCGGTTGTATTGATTGGTGATAATCTTACATATTTGGAGAGTTGGACAAAAATTTGAAGGAGGAACTTGGATTATTGATGAGTAAGGTTTTCTAAAAAGATTTTTTTTTTGCCAATTTCTTATAAGGTTAAGTATGAAGTAGAATTTATTTTAGTTGGCTTAATTCCTCCTTTATGTTTGTTCCAAATCACTTTCTATCGTGGCTAAATATATGTTTTGGTTGTCTTTTCAATTTGGTTTCTAATGTTTAAAAGTTTCAATTTACTCTTATTATGTTTTAACATTGTTAGAAAATATGAAATTTGCTTGTGATGAACAAACGTGATATGATACTAATTGAGTTGGTAGAAAGTTTGAGAATAAAATTTTTCGTGTAAGTGAATTTTTTTTTCAATTCAGCCTCTATATTTTAAGAAAGTTGCCAAAGCTATAAAAAACAAATTCTTTTAGGAAACGTAATTGTTCTTCCAAATTTCTCATGGTCATAAGCCAATTTGAAATTTTCTAACAGAAGGGTATAATCGAAACATATTAAAACAAAAAGGACTAAACTAAAAAATTTTAAATATTAGAAAACCGAAATATATATTTAGCCTAATTATAGTTGATAATTGTTGGAAACTAAAGACTCGTTTGGTAACAAGTTTCCTGTTTCCTGTTTTCATTTCTTAAGAAATGGAGGTGTTTGGTAACGTTTTATGTTTCTCGTTCTAAAAAAAGTAGAAATATTTATCATTTTATAAGGAATTATTGAAAACAAAAAAAAGTAGTTTCTTCTATTCTCGTTTTTTAATTATTTCTATTGGTTTCTTTTTCCTTTTTCTAAATTGTTTCTATTGTTTCCTTTTTCTAAATTGTTTCTATTGGTTTTCTTCTCTTTTTTCTCAAGAAGGAACAGGAAACCGGAAACAAGGAACAATGAAAAAAACAGGGAACGAAAATGTTACCAAACGAGCCCTAAGGAATTGAGTTGAAAGTTTTCAGAATTTAGAGACTAAATTGAAGAAACACCCAAACATATATTTGGCCTTTTTATCTTTCTATGGCCTTTAAATCTACAAATCCAACATTGTGAAATAATATAATCAGCCATGAACGATAATGGATTCTGCTTGCTTTCTGGTTGGAATGTTTCTCGTATTCATTCTCACAATTATGTATTGACTATTGATAAAAAAATTGTTAAAAACTGTTTTTTAATTATTATTTATTTTTTTATCTTCTTAATGTTTTGTAG

GTTTCCTTCCCCAAATTGGAGACATTAAAATTACACGCTTTGAACTCAGGAAAGATATGGCAGGATCAACTTCCTTCTAGCTTTTATGGCTTTAAAAATCTAACTTCTTTGAGTGTGGAGGGTTGTGCTTCAATAAAATATTTAATGACAATCACTGTGGCTAGAAGCCTTGTGAATCTTGAACGCCTTGAACTAAACGACTGTAAGTTGATGAAAGCTATAATCATTTCAGAAGATCAAGATCTGGACAACAATTACCCTTCCAAATCTATCTTGCAGAACAAG

GTACATTTTTAACCTCATCATACTTCTTCCGTCTATGTTTTGTTTATAAACTTTAAGCTTAATAATGTCTGTCTTAAAAATTTTCATTTATGTCCATTAACTTTAAGTTGTGGACAATCTATTTAGTGCCTATTGTTAACATAAGCAAGGTGATATGGTCATTACAACCTAATCTCCAAATATCTGATGTGATATGGTTCATTTTTGCTAGTATAAGCTTTCATGGTTTTGCTTTTGAAATCACCAAAAAGGCTTCATACCGATGGAAATAATTGTCTTCAATACTTATGATCATTTCCCTTTCCCTGTGTATGTTTAGAATGCAAATATAACCTTCTTATGGGATAAAAACTAATTGGTTACATAACAAACAAGTGATAAATACAAGTGATCGGATCTAAGATAGGTCAATATAGATGGGTTAAGTTAATACTTTTATGTCAGCTTTTGTTAAGATGTGCTAAATCATGTTATAATATGTTTATAATCATGCCATATCCAAAAAAAGAGGTATATATATTATTATAATTTTCTAAAAAGAGGTTCAGAGGCTTCCAATAGAATATTCAAAACTCAAGGACTTCAACTTGGTCATATATTTAAACTTACCATGTTAGTTTGATTTTTGAGCAAAAGTTATAATTAAACTGAAATGATGAATATAATGCAG

GATGTTTTTGCGAACCTGGAGTCCCTCTTAATCTCTCGCATGGATGCTTTGGAGACATTATGGGTCAATGAAGCTGCTTCAGGATCCTTTACAAAGCTGAAAAAAGTGGACATCAGAAACTGCAAAAAACTTGAGACAATCTTTCCAAATTACATGCTTAACAGAGTGACAAATCTCGAGAGATTAAACGTTACAGATTGCAGTTCCCTAGTGGAGATCTTTCAAGTGAAAGTCCCAGTTAACAATGGCAACCAAGTAAGAGACATTGGAGCTAACCATTTGAAAGAGTTGAAGCTGCTTCGTCTACCTAAACTAAAGCACATATGGAGCTCAGATCCACACAATTTTTTACGCTATCCATCTCTCCAACTTGTTCATACAATTCATTGTCAAAGCCTTTTGAATCTCTTCCCTGTATCCATAGCTAAGGATCTCATACAACTTGAAGTGCTTAAAATACAGTTCTGTGGAGTTGAGGAAATTGTTGCGAAACGAGGAGACGATGGAGATGGAGATGATGCTGCGTCGTTTTTGTTGAGTGGTTTGACATCATTGACTCTTTGGAATTTGTTCGAGTTCAAGAGGTTTTATCCTGGGAAATATACTTTGGATTGTCCATCATTGACAGCGCTAGATGTACGCCATTGCAAATCATTTAAGTTGATGGAAGGAACTTTGGAAAATTCGTCATCAATCTCATCCGCTGTTGAAAAG

GTATTACATTCTCTCTAAAAGAATATGCAGTTTAGGTTCCTTTTTATAATATAATTACTTTGTACTTTAATGTGCTTTTTATAATATGCCAAGTCTCATGCATAATTGATATCCTATGCTATTTTGGGCTTCATTTGATATCCATCTGATTTAAAAAAAAAACATTTATGTCCTCTTCATGTTAACCTATAACCATTCTGTTTTGTCTTGAAAATCAACCATACAAATCCTTTTTTCACCTCTACATTTCTTGTTTAGTTATATCAACCATACAAATCCTTTTTTTACCTCTAAATTTCTTGTTTAGTTATATACTTTGTACCAATGTTTTCAAGACCTAAATCTAGTTTTGAAAATTAAAAAAAAGATAATTTTAAACTTGTTTTAAAATAAAATTTGGCTAACTTTTCTTTTGATGAAATTTGACTAAGAATTGAACTCTAAAAAGTGAAAAAAAATCTAAAAATTGAAATTGGTATGAAATAAGCTTAATTAAAAAAGAAAAAAAAAAAACCTAAACTAAACCACATGCCTACAAAAGGGTGTCTTTTAAGGGTTTAATTTTTATTATACTAAGTCTTTGTAGATATAATTGTAATAATCACTGTTGTTGAAAGTTGTCTGTGCTCTTTAGAAAACATACATGTTTGTGACATTCTTACTGTTCATTATTAAAACAGTGAAACAAAGTTGATCGGTTTTGATTTATTATGGTTGTAATAATTAG

GTAGAAGTTGAGCAATCTTCACTGAGGGGGGAGTTCGAAAGAAGAGAATCAAAGGAGACATCCACAGGGAAAGAAGAAATAACAACCATAGTGCAAGGTGTAGTTGATGCTGAGTTAATTGAATTAAGAGCTCAATTACGAGCTCTTGTGGCAGGTCAAAACCAAATGATGGAACGCTTAGCCCAACTTACAACAATTCCTCGTGAGCCTGTCTCAAAGTGA

N8 (3-exon):

Same gene structure in other plant families:

Phytozome Locus name：ppa016994m [*Prunus persica*]

The sequence was re-annotated and added in this file.

>Csa007358

ATGGCCGAATTTATAATAAATGTTGCGTCAGTAATTGTAACACCAATAGGAAAGTATGTGATTAAACCAATTGGAAATCAACTTGGTTACATTGTTTTCTACAACAGAAACAAGAATGAGATTAAAGAGCAACTTGAAAGTCTTGAGACTACTAAAAAGGATTTGGATCTAAGGGTTGAAGATGCAAAAAGCAAGGCATATACCATCTTTACGAAAGTTTCAGAGTGGTTGGTCGCTGCGGATGACGAAATAAAGAAATCTGATGAGCTATTCAATTCCAACCCACCTTGCCTTAACTTTCTCCAACGACACCAACTAAGTAGAAAGGCAAGGAAGAGGGCGACGGATATCCGCCGACTCAAAGACGGAGGAAACAACTTTCTGGAAGTTGGTTGTCCTGCCCCTTTACCGGATACTATGAATACTATTGTTCCTGAAGCTTATCAAACTTTAGGATCAAAAACCTCAATGGCCAAGCAAATTAAGGACGCCCTTGCAAAACCTGAGGTAAGAAAGGTTGGAATCTATGGTATGGGAGGTGTTGGAAAAACATATTTGCTCAAGGAAGTTAAGAAATTGGTGTTGGAAGAAAAATTGTTTGATCTAGTGATTGATGTGACTGTAGGTCAATCTAATGATGTAATGAATATGCAACAACAAATTGGAGACTTCCTCAATAAAGAATTGCCAAAGAGTAAGGAGGGAAGAACATCCTTTCTACGAAATGCATTGGTGGAAATGAAAGGTAATATCCTGATCACATTCGATGATTTATGGAATGAATTTGATATCATAAACGATGTTGGAATTCCGTTAAGTAAAGAAGGATGTAAGACACTTGTCACAAGTCGTTTTCAAAATGTTCTAGCCAATAAAATGAATATAAAAGAGTGTTTTAAGGTGACTTGTCTAGACGATGAAGAGTCTTGGAAGTTTTTTAAGAAAATTATTGGTGATGAGTTTGATGCAAAAATGGAAAACATTGCAAAGGAAGTGGCCAAACAATGTGGAGGATTACCACTTGCACTTGATATCATTGCAAAAACATTAAAGAGATCAAGACATATAAATTATTATTGGGAGGGAGTGTTAAGTAAGCTGAAAAATTCAATTCCGGTGAATATTGACGTGGGTGAAAAAGTTTATGCTTCACTTAAACTAAGCTATGAACATTTGGATGGAGAAGAAGTCAAATCACTATTTCTTCTTTGTAGCGTATTTCCAGATGATCATGGGATTTCAGTAAACGATCTGCAAATGTATGTGATGGGTATGGGACTATTGAAAATGGTAAATACTTGGAAGGAAGCAAGAGCTGAAGCACATTACTTGGTCGAGGATCTTACATCATCTTCTTTACTTCAACGACTTAAGAATAGAGATGTTAAAATGCATGATATAGTTCGTGATGTTGCAATATACATTGGACCAGACTTTAACATGTCTACACTTTACTATGGATATAGTACAAGTAGCAAAGGGCTAGATGAGGATAAATGTAGATCTTATCGTGCAATCTTTGTAGACTGTAAGAAGTTTTGCAACCTTCTTCCAAACTTGAAGCTTCCAAAACTAGAATTGTTAATATTAAGTTTTCCTTTTTGGGGGAAAGATAGAAATATTGACATTATGGATGCATATTTTGAAGGAATGGAAAATCTTAAGGTTTTGGACATTGAAGGAACAAGTTTCCTTCAACCATTTTGGACACCGTTAAAGAACCTTCGAACGTTATGTATGTCATATTGTTGGTGTGAGGATATTGATACAATTGGGCACTTAAAGCAATTGGAAATTTTGAGGATTAGTAATTGTAGAGGCATCACAGAATTACCAACGTCTATGAGTGAATTGAAACAACTTAAGGTATTAGTTGTGTCGCATTGCTTCAAGTTGGTGGTGATTCACACAAACATTATTTCAAGCATGACCAAATTAGAAGAGTTGGATATACAAGACTGCTTTAAGGAATGGGGAGAAGAAGTAAGGTACAAGAACACATGGATTCCAAATGCACAACTTTCAGAATTGAATTGTCTGTCACATCTTTCTATTTTAAGAGTACGTGTTTTGAAGCTTACCATTCTCTCCGAGGCTTTGAGTTCACAAATGTTGAAAAACCTAAGAGAATTCTTTATTTATGTTGGTACCCATGAGCCTAAGTTTCATCCTTTTAAATCATGGTCGAGTTTTGATAAATATGAAAAAAATATGTCCTTTAATATGAAATCGCAGATTGTTTCAGTCAACCCGACGAAACTTAGCATATTATTAGAAGGAACTAAAAGGTTGATGATTCTAAATGACTCCAAAGGTTTTGCAAATGATATTTTCAAAGCAATTGGAAATGGTTATCCCCTGTTGAAGTGTCTTGAAATTCACGATAATTCAGAGACACCACATTTGAGAGGAAATGATTTCACATCTTTGAAGAGGTTGGTTCTTGATAGAATGGTGATGTTGGAGAGTATTATTCCGAGGCATTCTCCAATAAATCCTTTCAACAAACTTAAATTCATAAAAATAGGAAGGTGCGAGCAGCTAAGGAATTTTTTTCCACTCTCTGTTTTTAAAGGGCTTTCAAATCTTCGACAGATTGAGATCTATGAATGTAATATGATGGAGGAGATTGTATCAATAGAAATTGAAGATCATATCACTATTTACACTTCTCCTTTGACATCTTTACGCATCGAGCGTGTGAATAAACTTACAAGTTTTTGCAGTACCAAATCATCCATCCAACAAACAATT

GTTCCCTTATTTGATGAACGACGGGTATGTGGACCATAAAACTCAGCTCTTTTGCCTTCTATTTGTTGGTGTGATAAATAAATTATGTTTGTTTGTAGCTATTTATTGGGTAATTTACATTAATTTGAAGACTAGTACTATTTTCAAATAATTTGACGGAGGTTTTAATTTGAAAAGAAAAAACTGAGAAATTAGTTTGAAAACATGTTAATTTAG

ATTTTATGTTATGATAATTATATTGATATATATATTTTTTTTGTATCTGAGGAGATTATAGTAAAATTATTTCAGGTTTCATTTCCTGAATTGAAGTATTTATCAATTGGTAGAGCAAACAATTTGGAGATGCTATGGCATAAGAATGGAAGTTCCTTTTCCAAACTTCAAACAATAGAGATTAGTGATTGCAAGGAGTTGAGATGCGTGTTTCCTTCAAATATAGCGACGTCACTTGTCTTTTTAGATACATTGAAAATCTATGGTTGTGAGTTATTGGAAATGATATTTGAAATTGAAAAGCAGAAGACTTCGGGAGATACAAAAGTAGTGCCATTGAGATACTTATCTTTAGGATTTCTAAAAAATTTAAAGTACGTGTGGGACAAAGATGTTGACGATGTTGTGGCATTTCCAAACCTAAAGAAAGTTAAGGTTGGTAGATGCCCTAAGTTGAAAATTATTTTTCCAGCTTCCTTCACCAAATATATGAAAGAAATTGAAGAGTTAGAAATGGTTGAGCCGTTTAATTATGAAATATTTCCAGTGGATGAAGCATCAAAGTTAAAAGAGGTAATTCATACATCATGTCATCAATGTCTGTATGTAGGCAAGAATTGTATATGCTTCAATTTGATACTAATTAACTTCTATTAT

GTTAATCTACAAAGAATTTTGTTATTACATTTTTTTTTTTGGTGGAGCCAAATATATATATGTATGTGGATATTAAAAAAATAATGGTTTAGAGACTTTGTGTGTGTATTTCAGGTTGCATTGTTCCAAAGCTTGGAAACATTGAGAATGAGTTGTAAG

CAGGCTGTAAAAGAGAGGTTTTGGGTTATGTCAAAGTTCTTCAAACTCAAAAGTCTTGAATTGTTTGGTTGTGAAGATGGTAAAATGATTAGCTTGCCGATGGAAATGAATGAAGTATTATACAGCATTGAAGAATTGACAATTAGAGGATGCCTCCAGCTGGTAGATGTAATTGGAAATGACTATTATATCCAAAGATGTGCAAATTTGAAGAAGTTAAAATTGTATAATCTTCCGAAGCTTATGTACGTGTTGAAGAACATGAATCAAATGACTGCAACCACATTCTCCAAGTTGGTTTATCTTCAAGTAGGTGGTTGCAATGGAATGATAAATTTATTTAGTCCTTCAGTGGCAAAGAATCTAGCGAATCTCAATTCCATTGAAATATATGATTGTGGAGAAATGAGAACCGTAGTTGCAGCAAAAGCAGAGGAAGAAGAGGAAAATGTTGAAATTGTGTTCAGCAAGCTAACTGGTATGGAATTCCATAATTTAGCAGGATTGGAATGTTTTTACCCTGGAAAATGCACACTTGAATTCCCCTTATTAGATACGTTGAGGATAAGCAAATGCGATGACATGAAAATCTTTTCATACGGAATAACAAACACTCCCACTTTGAAAAACATCGAGATTGGAGAACATAACTCATTGCCAGTATTACCAACACAAGGGATAAATGACATTATCCATGCTTTTTTCACAATTGAGGTATGCATTATTTCATTTCAAAATACACTAAATAGTATTTCTCAATTTCTTGCTCAATTATATTATCAAAATCCACTTTGTGTGTGA

N9 (4-exon):

Same gene structure in other plant families: Not find

>Cucsa.091880

ATGGAAAGTATTCCTATTTCAATAATTGCAAAAATTTGTGAATACACTGTTAAACCTGTTGGACGTCAACTTTGTTATGTATGTTTCATTCATTCCAACTTTCAAAAACTCAAGAGTCAAGTAGAAAAGCTGACAGATACAAAAGGATCTGTGGAAGACAAGGTTTTTATTGCAAGAAGAAATGCAGAAGACATAAAACCTGCAGTTGAGAAATGGTTGGAAAAGGTTGATCGCCTTGTTAGAAAATCTGAGAAGATACTAGCCCATGAAGGTAGGCATGGTAGATTGTGTTCCACCAATTTGGTCCAAAGACACAAGGCAAGTAGAAAAGCAAGCAAAATGGCAGATGAGGTTCTTGAGATGAAAAATCAGGGAGAAAGTTTTGATATGGTATCCTTTAAAGGTCGTATCTCATTGGTTGAGAGTCCACTGCCAAAAGCACCTGACTTTCTTGACTTTGGCTCTAGAAAGTCAACAGTGGAACAAATCATGGATGCACTCTCTGATGATAATGTCCATAAGATTGGAGTGTACGGGATGGGGGGTGTTGGCAAAACAATGCTAGTGAAAGAAATTGTAAGAAAAATTGAGGAGAGTAAGAAGTCTTTTGATAAGGTGGTAACATCCACGATTAGCCAAACACCAGATTTTAAAAGGATTCAAGGACAACTAGCTGACAAGATAGGTTTAAAATTCGAACAAGAAACAATAGAAGGAAGGGCTACTTTTCTACGAAGGTGGTTGAAGGCGGAGAGAAGTATCCTAGTCGTGTTGGATGATGTCTGGGAGTATATTGATTTGGAAACAATAGGAATTCCAAGTGTTGAAGATCATAAAGGAATATGCAAGATCTTGTTTACCTCTAGGAATAAACAATTGATCTCAAATGATATGGGCGCCAATAAAATTTTTGAGATAAAAGTTTTAGGAGAAGATGAGTCCTGGAATTTATTTAAGGCAATGGCGGGTGAAATTGTTGAAGCAACTGATTTGAAGCCTATAGCCATTCAAATTATGAGAGAATGTGCAGGTTTGCCTATTGCTATTACTACTGTTGCTAAGGCATTACTAAATAAACCTTCCGACATTTGGAATGATGCCTTAGATCAACTTAAAAGTGTTGATGTGGGTATGGCAAACATTGGAGAAATGGACAAGAAAGTGTATTTGTCACTAAAACTGAGTTACGATTACTTGGGATATGAAGAGGTGAAGTTACTATTCTTGTTATGTAGCATGTTTCCAGAAGATTTTAACATTGATGTGGAAAAGTTGCACGTATATGCTATGAGCATGGGTTTCTTACGTGGTGTTGATACTGTGGTAAAAGGACGACGAAGGATTAAAAAATTGGTTGACGATCTTATATCTTCTTCCTTGCTTCAACAATATTCTGAGTATGGGAACAATTATGTGAAAATACATGATATGGTTCGTGATGTAGCCATACTAATAGCATCTCAGAATGATCACATACGTACATTGAGCTATGTGAAAAGATCGAATGAAGAATGGAAAGAAGAGAAATTGTCGGGTAACCATACTGTAGTGTTCTTAATTATTCAAGAATTGGATTCACCTGATTTCTCAAAGTTAATGCTACCTAAAGTTCAATTGTTCGTGTTATTTGGACCATCACCATCTATATATAATAGACATGTTGTGTCAGTGGTAGAAACTTTCTATAAAGAAATGAAGGAGCTCGAAGGTTTGGTAATAGAAAGGGTGAAAATATCCTTATCGCCACAAGCTCTTTACTCATTTGCAAACCTTAGATTATTAAGATTACATGACTGTGAATTAGGGAGCATAGATATGATTGGTGAATTAAAAAAGCTTGAAATTCTTGATTTTAGTAAATCTAACATCGTTGAAATTCCTATGACCTTTAGCAAATTGACGCAGTTAAAAGTGTTAAATTTATCTTTTTGTGATGAGCTTGAGGTAATTCCACCCAATATTCTTTCAAAATTGACAAAACTGGAAGAATTACATCTAGAAACTTTCGATAGTTGGGAAGGGGAAGAATGGTACGAAGGAAGGAAAAATGCTAGTCTTTCTGAGCTAAGGTACTTGCCACACCTTTATGCTTTAAATTTAACCATTCAAGATGATGAGATTATGCCAAAACACTTGTTTTTAGCTGGGGAGTTGAATCTTGAAAATTTTCACATTACTATTGGTTGTCAGAGACAAAAAAGACATATTGATAATAAGACCAATTTCTTTAGAATCAAGATGGAATCAGAAAGGTGCTTGGATGATTGGATAAAAACTTTGTTAAAGAGGTCAGAAGAAGTCCATTTGAAAGGATCAATTTGTTCAAAGGTTCTCCACGATGCAAATGAATTCTTACATTTGAAGTATTTATACATTTCTGATAATTTAGAATTTCAACATTTTATCCATGAAAAGAACAATCCTTTGCGAAAATGCTTACCCAAATTGGAGTACCTATATCTGGAAGAGTTAGAGAATTTGAAGAATATAATTCATGGGTATCATAGAGAATCTCTTTTTAGCAAGTTGAAAAGTGTAGTCGTAACGAAGTGCAATAAATTAGAAAAGCTCTTTTTCAACTGCATATTGGATGACATTCTGAGTCTTGAGGAGATTGCTATTCATTATTGTGAGAAGATGGAAGTGATGATTGTGATGGAAAACGAGGAGGCAACCAACCACATTGAGTTTACTCATTTAAAGTATTTATTTCTAACGTATGTACCACAACTTCAAAAATTTTGCTCCAAAATTGAGAAATTTGGACAATTAAGTCAGGATAACTCAATCAGCAATACCGTTGACATTGGTGAATCGTTTTTCAATGAAGAGGTAAATTCCATCAACACACTTTTCTAT

GTCTCTATATAATACATGTAAAAAATTAAATTACCTTTAATTTCTGTTCTTTTTATTGTTCCTCCCACACAAGTTCTTCCTTCTAGCTTTTGTGGTTCAGTAATGTATCAGAGATCAGATTCTATTCTTCTTTGTTTAAGAAGGTTCTTCACTCAACAAAGAGATGACTTTCTTGTTTCATCCATTATTAATAAAAAGATTTTTTCCTATCCAAGGAAGCTCGATCGTTTGAAATAACCATTTTACACAATTTGTTATGTTAATTTTTAAGGACTATAAACATTATTATATGTTGTTAGCATCAGTTGTGATGATGGTCAATTACGTTATCTTAAACTGAGTTTTCAAAGTATGCAAAATTGTAG

GTATCACTTCCTAATTTGGAGAAATTGGGAATTAAGTGTGCAGAGAATTTGACGATGATATGGTGCAATAATGTACACTTTCCTAATTCCTTTTCCAAACTGGAGGAAGTAGAGATTGCTTCATGCAACAATCTTCACAAAGTATTATTTCCTTCAAATGTGATGAGCATTCTTACATGCCTTAAAGTCTTAAGGATTAATTGTTGTAAGCTGTTGGAAGGTATATTTGAAGTGCAAGAGTCAAGTATTACAGATACAAGTCTTATTGTGCTCAAAAACTTGAGAGAGTTGAAATTATATAATCTTCCCAACCTTGAGTACGTATGGAGCAAAAATCCTTGTGAGCTTCTGAGTTTTGTAAATATAAAAGGTTTGGCCATTGATGAATGTCCAAGACTTAGAAGAGAATATTCAGTCAAAATTCTCAAGCAACTTGAAAGACTAACAATGGATATTAAACAATTGATGGAGGTTATTGAGAATCAAAAGTCAACCGATCATAATATGGTGAAATCAAAGCAATTGGAGACTTCTTCTAAG

GTAATAATAATTTCCAACTACACAACCCATCATGTTTATTTGATATTTCACAGAATTTGTTAAATCACTTCGTTATTTCTTGTTTATTAGGTAGCTCCTCAAAATCCGAGGAAAAATCTTGTGAAGTAAGATAAAAAGTTTCAAGATATATTGTTCAATTATTTCAAAAGAAATTATTTCGAATGAAAAAAATTTAATTTACTAATATAAAAATATTGTGATAAAAACCATGTTATGTTTGATATATAGACCTTAATTTAACATATGAATAAATAAAATAAGTGTATGATTATTAACTTATTACAATCAATTCTAATAATTATTTAATTTTTGTCACTTCAAGTTAAATTAATTTTAATTTTAATTCAAATAAATATATTTTTATAGGCAATGTATCAAATATATATGATATGCATGTTAATCTCATTTGACTTGATAGTTTTTTAATTATATTATTGATAGTAAGTAATCAATTATAAATATTGAATTGTTTGCCAATTAATTAAGCTAATCTCTATTAACTAATTAAATATATTTTATTCAGAGACATTTTTAAAAATAGCAAAATAAATTAAAATATTTGCAACCTATAACAAAATTTTGGATTATAACAATAATAGTTTTCTATTACTATAACATATCAATGATTATTAGTGGTAGAATTTGTTATTTTTAAAAACTCCCCTTTTAGTTAAGGAGAATCGATATAGATAGTAAAAAACATTAAAAAAAAAAACTATTTACAAATTTTTATTAACTAATTAAATTTACGAAATTTCTTTAAAATAGCAAATTTTACAAAATATTTACAATCTAGCAAATTCTATCACGGATAGTCATTGATATGCTATAATGATAGAGTATTATCATTATGATTGATAGAATCCAAAATTTTGTAAATATTTTAACTTATTTTGCTATTTTAAAAAATGTTTTTTTAAATATATTTATTTTAATAAATATTTACTCTAAATAATTTCAATATTTTACTATTTTTAAAATGTTTCTATATAAAATATACTTTTAGCTAATTAATTAATAATTTTGTGCATGAGTTATTGCATTCATTTAATAAACTTTTATTTGTAAAATTGAATCGAGTGAATATTTGAATGATTTGGATGATCAAAATCTACAATTATATTAATTTTGGTGAATTAATTTGAAGAAGTTATTTAATATAATTGTTATTTGCTCGCTCAGTTCATGACATGAAATTGTTATCCTTTGCGGTGGACAATACTTAATAATTTTAGTTCCAAATATGTAATCTTTACAGGAAATCTAGAAAAAAATAAATACATTCTCAACTTTATTATTTAACATAATATATATTCGTCAG

GTTGAGGTTCTACTTACGGGAGATGGTTCTGAGTTGTTTCCTAATCTTAAAGAATTGACGTTATATGGTTTTGTTGAGGATAACTCAACTCATTTGCCAGTGGAAATTGTACAAATCTTATACCAACTTGAGCACTTTGAATTGGAAGGAGCGTATATTGAAGAAGTTTTCCCCAGCAATATATTGATTCCAATGAAAAAACAGTACTATGCAAGATCTAAGAATTCAGTGCGTAGTTGGTTTCTATCTAAACTACCCAAGCTTAGGCATTTGTGGAGTGAATGTTCACAAAAGAATGCTTTTCCAATTCTACAAGATTTGAATGTAATAAGAATTTCAGAATGTGGTGGGTTGAGTTCCTTAGTTTCGTCATCAGTATCCTTTACAAACTTGACAGTTCTTAAAGTGGATAAGTGTGATAGACTAACCTATTTGTTGAATCCTTTGGTGGCTACAACCCTTGTGCAACTTGAAGAATTGACTTTAAGAGAATGCAAAATGATGAGTAGTGTAATTGAGGGAGGATCAGCTGAAGAAGATGGAAATGAGGAGACAACCAACCAGATTGA

GTTTACTCATTTGAAGTCTTTATTTCTAAAGGATTTACCACGACTACAAAAGTTTTACTCTAAAATTGAGACATTTGGTCAATTAAGCCGTGATAATTCCGAAAACCCTGAAACAACCACAATTCACAATCGCATTGGTGATTCATTTTTCAGTGAACAGGTGAACTCATAAATTCCAACACACTTCTTTCTGTATATGTACATCTACACACATCCAATCTTTTTTTCTTTTTATTGTTCCTCTGAATGACTTTTCTTTTCTTTTCTTTTCAGAAAACATTCTTCTTCTTTTTTTTTCAATGCAGAAATTTAAAATAAAAATTTCTATACACTGCTAGAACTTACAATATAACTGATCATCTCTCTTCAAACAATGCTGATGATGTTGTTAATTCATTGTATTGATAATGATGGTTATGTCGCCCCAACTGAATTTTCATGTATATGTAAATTTGTAGGAATCACTTCCTAATTTGGAGACATTGAG

AATTGATGGTGCAGAGAATTTGAGGATGATATGGAGTAATAATGTACTCATTCCTAATTCCTTTTCCAAACTCGAGGAAGTAGAGATTTATTCATGCAACAATCTTCAGGACGTATTATTTCATCCAAATATTATAAACATGCTTACATGCCTTAATACATTAAGGATTAAAAATTGTGAATTATTGGAGGGGATATTCGAAGTGCAAGAGCCGATTAGTGTTACAAAAACAAAAACAAATGCTATCGTGCTACCAAATAATTTGATAGAGTTGGAATTATATAATCTTCCAAACCTTGAGTACCTATGGAGTAAGAATCCAAATTTTGAACGGCTCGTGACTTTTGAAAGTATAAGAAGTTTGTCCATTGAAAAATGTTCAAAACTCAAAGGAGAATATTTTTTGTCAATCAAAACTTTCAAGCAACTTGTAAGACTGAAAATGGGTATTAGGCAATTGACAGTGGCTCTTGGGAAGGAAGTTAAGTCAGCAGATCATAGTATGTTATTGGAACCAAAGCAATTGGAGACTTCTTCTTCTAAGGTAAGTATATATTCTACAACACATCATGTTTATTCAATATTTCAGAAAATAAATTGTTATCATGACCTTAATTAG

> mrna34024.1-v1.0-hybrid

ATGGCGTTGAGTACCCAAGTCAGAGCTTCCACCGGTTCAGCTTTCTCATGGAAATACGACGTGTTTTTGAGCTTCCGAGGCGAAGACACCCGGAAGGGCTTCACTGATTACTTATACGACAAGTTGAAGTGGCGAGGAATCAGGACTTTCAGGGACGACCCTGAACTTGAAAGAGGCACAACCATCTCTCCGGAGCTCATCTCAGCAATCGAGCAGTCCAGGTTTGCGATCGTTGTTCTTTCTCCAAACTATGCTTCTTCCACGTGGTGTTTGCTTGAACTGTGTATTATTTTGGATTGCATGGAAAAGAGAGGGACCATTCTGCCGGTCTTTTATGAGGTCAACCCGTCTGATGTGAGACACCAAAGGGACAGCTTTGCTGAAGCGTTTGATGAACATGAAGAGAAGTTTGGAGAAGATAGTAAGGAAGTGGAAGGGTGGAGAGATGCTTTAACACAAGTGGCGAACCTCGCTGGCTGGAATTCAAAGGATTACAG

GTAAGTCTCAATTGTTGATTCAGAGATCAAATGACACGTAAATTGAAGTACACCAATATTTAGAAGTTATATAATGTTGGTGAGTTAGTTTAGGAAGTGCTTACACTGCCCTGCATCTTTGTATAACACCACTTTAATGTTGCGGTTCAGTTCTATCATACGGGTAGATTATATATTTTGTATGATCACATATTCAACATGAGAATTCTTATATTCTTCTTGTAG

GTATGGAACAGAGCTTATCAAAGAGATTGTGCAAGCACTGTGGAGCAAAGTGCATCCTAGTGCCACACTATTTGGTTCCTCAGAGATGTTAGTTGGAATGGATACTAAGCTGGAGGAATTAGATGTTCTTTTAGATAAAGAAGCAAATGATGTTCGCTTTATAGGGCTATGGGGAATGGGTGGGCTGGGTAAGACAACTCTTGCTAGACAAGTTTATGAGAAGATCTCTGATCAATTTGAAGTTTCCATCTTTGTTTCTAATGTCAGAGAGGTTTCTGCAACCCATGGTCTAGTTCATATACATAAGCAGATTCTGTCCCAAATCTTGAAGCAAGAAAATGAACAAGTATGGGATGTTTATAGTGCAATGAATATGACTAAGAGATGTTTATGCAATAAAGCGGTTCTTCTAGTTCTTGACGATGTGGATCAAGTAGAGCAACTGGAAACCTTGGTGGGAGAAAAAGACTGGTTTGGTATGAGGAGTAGAATCATCATTACAACTAGAAATCAACGTATACTAGTCACACATGGTATAGATGAACCATATGAGTTGAAGGGATTAAACAATGATGAAGCTCTCAAGCTCTTTAGTTTGAAAGCCTTTAGGAAATATGAGCCAGAAGAAGATTTTGCAGAGCTCTCTAGGTGCTTTGTTAGCTATGCCAGCGGTCTTCCCTTAGCTCTTAAGACTTTGGGGTCTTTCTTGTATAAAAGAAATGCAGATGCATGGATTTCTGCATTGGAAAAACTACGGAATACTCCCGACGGAACAATCTTTGACATCCTGAAAATAAGTTATGATGGACTCGATAAGATGGAGAAGAAAATTTTTCTGGACATCGCCTGTTTTTCTTCGGAATGTCAAGCCAAGTTTGTTATTGAACTACTGTATAGTTCTGACATTTGCACTCGTATTGCAATAGATGTTCTTGTTGAGAGATCTCTCTTAACTTTTTCATCAGACAATGAGATAGGTATACATGATTTGATACAAGAAATGGGACGTGAAATTGTACGGCAAGAGTCTTATGAAGAGCCTGGTGGACGCAGTCGTTTGTGGCTTCACAAAGACGTTTTTCATGTTTTCACCAAGAATACG

GTAAGATTTTAAACAAGTCTAATCACCAAGCTTGGATAGCACTGTCATTTATTTAACATGAATTATTTTTGAAATGACATATCTGTTGTAATAG

GGAACAGAAGCCATCGAAGGCATATGGTTACACTTGCCTGAATTAGAAGAAGCAGATTGGAATCATGAAGCCTTCTCTAAAATGGGTAAACTGAAGCTTCTCTACATTCATAACTTGAGGCTTGCTCTTGGCCCTAATTGTCTTCCTAATGCCTTGAGATTTCTGAACTGGAGTTGGTATCCTTCAAAATCTCTCCCACCAAGTTTTCAACCGGATGAACTCACTGAACTTAGCCTTCCCCATAGCAATATTCGTCACCTCTGGAATGGAATGAAA

GTAAGTTATCGAATATCTGTTATTTGCACAAGTATTATTAAATTTTTAGAGTTCAAGCATGAAGCTGATTCTTCTACCATTATTCATATTTTCTACAG

TGCTTGGTCAAGTTGAAATCTATCGACCTTAGTTACTCCATAAACTTGACAATGACCCCAGATTTCACAGGCATTCCAAACCTCGAGAGGTTGGTTTTTGAAGGTTGTACGAATTTAGTTAAGGTTCATCCATCCATTGCATTGCTGAAGAGACTCAAAATTTGGAACTTTAGATACTGCCGAAGTATCAAGAGTCTACCAAGTGAAGTGAAAAATGGAATATCTTGAGACTTTTGATGTATCTGGCTGCTCAAAACTAAAAATGATTCCAGAATTTATGGGGCAAATGAAAAGATTATCCAAGCTTTCTTTAAGTGGGACTGCTGTTAAAAACCCGCCTTCATCAGTTGAGCGTTTCAGTGAGAGTTTGGTGGAGCTTGATTTGTGTGGAATTGTTGCAAGAGAGCGGCCATACTCTCATTTTTTATCTTTTGGCTTATTTCCGAGAAAGAGTCCTCACCCTTTGCTTCCTCTGTTAGCTTCCTTGAAGCATTTGTCTTCTTTGACGTCATTAAAACTGAATGACTGTAACCTCCGTGAAGGAGAAATTCCGAATGAAATTGGTTCTCTGTCCACCTTAAGGGAATTGGAACTCGGAGGAAATAATTTTGTCACCCTTCCTGCCAGCCTTCATTTGCTTTCTGATCTGGAATACATTAGCGTGGAGGATTGCAAAAGGCTTGAGCAATTGCCAGACCTCGCATTCAATGACAGGTTATGTGTAGTAACCAGCAATTGTACTTCCTTAAAAGTGTTTCCAGATCCATCAGATTCGTGCAGATTCAGTACGGTTGGTAATCTGGATTCAAGTTATTTTTTATATTCAATGCTGAAGCGGTTACTTGAGGTAATCTGGATTCAATTTATTCTTTAAAGTTTTTATGTTCAATTATTGGTATTCATATCAATAATTATGATGGTTCATATGGTGCAGGAAGCCCCTTCTTTTCGTTTCTTCTGTTGTGTACTTCCTGGAAGTGAAATGTTTGAGTGGTTCAGTAATCAGAGTTTGGGAGACACGGTAACTGAGAAGATACCTCCGTATTCAAGTAATAGCAAGTGGATTGGGTTTGCTGTATGTGCGCTATTTGCAGCTCATGACAATCCATCTGCCGTTTCCGAATTCCCTTATTTACATCCTGGTCAAAGTTTGATTACGTGTTCATTTGATGGCCACGTGTTTTATGGACCACCTGTAAAACAAATTGGTTCAGATCACCTATTGCTATCTATTTTCTCCGCTCGTTACTGGAAGCCTGAGAACTGTCCAGATGACAACTTGA

>ppa016994m

ATGGATATTGTTATTTCAATTGCTTCCAAAATTGCAGAGTCCTTGGTGACACCAATAGGCAGAGAATTTGGCTATTTGATTTACTATGATACCAACATGAAAGATCTAAAGGATGAACTTAAACAGCTTTTTGAGATGAAAGATGGGGTGCAAGAATTGGTCAATGCTGCCAAAAGGAATGGTGAAGTTATCAACTCTGATGTTCAAAGTTGGCTAACAAATGTTAACAAGTTAATTCAGAAAGTGTCGCATTTTGAGGAAGAAGTCAACATGAAAAGGCGATTCTTGTACCGGTGGAACATAAGTAGGAAAGCCACTAAGATTACACAAGATGTTCGTCATCTCCAAAAAGAAAGAACATTCAATAATAATGTTGCCCATCCTGCACCTCCACCGATGATATGGTCAACATTCAAAGAAGGTTTTAAGGATTTTAAATCCAGAATGACATGTGTGAATAGCGTGATAGAGGTTTTGAAGAATGAAGAAGTTAGAATGATTGGGATTTGTGGTATGGGGGGTGTGGGTAAAACCACAATGGTGAAAGAAATCATCAAAAGATTGGCAGGATTGAAAGTATTCGATAATATTGTGATGGCAGTTGTGTCCCAAAGCCCAAATATTCAGAAGATTCAATCAGAAATTGCAGAGGAGCTGGGGTTTAAATATGACGAGAATACCGAAAGTGGAAGAGCTCGAAGGCTCTACGGAACACTCATGGAAATAAATAGGATCTTGATTGTATTAGATGATGTGTGGACAGAGCTCGATTTTGAGGCTATAGGACTTCCTTCTGGACTTACTCATAAAGGGTGCAAAGTTATGTTGACCTCACGAAACTTGGAAGTGTGCAATGCAATGGGAAGTCAAGAAATTTTTACAATCCCAGTTTTAACACCAGAGGAATCATGGGAACTCTTCCGTGAAATCATAGGTAAACCCTTGGACTATCCTGATTTGGCGAAACGAGTCATGAATGAGTGTGCAGGTTTACCTATTGCTATTTTAACTGTTGCGAAAGCACTAGAAAATAAGAGGAAGTATGAATGGGATGATGCCCTCAAACAGCTACAAAGTTCAGCTCCTGGAAGCATCTCTTCGATGAATGACAGAGTTTATTCAAGCATACAATGGAGTTATGATAGATTGGAGAGTGATGAAGCGAAGTCATGCCTTTTGCTTTGTTGTTTATTTCCTGAAGATTATGATATTCCAATTGAGTATTTGGTTAGATATGGATGTGGTCGAGGGTATTTTAGTAACACTGATTCAGTGGAAGAAGCAAGAAATAGAGTGCATTCTTTGGTTGACAAACTTCAAAGAAGGTTTTTGTTGCTAGATAGCAAATTGAAAGACCATACCAAGATGCATGACATAGTTCGTGATGTTGCCATATCAATTGCCTCAAGAGATCCACATAGATTTCTAATAAGATGTGATGCTGAAAAGAAAGGGTGGCCAAAGATATACGACCATTACACAACAATCTCACTGATACCAATTAATATTGATGAGATCCCAGTTGGTCTGGATTGTCCAAAACTTGAGCTTCTACACTTAGAAGGTGAACGTTATTCGGAAAATAGCATGGACATCATGTGTAAAGGGATGAAAAAGCTGAAGGTTTTAGCATTGGTAGACGTGCGGGGGATTTCAGCCTTGCCAAGTTCACTAGGACTACTGAAGAGCCTTCGAACCTTGTCCCTAAATGGTTGTCGTTATTTAACAAATATATCTGATGTTATTGGAAGATTGGAAAATTTAGAGATCCTCAGCTTTCGTCAATGCTCTAGAATCTTAAAGTTACCAAAGGAAATTGGACTTCTTAAACATTTAAGGTTGTTAGATATCACGGATTGTAATTGTCTTGAGAAGATTCCACATGGTCTCTTATCGAGCTTATCTAGTCTTGAAGAGTTGTATATGGAAAATAGTTTTTGCAAATGGGAACAATCAGCAGCAGAAAGCGAAGATAAGAGGATGGCAAGCCTTGTTGAGGTGATGTCTTTGTCTAATCATTTGAAGGTTTTAGTCATAGTTATACCAGATTTCAACTTCTTTCCAAAAGACTGTTACTTGACAATCCAGACAACAATAAGATTCCACATATCCAACAGAACGTTTCTACCATGGATTACAGGTATTCCATCTACAAGAACAGGTTGTTATGCATTTGAAAATAAGTTGGATATTGTTAGCAGTGATGCAACGGAATTCATGGAGATTCAAACTGTTCGACTCTTATTTAAGAAATGTGAAGATTTATACTTGCAAGAGATTAAGAATTTAAAGTATGTCCTCAATGAATTAGACCAAGAGGGATTGCAACACTTAAAAGTTTTAACAATTTCGGGCTGCCCTGAGATAGAATACCTTGTAAATGGAGCAAGTTGGACTCAACAAACAGCCTTCCCTCTCATCCAATCAATCCAACTCGAGTCGATGCCCGAGCTAAGAGCGATATGCCCCGACCAACTTCCACAGAGCTCTTTTATCAACTTAAGATCTCTTGAATTACATAATTGCCCCGTTTTAAAATATGTATTTTCTCTATCAGTAGCAAGCAACTTGGTTCAACTCCAAAGCTTACTTGTTGTCTTGTGTCCCCAAATGAAAGAAATCGTCTCAAAAGAGTGGAGGGAACATGAGACCGCCTCGGACATCATAGCTTTCCCTAAATTAATATATTTGATACTCCAAGGTCTATCTAATGAATTCGTTGGTTTCTACGAAGCCAACAAGCTATACTCCAACCATGAG

GTAACTTATTATTTCGATGCTTGAATAGTATAATTGTTTTGTTAATTGTTTGTGCCTCTATTTTTATTTTTGTTGGTTTACTTGCCATGAGGTCTGACTTTAGTTTGACTATTATTTACTCATTTTCTTCCTAAAAACTGAAAATGTTCTCATACTGATAAATGAGGAATTTAGTTTACTTGCCTTTACAAAAAATAAATAAATAAATAATTGAGCATGTGCAGAACACAAAGAAAATCATTCCATAAATATATCCATTCCATATATTTATTTTTTAAAAATTTGGATTCATTTTTGTTAATGGTCTCATTACATAATATATTTAGAAATCTCGAACTATCTAAAGAAGCAACCTGAATTTGTAAAAATTTTGGACTCATTTTTTAAGTTCGTGAAAACTTTCATGTATATATTTTCACCTGAATTGATTGTATTCGAGAAATCAAAGCTATAAACTACCGGTACAAGCTCCAAATTGTTTACTGGAAAATTCTTGCCACGTGGAAGCATTTAGTTTGACTTGCGACCACAAAGTCTAGCTTTTATATGATTATTATCTAGAAACTGCCTACTCAATATTATTTTAATATTTGGTTTTATTAATATTTTAATATGAAGTCATATTGGTAAGTTATTAATTTCATTTTTGAGAGGAAAATGTCTGAAGTTGATCTATTAATTAATTAATTAATCTTTTTAG

GTAACAACGCCTGAGAATCAAAATGTGGTAGGAACTTCATATGATGTGCATCAATCTTCTAGAAGCTTTGAAAGAGCAGTATTTCCATCAAAGTGCATTTTATGGTTACAAAATCTAGAAGAAGTGAAACTAGAATATTCCGATGTAGATGTGTTCTTTAATTTGAAAGGCCATATGGTTAGGGATGGGCAGGCAGTTCCAGCATTTTCTCATTTACAAAATTTGTTCATACGGGATTCTCGATGTCAACACCTTTGGAAGAACATTCCGCGTGGATTCCAGGGCTTCCAAAACTTAAGATATTTAAAAATACAAGATGGTGGTGATCTCCAGTATGTGTTCCCACATTCAATTGCCAGGCTACTTGTGAATCTTGAAGAGCTCAACATAGCGCAATGCTGGGAAATGGAAACTATAGTCAGATCTGCAGATGAAAATGAAAAAGAGGATCAGACGAGCATGACTTTGTTTCCCAAACTAAATAGTTTTGATCTACACTGGCTTCGGAGTCTTGAGAGTCTTTGTCCTGATGCTTCTACTTCTTTATGTTCAACCGCCAAAGTTATGTCTGTGAAAAGATGCGACAAACTGAAGACATTGGCTTCAGTAATTCCACAGATAAAAAAGTTGGAGAAAGATTCAACAGCTCATCACGAAGATGAAGACGAAGATATTTCATCAAGATCATGTGGATGTACACCTTATTCATGTGGCCCAATGACCAAACCAACTTCAAGAAGAAATATTGTTCAAATATTGCCACGTCCAGTCAATCAAGAG

GTAAGCATTTCAATTTATTTAGAGCCACAATTGAGAATGACAATTGACATTGCCATATTGAAATTATATAAATTATATACGGTTTACTCAATTGATGTTTGATTTTCTAG

GTTGCACCAACAAATCTTGATCAAGACTCAAACGATTACGATAATCTAGAACGTCTTTCTGTACAATCTTGTAAGTCATTGGAAGTTGTTTTCCAACTCAAGGGACCAAAGGCTGTGGAAAGCCACAATGTTCAAGCATTCAATAAGTTGTGTTACTTGTTGTTAAACAAATTGCCAAGTTTAATGCACGTATGGGAAACGGGCGGTTCGCCTCATATCACAGGCTTTGGAAACTTAACATTCTTAAGTGTATCCCACTGTGGCAGTTTGCGATATTTGTTCTTGTCAACTGTAGCCAAGCTCCTTATCAGTTTAAAGGACTTAAAAGTTGGGAACTGTCAGAAGATTGAACAAGTTATTGCAGAAGCAGATACTGAATGTGCTGACCAGGAAATTACATTTCGTCAACTGAATTCCATAACGCTTGAAGATCTACCAAACCTCATTTGTTTCTCTATTGAAGCTTATACTTTGAAGTTTCCATGTTTGAGGGAATTGAAGGTTATAAGATGTCCAGATTTAAGGACATTTGCTTCTAAGGTTGTCAACGCACATTCTGTAATCAAAGTACAGACAGAGTTCGGAAAGTCTGAGTGGATGGGGGACCTCAATAGCACTATAGGGAACATTCATGAAAAAAGGTATGCTCTTGAATGTTTAGTCAACAATATAACAATAAAAGTGTTTCTTTTTCAAACCTGA
